# Comprehensive Analysis Identified ETV7 as a Potential Prognostic Biomarker in Bladder Cancer

**DOI:** 10.1155/2021/8530186

**Published:** 2021-12-09

**Authors:** Haimeng Li, Yibo Zhang, Shangyong Zheng

**Affiliations:** School of Medicine, Yunnan University, Kunming, China

## Abstract

**Background:**

The tumor microenvironment (TME) plays a crucial role in the initiation and progression of cancer. Bladder cancer (BLCA) is a malignant tumor of the genitourinary system. Its heterogeneity results in significant differences in the prognosis of patients. To date, this is still a huge challenge for clinical treatment. In recent years, more and more evidence showed that dysregulation of transcription factors (TFs) plays an important role in tumor progression, invasion, and metastasis. Unfortunately, the role of TFs on the tumor microenvironment in bladder cancer is unclear.

**Methods:**

The original data of BLCA and corresponding adjacent tissues were obtained from The Cancer Genome Atlas (TCGA) database. TFs were downloaded from the Animal Transcription Factor DataBase (Animal TFDB). Intersection analysis was used to obtain TFs that were differentially expressed between tumor and adjacent tissues. Gene Set Cancer Analysis (GSCALite) and CIBERSORT software were used to reveal the key differentially expressed TFs (DE-TFs). Subsequently, UALCAN and Human Protein Atlas (HPA) databases were used to disclose the expression of key DE-TFs in BLCA. The *K*-*M* curve divulged the relationship between the key DE-TFs and the patient's overall survival (OS), and the univariate and multivariate Cox regression analyses were conducted to explore independent prognostic factors. The cluster profiler package and Gene Set Enrichment Analysis (GSEA) were used for functional enrichment of genes related to the key DE-TFs. Finally, CIBERSORT software analyzed the immune landscape of BLCA.

**Results:**

We obtained a total of 117 BLCA-related DE-TFs. Among them, *ETV7* was identified as the key DE-TFs due to its association with the autophagy activation pathway and various immune cells in cancer. Online databases of UALCAN and HPA indicated that *ETV7* was overexpressed in tumors and negatively correlated with tumor severity. The *K*-*M* curve showed that the OS of patients with high expression of *ETV7* was poor, which indicated that it was an independent prognostic factor. Functional enrichment of 87 DEGs between *ETV7*-high and -low expression groups indicated that it was closely related to the immune response and the functions of a variety of immune cells. Finally, CIBERSORT results proved that the high and low expression of *ETV7* also caused significant differences in the tumor immune microenvironment of patients.

**Conclusion:**

Overall, we proved that the transcription factor *ETV7* was a novel prognostic factor, which may improve the individualized outcome prediction in BLCA by regulating the tumor immune microenvironment.

## 1. Introduction

Bladder cancer (BLCA) is a urinary tract malignancy with high morbidity and mortality, which is reported as the 10th most common cancer with an estimate 549,000 new cases and 200,000 death in 2018 [1]. Bladder cancer is a heterogeneous disease which consist of two subtypes nonmuscle-invasive bladder cancer (NMIBC) and muscle-invasive bladder cancer (MIBC). NMIBC, the majority of the BLCA do not penetrate the detrusor muscle layer which is not fatal but have high potential recurrence [2].

Despite the widespread use of multiple BLCA therapies, including surgery, radiation, chemotherapy, and Bacillus Calmette-Guerin (BCG), these therapies have not significantly improved 5-year survival over the past decade [2]. To date, the prognosis of BLCA patients largely depended on the histopathological diagnosis and tumor grading system, which raised the risk of BLCA recurrence. Therefore, it is very important to find some latent prognostic biomarkers for BLCA.

Transcription factors (TFs) bind to DNA and regulate transcription in a sequence-specific manner to form complex systems that direct genome expression. TFs are crucial for tumor progression, metastasis, and cancer metabolism through a wide range of physiological regulatory processes. Recent studies have shown that E2F1 is highly expressed in prostate cancer cells as an oncogene, and its expression level is closely related to the occurrence, development, and poor clinical prognosis of prostate cancer. EWS/FLI (TFS) binds GGAA-microsatellite sequences in regulation of the NROB1 gene as well as for Ewing sarcoma proliferation and anchorage-independent growth [3].

The E26 transformation-specific (ETS) family is one of the largest transcription factors involved in many biological processes, including cell differentiation, cell cycle control [4], cell migration [5], and cell proliferation [6]. This family consists of 28 human TFs that bind to similar DNA sequences of MAPK [7]. Multiple studies have shown that the abnormal expression in many members of the ETS family is closely associated with tumor initiation, progression, and metastasis in cancer [8–10].

In addition, the ETS family has been proved to be related to a wide range of immune-related biological process [11–13]. ETS-1, ETV5, PU1, GABP-1, and ETV6 have been shown to play critical roles in T cell differentiation [12]. ELF4 inhibits the proliferation of naive CD8^+^ T cells by enhancing the KLF4 expression [12, 14]. It is reported that ETV7 regulates the immune microenvironment in melanoma [15]. And it is an essential component of a rapamycin-insensitive mTOR complex in cancers [16].

Tumor immune microenvironment (TIM) has been attracting interests over past years. The dysregulation of the immune microenvironment promotes the malignant progression of BLCA from NMIBC to MIBC. Interestingly, immunotherapy has played an important role in the treatment of BLCA, and Bacillus Calmette Guerin (BCG) remains to be the most efficacious intravesical medicament for NMIBC but with strong effects [17]. Therefore, it is very important to find a biomarker for bladder cancer tumor microenvironment.

In the current study, we systematically analyzed the expression profile and prognostic significance and role of ETV7 by integrating data from TCGA database, Animal Transcription Factor DataBase (AnimalTFDB), UALCAN, and Human Protein Atlas (HPA) databases, and our results indicated that *ETV7* was a novel prognostic factor, which may improve the individualized outcome prediction in BLCA by regulating the tumor immune microenvironment.

## 2. Materials and Methods

### 2.1. Data Source

The database of The Cancer Genome Atlas (TCGA) was consulted to extract the RNA-seq data (FPKM format) and respective clinical data for a total of 408 BLCA patients and 19 adjacent samples. Clinical parameters, including gender, age, pathologic stage, pathologic T stage, pathologic N stage, and pathologic M stage, were also evaluated. 1665 human transcription factors were from the AnimalTFDB were posted on Supplementary Table S1 [18].

### 2.2. Differentially Expressed Gene (DEGs) Analysis

The *R* package limma was utilized for gene expression data analysis [19]. Genes satisfying ∣log_2_ fold change (FC) |  of > 1 and adjust (adj.) *P* < 0.05 were perceived as DEGs in BLCA vs. adjacent tissue and high-*ETV7* vs. low-*ETV7*. For the volcano plots and heatmaps, the ggplot2 and pheatmap packages were employed to derive them, respectively.

DE-TFs were obtained through the intersection analysis of DEGs (BLCA vs. adjacent tissue) and TFs and then included in the univariate Cox regression analysis combined with the *K*-*M* method. With *P* < 0.05 as the threshold to obtain candidate DE-TFs.

### 2.3. GSCALite

GSCALite (http://bioinfo.life.hust.edu.cn/web/GSCALite/) was applied in the present study to provide a web-based analysis candidate DE-TFs and cancer pathways. This includes differential expression and survival analysis, the correlation between gene expression and cancer pathways, and other analyses [20]. All the candidate DE-TFs were uploaded to the GSCALite website with the aim of analyzing the correlation between cancer pathways and candidate DE-TFs using the TCGA-BLCA samples (*N* = 414).

### 2.4. The Human Protein Atlas (HPA)

The HPA is mapping all the human proteins in 64 cell lines, 48 human normal tissues, and 20 tumor types [21]. The key DE-TF, *ETV7*, was submitted to the HPA database, and the protein expression in BLCA was explored. The tissues information used in this study were as followed: patient ID: 1761, male, age 51, urinary bladder (T-74000), normal tissue, NOS (M-00100); patient ID: 2053, male, age 51, urinary bladder (T-74000), lymph node (T-08000), urothelial carcinoma, and high grade (M-812033).

### 2.5. UALCAN

UALCAN is a web resource designed to analyze cancer transcriptome data in an interactive way [22]. In this study, *ETV7* was presented to UALCAN, and the expression of its mRNA in TCGA-BLCA samples was investigated. Moreover, any correlation of the ETV7 expression with the clinicopathological features was also estimated. *P* < 0.05 suggested a meaningful variation.

### 2.6. Independent Validation of ETV7 Expression Levels in Multiple Databases

To enhance the reliability of the results of this study, we opted for three different databases, namely, the Gene Expression Omnibus (GEO, http://www.ncbi.nlm.nih.gov/geo/) database, the online Metabolic gEne RApid Visualizer (MERAV) database (http://merav.wi.mit.edu/), and the tumor immune estimation resource (TIMER) online database (http://timer.cistrome.org/) to further validate the differences in ETV7 expression between BLCA and normal tissues. For the GEO database, we downloaded the GSE7476 dataset (https://www.ncbi.nlm.nih.gov/geo/query/acc.cgi?acc=GSE7476) containing 3 normal bladder samples and 9 BLCA samples. Based on RNA sequencing data, the difference in the ETV7 expression between normal tissues and BLCA was detected by the Wilcoxon test method. The publicly available website, MERAV, was designed to analyze the human gene expression in 4454 arrays and normalize all arrays together to generate a database [23, 24] for the ETV7 expression differential validation. TIMER is a comprehensive resource for systematic analysis of immune infiltration of different cancer types [25]. Not as well known, the database also has cancer exploration capabilities, which can detect gene expression differences between tumor and normal groups.

### 2.7. Enrichment Analysis

Gene Ontology (GO) and the Kyoto Encyclopedia of Genes and Genomes (KEGG) were administered in clusterProfiler to enable enrichment analysis for all DEGs between the high-*ETV7* and low-*ETV7* groups. Furthermore, all the genes in the high-*ETV7* and low-*ETV7* groups were submitted to the GSEA and chose the C5 (c5.go.v7.2.symbols.gmt) and C2 (c2.cp.kegg.v7.2.symbols.gmt) subcollection downloaded from the Molecular Signatures Database (http://software.broadinstitute.org/gsea/msigdb/index.jsp) as the reference gene sets.

### 2.8. Assessment of Tumor-Infiltrating Immune Cells

With the CIBERSORT algorithm, the fractions of infiltrating immune cells in each BLCA sample were derived for the high-*ETV7* and low-*ETV7* groups. CIBERSORT is designed to calculate the cellular composition of complex tissues working from the standardized gene expression data [26]. This method energizes the abundance of specific cell types [27]. The results generated by CIBERSORT were regarded as accurate when *P* < 0.05 was met [28].

### 2.9. Statistical Analysis

In this study, statistical analyses were carried out using the *R* package. The correlation between 13 candidate DE-TFs and immune cells was revealed by Pearson correlation analysis. The log-rank test was invoked in the *K*-*M* survival analysis. Independent factors were established by univariate and multivariate Cox regression analysis. All *P* < 0.05 made a significant difference statistically.

## 3. Results

### 3.1. Screening of DE-TFs in the TCGA-BLCA Database

**Figure 1:**
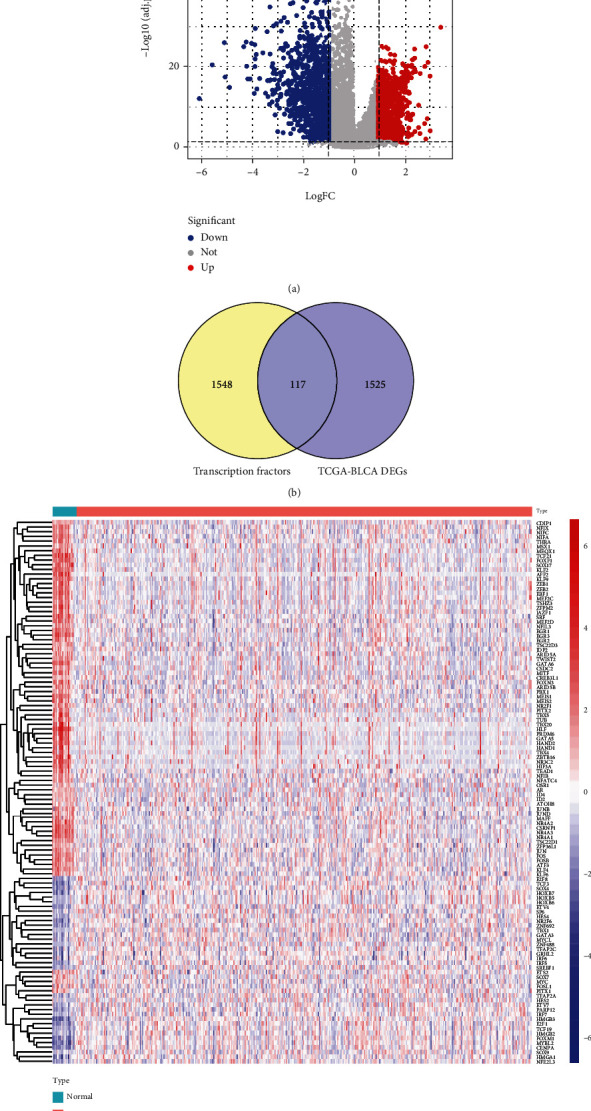
Distribution and intersection of DE-TFs in bladder cancer. (a) Distribution of the DEGs in the TGCA-BLCA database. (b) Venn plots showing 117 common DE-TFs shared by TFs and TCGA-BLCA DEGs. (c) Heatmap shows the expression levels of 117 DE-TFs, the red indicated tumor sample, and the blue indicated normal sample.

The analysis of the TCGA-BLCA database and comparison of the BLCA group with a normal group identified 1663 (759 upregulated and 904 downregulated) DEGs. The distribution of the DEGs in the TCGA-BLCA database was shown in Figure 1(a). The upregulated and downregulated DEGs of the TCGA-BLCA database were shown in Supplementary Tables S2 and S3, respectively. A total of 117 DE-TFs were obtained by further analysis of the DEGs and 1665 TFs (Figure 1(b)). All of the DE-TFs were displayed in Supplementary Table S4. Also, we showed the data by a heat map (Figure 1(c)).

### 3.2. The Key DE-TF Screening of BLCA

**Figure 2:**
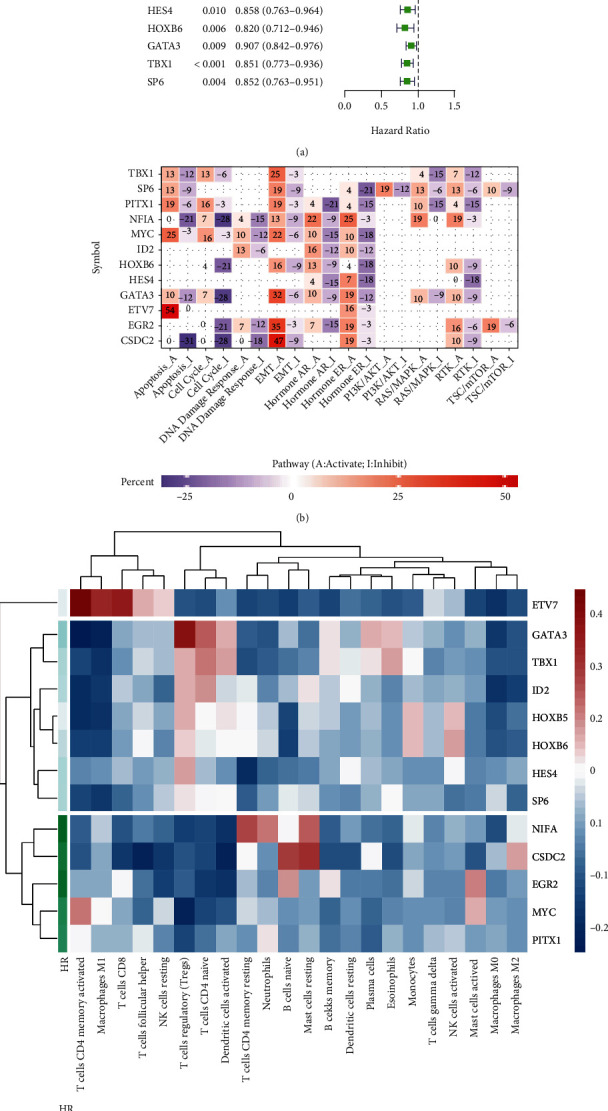
Screening of 13 TFs associated with immune infiltrating. (a) Univariate Cox analysis with 117 DE-TFs listing the top significant factors with *P* < 0.005. (b) The correlation of 13 candidate DE-TFs with various cancer pathway. The higher the absolute value, the stronger the correlation with this cancer pathway. (c) The correlation between the expression of 13 DE-TFs and immune infiltrating cell. The color represents the Pearson correlation, and the value represents the strength of the correlation.

We explored the relationship between the DE-TFs and OS by utilizing univariate Cox analysis combined with the *K*-*M* curve. Of note, among the 117 DE-TFs, 13 met the criteria for statistical significance via the *P* < 0.05 (Supplementary Table S5). *NFIA*, *EGR2*, *CSDC2*, *MYC*, and *PITX* were considered risk factors (HR > 1), and the remaining 7 DE-TFs were protective factors (HR < 1) (Figure 2(a)).

To learn more about the potential worked of the 13 candidate DE-TFs in BLCA, we first analyzed the relationship between DE-TFs and the activities of various cancer pathways using the GSEALite website. It was apparent that *ETV7* played a more prominent role in apoptosis activation. Also, we found that *CSDC2* was related to activation of the EMT pathway and related to inhibition of the apoptosis and cell cycle pathways (Figure 2(b)). Positive numbers represented activation and negative numbers represented inhibition. The higher the absolute value, the stronger the correlation with this cancer pathway. A correlation between the expression of 13 DE-TFs and immune infiltrating cells was also evaluated, which showed that the most DE-TFs had a strong correlation with immune infiltrating cells. Strikingly, the cluster analysis showed that *ETV7* was unique and had the strongest positive correlation with T cells CD4 memory activated. Coincidentally, there are also inextricable links between autophagy and T cells CD4 memory activated [29–31] (Figure 2(c)). The color represents the Pearson correlation, and the value represents the strength of the correlation. In summary, *ETV7* may play a vital role in the occurrence and development of BLCA. We regarded it as a key DE-TF and carried out further research.

### 3.3. Profiles of *ETV7* Expression in BLCA

**Figure 3:**
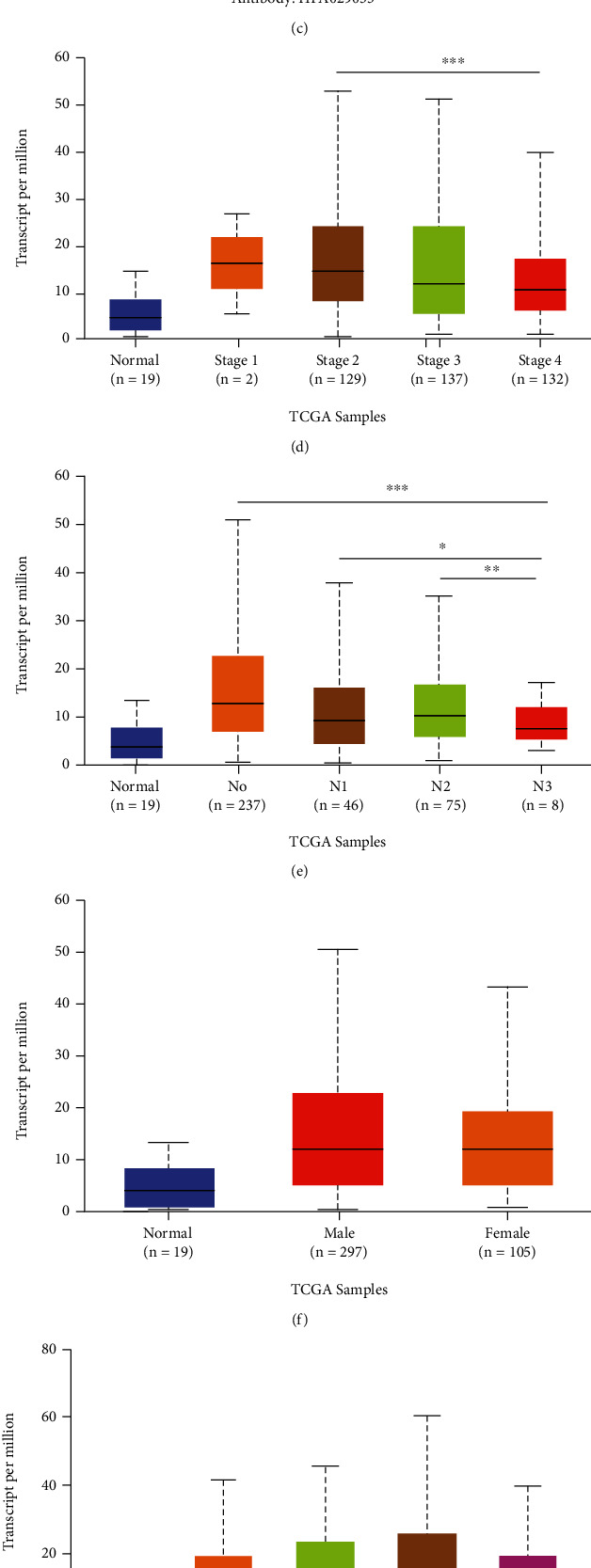
ETV7 differential expression in BLCA tissues. (a) ETV7 expression in tumor and normal samples. (b) The mRNA expression pattern of ETV7 measured by UALCAN database. (c) Immunohistochemical levels of ETV7 in BLCA samples. (d)–(h) Correlation between expression of ETV7 and clinicopathological staging characteristics.

BLCA tissues exhibited a significantly higher level of the *ETV7* (*P* = 5.86*E* − 05) expression (Figure 3(a)) than normal tissues. Naturally, the expression pattern of *ETV7* mRNA was characterized by the UALCAN database. As was shown in Figure 3(b), the mRNA expression of the key DE-TF was found to be significantly upregulated in primary BLCA tissues compared to normal samples (all *P* < 0.05). Furthermore, analysis of the dysregulated *ETV7*, using HPA, displayed increased protein levels in BLCA, with respect to specimens from normal controls (Figure 3(c)). Aggregately, a robust affirmation of the importance of *ETV7* in BLCA was noted in these findings.

Following the discovery of mRNA and protein overexpression in BLCA patients, we next analyzed the relationship between the mRNA expression of *ETV7* with clinicopathological parameters of BLCA patients by UALCAN, including a patient's gender, smoking habit, tumor type, tumor stage, and N stage. As was shown in Figure 3(d), the mRNA expression of *ETV7* was remarkably correlated with patients' cancer stages, where patients in advanced stages of cancer tended to express lower levels of *ETV7*. The highest expression of *ETV7* was found in stage 1. Similarly, as was illustrated in Figure 3(e), the *ETV7* expression levels were significantly related to N stages, and, as the N stage increased, the expression of *ETV7* tended to be lower. The highest mRNA expression of *ETV7* was noted in the N0 stage. The reason why the mRNA expression of *ETV7* in the N1 stage seemed to be lower than that in the N2 stage may be due to the small sample size (only 46 BLCA patients were at the N1 stage). However, the expression level of *EVT7* had no significant correlation with the patient's gender, smoking habits, and tumor type (Figures 3(f)–3(h)). These results suggested that *EVT7* was significantly associated with clinicopathological parameters in BLCA patients.

To ensure the accuracy of the results, we further examined the expression differences of ETV7 in BLCA and normal groups from the GSE7476 dataset, MERAV online database, and TIMER database, respectively. Consistent with the results in TCGA database, as shown in Supplementary Figure 1, all three independent external databases indicated that ETV7 was significantly overexpressed in BLCA (P <0.05).

### 3.4. Prognostic Value of *ETV7* in BLCA

**Figure 4:**
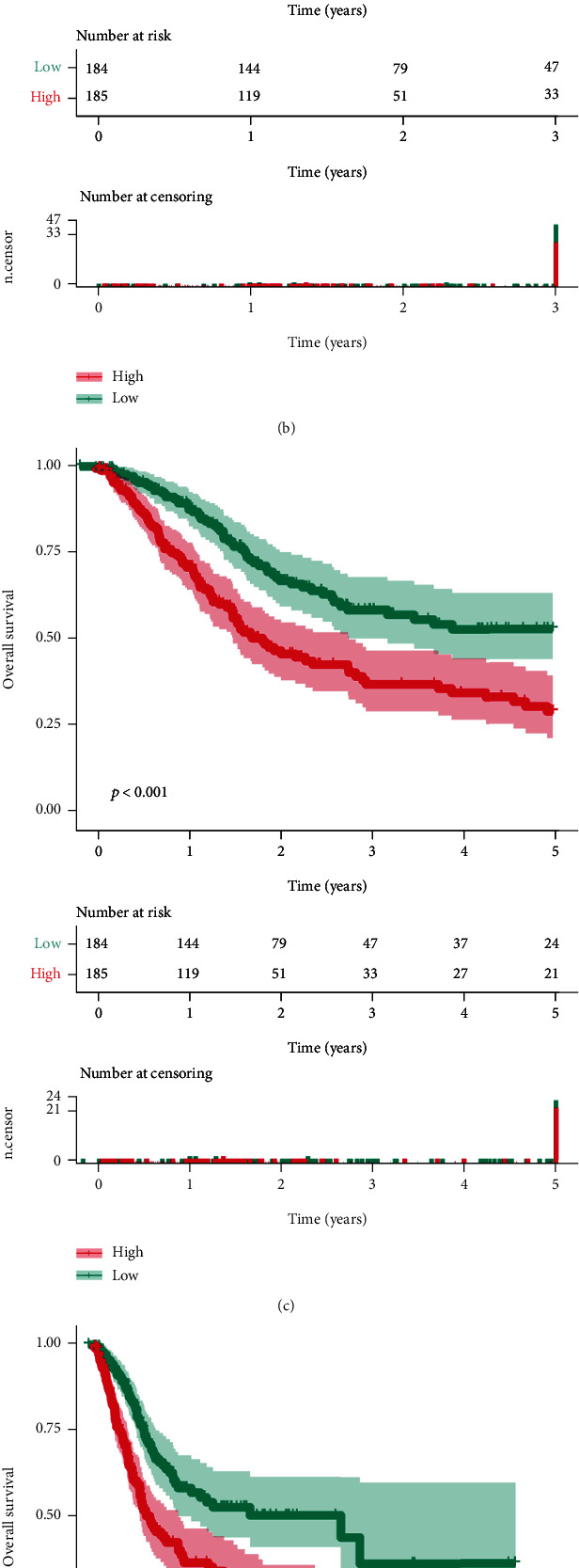
Prognostic analysis of ETV7 in BLCA patients. (a)–(d) The association between ETV7 and the prognosis of BLCA patients. (e, f) Univariate and multivariate Cox analysis of clinical characteristics and the ETV7 gene.

The association between *ETV7* and the prognosis of BLCA patients was subsequently reviewed. The 1-, 3-, and 5-year *K* − *M* curves showed that there was a significant difference in survival between the high and low expression groups of *ETV7* (Figures 4(a)–4(c); all *P* < 0.001). OS analysis revealed that BLCA patients with a high level of *ETV7* (*P* < 0.001) had a poor OS rate (Figure 4(d)). Thus, *ETV7* was a potential prognostic biomarker for BLCA.

The interaction among clinicopathological variables, *ETV7*, and OS was primarily adjusted by a univariate Cox regression model, following which, and salient variables (*P* < 0.05) were employed for multivariate Cox regression model estimation. In the univariate Cox analysis, we limited this to include gender, age, pathologic stage, pathologic T stage, pathologic N stage, pathologic M stage, and *ETV7*. Age (*P* < 0.001), pathologic stage (*P* < 0.001), pathologic T stage (*P* < 0.001), pathologic N stage (*P* < 0.001), pathologic M stage (*P* = 0.031), and *ETV7* (*P* = 0.004) were significant but gender was not (*P* = 0.299) (Figure 4(e); Supplementary Table S6). In the multivariate analysis, age (*P* < 0.001), pathologic T stage (*P* = 0.007), pathologic N stage (*P* = 0.007), pathologic M stage (*P* = 0.034), and *ETV7* (*P* = 0.019) were all remained significant (Figure 4(f); Supplementary Table S7).

### 3.5. Functional Enrichment of DEGs between *ETV7*-High and -Low Expression Groups

**Figure 5:**
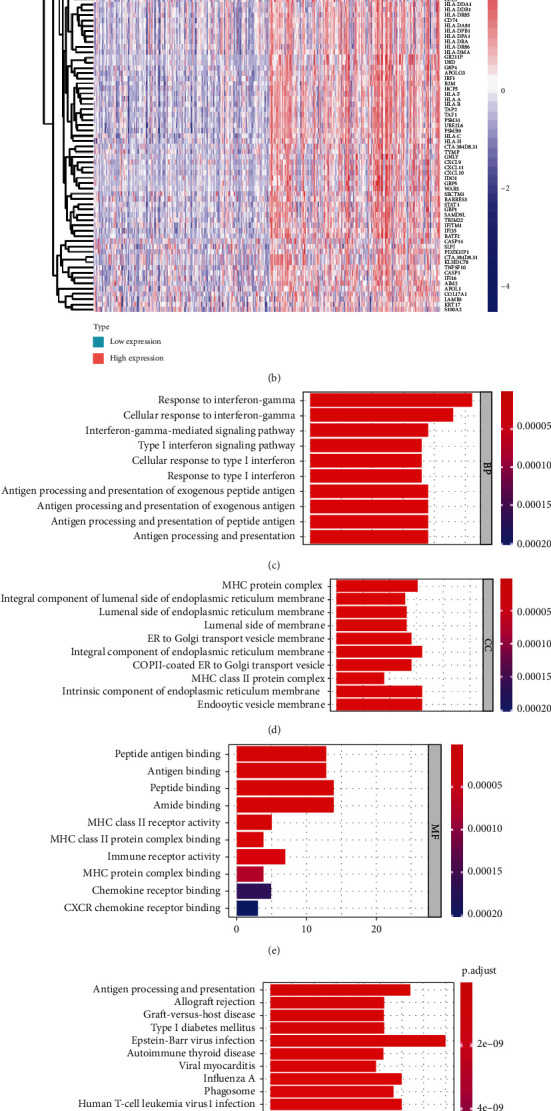
Functionnal pathway enrichment of ETV7. (a). distribution of the DEGs in the low and high ETV7 groups. (b). heat map shows the expression levels of 87 DEGs in the high and low ETV7 groups. (c–e). GO analysis of ETV7 related genes 5F KEGG analysis of ETV7 related genes. (g). GSEA plots of “antigen processing and presentation,” “natural killer cell mediated cytotoxicity,” “JAK-STAT signaling pathway,” and “cytokine-cytokine receptor interaction”.

DEGs between high- and low-*ETV7* expression groups were screened by adj.*P* < 0.05 and ∣log_2_FC | >1. A total of 87 DEGs (82 upregulated and 5 downregulated) were identified in the high *ETV7* group (Figure 5(a)) and visualized using a heat map (Figure 5(b)). The detailed DEGs were illustrated in Supplementary Table S8.

GO and KEGG analysis was carried out using the clusterProfiler package to investigate the potential capabilities of the DEGs identified above. Significance (*P* < 0.05) was achieved for a total of 227 GO terms of biological process (BP), 51 GO terms of cellular component (CC), and 25 GO terms of molecular function (MF). Top 10 GO BP terms indicated that the DEGs were primarily enriched in “response to interferon-gamma,” “cellular response to interferon-gamma,” and “interferon-gamma-mediated signaling pathway” (Figure 5(c)). Primary terms within CCs included “MHC protein complex,” “integral component of lumenal side of endoplasmic reticulum membrane,” and “lumenal side of endoplasmic reticulum membrane” (Figure 5(d)). MFs ascribed to these DEGs included mainly “peptide antigen binding,” “antigen binding,” and several subterms of MHC (Figure 5(e)). Additionally, on KEGG analysis the DEGs were mainly enriched in “Antigen processing and presentation,” “Allograft rejection,” “Graft-versus-host disease,” and “Type I diabetes mellitus” pathways (Figure 5(f)).

Surprisingly, in the GO-BP classification, immune response regulation and various immune cells (such as T cells, granulocytes, white blood cells, monocytes, and lymphocytes) were significantly enriched (Supplementary Table S9). Meanwhile, KEGG pathway analysis also uncovered that “Th1 and Th2 cell differentiation,” “Th17 cell differentiation,” and “natural killer cell mediated cytotoxicity” pathways were closely related to DEGs (Supplementary Table S10). Next, with the GSEA analysis, we were able to profile the pathways of the high-*ETV7* expression samples. The results showed that high-ETV7 expression samples were mainly enriched in “antigen processing and presentation,” “natural killer cell mediated cytotoxicity,” “JAK-STAT signaling pathway,” and “cytokine-cytokine receptor interaction” pathways (Figure 5(g)). Moreover, GSEA-GO analysis also showed that a large number of immune-related terms were substantially enriched. Detailed results of GSEA were shown in Supplementary Tables S11 and S12.

### 3.6. Analysis of Immune Landscape of *ETV7*-High and -Low Expression Group

The enrichment analysis showed the DEGs participated in a variety of immune-related terms and pathways; so, we analyzed the tumor microenvironment of BLCA.

**Figure 6:**
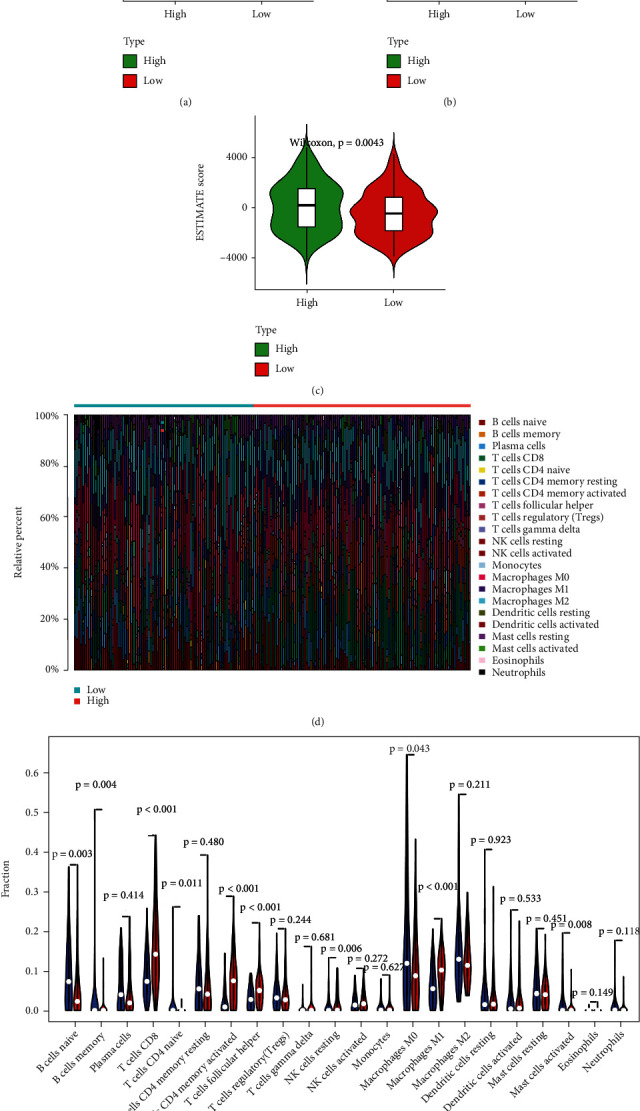
Immune landscape of ETV7 in various immune cells. (a)–(c) ESTIMATE algorithm immune, stromal, and estimate score. (d) Barplot showing the proportion of TICs in BLCA samples. Column names of plot were the sample's ID. (e) Violin plot showed the ratio differentiation of 21 kinds of immune cells between BLCA samples with low or high ETV7 expression relative to the median of ETV7 expression level, and Wilcoxon rank sum was used for the significance test.

ESTIMATE algorithm-derived immune scores ranged from -1658.36 to 3222.84 and stromal scores ranged from -2613.72 to 2173.23 (Supplementary Table S13). The average immune score of the high *ETV7* subtype was higher than that of the low *ETV7* subtype (*P* = 5*e* − 10), as well as the ESTIMATE score (*P* = 0.0043, Figures 6(a)–6(c)), which indicated that both scores were meaningfully correlated with *ETV7*.

The CIBERSORT algorithm demonstrated that tumors with high ETV7 were significantly associated with high fractions of T cells CD8, T cells CD4 memory activated, T cells follicular helper, and macrophages M1. In the low ETV7 group, there was a higher fraction of B cells naive, B cells memory, T cells CD4 naive, macrophages M0, and mast cells activated (Figures 6(d) and 6(e)).

## 4. Discussion

TFs are significant in modulating the progression and immune response of tumors. Unfortunately, the specific function of associated TFs in BLCA is rarely known. In this systematic analysis of TFs in BLCA, we first integrated the data from the TCGA and AnimalTFDB, and 13DE-TFs were found. In this current study, we correlate the 13DE-TFs to the immune infiltrating cells and strikingly found that ETV7 was unique and had the strongest positive correlation with T cells CD4 memory activated (Figure 2(c)). After profiles of ETV7 in BLCA, the result shown higher ETV7 expression in tumors than normal samples (Figures 3(a)–3(c)). The correlation between ETV7 with clinicopathological parameters by UALCAN shown that the ETV7 was remarkably correlated with cancer stage. After preliminary study, this gene can be used as an independent prognostic factor. Indeed, all patients in various subgroups of BLCA with the high ETV7 expression showed a significantly shorter OS than those with the l ow ETV7 expression. Recent study reviewed that the ETV7 was a crucial prognostic factor in melanoma, which could potentially regulate the immune microenvironment [15], but its results from OS is opposite from our study. This may have to do with the specific function of genes in different tumor tissues.

ETV7 is a vital member of the ETS family, but its role in bladder cancer has been rarely reported. Our understanding of the role in pathology and physiology is rather limited. Previously, ETV7 was found to be highly associated with oncogene ETV6 [14]. Nevertheless, recent study shown the opposite function of two genes [17, 18]. ETV has earlier been reported act as an essential component of a rapamycin-insensitive mTOR complex in cancer [16].

To explore the role of ETV7 in BLCA, we divided the patients into low and high ETV7 groups and identified 87 DEGs (Figures 5(a) and 5(b), Supplementary Table S8). We analyzed 87 DGEs by conducting GO, KEGG, and GSEA analyses which are widely used bioinformatic tools in the functional location of specific genes. All of these enrichment analyses broad ETV7 involvement in immune related process and pathways, including the following: response to inteferon-gamma, cellular response to inteferon-gamma, inteferon-gamma-mediated signaling pathways, antigen processing and presentation, natural killer cell mediated cytotoxicity, JAK-STAT signaling pathway, MHC complex, and Th1, Th2, and Th17 cell differentiation. Th cell differentiation has been reported by nearly reports in melanoma which had a strong correlation with ETV7, and this is constant with our current study [15].The immune score of BLCA patients with high expression of ETV7 was significantly higher than that of BLCA patients with low expression of ETV7, suggesting a higher degree of immune cell infiltration in the tumor microenvironment. Indeed, when the infiltration of various immune cells was estimated, the ETV7 showed strong positive correlation with high fraction of T cell CD8, T cell memory activated, T cell follicular helper and macrophages M1, which is similar to the previous study in melanoma [15].

In the low ETV7 group, there was a higher fraction of B cells naive, B cells memory T cells CD4 naive, macrophages M0, and Mast cell activated. These results confirm a strong association between ETV7 and T cell infiltration. In fact, in addition to find that ETV7 is closely related to the immune-related cell, process, and pathways, the KEGG and GO enrichment also showed the strong correlation with virus infection (HIV, HPV), and COVID-19 was also significantly enriched (Supplementary Table S9-S10); simultaneously, we found that ETV7 is involved in many intefron-related signaling pathway (Figure 5(c)). Indeed, recent study have found that ETV7 is an interferon-induced, repressive transcription factor that negatively regulates antiviral interferon-stimulated genes essential for controlling influenza virus and SARS-CoV-2 infections [32]. This result is in good agreement with our enrichment. Moreover, CSDC2 was identified a strong correlation with EMT pathway which can be associated with TIM in BLCA (Figure 2(b)), but it has not been investigated in BLCA microenvironment, we will continue to focus.

## Data Availability

Expression data along with all available clinical information were retrieved for bladder cancer patients from The Cancer Genome Atlas (https://portal.gdc.cancer.gov/).Transcription factors were downloaded from AnimalTFDB database (http://bioinfo.life.hust.edu.cn/AnimalTFDB#!/). Other data was also obtained from public database described in Materials and Methods.

## Supplementary Materials

Supplementary 1 Table S1: 1665 human transcription factors from the AnimalTFDB. Table S2-S3: the upregulated and down-regulated DEGs of the TCGA-BLCA database. Table S4: 117 DE-TFs in bladder cancer. Table S5: 13 genes met the criteria for statistical significance via the *P* < 0.05 among 117 DE-TFs in bladder cancer. Table S6-S7: univariate and multivariate analysis of *ETV7.* Table S8: a total of 87 DEGs (82 upregulated and 5 downregulated) were identified in the high *ETV7* group. Table S9: enrichment of immune response regulation and various immune cells by GO-BP classification. Table S10: KEGG pathway analysis of DEGs. Table S11-12: detailed results of GSEA-GO analysis. Table S13: ESTIMATE algorithm-derived immune scores.

Supplementary 2 Figure S1: supplementary Figure 1: ETV7 expression in multiple database. (a) The differential expression of ETV7 between BLCA and normal tissues in GEO database. (b) ETV7 expression in MERAV database. (c) ETV7 expression levels in different cancer types in the TIMER database.
