# Supplementary material for: Comprehensive Analysis Identified ETV7 as a Potential Prognostic Biomarker in Bladder Cancer
Source: Biomed Res Int. 2021 Dec 9;2021:8530186. doi: 10.1155/2021/8530186 (PMC8678556; doi:10.1155/2021/8530186)
Supplement: Supplementary 1 — Table S1: 1665 human transcription factors from the AnimalTFDB. Table S2-S3: the upregulated and down-regulated DEGs of the TCGA-BLCA database. Table S4: 117 DE-TFs in bladder cancer. Table S5: 13 genes met the criteria for statistical significance via the P < 0.05 among 117 DE-TFs in bladder cancer. Table S6-S7: univariate and multivariate analysis of ETV7. Table S8: a total of 87 DEGs (82 upregulated and 5 downregulated) were identified in the high ETV7 group. Table S9: enrichment of immune response regulation and various immune cells by GO-BP classification. Table S10: KEGG pathway analysis of DEGs. Table S11-12: detailed results of GSEA-GO analysis. Table S13: ESTIMATE algorithm-derived immune scores. [file 8530186.f1.pdf]

# Supplementary Table S1-S13

# Supplementary Table S1

| Species | Symbol | Ensembl | Family | Protein | Entrez ID |
|---|---|---|---|---|---|
| Homo_sapiens | ZBTB8B | ENSG00000273274 | ZBTB | ENSP00000476499; | 728116 |
| Homo_sapiens | GSX2 | ENSG00000180613 | Homeobox | ENSP00000319118; | 170825 |
| Homo_sapiens | TBX2 | ENSG00000121068 | T-box | ENSP00000404781; | 6909 |
| Homo_sapiens | PAX8 | ENSG00000125618 | PAX | ENSP00000395498; | 7849 |
| Homo_sapiens | CREB3L1 | ENSG00000157613 | TF_bZIP | ENSP00000481956; | 90993 |
| Homo_sapiens | NKX6-1 | ENSG00000163623 | Homeobox | ENSP00000295886; | 4825 |
| Homo_sapiens | ZNF621 | ENSG00000172888 | zf-C2H2 | ENSP00000386051; | 285268 |
| Homo_sapiens | ZNF138 | ENSG00000197008 | zf-C2H2 | ENSP00000303533; | 7697 |
| Homo_sapiens | ZNF654 | ENSG00000175105 | zf-C2H2 | ENSP00000312141; | 55279 |
| Homo_sapiens | POU3F3 | ENSG00000198914 | Pou | ENSP00000355001; | 5455 |
| Homo_sapiens | ALX1 | ENSG00000180318 | Homeobox | ENSP00000315417; | 8092 |
| Homo_sapiens | EMX1 | ENSG00000135638 | Homeobox | ENSP00000258106; | 2016 |
| Homo_sapiens | ZNF718 | ENSG00000250312 | zf-C2H2 | ENSP00000476435; | 255403 |
| Homo_sapiens | VSX2 | ENSG00000119614 | Homeobox | ENSP00000261980; | 338917 |
| Homo_sapiens | POU1F1 | ENSG00000064835 | Pou | ENSP00000454072; | 5449 |
| Homo_sapiens | RARA | ENSG00000131759 | THR-like | ENSP00000377649; | 5914 |
| Homo_sapiens | TLX3 | ENSG00000164438 | Homeobox | ENSP00000296921; | 30012 |
| Homo_sapiens | ZIC2 | ENSG00000043355 | zf-C2H2 | ENSP00000365514; | 7546 |
| Homo_sapiens | ZNF226 | ENSG00000167380 | zf-C2H2 | ENSP00000465121; | 7769 |
| Homo_sapiens | ZNF808 | ENSG00000198482 | zf-C2H2 | ENSP00000420522; | 388558 |
| Homo_sapiens | AC067968.1 | ENSG00000267022 | zf-C2H2 | ENSP00000467018; | - |
| Homo_sapiens | NR3C1 | ENSG00000113580 | ESR-like | ENSP00000427672; | 2908 |
| Homo_sapiens | HIC2 | ENSG00000169635 | ZBTB | ENSP00000385319; | 23119 |
| Homo_sapiens | ZNF442 | ENSG00000198342 | zf-C2H2 | ENSP00000440162; | 79973 |
| Homo_sapiens | FOSL1 | ENSG00000175592 | TF_bZIP | ENSP00000310170; | 8061 |
| Homo_sapiens | MTF1 | ENSG00000188786 | zf-C2H2 | ENSP00000362127; | 4520 |
| Homo_sapiens | YBX3 | ENSG00000060138 | CSD | ENSP00000279550; | 8531 |
| Homo_sapiens | ZNF705A | ENSG00000196946 | zf-C2H2 | ENSP00000379816; | 440077 |
| Homo_sapiens | NFE2 | ENSG00000123405 | TF_bZIP | ENSP00000397185; | 4778 |
| Homo_sapiens | RAX2 | ENSG00000173976 | Homeobox | ENSP00000450687; | 84839 |
| Homo_sapiens | SP1 | ENSG00000185591 | zf-C2H2 | ENSP00000329357; | 6667 |
| Homo_sapiens | ARID3A | ENSG00000116017 | ARID | ENSP00000263620; | 1820 |
| Homo_sapiens | FEZF1 | ENSG00000128610 | zf-C2H2 | ENSP00000392727; | 389549 |
| Homo_sapiens | THAP4 | ENSG00000176946 | THAP | ENSP00000385006; | 51078 |
| Homo_sapiens | ZNF286A | ENSG00000187607 | zf-C2H2 | ENSP00000466154; | 57335 |
| Homo_sapiens | FOXQ1 | ENSG00000164379 | Fork_head | ENSP00000296839; | 94234 |
| Homo_sapiens | AIRE | ENSG00000160224 | SAND | ENSP00000291582; | 326 |
| Homo_sapiens | SNAI3 | ENSG00000185669 | zf-C2H2 | ENSP00000327968; | 333929 |
| Homo_sapiens | ZNF224 | ENSG00000267680 | zf-C2H2 | ENSP00000337368; | 7767 |
| Homo_sapiens | ATOH8 | ENSG00000168874 | bHLH | ENSP00000304676; | 84913 |
| Homo_sapiens | MECOM | ENSG00000085276 | zf-C2H2 | ENSP00000420072; | 2122 |
| Homo_sapiens | ZNF45 | ENSG00000124459 | zf-C2H2 | ENSP00000269973; | 7596 |
| Homo_sapiens | ZNF674 | ENSG00000251192 | zf-C2H2 | ENSP00000428248; | 641339 |
| Homo_sapiens | ZXDA | ENSG00000198205 | zf-C2H2 | ENSP00000351530; | 7789 |
| Homo_sapiens | ST18 | ENSG00000147488 | zf-C2HC | ENSP00000428521; | 9705 |
| Homo_sapiens | NKX6-2 | ENSG00000148826 | Homeobox | ENSP00000357581; | 84504 |
| Homo_sapiens | ZNF689 | ENSG00000156853 | zf-C2H2 | ENSP00000287461; | 115509 |
| Homo_sapiens | ZNF449 | ENSG00000173275 | zf-C2H2 | ENSP00000339585; | 203523 |
| Homo_sapiens | ZNF2 | ENSG00000275111 | zf-C2H2 | ENSP00000480057; | 7549 |
| Homo_sapiens | TFDP2 | ENSG00000114126 | E2F | ENSP00000418122; | 7029 |
| Homo_sapiens | ZBTB25 | ENSG00000089775 | ZBTB | ENSP00000378204; | 7597 |
| Homo_sapiens | PIAS4 | ENSG00000105229 | zf-MIZ | ENSP00000262971; | 51588 |
| Homo_sapiens | HES4 | ENSG00000188290 | bHLH | ENSP00000393198; | 57801 |
| Homo_sapiens | ZNF592 | ENSG00000166716 | zf-C2H2 | ENSP00000452877; | 9640 |
| Homo_sapiens | WT1 | ENSG00000184937 | zf-C2H2 | ENSP00000491511; | 7490 |
| Homo_sapiens | ZBTB49 | ENSG00000168826 | ZBTB | ENSP00000338807; | 166793 |
| Homo_sapiens | PAX5 | ENSG00000196092 | PAX | ENSP00000429197; | 5079 |

| | | | | | |
|---|---|---|---|---|---|
| Homo_sapiens | MAFG | ENSG00000197063 | TF_bZIP | ENSP00000376173; | 4097 |
| Homo_sapiens | ZNF837 | ENSG00000152475 | zf-C2H2 | ENSP00000405699; | 116412 |
| Homo_sapiens | FOXD4 | ENSG00000170122 | Fork_head | ENSP00000371940; | 2298 |
| Homo_sapiens | GATA3 | ENSG00000107485 | zf-GATA | ENSP00000341619; | 2625 |
| Homo_sapiens | HOMEZ | ENSG00000215271 | Homeobox | ENSP00000453979; | 57594 |
| Homo_sapiens | ZNF26 | ENSG00000198393 | zf-C2H2 | ENSP00000333725; | 7574 |
| Homo_sapiens | ZSCAN20 | ENSG00000121903 | zf-C2H2 | ENSP00000355053; | 7579 |
| Homo_sapiens | MEOX2 | ENSG00000106511 | Homeobox | ENSP00000262041; | 4223 |
| Homo_sapiens | DMRTA1 | ENSG00000176399 | DM | ENSP00000319651; | 63951 |
| Homo_sapiens | ZNF567 | ENSG00000189042 | zf-C2H2 | ENSP00000467712; | 163081 |
| Homo_sapiens | OVOL1 | ENSG00000172818 | zf-C2H2 | ENSP00000434220; | 5017 |
| Homo_sapiens | PRRX2 | ENSG00000167157 | Homeobox | ENSP00000361547; | 51450 |
| Homo_sapiens | MAFB | ENSG00000204103 | TF_bZIP | ENSP00000362410; | 9935 |
| Homo_sapiens | ZNF195 | ENSG00000005801 | zf-C2H2 | ENSP00000387998; | 7748 |
| Homo_sapiens | ZNF75D | ENSG00000186376 | zf-C2H2 | ENSP00000359800; | 7626 |
| Homo_sapiens | PIAS2 | ENSG00000078043 | zf-MIZ | ENSP00000317163; | 9063 |
| Homo_sapiens | FOXJ1 | ENSG00000129654 | Fork_head | ENSP00000323880; | 2302 |
| Homo_sapiens | GRHL1 | ENSG00000134317 | CP2 | ENSP00000418275; | 29841 |
| Homo_sapiens | KLF11 | ENSG00000172059 | zf-C2H2 | ENSP00000444690; | 8462 |
| Homo_sapiens | ATF2 | ENSG00000115966 | TF_bZIP | ENSP00000407911; | 1386 |
| Homo_sapiens | NFAT5 | ENSG00000102908 | RHD | ENSP00000455628; | 10725 |
| Homo_sapiens | MXD1 | ENSG00000059728 | bHLH | ENSP00000410672; | 4084 |
| Homo_sapiens | SMAD3 | ENSG00000166949 | MH1 | ENSP00000332973; | 4088 |
| Homo_sapiens | DMRT2 | ENSG00000173253 | DM | ENSP00000397494; | 10655 |
| Homo_sapiens | BARHL1 | ENSG00000125492 | Homeobox | ENSP00000263610; | 56751 |
| Homo_sapiens | IKZF4 | ENSG00000123411 | zf-C2H2 | ENSP00000450020; | 64375 |
| Homo_sapiens | ZNF607 | ENSG00000198182 | zf-C2H2 | ENSP00000347338; | 84775 |
| Homo_sapiens | E2F7 | ENSG00000165891 | E2F | ENSP00000449284; | 144455 |
| Homo_sapiens | ZNF595 | ENSG00000272602 | zf-C2H2 | ENSP00000477392; | 152687 |
| Homo_sapiens | ETV5 | ENSG00000244405 | ETS | ENSP00000413755; | 2119 |
| Homo_sapiens | CSRNP3 | ENSG00000178662 | CSRNP_N | ENSP00000344042; | 80034 |
| Homo_sapiens | CEBPD | ENSG00000221869 | TF_bZIP | ENSP00000386165; | 1052 |
| Homo_sapiens | ZNF669 | ENSG00000188295 | zf-C2H2 | ENSP00000342818; | 79862 |
| Homo_sapiens | TBX22 | ENSG00000122145 | T-box | ENSP00000487527; | 50945 |
| Homo_sapiens | ZNF304 | ENSG00000131845 | zf-C2H2 | ENSP00000401642; | 57343 |
| Homo_sapiens | FOXN4 | ENSG00000139445 | Fork_head | ENSP00000347354; | 121643 |
| Homo_sapiens | TBX6 | ENSG00000149922 | T-box | ENSP00000279386; | 6911 |
| Homo_sapiens | ZBED2 | ENSG00000177494 | zf-BED | ENSP00000321370; | 79413 |
| Homo_sapiens | HES5 | ENSG00000197921 | bHLH | ENSP00000367714; | 388585 |
| Homo_sapiens | ZNF283 | ENSG00000167637 | zf-C2H2 | ENSP00000468708; | 284349 |
| Homo_sapiens | PLAG1 | ENSG00000181690 | zf-C2H2 | ENSP00000404067; | 5324 |
| Homo_sapiens | ZNF512B | ENSG00000196700 | zf-C2H2 | ENSP00000358904; | 57473 |
| Homo_sapiens | ZNF486 | ENSG00000256229 | zf-C2H2 | ENSP00000335042; | 90649 |
| Homo_sapiens | ZMIZ2 | ENSG00000122515 | zf-MIZ | ENSP00000409648; | 83637 |
| Homo_sapiens | TERF2 | ENSG00000132604 | MYB | ENSP00000254942; | 7014 |
| Homo_sapiens | TFAM | ENSG00000108064 | HMG | ENSP00000363002; | 7019 |
| Homo_sapiens | LCORL | ENSG00000178177 | HTH | ENSP00000371661; | 254251 |
| Homo_sapiens | THAP5 | ENSG00000177683 | THAP | ENSP00000400500; | 168451 |
| Homo_sapiens | TCF15 | ENSG00000125878 | bHLH | ENSP00000246080; | 6939 |
| Homo_sapiens | ZNF75A | ENSG00000162086 | zf-C2H2 | ENSP00000482769; | 7627 |
| Homo_sapiens | HMGB2 | ENSG00000164104 | HMG | ENSP00000296503; | 3148 |
| Homo_sapiens | EGR3 | ENSG00000179388 | zf-C2H2 | ENSP00000318057; | 1960 |
| Homo_sapiens | ZFP69B | ENSG00000187801 | zf-C2H2 | ENSP00000354547; | 65243 |
| Homo_sapiens | USF1 | ENSG00000158773 | bHLH | ENSP00000356998; | 7391 |
| Homo_sapiens | TBR1 | ENSG00000136535 | T-box | ENSP00000387023; | 10716 |
| Homo_sapiens | SMAD2 | ENSG00000175387 | MH1 | ENSP00000467075; | 4087 |
| Homo_sapiens | ZNF165 | ENSG00000197279 | zf-C2H2 | ENSP00000366542; | 7718 |
| Homo_sapiens | HOXA11 | ENSG00000005073 | Homeobox | ENSP00000448962; | 3207 |

| | | | | | |
|---|---|---|---|---|---|
| Homo_sapiens | LMX1A | ENSG00000162761 | Homeobox | ENSP00000356868; | 4009 |
| Homo_sapiens | LHX6 | ENSG00000106852 | Homeobox | ENSP00000362860; | 26468 |
| Homo_sapiens | ZNF155 | ENSG00000204920 | zf-C2H2 | ENSP00000385163; | 7711 |
| Homo_sapiens | ZNF16 | ENSG00000170631 | zf-C2H2 | ENSP00000378369; | 7564 |
| Homo_sapiens | ZNF225 | ENSG00000256294 | zf-C2H2 | ENSP00000468686; | 7768 |
| Homo_sapiens | ZSCAN10 | ENSG00000130182 | zf-C2H2 | ENSP00000459520; | 84891 |
| Homo_sapiens | HNF1B | ENSG00000275410 | Homeobox | ENSP00000482529; | 6928 |
| Homo_sapiens | AC008758.1 | ENSG00000196826 | zf-C2H2 | ENSP00000404127; | - |
| Homo_sapiens | HOXA13 | ENSG00000106031 | Homeobox | ENSP00000222753; | 3209 |
| Homo_sapiens | MKX | ENSG00000150051 | Homeobox | ENSP00000452751; | 283078 |
| Homo_sapiens | TBX1 | ENSG00000184058 | T-box | ENSP00000331791; | 6899 |
| Homo_sapiens | ZNF70 | ENSG00000187792 | zf-C2H2 | ENSP00000339314; | 7621 |
| Homo_sapiens | ZNF282 | ENSG00000170265 | zf-C2H2 | ENSP00000477841; | 8427 |
| Homo_sapiens | PRDM13 | ENSG00000112238 | zf-C2H2 | ENSP00000358217; | 59336 |
| Homo_sapiens | ZNF786 | ENSG00000197362 | zf-C2H2 | ENSP00000313516; | 136051 |
| Homo_sapiens | ZNF547 | ENSG00000152433 | zf-C2H2 | ENSP00000282282; | 284306 |
| Homo_sapiens | EOMES | ENSG00000163508 | T-box | ENSP00000295743; | 8320 |
| Homo_sapiens | HOXB1 | ENSG00000120094 | Homeobox | ENSP00000355140; | 3211 |
| Homo_sapiens | ZKSCAN3 | ENSG00000189298 | zf-C2H2 | ENSP00000341883; | 80317 |
| Homo_sapiens | ZNF648 | ENSG00000179930 | zf-C2H2 | ENSP00000344129; | 127665 |
| Homo_sapiens | MECP2 | ENSG00000169057 | MBD | ENSP00000301948; | 4204 |
| Homo_sapiens | WIZ | ENSG00000011451 | zf-C2H2 | ENSP00000445824; | 58525 |
| Homo_sapiens | ZNF317 | ENSG00000130803 | zf-C2H2 | ENSP00000353554; | 57693 |
| Homo_sapiens | ZGLP1 | ENSG00000220201 | zf-GATA | ENSP00000385403; | 100125288 |
| Homo_sapiens | BACH2 | ENSG00000112182 | TF_bZIP | ENSP00000437473; | 60468 |
| Homo_sapiens | IRX4 | ENSG00000113430 | Homeobox | ENSP00000424235; | 50805 |
| Homo_sapiens | IRF4 | ENSG00000137265 | IRF | ENSP00000436094; | 3662 |
| Homo_sapiens | HOXA9 | ENSG00000078399 | Homeobox | ENSP00000343619; | 3205 |
| Homo_sapiens | SMAD7 | ENSG00000101665 | MH1 | ENSP00000467621; | 4092 |
| Homo_sapiens | ZNF613 | ENSG00000176024 | zf-C2H2 | ENSP00000490115; | 79898 |
| Homo_sapiens | SOX30 | ENSG00000039600 | HMG | ENSP00000427984; | 11063 |
| Homo_sapiens | TSC22D4 | ENSG00000166925 | TSC22 | ENSP00000377560; | 81628 |
| Homo_sapiens | MEOX1 | ENSG00000005102 | Homeobox | ENSP00000321684; | 4222 |
| Homo_sapiens | ZNF438 | ENSG00000183621 | zf-C2H2 | ENSP00000406934; | 220929 |
| Homo_sapiens | TCF4 | ENSG00000196628 | bHLH | ENSP00000457649; | 6925 |
| Homo_sapiens | ZBTB18 | ENSG00000179456 | ZBTB | ENSP00000481278; | 10472 |
| Homo_sapiens | RBPJL | ENSG00000124232 | CSL | ENSP00000361826; | 11317 |
| Homo_sapiens | LYL1 | ENSG00000104903 | bHLH | ENSP00000264824; | 4066 |
| Homo_sapiens | BHLHE22 | ENSG00000180828 | bHLH | ENSP00000318799; | 27319 |
| Homo_sapiens | AC010422.6 | ENSG00000269693 | zf-C2H2 | ENSP00000473043; | - |
| Homo_sapiens | IRF3 | ENSG00000126456 | IRF | ENSP00000469113; | 3661 |
| Homo_sapiens | ZNF260 | ENSG00000254004 | zf-C2H2 | ENSP00000467219; | 339324 |
| Homo_sapiens | PROX2 | ENSG00000119608 | HPD | ENSP00000451223; | 283571 |
| Homo_sapiens | TP63 | ENSG00000073282 | P53 | ENSP00000389485; | 8626 |
| Homo_sapiens | HOXA6 | ENSG00000106006 | Homeobox | ENSP00000222728; | 3203 |
| Homo_sapiens | DMRTB1 | ENSG00000143006 | DM | ENSP00000360500; | 63948 |
| Homo_sapiens | CCDC169-SOHLH2 | ENSG00000250709 | bHLH | ENSP00000421868; | 100526761 |
| Homo_sapiens | YY2 | ENSG00000230797 | zf-C2H2 | ENSP00000389381; | 404281 |
| Homo_sapiens | ZNF143 | ENSG00000166478 | zf-C2H2 | ENSP00000379849; | 7702 |
| Homo_sapiens | PBRM1 | ENSG00000163939 | HMG | ENSP00000386643; | 55193 |
| Homo_sapiens | ZNF841 | ENSG00000197608 | zf-C2H2 | ENSP00000415453; | 284371 |
| Homo_sapiens | ADNP | ENSG00000101126 | Homeobox | ENSP00000495540; | 23394 |
| Homo_sapiens | SPIB | ENSG00000269404 | ETS | ENSP00000391877; | 6689 |
| Homo_sapiens | BCL11B | ENSG00000127152 | zf-C2H2 | ENSP00000387419; | 64919 |
| Homo_sapiens | ZNF513 | ENSG00000163795 | zf-C2H2 | ENSP00000318373; | 130557 |
| Homo_sapiens | NEUROD6 | ENSG00000164600 | bHLH | ENSP00000297142; | 63974 |
| Homo_sapiens | SRF | ENSG00000112658 | SRF | ENSP00000265354; | 6722 |
| Homo_sapiens | TBXT | ENSG00000164458 | T-box | ENSP00000355836; | 6862 |

| | | | | | |
|---|---|---|---|---|---|
| Homo_sapiens | HOXD9 | ENSG00000128709 | Homeobox | ENSP00000249499; | 3235 |
| Homo_sapiens | ZBTB47 | ENSG00000114853 | ZBTB | ENSP00000420968; | 92999 |
| Homo_sapiens | ZNF22 | ENSG00000165512 | zf-C2H2 | ENSP00000298299; | 7570 |
| Homo_sapiens | NFXL1 | ENSG00000170448 | zf-NF-X1 | ENSP00000370949; | 152518 |
| Homo_sapiens | ZNF543 | ENSG00000178229 | zf-C2H2 | ENSP00000322545; | 125919 |
| Homo_sapiens | ZNF540 | ENSG00000171817 | zf-C2H2 | ENSP00000465503; | 163255 |
| Homo_sapiens | SOX14 | ENSG00000168875 | HMG | ENSP00000305343; | 8403 |
| Homo_sapiens | TFAP4 | ENSG00000090447 | bHLH | ENSP00000459390; | 7023 |
| Homo_sapiens | ASCL2 | ENSG00000183734 | bHLH | ENSP00000332293; | 430 |
| Homo_sapiens | HEY1 | ENSG00000164683 | bHLH | ENSP00000429705; | 23462 |
| Homo_sapiens | EBF3 | ENSG00000108001 | COE | ENSP00000347463; | 253738 |
| Homo_sapiens | XBP1 | ENSG00000100219 | TF_bZIP | ENSP00000216037; | 7494 |
| Homo_sapiens | ZNF562 | ENSG00000171466 | zf-C2H2 | ENSP00000464716; | 54811 |
| Homo_sapiens | PROP1 | ENSG00000175325 | Homeobox | ENSP00000311290; | 5626 |
| Homo_sapiens | CDX4 | ENSG00000131264 | Homeobox | ENSP00000362613; | 1046 |
| Homo_sapiens | SOX10 | ENSG00000100146 | HMG | ENSP00000354130; | 6663 |
| Homo_sapiens | BORCS8-MEF2B | ENSG00000064489 | SRF | ENSP00000390762; | 4207 |
| Homo_sapiens | ZNF211 | ENSG00000121417 | zf-C2H2 | ENSP00000240731; | 10520 |
| Homo_sapiens | ZNF266 | ENSG00000174652 | zf-C2H2 | ENSP00000466714; | 10781 |
| Homo_sapiens | ZNF580 | ENSG00000213015 | zf-C2H2 | ENSP00000446126; | 51157 |
| Homo_sapiens | OVOL3 | ENSG00000105261 | zf-C2H2 | ENSP00000487603; | 728361 |
| Homo_sapiens | ZNF391 | ENSG00000124613 | zf-C2H2 | ENSP00000419498; | 346157 |
| Homo_sapiens | ATOH1 | ENSG00000172238 | bHLH | ENSP00000302216; | 474 |
| Homo_sapiens | ATF5 | ENSG00000169136 | TF_bZIP | ENSP00000470633; | 22809 |
| Homo_sapiens | MYB | ENSG00000118513 | MYB | ENSP00000432948; | 4602 |
| Homo_sapiens | ALX3 | ENSG00000156150 | Homeobox | ENSP00000358807; | 257 |
| Homo_sapiens | SP2 | ENSG00000167182 | zf-C2H2 | ENSP00000365931; | 6668 |
| Homo_sapiens | THAP2 | ENSG00000173451 | THAP | ENSP00000310796; | 83591 |
| Homo_sapiens | GATA2 | ENSG00000179348 | zf-GATA | ENSP00000417074; | 2624 |
| Homo_sapiens | CDX2 | ENSG00000165556 | Homeobox | ENSP00000370408; | 1045 |
| Homo_sapiens | OLIG2 | ENSG00000205927 | bHLH | ENSP00000371794; | 10215 |
| Homo_sapiens | SIM1 | ENSG00000112246 | bHLH | ENSP00000358210; | 6492 |
| Homo_sapiens | ZNF445 | ENSG00000185219 | zf-C2H2 | ENSP00000413073; | 353274 |
| Homo_sapiens | YBX2 | ENSG00000006047 | CSD | ENSP00000007699; | 51087 |
| Homo_sapiens | ZBTB10 | ENSG00000205189 | ZBTB | ENSP00000368384; | 65986 |
| Homo_sapiens | ZIK1 | ENSG00000171649 | zf-C2H2 | ENSP00000472867; | 284307 |
| Homo_sapiens | ZNF611 | ENSG00000213020 | zf-C2H2 | ENSP00000443505; | 81856 |
| Homo_sapiens | DLX2 | ENSG00000115844 | Homeobox | ENSP00000234198; | 1746 |
| Homo_sapiens | RREB1 | ENSG00000124782 | zf-C2H2 | ENSP00000420519; | 6239 |
| Homo_sapiens | GATA1 | ENSG00000102145 | zf-GATA | ENSP00000365853; | 2623 |
| Homo_sapiens | BHLHA15 | ENSG00000180535 | bHLH | ENSP00000326391; | 168620 |
| Homo_sapiens | SOX21 | ENSG00000125285 | HMG | ENSP00000366144; | 11166 |
| Homo_sapiens | MAFA | ENSG00000182759 | TF_bZIP | ENSP00000328364; | 389692 |
| Homo_sapiens | PITX1 | ENSG00000069011 | Homeobox | ENSP00000451199; | 5307 |
| Homo_sapiens | ZNF286B | ENSG00000249459 | zf-C2H2 | ENSP00000461413; | 729288 |
| Homo_sapiens | TGIF2LX | ENSG00000153779 | Homeobox | ENSP00000453704; | 90316 |
| Homo_sapiens | SATB1 | ENSG00000182568 | CUT | ENSP00000399518; | 6304 |
| Homo_sapiens | SIM2 | ENSG00000159263 | bHLH | ENSP00000290399; | 6493 |
| Homo_sapiens | EN1 | ENSG00000163064 | Homeobox | ENSP00000295206; | 2019 |
| Homo_sapiens | ZNF845 | ENSG00000213799 | zf-C2H2 | ENSP00000388311; | 91664 |
| Homo_sapiens | DRGX | ENSG00000165606 | Homeobox | ENSP00000401653; | 644168 |
| Homo_sapiens | MAX | ENSG00000125952 | bHLH | ENSP00000452197; | 4149 |
| Homo_sapiens | ZNF696 | ENSG00000185730 | zf-C2H2 | ENSP00000427857; | 79943 |
| Homo_sapiens | ZNF521 | ENSG00000198795 | zf-C2H2 | ENSP00000463000; | 25925 |
| Homo_sapiens | ZFP62 | ENSG00000196670 | zf-C2H2 | ENSP00000423820; | 643836 |
| Homo_sapiens | GLI3 | ENSG00000106571 | zf-C2H2 | ENSP00000379258; | 2737 |
| Homo_sapiens | ZBTB8A | ENSG00000160062 | ZBTB | ENSP00000317561; | 653121 |
| Homo_sapiens | ZNF791 | ENSG00000173875 | zf-C2H2 | ENSP00000342974; | 163049 |

| | | | | | |
|---|---|---|---|---|---|
| Homo_sapiens | ZFP42 | ENSG00000179059 | zf-C2H2 | ENSP00000483363; | 132625 |
| Homo_sapiens | TGIF1 | ENSG00000177426 | Homeobox | ENSP00000339631; | 7050 |
| Homo_sapiens | ZNF557 | ENSG00000130544 | zf-C2H2 | ENSP00000404065; | 79230 |
| Homo_sapiens | MEIS3 | ENSG00000105419 | Homeobox | ENSP00000454073; | 56917 |
| Homo_sapiens | ZNF565 | ENSG00000196357 | zf-C2H2 | ENSP00000306869; | 147929 |
| Homo_sapiens | AC002310.5 | ENSG00000261459 | zf-C2H2 | ENSP00000454241; | - |
| Homo_sapiens | DLX1 | ENSG00000144355 | Homeobox | ENSP00000354478; | 1745 |
| Homo_sapiens | OLIG1 | ENSG00000184221 | bHLH | ENSP00000371785; | 116448 |
| Homo_sapiens | GCFC2 | ENSG00000005436 | GCFC | ENSP00000318690; | 6936 |
| Homo_sapiens | ZNF124 | ENSG00000196418 | zf-C2H2 | ENSP00000440365; | 7678 |
| Homo_sapiens | ZBTB5 | ENSG00000168795 | ZBTB | ENSP00000307604; | 9925 |
| Homo_sapiens | ZNF554 | ENSG00000172006 | zf-C2H2 | ENSP00000321132; | 115196 |
| Homo_sapiens | ZBTB38 | ENSG00000177311 | ZBTB | ENSP00000406955; | 253461 |
| Homo_sapiens | ZNF600 | ENSG00000189190 | zf-C2H2 | ENSP00000344791; | 162966 |
| Homo_sapiens | BSX | ENSG00000188909 | Homeobox | ENSP00000344285; | 390259 |
| Homo_sapiens | BHLHE41 | ENSG00000123095 | bHLH | ENSP00000242728; | 79365 |
| Homo_sapiens | ZSCAN23 | ENSG00000187987 | zf-C2H2 | ENSP00000289788; | 222696 |
| Homo_sapiens | HOXA1 | ENSG00000105991 | Homeobox | ENSP00000494260; | 3198 |
| Homo_sapiens | MLX | ENSG00000108788 | bHLH | ENSP00000464697; | 6945 |
| Homo_sapiens | TSC22D1 | ENSG00000102804 | TSC22 | ENSP00000481585; | 8848 |
| Homo_sapiens | EGR4 | ENSG00000135625 | zf-C2H2 | ENSP00000445626; | 1961 |
| Homo_sapiens | DUXA | ENSG00000258873 | Homeobox | ENSP00000452398; | 503835 |
| Homo_sapiens | ZNF493 | ENSG00000196268 | zf-C2H2 | ENSP00000376110; | 284443 |
| Homo_sapiens | CREBZF | ENSG00000137504 | TF_bZIP | ENSP00000434281; | 58487 |
| Homo_sapiens | PAX1 | ENSG00000125813 | PAX | ENSP00000481334; | 5075 |
| Homo_sapiens | THAP11 | ENSG00000168286 | THAP | ENSP00000304689; | 57215 |
| Homo_sapiens | ARID4A | ENSG00000032219 | ARID | ENSP00000344556; | 5926 |
| Homo_sapiens | ZSCAN22 | ENSG00000182318 | zf-C2H2 | ENSP00000332433; | 342945 |
| Homo_sapiens | ZNF729 | ENSG00000196350 | zf-C2H2 | ENSP00000469582; | 100287226 |
| Homo_sapiens | ZNF221 | ENSG00000159905 | zf-C2H2 | ENSP00000467367; | 7638 |
| Homo_sapiens | FOXN1 | ENSG00000109101 | Fork_head | ENSP00000464645; | 8456 |
| Homo_sapiens | MSGN1 | ENSG00000151379 | bHLH | ENSP00000281047; | 343930 |
| Homo_sapiens | OTX2 | ENSG00000165588 | TF_Otx | ENSP00000451272; | 5015 |
| Homo_sapiens | TCF7L1 | ENSG00000152284 | HMG | ENSP00000282111; | 83439 |
| Homo_sapiens | THAP6 | ENSG00000174796 | THAP | ENSP00000422402; | 152815 |
| Homo_sapiens | IRF2 | ENSG00000168310 | IRF | ENSP00000425037; | 3660 |
| Homo_sapiens | TFAP2D | ENSG00000008197 | AP-2 | ENSP00000008391; | 83741 |
| Homo_sapiens | ZNF239 | ENSG00000196793 | zf-C2H2 | ENSP00000363569; | 8187 |
| Homo_sapiens | EBF1 | ENSG00000164330 | COE | ENSP00000477809; | 1879 |
| Homo_sapiens | GBX1 | ENSG00000164900 | Homeobox | ENSP00000297537; | 2636 |
| Homo_sapiens | REST | ENSG00000084093 | zf-C2H2 | ENSP00000311816; | 5978 |
| Homo_sapiens | CSDE1 | ENSG00000009307 | CSD | ENSP00000261443; | 7812 |
| Homo_sapiens | ZNF641 | ENSG00000167528 | zf-C2H2 | ENSP00000437832; | 121274 |
| Homo_sapiens | ZNF90 | ENSG00000213988 | zf-C2H2 | ENSP00000410466; | 7643 |
| Homo_sapiens | FOXN3 | ENSG00000053254 | Fork_head | ENSP00000451135; | 1112 |
| Homo_sapiens | ZNF385A | ENSG00000161642 | zf-C2H2 | ENSP00000338927; | 25946 |
| Homo_sapiens | ZNF727 | ENSG00000214652 | zf-C2H2 | ENSP00000485448; | 442319 |
| Homo_sapiens | ZNF257 | ENSG00000197134 | zf-C2H2 | ENSP00000470209; | 113835 |
| Homo_sapiens | C11orf95 | ENSG00000188070 | zf-BED | ENSP00000482180; | 65998 |
| Homo_sapiens | ZNF98 | ENSG00000197360 | zf-C2H2 | ENSP00000350418; | 148198 |
| Homo_sapiens | PPARD | ENSG00000112033 | THR-like | ENSP00000353916; | 5467 |
| Homo_sapiens | ZNF223 | ENSG00000178386 | zf-C2H2 | ENSP00000467647; | 7766 |
| Homo_sapiens | ZNF561 | ENSG00000171469 | zf-C2H2 | ENSP00000393074; | 93134 |
| Homo_sapiens | CDX1 | ENSG00000113722 | Homeobox | ENSP00000477928; | 1044 |
| Homo_sapiens | ZBTB3 | ENSG00000185670 | ZBTB | ENSP00000378286; | 79842 |
| Homo_sapiens | TADA2B | ENSG00000173011 | MYB | ENSP00000308022; | 93624 |
| Homo_sapiens | LHX2 | ENSG00000106689 | Homeobox | ENSP00000394978; | 9355 |
| Homo_sapiens | AC005324.4 | ENSG00000255104 | zf-C2H2 | ENSP00000458062; | - |

| | | | | | |
|---|---|---|---|---|---|
| Homo_sapiens | MEIS2 | ENSG00000134138 | Homeobox | ENSP00000453793; | 4212 |
| Homo_sapiens | MLXIPL | ENSG00000009950 | bHLH | ENSP00000392636; | 51085 |
| Homo_sapiens | ZNF274 | ENSG00000171606 | zf-C2H2 | ENSP00000484810; | 10782 |
| Homo_sapiens | HOXB2 | ENSG00000173917 | Homeobox | ENSP00000331741; | 3212 |
| Homo_sapiens | ZNF816 | ENSG00000180257 | zf-C2H2 | ENSP00000350295; | 125893 |
| Homo_sapiens | GTF3A | ENSG00000122034 | zf-C2H2 | ENSP00000389655; | 2971 |
| Homo_sapiens | NKX2-4 | ENSG00000125816 | Homeobox | ENSP00000345147; | 644524 |
| Homo_sapiens | ZNF134 | ENSG00000213762 | zf-C2H2 | ENSP00000379464; | 7693 |
| Homo_sapiens | ETV2 | ENSG00000105672 | ETS | ENSP00000368312; | 2116 |
| Homo_sapiens | POU2F1 | ENSG00000143190 | Pou | ENSP00000414660; | 5451 |
| Homo_sapiens | SALL1 | ENSG00000103449 | zf-C2H2 | ENSP00000251020; | 6299 |
| Homo_sapiens | ZNF136 | ENSG00000196646 | zf-C2H2 | ENSP00000344162; | 7695 |
| Homo_sapiens | SP140L | ENSG00000185404 | SAND | ENSP00000397911; | 93349 |
| Homo_sapiens | ZNF780B | ENSG00000128000 | zf-C2H2 | ENSP00000221355; | 163131 |
| Homo_sapiens | IRF5 | ENSG00000128604 | IRF | ENSP00000419950; | 3663 |
| Homo_sapiens | ZNF443 | ENSG00000180855 | zf-C2H2 | ENSP00000301547; | 10224 |
| Homo_sapiens | ETV1 | ENSG00000006468 | ETS | ENSP00000385686; | 2115 |
| Homo_sapiens | HMG20B | ENSG00000064961 | HMG | ENSP00000328269; | 10362 |
| Homo_sapiens | PGR | ENSG00000082175 | ESR-like | ENSP00000481227; | 5241 |
| Homo_sapiens | MYBL2 | ENSG00000101057 | MYB | ENSP00000380072; | 4605 |
| Homo_sapiens | EGR2 | ENSG00000122877 | zf-C2H2 | ENSP00000242480; | 1959 |
| Homo_sapiens | ZNF397 | ENSG00000186812 | zf-C2H2 | ENSP00000331577; | 84307 |
| Homo_sapiens | NFATC2 | ENSG00000101096 | RHD | ENSP00000396471; | 4773 |
| Homo_sapiens | OTX1 | ENSG00000115507 | TF_Otx | ENSP00000355631; | 5013 |
| Homo_sapiens | CREM | ENSG00000095794 | TF_bZIP | ENSP00000417807; | 1390 |
| Homo_sapiens | FOXO3 | ENSG00000118689 | Fork_head | ENSP00000339527; | 2309 |
| Homo_sapiens | ZBTB6 | ENSG00000186130 | ZBTB | ENSP00000362763; | 10773 |
| Homo_sapiens | ZNF433 | ENSG00000197647 | zf-C2H2 | ENSP00000448099; | 163059 |
| Homo_sapiens | CREB1 | ENSG00000118260 | TF_bZIP | ENSP00000236995; | 1385 |
| Homo_sapiens | POU3F4 | ENSG00000196767 | Pou | ENSP00000495996; | 5456 |
| Homo_sapiens | ZNF672 | ENSG00000171161 | zf-C2H2 | ENSP00000426199; | 79894 |
| Homo_sapiens | ZNF329 | ENSG00000181894 | zf-C2H2 | ENSP00000470008; | 79673 |
| Homo_sapiens | SCRT1 | ENSG00000261678 | zf-C2H2 | ENSP00000455711; | 83482 |
| Homo_sapiens | ZNF140 | ENSG00000196387 | zf-C2H2 | ENSP00000445411; | 7699 |
| Homo_sapiens | ZBED6 | ENSG00000257315 | zf-BED | ENSP00000447879; | 100381270 |
| Homo_sapiens | ZNF765 | ENSG00000196417 | zf-C2H2 | ENSP00000379689; | 91661 |
| Homo_sapiens | CRX | ENSG00000105392 | TF_Otx | ENSP00000221996; | 1406 |
| Homo_sapiens | ZNF853 | ENSG00000236609 | zf-C2H2 | ENSP00000455585; | 54753 |
| Homo_sapiens | BAZ2B | ENSG00000123636 | MBD | ENSP00000339670; | 29994 |
| Homo_sapiens | ZNF182 | ENSG00000147118 | zf-C2H2 | ENSP00000366142; | 7569 |
| Homo_sapiens | ZNF467 | ENSG00000181444 | zf-C2H2 | ENSP00000304769; | 168544 |
| Homo_sapiens | ZNF253 | ENSG00000256771 | zf-C2H2 | ENSP00000467235; | 56242 |
| Homo_sapiens | PRDM10 | ENSG00000170325 | zf-C2H2 | ENSP00000432237; | 56980 |
| Homo_sapiens | ZNF333 | ENSG00000160961 | zf-C2H2 | ENSP00000292530; | 84449 |
| Homo_sapiens | PRDM1 | ENSG00000057657 | zf-C2H2 | ENSP00000358092; | 639 |
| Homo_sapiens | ZIM3 | ENSG00000141946 | zf-C2H2 | ENSP00000269834; | 114026 |
| Homo_sapiens | GATA4 | ENSG00000136574 | zf-GATA | ENSP00000435043; | 2626 |
| Homo_sapiens | GLIS2 | ENSG00000126603 | zf-C2H2 | ENSP00000395547; | 84662 |
| Homo_sapiens | ZNF180 | ENSG00000167384 | zf-C2H2 | ENSP00000221327; | 7733 |
| Homo_sapiens | ZNF366 | ENSG00000178175 | zf-C2H2 | ENSP00000313158; | 167465 |
| Homo_sapiens | SOX18 | ENSG00000203883 | HMG | ENSP00000341815; | 54345 |
| Homo_sapiens | ZFP1 | ENSG00000184517 | zf-C2H2 | ENSP00000377080; | 162239 |
| Homo_sapiens | ZNF517 | ENSG00000197363 | zf-C2H2 | ENSP00000432025; | 340385 |
| Homo_sapiens | HOXD10 | ENSG00000128710 | Homeobox | ENSP00000249501; | 3236 |
| Homo_sapiens | ZNF112 | ENSG00000062370 | zf-C2H2 | ENSP00000337081; | 7771 |
| Homo_sapiens | CCDC88A | ENSG00000115355 | TF_bZIP | ENSP00000480931; | 55704 |
| Homo_sapiens | ZNF628 | ENSG00000197483 | zf-C2H2 | ENSP00000469591; | 89887 |
| Homo_sapiens | ZNF829 | ENSG00000185869 | zf-C2H2 | ENSP00000429266; | 374899 |

| | | | | | |
|---|---|---|---|---|---|
| Homo_sapiens | TADA2A | ENSG00000276234 | MYB | ENSP00000484884; | 6871 |
| Homo_sapiens | ASCL3 | ENSG00000176009 | bHLH | ENSP00000435770; | 56676 |
| Homo_sapiens | ZNF688 | ENSG00000229809 | zf-C2H2 | ENSP00000223459; | 146542 |
| Homo_sapiens | ZNF784 | ENSG00000179922 | zf-C2H2 | ENSP00000320096; | 147808 |
| Homo_sapiens | MYOD1 | ENSG00000129152 | bHLH | ENSP00000250003; | 4654 |
| Homo_sapiens | SMAD5 | ENSG00000113658 | MH1 | ENSP00000422954; | 4090 |
| Homo_sapiens | GSX1 | ENSG00000169840 | Homeobox | ENSP00000304331; | 219409 |
| Homo_sapiens | DMBX1 | ENSG00000197587 | Homeobox | ENSP00000361024; | 127343 |
| Homo_sapiens | ZNF419 | ENSG00000105136 | zf-C2H2 | ENSP00000390916; | 79744 |
| Homo_sapiens | SP110 | ENSG00000135899 | SAND | ENSP00000375902; | 3431 |
| Homo_sapiens | SALL3 | ENSG00000256463 | zf-C2H2 | ENSP00000461451; | 27164 |
| Homo_sapiens | BBX | ENSG00000114439 | HMG | ENSP00000385530; | 56987 |
| Homo_sapiens | FEZF2 | ENSG00000153266 | zf-C2H2 | ENSP00000418804; | 55079 |
| Homo_sapiens | EBF2 | ENSG00000221818 | COE | ENSP00000386178; | 64641 |
| Homo_sapiens | HMGA2 | ENSG00000149948 | HMGA | ENSP00000346658; | 8091 |
| Homo_sapiens | DACH1 | ENSG00000276644 | DACH | ENSP00000482245; | 1602 |
| Homo_sapiens | SETDB1 | ENSG00000143379 | MBD | ENSP00000432348; | 9869 |
| Homo_sapiens | VAX2 | ENSG00000116035 | Homeobox | ENSP00000405114; | 25806 |
| Homo_sapiens | MEF2D | ENSG00000116604 | SRF | ENSP00000477413; | 4209 |
| Homo_sapiens | ZNF273 | ENSG00000198039 | zf-C2H2 | ENSP00000418719; | 10793 |
| Homo_sapiens | ZNF287 | ENSG00000141040 | zf-C2H2 | ENSP00000379168; | 57336 |
| Homo_sapiens | ZNF37A | ENSG00000075407 | zf-C2H2 | ENSP00000329141; | 7587 |
| Homo_sapiens | RAX | ENSG00000134438 | Homeobox | ENSP00000334813; | 30062 |
| Homo_sapiens | OSR2 | ENSG00000164920 | zf-C2H2 | ENSP00000429910; | 116039 |
| Homo_sapiens | ZNF763 | ENSG00000197054 | zf-C2H2 | ENSP00000402017; | 284390 |
| Homo_sapiens | TSHZ3 | ENSG00000121297 | zf-C2H2 | ENSP00000240587; | 57616 |
| Homo_sapiens | ZNF555 | ENSG00000186300 | zf-C2H2 | ENSP00000467893; | 148254 |
| Homo_sapiens | HOXC10 | ENSG00000180818 | Homeobox | ENSP00000307321; | 3226 |
| Homo_sapiens | TOX | ENSG00000198846 | HMG | ENSP00000354842; | 9760 |
| Homo_sapiens | KLF8 | ENSG00000102349 | zf-C2H2 | ENSP00000417303; | 11279 |
| Homo_sapiens | ZNF235 | ENSG00000159917 | zf-C2H2 | ENSP00000291182; | 9310 |
| Homo_sapiens | ZNF746 | ENSG00000181220 | zf-C2H2 | ENSP00000395007; | 155061 |
| Homo_sapiens | VAX1 | ENSG00000148704 | Homeobox | ENSP00000277905; | 11023 |
| Homo_sapiens | BARX2 | ENSG00000043039 | Homeobox | ENSP00000281437; | 8538 |
| Homo_sapiens | ZNF311 | ENSG00000197935 | zf-C2H2 | ENSP00000366384; | 282890 |
| Homo_sapiens | RBPJ | ENSG00000168214 | CSL | ENSP00000423703; | 3516 |
| Homo_sapiens | PDX1 | ENSG00000139515 | Homeobox | ENSP00000370421; | 3651 |
| Homo_sapiens | ZXDB | ENSG00000198455 | zf-C2H2 | ENSP00000364023; | 158586 |
| Homo_sapiens | ZNF528 | ENSG00000167555 | zf-C2H2 | ENSP00000353652; | 84436 |
| Homo_sapiens | BATF3 | ENSG00000123685 | TF_bZIP | ENSP00000243440; | 55509 |
| Homo_sapiens | HOXC12 | ENSG00000123407 | Homeobox | ENSP00000243103; | 3228 |
| Homo_sapiens | SMAD1 | ENSG00000170365 | MH1 | ENSP00000426568; | 4086 |
| Homo_sapiens | ZFP91 | ENSG00000186660 | zf-C2H2 | ENSP00000339030; | 80829 |
| Homo_sapiens | HMX2 | ENSG00000188816 | Homeobox | ENSP00000341108; | 3167 |
| Homo_sapiens | ZNF416 | ENSG00000083817 | zf-C2H2 | ENSP00000196489; | 55659 |
| Homo_sapiens | ZNF776 | ENSG00000152443 | zf-C2H2 | ENSP00000321812; | 284309 |
| Homo_sapiens | SPDEF | ENSG00000124664 | ETS | ENSP00000363149; | 25803 |
| Homo_sapiens | ARID3C | ENSG00000205143 | ARID | ENSP00000368189; | 138715 |
| Homo_sapiens | ZNF417 | ENSG00000173480 | zf-C2H2 | ENSP00000472272; | 147687 |
| Homo_sapiens | FOXO1 | ENSG00000150907 | Fork_head | ENSP00000368880; | 2308 |
| Homo_sapiens | ZNF709 | ENSG00000242852 | zf-C2H2 | ENSP00000380840; | 163051 |
| Homo_sapiens | FOXH1 | ENSG00000160973 | Fork_head | ENSP00000366534; | 8928 |
| Homo_sapiens | ZNF80 | ENSG00000174255 | zf-C2H2 | ENSP00000417192; | 7634 |
| Homo_sapiens | ZNF296 | ENSG00000170684 | zf-C2H2 | ENSP00000302770; | 162979 |
| Homo_sapiens | ZFX | ENSG00000005889 | zf-C2H2 | ENSP00000438233; | 7543 |
| Homo_sapiens | SP4 | ENSG00000105866 | zf-C2H2 | ENSP00000222584; | 6671 |
| Homo_sapiens | ID1 | ENSG00000125968 | bHLH | ENSP00000365280; | 3397 |
| Homo_sapiens | ZNF701 | ENSG00000167562 | zf-C2H2 | ENSP00000301093; | 55762 |

| | | | | | |
|---|---|---|---|---|---|
| Homo_sapiens | CEBPB | ENSG00000172216 | TF_bZIP | ENSP00000305422; | 1051 |
| Homo_sapiens | ZNF66 | ENSG00000160229 | zf-C2H2 | ENSP00000461425; | 7617 |
| Homo_sapiens | TULP2 | ENSG00000104804 | Tub | ENSP00000429131; | 7288 |
| Homo_sapiens | RFX1 | ENSG00000132005 | RFX | ENSP00000254325; | 5989 |
| Homo_sapiens | ZNF724 | ENSG00000196081 | zf-C2H2 | ENSP00000413411; | 440519 |
| Homo_sapiens | GRHL2 | ENSG00000083307 | CP2 | ENSP00000379260; | 79977 |
| Homo_sapiens | ZNF879 | ENSG00000234284 | zf-C2H2 | ENSP00000414887; | 345462 |
| Homo_sapiens | TEAD3 | ENSG00000007866 | TEA | ENSP00000345772; | 7005 |
| Homo_sapiens | ZNF541 | ENSG00000118156 | zf-C2H2 | ENSP00000313258; | 84215 |
| Homo_sapiens | ZNF610 | ENSG00000167554 | zf-C2H2 | ENSP00000324441; | 162963 |
| Homo_sapiens | ELK4 | ENSG00000158711 | ETS | ENSP00000289703; | 2005 |
| Homo_sapiens | TERF1 | ENSG00000147601 | MYB | ENSP00000276603; | 7013 |
| Homo_sapiens | RXRG | ENSG00000143171 | RXR-like | ENSP00000482458; | 6258 |
| Homo_sapiens | USF2 | ENSG00000105698 | bHLH | ENSP00000368429; | 7392 |
| Homo_sapiens | ZNF35 | ENSG00000169981 | zf-C2H2 | ENSP00000379368; | 7584 |
| Homo_sapiens | FOXO4 | ENSG00000184481 | Fork_head | ENSP00000363377; | 4303 |
| Homo_sapiens | RFX2 | ENSG00000087903 | RFX | ENSP00000306335; | 5990 |
| Homo_sapiens | CTCFL | ENSG00000124092 | zf-C2H2 | ENSP00000243914; | 140690 |
| Homo_sapiens | AFF1 | ENSG00000172493 | AF-4 | ENSP00000425755; | 4299 |
| Homo_sapiens | ZNF775 | ENSG00000196456 | zf-C2H2 | ENSP00000417483; | 285971 |
| Homo_sapiens | ELF2 | ENSG00000109381 | ETS | ENSP00000377782; | 1998 |
| Homo_sapiens | ZNF32 | ENSG00000169740 | zf-C2H2 | ENSP00000363556; | 7580 |
| Homo_sapiens | LHX5 | ENSG00000089116 | Homeobox | ENSP00000261731; | 64211 |
| Homo_sapiens | SOHLH1 | ENSG00000165643 | bHLH | ENSP00000298466; | 402381 |
| Homo_sapiens | NSD2 | ENSG00000109685 | HMG | ENSP00000372348; | 7468 |
| Homo_sapiens | HMGB4 | ENSG00000176256 | HMG | ENSP00000429214; | 127540 |
| Homo_sapiens | ZBTB44 | ENSG00000196323 | ZBTB | ENSP00000433457; | 29068 |
| Homo_sapiens | ZNF444 | ENSG00000167685 | zf-C2H2 | ENSP00000468069; | 55311 |
| Homo_sapiens | NKX2-5 | ENSG00000183072 | Homeobox | ENSP00000327758; | 1482 |
| Homo_sapiens | ARID5B | ENSG00000150347 | ARID | ENSP00000308862; | 84159 |
| Homo_sapiens | JDP2 | ENSG00000140044 | TF_bZIP | ENSP00000399587; | 122953 |
| Homo_sapiens | FOS | ENSG00000170345 | TF_bZIP | ENSP00000451786; | 2353 |
| Homo_sapiens | BARX1 | ENSG00000131668 | Homeobox | ENSP00000253968; | 56033 |
| Homo_sapiens | LIN28B | ENSG00000187772 | CSD | ENSP00000489735; | 389421 |
| Homo_sapiens | LHX3 | ENSG00000107187 | Homeobox | ENSP00000483080; | 8022 |
| Homo_sapiens | NRF1 | ENSG00000106459 | Nrf1 | ENSP00000376924; | 4899 |
| Homo_sapiens | HOXC13 | ENSG00000123364 | Homeobox | ENSP00000243056; | 3229 |
| Homo_sapiens | FOXS1 | ENSG00000179772 | Fork_head | ENSP00000365145; | 2307 |
| Homo_sapiens | DMTF1 | ENSG00000135164 | MYB | ENSP00000463638; | 9988 |
| Homo_sapiens | ISL1 | ENSG00000016082 | Homeobox | ENSP00000230658; | 3670 |
| Homo_sapiens | STAT3 | ENSG00000168610 | STAT | ENSP00000373923; | 6774 |
| Homo_sapiens | ETV6 | ENSG00000139083 | ETS | ENSP00000379658; | 2120 |
| Homo_sapiens | IRX3 | ENSG00000177508 | Homeobox | ENSP00000331608; | 79191 |
| Homo_sapiens | ZNF627 | ENSG00000198551 | zf-C2H2 | ENSP00000354414; | 199692 |
| Homo_sapiens | ZKSCAN5 | ENSG00000196652 | zf-C2H2 | ENSP00000377725; | 23660 |
| Homo_sapiens | NFATC4 | ENSG00000100968 | RHD | ENSP00000451284; | 4776 |
| Homo_sapiens | ZNF212 | ENSG00000170260 | zf-C2H2 | ENSP00000338572; | 7988 |
| Homo_sapiens | KLF5 | ENSG00000102554 | zf-C2H2 | ENSP00000366915; | 688 |
| Homo_sapiens | ZNF572 | ENSG00000180938 | zf-C2H2 | ENSP00000319305; | 137209 |
| Homo_sapiens | HES1 | ENSG00000114315 | bHLH | ENSP00000232424; | 3280 |
| Homo_sapiens | ZNF343 | ENSG00000088876 | zf-C2H2 | ENSP00000278772; | 79175 |
| Homo_sapiens | ZNF91 | ENSG00000167232 | zf-C2H2 | ENSP00000300619; | 7644 |
| Homo_sapiens | THAP7 | ENSG00000184436 | THAP | ENSP00000382084; | 80764 |
| Homo_sapiens | GTF2IRD2B | ENSG00000174428 | GTF2I | ENSP00000480524; | 389524 |
| Homo_sapiens | ONECUT1 | ENSG00000169856 | CUT | ENSP00000476168; | 3175 |
| Homo_sapiens | ZNF79 | ENSG00000196152 | zf-C2H2 | ENSP00000438418; | 7633 |
| Homo_sapiens | ZSCAN5C | ENSG00000204532 | zf-C2H2 | ENSP00000435234; | 649137 |
| Homo_sapiens | TRERF1 | ENSG00000124496 | zf-C2H2 | ENSP00000346285; | 55809 |

| | | | | | |
|---|---|---|---|---|---|
| Homo_sapiens | BATF | ENSG00000156127 | TF_bZIP | ENSP00000450486; | 10538 |
| Homo_sapiens | LHX9 | ENSG00000143355 | Homeobox | ENSP00000356360; | 56956 |
| Homo_sapiens | TOX4 | ENSG00000092203 | HMG | ENSP00000393080; | 9878 |
| Homo_sapiens | REL | ENSG00000162924 | RHD | ENSP00000496299; | 5966 |
| Homo_sapiens | HOXD11 | ENSG00000128713 | Homeobox | ENSP00000249504; | 3237 |
| Homo_sapiens | ZBTB34 | ENSG00000177125 | ZBTB | ENSP00000362551; | 403341 |
| Homo_sapiens | ATF6B | ENSG00000213676 | TF_bZIP | ENSP00000364349; | 1388 |
| Homo_sapiens | FOXD4L6 | ENSG00000273514 | Fork_head | ENSP00000484875; | 653404 |
| Homo_sapiens | ELK3 | ENSG00000111145 | ETS | ENSP00000447857; | 2004 |
| Homo_sapiens | ZNF559 | ENSG00000188321 | zf-C2H2 | ENSP00000468153; | 84527 |
| Homo_sapiens | GSC2 | ENSG00000063515 | Homeobox | ENSP00000086933; | 2928 |
| Homo_sapiens | ONECUT3 | ENSG00000205922 | CUT | ENSP00000371786; | 390874 |
| Homo_sapiens | ARNT | ENSG00000143437 | bHLH | ENSP00000346372; | 405 |
| Homo_sapiens | ETV4 | ENSG00000175832 | ETS | ENSP00000465718; | 2118 |
| Homo_sapiens | ZNF792 | ENSG00000180884 | zf-C2H2 | ENSP00000385099; | 126375 |
| Homo_sapiens | IRF1 | ENSG00000125347 | IRF | ENSP00000480887; | 3659 |
| Homo_sapiens | NFATC3 | ENSG00000072736 | RHD | ENSP00000456120; | 4775 |
| Homo_sapiens | PAX2 | ENSG00000075891 | PAX | ENSP00000396259; | 5076 |
| Homo_sapiens | ZNF222 | ENSG00000159885 | zf-C2H2 | ENSP00000187879; | 7673 |
| Homo_sapiens | KLF4 | ENSG00000136826 | zf-C2H2 | ENSP00000363804; | 9314 |
| Homo_sapiens | TSC22D3 | ENSG00000157514 | TSC22 | ENSP00000361466; | 1831 |
| Homo_sapiens | ZNF229 | ENSG00000278318 | zf-C2H2 | ENSP00000479884; | 7772 |
| Homo_sapiens | TFEC | ENSG00000105967 | bHLH | ENSP00000318676; | 22797 |
| Homo_sapiens | ZNF668 | ENSG00000167394 | zf-C2H2 | ENSP00000440149; | 79759 |
| Homo_sapiens | HOXD1 | ENSG00000128645 | Homeobox | ENSP00000328598; | 3231 |
| Homo_sapiens | ASCL1 | ENSG00000139352 | bHLH | ENSP00000266744; | 429 |
| Homo_sapiens | RCOR3 | ENSG00000117625 | MYB | ENSP00000355972; | 55758 |
| Homo_sapiens | ZNF790 | ENSG00000197863 | zf-C2H2 | ENSP00000480834; | 388536 |
| Homo_sapiens | ZNF605 | ENSG00000196458 | zf-C2H2 | ENSP00000353314; | 100289635 |
| Homo_sapiens | POU4F3 | ENSG00000091010 | Pou | ENSP00000230732; | 5459 |
| Homo_sapiens | ESRRA | ENSG00000173153 | ESR-like | ENSP00000441970; | 2101 |
| Homo_sapiens | GLIS3 | ENSG00000107249 | zf-C2H2 | ENSP00000371398; | 169792 |
| Homo_sapiens | ZNF519 | ENSG00000175322 | zf-C2H2 | ENSP00000464872; | 162655 |
| Homo_sapiens | ZNF354A | ENSG00000169131 | zf-C2H2 | ENSP00000337122; | 6940 |
| Homo_sapiens | ZNF214 | ENSG00000149050 | zf-C2H2 | ENSP00000445373; | 7761 |
| Homo_sapiens | AC019117.3 | ENSG00000283321 | bHLH | ENSP00000490530; | - |
| Homo_sapiens | ZFAT | ENSG00000066827 | zf-C2H2 | ENSP00000429930; | 57623 |
| Homo_sapiens | ZKSCAN4 | ENSG00000187626 | zf-C2H2 | ENSP00000366509; | 387032 |
| Homo_sapiens | AC008770.2 | ENSG00000267179 | zf-C2H2 | ENSP00000467286; | - |
| Homo_sapiens | FOXA1 | ENSG00000129514 | Fork_head | ENSP00000250448; | 3169 |
| Homo_sapiens | ZNF658 | ENSG00000274349 | zf-C2H2 | ENSP00000479295; | 26149 |
| Homo_sapiens | PITX3 | ENSG00000107859 | Homeobox | ENSP00000359019; | 5309 |
| Homo_sapiens | ZNF496 | ENSG00000162714 | zf-C2H2 | ENSP00000294753; | 84838 |
| Homo_sapiens | ZNF248 | ENSG00000198105 | zf-C2H2 | ENSP00000349882; | 57209 |
| Homo_sapiens | KLF10 | ENSG00000155090 | zf-C2H2 | ENSP00000379222; | 7071 |
| Homo_sapiens | BCL6 | ENSG00000113916 | ZBTB | ENSP00000413122; | 604 |
| Homo_sapiens | HKR1 | ENSG00000181666 | zf-C2H2 | ENSP00000438261; | 284459 |
| Homo_sapiens | TCF7L2 | ENSG00000148737 | HMG | ENSP00000435694; | 6934 |
| Homo_sapiens | MAEL | ENSG00000143194 | HMG | ENSP00000356846; | 84944 |
| Homo_sapiens | NR1H4 | ENSG00000012504 | THR-like | ENSP00000315442; | 9971 |
| Homo_sapiens | POU5F1B | ENSG00000212993 | Pou | ENSP00000495779; | 5462 |
| Homo_sapiens | ZNF316 | ENSG00000205903 | zf-C2H2 | ENSP00000477706; | 100131017 |
| Homo_sapiens | POU2F2 | ENSG00000028277 | Pou | ENSP00000452930; | 5452 |
| Homo_sapiens | ZNF432 | ENSG00000256087 | zf-C2H2 | ENSP00000221315; | 9668 |
| Homo_sapiens | HSF5 | ENSG00000176160 | HSF | ENSP00000313243; | 124535 |
| Homo_sapiens | ZNF382 | ENSG00000161298 | zf-C2H2 | ENSP00000389722; | 84911 |
| Homo_sapiens | ZNF507 | ENSG00000168813 | zf-C2H2 | ENSP00000441549; | 22847 |
| Homo_sapiens | MYCL | ENSG00000116990 | bHLH | ENSP00000380494; | 4610 |

| | | | | | |
|---|---|---|---|---|---|
| Homo_sapiens | SNAI1 | ENSG00000124216 | zf-C2H2 | ENSP00000244050; | 6615 |
| Homo_sapiens | NKX6-3 | ENSG00000165066 | Homeobox | ENSP00000428361; | 157848 |
| Homo_sapiens | SREBF2 | ENSG00000198911 | bHLH | ENSP00000395728; | 6721 |
| Homo_sapiens | ZNF347 | ENSG00000197937 | zf-C2H2 | ENSP00000471712; | 84671 |
| Homo_sapiens | ZNF281 | ENSG00000162702 | zf-C2H2 | ENSP00000294740; | 23528 |
| Homo_sapiens | BNC2 | ENSG00000173068 | zf-C2H2 | ENSP00000370047; | 54796 |
| Homo_sapiens | HOXA10 | ENSG00000253293 | Homeobox | ENSP00000379633; | 3206 |
| Homo_sapiens | SOX4 | ENSG00000124766 | HMG | ENSP00000244745; | 6659 |
| Homo_sapiens | SPI1 | ENSG00000066336 | ETS | ENSP00000227163; | 6688 |
| Homo_sapiens | ZFP90 | ENSG00000184939 | zf-C2H2 | ENSP00000460547; | 146198 |
| Homo_sapiens | ZNF23 | ENSG00000167377 | zf-C2H2 | ENSP00000377171; | 7571 |
| Homo_sapiens | SOX11 | ENSG00000176887 | HMG | ENSP00000322568; | 6664 |
| Homo_sapiens | MIS18BP1 | ENSG00000129534 | MYB | ENSP00000309790; | 55320 |
| Homo_sapiens | HLX | ENSG00000136630 | Homeobox | ENSP00000408248; | 3142 |
| Homo_sapiens | ZNF85 | ENSG00000105750 | zf-C2H2 | ENSP00000471588; | 7639 |
| Homo_sapiens | ZNF799 | ENSG00000196466 | zf-C2H2 | ENSP00000415278; | 90576 |
| Homo_sapiens | MEF2C | ENSG00000081189 | SRF | ENSP00000422390; | 4208 |
| Homo_sapiens | PRDM16 | ENSG00000142611 | zf-C2H2 | ENSP00000426975; | 63976 |
| Homo_sapiens | ZNF408 | ENSG00000175213 | zf-C2H2 | ENSP00000309606; | 79797 |
| Homo_sapiens | ZNF200 | ENSG00000010539 | zf-C2H2 | ENSP00000395723; | 7752 |
| Homo_sapiens | ZNF883 | ENSG00000285447 | zf-C2H2 | ENSP00000490059; | 169834 |
| Homo_sapiens | ZNF526 | ENSG00000167625 | zf-C2H2 | ENSP00000301215; | 116115 |
| Homo_sapiens | DBP | ENSG00000105516 | TF_bZIP | ENSP00000469426; | 1628 |
| Homo_sapiens | ZNF473 | ENSG00000142528 | zf-C2H2 | ENSP00000375697; | 25888 |
| Homo_sapiens | ZNF551 | ENSG00000204519 | zf-C2H2 | ENSP00000472674; | 90233 |
| Homo_sapiens | IKZF2 | ENSG00000030419 | zf-C2H2 | ENSP00000342876; | 22807 |
| Homo_sapiens | E4F1 | ENSG00000167967 | zf-C2H2 | ENSP00000301727; | 1877 |
| Homo_sapiens | OTP | ENSG00000171540 | Homeobox | ENSP00000302814; | 23440 |
| Homo_sapiens | ZNF800 | ENSG00000048405 | zf-C2H2 | ENSP00000376989; | 168850 |
| Homo_sapiens | FERD3L | ENSG00000146618 | bHLH | ENSP00000275461; | 222894 |
| Homo_sapiens | ZNF865 | ENSG00000261221 | zf-C2H2 | ENSP00000457715; | 100507290 |
| Homo_sapiens | ZNF707 | ENSG00000181135 | zf-C2H2 | ENSP00000436212; | 101928160 |
| Homo_sapiens | ZNF606 | ENSG00000166704 | zf-C2H2 | ENSP00000343617; | 80095 |
| Homo_sapiens | SOX8 | ENSG00000005513 | HMG | ENSP00000293894; | 30812 |
| Homo_sapiens | GCM1 | ENSG00000137270 | GCM | ENSP00000259803; | 8521 |
| Homo_sapiens | BCL6B | ENSG00000161940 | ZBTB | ENSP00000445010; | 255877 |
| Homo_sapiens | HEYL | ENSG00000163909 | bHLH | ENSP00000361943; | 26508 |
| Homo_sapiens | SALL4 | ENSG00000101115 | zf-C2H2 | ENSP00000379319; | 57167 |
| Homo_sapiens | ZNF18 | ENSG00000154957 | zf-C2H2 | ENSP00000462383; | 7566 |
| Homo_sapiens | ZSCAN5B | ENSG00000197213 | zf-C2H2 | ENSP00000466072; | 342933 |
| Homo_sapiens | MTA1 | ENSG00000182979 | zf-GATA | ENSP00000394106; | 9112 |
| Homo_sapiens | NR6A1 | ENSG00000148200 | GCNF-like | ENSP00000420587; | 2649 |
| Homo_sapiens | JUN | ENSG00000177606 | TF_bZIP | ENSP00000360266; | 3725 |
| Homo_sapiens | ZNF141 | ENSG00000131127 | zf-C2H2 | ENSP00000240499; | 7700 |
| Homo_sapiens | NFX1 | ENSG00000086102 | zf-NF-X1 | ENSP00000368856; | 4799 |
| Homo_sapiens | ARX | ENSG00000004848 | Homeobox | ENSP00000368332; | 170302 |
| Homo_sapiens | PEG3 | ENSG00000198300 | zf-C2H2 | ENSP00000326581; | 5178 |
| Homo_sapiens | RARB | ENSG00000077092 | THR-like | ENSP00000332296; | 5915 |
| Homo_sapiens | NCOR2 | ENSG00000196498 | MYB | ENSP00000405367; | 9612 |
| Homo_sapiens | UBTFL1 | ENSG00000255009 | HMG | ENSP00000485108; | 642623 |
| Homo_sapiens | ZNF331 | ENSG00000130844 | zf-C2H2 | ENSP00000393817; | 55422 |
| Homo_sapiens | ZNF778 | ENSG00000170100 | zf-C2H2 | ENSP00000305203; | 197320 |
| Homo_sapiens | NRL | ENSG00000129535 | TF_bZIP | ENSP00000380193; | 4901 |
| Homo_sapiens | MYOG | ENSG00000122180 | bHLH | ENSP00000241651; | 4656 |
| Homo_sapiens | NOTO | ENSG00000214513 | Homeobox | ENSP00000381486; | 344022 |
| Homo_sapiens | ZNF205 | ENSG00000122386 | zf-C2H2 | ENSP00000371627; | 7755 |
| Homo_sapiens | LEF1 | ENSG00000138795 | HMG | ENSP00000369284; | 51176 |
| Homo_sapiens | ZNF529 | ENSG00000186020 | zf-C2H2 | ENSP00000465578; | 57711 |

| | | | | | |
|---|---|---|---|---|---|
| Homo_sapiens | HOXA2 | ENSG00000105996 | Homeobox | ENSP00000222718; | 3199 |
| Homo_sapiens | ZNF148 | ENSG00000163848 | zf-C2H2 | ENSP00000420448; | 7707 |
| Homo_sapiens | SMARCC1 | ENSG00000173473 | MYB | ENSP00000254480; | 6599 |
| Homo_sapiens | ZNF322 | ENSG00000181315 | zf-C2H2 | ENSP00000478899; | 79692 |
| Homo_sapiens | TAL2 | ENSG00000186051 | bHLH | ENSP00000334547; | 6887 |
| Homo_sapiens | EMX2 | ENSG00000170370 | Homeobox | ENSP00000450962; | 2018 |
| Homo_sapiens | KLF2 | ENSG00000127528 | zf-C2H2 | ENSP00000248071; | 10365 |
| Homo_sapiens | ZBTB22 | ENSG00000236104 | ZBTB | ENSP00000407545; | 9278 |
| Homo_sapiens | ZNF550 | ENSG00000251369 | zf-C2H2 | ENSP00000422344; | 162972 |
| Homo_sapiens | ZFP3 | ENSG00000180787 | zf-C2H2 | ENSP00000320347; | 124961 |
| Homo_sapiens | ZNF597 | ENSG00000167981 | zf-C2H2 | ENSP00000301744; | 146434 |
| Homo_sapiens | PBX4 | ENSG00000105717 | Homeobox | ENSP00000251203; | 80714 |
| Homo_sapiens | ZNF652 | ENSG00000198740 | zf-C2H2 | ENSP00000354686; | 22834 |
| Homo_sapiens | CSDC2 | ENSG00000172346 | CSD | ENSP00000417127; | 27254 |
| Homo_sapiens | TPRX1 | ENSG00000178928 | Homeobox | ENSP00000438832; | 284355 |
| Homo_sapiens | ZFHX3 | ENSG00000140836 | Homeobox | ENSP00000493252; | 463 |
| Homo_sapiens | ZNF358 | ENSG00000198816 | zf-C2H2 | ENSP00000472777; | 140467 |
| Homo_sapiens | FOXD1 | ENSG00000251493 | Fork_head | ENSP00000481581; | 2297 |
| Homo_sapiens | STAT2 | ENSG00000170581 | STAT | ENSP00000315768; | 6773 |
| Homo_sapiens | ZNF431 | ENSG00000196705 | zf-C2H2 | ENSP00000308578; | 170959 |
| Homo_sapiens | SREBF1 | ENSG00000072310 | bHLH | ENSP00000411516; | 6720 |
| Homo_sapiens | ZNF69 | ENSG00000198429 | zf-C2H2 | ENSP00000402985; | 7620 |
| Homo_sapiens | FOXB2 | ENSG00000204612 | Fork_head | ENSP00000365898; | 442425 |
| Homo_sapiens | MIER1 | ENSG00000198160 | MYB | ENSP00000360053; | 57708 |
| Homo_sapiens | LRRFIP2 | ENSG00000093167 | LRRFIP | ENSP00000338727; | 9209 |
| Homo_sapiens | GATA6 | ENSG00000141448 | zf-GATA | ENSP00000269216; | 2627 |
| Homo_sapiens | HIVEP2 | ENSG00000010818 | zf-C2H2 | ENSP00000356576; | 3097 |
| Homo_sapiens | ZNF100 | ENSG00000197020 | zf-C2H2 | ENSP00000445201; | 163227 |
| Homo_sapiens | SOHLH2 | ENSG00000120669 | bHLH | ENSP00000369210; | 54937 |
| Homo_sapiens | ZKSCAN8 | ENSG00000198315 | zf-C2H2 | ENSP00000402948; | 7745 |
| Homo_sapiens | HOXC9 | ENSG00000180806 | Homeobox | ENSP00000423861; | 3225 |
| Homo_sapiens | SP9 | ENSG00000217236 | zf-C2H2 | ENSP00000378418; | 100131390 |
| Homo_sapiens | ZNF684 | ENSG00000117010 | zf-C2H2 | ENSP00000361784; | 127396 |
| Homo_sapiens | ZNF484 | ENSG00000127081 | zf-C2H2 | ENSP00000364646; | 83744 |
| Homo_sapiens | ZNF570 | ENSG00000171827 | zf-C2H2 | ENSP00000331540; | 148268 |
| Homo_sapiens | ZNF251 | ENSG00000198169 | zf-C2H2 | ENSP00000292562; | 90987 |
| Homo_sapiens | NR1H3 | ENSG00000025434 | THR-like | ENSP00000384745; | 10062 |
| Homo_sapiens | ZIC3 | ENSG00000156925 | zf-C2H2 | ENSP00000359638; | 7547 |
| Homo_sapiens | FEV | ENSG00000163497 | ETS | ENSP00000295727; | 54738 |
| Homo_sapiens | NFYB | ENSG00000120837 | NF-YB | ENSP00000448250; | 4801 |
| Homo_sapiens | ZNF677 | ENSG00000197928 | zf-C2H2 | ENSP00000334394; | 342926 |
| Homo_sapiens | HOPX | ENSG00000171476 | Homeobox | ENSP00000396275; | 84525 |
| Homo_sapiens | ZNF460 | ENSG00000197714 | zf-C2H2 | ENSP00000446167; | 10794 |
| Homo_sapiens | ARID3B | ENSG00000179361 | ARID | ENSP00000455668; | 10620 |
| Homo_sapiens | ZNF581 | ENSG00000171425 | zf-C2H2 | ENSP00000466564; | 51545 |
| Homo_sapiens | GLMP | ENSG00000198715 | NCU-G1 | ENSP00000483691; | 112770 |
| Homo_sapiens | ZNF649 | ENSG00000198093 | zf-C2H2 | ENSP00000347043; | 65251 |
| Homo_sapiens | NKX1-1 | ENSG00000235608 | Homeobox | ENSP00000407978; | 54729 |
| Homo_sapiens | ZNF469 | ENSG00000225614 | zf-C2H2 | ENSP00000402343; | 84627 |
| Homo_sapiens | VEZF1 | ENSG00000136451 | zf-C2H2 | ENSP00000462337; | 7716 |
| Homo_sapiens | ZNF518A | ENSG00000177853 | zf-C2H2 | ENSP00000480280; | 9849 |
| Homo_sapiens | TFDP1 | ENSG00000198176 | E2F | ENSP00000364519; | 7027 |
| Homo_sapiens | RELA | ENSG00000173039 | RHD | ENSP00000436545; | 5970 |
| Homo_sapiens | CREB3L3 | ENSG00000060566 | TF_bZIP | ENSP00000469355; | 84699 |
| Homo_sapiens | GZF1 | ENSG00000125812 | ZBTB | ENSP00000366250; | 64412 |
| Homo_sapiens | ZNF19 | ENSG00000157429 | zf-C2H2 | ENSP00000462810; | 7567 |
| Homo_sapiens | SAMD11 | ENSG00000187634 | SAND | ENSP00000411579; | 148398 |
| Homo_sapiens | RUNX2 | ENSG00000124813 | Runt | ENSP00000496517; | 860 |

| | | | | | |
|---|---|---|---|---|---|
| Homo_sapiens | HSF2 | ENSG00000025156 | HSF | ENSP00000357440; | 3298 |
| Homo_sapiens | CARHSP1 | ENSG00000153048 | CSD | ENSP00000478055; | 23589 |
| Homo_sapiens | TFCP2L1 | ENSG00000115112 | CP2 | ENSP00000263707; | 29842 |
| Homo_sapiens | TSHZ1 | ENSG00000179981 | zf-C2H2 | ENSP00000464391; | 10194 |
| Homo_sapiens | ZNF234 | ENSG00000263002 | zf-C2H2 | ENSP00000465011; | 10780 |
| Homo_sapiens | ZNF43 | ENSG00000198521 | zf-C2H2 | ENSP00000350085; | 7594 |
| Homo_sapiens | HIF3A | ENSG00000124440 | bHLH | ENSP00000432809; | 64344 |
| Homo_sapiens | FOXE3 | ENSG00000186790 | Fork_head | ENSP00000334472; | 2301 |
| Homo_sapiens | POU5F2 | ENSG00000248483 | Pou | ENSP00000489796; | 134187 |
| Homo_sapiens | NOBOX | ENSG00000106410 | Homeobox | ENSP00000496732; | 135935 |
| Homo_sapiens | FOXR2 | ENSG00000189299 | Fork_head | ENSP00000427329; | 139628 |
| Homo_sapiens | ID4 | ENSG00000172201 | bHLH | ENSP00000367972; | 3400 |
| Homo_sapiens | ZNF93 | ENSG00000184635 | zf-C2H2 | ENSP00000467553; | 81931 |
| Homo_sapiens | PTF1A | ENSG00000168267 | bHLH | ENSP00000365687; | 256297 |
| Homo_sapiens | ZNF625 | ENSG00000257591 | zf-C2H2 | ENSP00000394380; | 90589 |
| Homo_sapiens | PAX6 | ENSG00000007372 | PAX | ENSP00000492316; | 5080 |
| Homo_sapiens | ZNF267 | ENSG00000185947 | zf-C2H2 | ENSP00000300870; | 10308 |
| Homo_sapiens | PAX7 | ENSG00000009709 | PAX | ENSP00000383502; | 5081 |
| Homo_sapiens | BHLHE23 | ENSG00000125533 | bHLH | ENSP00000480998; | 128408 |
| Homo_sapiens | GRHL3 | ENSG00000158055 | CP2 | ENSP00000348333; | 57822 |
| Homo_sapiens | ZNF512 | ENSG00000243943 | zf-C2H2 | ENSP00000347648; | 84450 |
| Homo_sapiens | ZFP2 | ENSG00000198939 | zf-C2H2 | ENSP00000430531; | 80108 |
| Homo_sapiens | ZNF787 | ENSG00000142409 | zf-C2H2 | ENSP00000478557; | 126208 |
| Homo_sapiens | GMEB1 | ENSG00000162419 | SAND | ENSP00000355186; | 10691 |
| Homo_sapiens | ZNF700 | ENSG00000196757 | zf-C2H2 | ENSP00000479449; | 90592 |
| Homo_sapiens | SIX5 | ENSG00000177045 | Homeobox | ENSP00000494267; | 147912 |
| Homo_sapiens | HOXD8 | ENSG00000175879 | Homeobox | ENSP00000406045; | 3234 |
| Homo_sapiens | EBF4 | ENSG00000088881 | COE | ENSP00000345030; | 57593 |
| Homo_sapiens | ZNF692 | ENSG00000171163 | zf-C2H2 | ENSP00000305483; | 55657 |
| Homo_sapiens | MYT1 | ENSG00000196132 | zf-C2HC | ENSP00000353269; | 4661 |
| Homo_sapiens | CPHXL | ENSG00000283755 | Homeobox | ENSP00000491599; | 105371346 |
| Homo_sapiens | ZNF615 | ENSG00000197619 | zf-C2H2 | ENSP00000365906; | 284370 |
| Homo_sapiens | NR2F1 | ENSG00000175745 | RXR-like | ENSP00000481517; | 7025 |
| Homo_sapiens | IRF7 | ENSG00000185507 | IRF | ENSP00000433903; | 3665 |
| Homo_sapiens | ZNF117 | ENSG00000152926 | zf-C2H2 | ENSP00000479944; | 109504726 |
| Homo_sapiens | ZNF579 | ENSG00000218891 | zf-C2H2 | ENSP00000320188; | 163033 |
| Homo_sapiens | ZNF556 | ENSG00000172000 | zf-C2H2 | ENSP00000467366; | 80032 |
| Homo_sapiens | ZNF777 | ENSG00000196453 | zf-C2H2 | ENSP00000247930; | 27153 |
| Homo_sapiens | FOXE1 | ENSG00000178919 | Fork_head | ENSP00000364265; | 2304 |
| Homo_sapiens | ZNF680 | ENSG00000173041 | zf-C2H2 | ENSP00000309330; | 340252 |
| Homo_sapiens | ZIC4 | ENSG00000174963 | zf-C2H2 | ENSP00000417855; | 84107 |
| Homo_sapiens | NFYC | ENSG00000066136 | NF-YC | ENSP00000436710; | 4802 |
| Homo_sapiens | PRDM15 | ENSG00000141956 | zf-C2H2 | ENSP00000408592; | 63977 |
| Homo_sapiens | ZNF468 | ENSG00000204604 | zf-C2H2 | ENSP00000375660; | 162967 |
| Homo_sapiens | ID2 | ENSG00000115738 | bHLH | ENSP00000385465; | 3398 |
| Homo_sapiens | ZSCAN5A | ENSG00000131848 | zf-C2H2 | ENSP00000466445; | 79149 |
| Homo_sapiens | ISL2 | ENSG00000159556 | Homeobox | ENSP00000290759; | 64843 |
| Homo_sapiens | ZNF710 | ENSG00000140548 | zf-C2H2 | ENSP00000268154; | 374655 |
| Homo_sapiens | ZIC5 | ENSG00000139800 | zf-C2H2 | ENSP00000267294; | 85416 |
| Homo_sapiens | ZBED4 | ENSG00000100426 | zf-BED | ENSP00000216268; | 9889 |
| Homo_sapiens | ZNF679 | ENSG00000197123 | zf-C2H2 | ENSP00000416809; | 168417 |
| Homo_sapiens | HMBOX1 | ENSG00000147421 | Homeobox | ENSP00000380516; | 79618 |
| Homo_sapiens | RFX5 | ENSG00000143390 | RFX | ENSP00000376502; | 5993 |
| Homo_sapiens | ZNF687 | ENSG00000143373 | zf-C2H2 | ENSP00000398821; | 57592 |
| Homo_sapiens | ZNF773 | ENSG00000152439 | zf-C2H2 | ENSP00000282292; | 374928 |
| Homo_sapiens | HOXB7 | ENSG00000260027 | Homeobox | ENSP00000239165; | 3217 |
| Homo_sapiens | ZNF470 | ENSG00000197016 | zf-C2H2 | ENSP00000333223; | 388566 |
| Homo_sapiens | HHEX | ENSG00000152804 | Homeobox | ENSP00000450017; | 3087 |

| | | | | | |
|---|---|---|---|---|---|
| Homo_sapiens | ANHX | ENSG00000227059 | Homeobox | ENSP00000439513; | 647589 |
| Homo_sapiens | L3MBTL1 | ENSG00000185513 | zf-C2HC | ENSP00000402107; | 26013 |
| Homo_sapiens | ASCL4 | ENSG00000187855 | bHLH | ENSP00000345420; | 121549 |
| Homo_sapiens | ZNF480 | ENSG00000198464 | zf-C2H2 | ENSP00000334164; | 147657 |
| Homo_sapiens | FOXI3 | ENSG00000214336 | Fork_head | ENSP00000478384; | 344167 |
| Homo_sapiens | ZFP57 | ENSG00000204644 | zf-C2H2 | ENSP00000366078; | 346171 |
| Homo_sapiens | RFX8 | ENSG00000196460 | RFX | ENSP00000494216; | 731220 |
| Homo_sapiens | GLI4 | ENSG00000250571 | zf-C2H2 | ENSP00000430987; | 2738 |
| Homo_sapiens | MBD3 | ENSG00000071655 | MBD | ENSP00000412302; | 53615 |
| Homo_sapiens | ZNF732 | ENSG00000186777 | zf-C2H2 | ENSP00000478210; | 654254 |
| Homo_sapiens | TCF12 | ENSG00000140262 | bHLH | ENSP00000444696; | 6938 |
| Homo_sapiens | ELK1 | ENSG00000126767 | ETS | ENSP00000345585; | 2002 |
| Homo_sapiens | DBX1 | ENSG00000109851 | Homeobox | ENSP00000436881; | 120237 |
| Homo_sapiens | GFI1 | ENSG00000162676 | zf-C2H2 | ENSP00000399719; | 2672 |
| Homo_sapiens | ZNF726 | ENSG00000213967 | zf-C2H2 | ENSP00000471516; | 730087 |
| Homo_sapiens | SIX3 | ENSG00000138083 | Homeobox | ENSP00000260653; | 6496 |
| Homo_sapiens | ZSCAN12 | ENSG00000158691 | zf-C2H2 | ENSP00000380039; | 9753 |
| Homo_sapiens | BATF2 | ENSG00000168062 | TF_bZIP | ENSP00000301887; | 116071 |
| Homo_sapiens | NR5A2 | ENSG00000116833 | SF-like | ENSP00000236914; | 2494 |
| Homo_sapiens | ZEB2 | ENSG00000169554 | zf-C2H2 | ENSP00000454157; | 9839 |
| Homo_sapiens | GTF2I | ENSG00000263001 | GTF2I | ENSP00000477837; | 2969 |
| Homo_sapiens | ZNF568 | ENSG00000198453 | zf-C2H2 | ENSP00000466901; | 374900 |
| Homo_sapiens | ZNF217 | ENSG00000171940 | zf-C2H2 | ENSP00000360526; | 7764 |
| Homo_sapiens | CDC5L | ENSG00000096401 | MYB | ENSP00000360532; | 988 |
| Homo_sapiens | TSC22D2 | ENSG00000196428 | TSC22 | ENSP00000354543; | 9819 |
| Homo_sapiens | PIAS3 | ENSG00000131788 | zf-MIZ | ENSP00000358304; | 10401 |
| Homo_sapiens | IRX5 | ENSG00000176842 | Homeobox | ENSP00000378132; | 10265 |
| Homo_sapiens | ERF | ENSG00000105722 | ETS | ENSP00000388173; | 2077 |
| Homo_sapiens | ZFP37 | ENSG00000136866 | zf-C2H2 | ENSP00000452552; | 7539 |
| Homo_sapiens | ETV3 | ENSG00000117036 | ETS | ENSP00000357175; | 2117 |
| Homo_sapiens | TFE3 | ENSG00000068323 | bHLH | ENSP00000314129; | 7030 |
| Homo_sapiens | CTCF | ENSG00000102974 | zf-C2H2 | ENSP00000495218; | 10664 |
| Homo_sapiens | EGR1 | ENSG00000120738 | zf-C2H2 | ENSP00000239938; | 1958 |
| Homo_sapiens | TFDP3 | ENSG00000183434 | E2F | ENSP00000385461; | 51270 |
| Homo_sapiens | E2F2 | ENSG00000007968 | E2F | ENSP00000355249; | 1870 |
| Homo_sapiens | ZFP36L2 | ENSG00000152518 | zf-CCCH | ENSP00000282388; | 678 |
| Homo_sapiens | NFIC | ENSG00000141905 | CTF/NFI | ENSP00000342194; | 4782 |
| Homo_sapiens | LCOR | ENSG00000196233 | HTH | ENSP00000348298; | 84458 |
| Homo_sapiens | ZNF614 | ENSG00000142556 | zf-C2H2 | ENSP00000270649; | 80110 |
| Homo_sapiens | RFX7 | ENSG00000181827 | RFX | ENSP00000453376; | 64864 |
| Homo_sapiens | ZBTB7A | ENSG00000178951 | ZBTB | ENSP00000471865; | 51341 |
| Homo_sapiens | ZNF717 | ENSG00000227124 | zf-C2H2 | ENSP00000419377; | 100131827 |
| Homo_sapiens | ZNF34 | ENSG00000196378 | zf-C2H2 | ENSP00000434049; | 80778 |
| Homo_sapiens | NR4A2 | ENSG00000153234 | NGFIB-like | ENSP00000386747; | 4929 |
| Homo_sapiens | MEF2A | ENSG00000068305 | SRF | ENSP00000452989; | 4205 |
| Homo_sapiens | ZNF367 | ENSG00000165244 | zf-C2H2 | ENSP00000364405; | 195828 |
| Homo_sapiens | RCOR1 | ENSG00000089902 | MYB | ENSP00000262241; | 23186 |
| Homo_sapiens | ZNF264 | ENSG00000083844 | zf-C2H2 | ENSP00000263095; | 9422 |
| Homo_sapiens | ZNF441 | ENSG00000197044 | zf-C2H2 | ENSP00000350576; | 126068 |
| Homo_sapiens | TWIST1 | ENSG00000122691 | bHLH | ENSP00000242261; | 7291 |
| Homo_sapiens | AFF4 | ENSG00000072364 | AF-4 | ENSP00000265343; | 27125 |
| Homo_sapiens | STAT5A | ENSG00000126561 | STAT | ENSP00000465437; | 6776 |
| Homo_sapiens | ZNF888 | ENSG00000213793 | zf-C2H2 | ENSP00000491567; | 388559 |
| Homo_sapiens | AC023509.3 | ENSG00000267281 | TF_bZIP | ENSP00000466174; | - |
| Homo_sapiens | ZNF805 | ENSG00000204524 | zf-C2H2 | ENSP00000412999; | 390980 |
| Homo_sapiens | ZHX3 | ENSG00000174306 | Homeobox | ENSP00000443783; | 23051 |
| Homo_sapiens | ZNF584 | ENSG00000171574 | zf-C2H2 | ENSP00000471105; | 201514 |
| Homo_sapiens | PRRX1 | ENSG00000116132 | Homeobox | ENSP00000450762; | 5396 |

| | | | | | |
|---|---|---|---|---|---|
| Homo_sapiens | HOXA3 | ENSG00000105997 | Homeobox | ENSP00000379640; | 3200 |
| Homo_sapiens | E2F5 | ENSG00000133740 | E2F | ENSP00000398124; | 1875 |
| Homo_sapiens | KLF17 | ENSG00000171872 | zf-C2H2 | ENSP00000361373; | 128209 |
| Homo_sapiens | RHOXF1 | ENSG00000101883 | Homeobox | ENSP00000217999; | 158800 |
| Homo_sapiens | NKX2-3 | ENSG00000119919 | Homeobox | ENSP00000479692; | 159296 |
| Homo_sapiens | ZNF691 | ENSG00000164011 | zf-C2H2 | ENSP00000361585; | 51058 |
| Homo_sapiens | ZSCAN31 | ENSG00000235109 | zf-C2H2 | ENSP00000391235; | 64288 |
| Homo_sapiens | ZNF337 | ENSG00000130684 | zf-C2H2 | ENSP00000252979; | 26152 |
| Homo_sapiens | ZNF429 | ENSG00000197013 | zf-C2H2 | ENSP00000351280; | 353088 |
| Homo_sapiens | YY1 | ENSG00000100811 | zf-C2H2 | ENSP00000262238; | 7528 |
| Homo_sapiens | ZNF76 | ENSG00000065029 | zf-C2H2 | ENSP00000419106; | 7629 |
| Homo_sapiens | AC020909.1 | ENSG00000142539 | ETS | ENSP00000473233; | - |
| Homo_sapiens | ZSCAN2 | ENSG00000176371 | zf-C2H2 | ENSP00000351257; | 54993 |
| Homo_sapiens | ZNF12 | ENSG00000164631 | zf-C2H2 | ENSP00000384405; | 7559 |
| Homo_sapiens | DNAJC1 | ENSG00000136770 | MYB | ENSP00000366179; | 64215 |
| Homo_sapiens | ZNF585A | ENSG00000196967 | zf-C2H2 | ENSP00000375998; | 199704 |
| Homo_sapiens | ZNF385B | ENSG00000144331 | zf-C2H2 | ENSP00000386845; | 151126 |
| Homo_sapiens | FOXA2 | ENSG00000125798 | Fork_head | ENSP00000366319; | 3170 |
| Homo_sapiens | ZSCAN9 | ENSG00000137185 | zf-C2H2 | ENSP00000252207; | 7746 |
| Homo_sapiens | ZNF814 | ENSG00000204514 | zf-C2H2 | ENSP00000480123; | 730051 |
| Homo_sapiens | ZNF697 | ENSG00000143067 | zf-C2H2 | ENSP00000396857; | 90874 |
| Homo_sapiens | ZNF462 | ENSG00000148143 | zf-C2H2 | ENSP00000405837; | 58499 |
| Homo_sapiens | ZNF618 | ENSG00000157657 | zf-C2H2 | ENSP00000363241; | 114991 |
| Homo_sapiens | CREB3L2 | ENSG00000182158 | TF_bZIP | ENSP00000329140; | 64764 |
| Homo_sapiens | ZNF827 | ENSG00000151612 | zf-C2H2 | ENSP00000423130; | 152485 |
| Homo_sapiens | ZNF77 | ENSG00000175691 | zf-C2H2 | ENSP00000319053; | 58492 |
| Homo_sapiens | NEUROG2 | ENSG00000178403 | bHLH | ENSP00000317333; | 63973 |
| Homo_sapiens | MSC | ENSG00000178860 | bHLH | ENSP00000321445; | 9242 |
| Homo_sapiens | NEUROD1 | ENSG00000162992 | bHLH | ENSP00000295108; | 4760 |
| Homo_sapiens | ZBTB12 | ENSG00000204366 | ZBTB | ENSP00000364677; | 221527 |
| Homo_sapiens | ZNF629 | ENSG00000102870 | zf-C2H2 | ENSP00000262525; | 23361 |
| Homo_sapiens | ATOH7 | ENSG00000179774 | bHLH | ENSP00000362777; | 220202 |
| Homo_sapiens | KLF7 | ENSG00000118263 | zf-C2H2 | ENSP00000387510; | 8609 |
| Homo_sapiens | ZBTB48 | ENSG00000204859 | ZBTB | ENSP00000466390; | 3104 |
| Homo_sapiens | MESP2 | ENSG00000188095 | bHLH | ENSP00000342392; | 145873 |
| Homo_sapiens | FOXK1 | ENSG00000164916 | Fork_head | ENSP00000328720; | 221937 |
| Homo_sapiens | ZBTB41 | ENSG00000177888 | ZBTB | ENSP00000356375; | 360023 |
| Homo_sapiens | NR2C2 | ENSG00000177463 | RXR-like | ENSP00000388387; | 7182 |
| Homo_sapiens | ZNF558 | ENSG00000167785 | zf-C2H2 | ENSP00000301475; | 148156 |
| Homo_sapiens | RFX4 | ENSG00000111783 | RFX | ENSP00000444163; | 5992 |
| Homo_sapiens | SMARCC2 | ENSG00000139613 | MYB | ENSP00000449396; | 6601 |
| Homo_sapiens | PLAGL1 | ENSG00000118495 | zf-C2H2 | ENSP00000356544; | 5325 |
| Homo_sapiens | ZBTB7B | ENSG00000160685 | ZBTB | ENSP00000438647; | 51043 |
| Homo_sapiens | ZNF425 | ENSG00000204947 | zf-C2H2 | ENSP00000367300; | 155054 |
| Homo_sapiens | HOXB4 | ENSG00000182742 | Homeobox | ENSP00000328928; | 3214 |
| Homo_sapiens | ZNF500 | ENSG00000103199 | zf-C2H2 | ENSP00000464747; | 26048 |
| Homo_sapiens | FOXL2 | ENSG00000183770 | Fork_head | ENSP00000333188; | 668 |
| Homo_sapiens | PHOX2B | ENSG00000109132 | Homeobox | ENSP00000226382; | 8929 |
| Homo_sapiens | ISX | ENSG00000175329 | Homeobox | ENSP00000386037; | 91464 |
| Homo_sapiens | ZBTB21 | ENSG00000173276 | ZBTB | ENSP00000381512; | 49854 |
| Homo_sapiens | ZNF582 | ENSG00000018869 | zf-C2H2 | ENSP00000483414; | 147948 |
| Homo_sapiens | SP100 | ENSG00000067066 | SAND | ENSP00000343023; | 6672 |
| Homo_sapiens | ZSCAN21 | ENSG00000166529 | zf-C2H2 | ENSP00000292450; | 7589 |
| Homo_sapiens | STAT6 | ENSG00000166888 | STAT | ENSP00000438451; | 6778 |
| Homo_sapiens | FOXC2 | ENSG00000176692 | Fork_head | ENSP00000326371; | 2303 |
| Homo_sapiens | TCF3 | ENSG00000071564 | bHLH | ENSP00000467508; | 6929 |
| Homo_sapiens | LHX1 | ENSG00000273706 | Homeobox | ENSP00000477829; | 3975 |
| Homo_sapiens | ID3 | ENSG00000117318 | bHLH | ENSP00000363689; | 3399 |

| | | | | | |
|---|---|---|---|---|---|
| Homo_sapiens | ARID5A | ENSG00000196843 | ARID | ENSP00000350078; | 10865 |
| Homo_sapiens | PAX3 | ENSG00000135903 | PAX | ENSP00000386750; | 5077 |
| Homo_sapiens | DEAF1 | ENSG00000177030 | SAND | ENSP00000371846; | 10522 |
| Homo_sapiens | ZNF44 | ENSG00000197857 | zf-C2H2 | ENSP00000377008; | 51710 |
| Homo_sapiens | KLF1 | ENSG00000105610 | zf-C2H2 | ENSP00000264834; | 10661 |
| Homo_sapiens | GATAD2B | ENSG00000143614 | zf-GATA | ENSP00000489595; | 57459 |
| Homo_sapiens | SIX1 | ENSG00000126778 | Homeobox | ENSP00000494686; | 6495 |
| Homo_sapiens | ONECUT2 | ENSG00000119547 | CUT | ENSP00000419185; | 9480 |
| Homo_sapiens | ZFY | ENSG00000067646 | zf-C2H2 | ENSP00000372525; | 7544 |
| Homo_sapiens | ATMIN | ENSG00000166454 | zf-C2H2 | ENSP00000464427; | 23300 |
| Homo_sapiens | NFIA | ENSG00000162599 | CTF/NFI | ENSP00000360233; | 4774 |
| Homo_sapiens | FOXI2 | ENSG00000186766 | Fork_head | ENSP00000373572; | 399823 |
| Homo_sapiens | TFCP2 | ENSG00000135457 | CP2 | ENSP00000449280; | 7024 |
| Homo_sapiens | MBD4 | ENSG00000129071 | MBD | ENSP00000394080; | 8930 |
| Homo_sapiens | ZIC1 | ENSG00000152977 | zf-C2H2 | ENSP00000419664; | 7545 |
| Homo_sapiens | POU6F1 | ENSG00000184271 | Pou | ENSP00000330190; | 5463 |
| Homo_sapiens | FIGLA | ENSG00000183733 | bHLH | ENSP00000333097; | 344018 |
| Homo_sapiens | TFAP2E | ENSG00000116819 | AP-2 | ENSP00000362332; | 339488 |
| Homo_sapiens | ZNF213 | ENSG00000085644 | zf-C2H2 | ENSP00000380087; | 7760 |
| Homo_sapiens | ZNF17 | ENSG00000186272 | zf-C2H2 | ENSP00000302455; | 7565 |
| Homo_sapiens | HAND1 | ENSG00000113196 | bHLH | ENSP00000231121; | 9421 |
| Homo_sapiens | HSFX1 | ENSG00000171116 | HSF | ENSP00000359444; | 100506164 |
| Homo_sapiens | FOXC1 | ENSG00000054598 | Fork_head | ENSP00000493906; | 2296 |
| Homo_sapiens | PAX9 | ENSG00000198807 | PAX | ENSP00000355245; | 5083 |
| Homo_sapiens | ZNF28 | ENSG00000198538 | zf-C2H2 | ENSP00000375661; | 7576 |
| Homo_sapiens | MNT | ENSG00000070444 | bHLH | ENSP00000174618; | 4335 |
| Homo_sapiens | NEUROD2 | ENSG00000171532 | bHLH | ENSP00000306754; | 4761 |
| Homo_sapiens | ZNF133 | ENSG00000125846 | zf-C2H2 | ENSP00000346090; | 7692 |
| Homo_sapiens | BNC1 | ENSG00000169594 | zf-C2H2 | ENSP00000456727; | 646 |
| Homo_sapiens | PRDM9 | ENSG00000164256 | zf-C2H2 | ENSP00000296682; | 56979 |
| Homo_sapiens | HNF1A | ENSG00000135100 | Homeobox | ENSP00000443112; | 6927 |
| Homo_sapiens | ZNF653 | ENSG00000161914 | zf-C2H2 | ENSP00000293771; | 115950 |
| Homo_sapiens | UNCX | ENSG00000164853 | Homeobox | ENSP00000314480; | 340260 |
| Homo_sapiens | TGIF2 | ENSG00000118707 | Homeobox | ENSP00000362979; | 60436 |
| Homo_sapiens | ZSCAN32 | ENSG00000140987 | zf-C2H2 | ENSP00000483210; | 54925 |
| Homo_sapiens | ZFHX4 | ENSG00000091656 | Homeobox | ENSP00000430848; | 79776 |
| Homo_sapiens | ZFP69 | ENSG00000187815 | zf-C2H2 | ENSP00000361791; | 339559 |
| Homo_sapiens | ETV7 | ENSG00000010030 | ETS | ENSP00000484485; | 51513 |
| Homo_sapiens | TLX1 | ENSG00000107807 | Homeobox | ENSP00000359215; | 3195 |
| Homo_sapiens | ZNF747 | ENSG00000169955 | zf-C2H2 | ENSP00000457274; | 65988 |
| Homo_sapiens | ATF7 | ENSG00000170653 | TF_bZIP | ENSP00000387406; | 11016 |
| Homo_sapiens | ZNF74 | ENSG00000185252 | zf-C2H2 | ENSP00000385855; | 7625 |
| Homo_sapiens | ETV3L | ENSG00000253831 | ETS | ENSP00000430271; | 440695 |
| Homo_sapiens | FIZ1 | ENSG00000179943 | zf-C2H2 | ENSP00000468342; | 84922 |
| Homo_sapiens | ZNF177 | ENSG00000188629 | zf-C2H2 | ENSP00000468531; | 7730 |
| Homo_sapiens | ZKSCAN1 | ENSG00000106261 | zf-C2H2 | ENSP00000443508; | 7586 |
| Homo_sapiens | HOXA7 | ENSG00000122592 | Homeobox | ENSP00000242159; | 3204 |
| Homo_sapiens | GATA5 | ENSG00000130700 | zf-GATA | ENSP00000252997; | 140628 |
| Homo_sapiens | THAP3 | ENSG00000041988 | THAP | ENSP00000311537; | 90326 |
| Homo_sapiens | TFEB | ENSG00000112561 | bHLH | ENSP00000412551; | 7942 |
| Homo_sapiens | SOX3 | ENSG00000134595 | HMG | ENSP00000359567; | 6658 |
| Homo_sapiens | MYSM1 | ENSG00000162601 | MYB | ENSP00000418734; | 114803 |
| Homo_sapiens | HEY2 | ENSG00000135547 | bHLH | ENSP00000357349; | 23493 |
| Homo_sapiens | ZNF84 | ENSG00000198040 | zf-C2H2 | ENSP00000376133; | 7637 |
| Homo_sapiens | NCOA1 | ENSG00000084676 | bHLH | ENSP00000288599; | 8648 |
| Homo_sapiens | SUB1 | ENSG00000113387 | PC4 | ENSP00000265073; | 10923 |
| Homo_sapiens | ZNF723 | ENSG00000268696 | zf-C2H2 | ENSP00000494306; | 646864 |
| Homo_sapiens | SOX13 | ENSG00000143842 | HMG | ENSP00000356172; | 9580 |

| | | | | | |
|---|---|---|---|---|---|
| Homo_sapiens | ZBTB37 | ENSG00000185278 | ZBTB | ENSP00000415293; | 84614 |
| Homo_sapiens | MSX1 | ENSG00000163132 | Homeobox | ENSP00000372170; | 4487 |
| Homo_sapiens | ZNF564 | ENSG00000249709 | zf-C2H2 | ENSP00000340004; | 163050 |
| Homo_sapiens | NKX2-1 | ENSG00000136352 | Homeobox | ENSP00000346879; | 7080 |
| Homo_sapiens | ZNF292 | ENSG00000188994 | zf-C2H2 | ENSP00000342847; | 23036 |
| Homo_sapiens | ZNF749 | ENSG00000186230 | zf-C2H2 | ENSP00000333980; | 388567 |
| Homo_sapiens | LHX4 | ENSG00000121454 | Homeobox | ENSP00000263726; | 89884 |
| Homo_sapiens | HES2 | ENSG00000069812 | bHLH | ENSP00000367065; | 54626 |
| Homo_sapiens | HLF | ENSG00000108924 | TF_bZIP | ENSP00000226067; | 3131 |
| Homo_sapiens | IRF9 | ENSG00000213928 | IRF | ENSP00000453974; | 10379 |
| Homo_sapiens | PPARG | ENSG00000132170 | THR-like | ENSP00000495840; | 5468 |
| Homo_sapiens | SHOX2 | ENSG00000168779 | Homeobox | ENSP00000419362; | 6474 |
| Homo_sapiens | ZNF277 | ENSG00000198839 | zf-C2H2 | ENSP00000354501; | 11179 |
| Homo_sapiens | ZNF878 | ENSG00000257446 | zf-C2H2 | ENSP00000447931; | 729747 |
| Homo_sapiens | ARID1B | ENSG00000049618 | ARID | ENSP00000344546; | 57492 |
| Homo_sapiens | ZNF426 | ENSG00000130818 | zf-C2H2 | ENSP00000468100; | 79088 |
| Homo_sapiens | DLX4 | ENSG00000108813 | Homeobox | ENSP00000240306; | 1748 |
| Homo_sapiens | ZNF33B | ENSG00000196693 | zf-C2H2 | ENSP00000481265; | 7582 |
| Homo_sapiens | ZNF639 | ENSG00000121864 | zf-C2H2 | ENSP00000419650; | 51193 |
| Homo_sapiens | BARHL2 | ENSG00000143032 | Homeobox | ENSP00000359474; | 343472 |
| Homo_sapiens | ZNF83 | ENSG00000167766 | zf-C2H2 | ENSP00000472619; | 55769 |
| Homo_sapiens | HAND2 | ENSG00000164107 | bHLH | ENSP00000352565; | 9464 |
| Homo_sapiens | ZBTB42 | ENSG00000179627 | ZBTB | ENSP00000450673; | 100128927 |
| Homo_sapiens | ARNTL2 | ENSG00000029153 | bHLH | ENSP00000379238; | 56938 |
| Homo_sapiens | ZNF320 | ENSG00000182986 | zf-C2H2 | ENSP00000473091; | 162967 |
| Homo_sapiens | ZNF10 | ENSG00000256223 | zf-C2H2 | ENSP00000248211; | 7556 |
| Homo_sapiens | TUB | ENSG00000166402 | Tub | ENSP00000299506; | 7275 |
| Homo_sapiens | ZNF107 | ENSG00000196247 | zf-C2H2 | ENSP00000343443; | 51427 |
| Homo_sapiens | ZFP36L1 | ENSG00000185650 | zf-CCCH | ENSP00000388402; | 677 |
| Homo_sapiens | ZNF713 | ENSG00000178665 | zf-C2H2 | ENSP00000487818; | 349075 |
| Homo_sapiens | ARID2 | ENSG00000189079 | ARID | ENSP00000397307; | 196528 |
| Homo_sapiens | ATF4 | ENSG00000128272 | TF_bZIP | ENSP00000336790; | 468 |
| Homo_sapiens | KMT2C | ENSG00000055609 | HMG | ENSP00000347325; | 58508 |
| Homo_sapiens | ZNF783 | ENSG00000204946 | zf-C2H2 | ENSP00000410890; | 100289678 |
| Homo_sapiens | HIVEP1 | ENSG00000095951 | zf-C2H2 | ENSP00000486543; | 3096 |
| Homo_sapiens | MGA | ENSG00000174197 | T-box | ENSP00000219905; | 23269 |
| Homo_sapiens | ZNF560 | ENSG00000198028 | zf-C2H2 | ENSP00000301480; | 147741 |
| Homo_sapiens | MSX2 | ENSG00000120149 | Homeobox | ENSP00000239243; | 4488 |
| Homo_sapiens | ZNF174 | ENSG00000103343 | zf-C2H2 | ENSP00000268655; | 7727 |
| Homo_sapiens | TRPS1 | ENSG00000104447 | zf-GATA | ENSP00000428680; | 7227 |
| Homo_sapiens | ZNF616 | ENSG00000204611 | zf-C2H2 | ENSP00000471000; | 90317 |
| Homo_sapiens | DNAJC2 | ENSG00000105821 | MYB | ENSP00000368565; | 27000 |
| Homo_sapiens | ATF3 | ENSG00000162772 | TF_bZIP | ENSP00000355954; | 467 |
| Homo_sapiens | HOXB3 | ENSG00000120093 | Homeobox | ENSP00000418035; | 3213 |
| Homo_sapiens | ZNF101 | ENSG00000181896 | zf-C2H2 | ENSP00000468049; | 94039 |
| Homo_sapiens | ZNF583 | ENSG00000198440 | zf-C2H2 | ENSP00000291598; | 147949 |
| Homo_sapiens | ZNF880 | ENSG00000221923 | zf-C2H2 | ENSP00000406318; | 400713 |
| Homo_sapiens | ELF4 | ENSG00000102034 | ETS | ENSP00000478297; | 2000 |
| Homo_sapiens | ZHX2 | ENSG00000178764 | Homeobox | ENSP00000314709; | 22882 |
| Homo_sapiens | GSC | ENSG00000133937 | Homeobox | ENSP00000238558; | 145258 |
| Homo_sapiens | ZNF71 | ENSG00000197951 | zf-C2H2 | ENSP00000328245; | 58491 |
| Homo_sapiens | HBP1 | ENSG00000105856 | HMG | ENSP00000418738; | 26959 |
| Homo_sapiens | HOXB8 | ENSG00000120068 | Homeobox | ENSP00000460659; | 3218 |
| Homo_sapiens | ZNF302 | ENSG00000089335 | zf-C2H2 | ENSP00000396379; | 55900 |
| Homo_sapiens | ZFP41 | ENSG00000181638 | zf-C2H2 | ENSP00000327427; | 286128 |
| Homo_sapiens | TBX20 | ENSG00000164532 | T-box | ENSP00000386170; | 57057 |
| Homo_sapiens | NEUROG1 | ENSG00000181965 | bHLH | ENSP00000317580; | 4762 |
| Homo_sapiens | DUX4 | ENSG00000260596 | Homeobox | ENSP00000458065; | 100288687 |

| | | | | | |
|---|---|---|---|---|---|
| Homo_sapiens | PKNOX2 | ENSG00000165495 | Homeobox | ENSP00000298282; | 63876 |
| Homo_sapiens | SMARCA1 | ENSG00000102038 | MYB | ENSP00000360162; | 6594 |
| Homo_sapiens | ZSCAN18 | ENSG00000121413 | zf-C2H2 | ENSP00000412253; | 65982 |
| Homo_sapiens | ZNF334 | ENSG00000198185 | zf-C2H2 | ENSP00000485779; | 55713 |
| Homo_sapiens | ZNF219 | ENSG00000165804 | zf-C2H2 | ENSP00000450609; | 51222 |
| Homo_sapiens | THRA | ENSG00000126351 | THR-like | ENSP00000264637; | 7067 |
| Homo_sapiens | ZIM2 | ENSG00000269699 | zf-C2H2 | ENSP00000470326; | 23619 |
| Homo_sapiens | TCF24 | ENSG00000261787 | bHLH | ENSP00000455444; | 100129654 |
| Homo_sapiens | ZNF181 | ENSG00000197841 | zf-C2H2 | ENSP00000420727; | 339318 |
| Homo_sapiens | ALX4 | ENSG00000052850 | Homeobox | ENSP00000332744; | 60529 |
| Homo_sapiens | HDX | ENSG00000165259 | Homeobox | ENSP00000297977; | 139324 |
| Homo_sapiens | ZNF530 | ENSG00000183647 | zf-C2H2 | ENSP00000332861; | 348327 |
| Homo_sapiens | GMEB2 | ENSG00000101216 | SAND | ENSP00000359094; | 26205 |
| Homo_sapiens | SMAD9 | ENSG00000120693 | MH1 | ENSP00000382216; | 4093 |
| Homo_sapiens | HOXC11 | ENSG00000123388 | Homeobox | ENSP00000446680; | 3227 |
| Homo_sapiens | ZNF502 | ENSG00000196653 | zf-C2H2 | ENSP00000296091; | 91392 |
| Homo_sapiens | ZNF114 | ENSG00000178150 | zf-C2H2 | ENSP00000469998; | 163071 |
| Homo_sapiens | TBX4 | ENSG00000121075 | T-box | ENSP00000495714; | 9496 |
| Homo_sapiens | E2F6 | ENSG00000169016 | E2F | ENSP00000446315; | 1876 |
| Homo_sapiens | FOXD4L4 | ENSG00000184659 | Fork_head | ENSP00000366630; | 349334 |
| Homo_sapiens | ZNF233 | ENSG00000159915 | zf-C2H2 | ENSP00000375820; | 353355 |
| Homo_sapiens | ZNF230 | ENSG00000159882 | zf-C2H2 | ENSP00000409318; | 7773 |
| Homo_sapiens | ZNF594 | ENSG00000180626 | zf-C2H2 | ENSP00000382513; | 84622 |
| Homo_sapiens | TULP1 | ENSG00000112041 | Tub | ENSP00000477534; | 7287 |
| Homo_sapiens | HOXB9 | ENSG00000170689 | Homeobox | ENSP00000309439; | 3219 |
| Homo_sapiens | SSRP1 | ENSG00000149136 | HMG | ENSP00000489564; | 6749 |
| Homo_sapiens | DLX3 | ENSG00000064195 | Homeobox | ENSP00000449976; | 1747 |
| Homo_sapiens | ZBTB1 | ENSG00000126804 | ZBTB | ENSP00000451000; | 22890 |
| Homo_sapiens | AHR | ENSG00000106546 | bHLH | ENSP00000436466; | 196 |
| Homo_sapiens | ZNF660 | ENSG00000144792 | zf-C2H2 | ENSP00000324605; | 285349 |
| Homo_sapiens | CSRNP2 | ENSG00000110925 | CSRNP_N | ENSP00000228515; | 81566 |
| Homo_sapiens | HIVEP3 | ENSG00000127124 | zf-C2H2 | ENSP00000361665; | 59269 |
| Homo_sapiens | FOXJ3 | ENSG00000198815 | Fork_head | ENSP00000393408; | 22887 |
| Homo_sapiens | PRDM8 | ENSG00000152784 | zf-C2H2 | ENSP00000406998; | 56978 |
| Homo_sapiens | RORC | ENSG00000143365 | THR-like | ENSP00000327025; | 6097 |
| Homo_sapiens | CEBPG | ENSG00000153879 | TF_bZIP | ENSP00000284000; | 1054 |
| Homo_sapiens | BACH1 | ENSG00000156273 | TF_bZIP | ENSP00000416569; | 100379661 |
| Homo_sapiens | ZNF705B | ENSG00000215356 | zf-C2H2 | ENSP00000382987; | 100132396 |
| Homo_sapiens | ZNF319 | ENSG00000166188 | zf-C2H2 | ENSP00000299237; | 57567 |
| Homo_sapiens | FOXR1 | ENSG00000176302 | Fork_head | ENSP00000314806; | 283150 |
| Homo_sapiens | ZNF41 | ENSG00000147124 | zf-C2H2 | ENSP00000315173; | 7592 |
| Homo_sapiens | YBX1 | ENSG00000065978 | CSD | ENSP00000361626; | 4904 |
| Homo_sapiens | SATB2 | ENSG00000119042 | CUT | ENSP00000388764; | 23314 |
| Homo_sapiens | ZSCAN1 | ENSG00000152467 | zf-C2H2 | ENSP00000282326; | 284312 |
| Homo_sapiens | ZNF571 | ENSG00000180479 | zf-C2H2 | ENSP00000351594; | 51276 |
| Homo_sapiens | NKX2-8 | ENSG00000136327 | Homeobox | ENSP00000258829; | 26257 |
| Homo_sapiens | ZNF3 | ENSG00000166526 | zf-C2H2 | ENSP00000299667; | 7551 |
| Homo_sapiens | TEAD2 | ENSG00000074219 | TEA | ENSP00000366419; | 8463 |
| Homo_sapiens | NR5A1 | ENSG00000136931 | SF-like | ENSP00000483309; | 2516 |
| Homo_sapiens | ZNF485 | ENSG00000198298 | zf-C2H2 | ENSP00000363558; | 220992 |
| Homo_sapiens | NR2F2 | ENSG00000185551 | RXR-like | ENSP00000377721; | 7026 |
| Homo_sapiens | ZNF491 | ENSG00000177599 | zf-C2H2 | ENSP00000313443; | 126069 |
| Homo_sapiens | SP6 | ENSG00000189120 | zf-C2H2 | ENSP00000340799; | 80320 |
| Homo_sapiens | ZNF439 | ENSG00000171291 | zf-C2H2 | ENSP00000395632; | 90594 |
| Homo_sapiens | KLF14 | ENSG00000266265 | zf-C2H2 | ENSP00000463287; | 136259 |
| Homo_sapiens | IKZF1 | ENSG00000185811 | zf-C2H2 | ENSP00000413025; | 10320 |
| Homo_sapiens | UBP1 | ENSG00000153560 | CP2 | ENSP00000395558; | 7342 |
| Homo_sapiens | DUXB | ENSG00000282757 | Homeobox | ENSP00000491301; | 100033411 |

| | | | | | |
|---|---|---|---|---|---|
| Homo_sapiens | FOXA3 | ENSG00000170608 | Fork_head | ENSP00000304004; | 3171 |
| Homo_sapiens | ZBTB26 | ENSG00000171448 | ZBTB | ENSP00000362760; | 57684 |
| Homo_sapiens | ZNF215 | ENSG00000149054 | zf-C2H2 | ENSP00000393202; | 7762 |
| Homo_sapiens | ZNF284 | ENSG00000186026 | zf-C2H2 | ENSP00000411032; | 342909 |
| Homo_sapiens | ZNF667 | ENSG00000198046 | zf-C2H2 | ENSP00000439402; | 63934 |
| Homo_sapiens | ZKSCAN2 | ENSG00000155592 | zf-C2H2 | ENSP00000331626; | 342357 |
| Homo_sapiens | THAP9 | ENSG00000168152 | THAP | ENSP00000305533; | 79725 |
| Homo_sapiens | TCF21 | ENSG00000118526 | bHLH | ENSP00000237316; | 6943 |
| Homo_sapiens | ZNF197 | ENSG00000186448 | zf-C2H2 | ENSP00000345809; | 10168 |
| Homo_sapiens | ZNF549 | ENSG00000121406 | zf-C2H2 | ENSP00000365407; | 256051 |
| Homo_sapiens | PROX1 | ENSG00000117707 | HPD | ENSP00000420283; | 5629 |
| Homo_sapiens | TCFL5 | ENSG00000101190 | bHLH | ENSP00000217162; | 10732 |
| Homo_sapiens | FOSL2 | ENSG00000075426 | TF_bZIP | ENSP00000264716; | 2355 |
| Homo_sapiens | E2F3 | ENSG00000112242 | E2F | ENSP00000443418; | 1871 |
| Homo_sapiens | BAZ2A | ENSG00000076108 | MBD | ENSP00000368754; | 11176 |
| Homo_sapiens | HMGB3 | ENSG00000029993 | HMG | ENSP00000410354; | 3149 |
| Homo_sapiens | ZNF599 | ENSG00000153896 | zf-C2H2 | ENSP00000333802; | 148103 |
| Homo_sapiens | ZNF492 | ENSG00000229676 | zf-C2H2 | ENSP00000413660; | 57615 |
| Homo_sapiens | ZNF232 | ENSG00000167840 | zf-C2H2 | ENSP00000250076; | 7775 |
| Homo_sapiens | MIER3 | ENSG00000155545 | MYB | ENSP00000386584; | 166968 |
| Homo_sapiens | GLI1 | ENSG00000111087 | zf-C2H2 | ENSP00000436671; | 2735 |
| Homo_sapiens | ZBTB24 | ENSG00000112365 | ZBTB | ENSP00000230122; | 9841 |
| Homo_sapiens | ZNF532 | ENSG00000074657 | zf-C2H2 | ENSP00000468532; | 55205 |
| Homo_sapiens | ZNF644 | ENSG00000122482 | zf-C2H2 | ENSP00000482748; | 84146 |
| Homo_sapiens | HOXD3 | ENSG00000128652 | Homeobox | ENSP00000386498; | 3232 |
| Homo_sapiens | UBTF | ENSG00000108312 | HMG | ENSP00000345297; | 7343 |
| Homo_sapiens | ZNF410 | ENSG00000119725 | zf-C2H2 | ENSP00000334170; | 57862 |
| Homo_sapiens | SP140 | ENSG00000079263 | SAND | ENSP00000393618; | 11262 |
| Homo_sapiens | ZNF92 | ENSG00000146757 | zf-C2H2 | ENSP00000400495; | 168374 |
| Homo_sapiens | HINFP | ENSG00000172273 | zf-C2H2 | ENSP00000318085; | 25988 |
| Homo_sapiens | LHX8 | ENSG00000162624 | Homeobox | ENSP00000294638; | 431707 |
| Homo_sapiens | AC025287.4 | ENSG00000284484 | Homeobox | ENSP00000491197; | - |
| Homo_sapiens | ZNF20 | ENSG00000132010 | zf-C2H2 | ENSP00000335437; | 7568 |
| Homo_sapiens | ZNF418 | ENSG00000196724 | zf-C2H2 | ENSP00000379451; | 147686 |
| Homo_sapiens | ZBTB14 | ENSG00000198081 | ZBTB | ENSP00000349503; | 7541 |
| Homo_sapiens | MLXIP | ENSG00000175727 | bHLH | ENSP00000445891; | 22877 |
| Homo_sapiens | CREB3L4 | ENSG00000143578 | TF_bZIP | ENSP00000357592; | 148327 |
| Homo_sapiens | SCX | ENSG00000260428 | bHLH | ENSP00000476384; | 642658 |
| Homo_sapiens | NANOGP8 | ENSG00000255192 | Homeobox | ENSP00000487045; | 388112 |
| Homo_sapiens | ZNF548 | ENSG00000188785 | zf-C2H2 | ENSP00000379482; | 147694 |
| Homo_sapiens | L3MBTL4 | ENSG00000154655 | zf-C2HC | ENSP00000318543; | 91133 |
| Homo_sapiens | ZNF655 | ENSG00000197343 | zf-C2H2 | ENSP00000252713; | 79027 |
| Homo_sapiens | AC118549 | ENSG00000036549 | MYB | ENSP00000359837; | 26009 |
| Homo_sapiens | TBX10 | ENSG00000167800 | T-box | ENSP00000335191; | 347853 |
| Homo_sapiens | ZNF587 | ENSG00000198466 | zf-C2H2 | ENSP00000345479; | 84914 |
| Homo_sapiens | AC012531.3 | ENSG00000273049 | Homeobox | ENSP00000476742; | - |
| Homo_sapiens | ELF1 | ENSG00000120690 | ETS | ENSP00000489586; | 1997 |
| Homo_sapiens | AC073612.1 | ENSG00000258064 | THAP | ENSP00000454911; | - |
| Homo_sapiens | LIN28A | ENSG00000131914 | CSD | ENSP00000254231; | 79727 |
| Homo_sapiens | HMGXB4 | ENSG00000100281 | HMG | ENSP00000216106; | 10042 |
| Homo_sapiens | ZNF396 | ENSG00000186496 | zf-C2H2 | ENSP00000466500; | 252884 |
| Homo_sapiens | NR2E1 | ENSG00000112333 | RXR-like | ENSP00000357982; | 7101 |
| Homo_sapiens | ZNF823 | ENSG00000197933 | zf-C2H2 | ENSP00000340683; | 55552 |
| Homo_sapiens | STAT4 | ENSG00000138378 | STAT | ENSP00000376134; | 6775 |
| Homo_sapiens | ZNF626 | ENSG00000188171 | zf-C2H2 | ENSP00000484232; | 199777 |
| Homo_sapiens | AC105001.2 | ENSG00000258724 | HMG | ENSP00000451145; | - |
| Homo_sapiens | TEF | ENSG00000167074 | TF_bZIP | ENSP00000266304; | 7008 |
| Homo_sapiens | PPARA | ENSG00000186951 | THR-like | ENSP00000385523; | 5465 |

| | | | | | |
|---|---|---|---|---|---|
| Homo_sapiens | ZNF8 | ENSG00000278129 | zf-C2H2 | ENSP00000477716; | 7554 |
| Homo_sapiens | ZNF501 | ENSG00000186446 | zf-C2H2 | ENSP00000379363; | 115560 |
| Homo_sapiens | ZNF454 | ENSG00000178187 | zf-C2H2 | ENSP00000326249; | 285676 |
| Homo_sapiens | PRDM2 | ENSG00000116731 | zf-C2H2 | ENSP00000312352; | 7799 |
| Homo_sapiens | ETS1 | ENSG00000134954 | ETS | ENSP00000441430; | 2113 |
| Homo_sapiens | PRDM6 | ENSG00000061455 | zf-C2H2 | ENSP00000384725; | 93166 |
| Homo_sapiens | ZBED3 | ENSG00000132846 | zf-BED | ENSP00000427487; | 84327 |
| Homo_sapiens | ZNF354C | ENSG00000177932 | zf-C2H2 | ENSP00000324064; | 30832 |
| Homo_sapiens | ESX1 | ENSG00000123576 | Homeobox | ENSP00000361669; | 80712 |
| Homo_sapiens | HOXB6 | ENSG00000108511 | Homeobox | ENSP00000420009; | 3216 |
| Homo_sapiens | NFKB1 | ENSG00000109320 | RHD | ENSP00000226574; | 4790 |
| Homo_sapiens | TULP3 | ENSG00000078246 | Tub | ENSP00000380321; | 7289 |
| Homo_sapiens | RARG | ENSG00000172819 | THR-like | ENSP00000343698; | 5916 |
| Homo_sapiens | ZNF705G | ENSG00000215372 | zf-C2H2 | ENSP00000383020; | 100131980 |
| Homo_sapiens | OLIG3 | ENSG00000177468 | bHLH | ENSP00000356708; | 167826 |
| Homo_sapiens | ZNF821 | ENSG00000102984 | zf-C2H2 | ENSP00000458112; | 55565 |
| Homo_sapiens | HNF4A | ENSG00000101076 | RXR-like | ENSP00000315180; | 3172 |
| Homo_sapiens | TCF7 | ENSG00000081059 | HMG | ENSP00000378472; | 6932 |
| Homo_sapiens | SMAD4 | ENSG00000141646 | MH1 | ENSP00000381452; | 4089 |
| Homo_sapiens | DMRTC2 | ENSG00000142025 | DM | ENSP00000470553; | 63946 |
| Homo_sapiens | ZBED5 | ENSG00000236287 | zf-BED | ENSP00000398106; | 58486 |
| Homo_sapiens | NEUROG3 | ENSG00000122859 | bHLH | ENSP00000242462; | 50674 |
| Homo_sapiens | ESR2 | ENSG00000140009 | ESR-like | ENSP00000450488; | 2100 |
| Homo_sapiens | ZNF683 | ENSG00000176083 | zf-C2H2 | ENSP00000388792; | 257101 |
| Homo_sapiens | ZEB1 | ENSG00000148516 | zf-C2H2 | ENSP00000452787; | 6935 |
| Homo_sapiens | NPAS3 | ENSG00000151322 | bHLH | ENSP00000319610; | 64067 |
| Homo_sapiens | ZNF785 | ENSG00000197162 | zf-C2H2 | ENSP00000420340; | 146540 |
| Homo_sapiens | HOXA4 | ENSG00000197576 | Homeobox | ENSP00000448015; | 3201 |
| Homo_sapiens | ZNF831 | ENSG00000124203 | zf-C2H2 | ENSP00000360069; | 128611 |
| Homo_sapiens | HOXB5 | ENSG00000120075 | Homeobox | ENSP00000239151; | 3215 |
| Homo_sapiens | HMGXB3 | ENSG00000113716 | HMG | ENSP00000422231; | 22993 |
| Homo_sapiens | ZNF227 | ENSG00000131115 | zf-C2H2 | ENSP00000375823; | 7770 |
| Homo_sapiens | ZNF446 | ENSG00000083838 | zf-C2H2 | ENSP00000472219; | 55663 |
| Homo_sapiens | PAXBP1 | ENSG00000159086 | GCFC | ENSP00000290178; | 94104 |
| Homo_sapiens | HSFX2 | ENSG00000268738 | HSF | ENSP00000469223; | 100130086 |
| Homo_sapiens | ARID4B | ENSG00000054267 | ARID | ENSP00000391497; | 51742 |
| Homo_sapiens | ZNF157 | ENSG00000147117 | zf-C2H2 | ENSP00000366273; | 7712 |
| Homo_sapiens | MXD4 | ENSG00000123933 | bHLH | ENSP00000337889; | 10608 |
| Homo_sapiens | LRRFIP1 | ENSG00000124831 | LRRFIP | ENSP00000375857; | 9208 |
| Homo_sapiens | ZFP30 | ENSG00000120784 | zf-C2H2 | ENSP00000343581; | 22835 |
| Homo_sapiens | ELF3 | ENSG00000163435 | ETS | ENSP00000356253; | 1999 |
| Homo_sapiens | INSM2 | ENSG00000168348 | zf-C2H2 | ENSP00000306523; | 84684 |
| Homo_sapiens | HMGA1 | ENSG00000137309 | HMGA | ENSP00000385693; | 3159 |
| Homo_sapiens | MITF | ENSG00000187098 | bHLH | ENSP00000418845; | 4286 |
| Homo_sapiens | MXI1 | ENSG00000119950 | bHLH | ENSP00000358625; | 4601 |
| Homo_sapiens | NR4A1 | ENSG00000123358 | NGFIB-like | ENSP00000353427; | 3164 |
| Homo_sapiens | ZNF585B | ENSG00000245680 | zf-C2H2 | ENSP00000436774; | 92285 |
| Homo_sapiens | TBX21 | ENSG00000073861 | T-box | ENSP00000177694; | 30009 |
| Homo_sapiens | TFAP2C | ENSG00000087510 | AP-2 | ENSP00000201031; | 7022 |
| Homo_sapiens | HMG20A | ENSG00000140382 | HMG | ENSP00000452864; | 10363 |
| Homo_sapiens | ZNF770 | ENSG00000198146 | zf-C2H2 | ENSP00000453474; | 54989 |
| Homo_sapiens | KLF6 | ENSG00000067082 | zf-C2H2 | ENSP00000419923; | 1316 |
| Homo_sapiens | GLI2 | ENSG00000074047 | zf-C2H2 | ENSP00000354586; | 2736 |
| Homo_sapiens | SIX4 | ENSG00000100625 | Homeobox | ENSP00000450761; | 51804 |
| Homo_sapiens | ZNF813 | ENSG00000198346 | zf-C2H2 | ENSP00000379684; | 126017 |
| Homo_sapiens | ZNF7 | ENSG00000147789 | zf-C2H2 | ENSP00000432724; | 7553 |
| Homo_sapiens | FOXK2 | ENSG00000141568 | Fork_head | ENSP00000335677; | 3607 |
| Homo_sapiens | ZNF577 | ENSG00000161551 | zf-C2H2 | ENSP00000491349; | 84765 |

| | | | | | |
|---|---|---|---|---|---|
| Homo_sapiens | RHOXF2B | ENSG00000203989 | Homeobox | ENSP00000360455; | 727940 |
| Homo_sapiens | ZNF623 | ENSG00000183309 | zf-C2H2 | ENSP00000435232; | 9831 |
| Homo_sapiens | USF3 | ENSG00000176542 | bHLH | ENSP00000420721; | 205717 |
| Homo_sapiens | SOX12 | ENSG00000177732 | HMG | ENSP00000347646; | 6666 |
| Homo_sapiens | HSF4 | ENSG00000102878 | HSF | ENSP00000427832; | 3299 |
| Homo_sapiens | ZNF705E | ENSG00000214534 | zf-C2H2 | ENSP00000492790; | 100131539 |
| Homo_sapiens | GCM2 | ENSG00000124827 | GCM | ENSP00000368805; | 9247 |
| Homo_sapiens | E2F4 | ENSG00000205250 | E2F | ENSP00000458082; | 1874 |
| Homo_sapiens | FOXP1 | ENSG00000114861 | Fork_head | ENSP00000482847; | 27086 |
| Homo_sapiens | FOXF1 | ENSG00000103241 | Fork_head | ENSP00000262426; | 2294 |
| Homo_sapiens | DMRT1 | ENSG00000137090 | DM | ENSP00000371711; | 1761 |
| Homo_sapiens | ZNF518B | ENSG00000178163 | zf-C2H2 | ENSP00000317614; | 85460 |
| Homo_sapiens | ZNF436 | ENSG00000125945 | zf-C2H2 | ENSP00000313582; | 80818 |
| Homo_sapiens | NHLH1 | ENSG00000171786 | bHLH | ENSP00000302189; | 4807 |
| Homo_sapiens | ZBTB33 | ENSG00000177485 | ZBTB | ENSP00000450969; | 10009 |
| Homo_sapiens | SP8 | ENSG00000164651 | zf-C2H2 | ENSP00000408792; | 221833 |
| Homo_sapiens | NR1D2 | ENSG00000174738 | THR-like | ENSP00000373283; | 9975 |
| Homo_sapiens | FLI1 | ENSG00000151702 | ETS | ENSP00000281428; | 2313 |
| Homo_sapiens | REPIN1 | ENSG00000214022 | zf-C2H2 | ENSP00000428562; | 29803 |
| Homo_sapiens | SNAI2 | ENSG00000019549 | zf-C2H2 | ENSP00000494171; | 6591 |
| Homo_sapiens | TFAP2A | ENSG00000137203 | AP-2 | ENSP00000419961; | 7020 |
| Homo_sapiens | ETS2 | ENSG00000157557 | ETS | ENSP00000354194; | 2114 |
| Homo_sapiens | ZNF735 | ENSG00000223614 | zf-C2H2 | ENSP00000485547; | 730291 |
| Homo_sapiens | HOXC5 | ENSG00000172789 | Homeobox | ENSP00000309336; | 3222 |
| Homo_sapiens | SOX9 | ENSG00000125398 | HMG | ENSP00000495527; | 6662 |
| Homo_sapiens | NR2C1 | ENSG00000120798 | RXR-like | ENSP00000376813; | 7181 |
| Homo_sapiens | NFYA | ENSG00000001167 | NF-YA | ENSP00000229418; | 4800 |
| Homo_sapiens | NKX3-2 | ENSG00000109705 | Homeobox | ENSP00000371875; | 579 |
| Homo_sapiens | TTF1 | ENSG00000125482 | MYB | ENSP00000481441; | 7270 |
| Homo_sapiens | NR4A3 | ENSG00000119508 | NGFIB-like | ENSP00000333122; | 8013 |
| Homo_sapiens | ZNF768 | ENSG00000169957 | zf-C2H2 | ENSP00000369777; | 79724 |
| Homo_sapiens | SETDB2 | ENSG00000136169 | MBD | ENSP00000346175; | 83852 |
| Homo_sapiens | FOXI1 | ENSG00000168269 | Fork_head | ENSP00000415483; | 2299 |
| Homo_sapiens | ZNF574 | ENSG00000105732 | zf-C2H2 | ENSP00000351939; | 64763 |
| Homo_sapiens | FOXL1 | ENSG00000176678 | Fork_head | ENSP00000326272; | 2300 |
| Homo_sapiens | ZNF527 | ENSG00000189164 | zf-C2H2 | ENSP00000390179; | 84503 |
| Homo_sapiens | CIC | ENSG00000079432 | HMG | ENSP00000160740; | 23152 |
| Homo_sapiens | HES6 | ENSG00000144485 | bHLH | ENSP00000387155; | 55502 |
| Homo_sapiens | NKX2-6 | ENSG00000180053 | Homeobox | ENSP00000320089; | 137814 |
| Homo_sapiens | NR1D1 | ENSG00000126368 | THR-like | ENSP00000246672; | 9572 |
| Homo_sapiens | ZNF121 | ENSG00000197961 | zf-C2H2 | ENSP00000468643; | 7675 |
| Homo_sapiens | ZBTB7C | ENSG00000184828 | ZBTB | ENSP00000468254; | 201501 |
| Homo_sapiens | ZNF619 | ENSG00000177873 | zf-C2H2 | ENSP00000398024; | 285267 |
| Homo_sapiens | SOX2 | ENSG00000181449 | HMG | ENSP00000323588; | 6657 |
| Homo_sapiens | ZNF131 | ENSG00000172262 | ZBTB | ENSP00000423945; | 7690 |
| Homo_sapiens | E2F1 | ENSG00000101412 | E2F | ENSP00000345571; | 1869 |
| Homo_sapiens | ZNF678 | ENSG00000181450 | zf-C2H2 | ENSP00000344828; | 339500 |
| Homo_sapiens | ZSCAN25 | ENSG00000197037 | zf-C2H2 | ENSP00000262941; | 221785 |
| Homo_sapiens | THAP8 | ENSG00000161277 | THAP | ENSP00000292894; | 199745 |
| Homo_sapiens | ZNF596 | ENSG00000172748 | zf-C2H2 | ENSP00000310033; | 169270 |
| Homo_sapiens | ZNF622 | ENSG00000173545 | zf-C2H2 | ENSP00000310042; | 90441 |
| Homo_sapiens | ZBTB2 | ENSG00000181472 | ZBTB | ENSP00000323183; | 57621 |
| Homo_sapiens | ZSCAN26 | ENSG00000197062 | zf-C2H2 | ENSP00000485228; | 7741 |
| Homo_sapiens | MYF6 | ENSG00000111046 | bHLH | ENSP00000228641; | 4618 |
| Homo_sapiens | ZNF236 | ENSG00000130856 | zf-C2H2 | ENSP00000463787; | 7776 |
| Homo_sapiens | ZNF589 | ENSG00000164048 | zf-C2H2 | ENSP00000404592; | 51385 |
| Homo_sapiens | TWIST2 | ENSG00000233608 | bHLH | ENSP00000405176; | 117581 |
| Homo_sapiens | PATZ1 | ENSG00000100105 | ZBTB | ENSP00000337520; | 23598 |

| | | | | | |
|---|---|---|---|---|---|
| Homo_sapiens | LBX1 | ENSG00000138136 | Homeobox | ENSP00000359212; | 10660 |
| Homo_sapiens | ZBTB40 | ENSG00000184677 | ZBTB | ENSP00000384527; | 9923 |
| Homo_sapiens | ZSCAN16 | ENSG00000196812 | zf-C2H2 | ENSP00000366527; | 80345 |
| Homo_sapiens | ZNF202 | ENSG00000166261 | zf-C2H2 | ENSP00000433881; | 7753 |
| Homo_sapiens | ZNF782 | ENSG00000196597 | zf-C2H2 | ENSP00000419397; | 158431 |
| Homo_sapiens | HSFX3 | ENSG00000283697 | HSF | ENSP00000490928; | 101928917 |
| Homo_sapiens | ZNF721 | ENSG00000182903 | zf-C2H2 | ENSP00000340524; | 170960 |
| Homo_sapiens | ZNF676 | ENSG00000196109 | zf-C2H2 | ENSP00000380310; | 163223 |
| Homo_sapiens | ZXDC | ENSG00000070476 | zf-C2H2 | ENSP00000374359; | 79364 |
| Homo_sapiens | ZNF576 | ENSG00000124444 | zf-C2H2 | ENSP00000435934; | 79177 |
| Homo_sapiens | POU3F1 | ENSG00000185668 | Pou | ENSP00000362103; | 5453 |
| Homo_sapiens | IRF6 | ENSG00000117595 | IRF | ENSP00000355988; | 3664 |
| Homo_sapiens | OSR1 | ENSG00000143867 | zf-C2H2 | ENSP00000272223; | 130497 |
| Homo_sapiens | PRDM14 | ENSG00000147596 | zf-C2H2 | ENSP00000276594; | 63978 |
| Homo_sapiens | ZNF586 | ENSG00000083828 | zf-C2H2 | ENSP00000375583; | 54807 |
| Homo_sapiens | TBX19 | ENSG00000143178 | T-box | ENSP00000356795; | 9095 |
| Homo_sapiens | FOXN2 | ENSG00000170802 | Fork_head | ENSP00000388486; | 3344 |
| Homo_sapiens | EVX2 | ENSG00000174279 | Homeobox | ENSP00000312385; | 344191 |
| Homo_sapiens | ZBED1 | ENSG00000214717 | zf-BED | ENSP00000370621; | 9189 |
| Homo_sapiens | IRX6 | ENSG00000159387 | Homeobox | ENSP00000290552; | 79190 |
| Homo_sapiens | PBX2 | ENSG00000204304 | Homeobox | ENSP00000364190; | 5089 |
| Homo_sapiens | CLOCK | ENSG00000134852 | bHLH | ENSP00000396649; | 9575 |
| Homo_sapiens | TAL1 | ENSG00000162367 | bHLH | ENSP00000294339; | 6886 |
| Homo_sapiens | HELT | ENSG00000187821 | bHLH | ENSP00000422140; | 391723 |
| Homo_sapiens | DLX5 | ENSG00000105880 | Homeobox | ENSP00000222598; | 1749 |
| Homo_sapiens | GTF2IRD2 | ENSG00000196275 | GTF2I | ENSP00000406723; | 84163 |
| Homo_sapiens | HMGB1 | ENSG00000189403 | HMG | ENSP00000369904; | 3146 |
| Homo_sapiens | HMX1 | ENSG00000215612 | Homeobox | ENSP00000383516; | 3166 |
| Homo_sapiens | GBX2 | ENSG00000168505 | Homeobox | ENSP00000302251; | 2637 |
| Homo_sapiens | NFIX | ENSG00000008441 | CTF/NFI | ENSP00000465094; | 4784 |
| Homo_sapiens | ZNF646 | ENSG00000167395 | zf-C2H2 | ENSP00000391271; | 9726 |
| Homo_sapiens | ZNF479 | ENSG00000185177 | zf-C2H2 | ENSP00000333776; | 90827 |
| Homo_sapiens | AC073111.3 | ENSG00000284041 | zf-C2H2 | ENSP00000419336; | - |
| Homo_sapiens | EVX1 | ENSG00000106038 | Homeobox | ENSP00000419266; | 2128 |
| Homo_sapiens | MZF1 | ENSG00000099326 | zf-C2H2 | ENSP00000469493; | 7593 |
| Homo_sapiens | RUNX3 | ENSG00000020633 | Runt | ENSP00000308051; | 864 |
| Homo_sapiens | HOXD12 | ENSG00000170178 | Homeobox | ENSP00000385586; | 3238 |
| Homo_sapiens | JUND | ENSG00000130522 | TF_bZIP | ENSP00000252818; | 3727 |
| Homo_sapiens | RXRB | ENSG00000204231 | RXR-like | ENSP00000363817; | 6257 |
| Homo_sapiens | ZNF208 | ENSG00000160321 | zf-C2H2 | ENSP00000380315; | 7757 |
| Homo_sapiens | ZNF383 | ENSG00000188283 | zf-C2H2 | ENSP00000464871; | 163087 |
| Homo_sapiens | ESRRB | ENSG00000119715 | ESR-like | ENSP00000370270; | 2103 |
| Homo_sapiens | ZNF891 | ENSG00000214029 | zf-C2H2 | ENSP00000437590; | 101060200 |
| Homo_sapiens | ZNF699 | ENSG00000196110 | zf-C2H2 | ENSP00000311596; | 374879 |
| Homo_sapiens | NR2E3 | ENSG00000278570 | RXR-like | ENSP00000482504; | 10002 |
| Homo_sapiens | ZNF335 | ENSG00000198026 | zf-C2H2 | ENSP00000325326; | 63925 |
| Homo_sapiens | STAT1 | ENSG00000115415 | STAT | ENSP00000386244; | 6772 |
| Homo_sapiens | GATAD2A | ENSG00000167491 | zf-GATA | ENSP00000351552; | 54815 |
| Homo_sapiens | ZFPM1 | ENSG00000179588 | zf-C2H2 | ENSP00000326630; | 161882 |
| Homo_sapiens | NFE2L2 | ENSG00000116044 | TF_bZIP | ENSP00000380252; | 4780 |
| Homo_sapiens | ZNF276 | ENSG00000158805 | zf-C2H2 | ENSP00000415836; | 92822 |
| Homo_sapiens | ESRRG | ENSG00000196482 | ESR-like | ENSP00000352077; | 2104 |
| Homo_sapiens | ZNF835 | ENSG00000127903 | zf-C2H2 | ENSP00000444747; | 90485 |
| Homo_sapiens | TP53 | ENSG00000141510 | P53 | ENSP00000425104; | 7157 |
| Homo_sapiens | ZNF514 | ENSG00000144026 | zf-C2H2 | ENSP00000295208; | 84874 |
| Homo_sapiens | ZFP82 | ENSG00000181007 | zf-C2H2 | ENSP00000431265; | 284406 |
| Homo_sapiens | ZNF728 | ENSG00000269067 | zf-C2H2 | ENSP00000471593; | 388523 |
| Homo_sapiens | PBX1 | ENSG00000185630 | Homeobox | ENSP00000453188; | 5087 |

| | | | | | |
|---|---|---|---|---|---|
| Homo_sapiens | NR1I3 | ENSG00000143257 | THR-like | ENSP00000421374; | 9970 |
| Homo_sapiens | ZNF780A | ENSG00000197782 | zf-C2H2 | ENSP00000472189; | 284323 |
| Homo_sapiens | NPAS2 | ENSG00000170485 | bHLH | ENSP00000388528; | 4862 |
| Homo_sapiens | ZNF154 | ENSG00000179909 | zf-C2H2 | ENSP00000469633; | 7710 |
| Homo_sapiens | RERE | ENSG00000142599 | zf-GATA | ENSP00000366684; | 473 |
| Homo_sapiens | NFATC1 | ENSG00000131196 | RHD | ENSP00000466489; | 4772 |
| Homo_sapiens | SALL2 | ENSG00000165821 | zf-C2H2 | ENSP00000483562; | 6297 |
| Homo_sapiens | ATF6 | ENSG00000118217 | TF_bZIP | ENSP00000356919; | 22926 |
| Homo_sapiens | PBX3 | ENSG00000167081 | Homeobox | ENSP00000362588; | 5090 |
| Homo_sapiens | JARID2 | ENSG00000008083 | ARID | ENSP00000341280; | 3720 |
| Homo_sapiens | PKNOX1 | ENSG00000160199 | Homeobox | ENSP00000402243; | 5316 |
| Homo_sapiens | ELF5 | ENSG00000135374 | ETS | ENSP00000484521; | 2001 |
| Homo_sapiens | ZNF714 | ENSG00000160352 | zf-C2H2 | ENSP00000478345; | 148206 |
| Homo_sapiens | NFIB | ENSG00000147862 | CTF/NFI | ENSP00000475362; | 4781 |
| Homo_sapiens | NR0B1 | ENSG00000169297 | Miscellaneous | ENSP00000368246; | 190 |
| Homo_sapiens | ZNF664 | ENSG00000179195 | zf-C2H2 | ENSP00000441405; | 144348 |
| Homo_sapiens | DACH2 | ENSG00000126733 | DACH | ENSP00000420896; | 117154 |
| Homo_sapiens | SOX15 | ENSG00000129194 | HMG | ENSP00000458286; | 6665 |
| Homo_sapiens | RBAK | ENSG00000146587 | zf-C2H2 | ENSP00000275423; | 57786 |
| Homo_sapiens | ZNF14 | ENSG00000105708 | zf-C2H2 | ENSP00000340514; | 7561 |
| Homo_sapiens | CDIP1 | ENSG00000089486 | zf-LITAF-like | ENSP00000382508; | 29965 |
| Homo_sapiens | VSX1 | ENSG00000100987 | Homeobox | ENSP00000386612; | 30813 |
| Homo_sapiens | HOXD13 | ENSG00000128714 | Homeobox | ENSP00000376322; | 3239 |
| Homo_sapiens | ZNF423 | ENSG00000102935 | zf-C2H2 | ENSP00000455426; | 23090 |
| Homo_sapiens | ZNF552 | ENSG00000178935 | zf-C2H2 | ENSP00000375582; | 79818 |
| Homo_sapiens | ZNF730 | ENSG00000183850 | zf-C2H2 | ENSP00000472959; | 100129543 |
| Homo_sapiens | ZNF781 | ENSG00000196381 | zf-C2H2 | ENSP00000351391; | 163115 |
| Homo_sapiens | AC010616.1 | ENSG00000268041 | ETS | ENSP00000491574; | 390937 |
| Homo_sapiens | IRX1 | ENSG00000170549 | Homeobox | ENSP00000305244; | 79192 |
| Homo_sapiens | ARID1A | ENSG00000117713 | ARID | ENSP00000478955; | 8289 |
| Homo_sapiens | ZNF716 | ENSG00000182111 | zf-C2H2 | ENSP00000394248; | 441234 |
| Homo_sapiens | PRDM12 | ENSG00000130711 | zf-C2H2 | ENSP00000253008; | 59335 |
| Homo_sapiens | TSHZ2 | ENSG00000182463 | zf-C2H2 | ENSP00000360552; | 128553 |
| Homo_sapiens | KLF13 | ENSG00000169926 | zf-C2H2 | ENSP00000452609; | 51621 |
| Homo_sapiens | FOXO6 | ENSG00000204060 | Fork_head | ENSP00000493184; | - |
| Homo_sapiens | IRX2 | ENSG00000170561 | Homeobox | ENSP00000372056; | 153572 |
| Homo_sapiens | MYBL1 | ENSG00000185697 | MYB | ENSP00000429633; | 4603 |
| Homo_sapiens | CEBPA | ENSG00000245848 | TF_bZIP | ENSP00000427514; | 1050 |
| Homo_sapiens | ZNF407 | ENSG00000215421 | zf-C2H2 | ENSP00000310359; | 55628 |
| Homo_sapiens | MIXL1 | ENSG00000185155 | Homeobox | ENSP00000355775; | 83881 |
| Homo_sapiens | MEIS1 | ENSG00000143995 | Homeobox | ENSP00000454209; | 4211 |
| Homo_sapiens | ZNF671 | ENSG00000083814 | zf-C2H2 | ENSP00000321848; | 79891 |
| Homo_sapiens | AC138696.1 | ENSG00000264668 | zf-C2H2 | ENSP00000428966; | - |
| Homo_sapiens | ZNF630 | ENSG00000221994 | zf-C2H2 | ENSP00000354683; | 57232 |
| Homo_sapiens | ZNF524 | ENSG00000171443 | zf-C2H2 | ENSP00000301073; | 147807 |
| Homo_sapiens | MEF2B | ENSG00000213999 | SRF | ENSP00000386480; | 100271849 |
| Homo_sapiens | ZNF662 | ENSG00000182983 | zf-C2H2 | ENSP00000329264; | 389114 |
| Homo_sapiens | ZNF534 | ENSG00000198633 | zf-C2H2 | ENSP00000391358; | 147658 |
| Homo_sapiens | PRDM5 | ENSG00000138738 | zf-C2H2 | ENSP00000404832; | 11107 |
| Homo_sapiens | TOX2 | ENSG00000124191 | HMG | ENSP00000390278; | 84969 |
| Homo_sapiens | ZNF415 | ENSG00000170954 | zf-C2H2 | ENSP00000395055; | 55786 |
| Homo_sapiens | ZBTB20 | ENSG00000181722 | ZBTB | ENSP00000420324; | 26137 |
| Homo_sapiens | ZNF740 | ENSG00000139651 | zf-C2H2 | ENSP00000409463; | 283337 |
| Homo_sapiens | SPIC | ENSG00000166211 | ETS | ENSP00000448580; | 121599 |
| Homo_sapiens | TBX15 | ENSG00000092607 | T-box | ENSP00000207157; | 6913 |
| Homo_sapiens | ZNF766 | ENSG00000196214 | zf-C2H2 | ENSP00000409652; | 90321 |
| Homo_sapiens | ZNF25 | ENSG00000175395 | zf-C2H2 | ENSP00000302222; | 219749 |
| Homo_sapiens | PMS1 | ENSG00000064933 | HMG | ENSP00000401064; | 5378 |

| | | | | | |
|---|---|---|---|---|---|
| Homo_sapiens | MYC | ENSG00000136997 | bHLH | ENSP00000367207; | 4609 |
| Homo_sapiens | SIX6 | ENSG00000184302 | Homeobox | ENSP00000328596; | 4990 |
| Homo_sapiens | POU2F3 | ENSG00000137709 | Pou | ENSP00000441687; | 25833 |
| Homo_sapiens | EHF | ENSG00000135373 | ETS | ENSP00000433508; | 26298 |
| Homo_sapiens | ZNF544 | ENSG00000198131 | zf-C2H2 | ENSP00000469635; | 27300 |
| Homo_sapiens | NANOGNB | ENSG00000205857 | Homeobox | ENSP00000492127; | 360030 |
| Homo_sapiens | ZBTB43 | ENSG00000169155 | ZBTB | ENSP00000362563; | 23099 |
| Homo_sapiens | ZNF536 | ENSG00000198597 | zf-C2H2 | ENSP00000465771; | 9745 |
| Homo_sapiens | ZNF440 | ENSG00000171295 | zf-C2H2 | ENSP00000393489; | 126070 |
| Homo_sapiens | RORB | ENSG00000198963 | THR-like | ENSP00000366093; | 6096 |
| Homo_sapiens | ZNF844 | ENSG00000223547 | zf-C2H2 | ENSP00000448588; | 284391 |
| Homo_sapiens | ZNF263 | ENSG00000006194 | zf-C2H2 | ENSP00000461146; | 10127 |
| Homo_sapiens | ZNF846 | ENSG00000196605 | zf-C2H2 | ENSP00000467891; | 100505555 |
| Homo_sapiens | MTA2 | ENSG00000149480 | zf-GATA | ENSP00000278823; | 9219 |
| Homo_sapiens | CBFB | ENSG00000067955 | CBF | ENSP00000415151; | 865 |
| Homo_sapiens | NPAS1 | ENSG00000130751 | bHLH | ENSP00000405290; | 4861 |
| Homo_sapiens | AC022167.5 | ENSG00000283516 | zf-LITAF-like | ENSP00000490446; | - |
| Homo_sapiens | ZNF57 | ENSG00000171970 | zf-C2H2 | ENSP00000478776; | 126295 |
| Homo_sapiens | EN2 | ENSG00000164778 | Homeobox | ENSP00000297375; | 2020 |
| Homo_sapiens | ZFPM2 | ENSG00000169946 | zf-C2H2 | ENSP00000428720; | 23414 |
| Homo_sapiens | HESX1 | ENSG00000163666 | Homeobox | ENSP00000295934; | 8820 |
| Homo_sapiens | SP3 | ENSG00000172845 | zf-C2H2 | ENSP00000492253; | 6670 |
| Homo_sapiens | E2F8 | ENSG00000129173 | E2F | ENSP00000481103; | 79733 |
| Homo_sapiens | ZNF563 | ENSG00000188868 | zf-C2H2 | ENSP00000469879; | 147837 |
| Homo_sapiens | MAFK | ENSG00000198517 | TF_bZIP | ENSP00000385437; | 7975 |
| Homo_sapiens | ZNF569 | ENSG00000196437 | zf-C2H2 | ENSP00000325018; | 148266 |
| Homo_sapiens | ZNF300 | ENSG00000145908 | zf-C2H2 | ENSP00000397178; | 91975 |
| Homo_sapiens | HES7 | ENSG00000179111 | bHLH | ENSP00000314774; | 84667 |
| Homo_sapiens | NR1I2 | ENSG00000144852 | THR-like | ENSP00000336528; | 8856 |
| Homo_sapiens | SOX7 | ENSG00000171056 | HMG | ENSP00000301921; | 83595 |
| Homo_sapiens | ZBTB39 | ENSG00000166860 | ZBTB | ENSP00000300101; | 9880 |
| Homo_sapiens | ZNF398 | ENSG00000197024 | zf-C2H2 | ENSP00000389972; | 57541 |
| Homo_sapiens | ZNF420 | ENSG00000197050 | zf-C2H2 | ENSP00000338770; | 147923 |
| Homo_sapiens | NCOR1 | ENSG00000141027 | MYB | ENSP00000379189; | 9611 |
| Homo_sapiens | SHOX | ENSG00000185960 | Homeobox | ENSP00000370987; | 6473 |
| Homo_sapiens | CEBPE | ENSG00000092067 | TF_bZIP | ENSP00000206513; | 1053 |
| Homo_sapiens | LITAF | ENSG00000189067 | zf-LITAF-like | ENSP00000461813; | 9516 |
| Homo_sapiens | SEBOX | ENSG00000274529 | Homeobox | ENSP00000444503; | 645832 |
| Homo_sapiens | ZBTB11 | ENSG00000066422 | ZBTB | ENSP00000326200; | 27107 |
| Homo_sapiens | FOXP2 | ENSG00000128573 | Fork_head | ENSP00000489229; | 93986 |
| Homo_sapiens | TLX2 | ENSG00000115297 | Homeobox | ENSP00000233638; | 3196 |
| Homo_sapiens | TOX3 | ENSG00000103460 | HMG | ENSP00000219746; | 27324 |
| Homo_sapiens | NR3C2 | ENSG00000151623 | ESR-like | ENSP00000423510; | 4306 |
| Homo_sapiens | POU5F1 | ENSG00000204531 | Pou | ENSP00000484778; | 5460 |
| Homo_sapiens | ZNF573 | ENSG00000189144 | zf-C2H2 | ENSP00000349861; | 126231 |
| Homo_sapiens | ZNF350 | ENSG00000256683 | zf-C2H2 | ENSP00000243644; | 59348 |
| Homo_sapiens | ZNF324 | ENSG00000083812 | zf-C2H2 | ENSP00000471778; | 25799 |
| Homo_sapiens | ZBTB32 | ENSG00000011590 | ZBTB | ENSP00000262630; | 27033 |
| Homo_sapiens | ZNF394 | ENSG00000160908 | zf-C2H2 | ENSP00000337363; | 84124 |
| Homo_sapiens | FOXD4L5 | ENSG00000204779 | Fork_head | ENSP00000366637; | 653427 |
| Homo_sapiens | AHRR | ENSG00000063438 | bHLH | ENSP00000428893; | 57491 |
| Homo_sapiens | PRDM4 | ENSG00000110851 | zf-C2H2 | ENSP00000228437; | 11108 |
| Homo_sapiens | GFI1B | ENSG00000165702 | zf-C2H2 | ENSP00000361195; | 8328 |
| Homo_sapiens | ZNF341 | ENSG00000131061 | zf-C2H2 | ENSP00000344308; | 84905 |
| Homo_sapiens | KLF15 | ENSG00000163884 | zf-C2H2 | ENSP00000296233; | 28999 |
| Homo_sapiens | ZMIZ1 | ENSG00000108175 | zf-MIZ | ENSP00000401558; | 57178 |
| Homo_sapiens | ZSCAN4 | ENSG00000180532 | zf-C2H2 | ENSP00000480210; | 201516 |
| Homo_sapiens | ZNF850 | ENSG00000267041 | zf-C2H2 | ENSP00000464976; | 342892 |

| | | | | | |
|---|---|---|---|---|---|
| Homo_sapiens | ZNF256 | ENSG00000152454 | zf-C2H2 | ENSP00000282308; | 10172 |
| Homo_sapiens | ZBTB46 | ENSG00000130584 | ZBTB | ENSP00000303102; | 140685 |
| Homo_sapiens | HSFY1 | ENSG00000172468 | HSF | ENSP00000342302; | 86614 |
| Homo_sapiens | HSFY2 | ENSG00000169953 | HSF | ENSP00000372303; | 159119 |
| Homo_sapiens | HOXC8 | ENSG00000037965 | Homeobox | ENSP00000040584; | 3224 |
| Homo_sapiens | ZNF33A | ENSG00000189180 | zf-C2H2 | ENSP00000485869; | 7581 |
| Homo_sapiens | ARNTL | ENSG00000133794 | bHLH | ENSP00000431488; | 406 |
| Homo_sapiens | RLF | ENSG00000117000 | zf-C2H2 | ENSP00000361857; | 6018 |
| Homo_sapiens | SOX1 | ENSG00000182968 | HMG | ENSP00000330218; | 6656 |
| Homo_sapiens | DMRT3 | ENSG00000064218 | DM | ENSP00000190165; | 58524 |
| Homo_sapiens | ZNF624 | ENSG00000197566 | zf-C2H2 | ENSP00000310472; | 57547 |
| Homo_sapiens | NPAS4 | ENSG00000174576 | bHLH | ENSP00000492526; | 266743 |
| Homo_sapiens | ZSCAN30 | ENSG00000186814 | zf-C2H2 | ENSP00000329738; | 100101467 |
| Homo_sapiens | GLIS1 | ENSG00000174332 | zf-C2H2 | ENSP00000486112; | 148979 |
| Homo_sapiens | ZNF146 | ENSG00000167635 | zf-C2H2 | ENSP00000400391; | 7705 |
| Homo_sapiens | FOXM1 | ENSG00000111206 | Fork_head | ENSP00000342307; | 2305 |
| Homo_sapiens | AC092329.3 | ENSG00000283201 | zf-C2H2 | ENSP00000478939; | - |
| Homo_sapiens | SIX2 | ENSG00000170577 | Homeobox | ENSP00000304502; | 10736 |
| Homo_sapiens | KLF18 | ENSG00000283039 | zf-C2H2 | ENSP00000489024; | 105378952 |
| Homo_sapiens | MESP1 | ENSG00000166823 | bHLH | ENSP00000300057; | 55897 |
| Homo_sapiens | BHLHE40 | ENSG00000134107 | bHLH | ENSP00000256495; | 8553 |
| Homo_sapiens | ZNF483 | ENSG00000173258 | zf-C2H2 | ENSP00000311679; | 158399 |
| Homo_sapiens | HES3 | ENSG00000173673 | bHLH | ENSP00000367130; | 390992 |
| Homo_sapiens | RUNX1 | ENSG00000159216 | Runt | ENSP00000388189; | 100506403 |
| Homo_sapiens | ZNF430 | ENSG00000118620 | zf-C2H2 | ENSP00000261560; | 80264 |
| Homo_sapiens | MYNN | ENSG00000085274 | ZBTB | ENSP00000326240; | 55892 |
| Homo_sapiens | HSFX4 | ENSG00000283463 | HSF | ENSP00000489814; | 101927685 |
| Homo_sapiens | ZNF711 | ENSG00000147180 | zf-C2H2 | ENSP00000276123; | 7552 |
| Homo_sapiens | TBX5 | ENSG00000089225 | T-box | ENSP00000384152; | 6910 |
| Homo_sapiens | POU6F2 | ENSG00000106536 | Pou | ENSP00000430514; | 11281 |
| Homo_sapiens | RXRA | ENSG00000186350 | RXR-like | ENSP00000419692; | 6256 |
| Homo_sapiens | WDHD1 | ENSG00000198554 | HMG | ENSP00000399349; | 11169 |
| Homo_sapiens | LBX2 | ENSG00000179528 | Homeobox | ENSP00000366789; | 85474 |
| Homo_sapiens | ZNF764 | ENSG00000169951 | zf-C2H2 | ENSP00000252797; | 92595 |
| Homo_sapiens | RFX6 | ENSG00000185002 | RFX | ENSP00000332208; | 222546 |
| Homo_sapiens | SOX17 | ENSG00000164736 | HMG | ENSP00000297316; | 64321 |
| Homo_sapiens | ZFP91-CNTF | ENSG00000255073 | zf-C2H2 | ENSP00000455911; | - |
| Homo_sapiens | ZHX1 | ENSG00000165156 | Homeobox | ENSP00000297857; | 11244 |
| Homo_sapiens | ZNF48 | ENSG00000180035 | zf-C2H2 | ENSP00000324056; | 197407 |
| Homo_sapiens | SP7 | ENSG00000170374 | zf-C2H2 | ENSP00000441367; | 121340 |
| Homo_sapiens | CSRNP1 | ENSG00000144655 | CSRNP_N | ENSP00000273153; | 64651 |
| Homo_sapiens | DLX6 | ENSG00000006377 | Homeobox | ENSP00000451635; | 1750 |
| Homo_sapiens | HSF1 | ENSG00000185122 | HSF | ENSP00000383590; | 3297 |
| Homo_sapiens | ERG | ENSG00000157554 | ETS | ENSP00000288319; | 2078 |
| Homo_sapiens | MAF | ENSG00000178573 | TF_bZIP | ENSP00000327048; | 4094 |
| Homo_sapiens | ZNF404 | ENSG00000176222 | zf-C2H2 | ENSP00000319479; | 342908 |
| Homo_sapiens | AL033529.1 | ENSG00000254553 | ZBTB | ENSP00000455300; | - |
| Homo_sapiens | ZNF275 | ENSG00000063587 | zf-C2H2 | ENSP00000359271; | 105373378 |
| Homo_sapiens | RFX3 | ENSG00000080298 | RFX | ENSP00000351574; | 5991 |
| Homo_sapiens | MAZ | ENSG00000103495 | zf-C2H2 | ENSP00000458201; | 4150 |
| Homo_sapiens | IKZF5 | ENSG00000095574 | zf-C2H2 | ENSP00000478056; | 64376 |
| Homo_sapiens | KLF9 | ENSG00000119138 | zf-C2H2 | ENSP00000366330; | 687 |
| Homo_sapiens | ZNF665 | ENSG00000197497 | zf-C2H2 | ENSP00000469154; | 79788 |
| Homo_sapiens | ZNF682 | ENSG00000197124 | zf-C2H2 | ENSP00000470718; | 91120 |
| Homo_sapiens | ZNF566 | ENSG00000186017 | zf-C2H2 | ENSP00000465343; | 84924 |
| Homo_sapiens | FOXB1 | ENSG00000171956 | Fork_head | ENSP00000379369; | 27023 |
| Homo_sapiens | DBX2 | ENSG00000185610 | Homeobox | ENSP00000331470; | 440097 |
| Homo_sapiens | FOXG1 | ENSG00000176165 | Fork_head | ENSP00000339004; | 2290 |

| | | | | | |
|---|---|---|---|---|---|
| Homo_sapiens | JUNB | ENSG00000171223 | TF_bZIP | ENSP00000303315; | 3726 |
| Homo_sapiens | DPRX | ENSG00000204595 | Homeobox | ENSP00000365838; | 503834 |
| Homo_sapiens | MAFF | ENSG00000185022 | TF_bZIP | ENSP00000416493; | 23764 |
| Homo_sapiens | SRY | ENSG00000184895 | HMG | ENSP00000372547; | 6736 |
| Homo_sapiens | FOXP4 | ENSG00000137166 | Fork_head | ENSP00000362154; | 116113 |
| Homo_sapiens | MYRF | ENSG00000124920 | NDT80/PhoG | ENSP00000265460; | 745 |
| Homo_sapiens | AFF2 | ENSG00000155966 | AF-4 | ENSP00000286437; | 2334 |
| Homo_sapiens | OVOL2 | ENSG00000125850 | zf-C2H2 | ENSP00000278780; | 58495 |
| Homo_sapiens | TGIF2LY | ENSG00000176679 | Homeobox | ENSP00000318502; | 90655 |
| Homo_sapiens | ZSCAN29 | ENSG00000140265 | zf-C2H2 | ENSP00000456883; | 146050 |
| Homo_sapiens | ZNF852 | ENSG00000178917 | zf-C2H2 | ENSP00000389841; | 285346 |
| Homo_sapiens | TCF23 | ENSG00000163792 | bHLH | ENSP00000296096; | 150921 |
| Homo_sapiens | FOSB | ENSG00000125740 | TF_bZIP | ENSP00000466530; | 2354 |
| Homo_sapiens | BHMG1 | ENSG00000237452 | HMG | ENSP00000402674; | 388553 |
| Homo_sapiens | NKX2-2 | ENSG00000125820 | Homeobox | ENSP00000366347; | 4821 |
| Homo_sapiens | POU3F2 | ENSG00000184486 | Pou | ENSP00000329170; | 5454 |
| Homo_sapiens | NR2F6 | ENSG00000160113 | RXR-like | ENSP00000291442; | 2063 |
| Homo_sapiens | BCL11A | ENSG00000119866 | zf-C2H2 | ENSP00000496168; | 53335 |
| Homo_sapiens | AC115220.1 | ENSG00000241149 | zf-C2H2 | ENSP00000492202; | - |
| Homo_sapiens | ZNF268 | ENSG00000090612 | zf-C2H2 | ENSP00000444412; | 10795 |
| Homo_sapiens | TULP4 | ENSG00000130338 | Tub | ENSP00000356064; | 56995 |
| Homo_sapiens | ASCL5 | ENSG00000232237 | bHLH | ENSP00000469019; | 647219 |
| Homo_sapiens | HIC1 | ENSG00000177374 | ZBTB | ENSP00000314080; | 3090 |
| Homo_sapiens | ZBTB45 | ENSG00000119574 | ZBTB | ENSP00000346603; | 84878 |
| Homo_sapiens | ZNF836 | ENSG00000196267 | zf-C2H2 | ENSP00000325038; | 162962 |
| Homo_sapiens | ZNF670 | ENSG00000277462 | zf-C2H2 | ENSP00000355459; | 93474 |
| Homo_sapiens | ZFHX2 | ENSG00000136367 | Homeobox | ENSP00000413418; | 85446 |
| Homo_sapiens | FOXF2 | ENSG00000137273 | Fork_head | ENSP00000259806; | 2295 |
| Homo_sapiens | SMARCE1 | ENSG00000073584 | HMG | ENSP00000495857; | 6605 |
| Homo_sapiens | ZNF789 | ENSG00000198556 | zf-C2H2 | ENSP00000331927; | 285989 |
| Homo_sapiens | MYT1L | ENSG00000186487 | zf-C2HC | ENSP00000384219; | 23040 |
| Homo_sapiens | ZNF24 | ENSG00000172466 | zf-C2H2 | ENSP00000382015; | 7572 |
| Homo_sapiens | TBX3 | ENSG00000135111 | T-box | ENSP00000257567; | 6926 |
| Homo_sapiens | NEUROD4 | ENSG00000123307 | bHLH | ENSP00000242994; | 58158 |
| Homo_sapiens | AR | ENSG00000169083 | ESR-like | ENSP00000484033; | 367 |
| Homo_sapiens | MBD2 | ENSG00000134046 | MBD | ENSP00000381435; | 8932 |
| Homo_sapiens | ZNF189 | ENSG00000136870 | zf-C2H2 | ENSP00000259395; | 7743 |
| Homo_sapiens | PHOX2A | ENSG00000165462 | Homeobox | ENSP00000298231; | 401 |
| Homo_sapiens | ZFP64 | ENSG00000020256 | zf-C2H2 | ENSP00000344615; | 55734 |
| Homo_sapiens | SMAD6 | ENSG00000137834 | MH1 | ENSP00000452955; | 4091 |
| Homo_sapiens | THAP10 | ENSG00000129028 | THAP | ENSP00000249861; | 56906 |
| Homo_sapiens | AC073111.5 | ENSG00000284691 | zf-C2H2 | ENSP00000492887; | - |
| Homo_sapiens | FOXD4L3 | ENSG00000187559 | Fork_head | ENSP00000341961; | 286380 |
| Homo_sapiens | TP73 | ENSG00000078900 | P53 | ENSP00000367537; | 7161 |
| Homo_sapiens | ZNF587B | ENSG00000269343 | zf-C2H2 | ENSP00000472004; | 100293516 |
| Homo_sapiens | HOXB13 | ENSG00000159184 | Homeobox | ENSP00000290295; | 10481 |
| Homo_sapiens | BHLHA9 | ENSG00000205899 | bHLH | ENSP00000375248; | 727857 |
| Homo_sapiens | ZNF184 | ENSG00000096654 | zf-C2H2 | ENSP00000366636; | 7738 |
| Homo_sapiens | ZNF471 | ENSG00000196263 | zf-C2H2 | ENSP00000309161; | 57573 |
| Homo_sapiens | HOXA5 | ENSG00000106004 | Homeobox | ENSP00000222726; | 3202 |
| Homo_sapiens | ZNF362 | ENSG00000160094 | zf-C2H2 | ENSP00000446335; | 149076 |
| Homo_sapiens | ZBTB17 | ENSG00000116809 | ZBTB | ENSP00000438529; | 7709 |
| Homo_sapiens | ZNF160 | ENSG00000170949 | zf-C2H2 | ENSP00000470961; | 90338 |
| Homo_sapiens | GABPA | ENSG00000154727 | ETS | ENSP00000346886; | 2551 |
| Homo_sapiens | ZNF142 | ENSG00000115568 | zf-C2H2 | ENSP00000408643; | 7701 |
| Homo_sapiens | ZNF771 | ENSG00000179965 | zf-C2H2 | ENSP00000323945; | 51333 |
| Homo_sapiens | THAP12 | ENSG00000137492 | THAP | ENSP00000433966; | 5612 |
| Homo_sapiens | HMX3 | ENSG00000188620 | Homeobox | ENSP00000350549; | 340784 |

| | | | | | |
|---|---|---|---|---|---|
| Homo_sapiens | ZNF736 | ENSG00000234444 | zf-C2H2 | ENSP00000347210; | 728927 |
| Homo_sapiens | ZFP14 | ENSG00000142065 | zf-C2H2 | ENSP00000270001; | 57677 |
| Homo_sapiens | RORA | ENSG00000069667 | THR-like | ENSP00000402971; | 6095 |
| Homo_sapiens | PAX4 | ENSG00000106331 | PAX | ENSP00000491782; | 5078 |
| Homo_sapiens | ZBTB4 | ENSG00000174282 | ZBTB | ENSP00000307858; | 57659 |
| Homo_sapiens | HOXC4 | ENSG00000198353 | Homeobox | ENSP00000305973; | 3221 |
| Homo_sapiens | CREB5 | ENSG00000146592 | TF_bZIP | ENSP00000379593; | 9586 |
| Homo_sapiens | POU4F1 | ENSG00000152192 | Pou | ENSP00000366413; | 5457 |
| Homo_sapiens | KLF12 | ENSG00000118922 | zf-C2H2 | ENSP00000366897; | 11278 |
| Homo_sapiens | ZNF345 | ENSG00000251247 | zf-C2H2 | ENSP00000491547; | 25850 |
| Homo_sapiens | TEAD1 | ENSG00000187079 | TEA | ENSP00000334754; | 7003 |
| Homo_sapiens | LMX1B | ENSG00000136944 | Homeobox | ENSP00000347684; | 4010 |
| Homo_sapiens | PIAS1 | ENSG00000033800 | zf-MIZ | ENSP00000249636; | 8554 |
| Homo_sapiens | NR1H2 | ENSG00000131408 | THR-like | ENSP00000472526; | 7376 |
| Homo_sapiens | ZNF324B | ENSG00000249471 | zf-C2H2 | ENSP00000438930; | 388569 |
| Homo_sapiens | MYCN | ENSG00000134323 | bHLH | ENSP00000281043; | 4613 |
| Homo_sapiens | ZNF175 | ENSG00000105497 | zf-C2H2 | ENSP00000262259; | 7728 |
| Homo_sapiens | VENTX | ENSG00000151650 | Homeobox | ENSP00000357556; | 27287 |
| Homo_sapiens | ZNF559-ZNF177 | ENSG00000270011 | zf-C2H2 | ENSP00000445323; | 100529215 |
| Homo_sapiens | VDR | ENSG00000111424 | THR-like | ENSP00000229022; | 7421 |
| Homo_sapiens | ZNF546 | ENSG00000187187 | zf-C2H2 | ENSP00000469540; | 339327 |
| Homo_sapiens | AFF3 | ENSG00000144218 | AF-4 | ENSP00000395068; | 3899 |
| Homo_sapiens | MBD1 | ENSG00000141644 | MBD | ENSP00000381502; | 4152 |
| Homo_sapiens | KLF16 | ENSG00000129911 | zf-C2H2 | ENSP00000483701; | 83855 |
| Homo_sapiens | ZNF737 | ENSG00000237440 | zf-C2H2 | ENSP00000471995; | 100129842 |
| Homo_sapiens | ZNF169 | ENSG00000175787 | zf-C2H2 | ENSP00000378792; | 169841 |
| Homo_sapiens | ZNF695 | ENSG00000197472 | zf-C2H2 | ENSP00000341236; | 57116 |
| Homo_sapiens | SP5 | ENSG00000204335 | zf-C2H2 | ENSP00000364430; | 389058 |
| Homo_sapiens | CAMTA2 | ENSG00000108509 | CG-1 | ENSP00000459519; | 23125 |
| Homo_sapiens | SOX6 | ENSG00000110693 | HMG | ENSP00000324948; | 55553 |
| Homo_sapiens | CAMTA1 | ENSG00000171735 | CG-1 | ENSP00000306522; | 23261 |
| Homo_sapiens | DDIT3 | ENSG00000175197 | TF_bZIP | ENSP00000494844; | 1649 |
| Homo_sapiens | ZNF525 | ENSG00000203326 | zf-C2H2 | ENSP00000419136; | 170958 |
| Homo_sapiens | LEUTX | ENSG00000213921 | Homeobox | ENSP00000380053; | 342900 |
| Homo_sapiens | AC092835.1 | ENSG00000233757 | zf-C2H2 | ENSP00000478941; | - |
| Homo_sapiens | ZKSCAN7 | ENSG00000196345 | zf-C2H2 | ENSP00000405034; | 55888 |
| Homo_sapiens | ZNF285 | ENSG00000267508 | zf-C2H2 | ENSP00000483662; | 26974 |
| Homo_sapiens | RCOR2 | ENSG00000167771 | MYB | ENSP00000301459; | 283248 |
| Homo_sapiens | ARGFX | ENSG00000186103 | Homeobox | ENSP00000335578; | 503582 |
| Homo_sapiens | ZNF30 | ENSG00000168661 | zf-C2H2 | ENSP00000403441; | 90075 |
| Homo_sapiens | MNX1 | ENSG00000130675 | Homeobox | ENSP00000438552; | 3110 |
| Homo_sapiens | MYF5 | ENSG00000111049 | bHLH | ENSP00000228644; | 4617 |
| Homo_sapiens | ZNF354B | ENSG00000178338 | zf-C2H2 | ENSP00000327143; | 117608 |
| Homo_sapiens | NKX3-1 | ENSG00000167034 | Homeobox | ENSP00000370253; | 4824 |
| Homo_sapiens | KLF3 | ENSG00000109787 | zf-C2H2 | ENSP00000261438; | 51274 |
| Homo_sapiens | FOXD4L1 | ENSG00000184492 | Fork_head | ENSP00000302756; | 200350 |
| Homo_sapiens | PITX2 | ENSG00000164093 | Homeobox | ENSP00000484909; | 5308 |
| Homo_sapiens | ZNF772 | ENSG00000197128 | zf-C2H2 | ENSP00000395967; | 400720 |
| Homo_sapiens | ZBTB16 | ENSG00000109906 | ZBTB | ENSP00000376721; | 7704 |
| Homo_sapiens | ZNF575 | ENSG00000176472 | zf-C2H2 | ENSP00000472578; | 284346 |
| Homo_sapiens | ZNF620 | ENSG00000177842 | zf-C2H2 | ENSP00000322265; | 253639 |
| Homo_sapiens | ZNF761 | ENSG00000160336 | zf-C2H2 | ENSP00000481272; | 388561 |
| Homo_sapiens | ZNF793 | ENSG00000188227 | zf-C2H2 | ENSP00000487183; | 390927 |
| Homo_sapiens | ZNF254 | ENSG00000213096 | zf-C2H2 | ENSP00000349494; | 9534 |
| Homo_sapiens | TFAP2B | ENSG00000008196 | AP-2 | ENSP00000377265; | 7021 |
| Homo_sapiens | SNAPC4 | ENSG00000165684 | MYB | ENSP00000298532; | 6621 |
| Homo_sapiens | ZNF675 | ENSG00000197372 | zf-C2H2 | ENSP00000352836; | 171392 |
| Homo_sapiens | ZNF860 | ENSG00000197385 | zf-C2H2 | ENSP00000373274; | 344787 |

| | | | | | |
|---|---|---|---|---|---|
| Homo_sapiens | FOXD3 | ENSG00000187140 | Fork_head | ENSP00000360157; | 27022 |
| Homo_sapiens | ZNF132 | ENSG00000131849 | zf-C2H2 | ENSP00000254166; | 7691 |
| Homo_sapiens | NFE2L3 | ENSG00000050344 | TF_bZIP | ENSP00000056233; | 9603 |
| Homo_sapiens | ZNF510 | ENSG00000081386 | zf-C2H2 | ENSP00000223428; | 22869 |
| Homo_sapiens | HOXC6 | ENSG00000197757 | Homeobox | ENSP00000243108; | 3223 |
| Homo_sapiens | THRB | ENSG00000151090 | THR-like | ENSP00000493709; | 7068 |
| Homo_sapiens | ZNF774 | ENSG00000196391 | zf-C2H2 | ENSP00000346348; | 342132 |
| Homo_sapiens | ZFP28 | ENSG00000196867 | zf-C2H2 | ENSP00000301318; | 140612 |
| Homo_sapiens | NFE2L1 | ENSG00000082641 | TF_bZIP | ENSP00000464446; | 4779 |
| Homo_sapiens | HOXD4 | ENSG00000170166 | Homeobox | ENSP00000302548; | 3233 |
| Homo_sapiens | ZNF681 | ENSG00000196172 | zf-C2H2 | ENSP00000384000; | 148213 |
| Homo_sapiens | NFIL3 | ENSG00000165030 | TF_bZIP | ENSP00000297689; | 4783 |
| Homo_sapiens | FOXP3 | ENSG00000049768 | Fork_head | ENSP00000365380; | 50943 |
| Homo_sapiens | CUX2 | ENSG00000111249 | CUT | ENSP00000261726; | 23316 |
| Homo_sapiens | CREB3 | ENSG00000107175 | TF_bZIP | ENSP00000342136; | 10488 |
| Homo_sapiens | ARNT2 | ENSG00000172379 | bHLH | ENSP00000453792; | 9915 |
| Homo_sapiens | MTA3 | ENSG00000057935 | zf-GATA | ENSP00000385045; | 57504 |
| Homo_sapiens | AEBP2 | ENSG00000139154 | zf-C2H2 | ENSP00000354267; | 121536 |
| Homo_sapiens | TEAD4 | ENSG00000197905 | TEA | ENSP00000444528; | 7004 |
| Homo_sapiens | ZNF250 | ENSG00000196150 | zf-C2H2 | ENSP00000292579; | 58500 |
| Homo_sapiens | FOXD2 | ENSG00000186564 | Fork_head | ENSP00000335493; | 2306 |
| Homo_sapiens | JAZF1 | ENSG00000153814 | zf-C2H2 | ENSP00000283928; | 221895 |
| Homo_sapiens | MYRFL | ENSG00000166268 | NDT80/PhoG | ENSP00000449598; | 196446 |
| Homo_sapiens | THAP1 | ENSG00000131931 | THAP | ENSP00000254250; | 55145 |
| Homo_sapiens | ZNF578 | ENSG00000258405 | zf-C2H2 | ENSP00000459216; | 147660 |
| Homo_sapiens | ZNF461 | ENSG00000197808 | zf-C2H2 | ENSP00000479057; | 92283 |
| Homo_sapiens | IKZF3 | ENSG00000161405 | zf-C2H2 | ENSP00000420463; | 22806 |
| Homo_sapiens | FOXJ2 | ENSG00000065970 | Fork_head | ENSP00000403411; | 55810 |
| Homo_sapiens | AC187653.1 | ENSG00000248767 | Fork_head | ENSP00000492766; | 100288524 |
| Homo_sapiens | ZNF788 | ENSG00000214189 | zf-C2H2 | ENSP00000380866; | - |
| Homo_sapiens | ZNF708 | ENSG00000182141 | zf-C2H2 | ENSP00000469961; | 7562 |
| Homo_sapiens | TERB1 | ENSG00000249961 | MYB | ENSP00000462883; | 283847 |
| Homo_sapiens | ZFP92 | ENSG00000189420 | zf-C2H2 | ENSP00000462054; | 139735 |
| Homo_sapiens | IRF8 | ENSG00000140968 | IRF | ENSP00000455784; | 3394 |
| Homo_sapiens | ZNF705D | ENSG00000215343 | zf-C2H2 | ENSP00000445477; | 728957 |
| Homo_sapiens | MXD3 | ENSG00000213347 | bHLH | ENSP00000425029; | 83463 |
| Homo_sapiens | ZNF506 | ENSG00000081665 | zf-C2H2 | ENSP00000440625; | 440515 |
| Homo_sapiens | RHOXF2 | ENSG00000131721 | Homeobox | ENSP00000360441; | 84528 |
| Homo_sapiens | NHLH2 | ENSG00000177551 | bHLH | ENSP00000322087; | 4808 |
| Homo_sapiens | STAT5B | ENSG00000173757 | STAT | ENSP00000293328; | 6777 |
| Homo_sapiens | ZNF490 | ENSG00000188033 | zf-C2H2 | ENSP00000311521; | 57474 |
| Homo_sapiens | ZNF516 | ENSG00000101493 | zf-C2H2 | ENSP00000478712; | 9658 |
| Homo_sapiens | NFKB2 | ENSG00000077150 | RHD | ENSP00000410256; | 4791 |
| Homo_sapiens | ZNF99 | ENSG00000213973 | zf-C2H2 | ENSP00000380293; | 7652 |
| Homo_sapiens | ESR1 | ENSG00000091831 | ESR-like | ENSP00000384064; | 2099 |
| Homo_sapiens | DMRTA2 | ENSG00000142700 | DM | ENSP00000383909; | 63950 |
| Homo_sapiens | INSM1 | ENSG00000173404 | zf-C2H2 | ENSP00000312631; | 3642 |
| Homo_sapiens | SOX5 | ENSG00000134532 | HMG | ENSP00000439832; | 6660 |
| Homo_sapiens | TBX18 | ENSG00000112837 | T-box | ENSP00000358677; | 9096 |
| Homo_sapiens | HNF4G | ENSG00000164749 | RXR-like | ENSP00000346339; | 3174 |
| Homo_sapiens | ATF1 | ENSG00000123268 | TF_bZIP | ENSP00000262053; | 466 |
| Homo_sapiens | SCRT2 | ENSG00000215397 | zf-C2H2 | ENSP00000246104; | 85508 |
| Homo_sapiens | NKX1-2 | ENSG00000229544 | Homeobox | ENSP00000451945; | 390010 |
| Homo_sapiens | ZNF384 | ENSG00000126746 | zf-C2H2 | ENSP00000473311; | 171017 |
| Homo_sapiens | PLAGL2 | ENSG00000126003 | zf-C2H2 | ENSP00000246229; | 5326 |
| Homo_sapiens | POU4F2 | ENSG00000151615 | Pou | ENSP00000281321; | 5458 |
| Homo_sapiens | NANOG | ENSG00000111704 | Homeobox | ENSP00000435288; | 79923 |
| Homo_sapiens | ZNF81 | ENSG00000197779 | zf-C2H2 | ENSP00000341151; | 347344 |

| | | | | | |
|---|---|---|---|---|---|
| Homo_sapiens | ZNF497 | ENSG00000174586 | zf-C2H2 | ENSP00000311183; | 162968 |
| Homo_sapiens | NR0B2 | ENSG00000131910 | Miscellaneous | ENSP00000254227; | 8431 |
| Homo_sapiens | AC010487.3 | ENSG00000283088 | zf-C2H2 | ENSP00000489045; | - |
| Homo_sapiens | AC004080.3 | ENSG00000257184 | Homeobox | ENSP00000421799; | - |
| Homo_sapiens | ZNF135 | ENSG00000176293 | zf-C2H2 | ENSP00000321406; | 7694 |
| Homo_sapiens | RELB | ENSG00000104856 | RHD | ENSP00000423287; | 5971 |
| Homo_sapiens | GTF2IRD1 | ENSG00000006704 | GTF2I | ENSP00000265755; | 9569 |
| Homo_sapiens | CUX1 | ENSG00000257923 | CUT | ENSP00000451558; | 1523 |
| Homo_sapiens | ZNF207 | ENSG00000010244 | Others | - | 7756 |
| Homo_sapiens | LTF | ENSG00000012223 | Others | - | 4057 |
| Homo_sapiens | PRDM11 | ENSG00000019485 | Others | - | 56981 |
| Homo_sapiens | BCLAF1 | ENSG00000029363 | Others | - | 9774 |
| Homo_sapiens | TFB1M | ENSG00000029639 | Others | - | 51106 |
| Homo_sapiens | ZNF280C | ENSG00000056277 | Others | - | 55609 |
| Homo_sapiens | PARP12 | ENSG00000059378 | Others | - | 64761 |
| Homo_sapiens | GPBP1 | ENSG00000062194 | Others | - | 65056 |
| Homo_sapiens | RFXANK | ENSG00000064490 | Others | - | 8625 |
| Homo_sapiens | MLLT10 | ENSG00000078403 | Others | - | 8028 |
| Homo_sapiens | KHSRP | ENSG00000088247 | Others | - | 8570 |
| Homo_sapiens | HIF1A | ENSG00000100644 | Others | - | 3091 |
| Homo_sapiens | ADNP2 | ENSG00000101544 | Others | - | 22850 |
| Homo_sapiens | CENPT | ENSG00000102901 | Others | - | 80152 |
| Homo_sapiens | CAPN15 | ENSG00000103326 | Others | - | 6650 |
| Homo_sapiens | GABPB1 | ENSG00000104064 | Others | - | 2553 |
| Homo_sapiens | MYEF2 | ENSG00000104177 | Others | - | 50804 |
| Homo_sapiens | TAX1BP1 | ENSG00000106052 | Others | - | 8887 |
| Homo_sapiens | AKNA | ENSG00000106948 | Others | - | 80709 |
| Homo_sapiens | CREBL2 | ENSG00000111269 | Others | - | 1389 |
| Homo_sapiens | CENPA | ENSG00000115163 | Others | - | 1058 |
| Homo_sapiens | CEBPZ | ENSG00000115816 | Others | - | 10153 |
| Homo_sapiens | PLEK | ENSG00000115956 | Others | - | 5341 |
| Homo_sapiens | EPAS1 | ENSG00000116016 | Others | - | 2034 |
| Homo_sapiens | SFPQ | ENSG00000116560 | Others | - | 6421 |
| Homo_sapiens | GON4L | ENSG00000116580 | Others | - | 54856 |
| Homo_sapiens | PHTF1 | ENSG00000116793 | Others | - | 10745 |
| Homo_sapiens | BLZF1 | ENSG00000117475 | Others | - | 8548 |
| Homo_sapiens | ZNF706 | ENSG00000120963 | Others | - | 51123 |
| Homo_sapiens | KIAA1549 | ENSG00000122778 | Others | - | 57670 |
| Homo_sapiens | ZNFX1 | ENSG00000124201 | Others | - | 57169 |
| Homo_sapiens | SLC2A4RG | ENSG00000125520 | Others | - | 56731 |
| Homo_sapiens | RBCK1 | ENSG00000125826 | Others | - | 10616 |
| Homo_sapiens | YEATS4 | ENSG00000127337 | Others | - | 8089 |
| Homo_sapiens | CASZ1 | ENSG00000130940 | Others | - | 54897 |
| Homo_sapiens | CC2D1A | ENSG00000132024 | Others | - | 54862 |
| Homo_sapiens | ZNF414 | ENSG00000133250 | Others | - | 84330 |
| Homo_sapiens | XPA | ENSG00000136936 | Others | - | 7507 |
| Homo_sapiens | TCF19 | ENSG00000137310 | Others | - | 6941 |
| Homo_sapiens | ZNF280D | ENSG00000137871 | Others | - | 54816 |
| Homo_sapiens | PREB | ENSG00000138073 | Others | - | 10113 |
| Homo_sapiens | SMARCAL1 | ENSG00000138375 | Others | - | 50485 |
| Homo_sapiens | CARF | ENSG00000138380 | Others | - | 79800 |
| Homo_sapiens | TCF25 | ENSG00000141002 | Others | - | 22980 |
| Homo_sapiens | ZNF750 | ENSG00000141579 | Others | - | 79755 |
| Homo_sapiens | GABPB2 | ENSG00000143458 | Others | - | 126626 |
| Homo_sapiens | TMF1 | ENSG00000144747 | Others | - | 7110 |
| Homo_sapiens | LYAR | ENSG00000145220 | Others | - | 55646 |
| Homo_sapiens | PURB | ENSG00000146676 | Others | - | 5814 |
| Homo_sapiens | CIZ1 | ENSG00000148337 | Others | - | 25792 |

| | | | | | |
|---|---|---|---|---|---|
| Homo_sapiens | ANKRD30A | ENSG00000148513 | Others | - | 91074 |
| Homo_sapiens | THYN1 | ENSG00000151500 | Others | - | 29087 |
| Homo_sapiens | SETBP1 | ENSG00000152217 | Others | - | 26040 |
| Homo_sapiens | AHCTF1 | ENSG00000153207 | Others | - | 25909 |
| Homo_sapiens | ELMSAN1 | ENSG00000156030 | Others | - | 91748 |
| Homo_sapiens | GATAD1 | ENSG00000157259 | Others | - | 57798 |
| Homo_sapiens | SON | ENSG00000159140 | Others | - | 6651 |
| Homo_sapiens | GPBP1L1 | ENSG00000159592 | Others | - | 60313 |
| Homo_sapiens | ZNF326 | ENSG00000162664 | Others | - | 284695 |
| Homo_sapiens | SPZ1 | ENSG00000164299 | Others | - | 84654 |
| Homo_sapiens | FAM170A | ENSG00000164334 | Others | - | 340069 |
| Homo_sapiens | CREBRF | ENSG00000164463 | Others | - | 153222 |
| Homo_sapiens | ZNF704 | ENSG00000164684 | Others | - | 619279 |
| Homo_sapiens | ZNF503 | ENSG00000165655 | Others | - | 84858 |
| Homo_sapiens | RAG1 | ENSG00000166349 | Others | - | 5896 |
| Homo_sapiens | PHB | ENSG00000167085 | Others | - | 5245 |
| Homo_sapiens | TET2 | ENSG00000168769 | Others | - | 54790 |
| Homo_sapiens | ZNF608 | ENSG00000168916 | Others | - | 57507 |
| Homo_sapiens | ZNF280A | ENSG00000169548 | Others | - | 129025 |
| Homo_sapiens | CENPX | ENSG00000169689 | Others | - | 201254 |
| Homo_sapiens | CNBP | ENSG00000169714 | Others | - | 7555 |
| Homo_sapiens | NFRKB | ENSG00000170322 | Others | - | 4798 |
| Homo_sapiens | ZNF318 | ENSG00000171467 | Others | - | 24149 |
| Homo_sapiens | PURG | ENSG00000172733 | Others | - | 29942 |
| Homo_sapiens | CENPS | ENSG00000175279 | Others | - | 378708 |
| Homo_sapiens | MAF1 | ENSG00000179632 | Others | - | 84232 |
| Homo_sapiens | ZNF609 | ENSG00000180357 | Others | - | 23060 |
| Homo_sapiens | DMRTC1B | ENSG00000184911 | Others | - | 728656 |
| Homo_sapiens | PURA | ENSG00000185129 | Others | - | 5813 |
| Homo_sapiens | NKRF | ENSG00000186416 | Others | - | 55922 |
| Homo_sapiens | ZNF395 | ENSG00000186918 | Others | - | 55893 |
| Homo_sapiens | PLSCR1 | ENSG00000188313 | Others | - | 5359 |
| Homo_sapiens | ZGPAT | ENSG00000197114 | Others | - | 84619 |
| Homo_sapiens | ZNF720 | ENSG00000197302 | Others | - | 107983990 |
| Homo_sapiens | ZNF511 | ENSG00000198546 | Others | - | 118472 |
| Homo_sapiens | L3MBTL3 | ENSG00000198945 | Others | - | 84456 |
| Homo_sapiens | CPEB1 | ENSG00000214575 | Others | - | 64506 |
| Homo_sapiens | ZNF487 | ENSG00000243660 | Others | - | 642819 |
| Homo_sapiens | NME2 | ENSG00000243678 | Others | - | 4831 |
| Homo_sapiens | ZNF488 | ENSG00000265763 | Others | - | 118738 |
| Homo_sapiens | DMRTC1 | ENSG00000269502 | Others | - | 63947 |
| Homo_sapiens | ZNF280B | ENSG00000275004 | Others | - | 140883 |

# Supplementary Table S2

| | logFC | AveExpr | t | P.Value | adj.P.Val | B |
|---|---|---|---|---|---|---|
| UBE2C | 3.41201759 | 5.69188515 | 13.1558921 | 2.09E-33 | 1.04E-30 | 65.0950591 |
| KRT7 | 2.99528215 | 7.77560607 | 4.62730069 | 4.93E-06 | 6.61E-05 | 3.12567825 |
| MYBL2 | 2.98696919 | 4.7399194 | 9.79880447 | 1.41E-20 | 1.70E-18 | 35.8790683 |
| CDC20 | 2.90691209 | 4.98535065 | 10.7978791 | 3.47E-24 | 6.28E-22 | 44.0853203 |
| MMP11 | 2.87817019 | 3.53364566 | 6.10194629 | 2.36E-09 | 6.05E-08 | 10.5090196 |
| TK1 | 2.83528457 | 5.40374817 | 11.8790387 | 2.65E-28 | 7.46E-26 | 53.4632032 |
| KRT14 | 2.82330608 | 4.00942061 | 3.31270123 | 0.00100291 | 0.00744715 | -1.8904316 |
| TPX2 | 2.78243086 | 4.64698273 | 10.5052365 | 4.16E-23 | 6.56E-21 | 41.6326979 |
| SPP1 | 2.77415327 | 4.683812 | 5.54872524 | 5.07E-08 | 1.01E-06 | 7.53232612 |
| TOP2A | 2.60362192 | 4.46344767 | 10.0003031 | 2.74E-21 | 3.62E-19 | 37.4952084 |
| CTA-384D8.3 | 2.59271794 | 3.59122075 | 4.23199221 | 2.84E-05 | 0.00031835 | 1.45590268 |
| KRT18 | 2.53765367 | 7.80134447 | 7.39945039 | 7.36E-13 | 3.24E-11 | 18.3936627 |
| CST6 | 2.53684035 | 3.61715955 | 5.06915002 | 5.97E-07 | 9.76E-06 | 5.15061659 |
| BIRC5 | 2.41413664 | 3.94495541 | 10.1326329 | 9.26E-22 | 1.31E-19 | 38.5676133 |
| CST1 | 2.39745323 | 2.48820586 | 4.53989961 | 7.34E-06 | 9.45E-05 | 2.74482932 |
| ZWINT | 2.38578128 | 4.21119272 | 11.097946 | 2.62E-25 | 5.41E-23 | 46.6396248 |
| AURKB | 2.38156219 | 3.74042219 | 10.3293248 | 1.81E-22 | 2.71E-20 | 40.177389 |
| CDK1 | 2.3808824 | 3.73424012 | 11.7185635 | 1.11E-27 | 2.89E-25 | 52.0417095 |
| CCNB1 | 2.30963398 | 4.57278224 | 10.0582046 | 1.71E-21 | 2.31E-19 | 37.9633797 |
| ASF1B | 2.30527388 | 3.89071965 | 10.992479 | 6.53E-25 | 1.31E-22 | 45.7374004 |
| SERINC2 | 2.30324382 | 6.72402727 | 6.54334255 | 1.73E-10 | 5.39E-09 | 13.0533079 |
| KLHDC7B | 2.27314281 | 3.34597977 | 4.07088421 | 5.58E-05 | 0.00058625 | 0.81462697 |
| KIF2C | 2.27087414 | 3.55176954 | 10.597372 | 1.91E-23 | 3.14E-21 | 42.4006788 |
| CDCA8 | 2.26464366 | 3.56337012 | 10.5027292 | 4.25E-23 | 6.68E-21 | 41.6118531 |
| NUSAP1 | 2.25172744 | 4.00001124 | 9.66955673 | 3.99E-20 | 4.53E-18 | 34.853336 |
| ISG15 | 2.22431253 | 6.86239597 | 6.34699854 | 5.63E-10 | 1.58E-08 | 11.9034099 |
| PRSS8 | 2.22427748 | 4.31630934 | 5.9673207 | 5.08E-09 | 1.23E-07 | 9.76256505 |
| SPINT1 | 2.17960335 | 6.39139929 | 7.63739071 | 1.48E-13 | 7.22E-12 | 19.9684247 |
| EPCAM | 2.17727153 | 5.35004048 | 6.08046491 | 2.67E-09 | 6.78E-08 | 10.388974 |
| RRM2 | 2.17022243 | 3.93334992 | 8.44489009 | 4.85E-16 | 3.35E-14 | 25.5875399 |
| SFN | 2.15805738 | 8.39886302 | 5.00617118 | 8.15E-07 | 1.30E-05 | 4.85193701 |
| FOXM1 | 2.15701092 | 3.39883057 | 8.55717199 | 2.12E-16 | 1.56E-14 | 26.4010892 |
| CCNB2 | 2.15426701 | 3.73599765 | 9.862037 | 8.46E-21 | 1.05E-18 | 36.3840189 |
| S100A14 | 2.14455983 | 6.32715958 | 3.73715183 | 0.00021151 | 0.00191944 | -0.44021046 |
| TROAP | 2.1327216 | 2.85833564 | 10.54932 | 2.87E-23 | 4.66E-21 | 41.9996611 |
| KRT19 | 2.12948103 | 9.58676784 | 4.36695568 | 1.58E-05 | 0.0001888 | 2.01069627 |
| HIST1H1C | 2.12658207 | 6.16201933 | 6.33677257 | 5.98E-10 | 1.67E-08 | 11.8443118 |
| PYCR1 | 2.12563648 | 3.82934531 | 6.67329301 | 7.81E-11 | 2.56E-09 | 13.8300827 |
| CDT1 | 2.12522507 | 3.31397669 | 10.0198333 | 2.34E-21 | 3.12E-19 | 37.6529371 |
| KIF20A | 2.12020545 | 3.1928401 | 9.81370304 | 1.25E-20 | 1.52E-18 | 35.9978588 |
| AURKA | 2.11408281 | 3.39357519 | 10.1173373 | 1.05E-21 | 1.47E-19 | 38.4432163 |
| KIFC1 | 2.11352645 | 3.68551018 | 9.42122581 | 2.87E-19 | 2.96E-17 | 32.9071051 |
| MELK | 2.09260053 | 3.09147729 | 8.59225683 | 1.63E-16 | 1.22E-14 | 26.6568545 |
| HIST1H2BD | 2.09082011 | 4.13344145 | 7.34996317 | 1.02E-12 | 4.41E-11 | 18.070954 |
| PAFAH1B3 | 2.08690356 | 5.42538381 | 9.68901913 | 3.42E-20 | 3.93E-18 | 35.0072398 |
| APOC1 | 2.08316513 | 4.36178864 | 6.1326947 | 1.98E-09 | 5.11E-08 | 10.68147 |
| CA9 | 2.07335664 | 2.75317949 | 4.19382711 | 3.34E-05 | 0.0003689 | 1.30191817 |
| HIST3H2A | 2.06457129 | 4.02809672 | 7.8327977 | 3.84E-14 | 2.04E-12 | 21.2898638 |
| RECQL4 | 2.0573617 | 3.3910884 | 10.2516012 | 3.46E-22 | 5.07E-20 | 39.5390523 |
| CKS2 | 2.045763 | 5.91629931 | 9.68563095 | 3.51E-20 | 4.01E-18 | 34.9804327 |
| TMEM132A | 2.04255287 | 3.39448731 | 9.28675554 | 8.25E-19 | 8.01E-17 | 31.8670222 |
| UBE2T | 2.03949576 | 4.28411362 | 10.5267647 | 3.47E-23 | 5.54E-21 | 41.811792 |
| PLK1 | 2.03867454 | 3.28639667 | 8.94265086 | 1.17E-17 | 9.78E-16 | 29.251018 |
| CEP55 | 2.02949613 | 3.26927572 | 9.21060471 | 1.49E-18 | 1.39E-16 | 31.2824048 |
| MKI67 | 2.02582861 | 3.18431458 | 9.22851647 | 1.30E-18 | 1.22E-16 | 31.4196277 |
| CDCA5 | 2.02385925 | 3.26088908 | 8.9170647 | 1.42E-17 | 1.18E-15 | 29.0591744 |
| HMGB3 | 2.00558731 | 4.52578864 | 10.5418133 | 3.06E-23 | 4.94E-21 | 41.9371098 |

| | | | | | | |
|---|---|---|---|---|---|---|
| C4orf48 | 2.005197 | 3.33754435 | 7.21232127 | 2.54E-12 | 1.03E-10 | 17.1822261 |
| CCNA2 | 1.99954465 | 3.5273234 | 9.08207796 | 4.03E-18 | 3.49E-16 | 30.3029877 |
| IQGAP3 | 1.99179777 | 2.67272213 | 9.54658033 | 1.06E-19 | 1.16E-17 | 33.8854554 |
| LSR | 1.98404166 | 6.45499072 | 7.20572545 | 2.65E-12 | 1.07E-10 | 17.1399668 |
| PTTG1 | 1.9828727 | 4.17919299 | 8.5427951 | 2.36E-16 | 1.72E-14 | 26.296496 |
| KIF18B | 1.97502167 | 2.52160182 | 9.95908861 | 3.84E-21 | 4.98E-19 | 37.1629793 |
| SAPCD2 | 1.94937662 | 2.85269191 | 8.0675537 | 7.38E-15 | 4.41E-13 | 22.9102117 |
| CDH3 | 1.9368922 | 3.84567192 | 4.48355359 | 9.45E-06 | 0.00011867 | 2.50279583 |
| SNORD104 | 1.93070685 | 4.46366753 | 6.97706793 | 1.16E-11 | 4.30E-10 | 15.6937823 |
| NUF2 | 1.9306937 | 2.69914794 | 8.76092209 | 4.64E-17 | 3.63E-15 | 27.8966147 |
| TACSTD2 | 1.92946247 | 8.11063097 | 4.87603991 | 1.53E-06 | 2.30E-05 | 4.24531481 |
| ETV4 | 1.92859548 | 3.01040846 | 6.55809805 | 1.58E-10 | 4.96E-09 | 13.1408826 |
| TACC3 | 1.92733815 | 3.80973568 | 9.46359689 | 2.06E-19 | 2.17E-17 | 33.2368573 |
| HN1 | 1.92305579 | 5.93808036 | 10.6949728 | 8.35E-24 | 1.45E-21 | 43.2184477 |
| NEK2 | 1.91845068 | 2.72794147 | 9.3336896 | 5.71E-19 | 5.66E-17 | 32.2289245 |
| SPAG5 | 1.90415472 | 3.18324464 | 9.70537924 | 2.99E-20 | 3.48E-18 | 35.1367639 |
| RAC3 | 1.89853858 | 2.98851185 | 7.54481038 | 2.77E-13 | 1.29E-11 | 19.3511767 |
| SDC1 | 1.89390571 | 8.14095091 | 5.13275 | 4.35E-07 | 7.29E-06 | 5.45559536 |
| HJURP | 1.89074704 | 2.66231666 | 10.0936576 | 1.28E-21 | 1.77E-19 | 38.2508589 |
| ST14 | 1.8896653 | 5.94149269 | 6.95231167 | 1.36E-11 | 4.98E-10 | 15.5394203 |
| CDC45 | 1.88791547 | 2.88258688 | 9.32473656 | 6.13E-19 | 6.06E-17 | 32.1597959 |
| LAMC2 | 1.88596128 | 4.06600146 | 3.98723559 | 7.87E-05 | 0.0007973 | 0.4907473 |
| STMN1 | 1.88349492 | 5.32496986 | 7.89791313 | 2.44E-14 | 1.34E-12 | 21.7357523 |
| KIF4A | 1.88009359 | 2.765584 | 9.1580435 | 2.24E-18 | 2.01E-16 | 30.8807574 |
| CENPA | 1.87454026 | 2.51307973 | 8.77763645 | 4.09E-17 | 3.23E-15 | 28.0203844 |
| ESM1 | 1.87417861 | 2.05435366 | 6.72121385 | 5.81E-11 | 1.93E-09 | 14.1196511 |
| KRT16 | 1.87382735 | 4.58879711 | 2.6143939 | 0.00925578 | 0.04814837 | -3.91290693 |
| AP1M2 | 1.86949612 | 5.2515032 | 6.42369191 | 3.56E-10 | 1.04E-08 | 12.3491421 |
| E2F1 | 1.85959409 | 3.54443081 | 7.34399534 | 1.06E-12 | 4.57E-11 | 18.0321506 |
| CASP14 | 1.85897303 | 2.21544267 | 3.1403308 | 0.00180525 | 0.01228042 | -2.4320597 |
| CDKN2A | 1.8464782 | 2.50419111 | 3.67808119 | 0.00026511 | 0.00234457 | -0.65187669 |
| MMP1 | 1.8447656 | 3.72922231 | 3.29401181 | 0.00107023 | 0.00787297 | -1.95048823 |
| NCAPH | 1.84405071 | 2.79740559 | 8.96154129 | 1.02E-17 | 8.53E-16 | 29.392898 |
| GJB2 | 1.83952836 | 5.14219576 | 3.5329967 | 0.00045586 | 0.00377051 | -1.15830047 |
| VGLL1 | 1.82762242 | 4.37864676 | 3.53004273 | 0.00046083 | 0.00380679 | -1.16841208 |
| LAD1 | 1.81687594 | 6.22003246 | 5.27409762 | 2.13E-07 | 3.79E-06 | 6.14538471 |
| HIST1H2AE | 1.81636601 | 2.47848491 | 5.66290719 | 2.74E-08 | 5.79E-07 | 8.12689649 |
| TRIP13 | 1.81483794 | 2.98287871 | 7.7537145 | 6.65E-14 | 3.38E-12 | 20.752041 |
| AGRN | 1.80933809 | 5.37552354 | 8.87263112 | 2.00E-17 | 1.64E-15 | 28.7269076 |
| COL7A1 | 1.80820583 | 3.71752267 | 4.85851754 | 1.67E-06 | 2.48E-05 | 4.16472169 |
| CDC6 | 1.80570193 | 2.89472716 | 9.46083431 | 2.10E-19 | 2.20E-17 | 33.2153282 |
| KIAA0101 | 1.8047584 | 3.01728652 | 9.22878575 | 1.30E-18 | 1.22E-16 | 31.421692 |
| MCM2 | 1.80434762 | 4.03000423 | 7.95541743 | 1.63E-14 | 9.19E-13 | 22.1318012 |
| CENPF | 1.80135448 | 2.82025226 | 8.41021114 | 6.25E-16 | 4.23E-14 | 25.3378137 |
| IGFL1 | 1.79744427 | 3.07707714 | 2.74741247 | 0.00626193 | 0.03501726 | -3.56301138 |
| DLGAP5 | 1.79367755 | 2.76039632 | 8.19425535 | 2.99E-15 | 1.87E-13 | 23.7993209 |
| H2AFX | 1.78681303 | 5.61297606 | 9.53237446 | 1.19E-19 | 1.30E-17 | 33.7741624 |
| CLIC3 | 1.78676916 | 4.01407917 | 3.55020611 | 0.00042787 | 0.00356939 | -1.09923298 |
| SCD | 1.78636782 | 5.68022843 | 5.26921073 | 2.18E-07 | 3.88E-06 | 6.12126156 |
| IFI6 | 1.7859501 | 7.26083927 | 3.91456625 | 0.00010544 | 0.00103517 | 0.21444448 |
| C15orf48 | 1.78456333 | 3.67973333 | 4.04094358 | 6.32E-05 | 0.00065414 | 0.69798418 |
| FAM83H | 1.7836104 | 5.13332032 | 8.0047249 | 1.15E-14 | 6.66E-13 | 22.4730876 |
| CDH1 | 1.77810428 | 5.95014884 | 5.0366188 | 7.01E-07 | 1.13E-05 | 4.99592252 |
| CCNE1 | 1.77203 | 3.15456851 | 7.35352233 | 9.99E-13 | 4.32E-11 | 18.0941076 |
| KRT17 | 1.76836343 | 8.36299855 | 2.77125288 | 0.00582893 | 0.03296528 | -3.4985296 |
| UHRF1 | 1.76825077 | 2.4710297 | 9.59211067 | 7.41E-20 | 8.20E-18 | 34.2428728 |
| LMNB1 | 1.76812022 | 4.11934182 | 8.37957653 | 7.81E-16 | 5.23E-14 | 25.1178213 |
| PABPC1L | 1.76322093 | 2.99394909 | 7.32906789 | 1.18E-12 | 5.02E-11 | 17.9351977 |

| | | | | | | |
|---|---|---|---|---|---|---|
| RAB25 | 1.76258948 | 6.2043699 | 4.87515137 | 1.54E-06 | 2.31E-05 | 4.2412218 |
| FXYD3 | 1.76106793 | 6.88042788 | 3.89624398 | 0.00011344 | 0.00110462 | 0.1455256 |
| CENPM | 1.75786825 | 3.09768676 | 8.95471603 | 1.07E-17 | 8.95E-16 | 29.3416121 |
| HIST1H2BH | 1.7552093 | 2.23099744 | 5.41203219 | 1.04E-07 | 1.96E-06 | 6.83433052 |
| SNORD99 | 1.75495501 | 3.22039247 | 7.72560784 | 8.07E-14 | 4.04E-12 | 20.5618824 |
| MAL2 | 1.75484725 | 5.70067211 | 4.6876607 | 3.73E-06 | 5.13E-05 | 3.3925262 |
| MARCKSL1 | 1.75424122 | 6.93987682 | 6.71355799 | 6.09E-11 | 2.02E-09 | 14.0732771 |
| MIR4653 | 1.74628549 | 2.75661451 | 6.99612902 | 1.03E-11 | 3.83E-10 | 15.81293 |
| GRB7 | 1.74092877 | 4.16202048 | 5.77128973 | 1.52E-08 | 3.36E-07 | 8.70088158 |
| SPC24 | 1.74032515 | 2.8253812 | 9.11289389 | 3.18E-18 | 2.78E-16 | 30.5369743 |
| PKP3 | 1.74007399 | 4.90980427 | 6.06797504 | 2.87E-09 | 7.24E-08 | 10.3193394 |
| PVRL4 | 1.73805055 | 5.39048467 | 4.51029907 | 8.38E-06 | 0.00010665 | 2.61733834 |
| ANLN | 1.73328115 | 3.2115664 | 6.83775326 | 2.80E-11 | 9.77E-10 | 14.8308157 |
| RASSF7 | 1.73197608 | 4.57622298 | 7.59396792 | 1.99E-13 | 9.47E-12 | 19.6782054 |
| PRC1 | 1.72719543 | 3.28680361 | 8.25694362 | 1.90E-15 | 1.22E-13 | 24.2429468 |
| COL10A1 | 1.72685084 | 1.72432755 | 4.37041315 | 1.56E-05 | 0.00018638 | 2.02511821 |
| C19orf33 | 1.72384122 | 6.75079408 | 4.1449272 | 4.10E-05 | 0.00044483 | 1.1064992 |
| CTD-2510F5. | 1.72307611 | 2.88144797 | 6.69255543 | 6.94E-11 | 2.29E-09 | 13.9462772 |
| KPNA2 | 1.72295558 | 5.54558526 | 7.93389532 | 1.90E-14 | 1.06E-12 | 21.9833228 |
| MEX3A | 1.71830784 | 2.44082528 | 5.73901437 | 1.81E-08 | 3.95E-07 | 8.52897984 |
| GINS1 | 1.715451 | 2.74579964 | 9.19762031 | 1.65E-18 | 1.52E-16 | 31.1830417 |
| HMGA1 | 1.71238062 | 6.9974225 | 7.21565098 | 2.48E-12 | 1.01E-10 | 17.2035709 |
| SOX4 | 1.70838328 | 4.9751251 | 6.73301913 | 5.40E-11 | 1.80E-09 | 14.191243 |
| PLAU | 1.70684499 | 5.32835488 | 4.51723825 | 8.13E-06 | 0.00010375 | 2.64715778 |
| MDK | 1.69697787 | 6.72464671 | 5.2566787 | 2.33E-07 | 4.11E-06 | 6.05948933 |
| IER5L | 1.6947148 | 3.25359932 | 8.19800192 | 2.91E-15 | 1.82E-13 | 23.8257653 |
| PODXL2 | 1.68874383 | 3.97047693 | 4.66950139 | 4.05E-06 | 5.54E-05 | 3.31191683 |
| GGH | 1.68835338 | 3.64747038 | 6.32043169 | 6.59E-10 | 1.83E-08 | 11.7500378 |
| HILPDA | 1.68622962 | 4.16249659 | 6.09957913 | 2.39E-09 | 6.12E-08 | 10.4957737 |
| GJB6 | 1.68323204 | 4.2092857 | 3.40507932 | 0.00072423 | 0.00562455 | -1.58885126 |
| SLC39A4 | 1.67918779 | 3.13262496 | 8.17752222 | 3.37E-15 | 2.10E-13 | 23.6813203 |
| C1QTNF6 | 1.67717302 | 2.74681776 | 6.52212537 | 1.97E-10 | 6.07E-09 | 12.9276653 |
| CXCL10 | 1.67454649 | 3.70143421 | 3.02978812 | 0.00259643 | 0.01678824 | -2.76486999 |
| KIF11 | 1.67439903 | 3.04650143 | 8.5001988 | 3.23E-16 | 2.30E-14 | 25.9873335 |
| PRR11 | 1.66981997 | 2.7080168 | 7.99766563 | 1.21E-14 | 6.96E-13 | 22.4241307 |
| SKA3 | 1.66364123 | 2.39393104 | 9.6882481 | 3.44E-20 | 3.95E-18 | 35.001139 |
| BUB1B | 1.66310888 | 2.48393428 | 8.76046227 | 4.66E-17 | 3.63E-15 | 27.8932121 |
| GTSE1 | 1.6616768 | 2.30521071 | 9.34905406 | 5.06E-19 | 5.04E-17 | 32.3476589 |
| CD24 | 1.66056337 | 6.75697305 | 4.06552146 | 5.71E-05 | 0.00059766 | 0.79367619 |
| FER1L4 | 1.65862991 | 3.61191303 | 3.40954425 | 0.00071279 | 0.00555788 | -1.57407585 |
| NDC80 | 1.65794048 | 2.74888279 | 7.92681777 | 1.99E-14 | 1.11E-12 | 21.9345607 |
| TYMS | 1.65565162 | 3.96284547 | 6.78928145 | 3.80E-11 | 1.30E-09 | 14.53383 |
| FEN1 | 1.64702981 | 4.44487123 | 7.89334655 | 2.52E-14 | 1.38E-12 | 21.7043923 |
| SQLE | 1.64576628 | 4.10349767 | 7.23476461 | 2.19E-12 | 9.03E-11 | 17.3262461 |
| ESRP1 | 1.63914569 | 4.70936791 | 5.89223779 | 7.75E-09 | 1.80E-07 | 9.35236671 |
| PKMYT1 | 1.63803925 | 2.39588726 | 8.57482756 | 1.86E-16 | 1.38E-14 | 26.5297047 |
| TTK | 1.63727086 | 2.26844463 | 8.90639489 | 1.55E-17 | 1.28E-15 | 28.9792837 |
| CDCA3 | 1.63632424 | 2.34067549 | 9.28443161 | 8.40E-19 | 8.13E-17 | 31.849134 |
| SKA1 | 1.63305751 | 2.3351431 | 8.82076587 | 2.96E-17 | 2.38E-15 | 28.3405101 |
| GATA3 | 1.63247475 | 5.40780509 | 3.31840399 | 0.00098317 | 0.00731728 | -1.87204209 |
| EIF4EBP1 | 1.62440814 | 5.77869568 | 7.09792097 | 5.34E-12 | 2.07E-10 | 16.4535636 |
| DHCR24 | 1.62437442 | 5.65028288 | 4.77933122 | 2.43E-06 | 3.48E-05 | 3.80375001 |
| RNASEH2A | 1.62230208 | 4.36256456 | 9.75214011 | 2.06E-20 | 2.43E-18 | 35.5077371 |
| YDJC | 1.61561173 | 4.25993009 | 9.48169076 | 1.78E-19 | 1.89E-17 | 33.3779659 |
| HIST1H2BK | 1.61501001 | 5.50615822 | 5.00339204 | 8.26E-07 | 1.31E-05 | 4.83883315 |
| NCAPG | 1.61408739 | 2.36915118 | 8.42519314 | 5.60E-16 | 3.81E-14 | 25.4456106 |
| BUB1 | 1.61293623 | 2.64520794 | 8.50828204 | 3.04E-16 | 2.18E-14 | 26.0459173 |
| SLC52A2 | 1.61103822 | 5.09899024 | 11.3038335 | 4.36E-26 | 9.67E-24 | 48.414419 |

| | | | | | | |
|---|---|---|---|---|---|---|
| PBK | 1.60866929 | 2.67478278 | 7.3483521 | 1.03E-12 | 4.46E-11 | 18.0604763 |
| NME1 | 1.60583981 | 4.57252755 | 10.9206456 | 1.21E-24 | 2.35E-22 | 45.1256355 |
| SNORD14E | 1.60511187 | 2.35996309 | 5.27891745 | 2.08E-07 | 3.71E-06 | 6.16919603 |
| PPP1R14BP3 | 1.6050794 | 5.52590015 | 9.1312684 | 2.76E-18 | 2.43E-16 | 30.6767461 |
| HMMR | 1.6035817 | 2.39701261 | 8.01165288 | 1.10E-14 | 6.36E-13 | 22.5211648 |
| CBLC | 1.60207647 | 4.88851776 | 4.59843736 | 5.62E-06 | 7.44E-05 | 2.99917977 |
| CENPU | 1.59543512 | 3.04802622 | 7.98449137 | 1.33E-14 | 7.58E-13 | 22.3328509 |
| FAM64A | 1.59527891 | 2.24648285 | 7.19810831 | 2.78E-12 | 1.12E-10 | 17.0912016 |
| RHPN1 | 1.59510935 | 2.917166 | 5.3285495 | 1.61E-07 | 2.93E-06 | 6.41550011 |
| GRIN2D | 1.59419738 | 1.91632838 | 5.0179279 | 7.69E-07 | 1.23E-05 | 4.90744218 |
| PSORS1C2 | 1.58909685 | 2.03285002 | 3.04645824 | 0.00245962 | 0.01603863 | -2.71541178 |
| ULBP2 | 1.58585418 | 2.23416671 | 6.07309648 | 2.78E-09 | 7.04E-08 | 10.3478784 |
| SLC5A6 | 1.58123409 | 3.66680319 | 8.20883574 | 2.69E-15 | 1.70E-13 | 23.9022832 |
| IGSF9 | 1.57973398 | 2.78175943 | 5.5928917 | 4.00E-08 | 8.15E-07 | 7.76107239 |
| UBE2S | 1.57624657 | 4.08760374 | 7.82824054 | 3.97E-14 | 2.10E-12 | 21.2587609 |
| MEST | 1.57262312 | 3.17138807 | 4.54545234 | 7.15E-06 | 9.25E-05 | 2.7688295 |
| TLCD1 | 1.57126577 | 3.83349475 | 6.63178727 | 1.01E-10 | 3.26E-09 | 13.5806347 |
| RCC1 | 1.57069546 | 4.51769721 | 10.7089494 | 7.42E-24 | 1.30E-21 | 43.3359072 |
| CKS1B | 1.56633258 | 3.90561701 | 8.02151622 | 1.02E-14 | 5.98E-13 | 22.5896651 |
| C19orf48 | 1.56361077 | 4.67474058 | 9.41586487 | 3.00E-19 | 3.07E-17 | 32.8654525 |
| KIF23 | 1.56255574 | 2.45992331 | 8.46511938 | 4.18E-16 | 2.92E-14 | 25.7335504 |
| PCNA | 1.55898425 | 6.6358789 | 8.00792689 | 1.13E-14 | 6.52E-13 | 22.4953042 |
| PPP1R14B | 1.55577377 | 6.22784037 | 10.0644905 | 1.62E-21 | 2.21E-19 | 38.0143054 |
| GINS2 | 1.55455591 | 2.79926634 | 8.2904751 | 1.49E-15 | 9.65E-14 | 24.4812415 |
| U47924.27 | 1.55454565 | 3.97099427 | 4.65918549 | 4.25E-06 | 5.79E-05 | 3.26625015 |
| CDKN3 | 1.55325842 | 2.83858268 | 7.11307934 | 4.84E-12 | 1.89E-10 | 16.5495878 |
| FAM83H-AS | 1.5511262 | 3.89145736 | 5.47081907 | 7.66E-08 | 1.48E-06 | 7.13266248 |
| EVPL | 1.54340614 | 4.82171849 | 4.45180775 | 1.09E-05 | 0.00013482 | 2.3676427 |
| FBXL6 | 1.54300839 | 3.42769687 | 10.3443009 | 1.60E-22 | 2.40E-20 | 40.3007181 |
| TTYH3 | 1.54185612 | 4.43511096 | 9.05219623 | 5.07E-18 | 4.34E-16 | 30.0766046 |
| ECT2 | 1.53887439 | 3.04050054 | 7.26523956 | 1.79E-12 | 7.53E-11 | 17.5223635 |
| POC1A | 1.5374829 | 3.09451337 | 8.45113446 | 4.63E-16 | 3.21E-14 | 25.6325839 |
| KDF1 | 1.53650925 | 3.93308916 | 6.83231122 | 2.90E-11 | 1.01E-09 | 14.797388 |
| MIR4477B | 1.53523677 | 3.02763988 | 5.82860596 | 1.11E-08 | 2.51E-07 | 9.00818073 |
| SKP2 | 1.5349609 | 3.51757671 | 7.73436601 | 7.60E-14 | 3.84E-12 | 20.6210811 |
| PSMG3 | 1.53481695 | 4.73187822 | 10.7783159 | 4.11E-24 | 7.36E-22 | 43.9201579 |
| PAQR4 | 1.53303974 | 2.51671529 | 7.46974329 | 4.60E-13 | 2.08E-11 | 18.8549117 |
| EFNA4 | 1.53062967 | 3.91458715 | 8.78653528 | 3.83E-17 | 3.03E-15 | 28.0863467 |
| KRT8 | 1.52715276 | 7.9509705 | 3.97097158 | 8.40E-05 | 0.00084548 | 0.42849851 |
| RAD51 | 1.52580816 | 2.45567386 | 9.37206747 | 4.23E-19 | 4.23E-17 | 32.5257433 |
| FAM110A | 1.52421127 | 4.02992696 | 9.03211145 | 5.92E-18 | 5.05E-16 | 29.9247262 |
| DHCR7 | 1.52373393 | 4.11466963 | 7.68752079 | 1.05E-13 | 5.20E-12 | 20.3050304 |
| C16orf59 | 1.52172547 | 2.0437409 | 9.39296706 | 3.59E-19 | 3.64E-17 | 32.6877191 |
| MCM4 | 1.52126455 | 4.38330609 | 7.78204539 | 5.46E-14 | 2.83E-12 | 20.9442415 |
| MFSD3 | 1.52073224 | 3.77910467 | 8.23186247 | 2.28E-15 | 1.45E-13 | 24.0651613 |
| MAGEA3 | 1.51975122 | 1.87630064 | 2.90597703 | 0.0038522 | 0.02338514 | -3.12405695 |
| EZH2 | 1.51697349 | 2.77206348 | 8.02951266 | 9.67E-15 | 5.68E-13 | 22.6452454 |
| HIST1H2BJ | 1.5140573 | 2.07437969 | 5.27283199 | 2.14E-07 | 3.81E-06 | 6.13913527 |
| SPC25 | 1.51298883 | 2.35353536 | 8.09651874 | 6.01E-15 | 3.63E-13 | 23.1125769 |
| TMC6 | 1.51006085 | 3.66187549 | 8.44143191 | 4.97E-16 | 3.42E-14 | 25.5626044 |
| PDIA4 | 1.50877151 | 6.55288052 | 9.24346135 | 1.16E-18 | 1.10E-16 | 31.5342567 |
| EFNA1 | 1.50697663 | 5.08771461 | 5.79395955 | 1.34E-08 | 2.99E-07 | 8.82211547 |
| SYNE4 | 1.50005585 | 2.41113795 | 5.10927575 | 4.89E-07 | 8.13E-06 | 5.34263872 |
| S100A11 | 1.49939878 | 10.6769907 | 6.50352848 | 2.20E-10 | 6.72E-09 | 12.8178136 |
| HIST1H2BG | 1.4980427 | 1.9287166 | 4.88248065 | 1.48E-06 | 2.24E-05 | 4.27500379 |
| RAD54L | 1.48906766 | 2.10023763 | 8.48990675 | 3.48E-16 | 2.45E-14 | 25.9127982 |
| KIFC2 | 1.48538474 | 3.20656182 | 5.69529554 | 2.30E-08 | 4.91E-07 | 8.29744541 |
| HIST1H3D | 1.48269777 | 1.87960738 | 5.72261496 | 1.98E-08 | 4.29E-07 | 8.44195103 |

| | | | | | | |
|---|---|---|---|---|---|---|
| TYMP | 1.48186373 | 4.75863427 | 3.76575496 | 0.0001894 | 0.0017426 | -0.33658319 |
| RAD51AP1 | 1.4781889 | 2.43197009 | 7.31312918 | 1.31E-12 | 5.55E-11 | 17.8318454 |
| MAGEA6 | 1.47808323 | 1.71094866 | 3.00150055 | 0.00284466 | 0.01811835 | -2.848201 |
| TSTD1 | 1.47781129 | 5.27463366 | 5.3741246 | 1.27E-07 | 2.36E-06 | 6.64344517 |
| ADAM8 | 1.47405265 | 3.53070975 | 5.24210153 | 2.51E-07 | 4.40E-06 | 5.98779869 |
| RHOV | 1.47360752 | 4.95626737 | 3.92836759 | 9.98E-05 | 0.00098497 | 0.26655653 |
| LRRC45 | 1.47329309 | 3.58062865 | 9.26342443 | 9.89E-19 | 9.50E-17 | 31.6875678 |
| OCIAD2 | 1.47113311 | 4.84987122 | 8.02373487 | 1.01E-14 | 5.90E-13 | 22.605082 |
| SPAG4 | 1.47075403 | 3.04018065 | 5.48733768 | 7.02E-08 | 1.37E-06 | 7.21699488 |
| NXPH4 | 1.46471553 | 1.66820991 | 4.52821185 | 7.73E-06 | 9.91E-05 | 2.69439913 |
| TINAGL1 | 1.46332462 | 5.73895878 | 4.23664636 | 2.78E-05 | 0.00031299 | 1.47476848 |
| JUP | 1.45634376 | 7.33286888 | 5.55773523 | 4.83E-08 | 9.71E-07 | 7.57886335 |
| FAM111B | 1.44977125 | 2.1400243 | 6.5440142 | 1.72E-10 | 5.37E-09 | 13.0572907 |
| ORC6 | 1.44965762 | 2.0787163 | 8.68900613 | 7.95E-17 | 6.09E-15 | 27.3659468 |
| CHMP4C | 1.44887946 | 3.77917396 | 6.24030145 | 1.06E-09 | 2.86E-08 | 11.2906714 |
| CKAP2L | 1.44873336 | 1.94907582 | 7.71165169 | 8.88E-14 | 4.44E-12 | 20.4676537 |
| HIST1H3H | 1.44528857 | 2.07383126 | 4.75968574 | 2.66E-06 | 3.79E-05 | 3.71501984 |
| RCC2 | 1.4452635 | 5.96843372 | 11.3669666 | 2.50E-26 | 5.69E-24 | 48.9621507 |
| FANCI | 1.4447881 | 2.85552571 | 9.14330101 | 2.51E-18 | 2.24E-16 | 30.7683783 |
| RMI2 | 1.44240203 | 3.20319226 | 6.70624097 | 6.37E-11 | 2.11E-09 | 14.0289955 |
| SHMT2 | 1.44128454 | 4.77609471 | 9.67873623 | 3.71E-20 | 4.23E-18 | 34.9259006 |
| HIST1H4I | 1.44028524 | 2.98476485 | 5.63071384 | 3.26E-08 | 6.76E-07 | 7.95820287 |
| FSCN1 | 1.43713517 | 5.80104167 | 4.25484647 | 2.57E-05 | 0.00029185 | 1.54872618 |
| PLA2G2F | 1.43625586 | 2.68922426 | 2.87832877 | 0.00419934 | 0.02510268 | -3.2023013 |
| PSPHP1 | 1.435937 | 1.85174189 | 2.66152833 | 0.00807311 | 0.04296526 | -3.79084345 |
| SAC3D1 | 1.43576258 | 3.31027027 | 8.12792199 | 4.80E-15 | 2.92E-13 | 23.3325758 |
| TNNT1 | 1.4347333 | 1.63793747 | 3.97132416 | 8.39E-05 | 0.00084457 | 0.42984548 |
| UBALD2 | 1.4346103 | 5.9425674 | 8.17126728 | 3.52E-15 | 2.18E-13 | 23.6372561 |
| STAP2 | 1.43317167 | 4.64606548 | 5.72076121 | 2.00E-08 | 4.33E-07 | 8.43212691 |
| CLDN7 | 1.4317118 | 5.5456466 | 4.50188859 | 8.71E-06 | 0.00011032 | 2.58125217 |
| IFI27 | 1.4311838 | 5.178232 | 2.9058479 | 0.00385376 | 0.02339217 | -3.12442406 |
| MAPK13 | 1.42874173 | 4.08119557 | 7.40257981 | 7.21E-13 | 3.18E-11 | 18.414126 |
| CENPW | 1.42829668 | 4.3505957 | 5.96062163 | 5.28E-09 | 1.27E-07 | 9.72578768 |
| CDCA4 | 1.42120367 | 3.70669276 | 7.90257117 | 2.36E-14 | 1.30E-12 | 21.7677542 |
| CNKSR1 | 1.42049254 | 3.49302133 | 6.57054416 | 1.47E-10 | 4.61E-09 | 13.2148759 |
| SMIM22 | 1.41959571 | 4.20659535 | 3.4187301 | 0.00068978 | 0.00540447 | -1.54362027 |
| TIMELESS | 1.41951021 | 3.4203677 | 8.52435266 | 2.70E-16 | 1.96E-14 | 26.1625073 |
| WDR34 | 1.41944994 | 5.07414423 | 6.5569601 | 1.59E-10 | 4.98E-09 | 13.134123 |
| CHI3L1 | 1.41850048 | 2.90207524 | 2.96320921 | 0.00321534 | 0.02007205 | -2.95980791 |
| HGH1 | 1.4169772 | 4.00038267 | 10.9183404 | 1.24E-24 | 2.38E-22 | 45.1060403 |
| PCAT6 | 1.41252328 | 2.88099584 | 6.17943867 | 1.51E-09 | 3.98E-08 | 10.9450187 |
| DTL | 1.40993954 | 2.1569477 | 7.99195365 | 1.26E-14 | 7.21E-13 | 22.3845407 |
| CHTF18 | 1.40924226 | 2.61770448 | 8.78759734 | 3.80E-17 | 3.01E-15 | 28.0942223 |
| MIR25 | 1.40769446 | 2.68828351 | 5.76205282 | 1.60E-08 | 3.51E-07 | 8.65160072 |
| HOXB7 | 1.40074785 | 4.07551675 | 6.17611716 | 1.54E-09 | 4.05E-08 | 10.9262364 |
| MDFI | 1.40061969 | 4.0843067 | 4.34738253 | 1.73E-05 | 0.00020395 | 1.9292486 |
| FAM83A | 1.39702896 | 3.08881673 | 2.8086255 | 0.00520445 | 0.03002391 | -3.39636599 |
| APOBEC3B | 1.39005927 | 2.9158717 | 4.39725272 | 1.39E-05 | 0.00016743 | 2.13742717 |
| TONSL | 1.3886326 | 2.5933305 | 9.23721581 | 1.21E-18 | 1.15E-16 | 31.4863376 |
| MFAP2 | 1.38501824 | 3.61014402 | 4.16780193 | 3.73E-05 | 0.00040791 | 1.19765079 |
| EPN3 | 1.38501483 | 3.6900074 | 4.41343817 | 1.29E-05 | 0.00015712 | 2.20545772 |
| RP11-783K16 | 1.38406223 | 2.46954771 | 6.18424471 | 1.47E-09 | 3.88E-08 | 10.9722106 |
| TRIB3 | 1.38166361 | 3.11854309 | 5.42227466 | 9.88E-08 | 1.87E-06 | 6.8861074 |
| LPAR2 | 1.38118117 | 3.66437308 | 7.44695333 | 5.36E-13 | 2.40E-11 | 18.705001 |
| CBX2 | 1.37869324 | 1.99759802 | 5.11905826 | 4.66E-07 | 7.77E-06 | 5.38965595 |
| CYB561 | 1.37663761 | 4.52636853 | 8.98360947 | 8.58E-18 | 7.26E-16 | 29.5589029 |
| FASN | 1.37252192 | 5.27233835 | 6.08707522 | 2.57E-09 | 6.55E-08 | 10.4258769 |
| SPINT2 | 1.36924975 | 6.85836774 | 5.2391781 | 2.54E-07 | 4.45E-06 | 5.97344229 |

| | | | | | | |
|---|---|---|---|---|---|---|
| COMTD1 | 1.36864879 | 3.71509216 | 6.48414017 | 2.48E-10 | 7.47E-09 | 12.7035604 |
| OXLD1 | 1.36679324 | 4.23491199 | 9.07174586 | 4.37E-18 | 3.76E-16 | 30.2246552 |
| RP13-463N1( | 1.36470201 | 1.51259691 | 4.16808745 | 3.72E-05 | 0.00040757 | 1.19879145 |
| HIST1H2BC | 1.36462016 | 2.06226709 | 4.71140551 | 3.34E-06 | 4.65E-05 | 3.49835502 |
| NMB | 1.36049184 | 3.7934381 | 5.93048772 | 6.26E-09 | 1.48E-07 | 9.56078701 |
| SNHG1 | 1.35605931 | 3.87922741 | 8.49739566 | 3.30E-16 | 2.34E-14 | 25.9670267 |
| PPP1R14C | 1.35519425 | 1.97891695 | 3.43742138 | 0.00064509 | 0.00509603 | -1.48141018 |
| DSP | 1.35206133 | 4.85371384 | 3.15504207 | 0.00171864 | 0.01178463 | -2.38690981 |
| MOGS | 1.35084157 | 5.02074525 | 11.7354158 | 9.58E-28 | 2.52E-25 | 52.1905278 |
| BCL2L12 | 1.35009153 | 3.70294896 | 8.81822711 | 3.01E-17 | 2.42E-15 | 28.3216363 |
| MAD2L1 | 1.34900956 | 2.42071439 | 7.24682627 | 2.02E-12 | 8.38E-11 | 17.4037904 |
| TMEM238 | 1.34892932 | 2.79420908 | 5.5670059 | 4.60E-08 | 9.28E-07 | 7.62681509 |
| ITGB6 | 1.34880246 | 4.00124817 | 3.78012488 | 0.00017913 | 0.00165622 | -0.28424302 |
| LIG1 | 1.3468843 | 3.3513747 | 9.86444758 | 8.30E-21 | 1.03E-18 | 36.4033091 |
| FCRLB | 1.34408869 | 2.07385244 | 3.77744117 | 0.00018101 | 0.00167198 | -0.29403218 |
| CDCP1 | 1.34256128 | 3.57057951 | 5.22236937 | 2.77E-07 | 4.81E-06 | 5.89103438 |
| OIP5 | 1.34144728 | 2.03258408 | 7.7317757 | 7.73E-14 | 3.90E-12 | 20.6035673 |
| F11R | 1.34141987 | 5.65022472 | 5.94101001 | 5.90E-09 | 1.41E-07 | 9.61832225 |
| SLC50A1 | 1.34112479 | 5.00287224 | 7.69281141 | 1.01E-13 | 5.03E-12 | 20.3406522 |
| EBP | 1.34077417 | 4.80260298 | 8.34188344 | 1.03E-15 | 6.76E-14 | 24.8479293 |
| SDF2L1 | 1.33745713 | 4.68387009 | 7.45055017 | 5.23E-13 | 2.35E-11 | 18.7286374 |
| S1PR5 | 1.336646 | 2.28028834 | 4.65149866 | 4.41E-06 | 5.98E-05 | 3.23228117 |
| MMP9 | 1.33648932 | 3.06545303 | 2.7972782 | 0.00538732 | 0.03089573 | -3.42752495 |
| DEPDC1B | 1.33644235 | 1.88278738 | 7.81750705 | 4.27E-14 | 2.25E-12 | 21.1855578 |
| VAMP8 | 1.33570557 | 7.28653551 | 7.0849862 | 5.80E-12 | 2.24E-10 | 16.371753 |
| MMP13 | 1.33216409 | 1.93722626 | 2.94080651 | 0.00345218 | 0.02129108 | -3.0244671 |
| COL11A1 | 1.33166812 | 1.38538059 | 3.85014402 | 0.00013618 | 0.00129946 | -0.02654639 |
| KIF22 | 1.3316136 | 4.34068441 | 7.99696081 | 1.22E-14 | 6.99E-13 | 22.4192445 |
| ORC1 | 1.33144967 | 1.9938178 | 7.57487562 | 2.26E-13 | 1.07E-11 | 19.5509986 |
| B3GNT3 | 1.33063667 | 4.51550471 | 3.46373043 | 0.00058675 | 0.00469293 | -1.3933025 |
| DUS1L | 1.32921947 | 4.67185912 | 9.50950599 | 1.43E-19 | 1.54E-17 | 33.5952293 |
| PLEK2 | 1.32807319 | 3.9284953 | 4.26532569 | 2.46E-05 | 0.00028 | 1.5914414 |
| PALM3 | 1.32641798 | 2.52161956 | 3.0213915 | 0.00266795 | 0.01717652 | -2.78968334 |
| MIR647 | 1.32607725 | 2.70536847 | 5.56745129 | 4.59E-08 | 9.26E-07 | 7.62912059 |
| RACGAP1 | 1.32251643 | 3.40060182 | 6.61701043 | 1.10E-10 | 3.55E-09 | 13.4921313 |
| NFE2L3 | 1.32228149 | 3.03852503 | 5.33474191 | 1.56E-07 | 2.85E-06 | 6.44637206 |
| HIST2H2BE | 1.32093006 | 3.62225443 | 4.55793258 | 6.76E-06 | 8.79E-05 | 2.82286905 |
| SLC29A2 | 1.32030377 | 2.54631852 | 5.82129565 | 1.15E-08 | 2.60E-07 | 8.96884275 |
| SERPINA1 | 1.3197249 | 2.66577739 | 3.08129602 | 0.00219483 | 0.01453935 | -2.61121402 |
| STRA13 | 1.31942353 | 5.16757894 | 8.42039214 | 5.80E-16 | 3.95E-14 | 25.4110521 |
| EXO1 | 1.31520887 | 1.85480452 | 7.80260928 | 4.74E-14 | 2.48E-12 | 21.0840786 |
| RFC4 | 1.3145919 | 3.31617086 | 7.35572909 | 9.85E-13 | 4.27E-11 | 18.1084677 |
| SLC12A8 | 1.31261363 | 1.96638545 | 6.61176653 | 1.14E-10 | 3.65E-09 | 13.4607624 |
| UNC13D | 1.3122717 | 3.23097677 | 4.39808604 | 1.38E-05 | 0.00016685 | 2.1409242 |
| GRHL2 | 1.31179948 | 3.66787213 | 5.27679866 | 2.10E-07 | 3.74E-06 | 6.15872625 |
| RBBP8NL | 1.30907269 | 2.3245344 | 4.40324257 | 1.35E-05 | 0.00016357 | 2.16257709 |
| PPP1R13L | 1.30795783 | 4.45087875 | 5.69772325 | 2.27E-08 | 4.85E-07 | 8.31026276 |
| NUP210 | 1.30695654 | 3.16672771 | 4.35112122 | 1.70E-05 | 0.00020094 | 1.94478017 |
| FZD2 | 1.30668382 | 2.37783306 | 5.63030854 | 3.27E-08 | 6.77E-07 | 7.95608439 |
| PFKFB4 | 1.30184387 | 1.94369831 | 7.19664671 | 2.81E-12 | 1.13E-10 | 17.0818491 |
| BOP1 | 1.30149463 | 4.63773992 | 7.85283102 | 3.34E-14 | 1.79E-12 | 21.4267522 |
| IRF5 | 1.30102611 | 3.07823902 | 5.72246019 | 1.98E-08 | 4.29E-07 | 8.44113069 |
| ABRACL | 1.29958953 | 5.36906626 | 7.21849247 | 2.43E-12 | 9.94E-11 | 17.2217921 |
| ZDHHC12 | 1.29835888 | 4.52947904 | 7.83949173 | 3.67E-14 | 1.96E-12 | 21.3355753 |
| ALDH3B2 | 1.29833439 | 3.65596607 | 3.22533725 | 0.00135531 | 0.00962445 | -2.16839244 |
| GGCT | 1.29774965 | 5.43696648 | 7.58028664 | 2.18E-13 | 1.03E-11 | 19.587026 |
| DSG2 | 1.29688992 | 4.57298539 | 4.54700712 | 7.10E-06 | 9.19E-05 | 2.77555437 |
| POLD1 | 1.29271478 | 3.24302881 | 8.81449264 | 3.10E-17 | 2.48E-15 | 28.2938801 |

| | | | | | | |
|---|---|---|---|---|---|---|
| CAPG | 1.29204243 | 6.61581333 | 5.103498 | 5.04E-07 | 8.35E-06 | 5.31490669 |
| KCNG1 | 1.29100093 | 2.15472057 | 3.34529634 | 0.00089483 | 0.00675103 | -1.78491864 |
| FABP6 | 1.29080558 | 2.60424355 | 3.10306282 | 0.00204298 | 0.01367456 | -2.54553581 |
| RP11-927P21 | 1.29011411 | 2.58600208 | 5.06779731 | 6.01E-07 | 9.82E-06 | 5.14416658 |
| FUT3 | 1.28819236 | 2.74110537 | 3.49966373 | 0.000515 | 0.00419257 | -1.27193854 |
| STXBP2 | 1.28787673 | 3.83169492 | 8.20537922 | 2.76E-15 | 1.74E-13 | 23.8778623 |
| MCM10 | 1.28655144 | 1.81214994 | 7.15431616 | 3.70E-12 | 1.47E-10 | 16.811626 |
| ELMO3 | 1.28578377 | 4.0517405 | 4.62442036 | 4.99E-06 | 6.68E-05 | 3.11302256 |
| AC006262.5 | 1.28524963 | 1.47755289 | 3.47467427 | 0.00056396 | 0.00453366 | -1.35646518 |
| CXCL11 | 1.28251563 | 1.94523806 | 2.87951589 | 0.00418387 | 0.02503418 | -3.19895652 |
| ALG3 | 1.28157284 | 4.43159261 | 10.8308859 | 2.62E-24 | 4.82E-22 | 44.3643656 |
| SNRPB | 1.2811723 | 7.69021648 | 8.94090832 | 1.19E-17 | 9.90E-16 | 29.2379406 |
| MYCL | 1.28050386 | 3.85044388 | 3.03830467 | 0.00252569 | 0.01640531 | -2.73963488 |
| FERMT1 | 1.2802768 | 3.54206716 | 4.97395524 | 9.54E-07 | 1.50E-05 | 4.70043385 |
| DTYMK | 1.27985742 | 3.95642271 | 8.45130675 | 4.62E-16 | 3.21E-14 | 25.6338271 |
| DNMT3B | 1.27904547 | 2.03263065 | 5.91004638 | 7.02E-09 | 1.65E-07 | 9.44926187 |
| TRAF4 | 1.27904263 | 5.07458524 | 5.96627026 | 5.11E-09 | 1.24E-07 | 9.75679588 |
| ASPM | 1.27826994 | 1.75900865 | 7.00696809 | 9.58E-12 | 3.59E-10 | 15.880798 |
| TCF19 | 1.27054261 | 3.52133377 | 5.41598313 | 1.02E-07 | 1.93E-06 | 6.8542929 |
| CALR | 1.26923946 | 8.68012274 | 10.1742014 | 6.57E-22 | 9.43E-20 | 38.9062608 |
| MEN1 | 1.26815077 | 3.88713191 | 11.842088 | 3.69E-28 | 1.02E-25 | 53.1350313 |
| LLGL2 | 1.26743247 | 3.76209504 | 5.1045115 | 5.01E-07 | 8.31E-06 | 5.31976929 |
| CDCA7 | 1.26533228 | 2.4300262 | 5.29845374 | 1.88E-07 | 3.38E-06 | 6.26590605 |
| SLC35A2 | 1.26517702 | 4.15744645 | 8.39027644 | 7.23E-16 | 4.86E-14 | 25.1945941 |
| SHCBP1 | 1.26270888 | 2.12311165 | 7.01174994 | 9.29E-12 | 3.49E-10 | 15.9107657 |
| TMEM160 | 1.26207389 | 3.76363303 | 6.68283852 | 7.37E-11 | 2.42E-09 | 13.8876291 |
| SBK1 | 1.26197165 | 1.79524039 | 3.82202156 | 0.00015211 | 0.00143431 | -0.13057805 |
| PSAT1 | 1.26106864 | 3.780597 | 4.85888654 | 1.66E-06 | 2.48E-05 | 4.16641618 |
| RP11-329L6.: | 1.26070154 | 2.14063362 | 6.21150243 | 1.25E-09 | 3.34E-08 | 11.1267639 |
| P3H4 | 1.25830486 | 3.35673942 | 6.04714709 | 3.23E-09 | 8.08E-08 | 10.2034854 |
| TBX1 | 1.25820086 | 2.2421235 | 3.22256399 | 0.00136818 | 0.00970404 | -2.17710021 |
| AUNIP | 1.25592275 | 1.83378963 | 7.75655559 | 6.52E-14 | 3.33E-12 | 20.7712917 |
| KIF15 | 1.25551505 | 1.8651413 | 7.29956833 | 1.43E-12 | 6.04E-11 | 17.7440495 |
| PSRC1 | 1.25454373 | 2.34820394 | 7.25796104 | 1.88E-12 | 7.84E-11 | 17.4754652 |
| CDC25C | 1.25365562 | 1.70199007 | 8.30678206 | 1.33E-15 | 8.62E-14 | 24.59738 |
| ARHGAP11A | 1.25238159 | 2.20143142 | 7.35403302 | 9.96E-13 | 4.31E-11 | 18.0974306 |
| HIST1H2AD | 1.25213582 | 1.38600815 | 5.08672359 | 5.47E-07 | 9.01E-06 | 5.23455026 |
| SGOL1 | 1.25187885 | 1.68667745 | 7.79950603 | 4.84E-14 | 2.54E-12 | 21.0629584 |
| C17orf53 | 1.25175581 | 2.01904916 | 8.59761907 | 1.57E-16 | 1.18E-14 | 26.6960096 |
| ESRP2 | 1.25133854 | 3.91033045 | 4.47396336 | 9.87E-06 | 0.00012342 | 2.46187474 |
| RP3-523K23. | 1.24854605 | 3.2160762 | 4.83245741 | 1.89E-06 | 2.77E-05 | 4.04533973 |
| SLC26A6 | 1.2463748 | 2.13936105 | 8.42869232 | 5.46E-16 | 3.72E-14 | 25.4708072 |
| MIS18A | 1.24623171 | 3.6414706 | 7.91830351 | 2.12E-14 | 1.18E-12 | 21.875943 |
| CKAP2 | 1.24506507 | 3.20754333 | 7.04960603 | 7.29E-12 | 2.77E-10 | 16.1485793 |
| MTFR2 | 1.24474278 | 1.8169714 | 8.48189293 | 3.69E-16 | 2.60E-14 | 25.8548063 |
| MTHFD1L | 1.24460287 | 2.90685801 | 7.11890656 | 4.66E-12 | 1.82E-10 | 16.5865445 |
| DDX39A | 1.2427325 | 4.69313374 | 9.28674969 | 8.25E-19 | 8.01E-17 | 31.8669771 |
| KNSTRN | 1.24218896 | 2.70030097 | 7.97860248 | 1.38E-14 | 7.88E-13 | 22.2920848 |
| CDC25A | 1.24015963 | 2.04269547 | 7.57834286 | 2.21E-13 | 1.05E-11 | 19.5740818 |
| DEPDC1 | 1.23877145 | 1.76190844 | 6.86789297 | 2.32E-11 | 8.24E-10 | 15.0163343 |
| RP11-480I12. | 1.2385696 | 1.85012405 | 4.84614371 | 1.77E-06 | 2.61E-05 | 4.10796529 |
| KRTCAP3 | 1.23829463 | 4.16135875 | 3.27407885 | 0.00114664 | 0.00834465 | -2.01418509 |
| RP11-532F12 | 1.23762697 | 3.41821597 | 5.094703 | 5.26E-07 | 8.68E-06 | 5.27274578 |
| HIST1H1PS1 | 1.23585675 | 1.40887404 | 4.91736402 | 1.26E-06 | 1.92E-05 | 4.43640875 |
| SLC25A39 | 1.2357065 | 5.8845699 | 9.86901214 | 8.00E-21 | 1.00E-18 | 36.4398443 |
| NUDT8 | 1.23474504 | 2.96523184 | 5.92359873 | 6.50E-09 | 1.54E-07 | 9.52316517 |
| PRSS22 | 1.23405729 | 3.91949721 | 3.69561288 | 0.000248 | 0.00221202 | -0.5893857 |
| KAT2A | 1.23047186 | 4.67652549 | 8.08198306 | 6.66E-15 | 4.00E-13 | 23.0109565 |

| | | | | | | |
|---|---|---|---|---|---|---|
| ATAD2 | 1.2297874 | 3.02515346 | 6.35072332 | 5.51E-10 | 1.55E-08 | 11.9249558 |
| CCNF | 1.22844049 | 2.55612863 | 8.41177799 | 6.18E-16 | 4.18E-14 | 25.3490809 |
| PPP1R35 | 1.22806394 | 4.25906891 | 7.89499592 | 2.49E-14 | 1.36E-12 | 21.7157174 |
| TMEM206 | 1.22780759 | 2.55809736 | 8.90836915 | 1.52E-17 | 1.26E-15 | 28.9940611 |
| GALE | 1.22476976 | 4.15652281 | 6.82395763 | 3.06E-11 | 1.06E-09 | 14.7461177 |
| POLR2H | 1.22383629 | 4.49438662 | 10.8828046 | 1.68E-24 | 3.18E-22 | 44.8042681 |
| OAS3 | 1.22260824 | 3.82059363 | 4.66833967 | 4.08E-06 | 5.57E-05 | 3.30676956 |
| TSTA3 | 1.22070637 | 4.96781418 | 8.0246385 | 1.00E-14 | 5.87E-13 | 22.6113621 |
| SYNGR2 | 1.21896064 | 5.99085543 | 7.54595514 | 2.75E-13 | 1.29E-11 | 19.358774 |
| SNHG12 | 1.21641943 | 2.95399291 | 7.74738565 | 6.94E-14 | 3.53E-12 | 20.7091773 |
| CDCA2 | 1.21274058 | 1.71909444 | 7.22581769 | 2.32E-12 | 9.52E-11 | 17.2687914 |
| OAS1 | 1.2125994 | 4.34958689 | 4.26988822 | 2.41E-05 | 0.00027521 | 1.61006928 |
| LMNB2 | 1.21232866 | 4.57275143 | 7.07818926 | 6.06E-12 | 2.33E-10 | 16.3288104 |
| NUDT1 | 1.2122017 | 3.73575083 | 7.57872524 | 2.20E-13 | 1.04E-11 | 19.576628 |
| MBOAT7 | 1.21017581 | 5.31805316 | 6.37935537 | 4.65E-10 | 1.33E-08 | 12.0909255 |
| INHBA | 1.20889873 | 1.99188928 | 3.74301889 | 0.00020679 | 0.00188388 | -0.41901479 |
| RP11-50C13. | 1.2074554 | 2.76275123 | 4.61478589 | 5.22E-06 | 6.95E-05 | 3.07074208 |
| CCDC167 | 1.20636727 | 4.86746107 | 7.45369353 | 5.12E-13 | 2.30E-11 | 18.7493009 |
| POP7 | 1.20594725 | 5.22394558 | 9.57051584 | 8.80E-20 | 9.66E-18 | 34.0732149 |
| RP6-65G23.3 | 1.20585444 | 2.31150205 | 6.39627513 | 4.20E-10 | 1.21E-08 | 12.1892927 |
| CENPH | 1.20419608 | 2.74976341 | 7.61629718 | 1.71E-13 | 8.28E-12 | 19.8272877 |
| TMSB10 | 1.20254262 | 11.2492347 | 5.38395692 | 1.21E-07 | 2.25E-06 | 6.69284393 |
| PROM2 | 1.20155744 | 4.79286799 | 3.07220632 | 0.00226127 | 0.01491512 | -2.63851004 |
| RHBDF2 | 1.20089845 | 2.94392781 | 7.56415471 | 2.43E-13 | 1.14E-11 | 19.4796751 |
| C1orf233 | 1.1990952 | 3.02946572 | 4.60467509 | 5.46E-06 | 7.25E-05 | 3.02645703 |
| ISYNA1 | 1.19892519 | 4.62727499 | 3.67173452 | 0.00027157 | 0.002394 | -0.6744304 |
| PLXNA1 | 1.1981396 | 3.63307915 | 6.78781695 | 3.84E-11 | 1.31E-09 | 14.5248835 |
| DBNDD1 | 1.19736684 | 2.29557562 | 4.82375003 | 1.97E-06 | 2.87E-05 | 4.00557921 |
| MRPL12 | 1.1969183 | 5.15537313 | 7.30794546 | 1.35E-12 | 5.74E-11 | 17.79827 |
| TMUB1 | 1.19576024 | 4.96776843 | 8.77900379 | 4.05E-17 | 3.20E-15 | 28.0305167 |
| PLK4 | 1.19562535 | 1.96610624 | 8.19328619 | 3.01E-15 | 1.88E-13 | 23.7924816 |
| PYCRL | 1.19359071 | 3.03969831 | 7.86495914 | 3.07E-14 | 1.65E-12 | 21.5097508 |
| MIR205HG | 1.19291333 | 4.11854887 | 2.89524916 | 0.00398366 | 0.02405565 | -3.15450211 |
| TMEM74B | 1.19242167 | 1.61320196 | 4.83243086 | 1.89E-06 | 2.77E-05 | 4.0452184 |
| STEAP3 | 1.19080628 | 3.76189449 | 5.2111927 | 2.93E-07 | 5.06E-06 | 5.83636755 |
| MCM7 | 1.19053834 | 5.35371476 | 7.23234927 | 2.22E-12 | 9.15E-11 | 17.31073 |
| PRRG2 | 1.18838614 | 3.1808538 | 5.53430184 | 5.47E-08 | 1.09E-06 | 7.45796419 |
| EPHA1 | 1.18675554 | 3.33665758 | 4.68372609 | 3.80E-06 | 5.22E-05 | 3.37503652 |
| RP3-416H24. | 1.18554915 | 2.8143828 | 3.13766918 | 0.00182134 | 0.0123793 | -2.44020688 |
| BAIAP2L1 | 1.18485507 | 3.95127939 | 6.38160328 | 4.58E-10 | 1.31E-08 | 12.1039819 |
| YIF1B | 1.18428202 | 3.92806049 | 7.53521411 | 2.96E-13 | 1.37E-11 | 19.2875248 |
| RTKN | 1.18185291 | 2.98315613 | 5.26959918 | 2.18E-07 | 3.87E-06 | 6.12317834 |
| HIST1H2AC | 1.18152143 | 4.27129927 | 4.11687034 | 4.61E-05 | 0.0004946 | 0.99532953 |
| ITPR3 | 1.18055258 | 3.17616575 | 5.33958141 | 1.52E-07 | 2.78E-06 | 6.47052095 |
| BID | 1.17969903 | 3.73301677 | 7.86796312 | 3.01E-14 | 1.62E-12 | 21.5303232 |
| DONSON | 1.17965974 | 2.81765712 | 7.06726211 | 6.51E-12 | 2.50E-10 | 16.2598415 |
| IGFBP3 | 1.17927112 | 6.9630687 | 2.6518981 | 0.00830309 | 0.04398804 | -3.81595426 |
| PCK2 | 1.17819799 | 3.85651976 | 6.68834087 | 7.12E-11 | 2.34E-09 | 13.920831 |
| ITGB4 | 1.17746505 | 6.01215578 | 3.60906899 | 0.00034385 | 0.0029481 | -0.89515653 |
| ACTL6A | 1.17698669 | 4.26952642 | 8.28918383 | 1.51E-15 | 9.73E-14 | 24.4720521 |
| MRPS12 | 1.17454954 | 4.67294091 | 7.18906377 | 2.95E-12 | 1.19E-10 | 17.0333505 |
| EPS8L2 | 1.17400592 | 4.557508 | 5.01648736 | 7.74E-07 | 1.24E-05 | 4.90063497 |
| FANCD2 | 1.17316703 | 2.1176386 | 7.89941497 | 2.41E-14 | 1.33E-12 | 21.7460688 |
| DSN1 | 1.17128393 | 3.46253548 | 7.63486394 | 1.50E-13 | 7.34E-12 | 19.9515025 |
| CHAF1B | 1.17015195 | 2.13317954 | 6.92490361 | 1.62E-11 | 5.85E-10 | 15.3690327 |
| SLC1A6 | 1.17004334 | 1.19272269 | 2.95077077 | 0.00334494 | 0.02076785 | -2.99576617 |
| SEZ6L2 | 1.1691057 | 3.03123704 | 3.53132053 | 0.00045867 | 0.00379058 | -1.16403909 |
| B4GALNT4 | 1.16868892 | 1.76667881 | 3.39927014 | 0.00073937 | 0.00572537 | -1.60804766 |

| | | | | | | |
|---|---|---|---|---|---|---|
| TRAIP | 1.16850167 | 2.01430372 | 8.25641882 | 1.91E-15 | 1.22E-13 | 24.2392228 |
| LYPLA2 | 1.16836663 | 5.81793769 | 8.57609422 | 1.84E-16 | 1.37E-14 | 26.538939 |
| AP1S1 | 1.16654762 | 5.46803354 | 8.4578574 | 4.41E-16 | 3.07E-14 | 25.6811065 |
| LY6E | 1.16578401 | 6.52576399 | 3.08638436 | 0.00215843 | 0.01432909 | -2.59590026 |
| RP11-295G2( | 1.16506218 | 2.66303047 | 5.92608614 | 6.41E-09 | 1.52E-07 | 9.53674506 |
| MIF | 1.1636419 | 5.71906667 | 6.28281019 | 8.23E-10 | 2.26E-08 | 11.5337578 |
| GPR56 | 1.16312539 | 4.63630954 | 4.97684383 | 9.40E-07 | 1.48E-05 | 4.71398263 |
| CEP131 | 1.16206245 | 3.24277644 | 7.28864711 | 1.54E-12 | 6.48E-11 | 17.6734351 |
| B4GALT3 | 1.1606602 | 4.35774775 | 6.61571258 | 1.11E-10 | 3.57E-09 | 13.4843657 |
| CDC7 | 1.16018247 | 2.22738206 | 6.8232088 | 3.07E-11 | 1.06E-09 | 14.7415242 |
| NUDT5 | 1.15894943 | 3.90133715 | 8.56015836 | 2.07E-16 | 1.53E-14 | 26.4228309 |
| CCDC137 | 1.15866178 | 4.24804666 | 9.47220401 | 1.92E-19 | 2.03E-17 | 33.3039597 |
| DPP3 | 1.15730235 | 4.32426445 | 9.12881072 | 2.81E-18 | 2.47E-16 | 30.65804 |
| C21orf58 | 1.15631021 | 1.57979257 | 7.17482559 | 3.24E-12 | 1.30E-10 | 16.9423949 |
| CTSV | 1.15517365 | 1.58686979 | 3.78219156 | 0.0001777 | 0.00164505 | -0.27670016 |
| WDR72 | 1.15510544 | 2.06433224 | 3.74472143 | 0.00020544 | 0.00187419 | -0.41285825 |
| PFDN2 | 1.15496667 | 6.82358378 | 6.33167323 | 6.17E-10 | 1.72E-08 | 11.814871 |
| MROH6 | 1.15127241 | 2.59498771 | 4.47173133 | 9.97E-06 | 0.00012439 | 2.45236221 |
| MANF | 1.15125316 | 5.45782486 | 7.82935618 | 3.94E-14 | 2.09E-12 | 21.266374 |
| PROSER2 | 1.15102397 | 2.51248762 | 4.90089819 | 1.36E-06 | 2.06E-05 | 4.36009375 |
| SLC9A3R1 | 1.15020976 | 5.0256759 | 4.7134425 | 3.31E-06 | 4.61E-05 | 3.50745619 |
| SLC19A1 | 1.14944402 | 1.95327191 | 7.75894754 | 6.41E-14 | 3.27E-12 | 20.787503 |
| MCM5 | 1.14876158 | 3.72453355 | 6.81666803 | 3.20E-11 | 1.10E-09 | 14.7014187 |
| ARHGAP39 | 1.14847609 | 2.07860133 | 8.92139508 | 1.38E-17 | 1.14E-15 | 29.0916169 |
| TINCR | 1.14811721 | 2.32584524 | 3.62705081 | 0.00032145 | 0.00277395 | -0.83218456 |
| NCAPD2 | 1.1480728 | 4.21176583 | 6.20936801 | 1.27E-09 | 3.38E-08 | 11.1146412 |
| KIAA1522 | 1.14761106 | 5.51560785 | 5.10554706 | 4.98E-07 | 8.27E-06 | 5.32473862 |
| KLC3 | 1.14541806 | 1.54437809 | 4.60949576 | 5.34E-06 | 7.10E-05 | 3.04756051 |
| MIIP | 1.14507781 | 3.87269324 | 7.99820305 | 1.21E-14 | 6.95E-13 | 22.4278567 |
| HOMER3 | 1.14417356 | 3.9440431 | 5.93823626 | 5.99E-09 | 1.43E-07 | 9.60314721 |
| MGME1 | 1.14403582 | 3.81373 | 9.15885492 | 2.23E-18 | 2.01E-16 | 30.8869462 |
| SLC16A3 | 1.14193427 | 3.89951116 | 4.26914714 | 2.42E-05 | 0.00027601 | 1.60704233 |
| ARHGEF19 | 1.14162039 | 3.49097537 | 4.86291384 | 1.63E-06 | 2.44E-05 | 4.18491783 |
| PQLC2 | 1.14070146 | 3.23406047 | 10.0935178 | 1.28E-21 | 1.77E-19 | 38.2497243 |
| STIL | 1.14063319 | 1.61272533 | 7.7742785 | 5.77E-14 | 2.98E-12 | 20.8914975 |
| SLC39A11 | 1.13831468 | 3.40835305 | 7.21421573 | 2.50E-12 | 1.02E-10 | 17.1943694 |
| AC098973.2 | 1.13756977 | 1.77634429 | 2.99487497 | 0.00290585 | 0.01842173 | -2.86761073 |
| TREM2 | 1.13629665 | 2.51007096 | 3.76056042 | 0.00019325 | 0.00177269 | -0.3554576 |
| FLAD1 | 1.13573084 | 3.99558095 | 9.20990958 | 1.50E-18 | 1.39E-16 | 31.277083 |
| ESPL1 | 1.13513495 | 1.93467232 | 6.38911377 | 4.38E-10 | 1.26E-08 | 12.1476321 |
| LSM4 | 1.13503809 | 5.29888103 | 9.30213018 | 7.31E-19 | 7.18E-17 | 31.9854414 |
| IGSF8 | 1.13468873 | 4.91036568 | 6.35739078 | 5.29E-10 | 1.49E-08 | 11.9635496 |
| RANBP1 | 1.13427611 | 4.43577999 | 7.68953811 | 1.03E-13 | 5.13E-12 | 20.3186109 |
| MIEN1 | 1.1340663 | 4.83446062 | 5.65193568 | 2.91E-08 | 6.10E-07 | 8.06931277 |
| CCNL2 | 1.13251146 | 4.5306724 | 5.43510356 | 9.24E-08 | 1.76E-06 | 6.95107917 |
| PFDN6 | 1.13194971 | 4.12843262 | 8.44170042 | 4.96E-16 | 3.42E-14 | 25.5645403 |
| MRPS26 | 1.13143863 | 5.94400178 | 7.95802999 | 1.60E-14 | 9.05E-13 | 22.1498452 |
| GPRIN1 | 1.12978274 | 1.49093505 | 5.51336202 | 6.12E-08 | 1.20E-06 | 7.35030397 |
| HES2 | 1.12905597 | 1.8577934 | 2.92789563 | 0.00359582 | 0.02204882 | -3.0615168 |
| E2F8 | 1.12520488 | 2.01167576 | 5.56870181 | 4.55E-08 | 9.20E-07 | 7.63559452 |
| PSME2 | 1.1250984 | 5.56335297 | 6.01038647 | 3.98E-09 | 9.81E-08 | 9.99982503 |
| RP1-86C11.7 | 1.12501289 | 1.43627675 | 4.90547181 | 1.33E-06 | 2.02E-05 | 4.38126841 |
| CHAC1 | 1.1245837 | 1.69770499 | 5.71378687 | 2.08E-08 | 4.48E-07 | 8.3951902 |
| PDXK | 1.12432894 | 4.03730542 | 7.0456907 | 7.47E-12 | 2.84E-10 | 16.1239361 |
| GALNT6 | 1.12263052 | 1.96955596 | 4.69873429 | 3.54E-06 | 4.89E-05 | 3.44182043 |
| SNX10 | 1.12251304 | 2.18228026 | 5.06107944 | 6.22E-07 | 1.01E-05 | 5.11215665 |
| TUBG1 | 1.12200468 | 4.77891129 | 8.06495263 | 7.52E-15 | 4.49E-13 | 22.8920653 |
| PVRL1 | 1.12136119 | 5.00664299 | 3.52880494 | 0.00046293 | 0.00382196 | -1.17264678 |

| | | | | | | |
|---|---|---|---|---|---|---|
| CHEK1 | 1.12040897 | 2.10982379 | 7.50291581 | 3.68E-13 | 1.68E-11 | 19.0737455 |
| P4HB | 1.11996949 | 7.72894837 | 8.82889069 | 2.78E-17 | 2.25E-15 | 28.4009372 |
| REEP4 | 1.11912378 | 4.85552423 | 6.47692131 | 2.59E-10 | 7.75E-09 | 12.6610919 |
| CLSTN3 | 1.11846732 | 3.64664611 | 6.15428916 | 1.74E-09 | 4.56E-08 | 10.8030146 |
| MAPK15 | 1.11809332 | 1.52079198 | 4.65024082 | 4.43E-06 | 6.01E-05 | 3.22672744 |
| UQCC3 | 1.11784982 | 3.65566719 | 7.21066725 | 2.56E-12 | 1.04E-10 | 17.171626 |
| MIR7111 | 1.11769922 | 1.55477712 | 6.07581238 | 2.74E-09 | 6.94E-08 | 10.3630207 |
| IRF6 | 1.11718732 | 5.21102553 | 3.42271116 | 0.00068002 | 0.00533377 | -1.53039702 |
| UNG | 1.11585094 | 4.67740522 | 8.24542438 | 2.07E-15 | 1.32E-13 | 24.1612451 |
| RGS19 | 1.11460555 | 3.47731181 | 7.25117579 | 1.96E-12 | 8.16E-11 | 17.4317782 |
| RFC2 | 1.11420357 | 4.29660868 | 8.07386503 | 7.06E-15 | 4.23E-13 | 22.9542607 |
| TOMM34 | 1.11351188 | 5.35855525 | 6.23549859 | 1.09E-09 | 2.93E-08 | 11.2632924 |
| AC016735.2 | 1.11350759 | 1.72724666 | 3.09895523 | 0.00207087 | 0.01383748 | -2.5579637 |
| C9orf142 | 1.11213854 | 4.89537071 | 6.556862 | 1.59E-10 | 4.98E-09 | 13.1335404 |
| TMEM97 | 1.11197722 | 4.78462089 | 2.81925871 | 0.00503821 | 0.02925611 | -3.36705756 |
| NCAPG2 | 1.11155278 | 2.4461044 | 6.25603328 | 9.63E-10 | 2.61E-08 | 11.3804742 |
| GPT2 | 1.11147532 | 2.87563371 | 5.17392469 | 3.54E-07 | 6.01E-06 | 5.65482873 |
| CENPE | 1.11089597 | 1.72621333 | 7.05933642 | 6.85E-12 | 2.62E-10 | 16.2098696 |
| RP1-34B20.4 | 1.11024288 | 1.22890451 | 4.56231542 | 6.63E-06 | 8.64E-05 | 2.84187864 |
| RP11-303E16 | 1.11017726 | 3.36663241 | 6.4065032 | 3.95E-10 | 1.14E-08 | 12.2488601 |
| SNRPF | 1.11003511 | 4.53859436 | 7.6070896 | 1.82E-13 | 8.76E-12 | 19.7657727 |
| EIF2AK1 | 1.10919787 | 5.14340321 | 10.366488 | 1.33E-22 | 2.01E-20 | 40.4836245 |
| FANCG | 1.10801014 | 3.09724848 | 7.12309015 | 4.53E-12 | 1.78E-10 | 16.6130918 |
| AC019129.2 | 1.10799351 | 1.62132652 | 3.55924377 | 0.00041383 | 0.0034666 | -1.06810491 |
| GMNN | 1.10796849 | 3.93408526 | 5.77595363 | 1.48E-08 | 3.28E-07 | 8.72579008 |
| IGFL1P1 | 1.10794159 | 1.30942975 | 3.29107072 | 0.0010812 | 0.00794066 | -1.95990975 |
| FANCA | 1.10755881 | 1.62428108 | 7.81065101 | 4.48E-14 | 2.36E-12 | 21.1388385 |
| RP11-109M1 | 1.10731006 | 1.47441802 | 2.9289168 | 0.00358426 | 0.02199876 | -3.05859211 |
| HMGB2 | 1.10637324 | 5.58541366 | 5.04549481 | 6.71E-07 | 1.09E-05 | 5.03804258 |
| TMEM184A | 1.1063724 | 2.94104836 | 3.7044698 | 0.00023975 | 0.00214734 | -0.55770967 |
| PARP12 | 1.10578554 | 3.26514278 | 5.79334081 | 1.34E-08 | 3.00E-07 | 8.8188012 |
| PTK6 | 1.10459756 | 4.26930204 | 2.98817409 | 0.00296897 | 0.01874859 | -2.88719922 |
| ARL6IP1 | 1.104156 | 6.21555155 | 6.50933315 | 2.13E-10 | 6.51E-09 | 12.8520742 |
| DARS2 | 1.10367582 | 3.28888892 | 6.8168615 | 3.20E-11 | 1.10E-09 | 14.7026046 |
| KIAA0907 | 1.10366524 | 3.26613907 | 6.94265103 | 1.44E-11 | 5.26E-10 | 15.479302 |
| GALNT14 | 1.10237363 | 2.20065223 | 3.66506046 | 0.00027853 | 0.00244732 | -0.69810813 |
| TARBP2 | 1.1014116 | 3.28913392 | 9.59009949 | 7.53E-20 | 8.30E-18 | 34.2270618 |
| EME1 | 1.10095321 | 1.39352252 | 7.79701257 | 4.93E-14 | 2.58E-12 | 21.0459929 |
| THEM6 | 1.10033408 | 4.62568489 | 3.85783996 | 0.00013211 | 0.00126387 | 0.00204662 |
| ZNF692 | 1.09936129 | 3.33150549 | 5.89598769 | 7.59E-09 | 1.77E-07 | 9.372749 |
| C1orf53 | 1.09928588 | 2.2083562 | 6.43723935 | 3.29E-10 | 9.65E-09 | 12.428336 |
| CASP2 | 1.09873131 | 2.97111555 | 9.37761185 | 4.05E-19 | 4.07E-17 | 32.5686902 |
| PRR36 | 1.0983413 | 1.50272851 | 3.70287191 | 0.00024122 | 0.00215924 | -0.56342965 |
| CHAF1A | 1.09731869 | 3.43457943 | 7.36875038 | 9.03E-13 | 3.93E-11 | 18.1932694 |
| DSCC1 | 1.09716871 | 2.16200764 | 6.32444811 | 6.44E-10 | 1.79E-08 | 11.7731907 |
| DUSP23 | 1.09646684 | 5.49422127 | 4.86178312 | 1.64E-06 | 2.45E-05 | 4.17972187 |
| HES4 | 1.09615902 | 3.88306873 | 3.64957783 | 0.00029532 | 0.002572 | -0.75288046 |
| CTD-2537I9. | 1.09538085 | 2.01268003 | 5.47606996 | 7.45E-08 | 1.44E-06 | 7.15944593 |
| ATAD3A | 1.09441289 | 3.90822023 | 7.8673716 | 3.02E-14 | 1.63E-12 | 21.5262718 |
| CENPK | 1.09387122 | 1.6123945 | 7.26233155 | 1.83E-12 | 7.64E-11 | 17.5036217 |
| TAGLN2 | 1.09332953 | 8.5470993 | 5.31497412 | 1.73E-07 | 3.12E-06 | 6.3479305 |
| BLOC1S3 | 1.09316538 | 3.19501699 | 8.63658351 | 1.18E-16 | 8.92E-15 | 26.9810425 |
| MIR621 | 1.09315003 | 3.4048716 | 5.23850601 | 2.55E-07 | 4.46E-06 | 5.97014281 |
| TOMM40 | 1.09253316 | 4.88378608 | 7.98544409 | 1.32E-14 | 7.53E-13 | 22.3394482 |
| ATP5J2 | 1.09194513 | 5.77942891 | 8.5720666 | 1.90E-16 | 1.41E-14 | 26.5095795 |
| FKBPL | 1.09084831 | 3.13990515 | 7.93028446 | 1.95E-14 | 1.09E-12 | 21.9584411 |
| SREBF1 | 1.0904303 | 4.3754413 | 4.78074981 | 2.41E-06 | 3.46E-05 | 3.81016987 |
| MRM1 | 1.09042204 | 2.97538045 | 7.54082345 | 2.85E-13 | 1.33E-11 | 19.324724 |

| | | | | | | |
|---|---|---|---|---|---|---|
| PPAP2C | 1.08934023 | 3.56101466 | 3.89097697 | 0.00011584 | 0.00112484 | 0.12576955 |
| ARHGAP40 | 1.08900932 | 2.04633036 | 3.5213289 | 0.00047579 | 0.00391744 | -1.19819365 |
| MYEOV | 1.08858173 | 1.83158753 | 2.68068655 | 0.00763255 | 0.04112223 | -3.7406267 |
| BRMS1 | 1.08800017 | 4.85043679 | 7.78847062 | 5.23E-14 | 2.72E-12 | 20.9879042 |
| ROMO1 | 1.08725472 | 6.81208241 | 6.59241078 | 1.28E-10 | 4.09E-09 | 13.3451518 |
| KNTC1 | 1.08669863 | 1.94524927 | 7.58811208 | 2.07E-13 | 9.83E-12 | 19.6391637 |
| PPP1CA | 1.08485922 | 6.45220601 | 8.58432116 | 1.73E-16 | 1.29E-14 | 26.5989398 |
| LPCAT1 | 1.08451759 | 4.09603813 | 4.45748048 | 1.06E-05 | 0.00013176 | 2.39172934 |
| NR2F6 | 1.08416885 | 5.05176533 | 5.90159074 | 7.36E-09 | 1.72E-07 | 9.40322445 |
| ERBB3 | 1.08349812 | 4.17743135 | 3.49630377 | 0.00052134 | 0.00423477 | -1.28333686 |
| LAMTOR2 | 1.08264518 | 5.35962334 | 7.33800879 | 1.11E-12 | 4.75E-11 | 17.9932499 |
| BAX | 1.08245451 | 5.0744508 | 8.51331563 | 2.93E-16 | 2.11E-14 | 26.0824186 |
| EXOSC4 | 1.08205296 | 4.49913558 | 6.71557621 | 6.01E-11 | 2.00E-09 | 14.0854979 |
| SPTBN2 | 1.08160815 | 2.53131511 | 4.68328461 | 3.80E-06 | 5.23E-05 | 3.37307492 |
| TMC4 | 1.08151887 | 4.66366887 | 3.03713002 | 0.00253534 | 0.01645518 | -2.74311948 |
| SP6 | 1.08018945 | 2.89379184 | 3.16246489 | 0.00167641 | 0.01154252 | -2.36405228 |
| PDRG1 | 1.07944796 | 4.00664996 | 7.43128464 | 5.95E-13 | 2.66E-11 | 18.6021377 |
| NR2C2AP | 1.07880401 | 4.21173767 | 8.10554572 | 5.63E-15 | 3.42E-13 | 23.1757526 |
| MYH14 | 1.07856598 | 4.27730239 | 2.89051526 | 0.00404296 | 0.02433572 | -3.16790225 |
| PERP | 1.07837721 | 7.05728061 | 3.47685576 | 0.00055952 | 0.00450044 | -1.3491091 |
| COASY | 1.07824304 | 4.67192651 | 9.69347166 | 3.30E-20 | 3.81E-18 | 35.042477 |
| ABHD12 | 1.07735829 | 5.24564346 | 5.48239425 | 7.21E-08 | 1.40E-06 | 7.19173407 |
| CTD-2021H9 | 1.0765001 | 1.5607768 | 3.02223855 | 0.00266066 | 0.01714278 | -2.78718316 |
| KDELR3 | 1.07634302 | 3.43651251 | 3.87999062 | 0.00012101 | 0.00116825 | 0.08464083 |
| LYPD6B | 1.07617954 | 3.03319966 | 3.46709611 | 0.00057965 | 0.00464443 | -1.38198521 |
| PSMB3 | 1.07604486 | 7.04120011 | 7.63235865 | 1.53E-13 | 7.46E-12 | 19.9347283 |
| RPS6KA1 | 1.07590829 | 3.45174731 | 6.50230652 | 2.22E-10 | 6.76E-09 | 12.8106045 |
| C10orf35 | 1.0754861 | 3.17124404 | 5.46058529 | 8.08E-08 | 1.55E-06 | 7.08052677 |
| CCNO | 1.07214577 | 2.43249793 | 4.47251543 | 9.93E-06 | 0.00012411 | 2.45570345 |
| KIAA1524 | 1.07116931 | 1.91674684 | 6.62359569 | 1.06E-10 | 3.41E-09 | 13.5315528 |
| PYCARD | 1.07089365 | 4.32242899 | 4.35365484 | 1.68E-05 | 0.00019889 | 1.95531246 |
| TFAP2A | 1.06973765 | 2.38167561 | 3.99619599 | 7.59E-05 | 0.00077079 | 0.52514314 |
| HOXB6 | 1.06954476 | 1.98073807 | 3.58883145 | 0.00037082 | 0.00314943 | -0.96567594 |
| INCENP | 1.06925659 | 2.88990115 | 6.09077298 | 2.52E-09 | 6.42E-08 | 10.4465348 |
| RSRP1 | 1.06888602 | 3.08911719 | 5.82126889 | 1.15E-08 | 2.60E-07 | 8.96869883 |
| TFAP2C | 1.06793505 | 3.10657251 | 4.37317851 | 1.54E-05 | 0.00018447 | 2.03666071 |
| MAFG-AS1 | 1.06784345 | 1.79349229 | 5.57574077 | 4.39E-08 | 8.88E-07 | 7.67205859 |
| SLC35F2 | 1.06767166 | 2.72564074 | 5.38090167 | 1.23E-07 | 2.28E-06 | 6.67748556 |
| PPP1R16A | 1.06750415 | 2.72667992 | 6.8426874 | 2.72E-11 | 9.51E-10 | 14.861142 |
| MYO19 | 1.06684003 | 2.34367761 | 8.3074532 | 1.32E-15 | 8.58E-14 | 24.6021634 |
| GAPDHP1 | 1.0649602 | 2.4846257 | 2.77415827 | 0.00577805 | 0.03272671 | -3.49063458 |
| RP11-465B22 | 1.06384171 | 2.54442561 | 3.25961275 | 0.00120523 | 0.00870592 | -2.06018212 |
| ATP13A2 | 1.0634931 | 3.86614613 | 8.07977555 | 6.77E-15 | 4.06E-13 | 22.9955352 |
| DDX11 | 1.06346433 | 2.38074971 | 7.61030293 | 1.78E-13 | 8.59E-12 | 19.7872342 |
| HENMT1 | 1.06325933 | 3.04542219 | 5.84272603 | 1.02E-08 | 2.33E-07 | 9.08428216 |
| CKLF | 1.06305014 | 3.33202136 | 7.26454341 | 1.80E-12 | 7.55E-11 | 17.5178763 |
| TNFAIP2 | 1.0628005 | 6.23561668 | 2.99958232 | 0.00286225 | 0.01820669 | -2.85382471 |
| GALNT3 | 1.06084724 | 3.21992638 | 4.27054529 | 2.41E-05 | 0.00027454 | 1.61275344 |
| LSM7 | 1.05915245 | 5.13646447 | 6.8761768 | 2.20E-11 | 7.83E-10 | 15.067438 |
| CASC9 | 1.05873347 | 2.31378488 | 3.58333174 | 0.00037848 | 0.00320306 | -0.98477566 |
| TNFRSF25 | 1.0585759 | 2.49214047 | 5.91646923 | 6.77E-09 | 1.59E-07 | 9.4842689 |
| DTX4 | 1.05826808 | 3.70681405 | 3.64144297 | 0.00030451 | 0.00264225 | -0.78157165 |
| TCF3 | 1.0580517 | 4.22971034 | 7.9884229 | 1.29E-14 | 7.38E-13 | 22.3600795 |
| ATP6V0B | 1.05787287 | 5.40833501 | 8.26079466 | 1.85E-15 | 1.19E-13 | 24.2702791 |
| SLC37A4 | 1.05749593 | 2.58618391 | 7.8978605 | 2.44E-14 | 1.34E-12 | 21.7353908 |
| KCNN4 | 1.05704174 | 4.18620001 | 2.88990363 | 0.00405068 | 0.02436781 | -3.16963205 |
| HIST1H4E | 1.05613939 | 1.28587243 | 3.8482818 | 0.00013719 | 0.00130733 | -0.03345721 |
| SOX9 | 1.05562797 | 2.79081951 | 2.86910653 | 0.00432132 | 0.02570386 | -3.22824018 |

| | | | | | | |
|---|---|---|---|---|---|---|
| HOXB5 | 1.05513075 | 1.79814584 | 3.68929281 | 0.00025404 | 0.00225974 | -0.61194547 |
| RP11-54H7.4 | 1.05487872 | 1.08227035 | 2.87924701 | 0.00418737 | 0.02505255 | -3.19971421 |
| ZNF488 | 1.05448236 | 1.521706 | 3.78070922 | 0.00017873 | 0.00165337 | -0.28211074 |
| RP11-12G12. | 1.05380395 | 3.23355153 | 5.75920321 | 1.62E-08 | 3.56E-07 | 8.63641106 |
| COA6 | 1.05336934 | 4.33440785 | 8.05524106 | 8.06E-15 | 4.79E-13 | 22.8243502 |
| CTD-2583A1 | 1.05298217 | 2.3629756 | 6.35398159 | 5.40E-10 | 1.52E-08 | 11.9438118 |
| RHBG | 1.05258364 | 1.46329054 | 3.15377765 | 0.00172593 | 0.01182986 | -2.39079831 |
| SLC2A6 | 1.05223393 | 2.52359111 | 3.9414936 | 9.46E-05 | 0.00094075 | 0.31627682 |
| RP11-764K9. | 1.05203141 | 2.51596313 | 4.77195158 | 2.51E-06 | 3.59E-05 | 3.77038087 |
| MRGBP | 1.05201531 | 3.58521843 | 8.78941446 | 3.74E-17 | 2.97E-15 | 28.1076984 |
| FBXO6 | 1.05185428 | 3.61548891 | 6.19975985 | 1.34E-09 | 3.56E-08 | 11.0601131 |
| NT5DC2 | 1.05178327 | 4.37868185 | 5.01980752 | 7.62E-07 | 1.22E-05 | 4.91632684 |
| AUP1 | 1.05125601 | 6.20297202 | 9.38534736 | 3.81E-19 | 3.85E-17 | 32.6286376 |
| HIP1R | 1.05021312 | 3.59634799 | 5.96242885 | 5.23E-09 | 1.26E-07 | 9.73570574 |
| BRI3BP | 1.04984572 | 2.41874161 | 7.06493184 | 6.60E-12 | 2.53E-10 | 16.2451444 |
| CCDC58 | 1.04980719 | 3.57100581 | 8.87148522 | 2.01E-17 | 1.65E-15 | 28.7183538 |
| SNHG3 | 1.04976531 | 3.20434585 | 6.25451993 | 9.72E-10 | 2.63E-08 | 11.3718272 |
| PCGF2 | 1.04973005 | 4.11338527 | 6.4399265 | 3.23E-10 | 9.51E-09 | 12.4440605 |
| GPR87 | 1.04933352 | 3.9816615 | 2.9169163 | 0.00372223 | 0.02270474 | -3.09290046 |
| CTD-2538C1 | 1.04895961 | 2.40150035 | 3.67428153 | 0.00026896 | 0.00237432 | -0.66538366 |
| CLN6 | 1.04672885 | 3.80400593 | 7.76118979 | 6.31E-14 | 3.23E-12 | 20.8027033 |
| TMEM139 | 1.04635667 | 1.78751228 | 3.04568837 | 0.00246579 | 0.01606988 | -2.71770159 |
| CBX3 | 1.04581103 | 5.53239416 | 9.14337596 | 2.51E-18 | 2.24E-16 | 30.7689494 |
| ITPA | 1.04489668 | 5.08224302 | 8.13765742 | 4.48E-15 | 2.75E-13 | 23.4009047 |
| RPN1 | 1.04423597 | 6.66393575 | 9.31319921 | 6.71E-19 | 6.59E-17 | 32.0707778 |
| PPP4C | 1.04413003 | 5.88676671 | 8.12932431 | 4.75E-15 | 2.90E-13 | 23.3424144 |
| MND1 | 1.04310527 | 1.97262645 | 6.31119769 | 6.96E-10 | 1.93E-08 | 11.696854 |
| UNC93B1 | 1.04295401 | 4.41882292 | 5.04321763 | 6.79E-07 | 1.10E-05 | 5.02723022 |
| MGAT4B | 1.04271648 | 4.57197815 | 7.53527723 | 2.96E-13 | 1.37E-11 | 19.2879433 |
| MANEAL | 1.04264497 | 2.48830555 | 4.03991007 | 6.34E-05 | 0.00065657 | 0.69397207 |
| ADCK5 | 1.04251166 | 2.93904111 | 6.80836352 | 3.37E-11 | 1.16E-09 | 14.6505431 |
| PDIA3 | 1.04203998 | 7.06772783 | 8.44362114 | 4.89E-16 | 3.38E-14 | 25.5783892 |
| XRCC2 | 1.04181288 | 1.36217237 | 7.88793585 | 2.62E-14 | 1.42E-12 | 21.667253 |
| OLR1 | 1.04103402 | 1.97857051 | 2.67618897 | 0.00773399 | 0.04156519 | -3.75244685 |
| PPAPDC1A | 1.04100086 | 1.15570608 | 3.73133704 | 0.0002163 | 0.0019592 | -0.46118663 |
| MFSD10 | 1.04031218 | 5.33137413 | 6.44242708 | 3.19E-10 | 9.38E-09 | 12.4586981 |
| TMEM223 | 1.04021457 | 3.42633284 | 8.50436365 | 3.13E-16 | 2.24E-14 | 26.0175136 |
| SMYD2 | 1.03995274 | 3.38819614 | 7.61630259 | 1.71E-13 | 8.28E-12 | 19.8273239 |
| CNPY2 | 1.03993648 | 3.60555426 | 7.90247965 | 2.36E-14 | 1.30E-12 | 21.7671253 |
| CTA-384D8.3 | 1.03988196 | 1.50953746 | 3.43549879 | 0.00064956 | 0.00512648 | -1.48782392 |
| RFC5 | 1.03907271 | 3.09556028 | 7.61027992 | 1.78E-13 | 8.59E-12 | 19.7870805 |
| CH17-360D5. | 1.03857575 | 2.06719698 | 4.36537255 | 1.60E-05 | 0.00018995 | 2.00409615 |
| RIPPLY3 | 1.03817369 | 1.56928513 | 4.25245202 | 2.60E-05 | 0.00029435 | 1.53897948 |
| HCAR1 | 1.03772667 | 1.84152725 | 3.7281158 | 0.00021899 | 0.00198053 | -0.4727937 |
| RP3-406A7.7 | 1.03761542 | 2.78444415 | 3.88563822 | 0.00011833 | 0.00114536 | 0.10576978 |
| SMPDL3B | 1.03741361 | 1.83497647 | 3.90446576 | 0.00010978 | 0.00107347 | 0.17641448 |
| EPS8L1 | 1.03585473 | 3.35589548 | 3.25655148 | 0.00121798 | 0.00878606 | -2.06989105 |
| MAD2L2 | 1.03581398 | 3.5958427 | 5.64454334 | 3.03E-08 | 6.31E-07 | 8.03056833 |
| NAA40 | 1.03481928 | 2.82517486 | 8.36041573 | 8.98E-16 | 5.96E-14 | 24.9805162 |
| KIF14 | 1.03397699 | 1.40910211 | 6.60764359 | 1.17E-10 | 3.73E-09 | 13.4361133 |
| ARRDC1 | 1.03307427 | 4.17652988 | 6.06572093 | 2.90E-09 | 7.32E-08 | 10.306785 |
| MREG | 1.03278776 | 2.81123849 | 5.6439716 | 3.04E-08 | 6.33E-07 | 8.02757356 |
| IRF7 | 1.03273146 | 3.67124389 | 3.7800717 | 0.00017917 | 0.0016563 | -0.28443708 |
| DDR1 | 1.03219372 | 5.17409728 | 4.59023625 | 5.84E-06 | 7.70E-05 | 2.96336763 |
| CTA-384D8.3 | 1.03192664 | 2.87882108 | 5.10450592 | 5.01E-07 | 8.31E-06 | 5.3197425 |
| SLC25A10 | 1.03145493 | 2.55481394 | 5.3432567 | 1.49E-07 | 2.73E-06 | 6.48887327 |
| ETV7 | 1.03111107 | 2.63273323 | 4.05935863 | 5.86E-05 | 0.00061129 | 0.76963127 |
| ZMYND19 | 1.03108485 | 3.74193641 | 7.60476619 | 1.85E-13 | 8.88E-12 | 19.750259 |

| | | | | | | |
|---|---|---|---|---|---|---|
| DNAJB11 | 1.03094781 | 4.06947336 | 8.76794489 | 4.40E-17 | 3.44E-15 | 27.9485986 |
| PTK7 | 1.03047103 | 3.78922367 | 4.57563273 | 6.24E-06 | 8.19E-05 | 2.89974112 |
| IFIT3 | 1.03033805 | 3.58161033 | 2.74815193 | 0.00624807 | 0.03495316 | -3.56101944 |
| WDR4 | 1.0299953 | 3.07251574 | 7.91494763 | 2.17E-14 | 1.20E-12 | 21.8528518 |
| NDC1 | 1.02971148 | 3.30542727 | 7.51258396 | 3.45E-13 | 1.58E-11 | 19.1376645 |
| AGTRAP | 1.02726389 | 4.57729415 | 5.74222088 | 1.78E-08 | 3.89E-07 | 8.54602116 |
| PGP | 1.02722522 | 3.35159819 | 7.51832444 | 3.32E-13 | 1.52E-11 | 19.1756462 |
| PNKP | 1.02690315 | 3.70267777 | 7.79401249 | 5.03E-14 | 2.63E-12 | 21.0255857 |
| MRPL14 | 1.02678245 | 5.99229516 | 7.07553904 | 6.17E-12 | 2.37E-10 | 16.3120753 |
| CYP4F23P | 1.02675866 | 1.84487553 | 2.69924942 | 0.00722643 | 0.03931848 | -3.69163839 |
| TRAF2 | 1.02668348 | 3.33849063 | 7.2583567 | 1.87E-12 | 7.82E-11 | 17.4780136 |
| SMUG1 | 1.02649883 | 3.12982144 | 9.13930932 | 2.59E-18 | 2.31E-16 | 30.7379714 |
| MICB | 1.02641136 | 1.8158224 | 4.52977795 | 7.68E-06 | 9.85E-05 | 2.70114966 |
| DDOST | 1.02571587 | 6.84337121 | 8.26759672 | 1.76E-15 | 1.13E-13 | 24.3185785 |
| RAD9A | 1.02548155 | 2.92850876 | 6.46169689 | 2.84E-10 | 8.43E-09 | 12.5716538 |
| VPS9D1-AS1 | 1.02372479 | 1.98217346 | 4.27955662 | 2.32E-05 | 0.00026544 | 1.64960368 |
| CDC42BPG | 1.02359664 | 3.36336522 | 4.67181355 | 4.01E-06 | 5.49E-05 | 3.32216487 |
| CBX8 | 1.02351245 | 1.95149723 | 7.71218311 | 8.85E-14 | 4.42E-12 | 20.4712394 |
| CASP6 | 1.02332253 | 3.75203848 | 7.00129749 | 9.94E-12 | 3.71E-10 | 15.8452816 |
| HSBP1L1 | 1.02189304 | 3.05492954 | 5.82788244 | 1.11E-08 | 2.52E-07 | 9.00428547 |
| CCT5 | 1.02144335 | 5.46376535 | 5.82799138 | 1.11E-08 | 2.51E-07 | 9.00487195 |
| IGF2BP2 | 1.02103991 | 1.99714575 | 2.78547671 | 0.00558367 | 0.03181995 | -3.45980215 |
| FCHO1 | 1.01932887 | 1.81746514 | 4.84226111 | 1.80E-06 | 2.66E-05 | 4.09018324 |
| CSTB | 1.01915238 | 7.62181148 | 3.83827718 | 0.0001427 | 0.00135413 | -0.07053142 |
| CA2 | 1.01909812 | 2.60223226 | 2.69068504 | 0.00741132 | 0.0401899 | -3.71428077 |
| FAM96A | 1.01909421 | 3.90926518 | 9.43382298 | 2.60E-19 | 2.70E-17 | 33.0050416 |
| SIRT7 | 1.01894549 | 3.04017884 | 7.68784686 | 1.05E-13 | 5.19E-12 | 20.3072254 |
| NAT14 | 1.01873457 | 3.30652139 | 4.98831486 | 8.89E-07 | 1.40E-05 | 4.76785598 |
| CDH24 | 1.01856633 | 2.26556673 | 5.51275007 | 6.14E-08 | 1.21E-06 | 7.34716298 |
| RP11-670E13 | 1.01853394 | 2.35550999 | 4.36526421 | 1.60E-05 | 0.00018996 | 2.00364455 |
| CXADR | 1.01739567 | 3.80256436 | 3.28519186 | 0.00110344 | 0.00808061 | -1.97871819 |
| GSS | 1.01722792 | 5.5201186 | 7.94310555 | 1.78E-14 | 1.00E-12 | 22.0468266 |
| CYBA | 1.01686498 | 5.29935014 | 3.99556338 | 7.61E-05 | 0.00077264 | 0.52271241 |
| AP1G2 | 1.01650466 | 3.36621625 | 5.37575307 | 1.26E-07 | 2.34E-06 | 6.65162133 |
| TSEN54 | 1.01626458 | 3.9373763 | 7.95544879 | 1.63E-14 | 9.19E-13 | 22.1320178 |
| SLC25A22 | 1.01526685 | 3.15210944 | 7.20940034 | 2.58E-12 | 1.05E-10 | 17.163508 |
| CAMSAP3 | 1.01418887 | 3.05238214 | 4.28552255 | 2.26E-05 | 0.00025917 | 1.67403947 |
| ECE2 | 1.0134034 | 1.69011205 | 7.86679045 | 3.03E-14 | 1.63E-12 | 21.5222917 |
| RPA3 | 1.01276805 | 3.23578853 | 6.91490793 | 1.72E-11 | 6.21E-10 | 15.307026 |
| FBXW9 | 1.01269471 | 2.98556167 | 8.34222833 | 1.03E-15 | 6.75E-14 | 24.8503949 |
| PLEKHJ1 | 1.01204065 | 3.90209533 | 7.04260684 | 7.62E-12 | 2.89E-10 | 16.1045339 |
| ENTPD6 | 1.01139862 | 4.1007204 | 7.54919108 | 2.69E-13 | 1.26E-11 | 19.3802544 |
| MARVELD3 | 1.01130857 | 2.00855273 | 5.91370655 | 6.87E-09 | 1.62E-07 | 9.46920725 |
| TPM3 | 1.01122849 | 5.5115274 | 7.36112289 | 9.50E-13 | 4.13E-11 | 18.143581 |
| AP2S1 | 1.01053578 | 5.94055919 | 6.91200628 | 1.76E-11 | 6.32E-10 | 15.2890394 |
| PIF1 | 1.00977225 | 1.53086568 | 7.00238049 | 9.87E-12 | 3.69E-10 | 15.8520629 |
| RUVBL1 | 1.0077824 | 3.50538333 | 7.42972371 | 6.01E-13 | 2.68E-11 | 18.5918995 |
| EDEM2 | 1.00749244 | 4.53317972 | 7.99378093 | 1.24E-14 | 7.13E-13 | 22.3972034 |
| TUBB | 1.00585542 | 8.12859995 | 6.59542158 | 1.26E-10 | 4.01E-09 | 13.363117 |
| AATBC | 1.00535874 | 1.88028869 | 3.08994562 | 0.00213329 | 0.01418312 | -2.58516801 |
| ACYP1 | 1.00473703 | 2.5938072 | 7.22273389 | 2.37E-12 | 9.69E-11 | 17.2490009 |
| MYDGF | 1.00465656 | 5.91853868 | 7.37789102 | 8.50E-13 | 3.71E-11 | 18.2528674 |
| MFI2-AS1 | 1.00430468 | 1.8263659 | 5.43279449 | 9.35E-08 | 1.78E-06 | 6.93937504 |
| MIR210HG | 1.00377997 | 2.20940622 | 4.34281275 | 1.76E-05 | 0.00020767 | 1.91028103 |
| LINC01518 | 1.00320743 | 1.08622867 | 3.12633924 | 0.00189133 | 0.01278265 | -2.47481385 |
| ERBB2 | 1.00259603 | 5.09141748 | 3.17648454 | 0.00159925 | 0.01108448 | -2.32074095 |
| UCK2 | 1.00229453 | 2.99580327 | 6.40492899 | 3.99E-10 | 1.15E-08 | 12.2396869 |
| METTL7B | 1.00217713 | 1.48277687 | 3.48795226 | 0.00053743 | 0.00434609 | -1.31162369 |

| | | | | | | |
|---|---|---|---|---|---|---|
| POLE2 | 1.00209833 | 1.6885146 | 7.22155917 | 2.39E-12 | 9.75E-11 | 17.2414638 |
| POLR2G | 1.00201718 | 5.18687418 | 8.51009564 | 3.00E-16 | 2.16E-14 | 26.059067 |
| ENO1 | 1.00161163 | 8.53271051 | 5.73674078 | 1.83E-08 | 3.99E-07 | 8.51690155 |
| KIF18A | 1.0002719 | 1.42685059 | 6.53606342 | 1.81E-10 | 5.61E-09 | 13.0101652 |
| DBI | 1.00018061 | 5.84351232 | 5.3979574 | 1.12E-07 | 2.11E-06 | 6.76331983 |
| NAT9 | 1.0001367 | 3.44856134 | 8.51407699 | 2.91E-16 | 2.10E-14 | 26.0879409 |

# Supplementary Table S3

| | logFC | AveExpr | t | P.Value | adj.P.Val | B |
|---|---|---|---|---|---|---|
| KITLG | -1.000106989 | 1.866386203 | -3.970794439 | 8.41E-05 | 0.000845941 | 0.427821835 |
| MYH3 | -1.000778882 | 0.654330481 | -6.253693503 | 9.77E-10 | 2.64E-08 | 11.36710601 |
| COLCA1 | -1.002344931 | 1.207337165 | -3.43507543 | 0.000650545 | 0.005133581 | -1.489235796 |
| SH3D19 | -1.003536628 | 2.659421452 | -6.744064063 | 5.04E-11 | 1.69E-09 | 14.25831556 |
| PTGER4 | -1.003692238 | 2.139118564 | -4.071754555 | 5.56E-05 | 0.00058467 | 0.818029568 |
| PRSS23 | -1.003717409 | 3.120703858 | -4.002265259 | 7.40E-05 | 0.000754081 | 0.548481598 |
| GPM6B | -1.003884406 | 0.86989705 | -7.597287256 | 1.94E-13 | 9.28E-12 | 19.70034604 |
| MPEG1 | -1.003891424 | 1.958956584 | -3.640738762 | 0.000305324 | 0.002647301 | -0.784052507 |
| EPB41L2 | -1.003954675 | 1.862882345 | -5.529117721 | 5.62E-08 | 1.12E-06 | 7.43127759 |
| INPP5A | -1.004648596 | 3.10199096 | -6.961096622 | 1.28E-11 | 4.73E-10 | 15.59414695 |
| MEG3 | -1.006374094 | 0.888234072 | -5.207397177 | 2.99E-07 | 5.14E-06 | 5.817826463 |
| CYS1 | -1.008979474 | 0.758603188 | -6.500607344 | 2.24E-10 | 6.83E-09 | 12.80058181 |
| PNRC1 | -1.009215285 | 4.512708946 | -5.946565285 | 5.71E-09 | 1.37E-07 | 9.648732994 |
| FSTL1 | -1.009281788 | 4.543430295 | -3.638132894 | 0.000308334 | 0.002670626 | -0.793228861 |
| ZFP36L1 | -1.010867072 | 6.171919074 | -5.286072892 | 2.00E-07 | 3.58E-06 | 6.204581107 |
| PYGB | -1.011496188 | 5.141796079 | -5.018524544 | 7.67E-07 | 1.23E-05 | 4.910262102 |
| MAP3K8 | -1.012310996 | 2.645810549 | -5.433021828 | 9.34E-08 | 1.77E-06 | 6.940527186 |
| GABARAPL1 | -1.012865752 | 3.68272068 | -5.591610021 | 4.03E-08 | 8.20E-07 | 7.754412281 |
| JDP2 | -1.013035006 | 2.087119333 | -5.444762924 | 8.78E-08 | 1.67E-06 | 7.000086954 |
| RP11-514D23.3 | -1.013322836 | 0.437620814 | -10.42054904 | 8.47E-23 | 1.30E-20 | 40.93026914 |
| INHBB | -1.016949362 | 1.707701062 | -4.536211366 | 7.46E-06 | 9.59E-05 | 2.728902596 |
| MEF2D | -1.018211752 | 3.236746255 | -9.462900006 | 2.07E-19 | 2.17E-17 | 33.23142601 |
| TSPAN11 | -1.019344967 | 0.820341512 | -6.673848034 | 7.79E-11 | 2.55E-09 | 13.83342689 |
| MITF | -1.019400447 | 0.920008314 | -7.44426329 | 5.46E-13 | 2.44E-11 | 18.68732935 |
| AR | -1.02087991 | 0.966052001 | -4.572260848 | 6.33E-06 | 8.30E-05 | 2.885076155 |
| STK40 | -1.021666591 | 3.233252063 | -7.547580327 | 2.72E-13 | 1.27E-11 | 19.36956121 |
| ARRDC4 | -1.021745488 | 2.297380656 | -5.063743927 | 6.13E-07 | 1.00E-05 | 5.124848148 |
| STEAP4 | -1.022011578 | 1.215583334 | -4.285983826 | 2.25E-05 | 0.000258702 | 1.675930095 |
| ROR1 | -1.022364142 | 0.457448928 | -9.18398942 | 1.83E-18 | 1.68E-16 | 31.07883189 |
| GHR | -1.023558225 | 0.408398198 | -9.2689309 | 9.48E-19 | 9.15E-17 | 31.72989464 |
| TBL1X | -1.026047231 | 2.461365301 | -5.715305791 | 2.06E-08 | 4.45E-07 | 8.403231238 |
| SCN4B | -1.026698407 | 0.567120018 | -9.072213571 | 4.35E-18 | 3.76E-16 | 30.2281998 |
| TLE2 | -1.02718075 | 3.373143056 | -2.947108594 | 0.003383995 | 0.020956867 | -3.006325487 |
| LEPR | -1.028155294 | 0.709451886 | -9.821244652 | 1.18E-20 | 1.44E-18 | 36.05803339 |
| PLEKHO2 | -1.029249549 | 3.192093548 | -5.648467413 | 2.97E-08 | 6.19E-07 | 8.051129587 |
| MICU3 | -1.029298172 | 0.308999824 | -14.16633446 | 1.35E-37 | 9.87E-35 | 74.64857636 |
| KLRB1 | -1.029550757 | 1.005300042 | -5.729036909 | 1.91E-08 | 4.15E-07 | 8.476005799 |
| NFKBIZ | -1.029934182 | 2.556970997 | -4.634088945 | 4.77E-06 | 6.42E-05 | 3.155532874 |
| RBP1 | -1.030640273 | 2.263530207 | -3.053095308 | 0.002407017 | 0.015746613 | -2.695648096 |
| PTPLA | -1.031136875 | 1.082162435 | -5.570107408 | 4.52E-08 | 9.14E-07 | 7.642872793 |
| UGCG | -1.031352636 | 3.877394525 | -5.921981305 | 6.56E-09 | 1.55E-07 | 9.514337547 |
| AHNAK2 | -1.031770498 | 1.579346892 | -3.078916256 | 0.002212051 | 0.014638446 | -2.618367806 |
| NCOA7 | -1.03296022 | 2.738293857 | -4.773831482 | 2.49E-06 | 3.56E-05 | 3.778876989 |
| LINC00641 | -1.03345129 | 0.925619106 | -9.784376774 | 1.59E-20 | 1.89E-18 | 35.76414058 |
| GNA14 | -1.033701026 | 1.247850305 | -4.5071195 | 8.50E-06 | 0.000108064 | 2.603688809 |
| FKBP5 | -1.033896906 | 2.236075219 | -3.932757564 | 9.80E-05 | 0.000970068 | 0.283168253 |
| ALDH1L1 | -1.035845375 | 1.631542424 | -3.222911093 | 0.001366558 | 0.009693749 | -2.176010736 |
| RP11-798K23.5 | -1.036659528 | 1.141135033 | -6.002691601 | 4.16E-09 | 1.02E-07 | 9.957326487 |
| WNT9A | -1.037716522 | 1.368751199 | -5.253839731 | 2.36E-07 | 4.16E-06 | 6.045513551 |
| NEURL1B | -1.039492354 | 2.28063979 | -4.826095623 | 1.94E-06 | 2.84E-05 | 4.016283578 |
| C1QTNF2 | -1.041549225 | 0.621663469 | -9.390760744 | 3.65E-19 | 3.69E-17 | 32.67060854 |
| RP11-102K13.5 | -1.041654254 | 0.202285499 | -8.85082181 | 2.36E-17 | 1.91E-15 | 28.56423789 |
| LRIG1 | -1.041735981 | 1.652083185 | -4.041546303 | 6.30E-05 | 0.000652703 | 0.700324411 |
| DYNC1I1 | -1.042091872 | 1.162557973 | -5.255528246 | 2.34E-07 | 4.13E-06 | 6.053825028 |
| TMEM220 | -1.04227284 | 0.610091048 | -10.96603094 | 8.21E-25 | 1.60E-22 | 45.51189707 |
| FAM46C | -1.04462024 | 1.594688598 | -5.269270467 | 2.18E-07 | 3.88E-06 | 6.121556303 |
| TUB | -1.045827415 | 0.732387638 | -7.430512187 | 5.98E-13 | 2.67E-11 | 18.59707093 |
| FYN | -1.046804434 | 2.126755351 | -5.152356297 | 3.94E-07 | 6.66E-06 | 5.550289914 |
| PBXIP1 | -1.047040794 | 5.339444052 | -5.989886085 | 4.47E-09 | 1.09E-07 | 9.886703787 |
| CPEB4 | -1.047371296 | 1.667437944 | -7.589030221 | 2.05E-13 | 9.78E-12 | 19.6452835 |
| CDKN1C | -1.047548054 | 2.874535591 | -3.597718449 | 0.000358741 | 0.003061807 | -0.934754394 |
| AC002398.12 | -1.047736702 | 0.185487225 | -12.80214004 | 5.71E-32 | 2.45E-29 | 61.81886289 |
| TTLL7 | -1.048163184 | 0.766215441 | -7.681074799 | 1.10E-13 | 5.43E-12 | 20.26165466 |
| MPP7 | -1.048712988 | 2.155048112 | -4.942582339 | 1.11E-06 | 1.72E-05 | 4.553731831 |
| NPR2 | -1.05036083 | 1.333788298 | -7.748592086 | 6.89E-14 | 3.50E-12 | 20.71734613 |
| MEIS2 | -1.050499239 | 1.31874562 | -7.205676542 | 2.65E-12 | 1.07E-10 | 17.13965361 |
| BCL2 | -1.050834934 | 1.080142868 | -7.361154135 | 9.50E-13 | 4.13E-11 | 18.14378447 |
| TMEM246 | -1.052116909 | 1.314821398 | -4.710229459 | 3.36E-06 | 4.67E-05 | 3.493102125 |
| LDB3 | -1.053235558 | 0.34339145 | -10.53692998 | 3.18E-23 | 5.14E-21 | 41.89643235 |

| | | | | | | |
|---|---|---|---|---|---|---|
| AL031587.1 | -1.053546606 | 3.150395773 | -4.468465693 | 1.01E-05 | 0.000126105 | 2.43845241 |
| PITX2 | -1.054500734 | 1.000565718 | -5.80832504 | 1.24E-08 | 2.78E-07 | 8.899149246 |
| TXNIP | -1.054714227 | 6.708401372 | -3.762732419 | 0.00019163 | 0.001760063 | -0.347568584 |
| PROS1 | -1.055817584 | 1.903089704 | -5.031255631 | 7.20E-07 | 1.16E-05 | 4.970504091 |
| NFIC | -1.055935621 | 3.177719314 | -6.578243346 | 1.40E-10 | 4.43E-09 | 13.26070554 |
| LINC01133 | -1.059080755 | 1.447475987 | -2.971904172 | 0.003127486 | 0.019607484 | -2.9345855 |
| FAM110D | -1.059322168 | 0.961746204 | -7.858359213 | 3.22E-14 | 1.72E-12 | 21.4645725 |
| LRRC4B | -1.060258494 | 0.557430309 | -7.771551479 | 5.88E-14 | 3.03E-12 | 20.8729881 |
| REEP2 | -1.06030927 | 0.965601688 | -5.525355303 | 5.74E-08 | 1.13E-06 | 7.411923126 |
| ZFPM2 | -1.060753383 | 0.53581358 | -9.365827352 | 4.44E-19 | 4.43E-17 | 32.47742702 |
| CHRM3 | -1.06096854 | 0.461813318 | -8.370427031 | 8.35E-16 | 5.56E-14 | 25.05222855 |
| PRKAG2 | -1.062373145 | 1.901202987 | -8.132635005 | 4.64E-15 | 2.84E-13 | 23.365647 |
| NT5DC3 | -1.062566916 | 1.177517923 | -7.34583951 | 1.05E-12 | 4.52E-11 | 18.04413894 |
| CXCR4 | -1.063334403 | 3.790108691 | -3.513219473 | 0.000490128 | 0.004016439 | -1.225847167 |
| FAM180A | -1.063976861 | 0.37189411 | -9.869397342 | 7.97E-21 | 9.99E-19 | 36.442928 |
| AC114498.1 | -1.064446505 | 2.12535057 | -3.487016866 | 0.000539257 | 0.004358475 | -1.314787932 |
| NRP2 | -1.065031677 | 1.705995481 | -4.436225628 | 1.17E-05 | 0.000143236 | 2.30162411 |
| LAMA4 | -1.065446144 | 2.459226898 | -4.546631904 | 7.12E-06 | 9.20E-05 | 2.773931272 |
| DAAM2 | -1.065617491 | 0.897307938 | -7.251697709 | 1.96E-12 | 8.14E-11 | 17.43513743 |
| ITGB1BP2 | -1.067320004 | 0.70586074 | -9.368788387 | 4.34E-19 | 4.34E-17 | 32.50035121 |
| PAM | -1.067751179 | 3.129054161 | -4.946072182 | 1.09E-06 | 1.69E-05 | 4.570009735 |
| RP11-703I16.1 | -1.067870748 | 1.294972519 | -6.367573089 | 4.98E-10 | 1.41E-08 | 12.02255328 |
| MUC21 | -1.068315705 | 0.303704516 | -5.01578481 | 7.77E-07 | 1.24E-05 | 4.897315722 |
| RP11-401P9.4 | -1.068510582 | 0.62502669 | -6.984732513 | 1.10E-11 | 4.11E-10 | 15.74166127 |
| HSPB2 | -1.068677035 | 0.560007446 | -9.376082795 | 4.10E-19 | 4.11E-17 | 32.55684444 |
| UTRN | -1.068701448 | 1.709068653 | -6.34980167 | 5.54E-10 | 1.56E-08 | 11.91962361 |
| GPC3 | -1.072383028 | 1.824525722 | -3.109945177 | 0.001997011 | 0.013402241 | -2.524677353 |
| CD93 | -1.073363854 | 2.857991783 | -4.626349509 | 4.95E-06 | 6.64E-05 | 3.121498141 |
| ITGA1 | -1.07405927 | 1.588118483 | -6.076644935 | 2.73E-09 | 6.92E-08 | 10.36766376 |
| PER3 | -1.074703615 | 1.498852006 | -6.26755109 | 9.00E-10 | 2.45E-08 | 11.44634094 |
| RND1 | -1.074758053 | 1.120866486 | -6.179403066 | 1.51E-09 | 3.98E-08 | 10.94481735 |
| SOWAHC | -1.075202853 | 3.444447341 | -4.695933407 | 3.59E-06 | 4.95E-05 | 3.429342364 |
| LATS2 | -1.075275521 | 2.098058384 | -6.709359046 | 6.25E-11 | 2.07E-09 | 14.0478609 |
| TMTC1 | -1.07532792 | 0.807298509 | -6.283647653 | 8.19E-10 | 2.25E-08 | 11.53856063 |
| LDLR | -1.075854222 | 3.017135108 | -3.913762901 | 0.000105777 | 0.001037982 | 0.211416392 |
| TNNT2 | -1.076023119 | 0.638784158 | -6.517783627 | 2.02E-10 | 6.21E-09 | 12.90199567 |
| PARD3B | -1.076050517 | 0.732846595 | -9.381340879 | 3.93E-19 | 3.96E-17 | 32.59758477 |
| PARVA | -1.076610668 | 3.00395582 | -6.773243132 | 4.20E-11 | 1.43E-09 | 14.43593726 |
| KLHL13 | -1.077635596 | 0.654934925 | -7.7900491 | 5.17E-14 | 2.70E-12 | 20.99863489 |
| EPM2A | -1.079836673 | 0.841851241 | -11.30981389 | 4.13E-26 | 9.21E-24 | 48.46623391 |
| CDC42EP3 | -1.082116337 | 2.296172955 | -5.433352821 | 9.33E-08 | 1.77E-06 | 6.942204705 |
| NCKAP1L | -1.083963115 | 1.274581347 | -4.797129593 | 2.23E-06 | 3.23E-05 | 3.884420812 |
| CACNA1C | -1.08523936 | 0.573635553 | -8.416241656 | 5.98E-16 | 4.06E-14 | 25.3811874 |
| PPP1R12A | -1.086856027 | 2.598790657 | -8.027230079 | 9.82E-15 | 5.77E-13 | 22.62937586 |
| CLMP | -1.087061663 | 1.914449136 | -3.677090487 | 0.000266108 | 0.002352343 | -0.655399684 |
| RGN | -1.087680015 | 0.47561093 | -8.499095939 | 3.25E-16 | 2.32E-14 | 25.9793435 |
| CDO1 | -1.089732774 | 0.51193222 | -6.840247018 | 2.76E-11 | 9.63E-10 | 14.84614073 |
| TBX5 | -1.090912047 | 0.451760969 | -9.323799565 | 6.17E-19 | 6.10E-17 | 32.15256371 |
| SLC25A4 | -1.091768467 | 2.645869318 | -6.978192812 | 1.15E-11 | 4.27E-10 | 15.70080652 |
| LAMB2 | -1.092088709 | 4.357735128 | -4.91815701 | 1.25E-06 | 1.91E-05 | 4.440089833 |
| CBX6 | -1.094324326 | 2.847701559 | -4.747031177 | 2.82E-06 | 4.00E-05 | 3.658038496 |
| FAM50B | -1.09510195 | 1.55252326 | -4.613429696 | 5.25E-06 | 6.99E-05 | 3.064796906 |
| MAPRE2 | -1.098418349 | 2.190577138 | -5.82916807 | 1.10E-08 | 2.50E-07 | 9.011207296 |
| SGCB | -1.099183792 | 2.65060041 | -5.759374061 | 1.62E-08 | 3.56E-07 | 8.637321609 |
| ANGPTL7 | -1.099590867 | 0.185224592 | -12.66549934 | 2.03E-31 | 8.45E-29 | 60.56386446 |
| DLC1 | -1.101771717 | 1.440704921 | -6.595384037 | 1.26E-10 | 4.01E-09 | 13.36289297 |
| PECAM1 | -1.102964146 | 3.488543624 | -4.744495665 | 2.86E-06 | 4.03E-05 | 3.646637921 |
| ARHGEF26 | -1.105449507 | 1.168683046 | -6.314123868 | 6.84E-10 | 1.89E-08 | 11.71370055 |
| COL21A1 | -1.105709573 | 0.462293701 | -8.472997802 | 3.94E-16 | 2.77E-14 | 25.79048226 |
| CTSF | -1.105756781 | 3.707937531 | -4.589820544 | 5.85E-06 | 7.72E-05 | 2.961553881 |
| CYYR1 | -1.106079932 | 1.733377624 | -6.824263516 | 3.05E-11 | 1.06E-09 | 14.74799418 |
| ABL1 | -1.107037866 | 3.761111788 | -7.601195503 | 1.89E-13 | 9.06E-12 | 19.72642429 |
| IGSF10 | -1.108034881 | 0.229547326 | -11.71066878 | 1.19E-27 | 3.09E-25 | 51.97203053 |
| TMEM47 | -1.111732959 | 3.115655217 | -3.516878995 | 0.00048361 | 0.003974179 | -1.213375477 |
| FOXN3 | -1.111893398 | 2.417113578 | -7.802653441 | 4.74E-14 | 2.48E-12 | 21.0843792 |
| MYCT1 | -1.113605309 | 1.297125294 | -7.389968274 | 7.84E-13 | 3.43E-11 | 18.33169992 |
| ADRB3 | -1.114700875 | 0.220480919 | -12.14289519 | 2.44E-29 | 7.88E-27 | 55.82123018 |
| ZEB2 | -1.114736321 | 0.887384678 | -8.309439605 | 1.30E-15 | 8.47E-14 | 24.61632267 |
| WEE1 | -1.115035798 | 2.768394826 | -6.462061238 | 2.83E-10 | 8.42E-09 | 12.57379216 |
| PDE7B | -1.116226224 | 0.925276822 | -7.040635146 | 7.72E-12 | 2.93E-10 | 16.09213236 |

| | | | | | | |
|---|---|---|---|---|---|---|
| GAS1 | -1.117097434 | 1.831895731 | -3.283145997 | 0.00111128 | 0.008126793 | -1.985256091 |
| HSPG2 | -1.117792804 | 3.238666222 | -4.474429775 | 9.85E-06 | 0.000123239 | 2.463863081 |
| ARHGEF37 | -1.118266391 | 1.409526439 | -6.108923039 | 2.27E-09 | 5.82E-08 | 10.54808455 |
| C1orf21 | -1.118731646 | 2.440076618 | -6.527537245 | 1.91E-10 | 5.88E-09 | 12.95968131 |
| LRCH2 | -1.118854921 | 0.630173891 | -8.38821291 | 7.34E-16 | 4.92E-14 | 25.17978261 |
| S100B | -1.119083152 | 1.47953914 | -4.535192915 | 7.49E-06 | 9.62E-05 | 2.724506749 |
| PTPRN2 | -1.119585385 | 0.870093759 | -6.214663149 | 1.23E-09 | 3.29E-08 | 11.14472207 |
| SPRY2 | -1.119912084 | 2.34241924 | -4.947286388 | 1.09E-06 | 1.69E-05 | 4.575675634 |
| MORN5 | -1.121604069 | 0.175666248 | -12.18908238 | 1.60E-29 | 5.29E-27 | 56.23658207 |
| OAT | -1.124195672 | 3.458846121 | -3.424867158 | 0.000674796 | 0.005298458 | -1.523229713 |
| ODF3L1 | -1.125846501 | 0.433281629 | -11.33600738 | 3.29E-26 | 7.38E-24 | 48.6933507 |
| WNT5B | -1.125873211 | 1.656262791 | -3.979216487 | 8.13E-05 | 0.000821674 | 0.460025535 |
| SOX7 | -1.125902325 | 1.572444946 | -4.272436319 | 2.39E-05 | 0.000272741 | 1.62048056 |
| CHRDL2 | -1.126992398 | 1.415262118 | -3.180059707 | 0.001580111 | 0.010968723 | -2.309666842 |
| EHD2 | -1.12726649 | 4.627151728 | -3.944561898 | 9.35E-05 | 0.000931268 | 0.327921513 |
| CDKN1A | -1.128008965 | 6.079818962 | -4.32385091 | 1.91E-05 | 0.0002236 | 1.831772069 |
| PTH1R | -1.12886355 | 0.499815359 | -10.63495882 | 1.39E-23 | 2.34E-21 | 42.71509542 |
| DUSP8 | -1.129509701 | 1.105549514 | -7.076714998 | 6.12E-12 | 2.35E-10 | 16.31950037 |
| ATOH8 | -1.12955501 | 1.140702674 | -4.199410761 | 3.26E-05 | 0.00036096 | 1.324366376 |
| CRNN | -1.129865128 | 0.243933035 | -4.427821643 | 1.21E-05 | 0.000148146 | 2.266105527 |
| PNMA1 | -1.130661576 | 3.465370744 | -4.775859833 | 2.47E-06 | 3.53E-05 | 3.788047384 |
| CD79B | -1.132370149 | 1.276922894 | -4.146472376 | 4.08E-05 | 0.000442199 | 1.112641856 |
| FMOD | -1.133062022 | 3.02616016 | -3.45313342 | 0.000609626 | 0.004850674 | -1.428867728 |
| LDB2 | -1.134024453 | 1.274290013 | -7.824468977 | 4.07E-14 | 2.15E-12 | 21.23303011 |
| HIPK3 | -1.135125382 | 3.061831377 | -7.613980233 | 1.73E-13 | 8.40E-12 | 19.81180309 |
| LRRC32 | -1.135621271 | 2.612207736 | -4.339769266 | 1.78E-05 | 0.000210113 | 1.897658728 |
| ATP8B2 | -1.137329869 | 1.613521959 | -5.526718374 | 5.70E-08 | 1.13E-06 | 7.418933659 |
| ADAMTSL4 | -1.137348573 | 2.009300892 | -3.71100313 | 0.00023384 | 0.002098877 | -0.534298218 |
| NAP1L2 | -1.137642321 | 0.789420853 | -8.078204929 | 6.84E-15 | 4.10E-13 | 22.98456508 |
| PLEKHO1 | -1.137685786 | 3.058745945 | -4.796630886 | 2.24E-06 | 3.23E-05 | 3.882156772 |
| CACNB2 | -1.138207009 | 0.485666532 | -10.16385187 | 7.16E-22 | 1.02E-19 | 38.82186742 |
| RP11-617F23.1 | -1.1382845 | 1.039672656 | -8.443588945 | 4.89E-16 | 3.38E-14 | 25.57815705 |
| MT-ND6 | -1.138740516 | 10.37017663 | -3.330756762 | 0.000941626 | 0.007052172 | -1.832105522 |
| RGCC | -1.139583699 | 3.774367587 | -5.318109386 | 1.70E-07 | 3.08E-06 | 6.363522485 |
| OXER1 | -1.143490471 | 0.96702127 | -7.653751337 | 1.32E-13 | 6.50E-12 | 20.07809739 |
| CDC42EP2 | -1.144622019 | 2.914997793 | -5.355202239 | 1.40E-07 | 2.58E-06 | 6.548598691 |
| RCSD1 | -1.146447723 | 1.290601087 | -5.768991675 | 1.54E-08 | 3.39E-07 | 8.688614677 |
| RP11-404P21.9 | -1.147319564 | 0.746392525 | -7.189733174 | 2.94E-12 | 1.18E-10 | 17.03763025 |
| FAM46A | -1.147618984 | 2.307268762 | -6.534970355 | 1.82E-10 | 5.64E-09 | 13.00369014 |
| OSR1 | -1.148508129 | 1.921665459 | -3.735774835 | 0.000212638 | 0.001928742 | -0.445180589 |
| PCDH18 | -1.148852896 | 1.042939939 | -7.194175741 | 2.85E-12 | 1.15E-10 | 17.06604096 |
| SCUBE2 | -1.149248605 | 2.328579504 | -2.627218948 | 0.008919511 | 0.04671123 | -3.879903088 |
| TMEM132C | -1.149258266 | 0.156302799 | -16.70987812 | 1.47E-48 | 3.74E-45 | 99.66915626 |
| ZBTB16 | -1.151201861 | 0.2693638 | -12.12196542 | 2.95E-29 | 9.42E-27 | 55.63326332 |
| KIAA0513 | -1.152328103 | 1.493948085 | -7.404145705 | 7.13E-13 | 3.15E-11 | 18.4243679 |
| NRN1 | -1.153991249 | 1.506453198 | -4.307550011 | 2.05E-05 | 0.00023793 | 1.764531783 |
| MIR24-2 | -1.153994406 | 0.364738191 | -8.63415187 | 1.20E-16 | 9.07E-15 | 26.96322814 |
| PPP1R15A | -1.156787592 | 5.386755012 | -5.768414228 | 1.54E-08 | 3.40E-07 | 8.685532949 |
| CPEB2 | -1.157400912 | 1.42558559 | -8.723833164 | 6.13E-17 | 4.73E-15 | 27.62255593 |
| TCEAL1 | -1.157630379 | 3.533712681 | -6.65410442 | 8.79E-11 | 2.86E-09 | 13.71460314 |
| ENPP2 | -1.15797788 | 1.74970226 | -4.703664685 | 3.46E-06 | 4.80E-05 | 3.463801848 |
| TBX4 | -1.158602492 | 0.561423378 | -6.791862246 | 3.74E-11 | 1.28E-09 | 14.54959972 |
| CTSK | -1.158724428 | 4.683293 | -2.985901715 | 0.002990661 | 0.018865141 | -2.89383243 |
| LGALS4 | -1.159027309 | 1.379689371 | -2.70822036 | 0.007037226 | 0.03849377 | -3.667846572 |
| SEMA3G | -1.160747335 | 1.244540356 | -7.695808572 | 9.90E-14 | 4.93E-12 | 20.36084022 |
| RBMS3 | -1.163001387 | 0.686933012 | -9.499850345 | 1.54E-19 | 1.66E-17 | 33.51976274 |
| SEMA3E | -1.165089349 | 0.543166238 | -8.832250539 | 2.71E-17 | 2.19E-15 | 28.42593682 |
| TRPA1 | -1.165197716 | 0.741346315 | -5.656494204 | 2.84E-08 | 5.96E-07 | 8.093226419 |
| NGFR | -1.166080981 | 1.18567002 | -4.149458285 | 4.03E-05 | 0.000437342 | 1.124517995 |
| SERPINE2 | -1.166285633 | 1.682646155 | -3.920109244 | 0.000103125 | 0.001014513 | 0.235353607 |
| FBN1 | -1.166294906 | 1.946156427 | -4.076960285 | 5.45E-05 | 0.000573356 | 0.838395354 |
| PBX1 | -1.166633665 | 2.275084701 | -5.646864804 | 2.99E-08 | 6.24E-07 | 8.042730795 |
| COL16A1 | -1.16782382 | 2.759385272 | -3.629028579 | 0.000319072 | 0.002753826 | -0.825240513 |
| GALNT15 | -1.168891187 | 0.610745019 | -7.541758767 | 2.83E-13 | 1.32E-11 | 19.33092871 |
| SERPING1 | -1.17059027 | 5.362325638 | -3.102367366 | 0.002047674 | 0.013699727 | -2.547641077 |
| STOM | -1.171155094 | 5.141816224 | -3.974384612 | 8.29E-05 | 0.000836039 | 0.441541971 |
| ARL4D | -1.171655788 | 3.876712182 | -3.663916297 | 0.000279738 | 0.002456096 | -0.702163228 |
| MEIS1 | -1.171941689 | 1.396764127 | -8.560128267 | 2.07E-16 | 1.53E-14 | 26.42261175 |
| RUSC2 | -1.175017497 | 2.163535013 | -6.48079695 | 2.53E-10 | 7.61E-09 | 12.68388744 |
| RECK | -1.176302811 | 0.957369303 | -8.973640661 | 9.26E-18 | 7.80E-16 | 29.48387949 |

| | | | | | | |
|---|---|---|---|---|---|---|
| HAND2-AS1 | -1.176794284 | 0.206035309 | -13.91096415 | 1.59E-36 | 1.07E-33 | 72.20875145 |
| SYNE1 | -1.178464287 | 0.642206918 | -11.8120142 | 4.83E-28 | 1.31E-25 | 52.8683139 |
| KCNJ8 | -1.178916383 | 1.55841378 | -5.453703133 | 8.38E-08 | 1.61E-06 | 7.045513423 |
| CRISP3 | -1.179541237 | 1.019299351 | -3.245082448 | 0.001266867 | 0.009084813 | -2.106188278 |
| OSBPL10 | -1.180512247 | 1.507593143 | -7.531907092 | 3.02E-13 | 1.40E-11 | 19.26560376 |
| ILK | -1.180609016 | 1.80262756 | -8.064117749 | 7.56E-15 | 4.51E-13 | 22.8862416 |
| ETS2 | -1.181553817 | 5.478912207 | -4.922840108 | 1.22E-06 | 1.88E-05 | 4.461839658 |
| LSP1 | -1.182278952 | 3.291202421 | -3.309482734 | 0.001014217 | 0.007516766 | -1.900796916 |
| ATP1B2 | -1.183024556 | 0.554128723 | -11.45745648 | 1.13E-26 | 2.67E-24 | 49.75004525 |
| CACNA2D1 | -1.184073719 | 0.824313284 | -6.640830373 | 9.54E-11 | 3.09E-09 | 13.63487588 |
| JUND | -1.184420227 | 6.40442784 | -6.005580351 | 4.09E-09 | 1.00E-07 | 9.973275586 |
| ADRA2A | -1.186080227 | 0.949619847 | -6.368896729 | 4.94E-10 | 1.40E-08 | 12.03022912 |
| NFIB | -1.186126196 | 2.032773785 | -5.984921801 | 4.60E-09 | 1.12E-07 | 9.859359874 |
| MSX1 | -1.186529841 | 1.420875716 | -5.448297189 | 8.62E-08 | 1.65E-06 | 7.018037315 |
| SCN7A | -1.187669494 | 0.183130736 | -14.11972771 | 2.12E-37 | 1.53E-34 | 74.20209828 |
| FAM149A | -1.188163716 | 0.859141953 | -7.330705105 | 1.16E-12 | 4.97E-11 | 17.94582382 |
| NAP1L5 | -1.188803171 | 1.586187175 | -8.180546446 | 3.30E-15 | 2.06E-13 | 23.70263392 |
| DOK6 | -1.18902661 | 0.296348759 | -11.64961579 | 2.06E-27 | 5.22E-25 | 51.43398691 |
| COL6A3 | -1.189235049 | 3.997365084 | -3.005977767 | 0.002803973 | 0.017898278 | -2.835061672 |
| THRA | -1.189972971 | 2.236554479 | -7.344261141 | 1.06E-12 | 4.57E-11 | 18.03387831 |
| PDLIM7 | -1.191275568 | 4.009211268 | -4.794678806 | 2.26E-06 | 3.26E-05 | 3.873296694 |
| KIAA1462 | -1.19170765 | 1.828568094 | -5.287916771 | 1.98E-07 | 3.55E-06 | 6.21370626 |
| NECAB1 | -1.191710016 | 0.46368709 | -10.12026689 | 1.03E-21 | 1.44E-19 | 38.46703352 |
| LIF | -1.194344091 | 2.156052188 | -4.102669319 | 4.90E-05 | 0.000521194 | 0.939326487 |
| APCDD1 | -1.194695629 | 2.528756775 | -4.310063548 | 2.03E-05 | 0.000235588 | 1.77488481 |
| SLC22A3 | -1.195677882 | 0.926084963 | -5.596632076 | 3.92E-08 | 8.00E-07 | 7.780516352 |
| STC1 | -1.199691937 | 2.691918846 | -4.291881387 | 2.20E-05 | 0.000252999 | 1.700118915 |
| PLCD4 | -1.200060728 | 0.661931162 | -13.02116639 | 7.39E-33 | 3.54E-30 | 63.84281954 |
| EML1 | -1.201318469 | 1.149940885 | -7.155292503 | 3.68E-12 | 1.46E-10 | 16.81784455 |
| TEAD1 | -1.201936758 | 2.618695726 | -6.922402383 | 1.64E-11 | 5.94E-10 | 15.35351002 |
| NBEA | -1.20289786 | 0.554511832 | -9.245891161 | 1.13E-18 | 1.08E-16 | 31.55290524 |
| TNFSF12 | -1.203884581 | 3.156487207 | -5.982816264 | 4.66E-09 | 1.13E-07 | 9.847768091 |
| SAMD4A | -1.205127944 | 1.047624262 | -8.243518585 | 2.10E-15 | 1.34E-13 | 24.14773596 |
| MEF2C | -1.206847114 | 1.10207629 | -8.501263135 | 3.20E-16 | 2.29E-14 | 25.99504509 |
| FBXO32 | -1.207043803 | 2.913884776 | -4.514845077 | 8.21E-06 | 0.00010476 | 2.636868993 |
| LRFN5 | -1.207397212 | 0.299731105 | -11.1679634 | 1.43E-25 | 3.02E-23 | 47.24120214 |
| NFASC | -1.208787694 | 0.547121577 | -10.96778095 | 8.08E-25 | 1.58E-22 | 45.52680882 |
| TRIB1 | -1.209190316 | 4.561970596 | -5.509631607 | 6.24E-08 | 1.22E-06 | 7.331161443 |
| FXYD1 | -1.212484905 | 0.326992667 | -14.85582308 | 1.63E-40 | 1.53E-37 | 81.31175484 |
| VCL | -1.214169581 | 4.136409024 | -6.696147827 | 6.78E-11 | 2.24E-09 | 13.96797719 |
| ANK2 | -1.215249472 | 0.374678039 | -11.57424677 | 4.02E-27 | 9.94E-25 | 50.77177603 |
| PPP1R3B | -1.216964089 | 2.620076197 | -6.165784193 | 1.63E-09 | 4.28E-08 | 10.86786012 |
| PLA2G4A | -1.217519081 | 2.321776768 | -4.884640442 | 1.47E-06 | 2.22E-05 | 4.28496734 |
| AKT3 | -1.219230594 | 1.575743178 | -5.808810689 | 1.23E-08 | 2.78E-07 | 8.901756341 |
| KIT | -1.223008675 | 1.289317784 | -4.460739592 | 1.05E-05 | 0.000130028 | 2.405580305 |
| GPC6 | -1.223372826 | 1.159348396 | -5.345132282 | 1.48E-07 | 2.71E-06 | 6.498243105 |
| SRGN | -1.229441175 | 5.198109508 | -3.240398781 | 0.001287348 | 0.009207933 | -2.120976128 |
| AMOTL1 | -1.230476203 | 2.487667884 | -5.636660691 | 3.16E-08 | 6.57E-07 | 7.989302183 |
| NFATC4 | -1.237280568 | 1.890835064 | -6.50178815 | 2.23E-10 | 6.78E-09 | 12.80754663 |
| GLIPR2 | -1.238109138 | 2.562711283 | -4.563014186 | 6.61E-06 | 8.61E-05 | 2.844910918 |
| DMPK | -1.238448836 | 3.217082137 | -7.236309205 | 2.17E-12 | 8.95E-11 | 17.33617061 |
| C20orf166-AS1 | -1.24386922 | 0.207965873 | -13.79975817 | 4.62E-36 | 2.93E-33 | 71.15140199 |
| ITGA9 | -1.24840907 | 1.053695391 | -8.214024095 | 2.59E-15 | 1.64E-13 | 23.93895385 |
| CSDC2 | -1.249832474 | 0.884830985 | -5.816169875 | 1.18E-08 | 2.67E-07 | 8.941285175 |
| LPPR4 | -1.250124877 | 0.461787126 | -10.37429121 | 1.25E-22 | 1.89E-20 | 40.54800879 |
| TMEM88 | -1.250197994 | 2.059640762 | -6.690975842 | 7.00E-11 | 2.31E-09 | 13.93673864 |
| AEBP1 | -1.252379744 | 4.885370874 | -2.704739139 | 0.00711011 | 0.038812888 | -3.677088164 |
| NPY6R | -1.254504919 | 0.17818666 | -12.65665937 | 2.20E-31 | 9.11E-29 | 60.48287962 |
| SH3BGRL | -1.255013453 | 5.080514633 | -5.611052744 | 3.63E-08 | 7.45E-07 | 7.855585614 |
| PLA2G5 | -1.256917408 | 0.637657862 | -7.601373199 | 1.89E-13 | 9.06E-12 | 19.72761023 |
| KCNK3 | -1.258385982 | 0.503352449 | -7.957478588 | 1.61E-14 | 9.07E-13 | 22.14603647 |
| PITX1 | -1.259338219 | 3.339753178 | -3.786368637 | 0.000174836 | 0.00162189 | -0.261443069 |
| RND3 | -1.261046302 | 4.208550386 | -5.039519386 | 6.91E-07 | 1.12E-05 | 5.009679665 |
| SGCG | -1.261317289 | 0.213178257 | -13.94384408 | 1.16E-36 | 7.95E-34 | 72.52197919 |
| ROR2 | -1.262661434 | 1.380442542 | -5.202838259 | 3.06E-07 | 5.25E-06 | 5.795571926 |
| CCDC3 | -1.265259463 | 2.300602286 | -5.748264962 | 1.72E-08 | 3.77E-07 | 8.578165142 |
| STARD13 | -1.265985678 | 1.223287439 | -8.453224484 | 4.56E-16 | 3.17E-14 | 25.64766564 |
| PPM1L | -1.266096203 | 1.432141136 | -7.428669384 | 6.06E-13 | 2.70E-11 | 18.58498503 |
| CREB3L1 | -1.266924534 | 2.613615078 | -4.039823607 | 6.35E-05 | 0.000656687 | 0.69363645 |
| IGFBP2 | -1.267464513 | 3.158641883 | -3.253253807 | 0.001231856 | 0.008867512 | -2.080340009 |

| | | | | | | |
|---|---|---|---|---|---|---|
| TLN1 | -1.268294127 | 4.858001759 | -7.286608529 | 1.56E-12 | 6.56E-11 | 17.66026314 |
| PLSCR4 | -1.269739708 | 1.439979258 | -6.858322938 | 2.46E-11 | 8.70E-10 | 14.95735726 |
| MPZ | -1.270598176 | 0.93214798 | -9.496232587 | 1.59E-19 | 1.70E-17 | 33.49149989 |
| CCL14 | -1.270921235 | 0.315288908 | -13.09578596 | 3.67E-33 | 1.80E-30 | 64.53571641 |
| ARID5B | -1.271542009 | 2.968883844 | -6.946802696 | 1.41E-11 | 5.14E-10 | 15.5051297 |
| FOLR2 | -1.271723206 | 2.473640701 | -3.770810876 | 0.000185726 | 0.001712572 | -0.318189069 |
| ID2 | -1.274859142 | 4.043051151 | -4.417926561 | 1.27E-05 | 0.0001541 | 2.224363681 |
| SGCD | -1.274943096 | 0.532340171 | -9.46015986 | 2.11E-19 | 2.21E-17 | 33.21007276 |
| SCARA3 | -1.277757616 | 2.836899932 | -3.993321588 | 7.67E-05 | 0.000779044 | 0.514101518 |
| GNAZ | -1.278284603 | 0.771441789 | -7.381011583 | 8.32E-13 | 3.64E-11 | 18.2732269 |
| SELP | -1.279476106 | 1.140131513 | -5.790186964 | 1.37E-08 | 3.05E-07 | 8.801912277 |
| SMIM10 | -1.28108647 | 1.873050582 | -6.398205247 | 4.15E-10 | 1.20E-08 | 12.20052748 |
| CPE | -1.281636822 | 2.851154358 | -4.322078716 | 1.93E-05 | 0.000225012 | 1.824450602 |
| PPP1R1B | -1.281982559 | 1.466566299 | -3.334959527 | 0.00092787 | 0.006960751 | -1.818485864 |
| CD55 | -1.284033522 | 4.355152509 | -4.964987626 | 9.96E-07 | 1.56E-05 | 4.658416281 |
| TBX20 | -1.285414841 | 0.317154854 | -10.61242586 | 1.68E-23 | 2.79E-21 | 42.52652789 |
| RGAG4 | -1.286212165 | 0.848280464 | -8.022118124 | 1.02E-14 | 5.97E-13 | 22.59384728 |
| FGF10 | -1.286444305 | 0.222222902 | -14.11470993 | 2.23E-37 | 1.59E-34 | 74.15406088 |
| PDE4D | -1.287155541 | 1.163296785 | -10.02948041 | 2.16E-21 | 2.89E-19 | 37.73091812 |
| ACKR3 | -1.287803399 | 4.251037366 | -3.895023874 | 0.00011399 | 0.00110926 | 0.140946887 |
| ARID5A | -1.291319338 | 2.975162426 | -6.842392936 | 2.72E-11 | 9.52E-10 | 14.85933172 |
| HAND1 | -1.291559916 | 0.282478379 | -8.957624893 | 1.05E-17 | 8.78E-16 | 29.36346639 |
| CTD-3252C9.4 | -1.293170946 | 3.519850244 | -5.114553435 | 4.77E-07 | 7.93E-06 | 5.367994738 |
| KCTD12 | -1.293314591 | 3.089735136 | -5.177441236 | 3.48E-07 | 5.91E-06 | 5.671909415 |
| C1orf95 | -1.293348531 | 0.318993147 | -9.797690969 | 1.42E-20 | 1.71E-18 | 35.87019458 |
| PDE4B | -1.297076146 | 0.976353914 | -7.865600023 | 3.06E-14 | 1.65E-12 | 21.51413936 |
| CRY2 | -1.298856924 | 2.339338068 | -11.48132026 | 9.14E-27 | 2.20E-24 | 49.95837513 |
| PDE1A | -1.299886869 | 0.557324059 | -10.97659085 | 7.49E-25 | 1.48E-22 | 45.60189744 |
| RP11-753H16.3 | -1.300743644 | 0.294743848 | -10.23559658 | 3.95E-22 | 5.73E-20 | 39.40796706 |
| TPSD1 | -1.301015331 | 0.549784124 | -7.87559703 | 2.85E-14 | 1.55E-12 | 21.5826296 |
| TGFB3 | -1.301067311 | 2.096454844 | -4.998741051 | 8.45E-07 | 1.34E-05 | 4.816917912 |
| TNFAIP3 | -1.302702573 | 3.288456013 | -4.430952437 | 1.19E-05 | 0.000146319 | 2.279330337 |
| FYCO1 | -1.303088455 | 2.625980931 | -8.201383788 | 2.84E-15 | 1.78E-13 | 23.84964317 |
| NTN1 | -1.303494813 | 1.000740698 | -5.996701427 | 4.30E-09 | 1.05E-07 | 9.924274709 |
| KANK1 | -1.30361304 | 2.408314058 | -6.616309869 | 1.11E-10 | 3.56E-09 | 13.4879394 |
| NCALD | -1.306165288 | 1.011726333 | -8.508635414 | 3.03E-16 | 2.18E-14 | 26.04847935 |
| JAZF1 | -1.306506635 | 1.641343739 | -7.560595513 | 2.49E-13 | 1.17E-11 | 19.45601359 |
| LINC00675 | -1.306641895 | 1.621260336 | -3.578042377 | 0.000385991 | 0.00326007 | -1.003118883 |
| CPXM1 | -1.307537871 | 2.843655092 | -3.40649253 | 0.000720586 | 0.005603738 | -1.584176638 |
| ASPA | -1.308109663 | 0.318601719 | -15.07195507 | 1.94E-41 | 2.05E-38 | 83.4212601 |
| BVES | -1.309816846 | 0.793974235 | -7.421029088 | 6.37E-13 | 2.83E-11 | 18.53490128 |
| LIFR | -1.310185245 | 1.02008511 | -7.369466879 | 8.99E-13 | 3.91E-11 | 18.19793901 |
| RARRES2 | -1.310523597 | 4.162276861 | -3.359537964 | 0.00085109 | 0.00646534 | -1.738509742 |
| CDIP1 | -1.31113146 | 2.072269112 | -5.439665058 | 9.02E-08 | 1.72E-06 | 6.97421297 |
| CSPG4 | -1.312070633 | 2.217959395 | -4.790555796 | 2.30E-06 | 3.32E-05 | 3.854593866 |
| TSPYL2 | -1.316650145 | 2.634876484 | -8.098771946 | 5.91E-15 | 3.58E-13 | 23.12834119 |
| NDNF | -1.318806195 | 0.496696668 | -8.88090289 | 1.88E-17 | 1.54E-15 | 28.78867635 |
| NES | -1.321572405 | 2.81102464 | -5.033719335 | 7.11E-07 | 1.15E-05 | 4.982177686 |
| ACOX2 | -1.324360776 | 0.562181686 | -10.80506163 | 3.27E-24 | 5.93E-22 | 44.14600165 |
| CD69 | -1.324379783 | 1.331883678 | -5.260985613 | 2.28E-07 | 4.03E-06 | 6.080704173 |
| ARHGAP6 | -1.324936091 | 0.730973302 | -7.610742401 | 1.77E-13 | 8.58E-12 | 19.79016994 |
| RP11-6O2.3 | -1.327149398 | 0.307763501 | -13.20625868 | 1.30E-33 | 6.77E-31 | 65.56459087 |
| P2RY14 | -1.327834085 | 0.546509018 | -11.91393109 | 1.93E-28 | 5.61E-26 | 53.77356226 |
| ADH1C | -1.330822893 | 1.315288047 | -3.3941911 | 0.000752843 | 0.005819708 | -1.624805873 |
| TGFBR2 | -1.331220395 | 3.994501569 | -6.177767484 | 1.52E-09 | 4.02E-08 | 10.93556755 |
| GNAO1 | -1.332395641 | 0.451777159 | -9.844787141 | 9.73E-21 | 1.20E-18 | 36.24606602 |
| DAPL1 | -1.33386199 | 1.012467828 | -3.904350768 | 0.00010983 | 0.001073781 | 0.175982028 |
| EMP3 | -1.337146065 | 3.641919535 | -4.011394479 | 7.13E-05 | 0.000729328 | 0.583648477 |
| AXL | -1.338250983 | 2.783962903 | -4.306371907 | 2.06E-05 | 0.00023891 | 1.759681182 |
| RBP7 | -1.339462042 | 1.950104281 | -5.113002973 | 4.80E-07 | 7.99E-06 | 5.360543324 |
| ADARB1 | -1.34098351 | 1.66417087 | -6.845289906 | 2.67E-11 | 9.39E-10 | 14.87714471 |
| CLEC10A | -1.341436733 | 1.020865689 | -6.424894299 | 3.54E-10 | 1.03E-08 | 12.35616534 |
| PHYHD1 | -1.341511373 | 1.321179168 | -5.690717964 | 2.36E-08 | 5.02E-07 | 8.273290398 |
| SRF | -1.34310841 | 4.111002805 | -10.99625723 | 6.32E-25 | 1.27E-22 | 45.76963898 |
| RASSF3 | -1.343457213 | 3.25539703 | -7.329148446 | 1.17E-12 | 5.02E-11 | 17.93572045 |
| AFF3 | -1.344022772 | 0.261519174 | -13.46232334 | 1.15E-34 | 6.67E-32 | 67.96304416 |
| PDLIM4 | -1.344573372 | 2.415455673 | -4.365294071 | 1.60E-05 | 0.000189956 | 2.003769009 |
| RNF122 | -1.346267161 | 2.53572781 | -7.789417543 | 5.19E-14 | 2.70E-12 | 20.99434129 |
| RPS6KA2 | -1.34663867 | 1.965789121 | -7.865187385 | 3.07E-14 | 1.65E-12 | 21.51131374 |
| KLF6 | -1.347508617 | 4.957231172 | -6.543448583 | 1.73E-10 | 5.39E-09 | 13.05393667 |

| | | | | | | |
|---|---|---|---|---|---|---|
| GFRA3 | -1.348241566 | 0.72753735 | -7.056152002 | 6.99E-12 | 2.66E-10 | 16.18980405 |
| RASGRP2 | -1.348600511 | 0.793109613 | -8.319712225 | 1.21E-15 | 7.89E-14 | 24.68958562 |
| ODC1 | -1.348740014 | 4.58538079 | -4.903240342 | 1.34E-06 | 2.04E-05 | 4.370935095 |
| DKK1 | -1.351296131 | 2.718749279 | -2.903652018 | 0.00388035 | 0.023519359 | -3.130664407 |
| SSPN | -1.355386896 | 1.802321182 | -7.717141517 | 8.55E-14 | 4.28E-12 | 20.50470443 |
| SYT11 | -1.35557555 | 1.571547368 | -6.51084802 | 2.11E-10 | 6.47E-09 | 12.86101944 |
| MMP2 | -1.356111954 | 5.668428941 | -3.462422809 | 0.000589526 | 0.004710637 | -1.397696652 |
| THSD4 | -1.358274222 | 1.133907187 | -9.266923626 | 9.63E-19 | 9.26E-17 | 31.71446331 |
| DPYSL2 | -1.358813913 | 2.624318656 | -5.926642922 | 6.39E-09 | 1.51E-07 | 9.539785415 |
| VIM | -1.360313697 | 5.940089285 | -4.173228714 | 3.64E-05 | 0.000399658 | 1.219343353 |
| DCHS1 | -1.360861863 | 1.284356959 | -7.57140126 | 2.31E-13 | 1.09E-11 | 19.52787616 |
| TMEM100 | -1.361076976 | 0.437220571 | -10.82762812 | 2.69E-24 | 4.93E-22 | 44.33680224 |
| PALM | -1.362705336 | 1.594302003 | -5.215134709 | 2.87E-07 | 4.96E-06 | 5.855636765 |
| LYNX1 | -1.364490465 | 1.57897626 | -4.899224797 | 1.37E-06 | 2.08E-05 | 4.352350766 |
| MCAM | -1.366032247 | 3.57188168 | -5.907702774 | 7.11E-09 | 1.67E-07 | 9.436496313 |
| TACC1 | -1.367750885 | 3.003933089 | -5.609644547 | 3.66E-08 | 7.49E-07 | 7.848247665 |
| EMCN | -1.368001122 | 1.208369744 | -8.373198175 | 8.19E-16 | 5.47E-14 | 25.07208948 |
| ACTA2-AS1 | -1.368123815 | 0.558753086 | -9.686114319 | 3.50E-20 | 4.00E-18 | 34.98425678 |
| TSHZ3 | -1.371849221 | 1.246379624 | -7.145294426 | 3.93E-12 | 1.55E-10 | 16.75419602 |
| FOSL1 | -1.372651585 | 3.857487757 | -3.848128309 | 0.000137271 | 0.001307814 | -0.034026668 |
| NPR1 | -1.372947885 | 1.156142289 | -7.598359561 | 1.93E-13 | 9.23E-12 | 19.70750011 |
| HEPH | -1.374644958 | 1.324023131 | -6.27559638 | 8.59E-10 | 2.35E-08 | 11.49240911 |
| C3orf70 | -1.374748305 | 0.911576056 | -8.048714594 | 8.44E-15 | 5.01E-13 | 22.77887719 |
| SYNPO | -1.374771971 | 2.720434556 | -6.5747147 | 1.43E-10 | 4.51E-09 | 13.23969574 |
| WLS | -1.375974814 | 3.770811622 | -5.031851637 | 7.18E-07 | 1.16E-05 | 4.97332764 |
| RP11-887P2.5 | -1.376431339 | 0.195034912 | -14.8909894 | 1.15E-40 | 1.12E-37 | 81.65434909 |
| PJA2 | -1.376910887 | 3.675545256 | -8.821472839 | 2.94E-17 | 2.37E-15 | 28.34576653 |
| ITPKB | -1.377494431 | 2.634292415 | -9.747805592 | 2.13E-20 | 2.51E-18 | 35.47330206 |
| NFIL3 | -1.377575993 | 3.804957401 | -6.366292061 | 5.02E-10 | 1.42E-08 | 12.01512579 |
| TACC2 | -1.380920865 | 1.855568575 | -8.644097999 | 1.11E-16 | 8.47E-15 | 27.03611641 |
| EPHX2 | -1.38486487 | 1.554634045 | -6.743507949 | 5.06E-11 | 1.70E-09 | 14.25493632 |
| CPQ | -1.385077448 | 2.775797892 | -5.971454714 | 4.97E-09 | 1.20E-07 | 9.785277833 |
| CXCL14 | -1.38764979 | 3.617042337 | -2.867603117 | 0.004341505 | 0.025800249 | -3.232461157 |
| PDGFD | -1.388104884 | 1.384307991 | -7.255166937 | 1.91E-12 | 7.97E-11 | 17.45747142 |
| MEOX1 | -1.38903834 | 0.775564741 | -8.452318855 | 4.59E-16 | 3.19E-14 | 25.64113023 |
| CFH | -1.389375725 | 2.896817883 | -4.657622297 | 4.28E-06 | 5.82E-05 | 3.25933811 |
| DNAJB4 | -1.389862755 | 2.292194255 | -8.298865979 | 1.40E-15 | 9.10E-14 | 24.54098102 |
| DENND2A | -1.391911189 | 1.243698867 | -8.011612384 | 1.10E-14 | 6.36E-13 | 22.5208837 |
| LPAR1 | -1.392258954 | 2.086301331 | -7.023382593 | 8.63E-12 | 3.26E-10 | 15.98373479 |
| CYP27A1 | -1.39368514 | 2.292344072 | -5.031404774 | 7.20E-07 | 1.16E-05 | 4.97121062 |
| JUNB | -1.39528268 | 7.503652273 | -6.434274333 | 3.35E-10 | 9.80E-09 | 12.41099178 |
| CAP2 | -1.395370679 | 1.599663012 | -6.809647532 | 3.35E-11 | 1.15E-09 | 14.65840603 |
| RRAD | -1.397938629 | 2.353621566 | -3.425964733 | 0.000672149 | 0.005279801 | -1.51957934 |
| COL6A1 | -1.398482082 | 5.730598077 | -3.548831542 | 0.000430047 | 0.003583946 | -1.10396082 |
| CNRIP1 | -1.399419368 | 1.28946775 | -7.819713634 | 4.21E-14 | 2.22E-12 | 21.20060078 |
| MMRN1 | -1.399849666 | 0.518761455 | -9.932761388 | 4.76E-21 | 6.11E-19 | 36.9512013 |
| RSPO3 | -1.402158162 | 0.705769231 | -7.639588618 | 1.46E-13 | 7.12E-12 | 19.98314793 |
| FAM189A2 | -1.403360165 | 0.738351904 | -10.57775846 | 2.26E-23 | 3.70E-21 | 42.23686585 |
| GFRA1 | -1.404511742 | 0.347349477 | -12.47928528 | 1.13E-30 | 4.25E-28 | 58.86334761 |
| CD200 | -1.406250349 | 1.806719645 | -5.960552745 | 5.28E-09 | 1.27E-07 | 9.725409674 |
| RGS1 | -1.407656673 | 3.077354085 | -4.093014971 | 5.10E-05 | 0.00054055 | 0.901355732 |
| PRKCB | -1.408407628 | 0.767603112 | -8.852289284 | 2.33E-17 | 1.89E-15 | 28.57517474 |
| PTX3 | -1.409276546 | 1.078207991 | -4.918679986 | 1.25E-06 | 1.91E-05 | 4.442517787 |
| RP11-554A11.4 | -1.409480635 | 0.555916347 | -11.10241186 | 2.52E-25 | 5.24E-23 | 46.6779328 |
| AHNAK | -1.410131908 | 5.051467441 | -5.686682253 | 2.41E-08 | 5.13E-07 | 8.252008527 |
| PRKG1 | -1.412098965 | 0.740111864 | -10.09298573 | 1.28E-21 | 1.77E-19 | 38.2454055 |
| LMCD1 | -1.415213613 | 1.884054517 | -5.927397474 | 6.37E-09 | 1.51E-07 | 9.54390612 |
| SGCE | -1.416040396 | 2.48430499 | -5.044679496 | 6.74E-07 | 1.09E-05 | 5.034170862 |
| ABLIM1 | -1.416158865 | 3.261687151 | -6.511611113 | 2.10E-10 | 6.44E-09 | 12.86552613 |
| ANGPTL2 | -1.416576752 | 3.248460224 | -4.615616734 | 5.20E-06 | 6.93E-05 | 3.074385088 |
| TMEM119 | -1.425608409 | 2.221342412 | -4.817969613 | 2.02E-06 | 2.95E-05 | 3.979219548 |
| DSTN | -1.425685983 | 6.588615313 | -9.376048421 | 4.10E-19 | 4.11E-17 | 32.55657815 |
| FAIM2 | -1.426076535 | 0.310452767 | -13.68851944 | 1.34E-35 | 8.05E-33 | 70.09695572 |
| LUM | -1.426248874 | 5.80827435 | -3.195137125 | 0.001501684 | 0.010489149 | -2.262833819 |
| PCDH7 | -1.429085303 | 1.393758794 | -5.501287114 | 6.52E-08 | 1.28E-06 | 7.288382558 |
| EBF1 | -1.430281015 | 0.808570245 | -10.30593286 | 2.20E-22 | 3.26E-20 | 39.98496964 |
| IL6ST | -1.431496794 | 3.090867229 | -7.532364552 | 3.02E-13 | 1.40E-11 | 19.26863566 |
| AARD | -1.433940158 | 0.310135336 | -10.50917234 | 4.02E-23 | 6.37E-21 | 41.66542426 |
| NCS1 | -1.439098128 | 3.173372141 | -6.821725298 | 3.10E-11 | 1.07E-09 | 14.73242521 |
| RBPMS | -1.440934297 | 3.009149342 | -6.574214252 | 1.43E-10 | 4.52E-09 | 13.23671678 |

| | | | | | | |
|---|---|---|---|---|---|---|
| LINC00865 | -1.441388612 | 1.19366683 | -8.611657707 | 1.42E-16 | 1.07E-14 | 26.79860092 |
| PRDM6 | -1.445903244 | 0.558391601 | -11.65193795 | 2.02E-27 | 5.14E-25 | 51.45442515 |
| LGI4 | -1.446063579 | 0.852770348 | -10.59952555 | 1.88E-23 | 3.10E-21 | 42.41867568 |
| C1R | -1.446950926 | 5.069089697 | -4.061025989 | 5.82E-05 | 0.000607536 | 0.776133321 |
| TSC22D3 | -1.447352077 | 4.185880314 | -6.069189889 | 2.85E-09 | 7.19E-08 | 10.32610728 |
| MAFF | -1.447869553 | 3.401231526 | -6.688183922 | 7.13E-11 | 2.34E-09 | 13.91988369 |
| ATP2B4 | -1.448829019 | 3.553785524 | -6.111038678 | 2.24E-09 | 5.75E-08 | 10.55993805 |
| PRICKLE2 | -1.450019509 | 1.185260483 | -9.810191885 | 1.29E-20 | 1.56E-18 | 35.9698531 |
| NXPH3 | -1.45282058 | 0.683306882 | -9.592866973 | 7.37E-20 | 8.17E-18 | 34.24881914 |
| MTURN | -1.453530106 | 2.261604263 | -8.580204234 | 1.79E-16 | 1.33E-14 | 26.56890913 |
| OMD | -1.457652954 | 0.479880346 | -9.424978416 | 2.79E-19 | 2.89E-17 | 32.93627076 |
| GRASP | -1.458240347 | 1.800887529 | -7.859520009 | 3.19E-14 | 1.71E-12 | 21.47251642 |
| EDNRA | -1.459531448 | 1.665581914 | -6.829776018 | 2.95E-11 | 1.02E-09 | 14.78182286 |
| MT1M | -1.45984163 | 1.274388723 | -4.797541456 | 2.23E-06 | 3.22E-05 | 3.886290762 |
| INMT | -1.460500516 | 1.030871468 | -8.707212036 | 6.94E-17 | 5.33E-15 | 27.50000067 |
| RAB9B | -1.460765286 | 0.628366784 | -10.85291581 | 2.17E-24 | 4.06E-22 | 44.55087799 |
| RERGL | -1.460942607 | 0.342214889 | -14.65889054 | 1.12E-39 | 9.63E-37 | 79.39795662 |
| RHOJ | -1.461052355 | 1.496870242 | -8.431100105 | 5.36E-16 | 3.66E-14 | 25.48814935 |
| GSTM5 | -1.463166274 | 0.686008084 | -8.345325697 | 1.00E-15 | 6.62E-14 | 24.87254057 |
| MIR4697HG | -1.46376571 | 0.400847413 | -11.26206826 | 6.28E-26 | 1.37E-23 | 48.05296867 |
| WWTR1 | -1.468198185 | 2.716973136 | -7.894357954 | 2.50E-14 | 1.37E-12 | 21.71133677 |
| ADAMTS4 | -1.469639386 | 1.687785808 | -5.92547142 | 6.44E-09 | 1.52E-07 | 9.533388564 |
| RANBP3L | -1.471554368 | 0.303279434 | -12.30665317 | 5.50E-30 | 1.94E-27 | 57.29726486 |
| RP11-553L6.5 | -1.471803899 | 2.416192133 | -8.356010159 | 9.28E-16 | 6.14E-14 | 24.94897806 |
| FAM43A | -1.474940168 | 2.000443869 | -6.982529828 | 1.12E-11 | 4.16E-10 | 15.72789729 |
| STON1 | -1.475442796 | 1.1024855 | -9.209155132 | 1.51E-18 | 1.40E-16 | 31.27130731 |
| ADAMTSL3 | -1.476453228 | 0.280879455 | -16.3586685 | 5.12E-47 | 1.11E-43 | 96.15145077 |
| HIF3A | -1.477714473 | 0.42384126 | -9.710667233 | 2.87E-20 | 3.35E-18 | 35.17865892 |
| AC053503.12 | -1.480231254 | 0.262302899 | -11.45631582 | 1.14E-26 | 2.68E-24 | 49.74009305 |
| ISLR | -1.480652147 | 3.696790897 | -3.151759926 | 0.001737622 | 0.011899427 | -2.397000389 |
| ACACB | -1.481067711 | 0.855106554 | -11.85577428 | 3.26E-28 | 9.06E-26 | 53.256524 |
| WISP2 | -1.481726131 | 0.877956136 | -6.170394601 | 1.59E-09 | 4.18E-08 | 10.89389664 |
| PDZD4 | -1.486369918 | 0.635792143 | -9.2334579 | 1.25E-18 | 1.18E-16 | 31.45751538 |
| KLHL41 | -1.486503165 | 0.287490191 | -14.69881524 | 7.59E-40 | 6.71E-37 | 79.78528626 |
| CSGALNACT1 | -1.487885121 | 1.38189804 | -10.17084161 | 6.76E-22 | 9.65E-20 | 38.87885846 |
| BDKRB1 | -1.490628423 | 0.815194316 | -9.070235882 | 4.42E-18 | 3.80E-16 | 30.21321233 |
| SSC5D | -1.492274552 | 1.252908987 | -6.478981801 | 2.56E-10 | 7.67E-09 | 12.67320978 |
| SGK1 | -1.495461082 | 2.957349579 | -4.991648663 | 8.75E-07 | 1.38E-05 | 4.783533766 |
| BOC | -1.496135147 | 0.833421387 | -8.047716919 | 8.50E-15 | 5.04E-13 | 22.77192831 |
| SPEG | -1.496231051 | 0.755938487 | -8.571317827 | 1.91E-16 | 1.41E-14 | 26.50412243 |
| SOBP | -1.496614229 | 0.641381438 | -11.92238893 | 1.79E-28 | 5.23E-26 | 53.84886079 |
| TIPARP | -1.49676903 | 3.074627078 | -7.373350853 | 8.76E-13 | 3.82E-11 | 18.22325788 |
| OLFM1 | -1.49903917 | 0.636995178 | -9.320899621 | 6.31E-19 | 6.23E-17 | 32.1301834 |
| TPPP3 | -1.502168526 | 2.265657462 | -5.455339128 | 8.31E-08 | 1.59E-06 | 7.053833156 |
| TUBB6 | -1.503417533 | 3.844450744 | -4.063070339 | 5.77E-05 | 0.000602975 | 0.78410885 |
| NDN | -1.503689982 | 2.793453828 | -5.08116539 | 5.63E-07 | 9.23E-06 | 5.207975772 |
| CLIC4 | -1.505384154 | 4.538308506 | -5.042285129 | 6.82E-07 | 1.10E-05 | 5.022803826 |
| LHFP | -1.505924909 | 3.133126385 | -6.152708981 | 1.76E-09 | 4.60E-08 | 10.79410845 |
| NFIA | -1.513809192 | 1.880472378 | -8.674849832 | 8.84E-17 | 6.76E-15 | 27.26184629 |
| GAS7 | -1.514900608 | 1.284975857 | -7.283724672 | 1.59E-12 | 6.68E-11 | 17.64163446 |
| FGF2 | -1.515554206 | 0.639133855 | -10.1606489 | 7.35E-22 | 1.04E-19 | 38.7957598 |
| SLMAP | -1.516950838 | 2.358438847 | -9.776283697 | 1.69E-20 | 2.01E-18 | 35.69971975 |
| PDGFRA | -1.51829842 | 1.577570495 | -6.814844758 | 3.24E-11 | 1.11E-09 | 14.69024458 |
| RNF150 | -1.519331243 | 0.541755209 | -11.48157305 | 9.12E-27 | 2.20E-24 | 49.96058326 |
| RERG | -1.520644803 | 0.930532734 | -9.129537125 | 2.80E-18 | 2.46E-16 | 30.66356849 |
| SLC9A9 | -1.521010968 | 1.354599141 | -8.752468734 | 4.94E-17 | 3.84E-15 | 27.83407992 |
| CCL19 | -1.522513852 | 2.123429891 | -3.26881383 | 0.001167656 | 0.008475413 | -2.030948394 |
| ARMCX1 | -1.522785763 | 2.033896589 | -6.099590505 | 2.39E-09 | 6.12E-08 | 10.4958373 |
| PER2 | -1.52435437 | 1.748303689 | -10.99157727 | 6.58E-25 | 1.31E-22 | 45.7297068 |
| FBXL22 | -1.524681941 | 0.698146476 | -10.66388096 | 1.09E-23 | 1.85E-21 | 42.95746926 |
| PRIMA1 | -1.524799602 | 0.447085046 | -12.3749481 | 2.94E-30 | 1.06E-27 | 57.9156047 |
| S1PR1 | -1.526806246 | 2.167774781 | -7.123728849 | 4.52E-12 | 1.77E-10 | 16.6171458 |
| KCNE4 | -1.527551245 | 1.15546318 | -7.304449664 | 1.38E-12 | 5.86E-11 | 17.77563777 |
| GREM2 | -1.527863147 | 0.422224212 | -11.98826244 | 9.90E-29 | 2.99E-26 | 54.43622469 |
| PELI2 | -1.528652284 | 1.050529363 | -9.400018086 | 3.39E-19 | 3.46E-17 | 32.74241917 |
| TIMP2 | -1.529670018 | 4.714768907 | -4.151284209 | 4.00E-05 | 0.000434326 | 1.131784299 |
| RAB23 | -1.53299554 | 1.537892951 | -8.015555062 | 1.07E-14 | 6.22E-13 | 22.5482578 |
| HBA2 | -1.533351284 | 2.327896923 | -4.624654263 | 4.99E-06 | 6.68E-05 | 3.114050039 |
| TUBA1A | -1.534009914 | 4.725340749 | -4.725427846 | 3.13E-06 | 4.37E-05 | 3.561077609 |
| CLEC3A | -1.534323587 | 0.268505485 | -9.864791627 | 8.27E-21 | 1.03E-18 | 36.40606256 |

| | | | | | | |
|---|---|---|---|---|---|---|
| TSC22D1 | -1.535839192 | 4.302640605 | -8.176446283 | 3.39E-15 | 2.11E-13 | 23.67373892 |
| NNAT | -1.536618983 | 1.565696729 | -3.583844338 | 0.00037776 | 0.003198419 | -0.982996656 |
| OLFML1 | -1.538115334 | 1.144240383 | -9.107300591 | 3.32E-18 | 2.89E-16 | 30.49446461 |
| AGTR1 | -1.53845666 | 0.433107948 | -11.09955471 | 2.59E-25 | 5.35E-23 | 46.65342316 |
| MYC | -1.538500587 | 4.622503242 | -4.584638766 | 5.99E-06 | 7.88E-05 | 2.93895808 |
| C8orf88 | -1.540585646 | 1.00484394 | -6.84483194 | 2.68E-11 | 9.40E-10 | 14.87432835 |
| EVA1C | -1.545166354 | 1.717512427 | -6.74333508 | 5.06E-11 | 1.70E-09 | 14.25388593 |
| ABCA8 | -1.549129205 | 0.400045074 | -13.76766288 | 6.28E-36 | 3.90E-33 | 70.84683321 |
| APBB1 | -1.549395913 | 1.562443582 | -7.318961202 | 1.26E-12 | 5.35E-11 | 17.86964209 |
| HAS1 | -1.550001918 | 0.573266788 | -7.597870481 | 1.93E-13 | 9.26E-12 | 19.70423703 |
| GNAL | -1.552459393 | 0.77054295 | -11.45181078 | 1.19E-26 | 2.78E-24 | 49.70079182 |
| SELM | -1.552668647 | 3.589351097 | -4.947043022 | 1.09E-06 | 1.69E-05 | 4.574539908 |
| NACC2 | -1.555068998 | 2.038245189 | -9.423517134 | 2.82E-19 | 2.91E-17 | 32.92491262 |
| EPHA7 | -1.556524889 | 0.575821198 | -9.818134004 | 1.21E-20 | 1.47E-18 | 36.03320996 |
| F3 | -1.557639613 | 3.590496594 | -3.557922519 | 0.000415855 | 0.003481574 | -1.072660307 |
| TWIST2 | -1.558427388 | 0.976697984 | -7.372343081 | 8.82E-13 | 3.84E-11 | 18.21668742 |
| FBLN1 | -1.562642664 | 6.002241507 | -3.709196715 | 0.000235461 | 0.002112457 | -0.540775173 |
| MXRA7 | -1.567294841 | 2.611393832 | -8.038838673 | 9.05E-15 | 5.34E-13 | 22.71011851 |
| SLIT2 | -1.567771605 | 0.676708914 | -10.39270893 | 1.07E-22 | 1.63E-20 | 40.70008637 |
| CYP1B1 | -1.569428073 | 1.563559238 | -4.96411148 | 1.00E-06 | 1.57E-05 | 4.654314738 |
| SLC2A3 | -1.570367048 | 2.560478184 | -5.253999465 | 2.36E-07 | 4.16E-06 | 6.046299716 |
| CRYM | -1.571080809 | 0.728863227 | -8.062489025 | 7.65E-15 | 4.56E-13 | 22.8748818 |
| ADAMTS8 | -1.57337477 | 0.514010168 | -11.27159327 | 5.78E-26 | 1.27E-23 | 48.13533798 |
| FCER1A | -1.576260388 | 0.929842086 | -7.901923714 | 2.37E-14 | 1.31E-12 | 21.7633052 |
| EDNRB | -1.576332771 | 1.434129445 | -7.523914592 | 3.19E-13 | 1.47E-11 | 19.21265462 |
| HSPA2 | -1.576546375 | 1.973703887 | -6.906481669 | 1.82E-11 | 6.52E-10 | 15.25481035 |
| SELENBP1 | -1.58026853 | 3.008387236 | -4.541035442 | 7.30E-06 | 9.41E-05 | 2.749736485 |
| FAXDC2 | -1.583569319 | 0.877989286 | -12.13890743 | 2.53E-29 | 8.12E-27 | 55.78540468 |
| C2CD4B | -1.587761451 | 0.916476509 | -7.932316149 | 1.92E-14 | 1.07E-12 | 21.97244001 |
| AKR1B10 | -1.589288772 | 2.543319077 | -2.645487052 | 0.008459427 | 0.044706787 | -3.832622245 |
| SLC25A23 | -1.594020011 | 2.859503638 | -7.212077134 | 2.54E-12 | 1.03E-10 | 17.18066138 |
| PDE5A | -1.596610078 | 1.172286556 | -9.963287349 | 3.71E-21 | 4.82E-19 | 37.1967864 |
| CD248 | -1.596884066 | 3.606916612 | -5.135397347 | 4.29E-07 | 7.20E-06 | 5.468362905 |
| BMP5 | -1.60059174 | 0.546135195 | -9.628018259 | 5.56E-20 | 6.25E-18 | 34.52552114 |
| SH3BGR | -1.600777819 | 1.685557842 | -9.451591987 | 2.26E-19 | 2.35E-17 | 33.14333149 |
| NR2F1 | -1.608578521 | 1.902874759 | -6.93923598 | 1.48E-11 | 5.37E-10 | 15.45806591 |
| EPHA3 | -1.60925307 | 0.855481077 | -9.964214382 | 3.69E-21 | 4.79E-19 | 37.2042518 |
| PLP1 | -1.610029755 | 0.223538499 | -19.86852216 | 1.25E-62 | 2.44E-58 | 131.7860579 |
| RBM24 | -1.610895926 | 0.396071612 | -13.17732264 | 1.71E-33 | 8.82E-31 | 65.29474954 |
| BAG2 | -1.612202129 | 1.533847474 | -8.323049602 | 1.18E-15 | 7.71E-14 | 24.71340135 |
| ALDH1B1 | -1.613372473 | 3.865094114 | -6.563292957 | 1.53E-10 | 4.81E-09 | 13.17175288 |
| AF131217.1 | -1.618415685 | 0.271219367 | -16.38861403 | 3.79E-47 | 8.50E-44 | 96.45075703 |
| FBXL7 | -1.618846993 | 1.120736037 | -9.921341728 | 5.23E-21 | 6.65E-19 | 36.85944908 |
| GLP2R | -1.618948661 | 0.207627424 | -16.87178035 | 2.86E-49 | 7.94E-46 | 101.295906 |
| CYTL1 | -1.619196228 | 0.915241086 | -8.03894293 | 9.04E-15 | 5.34E-13 | 22.71084405 |
| CYGB | -1.62082242 | 2.750806958 | -6.85103485 | 2.58E-11 | 9.08E-10 | 14.91248724 |
| MIR22HG | -1.626091229 | 2.805375195 | -8.15200857 | 4.04E-15 | 2.50E-13 | 23.50173809 |
| SEPP1 | -1.626906744 | 2.186728956 | -5.462919388 | 7.98E-08 | 1.54E-06 | 7.09241029 |
| PTGFR | -1.628687107 | 0.496516921 | -11.72978762 | 1.01E-27 | 2.64E-25 | 52.14081472 |
| SVEP1 | -1.630098244 | 0.735874969 | -13.35282488 | 3.26E-34 | 1.79E-31 | 66.93512609 |
| AREG | -1.632998571 | 3.097662334 | -3.351770637 | 0.000874691 | 0.006614501 | -1.76384415 |
| C1S | -1.634215104 | 4.643976595 | -3.935320038 | 9.70E-05 | 0.000961604 | 0.292872656 |
| ADAMTS9-AS1 | -1.634332876 | 0.214214356 | -17.78790956 | 2.52E-53 | 8.64E-50 | 110.5527397 |
| VSTM4 | -1.635279235 | 1.031016231 | -11.04085122 | 4.30E-25 | 8.72E-23 | 46.15061329 |
| GAS6 | -1.637115389 | 3.542345366 | -5.051924063 | 6.50E-07 | 1.06E-05 | 5.06859292 |
| GPIHBP1 | -1.637483983 | 0.646238923 | -11.37611263 | 2.31E-26 | 5.29E-24 | 49.04163502 |
| DUSP2 | -1.643578932 | 3.485791784 | -4.299743433 | 2.12E-05 | 0.000245276 | 1.732412475 |
| MIR568 | -1.644933755 | 1.730856159 | -8.084855504 | 6.53E-15 | 3.93E-13 | 23.03102741 |
| FIGF | -1.645515924 | 0.478661133 | -12.63054151 | 2.80E-31 | 1.14E-28 | 60.24375703 |
| GPR183 | -1.648317519 | 2.198562897 | -5.852566396 | 9.68E-09 | 2.21E-07 | 9.137410363 |
| GNG11 | -1.650558607 | 2.586808258 | -7.755478808 | 6.57E-14 | 3.35E-12 | 20.76399499 |
| ITPR1 | -1.650656047 | 1.334821938 | -9.593087389 | 7.35E-20 | 8.17E-18 | 34.25055218 |
| GPR133 | -1.651124713 | 0.353985794 | -14.08550408 | 2.95E-37 | 2.08E-34 | 73.87458184 |
| SDC2 | -1.653843485 | 3.026748752 | -6.002483753 | 4.16E-09 | 1.02E-07 | 9.956179185 |
| NR3C2 | -1.655931689 | 0.640870592 | -11.64118105 | 2.22E-27 | 5.58E-25 | 51.35976717 |
| SERPINF1 | -1.661559442 | 4.311942621 | -4.455907853 | 1.07E-05 | 0.000132632 | 2.385049109 |
| COLEC12 | -1.667710173 | 1.163920877 | -7.010093595 | 9.39E-12 | 3.52E-10 | 15.90038366 |
| ID4 | -1.667919495 | 2.777286259 | -4.73944168 | 2.93E-06 | 4.12E-05 | 3.62392971 |
| TGFB1I1 | -1.670721933 | 2.573602537 | -6.951362304 | 1.37E-11 | 5.01E-10 | 15.53350944 |
| KCNH2 | -1.671690508 | 0.689777503 | -8.519516614 | 2.80E-16 | 2.03E-14 | 26.12740613 |

| | | | | | | |
|---|---|---|---|---|---|---|
| PRKAR2B | -1.673269465 | 1.507279114 | -7.578824782 | 2.20E-13 | 1.04E-11 | 19.57729085 |
| KCNMA1 | -1.675615838 | 0.541135908 | -12.04436121 | 5.96E-29 | 1.83E-26 | 54.93768616 |
| GPRASP1 | -1.677659033 | 0.852826785 | -12.3420885 | 3.97E-30 | 1.42E-27 | 57.61789443 |
| FNBP1 | -1.678461559 | 2.82171641 | -9.188975513 | 1.77E-18 | 1.62E-16 | 31.11693922 |
| DMD | -1.679544696 | 0.61608569 | -11.06187234 | 3.59E-25 | 7.34E-23 | 46.33049572 |
| DTNA | -1.683060979 | 0.580721308 | -11.1988107 | 1.09E-25 | 2.34E-23 | 47.50689044 |
| FZD7 | -1.685226222 | 2.62821969 | -7.732595239 | 7.69E-14 | 3.88E-12 | 20.6091079 |
| MYZAP | -1.694252929 | 1.886172524 | -7.145061146 | 3.93E-12 | 1.55E-10 | 16.75271178 |
| MAP1A | -1.696707563 | 1.046560316 | -8.899975783 | 1.62E-17 | 1.34E-15 | 28.93125188 |
| KCND3 | -1.700027721 | 0.716670711 | -10.97010847 | 7.92E-25 | 1.56E-22 | 45.54664349 |
| LAMA2 | -1.70078335 | 1.234985968 | -9.645313574 | 4.85E-20 | 5.47E-18 | 34.66190337 |
| PKIG | -1.702795748 | 3.841445449 | -7.768856803 | 5.99E-14 | 3.08E-12 | 20.85470296 |
| CELF2 | -1.704081353 | 1.074522051 | -10.04858661 | 1.85E-21 | 2.49E-19 | 37.88549707 |
| ALDH1A1 | -1.706801146 | 2.509464403 | -3.964051647 | 8.64E-05 | 0.00086634 | 0.402084825 |
| CD34 | -1.713208265 | 2.266245453 | -9.454823564 | 2.20E-19 | 2.30E-17 | 33.16849993 |
| HBB | -1.717345395 | 2.945370253 | -4.450654434 | 1.09E-05 | 0.000135338 | 2.362749082 |
| MIR221 | -1.717380841 | 0.756562513 | -9.56810689 | 8.97E-20 | 9.83E-18 | 34.05430443 |
| PLCB4 | -1.71769638 | 0.800586567 | -10.14626346 | 8.28E-22 | 1.17E-19 | 38.67856502 |
| HAAO | -1.717762995 | 1.352565471 | -10.1724485 | 6.67E-22 | 9.54E-20 | 38.89196365 |
| PDE2A | -1.720118323 | 0.921802861 | -12.10432589 | 3.46E-29 | 1.08E-26 | 55.47496738 |
| C16orf45 | -1.726921745 | 1.919048746 | -7.731088803 | 7.77E-14 | 3.91E-12 | 20.59892369 |
| MBNL1-AS1 | -1.728120035 | 0.654994286 | -12.23004999 | 1.11E-29 | 3.75E-27 | 56.60562732 |
| TMEM252 | -1.728763065 | 0.247933264 | -15.01354794 | 3.44E-41 | 3.59E-38 | 82.85027625 |
| CRTAC1 | -1.731161913 | 1.566514774 | -3.485071047 | 0.000543084 | 0.00438515 | -1.321367648 |
| CX3CL1 | -1.732454295 | 2.323918892 | -5.42641708 | 9.67E-08 | 1.83E-06 | 6.907072022 |
| NFIX | -1.733658229 | 2.667446675 | -7.993162709 | 1.25E-14 | 7.15E-13 | 22.39291905 |
| BDKRB2 | -1.736515406 | 1.608348741 | -8.861267969 | 2.18E-17 | 1.77E-15 | 28.64211864 |
| TNS2 | -1.742268417 | 2.355378373 | -10.0135702 | 2.46E-21 | 3.26E-19 | 37.60233461 |
| ARHGEF25 | -1.743114146 | 2.157941625 | -7.519527288 | 3.29E-13 | 1.51E-11 | 19.18360764 |
| CHRM2 | -1.743524554 | 0.299779205 | -13.82617013 | 3.58E-36 | 2.32E-33 | 71.40223947 |
| SVIL | -1.745110945 | 3.281171809 | -7.332090421 | 1.15E-12 | 4.93E-11 | 17.95481648 |
| WBSCR17 | -1.745575488 | 0.531567147 | -11.17908047 | 1.30E-25 | 2.75E-23 | 47.33690783 |
| LPP | -1.748495864 | 2.510201133 | -9.396499105 | 3.49E-19 | 3.54E-17 | 32.71511643 |
| TSPAN18 | -1.752680461 | 1.499515674 | -8.227160524 | 2.36E-15 | 1.50E-13 | 24.03187553 |
| ZEB1 | -1.754440876 | 1.44102647 | -9.197907554 | 1.65E-18 | 1.52E-16 | 31.18523884 |
| DUSP5 | -1.754973637 | 4.484008914 | -5.566954069 | 4.60E-08 | 9.28E-07 | 7.62654682 |
| F13A1 | -1.757569572 | 1.979011701 | -5.364251358 | 1.34E-07 | 2.47E-06 | 6.593920084 |
| CAV1 | -1.758511176 | 4.501115806 | -4.473541129 | 9.89E-06 | 0.000123597 | 2.460074946 |
| SOX17 | -1.764658111 | 0.980941272 | -11.96305791 | 1.24E-28 | 3.70E-26 | 54.2112987 |
| GATA5 | -1.765014634 | 0.447177296 | -10.91328124 | 1.29E-24 | 2.48E-22 | 45.06304427 |
| XPNPEP2 | -1.766802788 | 0.378513424 | -12.59075609 | 4.04E-31 | 1.62E-28 | 59.87992988 |
| JAM2 | -1.76758352 | 0.964060482 | -12.28534613 | 6.68E-30 | 2.33E-27 | 57.10468164 |
| LMO3 | -1.768248061 | 0.600464877 | -9.79124258 | 1.50E-20 | 1.80E-18 | 35.8188187 |
| AF001548.6 | -1.768884753 | 0.294093354 | -15.20957486 | 4.97E-42 | 5.80E-39 | 84.76922257 |
| DNAJB5 | -1.774746733 | 1.441042191 | -9.197995141 | 1.65E-18 | 1.52E-16 | 31.1859088 |
| CGNL1 | -1.77497991 | 0.837473567 | -11.82124422 | 4.44E-28 | 1.21E-25 | 52.95013643 |
| SELE | -1.775002733 | 1.178582711 | -6.386134005 | 4.46E-10 | 1.28E-08 | 12.13030896 |
| GNG7 | -1.775292507 | 0.809112314 | -12.47762393 | 1.15E-30 | 4.29E-28 | 58.84822798 |
| C1QTNF7 | -1.775591542 | 0.436506918 | -18.123253 | 8.09E-55 | 4.11E-51 | 113.9594529 |
| TMEM35 | -1.785662386 | 0.723564949 | -8.286011462 | 1.54E-15 | 9.95E-14 | 24.44948006 |
| BCHE | -1.786717383 | 0.608677102 | -9.233344472 | 1.25E-18 | 1.18E-16 | 31.45664553 |
| RCAN1 | -1.791612309 | 2.723540718 | -7.975086092 | 1.42E-14 | 8.06E-13 | 22.267753 |
| ANTXR2 | -1.794700101 | 2.269641579 | -7.899625389 | 2.41E-14 | 1.33E-12 | 21.74751433 |
| WFDC1 | -1.796410813 | 1.129619173 | -10.51324978 | 3.89E-23 | 6.18E-21 | 41.69933576 |
| ALDH2 | -1.799694162 | 2.31336167 | -6.397603962 | 4.17E-10 | 1.20E-08 | 12.19702721 |
| COL4A6 | -1.805802148 | 1.899890143 | -5.963265635 | 5.20E-09 | 1.26E-07 | 9.740298869 |
| RP11-498E2.9 | -1.807502308 | 0.585732244 | -11.19391241 | 1.14E-25 | 2.43E-23 | 47.46467483 |
| CCL21 | -1.807994316 | 2.42634846 | -3.690114109 | 0.000253249 | 0.002254723 | -0.609015848 |
| TNFAIP8L3 | -1.810004959 | 1.210317791 | -8.79851328 | 3.50E-17 | 2.79E-15 | 28.17520617 |
| CMA1 | -1.811210171 | 0.50258157 | -9.257606074 | 1.04E-18 | 9.92E-17 | 31.64286176 |
| ANGPTL1 | -1.815484381 | 0.617725706 | -9.352124217 | 4.94E-19 | 4.93E-17 | 32.3714 |
| CCND2 | -1.82354804 | 2.330296066 | -6.357873244 | 5.28E-10 | 1.49E-08 | 11.96634364 |
| LAMC3 | -1.823908773 | 1.313417563 | -9.622655279 | 5.81E-20 | 6.51E-18 | 34.48326319 |
| TSPAN2 | -1.824276774 | 1.695402818 | -6.960342538 | 1.29E-11 | 4.75E-10 | 15.58944716 |
| RASL11A | -1.826066374 | 1.96481955 | -7.642748491 | 1.42E-13 | 6.98E-12 | 20.00432076 |
| LRRN4CL | -1.82757627 | 0.904841713 | -11.51650612 | 6.70E-27 | 1.62E-24 | 50.26596037 |
| EGR2 | -1.827998466 | 1.558376128 | -7.765049539 | 6.15E-14 | 3.16E-12 | 20.8288763 |
| MIR143 | -1.831139348 | 0.338727636 | -12.83346279 | 4.26E-32 | 1.87E-29 | 62.10739044 |
| SPRY1 | -1.834296486 | 2.927217963 | -8.602806994 | 1.51E-16 | 1.14E-14 | 26.73390811 |
| REEP1 | -1.835575692 | 0.578040647 | -10.31651816 | 2.02E-22 | 3.00E-20 | 40.07201102 |

| | | | | | | |
|---|---|---|---|---|---|---|
| SHH | -1.835887795 | 1.409627653 | -4.53619425 | 7.46E-06 | 9.59E-05 | 2.728828715 |
| BHMT2 | -1.839202077 | 0.455626707 | -15.38781797 | 8.49E-43 | 1.10E-39 | 86.52034406 |
| RP11-1100L3.8 | -1.840712875 | 0.788182549 | -11.9830189 | 1.04E-28 | 3.11E-26 | 54.389412 |
| MGLL | -1.841880735 | 2.050631366 | -8.014670719 | 1.07E-14 | 6.24E-13 | 22.54211692 |
| PCP4L1 | -1.84303444 | 2.865127303 | -3.675763279 | 0.000267452 | 0.002362081 | -0.660117918 |
| BIN1 | -1.844772136 | 2.081028854 | -7.203346789 | 2.69E-12 | 1.09E-10 | 17.12473424 |
| RBFOX3 | -1.851243352 | 0.25555757 | -14.79202656 | 3.04E-40 | 2.78E-37 | 80.69088986 |
| GADD45B | -1.852147708 | 4.215020403 | -6.721304372 | 5.80E-11 | 1.93E-09 | 14.12019965 |
| DIXDC1 | -1.853053403 | 1.107196331 | -11.26621645 | 6.06E-26 | 1.32E-23 | 48.08883632 |
| NEGR1 | -1.863492703 | 0.462376421 | -14.86775266 | 1.45E-40 | 1.38E-37 | 81.42794583 |
| PTRF | -1.863856828 | 5.501758978 | -5.418642657 | 1.01E-07 | 1.90E-06 | 6.867737453 |
| MEDAG | -1.86399296 | 1.574065193 | -6.61554386 | 1.11E-10 | 3.57E-09 | 13.48335629 |
| PRAC1 | -1.86851045 | 0.461578616 | -7.861476965 | 3.15E-14 | 1.69E-12 | 21.48591086 |
| TCEAL7 | -1.869418731 | 1.044187978 | -10.60027347 | 1.87E-23 | 3.08E-21 | 42.42492656 |
| COL6A2 | -1.871921087 | 6.037347581 | -4.448810106 | 1.10E-05 | 0.000136251 | 2.354925849 |
| IER3 | -1.873240335 | 5.496807283 | -6.049436021 | 3.19E-09 | 7.99E-08 | 10.21620106 |
| THBS1 | -1.877796517 | 4.587488573 | -5.320232466 | 1.68E-07 | 3.05E-06 | 6.374085331 |
| PGM5P4 | -1.879175864 | 0.25395465 | -17.98450789 | 3.36E-54 | 1.40E-50 | 112.5489509 |
| JUN | -1.88014467 | 5.779068383 | -6.89031123 | 2.01E-11 | 7.18E-10 | 15.15474807 |
| ITGA5 | -1.883469239 | 3.868124552 | -5.901036245 | 7.38E-09 | 1.72E-07 | 9.400207426 |
| GPR124 | -1.886072383 | 2.097472971 | -7.833607942 | 3.82E-14 | 2.03E-12 | 21.29539512 |
| PHYHIP | -1.887515397 | 0.806288799 | -10.44309069 | 7.01E-23 | 1.09E-20 | 41.11691073 |
| pk | -1.8897221 | 2.046856301 | -9.34585549 | 5.19E-19 | 5.16E-17 | 32.32293018 |
| CXCL2 | -1.890102495 | 1.845845774 | -5.512563848 | 6.14E-08 | 1.21E-06 | 7.346207203 |
| PDZRN4 | -1.890225408 | 0.360403947 | -13.16372792 | 1.94E-33 | 9.76E-31 | 65.16805792 |
| MT1A | -1.895385679 | 1.117256522 | -6.26967291 | 8.89E-10 | 2.43E-08 | 11.45848594 |
| HSD17B6 | -1.899065844 | 1.459646192 | -8.589276772 | 1.67E-16 | 1.25E-14 | 26.63510153 |
| MIR27A | -1.901641499 | 0.624796444 | -10.04720789 | 1.87E-21 | 2.52E-19 | 37.87433643 |
| FGF7 | -1.905699898 | 1.022053814 | -8.198989821 | 2.89E-15 | 1.81E-13 | 23.83273971 |
| VIT | -1.911949918 | 0.283486837 | -14.99858428 | 3.99E-41 | 4.09E-38 | 82.70410034 |
| TP53INP2 | -1.913683639 | 3.162632489 | -9.158086025 | 2.24E-18 | 2.01E-16 | 30.88108173 |
| C5orf66-AS1 | -1.919531115 | 0.600692018 | -11.91222798 | 1.96E-28 | 5.65E-26 | 53.75840299 |
| CBX7 | -1.919953426 | 1.984483496 | -11.57648169 | 3.94E-27 | 9.79E-25 | 50.79138063 |
| DDR2 | -1.925833864 | 1.348478324 | -8.894690627 | 1.69E-17 | 1.39E-15 | 28.89172278 |
| DPYSL3 | -1.927239928 | 3.480657176 | -5.236607756 | 2.58E-07 | 4.50E-06 | 5.960825653 |
| KIAA1644 | -1.927292844 | 0.535107916 | -12.41923505 | 1.96E-30 | 7.15E-28 | 58.31743216 |
| SLC25A25 | -1.929199254 | 2.264245158 | -11.22391903 | 8.76E-26 | 1.89E-23 | 47.72344217 |
| FILIP1 | -1.931800048 | 0.575426364 | -13.42023233 | 1.72E-34 | 9.84E-32 | 67.56751506 |
| SLC24A3 | -1.932668545 | 1.471414608 | -8.501119595 | 3.21E-16 | 2.29E-14 | 25.99400503 |
| TGFBR3 | -1.932881445 | 2.252171692 | -7.62667443 | 1.59E-13 | 7.75E-12 | 19.89668501 |
| PARM1 | -1.935945518 | 2.544089727 | -6.428998978 | 3.45E-10 | 1.01E-08 | 12.38014916 |
| RP11-867G23.10 | -1.945429601 | 0.393950846 | -17.41511992 | 1.13E-51 | 3.49E-48 | 106.7762519 |
| IGFBP6 | -1.955395858 | 3.852867662 | -4.832336215 | 1.89E-06 | 2.77E-05 | 4.044785891 |
| LYVE1 | -1.962193892 | 0.892023463 | -9.12241439 | 2.95E-18 | 2.59E-16 | 30.60937155 |
| CH25H | -1.966841049 | 1.350170096 | -8.815501485 | 3.08E-17 | 2.47E-15 | 28.30137746 |
| CLCA4 | -1.967838707 | 2.183791973 | -3.64316581 | 0.000302545 | 0.002627889 | -0.775500304 |
| FERMT2 | -1.968396105 | 1.995063309 | -8.494481716 | 3.37E-16 | 2.37E-14 | 25.94592235 |
| TSPAN7 | -1.969066628 | 1.631277791 | -6.405441117 | 3.98E-10 | 1.15E-08 | 12.24267094 |
| AOX1 | -1.970916129 | 0.566274481 | -12.54979399 | 5.90E-31 | 2.27E-28 | 59.50588702 |
| HBEGF | -1.971552407 | 3.198233024 | -6.927605519 | 1.59E-11 | 5.75E-10 | 15.38580586 |
| FGFR1 | -1.975894763 | 1.747387135 | -7.781341287 | 5.49E-14 | 2.85E-12 | 20.93945838 |
| DACT3 | -1.982891719 | 0.985876744 | -9.889343678 | 6.78E-21 | 8.51E-19 | 36.60270827 |
| MYOM1 | -1.986606098 | 0.536430646 | -14.39234998 | 1.51E-38 | 1.21E-35 | 76.82103413 |
| CLDN5 | -1.99083184 | 1.90090441 | -8.070607187 | 7.22E-15 | 4.32E-13 | 22.93151993 |
| APOLD1 | -1.991483729 | 2.026798949 | -9.847353261 | 9.53E-21 | 1.18E-18 | 36.26657855 |
| GATA6 | -1.994976245 | 1.185621318 | -10.53396948 | 3.26E-23 | 5.25E-21 | 41.87177713 |
| GYPC | -1.99632916 | 1.982988873 | -7.728139987 | 7.93E-14 | 3.98E-12 | 20.57899263 |
| PPAP2B | -2.000832892 | 3.18993857 | -10.01427813 | 2.45E-21 | 3.26E-19 | 37.60805329 |
| RP11-64B16.2 | -2.001158416 | 1.118636309 | -9.730642069 | 2.44E-20 | 2.87E-18 | 35.33704386 |
| CFL2 | -2.002779924 | 1.727311662 | -10.50972705 | 4.00E-23 | 6.35E-21 | 41.67003724 |
| HAND2 | -2.003634744 | 0.717658666 | -10.44023471 | 7.18E-23 | 1.11E-20 | 41.09325045 |
| RASD1 | -2.008117521 | 1.948210371 | -6.639474484 | 9.62E-11 | 3.12E-09 | 13.62673934 |
| RP11-166D19.1 | -2.014199086 | 0.92282187 | -11.02496797 | 4.93E-25 | 9.93E-23 | 46.01482207 |
| CPXM2 | -2.021947781 | 1.617596252 | -6.949530898 | 1.38E-11 | 5.06E-10 | 15.52210869 |
| PKDCC | -2.025541484 | 1.20075647 | -7.241602483 | 2.09E-12 | 8.66E-11 | 17.37019435 |
| TCEAL2 | -2.032359992 | 0.561161979 | -10.67030221 | 1.03E-23 | 1.76E-21 | 43.01133213 |
| RHOB | -2.036736291 | 6.411152885 | -9.119717616 | 3.02E-18 | 2.64E-16 | 30.58885918 |
| CRISPLD2 | -2.043640863 | 2.702193774 | -7.16841389 | 3.38E-12 | 1.35E-10 | 16.90148217 |
| JPH2 | -2.047990687 | 1.214440697 | -8.144511055 | 4.27E-15 | 2.62E-13 | 23.44904322 |
| ITGA7 | -2.051037022 | 1.719747574 | -8.172947967 | 3.48E-15 | 2.16E-13 | 23.64909362 |

| | | | | | | |
|---|---|---|---|---|---|---|
| PER1 | -2.064549508 | 2.780809073 | -9.414296208 | 3.03E-19 | 3.11E-17 | 32.85326748 |
| PLIN4 | -2.06623944 | 2.084709645 | -6.672541794 | 7.85E-11 | 2.57E-09 | 13.82555669 |
| GPX3 | -2.068451832 | 3.986582396 | -5.244243447 | 2.48E-07 | 4.35E-06 | 5.998321717 |
| KANK2 | -2.07809101 | 3.155976886 | -9.132459181 | 2.73E-18 | 2.41E-16 | 30.68581065 |
| SBSPON | -2.079329911 | 1.232373232 | -8.4174153 | 5.93E-16 | 4.03E-14 | 25.38963125 |
| PMP22 | -2.083610819 | 3.794368938 | -6.34756862 | 5.61E-10 | 1.58E-08 | 11.90670689 |
| RGS5 | -2.086365158 | 2.608243799 | -8.008965985 | 1.12E-14 | 6.48E-13 | 22.50251525 |
| IL33 | -2.08748132 | 1.861686034 | -6.012157003 | 3.94E-09 | 9.72E-08 | 10.00961011 |
| EFEMP1 | -2.087527447 | 2.80310667 | -4.728686663 | 3.08E-06 | 4.31E-05 | 3.575678476 |
| JAM3 | -2.091017622 | 1.879579584 | -9.642229301 | 4.97E-20 | 5.59E-18 | 34.63757086 |
| MIR23A | -2.093510928 | 1.054323467 | -9.317176547 | 6.50E-19 | 6.40E-17 | 32.10145732 |
| TACR2 | -2.10124133 | 0.68028022 | -10.70425745 | 7.72E-24 | 1.35E-21 | 43.29646625 |
| SORBS2 | -2.103438033 | 0.966311779 | -11.38556472 | 2.13E-26 | 4.91E-24 | 49.12381457 |
| CPED1 | -2.103582731 | 0.754923005 | -12.9482466 | 1.46E-32 | 6.72E-30 | 63.16734069 |
| MFAP5 | -2.112260432 | 1.378388721 | -6.459067797 | 2.88E-10 | 8.56E-09 | 12.55622624 |
| CYBRD1 | -2.112621485 | 3.024697777 | -7.161600421 | 3.53E-12 | 1.41E-10 | 16.85803714 |
| FBLN5 | -2.112989347 | 2.14064888 | -8.637138254 | 1.17E-16 | 8.89E-15 | 26.9851071 |
| PID1 | -2.115477006 | 0.921364957 | -12.45607138 | 1.40E-30 | 5.17E-28 | 58.65216609 |
| ASB5 | -2.118267814 | 0.368946026 | -12.58030449 | 4.45E-31 | 1.75E-28 | 59.78443905 |
| C3 | -2.124913842 | 4.242049897 | -4.637050083 | 4.71E-06 | 6.34E-05 | 3.168568284 |
| HLF | -2.126780386 | 0.366242972 | -20.37633493 | 6.59E-65 | 1.92E-60 | 136.9884345 |
| ASB2 | -2.127044706 | 0.904152595 | -11.36608203 | 2.52E-26 | 5.71E-24 | 48.95446534 |
| TMOD1 | -2.127680446 | 0.778820708 | -11.90147081 | 2.16E-28 | 6.16E-26 | 53.66267908 |
| POPDC2 | -2.13395481 | 1.27187214 | -9.189843369 | 1.75E-18 | 1.61E-16 | 31.12357342 |
| ZCCHC24 | -2.13605866 | 2.127612103 | -9.268367101 | 9.52E-19 | 9.17E-17 | 31.7255601 |
| CADM3 | -2.139028829 | 0.522596112 | -11.07419206 | 3.22E-25 | 6.62E-23 | 46.43600588 |
| PDZRN3 | -2.140774777 | 1.252765435 | -9.691030502 | 3.36E-20 | 3.88E-18 | 35.02315648 |
| ANXA6 | -2.146872095 | 3.63810078 | -6.749726964 | 4.86E-11 | 1.64E-09 | 14.29273903 |
| ELN | -2.164220606 | 2.23844533 | -6.448337107 | 3.07E-10 | 9.09E-09 | 12.49331201 |
| PYGM | -2.166732518 | 0.470179857 | -15.795291 | 1.46E-44 | 2.30E-41 | 90.5444443 |
| CPA3 | -2.173386153 | 1.793262407 | -7.114753149 | 4.79E-12 | 1.87E-10 | 16.56020074 |
| CALD1 | -2.174035147 | 4.016863835 | -6.709419667 | 6.25E-11 | 2.07E-09 | 14.04822775 |
| CNTN1 | -2.178001288 | 0.993298888 | -8.247607999 | 2.03E-15 | 1.30E-13 | 24.17672636 |
| NCAM1 | -2.182370438 | 0.554442921 | -11.4584507 | 1.12E-26 | 2.66E-24 | 49.75872028 |
| RAI2 | -2.185204002 | 1.490344501 | -10.628204 | 1.47E-23 | 2.47E-21 | 42.6585433 |
| EMILIN1 | -2.192734723 | 3.772996198 | -5.700429049 | 2.24E-08 | 4.79E-07 | 8.324553788 |
| NEXN | -2.196529536 | 1.645976431 | -9.2192718 | 1.40E-18 | 1.30E-16 | 31.34878167 |
| AC053503.6 | -2.202482729 | 0.609901017 | -9.739128258 | 2.28E-20 | 2.69E-18 | 35.40439522 |
| GSN | -2.20461669 | 5.018035452 | -9.676038788 | 3.79E-20 | 4.31E-18 | 34.9045726 |
| AKAP12 | -2.20477142 | 2.151661677 | -7.919202174 | 2.10E-14 | 1.17E-12 | 21.88212777 |
| ABI3BP | -2.207883224 | 0.877385515 | -12.11669904 | 3.10E-29 | 9.83E-27 | 55.58599152 |
| ADAM33 | -2.207952691 | 0.833717278 | -12.10142789 | 3.56E-29 | 1.10E-26 | 55.4489716 |
| C8orf4 | -2.21238281 | 4.765209601 | -5.371629733 | 1.29E-07 | 2.38E-06 | 6.630923185 |
| HPSE2 | -2.219153273 | 0.61649783 | -10.83056608 | 2.63E-24 | 4.82E-22 | 44.36165941 |
| SOCS3 | -2.224100566 | 5.181313989 | -7.085092614 | 5.80E-12 | 2.24E-10 | 16.37242555 |
| PRUNE2 | -2.225596635 | 0.706670001 | -10.41920081 | 8.56E-23 | 1.32E-20 | 40.91911356 |
| CSF3 | -2.22826826 | 1.007261988 | -6.629639104 | 1.02E-10 | 3.30E-09 | 13.56775867 |
| OLFML3 | -2.236093351 | 2.842404557 | -6.395525686 | 4.22E-10 | 1.21E-08 | 12.18493102 |
| CTSG | -2.236896665 | 0.903235711 | -8.90087102 | 1.61E-17 | 1.33E-15 | 28.93794919 |
| PODN | -2.237888906 | 2.222201131 | -7.0868485 | 5.73E-12 | 2.22E-10 | 16.38352453 |
| CLIP3 | -2.237926796 | 2.032015302 | -8.351125076 | 9.61E-16 | 6.36E-14 | 24.91402109 |
| BTG2 | -2.241995518 | 6.173827269 | -6.847574481 | 2.64E-11 | 9.27E-10 | 14.89119642 |
| ADCY5 | -2.24241971 | 0.794955369 | -11.65247071 | 2.01E-27 | 5.14E-25 | 51.45911445 |
| CCL2 | -2.244777044 | 3.008501953 | -6.433126921 | 3.37E-10 | 9.86E-09 | 12.40428161 |
| TPPP | -2.244921566 | 0.807196272 | -13.89830566 | 1.79E-36 | 1.19E-33 | 72.08823448 |
| MAP1B | -2.249607643 | 1.591578006 | -9.069491363 | 4.44E-18 | 3.82E-16 | 30.20757073 |
| MAMDC2 | -2.254486405 | 0.891900858 | -10.81512924 | 3.00E-24 | 5.47E-22 | 44.23109586 |
| KLF9 | -2.258392868 | 2.421385235 | -9.709234037 | 2.90E-20 | 3.38E-18 | 35.16730273 |
| FILIP1L | -2.260408763 | 2.440151189 | -8.543721196 | 2.34E-16 | 1.71E-14 | 26.30322966 |
| MYOC | -2.273261799 | 0.243971329 | -17.23407667 | 7.18E-51 | 2.10E-47 | 104.9468013 |
| PAMR1 | -2.277227756 | 1.532415072 | -11.77980686 | 6.44E-28 | 1.72E-25 | 52.58305357 |
| SMTN | -2.279897934 | 3.158266696 | -10.83421705 | 2.55E-24 | 4.72E-22 | 44.39255438 |
| F10 | -2.289459945 | 0.638571335 | -17.84910695 | 1.34E-53 | 4.91E-50 | 111.1738038 |
| CCDC69 | -2.289602446 | 2.791935273 | -9.921307208 | 5.23E-21 | 6.65E-19 | 36.85917182 |
| EMP1 | -2.291059708 | 3.809265871 | -7.57157911 | 2.31E-13 | 1.09E-11 | 19.52905958 |
| MRVI1 | -2.310857091 | 1.680477915 | -9.780543178 | 1.64E-20 | 1.95E-18 | 35.733621 |
| FAM46B | -2.313887645 | 1.662248083 | -7.834986534 | 3.78E-14 | 2.01E-12 | 21.30480746 |
| KLF4 | -2.318664707 | 3.369327736 | -8.643547283 | 1.12E-16 | 8.49E-15 | 27.03207906 |
| CXCL12 | -2.322239472 | 2.004652621 | -7.515388134 | 3.38E-13 | 1.55E-11 | 19.15621545 |
| PTGS2 | -2.324009766 | 2.99189498 | -5.992872283 | 4.40E-09 | 1.07E-07 | 9.903161356 |

| | | | | | | |
|---|---|---|---|---|---|---|
| TPSB2 | -2.325203293 | 2.126995425 | -7.263348537 | 1.81E-12 | 7.60E-11 | 17.51017536 |
| AP000892.6 | -2.327660258 | 0.999016837 | -10.66604505 | 1.07E-23 | 1.82E-21 | 42.97562001 |
| SOD3 | -2.331226439 | 3.313680406 | -6.32390419 | 6.46E-10 | 1.80E-08 | 11.77005456 |
| COX7A1 | -2.331806638 | 2.407611715 | -9.477038358 | 1.85E-19 | 1.96E-17 | 33.34166645 |
| LTBP4 | -2.353800523 | 3.186145628 | -10.09341656 | 1.28E-21 | 1.77E-19 | 38.24890267 |
| PALLD | -2.35669046 | 3.551663209 | -8.13267585 | 4.64E-15 | 2.84E-13 | 23.36593367 |
| RASL12 | -2.362982448 | 1.763676177 | -9.952417059 | 4.06E-21 | 5.24E-19 | 37.10927998 |
| APOD | -2.365286552 | 2.952427835 | -4.875755076 | 1.53E-06 | 2.31E-05 | 4.244002668 |
| ITGA8 | -2.384120245 | 0.969941129 | -14.68843428 | 8.40E-40 | 7.32E-37 | 79.68454258 |
| FAM129A | -2.393744627 | 2.430824834 | -8.401757129 | 6.65E-16 | 4.49E-14 | 25.27704675 |
| PSD | -2.39787515 | 1.11697337 | -10.89807016 | 1.47E-24 | 2.81E-22 | 44.93383652 |
| TPSAB1 | -2.404470802 | 2.094988444 | -7.64879201 | 1.37E-13 | 6.72E-12 | 20.04483401 |
| PPP1R14A | -2.405146855 | 2.392908005 | -8.730665427 | 5.82E-17 | 4.52E-15 | 27.67298048 |
| TPM1 | -2.411259432 | 3.745766519 | -8.549395443 | 2.25E-16 | 1.65E-14 | 26.34449867 |
| CSRP1 | -2.414268026 | 4.493773938 | -10.68927938 | 8.77E-24 | 1.52E-21 | 43.17062596 |
| AQP1 | -2.415445977 | 4.283776457 | -9.419228918 | 2.92E-19 | 3.00E-17 | 32.89158816 |
| CACNA1H | -2.415599528 | 1.381734734 | -10.35410461 | 1.48E-22 | 2.22E-20 | 40.38150947 |
| FLNA | -2.424352481 | 6.125495403 | -6.983599052 | 1.11E-11 | 4.14E-10 | 15.73457815 |
| CKB | -2.425380208 | 4.315421645 | -6.649186164 | 9.06E-11 | 2.94E-09 | 13.68504781 |
| MYOCD | -2.42674777 | 0.518216338 | -15.40714088 | 7.01E-43 | 9.51E-40 | 86.7105255 |
| RP11-394O4.5 | -2.430570157 | 1.541642578 | -8.727119955 | 5.98E-17 | 4.62E-15 | 27.6468102 |
| CSRNP1 | -2.432388194 | 3.590373814 | -11.63874385 | 2.27E-27 | 5.68E-25 | 51.33832666 |
| LIMS2 | -2.433827837 | 1.620670498 | -10.86127246 | 2.02E-24 | 3.79E-22 | 44.62168407 |
| TCF21 | -2.442742989 | 0.757952224 | -16.68960113 | 1.81E-48 | 4.40E-45 | 99.46563888 |
| IGFBP5 | -2.454991598 | 4.443617462 | -5.545883858 | 5.14E-08 | 1.03E-06 | 7.517663736 |
| ITM2A | -2.457701984 | 2.026734754 | -9.235767248 | 1.23E-18 | 1.16E-16 | 31.4752266 |
| MATN2 | -2.459221527 | 2.34644755 | -8.226784084 | 2.36E-15 | 1.50E-13 | 24.02921125 |
| KCNMB1 | -2.479345363 | 1.037194575 | -11.15600335 | 1.58E-25 | 3.34E-23 | 47.1382973 |
| PLA2G2A | -2.480358798 | 3.458506688 | -4.21773581 | 3.02E-05 | 0.000336612 | 1.398232396 |
| NR4A2 | -2.483721982 | 2.152095713 | -10.68183726 | 9.34E-24 | 1.60E-21 | 43.10813761 |
| SLC2A4 | -2.48721707 | 0.548813195 | -14.61754399 | 1.68E-39 | 1.42E-36 | 78.99719351 |
| SGCA | -2.492966791 | 0.945895554 | -10.62031081 | 1.57E-23 | 2.63E-21 | 42.59248693 |
| MGP | -2.498432536 | 4.833066588 | -5.545730364 | 5.15E-08 | 1.03E-06 | 7.516871843 |
| A2M | -2.50050512 | 5.634064895 | -7.906970443 | 2.29E-14 | 1.27E-12 | 21.79799123 |
| MSRB3 | -2.504763509 | 1.975053136 | -8.790740377 | 3.71E-17 | 2.95E-15 | 28.1175329 |
| CILP | -2.50753217 | 1.155780939 | -7.471625341 | 4.54E-13 | 2.06E-11 | 18.86730741 |
| RAMP1 | -2.507662666 | 2.767365354 | -6.339349689 | 5.89E-10 | 1.65E-08 | 11.85919812 |
| FBLN2 | -2.520218987 | 3.107063289 | -6.845763526 | 2.67E-11 | 9.37E-10 | 14.88005749 |
| MYADM | -2.542202891 | 4.0891404 | -7.618814785 | 1.68E-13 | 8.17E-12 | 19.8441175 |
| CLU | -2.552931254 | 4.963201057 | -5.552568884 | 4.96E-08 | 9.96E-07 | 7.552170841 |
| CCDC80 | -2.554769649 | 2.186422579 | -7.459563724 | 4.92E-13 | 2.22E-11 | 18.78790782 |
| RBPMS2 | -2.555771355 | 1.795331301 | -9.097459212 | 3.58E-18 | 3.12E-16 | 30.41971157 |
| SPON1 | -2.560582828 | 2.129831422 | -7.77830553 | 5.61E-14 | 2.90E-12 | 20.91883966 |
| SLIT3 | -2.570855772 | 1.521416457 | -11.43944638 | 1.32E-26 | 3.08E-24 | 49.5929687 |
| FXYD6 | -2.573978789 | 1.443874368 | -9.454392703 | 2.21E-19 | 2.30E-17 | 33.16514393 |
| CHRDL1 | -2.595038705 | 0.801631432 | -10.52962182 | 3.39E-23 | 5.43E-21 | 41.83557691 |
| TNXB | -2.595091798 | 0.641530935 | -15.68462004 | 4.42E-44 | 6.46E-41 | 89.44872378 |
| ITIH5 | -2.602672469 | 1.134670191 | -13.47587981 | 1.01E-34 | 5.92E-32 | 68.09053953 |
| RCAN2 | -2.605735268 | 1.78202142 | -11.93673589 | 1.57E-28 | 4.62E-26 | 53.97664972 |
| PDLIM3 | -2.611715612 | 1.858852232 | -8.434854911 | 5.22E-16 | 3.57E-14 | 25.51520045 |
| HSPB7 | -2.615813984 | 1.223820653 | -8.59685501 | 1.58E-16 | 1.18E-14 | 26.69042935 |
| TPM2 | -2.629239209 | 5.169832558 | -7.012477305 | 9.25E-12 | 3.48E-10 | 15.91532555 |
| SFRP1 | -2.64356928 | 1.320898291 | -8.3857584 | 7.47E-16 | 5.01E-14 | 25.16216822 |
| PCOLCE2 | -2.648051128 | 0.927695085 | -11.901383 | 2.16E-28 | 6.16E-26 | 53.6618979 |
| ZFP36 | -2.677550711 | 6.73371758 | -9.290116382 | 8.03E-19 | 7.86E-17 | 31.89289713 |
| PPP1R12B | -2.677800954 | 1.722033845 | -12.08524441 | 4.12E-29 | 1.27E-26 | 55.30385733 |
| PTGDS | -2.720481208 | 2.922598799 | -6.558751873 | 1.58E-10 | 4.95E-09 | 13.14476675 |
| FENDRR | -2.732875915 | 0.945842099 | -13.77000697 | 6.14E-36 | 3.85E-33 | 70.86906838 |
| PLAC9 | -2.752440435 | 1.736754747 | -11.79817776 | 5.46E-28 | 1.48E-25 | 52.74571644 |
| COL14A1 | -2.753622993 | 1.975859525 | -10.11908252 | 1.04E-21 | 1.45E-19 | 38.45740432 |
| KLF2 | -2.754207794 | 2.820128864 | -11.10878252 | 2.39E-25 | 4.98E-23 | 46.73259483 |
| FOXF1 | -2.766225141 | 1.867072811 | -11.64116653 | 2.22E-27 | 5.58E-25 | 51.35963945 |
| MAOB | -2.768026078 | 1.518097937 | -9.186620168 | 1.80E-18 | 1.65E-16 | 31.09893625 |
| FABP4 | -2.773657811 | 3.1464568 | -4.363210275 | 1.61E-05 | 0.000191418 | 1.995085047 |
| MRGPRF | -2.848647245 | 2.026741366 | -9.813151304 | 1.26E-20 | 1.53E-18 | 35.99345757 |
| EGR3 | -2.84929627 | 1.276036919 | -12.90818605 | 2.13E-32 | 9.62E-30 | 62.7969431 |
| SRPX | -2.858002457 | 2.393900629 | -8.374578677 | 8.10E-16 | 5.42E-14 | 25.08198537 |
| CTC-296K1.4 | -2.85868433 | 0.624394719 | -13.53986901 | 5.52E-35 | 3.26E-32 | 68.69302951 |
| IL6 | -2.871593657 | 1.638363641 | -8.185180449 | 3.19E-15 | 1.99E-13 | 23.73530373 |
| P2RX1 | -2.873275217 | 0.878163007 | -11.75822578 | 7.81E-28 | 2.07E-25 | 52.39213023 |

| | | | | | | |
|---|---|---|---|---|---|---|
| FGL2 | -2.887808286 | 2.031529348 | -10.0547673 | 1.76E-21 | 2.37E-19 | 37.93554069 |
| ATF3 | -2.894557157 | 3.780238685 | -8.143566347 | 4.29E-15 | 2.64E-13 | 23.44240602 |
| GEM | -2.915539759 | 2.332012556 | -9.499417788 | 1.55E-19 | 1.66E-17 | 33.51638313 |
| CTGF | -2.917300851 | 5.166828498 | -7.116875104 | 4.72E-12 | 1.85E-10 | 16.57365809 |
| FAM107A | -2.923388315 | 0.842090541 | -16.55212296 | 7.27E-48 | 1.70E-44 | 98.08712957 |
| RGS2 | -2.934023291 | 3.893252327 | -8.859022674 | 2.21E-17 | 1.80E-15 | 28.62537364 |
| ATP1A2 | -2.948957504 | 0.490418964 | -15.85234352 | 8.28E-45 | 1.38E-41 | 91.11007535 |
| C2orf40 | -2.95479639 | 0.592208643 | -15.18374125 | 6.41E-42 | 7.07E-39 | 84.51591219 |
| SMOC2 | -2.977765257 | 2.32771378 | -8.834951565 | 2.66E-17 | 2.15E-15 | 28.44603905 |
| CRYAB | -2.986935936 | 2.352595283 | -9.137473825 | 2.63E-18 | 2.33E-16 | 30.72399232 |
| SFRP2 | -2.987239783 | 4.003975361 | -4.522238882 | 7.94E-06 | 0.000101638 | 2.668672594 |
| PTGIS | -3.003823143 | 1.902770778 | -7.851628913 | 3.37E-14 | 1.80E-12 | 21.41853083 |
| DCN | -3.014210966 | 3.596078987 | -7.404238809 | 7.13E-13 | 3.15E-11 | 18.42497691 |
| PLN | -3.032370874 | 1.534306601 | -8.773227186 | 4.23E-17 | 3.32E-15 | 27.98771795 |
| NR4A3 | -3.037451325 | 1.177736914 | -13.39172716 | 2.25E-34 | 1.26E-31 | 67.29993451 |
| AOC3 | -3.054537863 | 2.487500005 | -9.965843173 | 3.64E-21 | 4.75E-19 | 37.21736952 |
| CES1 | -3.087902868 | 1.85688785 | -6.476881783 | 2.59E-10 | 7.75E-09 | 12.66085943 |
| CYR61 | -3.095690468 | 5.452860661 | -8.496179145 | 3.33E-16 | 2.35E-14 | 25.95821546 |
| MYLK | -3.101843696 | 2.011639077 | -10.27791035 | 2.78E-22 | 4.09E-20 | 39.7548032 |
| C11orf96 | -3.107650828 | 3.258411448 | -9.543219971 | 1.09E-19 | 1.19E-17 | 33.85911969 |
| PRELP | -3.126842992 | 1.729082657 | -10.11194751 | 1.10E-21 | 1.53E-19 | 38.39940943 |
| SORBS1 | -3.132217448 | 1.673065863 | -10.31029437 | 2.13E-22 | 3.15E-20 | 40.0208272 |
| C16orf89 | -3.154573303 | 0.610827585 | -18.88240451 | 3.30E-58 | 2.75E-54 | 121.6980467 |
| LINC01082 | -3.154961731 | 0.961690009 | -16.10564927 | 6.53E-46 | 1.23E-42 | 93.62749083 |
| CTC-296K1.3 | -3.156672087 | 0.828948607 | -13.24450363 | 9.06E-34 | 4.81E-31 | 65.92161811 |
| SPARCL1 | -3.166352822 | 4.59030219 | -10.01365491 | 2.46E-21 | 3.26E-19 | 37.60301891 |
| ACKR1 | -3.181804678 | 2.116679663 | -8.362581768 | 8.84E-16 | 5.87E-14 | 24.99602668 |
| CASQ2 | -3.183685164 | 0.952342615 | -10.7174048 | 6.90E-24 | 1.21E-21 | 43.40700866 |
| FOS | -3.19269383 | 6.292509469 | -8.70881408 | 6.86E-17 | 5.28E-15 | 27.5118062 |
| HSPB8 | -3.20908326 | 3.554488346 | -7.696830359 | 9.83E-14 | 4.90E-12 | 20.36772406 |
| TNS1 | -3.213376529 | 2.499909974 | -10.49114048 | 4.68E-23 | 7.33E-21 | 41.51554854 |
| DUSP1 | -3.268846791 | 6.138565839 | -9.510780942 | 1.41E-19 | 1.53E-17 | 33.60519777 |
| ADAMTS1 | -3.29019114 | 2.857215813 | -11.38382204 | 2.16E-26 | 4.96E-24 | 49.10866045 |
| OGN | -3.293781556 | 0.725694673 | -14.43356726 | 1.01E-38 | 8.32E-36 | 77.21848878 |
| EGR1 | -3.300361329 | 5.468037348 | -9.46073562 | 2.10E-19 | 2.20E-17 | 33.21455917 |
| TAGLN | -3.31423293 | 5.485185691 | -7.474309488 | 4.46E-13 | 2.02E-11 | 18.88499008 |
| ACTA2 | -3.371749446 | 5.763277469 | -8.167903767 | 3.61E-15 | 2.23E-13 | 23.61357123 |
| PTGS1 | -3.382094655 | 2.153486999 | -9.459796293 | 2.12E-19 | 2.21E-17 | 33.20723988 |
| SDPR | -3.402933008 | 1.779577523 | -12.87385731 | 2.93E-32 | 1.31E-29 | 62.47993781 |
| PGM5-AS1 | -3.435193171 | 0.591722861 | -15.07378704 | 1.90E-41 | 2.05E-38 | 83.43918007 |
| MYL9 | -3.466115942 | 5.889213881 | -8.45562395 | 4.48E-16 | 3.12E-14 | 25.6649836 |
| PDK4 | -3.570661116 | 2.179026689 | -11.46029904 | 1.10E-26 | 2.62E-24 | 49.77484876 |
| DPT | -3.695470812 | 1.759580477 | -9.616869486 | 6.08E-20 | 6.80E-18 | 34.43769056 |
| SCARA5 | -3.760991268 | 0.695064267 | -19.74874173 | 4.32E-62 | 5.05E-58 | 130.5593832 |
| MFAP4 | -3.805716065 | 3.920850358 | -8.801109857 | 3.43E-17 | 2.74E-15 | 28.19448005 |
| C7 | -3.830862834 | 1.245566547 | -11.35474542 | 2.79E-26 | 6.28E-24 | 48.85599519 |
| ADH1B | -3.847621583 | 0.800349637 | -15.89182969 | 5.58E-45 | 9.57E-42 | 91.50185106 |
| PGM5 | -3.870763221 | 1.251287901 | -13.07600667 | 4.42E-33 | 2.13E-30 | 64.35188798 |
| FHL1 | -3.876247111 | 2.191294431 | -11.04379352 | 4.19E-25 | 8.53E-23 | 46.17577982 |
| FLNC | -3.882433477 | 1.856569966 | -10.50061112 | 4.32E-23 | 6.79E-21 | 41.59424702 |
| NR4A1 | -3.889159436 | 3.082770523 | -12.01809383 | 7.56E-29 | 2.30E-26 | 54.7027417 |
| PCP4 | -3.907196759 | 1.682131771 | -8.436984458 | 5.14E-16 | 3.52E-14 | 25.53054636 |
| CLEC3B | -3.958376422 | 1.616404298 | -17.64774207 | 1.05E-52 | 3.42E-49 | 109.1313881 |
| SYNPO2 | -4.017361495 | 1.66752581 | -12.10452987 | 3.46E-29 | 1.08E-26 | 55.47679719 |
| FOSB | -4.074912047 | 3.506525709 | -9.590432298 | 7.51E-20 | 8.29E-18 | 34.22967806 |
| ACTC1 | -4.163797761 | 1.402960214 | -9.592717199 | 7.38E-20 | 8.17E-18 | 34.24764155 |
| LMOD1 | -4.207963664 | 2.481478635 | -10.52668129 | 3.47E-23 | 5.54E-21 | 41.81109796 |
| CFD | -4.229717944 | 3.567121323 | -12.19622125 | 1.50E-29 | 5.02E-27 | 56.30084792 |
| SYNM | -4.322808213 | 1.882727156 | -11.8815279 | 2.59E-28 | 7.33E-26 | 53.48532873 |
| ACTG2 | -4.868656851 | 4.050553015 | -8.926073803 | 1.33E-17 | 1.11E-15 | 29.12668135 |
| CNN1 | -5.043359839 | 3.350814329 | -9.729907201 | 2.46E-20 | 2.88E-18 | 35.33121327 |
| HSPB6 | -5.070281528 | 2.275511792 | -12.15467874 | 2.19E-29 | 7.16E-27 | 55.92712513 |
| PI16 | -5.29308973 | 0.860198074 | -18.63743732 | 4.11E-57 | 2.66E-53 | 119.1974943 |
| MYH11 | -5.548364503 | 2.946500346 | -10.62226017 | 1.55E-23 | 2.59E-21 | 42.60879811 |
| DES | -6.058681878 | 4.138784333 | -7.997416175 | 1.21E-14 | 6.97E-13 | 22.42240127 |

# Supplementary Table S4

| Gene |
|---|
| HLF |
| TCF21 |
| AFF3 |
| NR4A3 |
| EGR3 |
| ZBTB16 |
| NR4A1 |
| SOX17 |
| PRDM6 |
| NR3C2 |
| FOXF1 |
| CSRNP1 |
| KLF2 |
| SRF |
| GATA5 |
| NR4A2 |
| TBX20 |
| HMGB3 |
| GATA6 |
| HAND2 |
| EBF1 |
| MYBL2 |
| HIF3A |
| KLF9 |
| FOSB |
| MEF2D |
| EGR1 |
| ZFPM2 |
| TBX5 |
| ZEB1 |
| HAND1 |
| CENPA |
| FOS |
| NFIA |
| KLF4 |
| MEIS1 |
| FOXM1 |
| MEF2C |
| MEOX1 |
| ZEB2 |
| TSC22D1 |
| ATF3 |
| NFIX |
| TCF3 |
| FOXN3 |
| EGR2 |
| JAZF1 |
| MITF |
| TUB |
| TWIST2 |
| THRA |
| E2F1 |
| HMGA1 |
| MEIS2 |
| TSHZ3 |
| ARID5B |
| NR2F1 |

TEAD1
JUN
ARID5A
TBX4
SOX4
MAFF
NFIC
ETV4
KLF6
NFATC4
JUNB
NFIL3
HOXB7
TSC22D3
JUND
NFIB
NR2F6
ZNF692
CSDC2
PITX2
PARP12
IRF5
PBX1
E2F8
MSX1
JDP2
CDIP1
TCF19
NFE2L3
ZFP36L1
GRHL2
HMGB2
ETS2
SREBF1
ID4
MYC
AR
ID2
TFAP2C
SOX7
ATOH8
ETV7
CREB3L1
TFAP2A
FOSL1
PITX1
ZNF488
IRF7
OSR1
HOXB5
HES4
HOXB6
IRF6
GATA3
TBX1
SP6
MYCL
HES2

SOX9

# Supplementary Table S5

| gene | KM.Pvalue | HR | HR.95L | HR.95H | coxPvalue |
|---|---|---|---|---|---|
| NFIA | 0.00052983 | 1.24774802 | 1.02089044 | 1.52501684 | 0.03062543 |
| EGR2 | 0.00086847 | 1.21606925 | 1.04430364 | 1.41608664 | 0.01180433 |
| CSDC2 | 0.03341661 | 1.18841672 | 1.0209911 | 1.38329736 | 0.02587385 |
| MYC | 0.00441424 | 1.12852106 | 1.00907799 | 1.26210243 | 0.03415038 |
| ID2 | 0.02582234 | 0.83564424 | 0.73559758 | 0.94929797 | 0.00578548 |
| ETV7 | 8.67E-06 | 0.77394341 | 0.67182928 | 0.89157828 | 0.00038577 |
| PITX1 | 0.02408171 | 1.11473651 | 1.00405353 | 1.23762075 | 0.04177204 |
| HOXB5 | 0.00551152 | 0.77656823 | 0.67208786 | 0.89729075 | 0.00060361 |
| HES4 | 0.00903485 | 0.85755488 | 0.76319043 | 0.963587 | 0.009778 |
| HOXB6 | 0.01578521 | 0.82041648 | 0.71185153 | 0.94553876 | 0.00627175 |
| GATA3 | 0.02396519 | 0.90683608 | 0.84233547 | 0.97627573 | 0.00938324 |
| TBX1 | 0.00317948 | 0.85084648 | 0.77329905 | 0.93617047 | 0.00092402 |
| SP6 | 0.01643687 | 0.85184172 | 0.76325411 | 0.95071132 | 0.00420811 |

# Supplementary Table S6

| id | HR | HR.95L | HR.95H | pvalue |
|---|---|---|---|---|
| age | 1.034145192 | 1.017462595 | 1.051101322 | 5.20E-05 |
| gender | 0.836555185 | 0.597360941 | 1.171527179 | 0.298976559 |
| pathologic_stage | 1.786622656 | 1.451989749 | 2.198376757 | 4.15E-08 |
| pathologic_t | 1.716259328 | 1.368824022 | 2.151880763 | 2.86E-06 |
| pathologic_m | 1.186406933 | 1.015673049 | 1.385841057 | 0.031073257 |
| pathologic_n | 1.218743707 | 1.093316548 | 1.358560085 | 0.000356955 |
| ETV7 | 0.963250309 | 0.939008344 | 0.988118118 | 0.003988259 |

# Supplementary Table S7

| id | HR | HR.95L | HR.95H | pvalue |
|---|---|---|---|---|
| age | 1.032082299 | 1.01508778 | 1.049361338 | 0.000193219 |
| pathologic_stage | 1.25938642 | 0.961731613 | 1.649165041 | 0.093671518 |
| pathologic_t | 1.479192444 | 1.114648582 | 1.962959736 | 0.006692214 |
| pathologic_m | 1.188906879 | 1.013452975 | 1.394736215 | 0.033670267 |
| pathologic_n | 1.206125359 | 1.051946254 | 1.382901812 | 0.007238297 |
| ETV7 | 0.971290655 | 0.947953524 | 0.995202309 | 0.0188979 |

# Supplementary Table S8

| | logFC | AveExpr | t | P.Value | adj.P.Val | B |
|---|---|---|---|---|---|---|
| ETV7 | 1.749772046 | 2.678614052 | 27.47174172 | 1.08E-94 | 6.33E-90 | 205.4113409 |
| PSMB8 | 1.328317517 | 5.539403664 | 15.45528694 | 1.02E-42 | 2.98E-38 | 85.93208743 |
| RTP4 | 1.413862703 | 2.869373662 | 13.79428799 | 8.90E-36 | 1.73E-31 | 70.00463227 |
| IFI35 | 1.217763071 | 4.420968973 | 12.95099938 | 2.34E-32 | 1.95E-28 | 62.16394835 |
| PSMB9 | 1.739924186 | 3.79682187 | 12.80668413 | 8.82E-32 | 6.44E-28 | 60.84216007 |
| TAP1 | 1.533151009 | 4.912530527 | 12.65924075 | 3.40E-31 | 2.12E-27 | 59.49823619 |
| IRF1 | 1.148894666 | 3.405502923 | 12.65214517 | 3.63E-31 | 2.12E-27 | 59.43373043 |
| HLA-F | 1.588142799 | 3.922234477 | 12.59805735 | 5.95E-31 | 2.89E-27 | 58.94253687 |
| APOL3 | 1.125780966 | 2.417882433 | 12.56966817 | 7.70E-31 | 3.40E-27 | 58.68509161 |
| BATF2 | 1.344060432 | 1.780182756 | 12.52093254 | 1.20E-30 | 4.67E-27 | 58.2437327 |
| HLA-A | 1.439018508 | 8.518930621 | 12.4719486 | 1.87E-30 | 6.84E-27 | 57.80089091 |
| B2M | 1.197326367 | 9.025079023 | 12.25756425 | 1.30E-29 | 3.73E-26 | 55.87195077 |
| UBE2L6 | 1.254700538 | 5.336784083 | 12.25431695 | 1.34E-29 | 3.73E-26 | 55.84285019 |
| HLA-B | 1.474209435 | 8.884945599 | 12.14978609 | 3.43E-29 | 9.11E-26 | 54.90800563 |
| TAP2 | 1.033733952 | 3.082742232 | 12.12124543 | 4.43E-29 | 1.13E-25 | 54.6534077 |
| HCP5 | 1.46071386 | 3.252270451 | 11.68196488 | 2.20E-27 | 4.94E-24 | 50.77108515 |
| HLA-C | 1.074021251 | 8.70003431 | 11.38781058 | 2.89E-26 | 6.02E-23 | 48.21122539 |
| GBP4 | 1.27990766 | 2.990958746 | 10.9740628 | 1.02E-24 | 1.86E-21 | 44.66834974 |
| UBD | 1.727614985 | 1.921418352 | 10.95451307 | 1.21E-24 | 2.13E-21 | 44.5026855 |
| OASL | 1.257458407 | 2.473418608 | 10.88340804 | 2.21E-24 | 3.69E-21 | 43.90150051 |
| CXCL11 | 1.781598109 | 2.002305496 | 10.51153661 | 5.06E-23 | 7.38E-20 | 40.79295113 |
| HLA-H | 1.149872835 | 4.889010592 | 10.39754352 | 1.30E-22 | 1.77E-19 | 39.85237305 |
| IFIT3 | 1.477729382 | 3.627456749 | 10.31187753 | 2.65E-22 | 3.52E-19 | 39.14944172 |
| IFI44 | 1.456355946 | 3.963265916 | 10.17510372 | 8.15E-22 | 1.06E-18 | 38.03422709 |
| OR2I1P | 1.509587989 | 1.707161933 | 10.12844278 | 1.19E-21 | 1.52E-18 | 37.65578589 |
| SAMD9L | 1.043165366 | 1.994661364 | 10.09800073 | 1.53E-21 | 1.82E-18 | 37.40944668 |
| TRIM22 | 1.12368215 | 2.807012781 | 10.04904668 | 2.28E-21 | 2.61E-18 | 37.01423923 |
| GBP1 | 1.334970622 | 3.67563619 | 10.00565444 | 3.24E-21 | 3.64E-18 | 36.66489924 |
| HLA-DMA | 1.16910447 | 4.376872537 | 9.969427539 | 4.35E-21 | 4.62E-18 | 36.37394571 |
| AC092580.4 | 1.098182697 | 1.464995921 | 9.943053836 | 5.38E-21 | 5.61E-18 | 36.16253026 |
| IFI44L | 1.182740946 | 1.86886036 | 9.924513054 | 6.25E-21 | 6.40E-18 | 36.01410862 |
| LAMP3 | 1.023491842 | 2.326823955 | 9.616428342 | 7.33E-20 | 7.14E-17 | 33.57291299 |
| IDO1 | 1.674346886 | 2.087584825 | 9.60644812 | 7.94E-20 | 7.60E-17 | 33.49463525 |
| CD74 | 1.481370247 | 8.201741802 | 9.566887306 | 1.08E-19 | 1.01E-16 | 33.18485274 |
| AIM2 | 1.609943787 | 2.470992566 | 9.547220544 | 1.27E-19 | 1.16E-16 | 33.03115204 |
| OAS2 | 1.209762624 | 3.711025018 | 9.516749758 | 1.61E-19 | 1.45E-16 | 32.79341116 |
| CTA-384D8.35 | 1.103221747 | 1.555808551 | 9.349897772 | 5.94E-19 | 4.93E-16 | 31.5002076 |
| GNLY | 1.161436122 | 1.493568173 | 9.294977883 | 9.10E-19 | 7.09E-16 | 31.07777016 |
| CASP1 | 1.019589515 | 2.57671255 | 9.191343775 | 2.03E-18 | 1.52E-15 | 30.28504305 |
| CXCL10 | 1.973039439 | 3.775945644 | 9.158861703 | 2.60E-18 | 1.92E-15 | 30.03777626 |
| WARS | 1.229298574 | 4.697364037 | 9.080555139 | 4.74E-18 | 3.37E-15 | 29.44404753 |
| STAT1 | 1.052289791 | 5.188311788 | 8.893779484 | 1.96E-17 | 1.27E-14 | 28.04161294 |
| TNFSF10 | 1.284594455 | 4.834056743 | 8.874044096 | 2.27E-17 | 1.46E-14 | 27.89456985 |
| GZMH | 1.061456453 | 1.46788775 | 8.81550147 | 3.52E-17 | 2.19E-14 | 27.45968444 |
| GBP5 | 1.226012661 | 1.484701858 | 8.801819466 | 3.90E-17 | 2.40E-14 | 27.35832875 |
| NKG7 | 1.266884935 | 2.470888951 | 8.694908747 | 8.66E-17 | 4.86E-14 | 26.57003833 |
| APOL1 | 1.270029814 | 6.443354069 | 8.639980641 | 1.30E-16 | 7.16E-14 | 26.16760371 |
| GZMA | 1.30104792 | 2.277712452 | 8.630190997 | 1.40E-16 | 7.63E-14 | 26.09606364 |
| BST2 | 1.460348275 | 6.73855428 | 8.548859981 | 2.55E-16 | 1.33E-13 | 25.50388929 |
| HLA-DQB1 | 1.310570832 | 3.692461587 | 8.272410724 | 1.90E-15 | 8.92E-13 | 23.52046001 |
| CD2 | 1.048203307 | 2.178992456 | 8.245539442 | 2.30E-15 | 1.06E-12 | 23.330129 |
| GZMB | 1.251268005 | 2.118614492 | 8.219696575 | 2.76E-15 | 1.26E-12 | 23.14749913 |
| MX1 | 1.066745478 | 3.719905379 | 8.160927813 | 4.20E-15 | 1.86E-12 | 22.73371211 |
| ISG15 | 1.120802833 | 6.961370064 | 8.085046412 | 7.20E-15 | 3.07E-12 | 22.2025957 |
| HLA-DRB1 | 1.412337854 | 7.128450159 | 8.014447702 | 1.18E-14 | 4.84E-12 | 21.7116779 |
| HLA-DRB5 | 1.455113537 | 5.327326045 | 7.990639594 | 1.40E-14 | 5.60E-12 | 21.54683019 |
| CD3D | 1.022961728 | 2.298591372 | 7.978491926 | 1.52E-14 | 6.05E-12 | 21.46285716 |

| | | | | | | |
|---|---|---|---|---|---|---|
| CXCL9 | 1.55708421 | 2.83400403 | 7.687197179 | 1.14E-13 | 3.97E-11 | 19.4774235 |
| IFI27 | 1.513116988 | 5.241914651 | 7.663941355 | 1.34E-13 | 4.57E-11 | 19.32127651 |
| HLA-DPA1 | 1.254814991 | 4.151252323 | 7.659463818 | 1.38E-13 | 4.68E-11 | 19.29125349 |
| RARRES3 | 1.370987427 | 4.395298565 | 7.627133746 | 1.72E-13 | 5.76E-11 | 19.07486178 |
| HLA-DRA | 1.371065061 | 7.654804898 | 7.590606495 | 2.20E-13 | 7.30E-11 | 18.83120338 |
| CCL5 | 1.383657018 | 3.970407058 | 7.571692946 | 2.50E-13 | 8.20E-11 | 18.70538421 |
| IL2RG | 1.051234579 | 2.723889904 | 7.366124617 | 9.87E-13 | 2.94E-10 | 17.35322093 |
| IFI16 | 1.004169365 | 4.228530367 | 7.193162099 | 3.07E-12 | 8.37E-10 | 16.23761025 |
| IFITM1 | 1.164264569 | 5.715766023 | 7.143303437 | 4.24E-12 | 1.11E-09 | 15.91982479 |
| HLA-DPB1 | 1.147900051 | 5.15818674 | 7.14152805 | 4.29E-12 | 1.12E-09 | 15.90854059 |
| HLA-DQA1 | 1.112652935 | 2.916819658 | 7.105546981 | 5.41E-12 | 1.39E-09 | 15.68031828 |
| KLHDC7B | 1.595714174 | 3.447126636 | 7.0588815 | 7.30E-12 | 1.82E-09 | 15.38566499 |
| TYMP | 1.113816999 | 4.824572005 | 7.046305369 | 7.91E-12 | 1.95E-09 | 15.30651671 |
| SECTM1 | 1.043089404 | 2.719903416 | 6.986173414 | 1.16E-11 | 2.81E-09 | 14.92960284 |
| IFI6 | 1.24597877 | 7.340307772 | 6.700014781 | 6.97E-11 | 1.52E-08 | 13.17099 |
| PDZK1IP1 | 1.419947702 | 2.982252561 | 6.6390924 | 1.01E-10 | 2.17E-08 | 12.8041685 |
| HLA-DRB6 | 1.019086564 | 3.416528309 | 6.272737133 | 9.09E-10 | 1.66E-07 | 10.65572211 |
| LAMB3 | 1.166010585 | 5.286262677 | 6.238523526 | 1.11E-09 | 2.00E-07 | 10.46018082 |
| SAA1 | 1.41600332 | 2.654930863 | 6.048062623 | 3.33E-09 | 5.38E-07 | 9.387850504 |
| CTA-384D8.31 | 1.496002497 | 3.706587592 | 5.926902493 | 6.59E-09 | 9.89E-07 | 8.72014756 |
| KRT20 | -1.651014337 | 3.849272968 | -5.111245432 | 4.94E-07 | 4.44E-05 | 4.527055269 |
| PTN | -1.187229059 | 3.467783696 | -4.940954515 | 1.14E-06 | 9.03E-05 | 3.719953799 |
| COL17A1 | 1.052893569 | 2.930380651 | 4.84201851 | 1.83E-06 | 0.000132701 | 3.262179777 |
| SLPI | 1.253477216 | 6.10662035 | 4.642403218 | 4.65E-06 | 0.000289119 | 2.363762761 |
| S100A2 | 1.340524778 | 6.890815914 | 4.557765834 | 6.85E-06 | 0.000394335 | 1.993093726 |
| TAC3 | -1.020517525 | 1.387124891 | -4.452689084 | 1.10E-05 | 0.000575002 | 1.541488142 |
| CASP14 | 1.074722252 | 2.298160438 | 4.32014166 | 1.96E-05 | 0.000906287 | 0.985465445 |
| KRT17 | 1.034247792 | 8.441684512 | 4.032005215 | 6.60E-05 | 0.002344174 | -0.170126098 |
| IGF2 | -1.315859273 | 4.783349661 | -3.964475176 | 8.69E-05 | 0.002884411 | -0.430331139 |
| UPK2 | -1.110485535 | 5.791721532 | -3.239613774 | 0.0012953 | 0.021795005 | -2.963902709 |

# Supplementary Table S9
# BP

| ONTOLOC | ID | Description | GeneRatio | BgRatio | pvalue | p.adjust | qvalue | geneID | Count |
|---|---|---|---|---|---|---|---|---|---|
| BP | GO:0034341 | response to interfe | 26/77 | 199/18670 | 1.42E-32 | 2.16E-29 | 1.62E-29 | IRF1/HLA-F | 26 |
| BP | GO:0071346 | cellular response t | 23/77 | 180/18670 | 1.58E-28 | 1.20E-25 | 9.03E-26 | IRF1/HLA-F | 23 |
| BP | GO:0060333 | interferon-gamma- | 19/77 | 91/18670 | 6.44E-28 | 3.26E-25 | 2.45E-25 | IRF1/HLA-F | 19 |
| BP | GO:0060337 | type I interferon si | 18/77 | 95/18670 | 1.23E-25 | 3.72E-23 | 2.80E-23 | PSMB8/IFI3 | 18 |
| BP | GO:0071357 | cellular response t | 18/77 | 95/18670 | 1.23E-25 | 3.72E-23 | 2.80E-23 | PSMB8/IFI3 | 18 |
| BP | GO:0034340 | response to type I | 18/77 | 99/18670 | 2.74E-25 | 6.93E-23 | 5.21E-23 | PSMB8/IFI3 | 18 |
| BP | GO:0002478 | antigen processing | 19/77 | 175/18670 | 3.42E-22 | 7.42E-20 | 5.58E-20 | PSMB8/PSM | 19 |
| BP | GO:0019884 | antigen processing | 19/77 | 182/18670 | 7.35E-22 | 1.40E-19 | 1.05E-19 | PSMB8/PSM | 19 |
| BP | GO:0048002 | antigen processing | 19/77 | 189/18670 | 1.53E-21 | 2.58E-19 | 1.94E-19 | PSMB8/PSM | 19 |
| BP | GO:0019882 | antigen processing | 19/77 | 226/18670 | 4.80E-20 | 7.28E-18 | 5.47E-18 | PSMB8/PSM | 19 |
| BP | GO:0009615 | response to virus | 21/77 | 323/18670 | 8.67E-20 | 1.20E-17 | 9.00E-18 | RTP4/IRF1/( | 21 |
| BP | GO:0051607 | defense response t | 19/77 | 238/18670 | 1.29E-19 | 1.63E-17 | 1.22E-17 | RTP4/IRF1/( | 19 |
| BP | GO:0019883 | antigen processing | 9/77 | 22/18670 | 1.01E-16 | 1.18E-14 | 8.90E-15 | TAP1/HLA-] | 9 |
| BP | GO:0002483 | antigen processing | 8/77 | 15/18670 | 3.62E-16 | 3.66E-14 | 2.75E-14 | TAP1/HLA-] | 8 |
| BP | GO:0019885 | antigen processing | 8/77 | 15/18670 | 3.62E-16 | 3.66E-14 | 2.75E-14 | TAP1/HLA-] | 8 |
| BP | GO:0002475 | antigen processing | 7/77 | 16/18670 | 1.70E-13 | 1.62E-11 | 1.22E-11 | HLA-F/HLA | 7 |
| BP | GO:0002479 | antigen processing | 10/77 | 75/18670 | 5.20E-13 | 4.64E-11 | 3.49E-11 | PSMB8/PSM | 10 |
| BP | GO:0042590 | antigen processing | 10/77 | 79/18670 | 8.91E-13 | 7.52E-11 | 5.65E-11 | PSMB8/PSM | 10 |
| BP | GO:0002474 | antigen processing | 10/77 | 96/18670 | 6.60E-12 | 5.27E-10 | 3.96E-10 | PSMB8/PSM | 10 |
| BP | GO:1903900 | regulation of viral | 11/77 | 149/18670 | 2.45E-11 | 1.86E-09 | 1.40E-09 | OASL/LAM | 11 |
| BP | GO:0050792 | regulation of viral | 12/77 | 208/18670 | 5.18E-11 | 3.75E-09 | 2.82E-09 | OASL/LAM | 12 |
| BP | GO:0045071 | negative regulatio | 8/77 | 59/18670 | 1.08E-10 | 7.29E-09 | 5.48E-09 | OASL/BST2 | 8 |
| BP | GO:0043903 | regulation of inter | 12/77 | 222/18670 | 1.10E-10 | 7.29E-09 | 5.48E-09 | OASL/LAM | 12 |
| BP | GO:0045069 | regulation of viral | 9/77 | 95/18670 | 1.88E-10 | 1.19E-08 | 8.96E-09 | OASL/BST2 | 9 |
| BP | GO:0019886 | antigen processing | 9/77 | 98/18670 | 2.50E-10 | 1.52E-08 | 1.14E-08 | HLA-DMA/( | 9 |
| BP | GO:0002495 | antigen processing | 9/77 | 101/18670 | 3.28E-10 | 1.85E-08 | 1.39E-08 | HLA-DMA/( | 9 |
| BP | GO:0048525 | negative regulatio | 9/77 | 101/18670 | 3.28E-10 | 1.85E-08 | 1.39E-08 | OASL/STAT | 9 |
| BP | GO:0002504 | antigen processing | 9/77 | 102/18670 | 3.59E-10 | 1.95E-08 | 1.46E-08 | HLA-DMA/( | 9 |
| BP | GO:0050852 | T cell receptor sig | 11/77 | 202/18670 | 6.49E-10 | 3.40E-08 | 2.55E-08 | PSMB8/PSM | 11 |
| BP | GO:0001916 | positive regulation | 6/77 | 26/18670 | 8.70E-10 | 4.40E-08 | 3.31E-08 | HLA-F/HLA | 6 |
| BP | GO:0035456 | response to interfe | 6/77 | 28/18670 | 1.41E-09 | 6.93E-08 | 5.21E-08 | IRF1/AIM2/ | 6 |
| BP | GO:0019079 | viral genome repli | 9/77 | 122/18670 | 1.79E-09 | 8.49E-08 | 6.38E-08 | OASL/BST2 | 9 |
| BP | GO:1903901 | negative regulatio | 8/77 | 85/18670 | 2.15E-09 | 9.89E-08 | 7.43E-08 | OASL/BST2 | 8 |
| BP | GO:0001914 | regulation of T cel | 6/77 | 33/18670 | 4.09E-09 | 1.83E-07 | 1.37E-07 | HLA-F/HLA | 6 |
| BP | GO:0001819 | positive regulation | 14/77 | 464/18670 | 5.67E-09 | 2.46E-07 | 1.85E-07 | IRF1/HLA-F | 14 |
| BP | GO:0001906 | cell killing | 9/77 | 168/18670 | 2.98E-08 | 1.26E-06 | 9.45E-07 | HLA-F/HLA | 9 |
| BP | GO:0002711 | positive regulation | 6/77 | 48/18670 | 4.32E-08 | 1.77E-06 | 1.33E-06 | HLA-F/HLA | 6 |
| BP | GO:0001913 | T cell mediated cy | 6/77 | 49/18670 | 4.90E-08 | 1.96E-06 | 1.47E-06 | HLA-F/HLA | 6 |
| BP | GO:0050851 | antigen receptor-m | 11/77 | 316/18670 | 6.79E-08 | 2.64E-06 | 1.99E-06 | PSMB8/PSM | 11 |
| BP | GO:0019058 | viral life cycle | 11/77 | 328/18670 | 9.90E-08 | 3.76E-06 | 2.82E-06 | OASL/LAM | 11 |
| BP | GO:0001912 | positive regulation | 6/77 | 56/18670 | 1.11E-07 | 4.12E-06 | 3.10E-06 | HLA-F/HLA | 6 |
| BP | GO:0001909 | leukocyte mediate | 7/77 | 107/18670 | 2.88E-07 | 1.04E-05 | 7.83E-06 | HLA-F/HLA | 7 |
| BP | GO:0031343 | positive regulation | 6/77 | 68/18670 | 3.61E-07 | 1.27E-05 | 9.57E-06 | HLA-F/HLA | 6 |
| BP | GO:0002709 | regulation of T cel | 6/77 | 70/18670 | 4.29E-07 | 1.48E-05 | 1.11E-05 | HLA-F/HLA | 6 |
| BP | GO:0043122 | regulation of I-kap | 9/77 | 237/18670 | 5.60E-07 | 1.89E-05 | 1.42E-05 | APOL3/UBI | 9 |
| BP | GO:0001910 | regulation of leuk | 6/77 | 78/18670 | 8.19E-07 | 2.70E-05 | 2.03E-05 | HLA-F/HLA | 6 |
| BP | GO:0043123 | positive regulation | 8/77 | 183/18670 | 8.64E-07 | 2.79E-05 | 2.10E-05 | APOL3/UBI | 8 |
| BP | GO:0035458 | cellular response t | 4/77 | 20/18670 | 1.23E-06 | 3.90E-05 | 2.93E-05 | IRF1/AIM2/ | 4 |
| BP | GO:0035455 | response to interfe | 4/77 | 21/18670 | 1.52E-06 | 4.70E-05 | 3.53E-05 | IFIT3/LAMF | 4 |
| BP | GO:0007249 | I-kappaB kinase/N | 9/77 | 269/18670 | 1.61E-06 | 4.88E-05 | 3.66E-05 | APOL3/UBI | 9 |
| BP | GO:0031341 | regulation of cell l | 6/77 | 98/18670 | 3.15E-06 | 9.36E-05 | 7.04E-05 | HLA-F/HLA | 6 |
| BP | GO:0002824 | positive regulation | 6/77 | 100/18670 | 3.54E-06 | 0.000101 | 7.60E-05 | HLA-F/HLA | 6 |
| BP | GO:0002429 | immune response- | 11/77 | 473/18670 | 3.64E-06 | 0.000101 | 7.60E-05 | PSMB8/PSM | 11 |
| BP | GO:0002757 | immune response- | 11/77 | 473/18670 | 3.64E-06 | 0.000101 | 7.60E-05 | PSMB8/PSM | 11 |
| BP | GO:0070663 | regulation of leuk | 8/77 | 222/18670 | 3.66E-06 | 0.000101 | 7.60E-05 | IRF1/IDO1/( | 8 |
| BP | GO:0002819 | regulation of adap | 7/77 | 160/18670 | 4.33E-06 | 0.000117 | 8.82E-05 | IRF1/HLA-F | 7 |
| BP | GO:0030593 | neutrophil chemot | 6/77 | 104/18670 | 4.45E-06 | 0.000117 | 8.83E-05 | CXCL11/CD | 6 |
| BP | GO:0045088 | regulation of innat | 9/77 | 305/18670 | 4.49E-06 | 0.000117 | 8.83E-05 | PSMB8/PSM | 9 |
| BP | GO:0002708 | positive regulation | 6/77 | 105/18670 | 4.70E-06 | 0.000119 | 8.94E-05 | HLA-F/HLA | 6 |
| BP | GO:0002821 | positive regulation | 6/77 | 105/18670 | 4.70E-06 | 0.000119 | 8.94E-05 | HLA-F/HLA | 6 |
| BP | GO:0002456 | T cell mediated im | 6/77 | 106/18670 | 4.97E-06 | 0.000124 | 9.29E-05 | HLA-F/HLA | 6 |
| BP | GO:1990266 | neutrophil migrati | 6/77 | 118/18670 | 9.22E-06 | 0.000226 | 0.00017 | CXCL11/CD | 6 |
| BP | GO:0052548 | regulation of endo | 10/77 | 425/18670 | 9.46E-06 | 0.000228 | 0.00017 | PSMB8/PSM | 10 |
| BP | GO:0071621 | granulocyte chem | 6/77 | 123/18670 | 1.17E-05 | 0.000278 | 0.00021 | CXCL11/CD | 6 |
| BP | GO:0002449 | lymphocyte media | 9/77 | 352/18670 | 1.42E-05 | 0.000331 | 0.00025 | HLA-F/HLA | 9 |
| BP | GO:0052547 | regulation of pepti | 10/77 | 452/18670 | 1.61E-05 | 0.000371 | 0.00028 | PSMB8/PSM | 10 |
| BP | GO:0002697 | regulation of imm | 10/77 | 462/18670 | 1.95E-05 | 0.000441 | 0.00033 | HLA-F/HLA | 10 |
| BP | GO:0042110 | T cell activation | 10/77 | 464/18670 | 2.02E-05 | 0.000451 | 0.00034 | IRF1/B2M/I | 10 |
| BP | GO:0002705 | positive regulation | 6/77 | 136/18670 | 2.07E-05 | 0.000456 | 0.00034 | HLA-F/HLA | 6 |

| | | | | | | | | | |
|---|---|---|---|---|---|---|---|---|---|
| BP | GO:0002703 | regulation of leuk | 7/77 | 205/18670 | 2.18E-05 | 0.000467 | 0.00035 | HLA-F/HLA | 7 |
| BP | GO:0019835 | cytolysis | 4/77 | 40/18670 | 2.18E-05 | 0.000467 | 0.00035 | GZMH/APO | 4 |
| BP | GO:0070665 | positive regulation | 6/77 | 139/18670 | 2.35E-05 | 0.000495 | 0.00037 | CD74/BST2/ | 6 |
| BP | GO:0050670 | regulation of lymp | 7/77 | 208/18670 | 2.39E-05 | 0.000498 | 0.00037 | IRF1/IDO1/ | 7 |
| BP | GO:0032944 | regulation of mon | 7/77 | 209/18670 | 2.47E-05 | 0.000506 | 0.00038 | IRF1/IDO1/ | 7 |
| BP | GO:0097530 | granulocyte migra | 6/77 | 141/18670 | 2.55E-05 | 0.000515 | 0.00039 | CXCL11/CD | 6 |
| BP | GO:0045089 | positive regulation | 7/77 | 214/18670 | 2.87E-05 | 0.000574 | 0.00043 | PSMB8/PSM | 7 |
| BP | GO:0002822 | regulation of adap | 6/77 | 145/18670 | 2.98E-05 | 0.000588 | 0.00044 | HLA-F/HLA | 6 |
| BP | GO:0070661 | leukocyte prolifer | 8/77 | 298/18670 | 3.11E-05 | 0.000605 | 0.00045 | IRF1/IDO1/ | 8 |
| BP | GO:0002699 | positive regulation | 7/77 | 219/18670 | 3.33E-05 | 0.000639 | 0.00048 | HLA-F/HLA | 7 |
| BP | GO:0002706 | regulation of lymp | 6/77 | 149/18670 | 3.47E-05 | 0.000659 | 0.0005 | HLA-F/HLA | 6 |
| BP | GO:0030595 | leukocyte chemot | 7/77 | 224/18670 | 3.84E-05 | 0.000717 | 0.00054 | CXCL11/CD | 7 |
| BP | GO:0002831 | regulation of resp | 9/77 | 400/18670 | 3.87E-05 | 0.000717 | 0.00054 | PSMB8/PSM | 9 |
| BP | GO:0034612 | response to tumor | 8/77 | 312/18670 | 4.30E-05 | 0.000787 | 0.00059 | PSMB8/PSM | 8 |
| BP | GO:0042129 | regulation of T ce | 6/77 | 156/18670 | 4.49E-05 | 0.000804 | 0.0006 | IRF1/IDO1/I | 6 |
| BP | GO:0050863 | regulation of T ce | 8/77 | 314/18670 | 4.50E-05 | 0.000804 | 0.0006 | IRF1/IDO1/ | 8 |
| BP | GO:2000116 | regulation of cyste | 7/77 | 239/18670 | 5.79E-05 | 0.001023 | 0.00077 | PSMB9/LAM | 7 |
| BP | GO:0050663 | cytokine secretion | 7/77 | 240/18670 | 5.95E-05 | 0.001038 | 0.00078 | GBP1/AIM2 | 7 |
| BP | GO:0002833 | positive regulation | 7/77 | 249/18670 | 7.50E-05 | 0.001293 | 0.00097 | PSMB8/PSM | 7 |
| BP | GO:0002237 | response to molec | 8/77 | 343/18670 | 8.36E-05 | 0.001425 | 0.00107 | B2M/CXCL | 8 |
| BP | GO:0032069 | regulation of nucl | 3/77 | 22/18670 | 9.82E-05 | 0.001656 | 0.00124 | OASL/OAS2 | 3 |
| BP | GO:0032732 | positive regulation | 4/77 | 59/18670 | 0.0001025 | 0.001709 | 0.00128 | AIM2/CASP | 4 |
| BP | GO:0042098 | T cell proliferation | 6/77 | 184/18670 | 0.0001121 | 0.00184 | 0.00138 | IRF1/IDO1/I | 6 |
| BP | GO:0032612 | interleukin-1 prod | 5/77 | 115/18670 | 0.0001127 | 0.00184 | 0.00138 | AIM2/CASP | 5 |
| BP | GO:0050701 | interleukin-1 secr | 4/77 | 61/18670 | 0.0001168 | 0.001886 | 0.00142 | AIM2/CASP | 4 |
| BP | GO:0002460 | adaptive immune | 8/77 | 361/18670 | 0.0001191 | 0.001902 | 0.00143 | HLA-F/HLA | 8 |
| BP | GO:0046651 | lymphocyte prolif | 7/77 | 272/18670 | 0.00013 | 0.002055 | 0.00154 | IRF1/IDO1/ | 7 |
| BP | GO:0032943 | mononuclear cell | 7/77 | 274/18670 | 0.000136 | 0.002128 | 0.0016 | IRF1/IDO1/ | 7 |
| BP | GO:0048247 | lymphocyte chem | 4/77 | 64/18670 | 0.0001408 | 0.002182 | 0.00164 | CXCL11/CX | 4 |
| BP | GO:1903706 | regulation of hem | 9/77 | 475/18670 | 0.0001441 | 0.00221 | 0.00166 | PSMB8/PSM | 9 |
| BP | GO:0019730 | antimicrobial hum | 5/77 | 122/18670 | 0.0001489 | 0.00226 | 0.0017 | CXCL11/GN | 5 |
| BP | GO:0051249 | regulation of lymp | 9/77 | 485/18670 | 0.0001685 | 0.002532 | 0.0019 | IRF1/HLA-F | 9 |
| BP | GO:0032479 | regulation of type | 5/77 | 126/18670 | 0.0001732 | 0.002577 | 0.00194 | IRF1/UBE2I | 5 |
| BP | GO:0031349 | positive regulation | 8/77 | 384/18670 | 0.0001818 | 0.002679 | 0.00201 | PSMB8/PSM | 8 |
| BP | GO:0032606 | type I interferon p | 5/77 | 128/18670 | 0.0001864 | 0.002721 | 0.00205 | IRF1/UBE2I | 5 |
| BP | GO:0071356 | cellular response t | 7/77 | 291/18670 | 0.0001969 | 0.002847 | 0.00214 | PSMB8/PSM | 7 |
| BP | GO:0050671 | positive regulation | 5/77 | 130/18670 | 0.0002004 | 0.00287 | 0.00216 | CD74/HLA- | 5 |
| BP | GO:0001773 | myeloid dendritic | 3/77 | 28/18670 | 0.0002052 | 0.002884 | 0.00217 | BATF2/UBD | 3 |
| BP | GO:0010818 | T cell chemotaxis | 3/77 | 28/18670 | 0.0002052 | 0.002884 | 0.00217 | CXCL11/CX | 3 |
| BP | GO:0032946 | positive regulation | 5/77 | 131/18670 | 0.0002077 | 0.002892 | 0.00217 | CD74/HLA- | 5 |
| BP | GO:0070229 | negative regulatio | 3/77 | 29/18670 | 0.0002281 | 0.003146 | 0.00236 | IDO1/CD74/ | 3 |
| BP | GO:0097529 | myeloid leukocyte | 6/77 | 210/18670 | 0.00023 | 0.003146 | 0.00236 | CXCL11/CD | 6 |
| BP | GO:0061844 | antimicrobial hum | 4/77 | 73/18670 | 0.0002346 | 0.00318 | 0.00239 | CXCL11/GN | 4 |
| BP | GO:0060326 | cell chemotaxis | 7/77 | 304/18670 | 0.000257 | 0.003423 | 0.00257 | CXCL11/CD | 7 |
| BP | GO:1903037 | regulation of leuk | 7/77 | 304/18670 | 0.000257 | 0.003423 | 0.00257 | IRF1/IDO1/ | 7 |
| BP | GO:0002718 | regulation of cytol | 4/77 | 84/18670 | 0.0004019 | 0.005305 | 0.00399 | HLA-F/B2M | 4 |
| BP | GO:0032496 | response to lipopo | 7/77 | 330/18670 | 0.0004216 | 0.005517 | 0.00415 | CXCL11/ID | 7 |
| BP | GO:0002690 | positive regulation | 4/77 | 87/18670 | 0.0004593 | 0.005959 | 0.00448 | CD74/CXCL | 4 |
| BP | GO:0007159 | leukocyte cell-cell | 7/77 | 337/18670 | 0.0004779 | 0.006119 | 0.0046 | IRF1/IDO1/ | 7 |
| BP | GO:0070098 | chemokine-media | 4/77 | 88/18670 | 0.0004797 | 0.006119 | 0.0046 | CXCL11/CX | 4 |
| BP | GO:0050716 | positive regulation | 3/77 | 38/18670 | 0.0005129 | 0.006488 | 0.00488 | AIM2/CASP | 3 |
| BP | GO:0051281 | positive regulation | 3/77 | 40/18670 | 0.0005971 | 0.007491 | 0.00563 | CXCL11/CX | 3 |
| BP | GO:0033209 | tumor necrosis fac | 5/77 | 167/18670 | 0.0006338 | 0.007887 | 0.00593 | PSMB8/PSM | 5 |
| BP | GO:0042102 | positive regulation | 4/77 | 95/18670 | 0.0006408 | 0.007909 | 0.00594 | HLA-DPA1/ | 4 |
| BP | GO:1990868 | response to chemo | 4/77 | 97/18670 | 0.0006931 | 0.008417 | 0.00633 | CXCL11/CX | 4 |
| BP | GO:1990869 | cellular response t | 4/77 | 97/18670 | 0.0006931 | 0.008417 | 0.00633 | CXCL11/CX | 4 |
| BP | GO:0071104 | response to interle | 2/77 | 10/18670 | 0.0007395 | 0.008909 | 0.0067 | STAT1/IL2R | 2 |
| BP | GO:0032611 | interleukin-1 beta | 4/77 | 101/18670 | 0.0008067 | 0.009567 | 0.00719 | AIM2/CASP | 4 |
| BP | GO:0032649 | regulation of inter | 4/77 | 101/18670 | 0.0008067 | 0.009567 | 0.00719 | CD2/ISG15/ | 4 |
| BP | GO:0002367 | cytokine productio | 4/77 | 102/18670 | 0.000837 | 0.009849 | 0.0074 | HLA-F/B2M | 4 |
| BP | GO:0032652 | regulation of inter | 4/77 | 103/18670 | 0.0008681 | 0.010137 | 0.00762 | AIM2/CASP | 4 |
| BP | GO:0045646 | regulation of eryth | 3/77 | 47/18670 | 0.0009599 | 0.011039 | 0.0083 | B2M/STAT1 | 3 |
| BP | GO:2000107 | negative regulatio | 3/77 | 47/18670 | 0.0009599 | 0.011039 | 0.0083 | IDO1/CD74/ | 3 |
| BP | GO:0002696 | positive regulation | 7/77 | 380/18670 | 0.0009691 | 0.011061 | 0.00831 | HLA-F/CD7 | 7 |
| BP | GO:0050900 | leukocyte migratio | 8/77 | 499/18670 | 0.0010294 | 0.011662 | 0.00877 | CXCL11/CD | 8 |
| BP | GO:0002791 | regulation of pept | 8/77 | 500/18670 | 0.0010427 | 0.011725 | 0.00881 | GBP1/CD74 | 8 |
| BP | GO:0031581 | hemidesmosome a | 2/77 | 12/18670 | 0.0010788 | 0.012042 | 0.00905 | LAMB3/CO | 2 |
| BP | GO:0072676 | lymphocyte migra | 4/77 | 111/18670 | 0.0011471 | 0.01271 | 0.00955 | CXCL11/CX | 4 |
| BP | GO:0050867 | positive regulation | 7/77 | 394/18670 | 0.0011949 | 0.013144 | 0.00988 | HLA-F/CD7 | 7 |
| BP | GO:0032609 | interferon-gamma | 4/77 | 113/18670 | 0.0012256 | 0.013385 | 0.01006 | CD2/ISG15/ | 4 |

| | | | | | | | | | |
|---|---|---|---|---|---|---|---|---|---|
| BP | GO:0002688 | regulation of leuk | 4/77 | 114/18670 | 0.0012662 | 0.01369 | 0.01029 | CD74/CXCL | 4 |
| BP | GO:0045059 | positive thymic T | 2/77 | 13/18670 | 0.0012716 | 0.01369 | 0.01029 | CD74/CD3D | 2 |
| BP | GO:0032731 | positive regulation | 3/77 | 52/18670 | 0.0012891 | 0.01378 | 0.01036 | AIM2/CASP | 3 |
| BP | GO:0022407 | regulation of cell- | 7/77 | 402/18670 | 0.0013413 | 0.014069 | 0.01058 | IRF1/IDO1/C | 7 |
| BP | GO:0045785 | positive regulation | 7/77 | 403/18670 | 0.0013606 | 0.014069 | 0.01058 | CD74/HLA- | 7 |
| BP | GO:0050702 | interleukin-1 beta | 3/77 | 53/18670 | 0.0013624 | 0.014069 | 0.01058 | AIM2/CASP | 3 |
| BP | GO:0050704 | regulation of inter | 3/77 | 53/18670 | 0.0013624 | 0.014069 | 0.01058 | AIM2/CASP | 3 |
| BP | GO:0070228 | regulation of lymp | 3/77 | 53/18670 | 0.0013624 | 0.014069 | 0.01058 | IDO1/CD74/ | 3 |
| BP | GO:0001818 | negative regulatio | 6/77 | 296/18670 | 0.001394 | 0.014298 | 0.01075 | HLA-F/UBE | 6 |
| BP | GO:0002720 | positive regulation | 3/77 | 54/18670 | 0.0014383 | 0.014555 | 0.01094 | HLA-F/B2M | 3 |
| BP | GO:0010524 | positive regulation | 3/77 | 54/18670 | 0.0014383 | 0.014555 | 0.01094 | CXCL11/CX | 3 |
| BP | GO:0070234 | positive regulation | 2/77 | 14/18670 | 0.0014795 | 0.014815 | 0.01114 | IDO1/CCL5 | 2 |
| BP | GO:0050870 | positive regulation | 5/77 | 202/18670 | 0.0014834 | 0.014815 | 0.01114 | CD74/HLA- | 5 |
| BP | GO:0071222 | cellular response t | 5/77 | 205/18670 | 0.0015829 | 0.015705 | 0.0118 | CXCL11/CA | 5 |
| BP | GO:0030099 | myeloid cell differ | 7/77 | 416/18670 | 0.0016312 | 0.016079 | 0.01209 | BATF2/B2M | 7 |
| BP | GO:0051928 | positive regulation | 4/77 | 123/18670 | 0.0016753 | 0.016407 | 0.01233 | CXCL11/CX | 4 |
| BP | GO:0009612 | response to mecha | 5/77 | 210/18670 | 0.0017594 | 0.017012 | 0.01279 | IRF1/CASP1 | 5 |
| BP | GO:0050707 | regulation of cytol | 5/77 | 210/18670 | 0.0017594 | 0.017012 | 0.01279 | GBP1/AIM2 | 5 |
| BP | GO:0071219 | cellular response t | 5/77 | 212/18670 | 0.001834 | 0.01762 | 0.01324 | CXCL11/CA | 5 |
| BP | GO:0002687 | positive regulation | 4/77 | 128/18670 | 0.0019384 | 0.018412 | 0.01384 | CD74/CXCL | 4 |
| BP | GO:0002830 | positive regulation | 2/77 | 16/18670 | 0.0019406 | 0.018412 | 0.01384 | IDO1/CD74 | 2 |
| BP | GO:1903039 | positive regulation | 5/77 | 218/18670 | 0.0020714 | 0.019531 | 0.01468 | CD74/HLA- | 5 |
| BP | GO:0002683 | negative regulatio | 7/77 | 435/18670 | 0.0021002 | 0.01968 | 0.01479 | IRF1/HLA-F | 7 |
| BP | GO:0010819 | regulation of T cel | 2/77 | 17/18670 | 0.0021935 | 0.020428 | 0.01536 | CXCL10/CC | 2 |
| BP | GO:0050921 | positive regulation | 4/77 | 135/18670 | 0.0023528 | 0.021778 | 0.01637 | CD74/CXCL | 4 |
| BP | GO:0032729 | positive regulation | 3/77 | 65/18670 | 0.0024519 | 0.021976 | 0.01652 | CD2/HLA-D | 3 |
| BP | GO:0072678 | T cell migration | 3/77 | 65/18670 | 0.0024519 | 0.021976 | 0.01652 | CXCL11/CX | 3 |
| BP | GO:0043011 | myeloid dendritic | 2/77 | 18/18670 | 0.0024611 | 0.021976 | 0.01652 | BATF2/UBD | 2 |
| BP | GO:0070230 | positive regulation | 2/77 | 18/18670 | 0.0024611 | 0.021976 | 0.01652 | IDO1/CCL5 | 2 |
| BP | GO:0070233 | negative regulatio | 2/77 | 18/18670 | 0.0024611 | 0.021976 | 0.01652 | IDO1/CCL5 | 2 |
| BP | GO:0090026 | positive regulation | 2/77 | 18/18670 | 0.0024611 | 0.021976 | 0.01652 | CXCL10/CC | 2 |
| BP | GO:0051251 | positive regulation | 6/77 | 334/18670 | 0.0025594 | 0.02272 | 0.01708 | HLA-F/CD7 | 6 |
| BP | GO:0002700 | regulation of prod | 4/77 | 139/18670 | 0.0026152 | 0.022947 | 0.01725 | HLA-F/B2M | 4 |
| BP | GO:0050715 | positive regulation | 4/77 | 139/18670 | 0.0026152 | 0.022947 | 0.01725 | AIM2/CASP | 4 |
| BP | GO:0002544 | chronic inflammat | 2/77 | 19/18670 | 0.0027433 | 0.023933 | 0.01799 | IDO1/CCL5 | 2 |
| BP | GO:0050918 | positive chemotax | 3/77 | 68/18670 | 0.0027884 | 0.024187 | 0.01818 | CXCL10/CC | 3 |
| BP | GO:0002218 | activation of innat | 4/77 | 142/18670 | 0.0028247 | 0.024363 | 0.01831 | PSMB8/PSM | 4 |
| BP | GO:0070227 | lymphocyte apopt | 3/77 | 69/18670 | 0.0029066 | 0.024893 | 0.01871 | IDO1/CD74/ | 3 |
| BP | GO:0071216 | cellular response t | 5/77 | 236/18670 | 0.002919 | 0.024893 | 0.01871 | CXCL11/CA | 5 |
| BP | GO:0051346 | negative regulatio | 7/77 | 463/18670 | 0.0029749 | 0.025228 | 0.01896 | LAMP3/GZN | 7 |
| BP | GO:0033077 | T cell differentiati | 3/77 | 70/18670 | 0.0030278 | 0.025534 | 0.01919 | B2M/CD74/ | 3 |
| BP | GO:0051250 | negative regulatio | 4/77 | 146/18670 | 0.0031215 | 0.026147 | 0.01965 | IRF1/HLA-F | 4 |
| BP | GO:0030217 | T cell differentiati | 5/77 | 240/18670 | 0.003137 | 0.026147 | 0.01965 | IRF1/B2M/C | 5 |
| BP | GO:1904427 | positive regulation | 3/77 | 71/18670 | 0.0031521 | 0.026147 | 0.01965 | CXCL11/CX | 3 |
| BP | GO:0050708 | regulation of prote | 7/77 | 472/18670 | 0.0033084 | 0.027257 | 0.02049 | GBP1/AIM2 | 7 |
| BP | GO:0045061 | thymic T cell selec | 2/77 | 21/18670 | 0.0033511 | 0.027257 | 0.02049 | CD74/CD3D | 2 |
| BP | GO:1901739 | regulation of myol | 2/77 | 21/18670 | 0.0033511 | 0.027257 | 0.02049 | CXCL10/CX | 2 |
| BP | GO:2001056 | positive regulation | 4/77 | 149/18670 | 0.0033577 | 0.027257 | 0.02049 | AIM2/CASP | 4 |
| BP | GO:0006959 | humoral immune r | 6/77 | 356/18670 | 0.0035051 | 0.028302 | 0.02127 | CXCL11/GN | 6 |
| BP | GO:0072643 | interferon-gamma | 2/77 | 22/18670 | 0.0036764 | 0.029528 | 0.0222 | CD2/ISG15 | 2 |
| BP | GO:0010951 | negative regulatio | 5/77 | 250/18670 | 0.0037333 | 0.029827 | 0.02242 | LAMP3/BST | 5 |
| BP | GO:0061025 | membrane fusion | 4/77 | 154/18670 | 0.0037779 | 0.030025 | 0.02257 | TAP1/TAP2/ | 4 |
| BP | GO:0032481 | positive regulation | 3/77 | 77/18670 | 0.0039635 | 0.031337 | 0.02356 | IRF1/STAT1 | 3 |
| BP | GO:0010499 | proteasomal ubiqu | 2/77 | 23/18670 | 0.0040158 | 0.031423 | 0.02362 | PSMB8/PSM | 2 |
| BP | GO:0045109 | intermediate filam | 2/77 | 23/18670 | 0.0040158 | 0.031423 | 0.02362 | KRT20/KRT | 2 |
| BP | GO:0022409 | positive regulation | 5/77 | 255/18670 | 0.0040602 | 0.031607 | 0.02376 | CD74/HLA- | 5 |
| BP | GO:0042832 | defense response t | 2/77 | 24/18670 | 0.0043692 | 0.033667 | 0.02531 | BATF2/GBP | 2 |
| BP | GO:0071677 | positive regulation | 2/77 | 24/18670 | 0.0043692 | 0.033667 | 0.02531 | CXCL10/CC | 2 |
| BP | GO:0010466 | negative regulatio | 5/77 | 262/18670 | 0.0045516 | 0.034896 | 0.02623 | LAMP3/BST | 5 |
| BP | GO:0051279 | regulation of relea | 3/77 | 82/18670 | 0.0047285 | 0.035594 | 0.02676 | CXCL11/CX | 3 |
| BP | GO:0001562 | response to protoz | 2/77 | 25/18670 | 0.0047365 | 0.035594 | 0.02676 | BATF2/GBP | 2 |
| BP | GO:0002719 | negative regulatio | 2/77 | 25/18670 | 0.0047365 | 0.035594 | 0.02676 | HLA-F/BST2 | 2 |
| BP | GO:0090025 | regulation of mon | 2/77 | 25/18670 | 0.0047365 | 0.035594 | 0.02676 | CXCL10/CC | 2 |
| BP | GO:2000106 | regulation of leuk | 3/77 | 83/18670 | 0.0048914 | 0.036577 | 0.02749 | IDO1/CD74/ | 3 |
| BP | GO:0002230 | positive regulation | 2/77 | 26/18670 | 0.0051176 | 0.037895 | 0.02848 | AIM2/STAT | 2 |
| BP | GO:0010996 | response to audito | 2/77 | 26/18670 | 0.0051176 | 0.037895 | 0.02848 | CXCL10/PT | 2 |
| BP | GO:0051100 | negative regulatio | 4/77 | 169/18670 | 0.0052496 | 0.038684 | 0.02908 | B2M/GZMA | 4 |
| BP | GO:1901623 | regulation of lymp | 2/77 | 27/18670 | 0.0055123 | 0.040423 | 0.03039 | CXCL10/CC | 2 |
| BP | GO:0051262 | protein tetrameriz | 4/77 | 172/18670 | 0.005584 | 0.040753 | 0.03063 | B2M/CD74/ | 4 |
| BP | GO:2000108 | positive regulation | 2/77 | 28/18670 | 0.0059205 | 0.042883 | 0.03223 | IDO1/CCL5 | 2 |

| | | | | | | | | | |
|---|---|---|---|---|---|---|---|---|---|
| BP | GO:0002695 | negative regulatio | 4/77 | 175/18670 | 0.0059324 | 0.042883 | 0.03223 | IRF1/HLA-F | 4 |
| BP | GO:0032651 | regulation of inter | 3/77 | 90/18670 | 0.0061269 | 0.043871 | 0.03298 | AIM2/CASP | 3 |
| BP | GO:0045778 | positive regulation | 3/77 | 90/18670 | 0.0061269 | 0.043871 | 0.03298 | ISG15/IFITM | 3 |
| BP | GO:0001959 | regulation of cytol | 4/77 | 177/18670 | 0.0061726 | 0.043991 | 0.03307 | CD74/CASP | 4 |
| BP | GO:0010950 | positive regulation | 4/77 | 178/18670 | 0.0062951 | 0.044571 | 0.0335 | AIM2/CASP | 4 |
| BP | GO:0045639 | positive regulation | 3/77 | 91/18670 | 0.0063172 | 0.044571 | 0.0335 | CD74/STAT | 3 |
| BP | GO:0060142 | regulation of sync | 2/77 | 29/18670 | 0.0063422 | 0.044571 | 0.0335 | CXCL10/CX | 2 |
| BP | GO:0071347 | cellular response t | 4/77 | 179/18670 | 0.0064192 | 0.044905 | 0.03375 | PSMB8/PSM | 4 |
| BP | GO:2000117 | negative regulatio | 3/77 | 92/18670 | 0.006511 | 0.045259 | 0.03402 | LAMP3/IFI1 | 3 |
| BP | GO:1905330 | regulation of morp | 4/77 | 180/18670 | 0.0065449 | 0.045259 | 0.03402 | PSMB8/PSM | 4 |
| BP | GO:0002440 | production of mol | 5/77 | 286/18670 | 0.0065592 | 0.045259 | 0.03402 | HLA-F/B2M | 5 |
| BP | GO:0002828 | regulation of type | 2/77 | 30/18670 | 0.0067771 | 0.046341 | 0.03483 | IDO1/CD74 | 2 |
| BP | GO:0046596 | regulation of viral | 2/77 | 30/18670 | 0.0067771 | 0.046341 | 0.03483 | CD74/IFITM | 2 |
| BP | GO:0006906 | vesicle fusion | 3/77 | 94/18670 | 0.0069092 | 0.047032 | 0.03535 | TAP1/TAP2 | 3 |
| BP | GO:0002702 | positive regulation | 3/77 | 95/18670 | 0.0071136 | 0.048207 | 0.03624 | HLA-F/B2M | 3 |
| BP | GO:0002724 | regulation of T cel | 2/77 | 31/18670 | 0.0072253 | 0.048317 | 0.03632 | HLA-F/B2M | 2 |
| BP | GO:0045648 | positive regulation | 2/77 | 31/18670 | 0.0072253 | 0.048317 | 0.03632 | STAT1/ISG1 | 2 |
| BP | GO:2000406 | positive regulation | 2/77 | 31/18670 | 0.0072253 | 0.048317 | 0.03632 | CXCL10/CC | 2 |

# CC

| ONTOLOGY | ID | Description | GeneRatio | BgRatio | pvalue | p.adjust | qvalue | geneID | Count |
|---|---|---|---|---|---|---|---|---|---|
| CC | GO:0042611 | MHC protein | 15/79 | 25/19717 | 8.49E-31 | 1.07E-28 | 5.90E-29 | HLA-F/HLA- | 15 |
| CC | GO:0071556 | integral comp | 13/79 | 29/19717 | 1.57E-24 | 6.57E-23 | 3.62E-23 | HLA-F/HLA- | 13 |
| CC | GO:0098553 | lumenal side c | 13/79 | 29/19717 | 1.57E-24 | 6.57E-23 | 3.62E-23 | HLA-F/HLA- | 13 |
| CC | GO:0098576 | lumenal side c | 13/79 | 34/19717 | 2.11E-23 | 6.64E-22 | 3.66E-22 | HLA-F/HLA- | 13 |
| CC | GO:0012507 | ER to Golgi tr | 14/79 | 62/19717 | 2.04E-21 | 5.13E-20 | 2.83E-20 | HLA-F/HLA- | 14 |
| CC | GO:0030176 | integral comp | 16/79 | 150/19717 | 7.95E-19 | 1.67E-17 | 9.21E-18 | TAP1/HLA-F | 16 |
| CC | GO:0030134 | COPII-coated | 14/79 | 93/19717 | 9.44E-19 | 1.70E-17 | 9.37E-18 | HLA-F/HLA- | 14 |
| CC | GO:0042613 | MHC class II | 9/79 | 16/19717 | 1.86E-18 | 2.61E-17 | 1.44E-17 | HLA-DMA/C | 9 |
| CC | GO:0031227 | intrinsic comp | 16/79 | 158/19717 | 1.86E-18 | 2.61E-17 | 1.44E-17 | TAP1/HLA-F | 16 |
| CC | GO:0030666 | endocytic vesi | 16/79 | 167/19717 | 4.59E-18 | 5.79E-17 | 3.19E-17 | TAP1/HLA-F | 16 |
| CC | GO:0030660 | Golgi-associat | 14/79 | 114/19717 | 1.86E-17 | 2.13E-16 | 1.18E-16 | HLA-F/HLA- | 14 |
| CC | GO:0030139 | endocytic vesi | 18/79 | 303/19717 | 1.63E-16 | 1.71E-15 | 9.45E-16 | TAP1/HLA-F | 18 |
| CC | GO:0030662 | coated vesicle | 15/79 | 182/19717 | 5.41E-16 | 5.24E-15 | 2.89E-15 | HLA-F/HLA- | 15 |
| CC | GO:0005798 | Golgi-associat | 14/79 | 178/19717 | 1.06E-14 | 9.58E-14 | 5.28E-14 | HLA-F/HLA- | 14 |
| CC | GO:0030658 | transport vesic | 14/79 | 208/19717 | 9.27E-14 | 7.79E-13 | 4.29E-13 | HLA-F/HLA- | 14 |
| CC | GO:0030135 | coated vesicle | 15/79 | 289/19717 | 5.01E-13 | 3.94E-12 | 2.17E-12 | HLA-F/HLA- | 15 |
| CC | GO:0031301 | integral comp | 16/79 | 350/19717 | 5.41E-13 | 4.01E-12 | 2.21E-12 | TAP1/HLA-F | 16 |
| CC | GO:0031300 | intrinsic comp | 16/79 | 381/19717 | 1.97E-12 | 1.38E-11 | 7.60E-12 | TAP1/HLA-F | 16 |
| CC | GO:0030669 | clathrin-coate | 8/79 | 44/19717 | 7.28E-12 | 4.83E-11 | 2.66E-11 | CD74/HLA-D | 8 |
| CC | GO:0045334 | clathrin-coate | 8/79 | 63/19717 | 1.50E-10 | 9.43E-10 | 5.20E-10 | CD74/HLA-D | 8 |
| CC | GO:0030133 | transport vesic | 14/79 | 392/19717 | 4.61E-10 | 2.77E-09 | 1.53E-09 | HLA-F/HLA- | 14 |
| CC | GO:0010008 | endosome me | 15/79 | 479/19717 | 6.32E-10 | 3.62E-09 | 2.00E-09 | HLA-F/HLA- | 15 |
| CC | GO:0030670 | phagocytic ve | 8/79 | 76/19717 | 6.99E-10 | 3.83E-09 | 2.11E-09 | TAP1/HLA-F | 8 |
| CC | GO:0030665 | clathrin-coate | 9/79 | 115/19717 | 8.32E-10 | 4.37E-09 | 2.41E-09 | CD74/HLA-D | 9 |
| CC | GO:0005765 | lysosomal me | 13/79 | 354/19717 | 1.47E-09 | 7.36E-09 | 4.06E-09 | HLA-F/HLA- | 13 |
| CC | GO:0098852 | lytic vacuole r | 13/79 | 355/19717 | 1.52E-09 | 7.36E-09 | 4.06E-09 | HLA-F/HLA- | 13 |
| CC | GO:0045335 | phagocytic ve | 9/79 | 132/19717 | 2.84E-09 | 1.33E-08 | 7.31E-09 | TAP1/HLA-F | 9 |
| CC | GO:0032588 | trans-Golgi ne | 8/79 | 92/19717 | 3.28E-09 | 1.48E-08 | 8.14E-09 | CD74/HLA-D | 8 |
| CC | GO:0005774 | vacuolar mem | 13/79 | 412/19717 | 9.07E-09 | 3.94E-08 | 2.17E-08 | HLA-F/HLA- | 13 |
| CC | GO:0009897 | external side c | 12/79 | 393/19717 | 5.12E-08 | 2.15E-07 | 1.18E-07 | HLA-F/HLA- | 12 |
| CC | GO:0030136 | clathrin-coate | 9/79 | 188/19717 | 6.23E-08 | 2.53E-07 | 1.40E-07 | CD74/HLA-D | 9 |
| CC | GO:0055038 | recycling endo | 6/79 | 81/19717 | 8.72E-07 | 3.43E-06 | 1.89E-06 | HLA-F/HLA- | 6 |
| CC | GO:0005802 | trans-Golgi ne | 8/79 | 236/19717 | 4.70E-06 | 1.79E-05 | 9.89E-06 | CD74/HLA-D | 8 |
| CC | GO:0031901 | early endoson | 6/79 | 149/19717 | 2.97E-05 | 0.00011 | 6.07E-05 | HLA-F/HLA- | 6 |
| CC | GO:0005770 | late endosome | 7/79 | 256/19717 | 7.49E-05 | 0.000263 | 0.000145 | HLA-DMA/L | 7 |
| CC | GO:0055037 | recycling endo | 6/79 | 176/19717 | 7.53E-05 | 0.000263 | 0.000145 | HLA-F/HLA- | 6 |
| CC | GO:0005769 | early endoson | 8/79 | 350/19717 | 7.93E-05 | 0.00027 | 0.000149 | HLA-F/HLA- | 8 |
| CC | GO:0098791 | Golgi apparatu | 8/79 | 359/19717 | 9.46E-05 | 0.000314 | 0.000173 | CD74/HLA-D | 8 |
| CC | GO:0031984 | organelle subc | 8/79 | 380/19717 | 0.00014 | 0.000452 | 0.000249 | CD74/HLA-D | 8 |
| CC | GO:0031902 | late endosome | 5/79 | 134/19717 | 0.0002026 | 0.000638 | 0.000352 | HLA-DMA/L | 5 |
| CC | GO:0019774 | proteasome cc | 2/79 | 11/19717 | 0.0008516 | 0.002617 | 0.001443 | PSMB8/PSM | 2 |
| CC | GO:0061702 | inflammasome | 2/79 | 14/19717 | 0.0013981 | 0.004194 | 0.002313 | AIM2/CASP1 | 2 |
| CC | GO:0005839 | proteasome cc | 2/79 | 20/19717 | 0.002874 | 0.00823 | 0.004538 | PSMB8/PSM | 2 |
| CC | GO:0071682 | endocytic vesi | 2/79 | 20/19717 | 0.002874 | 0.00823 | 0.004538 | GNLY/SAA1 | 2 |
| CC | GO:0034364 | high-density l | 2/79 | 26/19717 | 0.0048402 | 0.013553 | 0.007473 | APOL1/SAA1 | 2 |
| CC | GO:0005604 | basement men | 3/79 | 95/19717 | 0.0065778 | 0.018017 | 0.009934 | LAMB3/PTN | 3 |
| CC | GO:0001772 | immunologica | 2/79 | 36/19717 | 0.009143 | 0.024511 | 0.013515 | GZMA/GZMI | 2 |
| CC | GO:0034358 | plasma lipopro | 2/79 | 37/19717 | 0.0096406 | 0.02479 | 0.013669 | APOL1/SAA1 | 2 |
| CC | GO:1990777 | lipoprotein pa | 2/79 | 37/19717 | 0.0096406 | 0.02479 | 0.013669 | APOL1/SAA1 | 2 |
| CC | GO:0032994 | protein-lipid c | 2/79 | 39/19717 | 0.010671 | 0.026891 | 0.014827 | APOL1/SAA1 | 2 |
| CC | GO:0005771 | multivesicular | 2/79 | 51/19717 | 0.0178024 | 0.043983 | 0.024251 | CD74/BST2 | 2 |

# MF

| ONTOLOGY | ID | Description | GeneRatio | BgRatio | pvalue | p.adjust | qvalue | geneID | Count |
|---|---|---|---|---|---|---|---|---|---|
| MF | GO:0042605 | peptide antige | 13/75 | 31/17696 | 9.23E-24 | 1.58E-21 | 1.13E-21 | TAP1/HLA-F | 13 |
| MF | GO:0003823 | antigen bindir | 13/75 | 160/17696 | 1.29E-13 | 1.10E-11 | 7.85E-12 | TAP1/HLA-F | 13 |
| MF | GO:0042277 | peptide bindir | 14/75 | 295/17696 | 2.12E-11 | 1.21E-09 | 8.63E-10 | TAP1/HLA-F | 14 |
| MF | GO:0033218 | amide binding | 14/75 | 356/17696 | 2.55E-10 | 1.01E-08 | 7.23E-09 | TAP1/HLA-F | 14 |
| MF | GO:0032395 | MHC class II | 5/75 | 10/17696 | 2.96E-10 | 1.01E-08 | 7.23E-09 | HLA-DQB1/I | 5 |
| MF | GO:0023026 | MHC class II | 4/75 | 16/17696 | 5.21E-07 | 1.49E-05 | 1.06E-05 | HLA-DMA/C | 4 |
| MF | GO:0140375 | immune recep | 7/75 | 128/17696 | 1.16E-06 | 2.83E-05 | 2.02E-05 | CD74/HLA-D | 7 |
| MF | GO:0023023 | MHC protein | 4/75 | 25/17696 | 3.52E-06 | 7.52E-05 | 5.37E-05 | HLA-DMA/C | 4 |
| MF | GO:0042379 | chemokine re | 5/75 | 66/17696 | 8.73E-06 | 0.000166 | 0.00012 | CXCL11/CX( | 5 |
| MF | GO:0045236 | CXCR chemo | 3/75 | 11/17696 | 1.18E-05 | 0.000201 | 0.00014 | CXCL11/CX( | 3 |
| MF | GO:0042287 | MHC protein | 4/75 | 40/17696 | 2.42E-05 | 0.000377 | 0.00027 | TAP1/TAP2/( | 4 |
| MF | GO:0048018 | receptor ligan | 10/75 | 482/17696 | 3.48E-05 | 0.000495 | 0.00035 | CXCL11/CX( | 10 |
| MF | GO:0030546 | signaling rece | 10/75 | 487/17696 | 3.79E-05 | 0.000499 | 0.00036 | CXCL11/CX( | 10 |
| MF | GO:0008009 | chemokine ac | 4/75 | 49/17696 | 5.46E-05 | 0.000667 | 0.00048 | CXCL11/CX( | 4 |
| MF | GO:0001664 | G protein-cou | 7/75 | 280/17696 | 0.000183 | 0.002081 | 0.00149 | RTP4/CXCL1 | 7 |
| MF | GO:0005125 | cytokine activ | 6/75 | 220/17696 | 0.00034 | 0.003633 | 0.00259 | CXCL11/CX( | 6 |
| MF | GO:0042056 | chemoattracta | 3/75 | 38/17696 | 0.000554 | 0.005577 | 0.00398 | CXCL10/CCI | 3 |
| MF | GO:0005126 | cytokine recep | 6/75 | 286/17696 | 0.001338 | 0.012715 | 0.00908 | CXCL11/CX( | 6 |
| MF | GO:0097153 | cysteine-type | 2/75 | 15/17696 | 0.001796 | 0.016161 | 0.01154 | CASP1/CASI | 2 |
| MF | GO:0004175 | endopeptidase | 7/75 | 427/17696 | 0.002194 | 0.018761 | 0.0134 | PSMB8/PSM | 7 |
| MF | GO:0042288 | MHC class I p | 2/75 | 20/17696 | 0.003205 | 0.026099 | 0.01864 | TAP1/TAP2 | 2 |
| MF | GO:0004298 | threonine-type | 2/75 | 21/17696 | 0.003533 | 0.026266 | 0.01876 | PSMB8/PSM | 2 |
| MF | GO:0070003 | threonine-type | 2/75 | 21/17696 | 0.003533 | 0.026266 | 0.01876 | PSMB8/PSM | 2 |
| MF | GO:0030247 | polysaccharid | 2/75 | 25/17696 | 0.004992 | 0.035568 | 0.0254 | HLA-DRB1/I | 2 |
| MF | GO:0008201 | heparin bindir | 4/75 | 169/17696 | 0.005764 | 0.039428 | 0.02815 | CXCL11/CX( | 4 |

# Supplementary Table S10

| ID | Description | GeneRatio | BgRatio | pvalue | p.adjust | qvalue | geneID | Count |
|---|---|---|---|---|---|---|---|---|
| hsa04612 | Antigen processing and | | 16/55 | 78/8081 | 2.47E-20 | 2.35E-18 | 1.22E-18 | TAP1/HLA-] | 16 |
| hsa05330 | Allograft rejection | 13/55 | 38/8081 | 6.98E-20 | 3.32E-18 | 1.73E-18 | HLA-F/HLA | 13 |
| hsa05332 | Graft-versus-host diseas | 13/55 | 42/8081 | 3.23E-19 | 1.02E-17 | 5.32E-18 | HLA-F/HLA | 13 |
| hsa04940 | Type I diabetes mellitus | 13/55 | 43/8081 | 4.60E-19 | 1.09E-17 | 5.69E-18 | HLA-F/HLA | 13 |
| hsa05169 | Epstein-Barr virus infect | 20/55 | 202/8081 | 8.28E-19 | 1.57E-17 | 8.20E-18 | TAP1/HLA-] | 20 |
| hsa05320 | Autoimmune thyroid dis | 13/55 | 53/8081 | 1.01E-17 | 1.60E-16 | 8.32E-17 | HLA-F/HLA | 13 |
| hsa05416 | Viral myocarditis | 12/55 | 60/8081 | 3.02E-15 | 4.09E-14 | 2.13E-14 | HLA-F/HLA | 12 |
| hsa05164 | Influenza A | 15/55 | 171/8081 | 2.35E-13 | 2.79E-12 | 1.45E-12 | HLA-DMA/( | 15 |
| hsa04145 | Phagosome | 14/55 | 152/8081 | 8.51E-13 | 8.99E-12 | 4.68E-12 | TAP1/HLA-] | 14 |
| hsa05166 | Human T-cell leukemia | 15/55 | 219/8081 | 8.83E-12 | 8.39E-11 | 4.37E-11 | HLA-F/HLA | 15 |
| hsa05321 | Inflammatory bowel dise | 10/55 | 65/8081 | 1.22E-11 | 9.93E-11 | 5.17E-11 | HLA-DMA/S | 10 |
| hsa04514 | Cell adhesion molecules | 13/55 | 149/8081 | 1.25E-11 | 9.93E-11 | 5.17E-11 | HLA-F/HLA | 13 |
| hsa04658 | Th1 and Th2 cell differe | 11/55 | 92/8081 | 1.79E-11 | 1.29E-10 | 6.70E-11 | HLA-DMA/S | 11 |
| hsa05310 | Asthma | 8/55 | 31/8081 | 1.90E-11 | 1.29E-10 | 6.70E-11 | HLA-DMA/I | 8 |
| hsa05168 | Herpes simplex virus 1 i | 20/55 | 498/8081 | 2.87E-11 | 1.82E-10 | 9.46E-11 | TAP1/HLA-] | 20 |
| hsa04659 | Th17 cell differentiation | 11/55 | 107/8081 | 9.58E-11 | 5.69E-10 | 2.96E-10 | HLA-DMA/S | 11 |
| hsa05150 | Staphylococcus aureus i | 10/55 | 96/8081 | 6.54E-10 | 3.65E-09 | 1.90E-09 | HLA-DMA/I | 10 |
| hsa04640 | Hematopoietic cell linea | 10/55 | 99/8081 | 8.90E-10 | 4.69E-09 | 2.44E-09 | HLA-DMA/I | 10 |
| hsa04672 | Intestinal immune netwo | 8/55 | 49/8081 | 9.87E-10 | 4.94E-09 | 2.57E-09 | HLA-DMA/I | 8 |
| hsa05140 | Leishmaniasis | 9/55 | 77/8081 | 1.79E-09 | 8.51E-09 | 4.43E-09 | HLA-DMA/S | 9 |
| hsa05145 | Toxoplasmosis | 10/55 | 112/8081 | 3.02E-09 | 1.37E-08 | 7.12E-09 | HLA-DMA/S | 10 |
| hsa05323 | Rheumatoid arthritis | 9/55 | 93/8081 | 9.84E-09 | 4.25E-08 | 2.21E-08 | HLA-DMA/I | 9 |
| hsa05152 | Tuberculosis | 10/55 | 180/8081 | 2.88E-07 | 1.19E-06 | 6.19E-07 | HLA-DMA/( | 10 |
| hsa04621 | NOD-like receptor signa | 9/55 | 181/8081 | 3.04E-06 | 1.20E-05 | 6.27E-06 | GBP4/GBP1 | 9 |
| hsa05322 | Systemic lupus erythema | 8/55 | 136/8081 | 3.28E-06 | 1.24E-05 | 6.48E-06 | HLA-DMA/I | 8 |
| hsa05170 | Human immunodeficien | 9/55 | 212/8081 | 1.11E-05 | 4.05E-05 | 2.11E-05 | TAP1/HLA-] | 9 |
| hsa04061 | Viral protein interaction | 6/55 | 100/8081 | 5.48E-05 | 0.0001929 | 0.0001 | CXCL11/CX | 6 |
| hsa05165 | Human papillomavirus i | 10/55 | 331/8081 | 6.55E-05 | 0.0002224 | 0.000116 | IRF1/HLA-F | 10 |
| hsa05340 | Primary immunodeficien | 4/55 | 38/8081 | 0.0001194 | 0.0003911 | 0.000204 | TAP1/TAP2/ | 4 |
| hsa05163 | Human cytomegalovirus | 8/55 | 225/8081 | 0.0001256 | 0.0003977 | 0.000207 | TAP1/HLA-] | 8 |
| hsa04620 | Toll-like receptor signali | 5/55 | 104/8081 | 0.0006692 | 0.0020508 | 0.001068 | CXCL11/CX | 5 |
| hsa04623 | Cytosolic DNA-sensing | 4/55 | 63/8081 | 0.0008495 | 0.002522 | 0.001313 | AIM2/CASP | 4 |
| hsa04650 | Natural killer cell media | 5/55 | 131/8081 | 0.001885 | 0.0054265 | 0.002826 | HLA-A/HLA | 5 |
| hsa05162 | Measles | 5/55 | 139/8081 | 0.0024442 | 0.0068295 | 0.003557 | OAS2/STAT | 5 |
| hsa05171 | Coronavirus disease - C( | 6/55 | 232/8081 | 0.0046964 | 0.0127474 | 0.006639 | OAS2/CASP | 6 |
| hsa04062 | Chemokine signaling pa | 5/55 | 192/8081 | 0.00956 | 0.0250703 | 0.013056 | CXCL11/CX | 5 |
| hsa05167 | Kaposi sarcoma-associat | 5/55 | 193/8081 | 0.0097642 | 0.0250703 | 0.013056 | HLA-F/HLA | 5 |
| hsa04060 | Cytokine-cytokine recep | 6/55 | 295/8081 | 0.0145206 | 0.0363015 | 0.018905 | CXCL11/CX | 6 |

# Supplementary Table S11
# HIGH-ETV7

| NAME | GS<br> follo | GS DETA | SIZE | ES | NES | NOM p- | FDR q-val | FWER p-v | RANK A | LEADING EDG |
|---|---|---|---|---|---|---|---|---|---|---|
| GO_HEART | GO_HEART_ | Details ... | 33 | -0.6801 | -2.1359 | 0 | 0.095677 | 0.061 | 8882 | tags=52%, list=1 |
| GO_POSITI | GO_POSITI | Details ... | 38 | -0.6171 | -2.1261 | 0 | 0.058888 | 0.072 | 12788 | tags=53%, list=2 |
| GO_SMOOT | GO_SMOOT | Details ... | 24 | -0.746 | -2.0563 | 0 | 0.14334 | 0.173 | 7471 | tags=58%, list=1 |
| GO_CARDI | GO_CARDI | Details ... | 73 | -0.5774 | -2.0543 | 0 | 0.111961 | 0.175 | 9208 | tags=47%, list=1 |
| GO_CARDI | GO_CARDI | Details ... | 127 | -0.5299 | -2.0539 | 0 | 0.089712 | 0.175 | 12749 | tags=48%, list=2 |
| GO_VENTR | GO_VENTR | Details ... | 72 | -0.5606 | -1.9996 | 0 | 0.170851 | 0.301 | 12749 | tags=50%, list=2 |
| GO_AORTA | GO_AORTA | Details ... | 35 | -0.6539 | -1.9865 | 0 | 0.181701 | 0.345 | 8882 | tags=49%, list=1 |
| GO_VENTR | GO_VENTR | Details ... | 16 | -0.7194 | -1.9807 | 0 | 0.171366 | 0.36 | 8882 | tags=50%, list=1 |
| GO_CARDI | GO_CARDI | Details ... | 127 | -0.5187 | -1.9764 | 0 | 0.162129 | 0.371 | 12788 | tags=48%, list=2 |
| GO_ENDOC | GO_ENDOC | Details ... | 45 | -0.641 | -1.9692 | 0 | 0.160401 | 0.388 | 13311 | tags=62%, list=2 |
| GO_REGUL | GO_REGUL | Details ... | 34 | -0.6043 | -1.965 | 0 | 0.154823 | 0.401 | 7643 | tags=47%, list=1 |
| GO_POSITI | GO_POSITI | Details ... | 52 | -0.5502 | -1.9555 | 0 | 0.161212 | 0.432 | 12950 | tags=48%, list=2 |
| GO_CARDI | GO_CARDI | Details ... | 74 | -0.5518 | -1.9406 | 0 | 0.179121 | 0.473 | 15844 | tags=58%, list=2 |
| GO_CARDI | GO_CARDI | Details ... | 169 | -0.4954 | -1.9205 | 0 | 0.1993 | 0.53 | 12788 | tags=45%, list=2 |
| GO_VENTR | GO_VENTR | Details ... | 43 | -0.583 | -1.9154 | 0 | 0.186481 | 0.545 | 12664 | tags=53%, list=2 |
| GO_POSITI | GO_POSITI | Details ... | 61 | -0.5145 | -1.9056 | 0 | 0.200393 | 0.579 | 6716 | tags=34%, list=1 |
| GO_SYNAP | GO_SYNAP | Details ... | 16 | -0.7738 | -1.9001 | 0 | 0.193154 | 0.589 | 9188 | tags=75%, list=1 |
| GO_STERO | GO_STEROI | Details ... | 20 | -0.6676 | -1.8815 | 0 | 0.227893 | 0.637 | 8941 | tags=40%, list=1 |
| GO_CARDI | GO_CARDI | Details ... | 110 | -0.5122 | -1.875 | 0 | 0.23483 | 0.655 | 12788 | tags=45%, list=2 |
| GO_ENDOC | GO_ENDOC | Details ... | 35 | -0.6373 | -1.8728 | 0 | 0.229994 | 0.657 | 13311 | tags=60%, list=2 |
| GO_GAMM | GO_GAMM | Details ... | 26 | -0.6494 | -1.8661 | 0 | 0.219056 | 0.666 | 12500 | tags=65%, list=2 |
| GO_VENTR | GO_VENTR | Details ... | 55 | -0.531 | -1.8652 | 0 | 0.212414 | 0.667 | 9208 | tags=42%, list=1 |
| GO_MESEN | GO_MESEN | Details ... | 52 | -0.6044 | -1.8577 | 0 | 0.215545 | 0.685 | 13311 | tags=62%, list=2 |
| GO_TRABE | GO_TRABE | Details ... | 48 | -0.55 | -1.8515 | 0 | 0.208678 | 0.697 | 8882 | tags=46%, list=1 |
| GO_LATER | GO_LATER | Details ... | 15 | -0.6554 | -1.8474 | 0 | 0.194019 | 0.705 | 1087 | tags=40%, list=2 |
| GO_CARDI | GO_CARDI | Details ... | 20 | -0.6248 | -1.8417 | 0 | 0.201133 | 0.718 | 8882 | tags=50%, list=1 |
| GO_ORGAN | GO_ORGAN_INDUCT | | 24 | -0.6365 | -1.835 | 0 | 0.194407 | 0.74 | 11983 | tags=54%, list=2 |
| GO_FIBRO | GO_FIBROBLAST_GR | | 115 | -0.4726 | -1.8308 | 0 | 0.193739 | 0.748 | 9127 | tags=37%, list=1 |
| GO_CELL_ | GO_CELL_SURFACE_ | | 33 | -0.5979 | -1.8272 | 0 | 0.192085 | 0.757 | 11983 | tags=48%, list=2 |
| GO_HINDB | GO_HINDBRAIN_MO | | 42 | -0.5574 | -1.7899 | 0 | 0.233857 | 0.83 | 11744 | tags=50%, list=2 |
| GO_HEART | GO_HEART_MORPHO | | 252 | -0.4438 | -1.7673 | 0 | 0.228275 | 0.866 | 12817 | tags=42%, list=2 |
| GO_SPECIF | GO_SPECIFICATION_ | | 133 | -0.4334 | -1.7112 | 0 | 0.268296 | 0.935 | 15533 | tags=46%, list=2 |
| GO_REGUL | GO_REGULATION_OI | | 77 | -0.5009 | -1.7546 | 0.0019 | 0.246566 | 0.877 | 8997 | tags=38%, list=1 |
| GO_RESPO | GO_RESPONSE_TO_F | | 150 | -0.4152 | -1.6615 | 0.0019 | 0.296147 | 0.97 | 9127 | tags=33%, list=1 |
| GO_SULFU | GO_SULFU | Details ... | 15 | -0.678 | -1.836 | 0.0019 | 0.19727 | 0.738 | 7021 | tags=40%, list=1 |
| GO_CONVE | GO_CONVE | Details ... | 15 | -0.6675 | -1.8392 | 0.002 | 0.195507 | 0.725 | 11983 | tags=53%, list=2 |
| GO_PDZ_D | GO_PDZ_DOMAIN_B | | 82 | -0.4908 | -1.7985 | 0.002 | 0.227175 | 0.809 | 10885 | tags=46%, list=1 |
| GO_ENDOC | GO_ENDOCRINE_HO | | 45 | -0.4919 | -1.6933 | 0.002 | 0.285436 | 0.951 | 14012 | tags=42%, list=2 |
| GO_DIGEST | GO_DIGESTIVE_TRA | | 49 | -0.5509 | -1.8286 | 0.002 | 0.193367 | 0.752 | 12111 | tags=51%, list=2 |
| GO_REGUL | GO_REGUL | Details ... | 57 | -0.5184 | -1.8551 | 0.002 | 0.207934 | 0.692 | 8230 | tags=39%, list=1 |
| GO_NEGAT | GO_NEGAT | Details ... | 19 | -0.6637 | -1.8695 | 0.002 | 0.220189 | 0.66 | 4615 | tags=42%, list=8 |
| GO_ANIMA | GO_ANIMAL_ORGAN | | 65 | -0.4933 | -1.7686 | 0.002 | 0.235579 | 0.863 | 11983 | tags=48%, list=2 |
| GO_SPECIF | GO_SPECIF | Details ... | 36 | -0.5854 | -1.8712 | 0.002 | 0.224902 | 0.658 | 11983 | tags=50%, list=2 |
| GO_ATRIO | GO_ATRIOVENTRICU | | 25 | -0.6272 | -1.8127 | 0.002 | 0.214023 | 0.787 | 12664 | tags=64%, list=2 |
| GO_REGUL | GO_REGULATION_OI | | 93 | -0.4876 | -1.7216 | 0.002 | 0.265799 | 0.925 | 10004 | tags=43%, list=1 |
| GO_CARDI | GO_CARDIAC_MUSC | | 63 | -0.4897 | -1.7424 | 0.0021 | 0.266048 | 0.898 | 12664 | tags=46%, list=2 |
| GO_AORTA | GO_AORTA_DEVELO | | 60 | -0.5397 | -1.7382 | 0.0021 | 0.2561 | 0.904 | 8882 | tags=40%, list=1 |
| GO_ARTER | GO_ARTER | Details ... | 74 | -0.5366 | -1.8489 | 0.0021 | 0.201336 | 0.702 | 15325 | tags=51%, list=2 |
| GO_REGUL | GO_REGUL | Details ... | 38 | -0.5885 | -1.8478 | 0.0021 | 0.198626 | 0.703 | 11983 | tags=50%, list=2 |
| GO_ENDOC | GO_ENDOC | Details ... | 24 | -0.6882 | -1.8411 | 0.0021 | 0.196917 | 0.719 | 8882 | tags=54%, list=1 |
| GO_DENDR | GO_DENDRITE_MEM | | 43 | -0.5942 | -1.7789 | 0.0021 | 0.227658 | 0.846 | 12500 | tags=65%, list=2 |
| GO_HEART | GO_HEART_FORMAT | | 31 | -0.5588 | -1.7687 | 0.0021 | 0.238845 | 0.863 | 11983 | tags=52%, list=2 |
| GO_GABA_ | GO_GABA_ | Details ... | 22 | -0.7053 | -1.9017 | 0.0021 | 0.199603 | 0.587 | 12825 | tags=73%, list=2 |
| GO_GABA_ | GO_GABA_ | Details ... | 20 | -0.7341 | -1.9375 | 0.0022 | 0.171831 | 0.477 | 12825 | tags=80%, list=2 |
| GO_WNT_A | GO_WNT_A | Details ... | 15 | -0.7217 | -1.9173 | 0.0022 | 0.193319 | 0.54 | 12292 | tags=73%, list=2 |
| GO_POSITI | GO_POSITIVE_REGU | | 74 | -0.4924 | -1.7391 | 0.0038 | 0.257303 | 0.904 | 9553 | tags=38%, list=1 |
| GO_REGUL | GO_REGULATION_OI | | 153 | -0.4506 | -1.6888 | 0.0039 | 0.2862 | 0.953 | 10004 | tags=38%, list=1 |

| | | | | | | | | | |
|---|---|---|---|---|---|---|---|---|---|
| GO_MODUI | GO_MODULATION_O | 45 | -0.591 | -1.8327 | 0.0039 | 0.194695 | 0.743 | 8229 | tags=49%, list=1 |
| GO_CARDI | GO_CARDIAC_MUSC | 228 | -0.4207 | -1.6979 | 0.0039 | 0.282111 | 0.946 | 12976 | tags=41%, list=2 |
| GO_DIGES | GO_DIGESTIVE_SYST | 145 | -0.4284 | -1.6318 | 0.004 | 0.312793 | 0.983 | 12111 | tags=37%, list=2 |
| GO_REGUL | GO_REGULATION_OI | 64 | -0.45 | -1.6374 | 0.0041 | 0.319673 | 0.981 | 15082 | tags=50%, list=2 |
| GO_ROOF_ | GO_ROOF_OF_MOUT | 90 | -0.49 | -1.7858 | 0.0041 | 0.234935 | 0.835 | 14543 | tags=49%, list=2 |
| GO_OUTFL | GO_OUTFLOW_TRAC | 80 | -0.5121 | -1.7826 | 0.0041 | 0.234632 | 0.841 | 16390 | tags=58%, list=2 |
| GO_DENDR | GO_DENDR Details ... | 15 | -0.6735 | -1.8651 | 0.0041 | 0.205078 | 0.668 | 12388 | tags=73%, list=2 |
| GO_COCHL | GO_COCHLEA_DEVE | 51 | -0.5053 | -1.7684 | 0.0041 | 0.23248 | 0.863 | 11028 | tags=43%, list=1 |
| GO_CGMP_ | GO_CGMP_METABOI | 15 | -0.6779 | -1.7508 | 0.0042 | 0.252474 | 0.88 | 2051 | tags=47%, list=4 |
| GO_AXIS_E | GO_AXIS_ELONGATI | 30 | -0.6015 | -1.7882 | 0.0042 | 0.233735 | 0.832 | 11983 | tags=50%, list=2 |
| GO_CALCIU | GO_CALCIUM_DEPEI | 43 | -0.5852 | -1.8064 | 0.0043 | 0.214348 | 0.799 | 4441 | tags=40%, list=8 |
| GO_REGUL | GO_REGULATION_OI | 29 | -0.5145 | -1.6349 | 0.0043 | 0.317368 | 0.982 | 20825 | tags=69%, list=3 |
| GO_TRABE | GO_TRABECULA_FOI | 25 | -0.6007 | -1.7683 | 0.0043 | 0.22932 | 0.863 | 8882 | tags=48%, list=1 |
| GO_NEGAT | GO_NEGATIVE_REGU | 15 | -0.642 | -1.7424 | 0.0057 | 0.262619 | 0.898 | 15514 | tags=60%, list=2 |
| GO_NEGAT | GO_NEGATIVE_REGU | 48 | -0.493 | -1.7408 | 0.0058 | 0.256503 | 0.9 | 3722 | tags=33%, list=6 |
| GO_NEGAT | GO_NEGATIVE_REGU | 34 | -0.5648 | -1.8268 | 0.0059 | 0.188771 | 0.757 | 8229 | tags=44%, list=1 |
| GO_CARDI | GO_CARDIAC_ATRIU | 34 | -0.5342 | -1.7324 | 0.0059 | 0.260933 | 0.915 | 17220 | tags=56%, list=2 |
| GO_HEART | GO_HEART_VALVE_ | 60 | -0.565 | -1.8074 | 0.0059 | 0.220867 | 0.798 | 11808 | tags=58%, list=2 |
| GO_NEPHR | GO_NEPHRIC_DUCT_ | 16 | -0.6321 | -1.7956 | 0.0059 | 0.229161 | 0.813 | 15942 | tags=75%, list=2 |
| GO_AXIS_S | GO_AXIS_SPECIFICA | 87 | -0.4587 | -1.7176 | 0.006 | 0.266782 | 0.928 | 14390 | tags=49%, list=2 |
| GO_ATRIAI | GO_ATRIAL_SEPTUM | 21 | -0.5714 | -1.701 | 0.006 | 0.276907 | 0.943 | 15184 | tags=57%, list=2 |
| GO_CORTIC | GO_CORTIC Details ... | 15 | -0.6926 | -1.8569 | 0.006 | 0.210484 | 0.686 | 7396 | tags=40%, list=1 |
| GO_NEGAT | GO_NEGATIVE_REGU | 45 | -0.4973 | -1.7414 | 0.006 | 0.258239 | 0.9 | 8426 | tags=36%, list=1 |
| GO_CARDI | GO_CARDIAC_MUSC | 67 | -0.4841 | -1.7943 | 0.006 | 0.227776 | 0.815 | 11823 | tags=40%, list=2 |
| GO_CALCIU | GO_CALCIUM_ION_T | 17 | -0.619 | -1.76 | 0.0061 | 0.241507 | 0.874 | 11495 | tags=35%, list=2 |
| GO_REGUL | GO_REGULATION_OI | 17 | -0.6018 | -1.7304 | 0.0061 | 0.262749 | 0.916 | 6841 | tags=35%, list=1 |
| GO_OUTFL | GO_OUTFL( Details ... | 27 | -0.6291 | -1.8508 | 0.0061 | 0.203708 | 0.697 | 15844 | tags=70%, list=2 |
| GO_PARAX | GO_PARAXIAL_MES( | 18 | -0.6327 | -1.781 | 0.0062 | 0.23079 | 0.845 | 11983 | tags=72%, list=2 |
| GO_EMBRY | GO_EMBRYONIC_AP | 123 | -0.4707 | -1.6815 | 0.0062 | 0.284407 | 0.958 | 8585 | tags=39%, list=1 |
| GO_CELLU | GO_CELLULAR_RESI | 21 | -0.6027 | -1.7701 | 0.0062 | 0.239303 | 0.86 | 806 | tags=24%, list=1 |
| GO_NEGAT | GO_NEGATIVE_REGU | 15 | -0.6051 | -1.677 | 0.0063 | 0.279086 | 0.961 | 8230 | tags=40%, list=1 |
| GO_HOMOI | GO_HOMOPHILIC_CE | 164 | -0.5014 | -1.8074 | 0.0064 | 0.216451 | 0.798 | 12572 | tags=48%, list=2 |
| GO_WNT_P | GO_WNT_PROTEIN_E | 30 | -0.6261 | -1.7821 | 0.0064 | 0.232098 | 0.843 | 12292 | tags=63%, list=2 |
| GO_TRACH | GO_TRACHEA_DEVE | 19 | -0.6283 | -1.757 | 0.0075 | 0.244656 | 0.875 | 6505 | tags=53%, list=1 |
| GO_REGUL | GO_REGULATION_OI | 86 | -0.4491 | -1.7228 | 0.0076 | 0.265901 | 0.925 | 12950 | tags=40%, list=2 |
| GO_DOPAM | GO_DOPAMINERGIC_ | 36 | -0.5597 | -1.7847 | 0.0076 | 0.233731 | 0.838 | 13955 | tags=58%, list=2 |
| GO_DENDR | GO_DENDRITE_MORI | 143 | -0.4489 | -1.6505 | 0.0079 | 0.308306 | 0.976 | 10004 | tags=38%, list=1 |
| GO_REGUL | GO_REGULATION_OI | 24 | -0.5207 | -1.6918 | 0.0079 | 0.283822 | 0.952 | 6841 | tags=29%, list=1 |
| GO_REGUL | GO_REGULATION_OI | 32 | -0.5006 | -1.6573 | 0.008 | 0.302435 | 0.973 | 7654 | tags=31%, list=1 |
| GO_MESEN | GO_MESENCHYME_I | 286 | -0.416 | -1.6368 | 0.008 | 0.31719 | 0.981 | 11983 | tags=42%, list=2 |
| GO_HEART | GO_HEART_FIELD_SI | 17 | -0.6184 | -1.7415 | 0.0082 | 0.261318 | 0.899 | 11983 | tags=59%, list=2 |
| GO_CGMP_ | GO_CGMP_MEDIATE | 27 | -0.5741 | -1.7216 | 0.0083 | 0.263013 | 0.925 | 4174 | tags=44%, list=7 |
| GO_CORON | GO_CORONARY_VAS | 17 | -0.64 | -1.721 | 0.0084 | 0.261434 | 0.925 | 8882 | tags=53%, list=1 |
| GO_REGUL | GO_REGULATION_OI | 41 | -0.518 | -1.7116 | 0.0085 | 0.270248 | 0.935 | 12788 | tags=44%, list=2 |
| GO_HEART | GO_HEART_GROWTH | 112 | -0.4297 | -1.6774 | 0.0095 | 0.2849 | 0.961 | 12950 | tags=39%, list=2 |
| GO_UROGE | GO_UROGENITAL_SY | 328 | -0.399 | -1.6481 | 0.0096 | 0.311945 | 0.977 | 8254 | tags=30%, list=1 |
| GO_EMBRY | GO_EMBRYONIC_DIG | 57 | -0.4844 | -1.7029 | 0.0097 | 0.277421 | 0.941 | 17220 | tags=54%, list=2 |
| GO_POSITI | GO_POSITIVE_REGUI | 63 | -0.449 | -1.6343 | 0.0098 | 0.316807 | 0.982 | 13702 | tags=46%, list=2 |
| GO_POSITI | GO_POSITIVE_REGUI | 25 | -0.5377 | -1.7121 | 0.0098 | 0.274478 | 0.934 | 7790 | tags=44%, list=1 |
| GO_POSITI | GO_POSITIVE_REGUI | 38 | -0.5358 | -1.7481 | 0.0098 | 0.255694 | 0.887 | 8876 | tags=39%, list=1 |
| GO_POSITI | GO_POSITIVE_REGUI | 27 | -0.5235 | -1.6748 | 0.0098 | 0.277639 | 0.963 | 16249 | tags=52%, list=2 |
| GO_REGUL | GO_REGULATION_OI | 44 | -0.4834 | -1.5901 | 0.01 | 0.360667 | 0.993 | 15424 | tags=45%, list=2 |
| GO_DEVEL | GO_DEVELOPMENTA | 36 | -0.5199 | -1.6888 | 0.0101 | 0.283722 | 0.953 | 11983 | tags=47%, list=2 |
| GO_NEURO | GO_NEURON_PROJE( | 59 | -0.5371 | -1.664 | 0.0101 | 0.292454 | 0.97 | 13008 | tags=58%, list=2 |
| GO_ARTER | GO_ARTERY_DEVEL( | 101 | -0.479 | -1.7058 | 0.0101 | 0.275843 | 0.939 | 10490 | tags=38%, list=1 |
| GO_SODIUI | GO_SODIUM_ION_TR | 174 | -0.3677 | -1.5024 | 0.0101 | 0.374713 | 0.999 | 11031 | tags=33%, list=1 |
| GO_POSITI | GO_POSITIVE_REGUI | 17 | -0.6051 | -1.7245 | 0.0102 | 0.264603 | 0.922 | 4975 | tags=47%, list=9 |
| GO_CARDI | GO_CARDIAC_ATRIU | 28 | -0.5579 | -1.7025 | 0.0102 | 0.275639 | 0.941 | 17220 | tags=61%, list=2 |

| | | | | | | | | | |
|---|---|---|---|---|---|---|---|---|---|
| GO_MATIN | GO_MATING_BEHAV | 23 | -0.5307 | -1.6938 | 0.0102 | 0.287106 | 0.951 | 7870 | tags=35%, list=1 |
| GO_FACE_I | GO_FACE_DEVELOPI | 48 | -0.5156 | -1.7121 | 0.0102 | 0.271734 | 0.934 | 10943 | tags=46%, list=1 |
| GO_SYMPA | GO_SYMPATHETIC_N | 21 | -0.6084 | -1.6771 | 0.0103 | 0.281119 | 0.961 | 7998 | tags=52%, list=1 |
| GO_REGUL | GO_REGULATION_OI | 115 | -0.4143 | -1.6335 | 0.0115 | 0.316891 | 0.982 | 13702 | tags=42%, list=2 |
| GO_POSITI | GO_POSITIVE_REGUI | 83 | -0.4408 | -1.6527 | 0.0116 | 0.307354 | 0.975 | 12950 | tags=41%, list=2 |
| GO_RESPIR | GO_RESPIRATORY_S | 201 | -0.4137 | -1.5958 | 0.0117 | 0.357493 | 0.992 | 10951 | tags=36%, list=1 |
| GO_RETINA | GO_RETINA_DEVELC | 147 | -0.4471 | -1.6772 | 0.0118 | 0.283072 | 0.961 | 15415 | tags=49%, list=2 |
| GO_HEAD_ | GO_HEAD_MORPHOC | 36 | -0.5434 | -1.7174 | 0.0118 | 0.264449 | 0.928 | 17220 | tags=61%, list=2 |
| GO_REGUL | GO_REGULATION_OI | 101 | -0.436 | -1.6758 | 0.0119 | 0.277536 | 0.961 | 12950 | tags=40%, list=2 |
| GO_RESPIR | GO_RESPIRATORY_S | 32 | -0.5173 | -1.6209 | 0.0119 | 0.324505 | 0.985 | 16720 | tags=69%, list=2 |
| GO_BETA_( | GO_BETA_CATENIN_ | 84 | -0.503 | -1.7806 | 0.012 | 0.227891 | 0.845 | 8878 | tags=38%, list=1 |
| GO_PHOSPI | GO_PHOSPHATASE_I | 45 | -0.4349 | -1.5872 | 0.0121 | 0.362569 | 0.993 | 12823 | tags=47%, list=2 |
| GO_NEURO | GO_NEUROMUSCULA | 76 | -0.4719 | -1.6335 | 0.0121 | 0.314964 | 0.982 | 10655 | tags=46%, list=1 |
| GO_EMBRY | GO_EMBRYONIC_PLA | 24 | -0.5259 | -1.6382 | 0.0123 | 0.319739 | 0.98 | 10234 | tags=50%, list=1 |
| GO_MUSCL | GO_MUSCLE_ORGAN | 83 | -0.4621 | -1.6764 | 0.0125 | 0.278386 | 0.961 | 10943 | tags=42%, list=1 |
| GO_CELL_N | GO_CELL_MIGRATIO | 18 | -0.6241 | -1.7377 | 0.0126 | 0.254365 | 0.905 | 15503 | tags=56%, list=2 |
| GO_POSTSY | GO_POSTSYNAPTIC_ | 116 | -0.4878 | -1.5721 | 0.0127 | 0.372215 | 0.993 | 15009 | tags=54%, list=2 |
| GO_POSITI | GO_POSITIVE_REGUI | 43 | -0.5075 | -1.6301 | 0.0135 | 0.313147 | 0.983 | 8907 | tags=37%, list=1 |
| GO_POSITI | GO_POSITIVE_REGUI | 108 | -0.3967 | -1.5495 | 0.0136 | 0.37951 | 0.995 | 15514 | tags=46%, list=2 |
| GO_REGUL | GO_REGULATION_OI | 44 | -0.4935 | -1.63 | 0.0136 | 0.311661 | 0.983 | 12067 | tags=41%, list=2 |
| GO_NEGAT | GO_NEGATIVE_REGU | 27 | -0.5322 | -1.6928 | 0.0137 | 0.283929 | 0.952 | 8229 | tags=37%, list=1 |
| GO_EMBRY | GO_EMBRYONIC_FO | 32 | -0.5161 | -1.6364 | 0.0138 | 0.316016 | 0.981 | 8338 | tags=50%, list=1 |
| GO_EMBRY | GO_EMBRYONIC_DIG | 18 | -0.6458 | -1.7786 | 0.0139 | 0.224678 | 0.847 | 4739 | tags=44%, list=8 |
| GO_MESEN | GO_MESENCHYMAL_ | 44 | -0.5281 | -1.7258 | 0.014 | 0.264606 | 0.92 | 10943 | tags=52%, list=1 |
| GO_SMOOT | GO_SMOOTH_MUSCL | 68 | -0.4953 | -1.6673 | 0.014 | 0.288944 | 0.969 | 9318 | tags=37%, list=1 |
| GO_REGUL | GO_REGULATION_OI | 105 | -0.442 | -1.6184 | 0.0141 | 0.32706 | 0.986 | 11448 | tags=42%, list=2 |
| GO_REGUL | GO_REGULATION_OI | 34 | -0.5269 | -1.6194 | 0.0142 | 0.326285 | 0.986 | 7155 | tags=38%, list=1 |
| GO_MORPH | GO_MORPHOGENESI | 25 | -0.527 | -1.6398 | 0.0143 | 0.319974 | 0.98 | 18351 | tags=64%, list=3 |
| GO_SODIUI | GO_SODIUM_ION_TR | 240 | -0.3382 | -1.4221 | 0.0143 | 0.385582 | 1 | 11099 | tags=31%, list=1 |
| GO_POSTSY | GO_POSTSYNAPTIC_ | 275 | -0.4513 | -1.6157 | 0.0143 | 0.328052 | 0.989 | 15009 | tags=52%, list=2 |
| GO_POSTSY | GO_POSTSYNAPTIC_ | 34 | -0.5496 | -1.5925 | 0.0143 | 0.361946 | 0.993 | 6442 | tags=41%, list=1 |
| GO_SYNAP | GO_SYNAPTIC_TRAN | 47 | -0.5372 | -1.6836 | 0.0143 | 0.286265 | 0.955 | 12500 | tags=51%, list=2 |
| GO_SPINAL | GO_SPINAL_CORD_D | 109 | -0.4704 | -1.5881 | 0.0144 | 0.362022 | 0.993 | 17220 | tags=54%, list=2 |
| GO_EMBRY | GO_EMBRYONIC_HIN | 29 | -0.5592 | -1.6887 | 0.0144 | 0.281409 | 0.953 | 8585 | tags=52%, list=1 |
| GO_POSTSY | GO_POSTSYNAPTIC_ | 90 | -0.4907 | -1.5833 | 0.0149 | 0.359687 | 0.993 | 13214 | tags=51%, list=2 |
| GO_DENDR | GO_DENDRITIC_SPIN | 101 | -0.4523 | -1.6152 | 0.0151 | 0.32559 | 0.989 | 10263 | tags=38%, list=1 |
| GO_SODIUI | GO_SODIUM_ION_TR | 152 | -0.3711 | -1.4906 | 0.0154 | 0.371473 | 0.999 | 16280 | tags=44%, list=2 |
| GO_NEURO | GO_NEURON_TO_NE | 358 | -0.4123 | -1.6453 | 0.0156 | 0.312745 | 0.978 | 13214 | tags=43%, list=2 |
| GO_RNAI_E | GO_RNAI_EFFECTOR | 16 | -0.6902 | -1.6876 | 0.016 | 0.281546 | 0.953 | 4956 | tags=44%, list=8 |
| GO_TRANS | GO_TRANSMEMBRAI | 79 | -0.4885 | -1.7035 | 0.0161 | 0.27862 | 0.941 | 13311 | tags=49%, list=2 |
| GO_TRANS | GO_TRANSMEMBRAI | 61 | -0.4735 | -1.6231 | 0.0162 | 0.321097 | 0.985 | 13245 | tags=49%, list=2 |
| GO_PERICA | GO_PERICARDIUM_D | 20 | -0.5699 | -1.6964 | 0.0162 | 0.283191 | 0.947 | 11652 | tags=65%, list=2 |
| GO_METEN | GO_METENCEPHALC | 110 | -0.4563 | -1.6418 | 0.0164 | 0.316966 | 0.98 | 11744 | tags=44%, list=2 |
| GO_CELL_I | GO_CELL_PROLIFER | 28 | -0.5518 | -1.6101 | 0.0164 | 0.329333 | 0.989 | 15207 | tags=61%, list=2 |
| GO_SECON | GO_SECONDARY_PA | 24 | -0.5656 | -1.6561 | 0.0165 | 0.303069 | 0.974 | 12664 | tags=63%, list=2 |
| GO_HINDLI | GO_HINDLIMB_MOR | 37 | -0.5268 | -1.6328 | 0.0168 | 0.312615 | 0.982 | 8585 | tags=49%, list=1 |
| GO_MIDBR | GO_MIDBRAIN_DOPA | 17 | -0.6031 | -1.7267 | 0.0174 | 0.265512 | 0.919 | 10943 | tags=53%, list=1 |
| GO_HINDBI | GO_HINDBRAIN_DEV | 152 | -0.4252 | -1.5774 | 0.0177 | 0.36581 | 0.993 | 12566 | tags=42%, list=2 |
| GO_RESPOI | GO_RESPONSE_TO_P | 24 | -0.5437 | -1.6811 | 0.0178 | 0.282889 | 0.959 | 3744 | tags=33%, list=6 |
| GO_BODY_ | GO_BODY_MORPHOC | 49 | -0.5211 | -1.7101 | 0.0178 | 0.268443 | 0.937 | 8248 | tags=39%, list=1 |
| GO_POSITI | GO_POSITIVE_REGUI | 26 | -0.5912 | -1.7369 | 0.0179 | 0.252992 | 0.905 | 9318 | tags=42%, list=1 |
| GO_NEGAT | GO_NEGATIVE_REGU | 309 | -0.3924 | -1.563 | 0.0179 | 0.376859 | 0.994 | 8823 | tags=35%, list=1 |
| GO_CLATH | GO_CLATHRIN_COAT | 71 | -0.4355 | -1.5789 | 0.0179 | 0.365406 | 0.993 | 12509 | tags=51%, list=2 |
| GO_MAINT | GO_MAINTENANCE_ | 34 | -0.5409 | -1.6776 | 0.018 | 0.286673 | 0.961 | 7372 | tags=41%, list=1 |
| GO_CARDI | GO_CARDIAC_MUSC | 124 | -0.3991 | -1.5307 | 0.018 | 0.388098 | 0.996 | 12950 | tags=39%, list=2 |
| GO_VASCU | GO_VASCULAR_ASS | 46 | -0.5352 | -1.6904 | 0.0185 | 0.284758 | 0.953 | 9318 | tags=35%, list=1 |
| GO_REGUL | GO_REGULATION_OI | 21 | -0.6021 | -1.6791 | 0.0185 | 0.285247 | 0.959 | 3847 | tags=43%, list=7 |
| GO_POSTSY | GO_POSTSYNAPTIC_ | 21 | -0.6394 | -1.633 | 0.0186 | 0.314217 | 0.982 | 6442 | tags=52%, list=1 |

| | | | | | | | | | |
|---|---|---|---|---|---|---|---|---|---|
| GO_ORGAN | GO_ORGAN_GROWTI | 188 | -0.4021 | -1.6143 | 0.0187 | 0.325959 | 0.989 | 13328 | tags=39%, list=2 |
| GO_PROTEI | GO_PROTEIN_DNA_C | 18 | -0.6365 | -1.6819 | 0.0191 | 0.285637 | 0.958 | 3233 | tags=39%, list=6 |
| GO_TRANS | GO_TRANSLATION_F | 26 | -0.5795 | -1.6824 | 0.0195 | 0.286854 | 0.957 | 5527 | tags=38%, list=9 |
| GO_STEROI | GO_STEROL_BIOSYN | 79 | -0.4673 | -1.5851 | 0.0197 | 0.364205 | 0.993 | 4065 | tags=23%, list=7 |
| GO_CEREB | GO_CEREBELLAR_CO | 31 | -0.5569 | -1.6857 | 0.0198 | 0.283636 | 0.953 | 11744 | tags=52%, list=2 |
| GO_METAN | GO_METANEPHROS_ | 91 | -0.4628 | -1.6558 | 0.02 | 0.301572 | 0.975 | 9318 | tags=40%, list=1 |
| GO_CYCLIC | GO_CYCLIC_NUCLEO | 25 | -0.5466 | -1.6435 | 0.0201 | 0.314872 | 0.978 | 3593 | tags=32%, list=6 |
| GO_SOLUT | GO_SOLUTE_CATION | 98 | -0.3774 | -1.4726 | 0.0201 | 0.376564 | 0.999 | 16260 | tags=46%, list=2 |
| GO_CARDIO | GO_CARDIOCYTE_DI | 154 | -0.3988 | -1.5578 | 0.0202 | 0.379201 | 0.995 | 18260 | tags=49%, list=3 |
| GO_NEGAT | GO_NEGATIVE_REGU | 73 | -0.4477 | -1.5613 | 0.0203 | 0.378072 | 0.995 | 11596 | tags=41%, list=2 |
| GO_PHOTO | GO_PHOTORECEPTO | 16 | -0.6205 | -1.6701 | 0.0214 | 0.28439 | 0.966 | 18221 | tags=63%, list=3 |
| GO_NEGAT | GO_NEGATIVE_REGU | 330 | -0.3711 | -1.5224 | 0.0216 | 0.37683 | 0.997 | 8823 | tags=34%, list=1 |
| GO_REGUL | GO_REGULATION_OI | 249 | -0.3839 | -1.5608 | 0.0217 | 0.377816 | 0.995 | 13802 | tags=40%, list=2 |
| GO_RESPO | GO_RESPONSE_TO_B | 164 | -0.4014 | -1.5542 | 0.0217 | 0.381876 | 0.995 | 14016 | tags=42%, list=2 |
| GO_MESON | GO_MESONEPHROS_ | 103 | -0.4365 | -1.6385 | 0.0217 | 0.321175 | 0.98 | 8585 | tags=34%, list=1 |
| GO_CELLU | GO_CELLULAR_RESP | 26 | -0.5298 | -1.6316 | 0.0222 | 0.311404 | 0.983 | 1071 | tags=23%, list=2 |
| GO_NEURO | GO_NEUROTRANSMI | 19 | -0.5524 | -1.5846 | 0.0223 | 0.361706 | 0.993 | 17190 | tags=68%, list=2 |
| GO_SECRE | GO_SECRETORY_GRA | 15 | -0.6648 | -1.6288 | 0.0224 | 0.312399 | 0.983 | 5721 | tags=47%, list=1 |
| GO_CELL_I | GO_CELL_BODY_ME | 31 | -0.5585 | -1.6079 | 0.0226 | 0.332873 | 0.99 | 14731 | tags=68%, list=2 |
| GO_SPINAL | GO_SPINAL_CORD_P | 24 | -0.5745 | -1.5807 | 0.0226 | 0.362632 | 0.993 | 16549 | tags=63%, list=2 |
| GO_REGUL | GO_REGULATION_OI | 47 | -0.5132 | -1.6746 | 0.0227 | 0.276026 | 0.963 | 9869 | tags=40%, list=1 |
| GO_CAMEF | GO_CAMERA_TYPE_ | 329 | -0.3625 | -1.4993 | 0.0228 | 0.368564 | 0.999 | 15683 | tags=43%, list=2 |
| GO_EPITHE | GO_EPITHELIAL_TUE | 132 | -0.413 | -1.5783 | 0.0228 | 0.365246 | 0.993 | 10943 | tags=39%, list=1 |
| GO_EMBRY | GO_EMBRYONIC_PA | 69 | -0.426 | -1.5847 | 0.023 | 0.363291 | 0.993 | 12828 | tags=43%, list=2 |
| GO_POSITI | GO_POSITIVE_REGUI | 48 | -0.454 | -1.5844 | 0.0236 | 0.360437 | 0.993 | 18362 | tags=52%, list=3 |
| GO_KIDNE | GO_KIDNEY_EPITHE | 142 | -0.4243 | -1.6244 | 0.024 | 0.319798 | 0.985 | 7643 | tags=32%, list=1 |
| GO_PEPTID | GO_PEPTIDE_HORMO | 50 | -0.4687 | -1.5649 | 0.0242 | 0.376976 | 0.994 | 20263 | tags=62%, list=3 |
| GO_RESPO | GO_RESPONSE_TO_C | 36 | -0.4759 | -1.5428 | 0.0242 | 0.37839 | 0.996 | 8443 | tags=33%, list=1 |
| GO_WNT_S | GO_WNT_SIGNALING | 38 | -0.4995 | -1.6133 | 0.0243 | 0.326815 | 0.989 | 12463 | tags=53%, list=2 |
| GO_ADULT | GO_ADULT_BEHAVIO | 135 | -0.445 | -1.5853 | 0.0245 | 0.365517 | 0.993 | 15699 | tags=50%, list=2 |
| GO_COPI_C | GO_COPI_COATED_V | 27 | -0.6025 | -1.729 | 0.0249 | 0.263145 | 0.916 | 7711 | tags=37%, list=1 |
| GO_REGUL | GO_REGULATION_OI | 33 | -0.5048 | -1.6372 | 0.0258 | 0.318085 | 0.981 | 5634 | tags=39%, list=1 |
| GO_PLACEI | GO_PLACENTA_BLOO | 34 | -0.4513 | -1.5407 | 0.0262 | 0.380603 | 0.996 | 10185 | tags=44%, list=1 |
| GO_SCHWA | GO_SCHWANN_CELL | 38 | -0.4962 | -1.6472 | 0.0262 | 0.312183 | 0.977 | 15394 | tags=50%, list=2 |
| GO_APPEN | GO_APPENDAGE_MO | 145 | -0.4343 | -1.5684 | 0.0264 | 0.375026 | 0.994 | 8807 | tags=37%, list=1 |
| GO_NEGAT | GO_NEGATIVE_REGU | 28 | -0.4974 | -1.5531 | 0.0264 | 0.381506 | 0.995 | 8426 | tags=39%, list=1 |
| GO_CELLU | GO_CELLULAR_RESP | 16 | -0.5868 | -1.6156 | 0.0265 | 0.326286 | 0.989 | 3347 | tags=38%, list=6 |
| GO_SOMITI | GO_SOMITE_DEVELO | 88 | -0.4226 | -1.5587 | 0.0265 | 0.378348 | 0.995 | 6069 | tags=28%, list=1 |
| GO_PANCR | GO_PANCREAS_DEV | 78 | -0.4425 | -1.5103 | 0.0268 | 0.37477 | 0.999 | 11638 | tags=45%, list=2 |
| GO_CELL_C | GO_CELL_CELL_ADH | 270 | -0.3962 | -1.538 | 0.0271 | 0.382841 | 0.996 | 12572 | tags=40%, list=2 |
| GO_OVULA | GO_OVULATION_CY | 47 | -0.453 | -1.5473 | 0.0274 | 0.380378 | 0.996 | 20688 | tags=62%, list=3 |
| GO_NEURA | GO_NEURAL_TUBE_I | 161 | -0.4123 | -1.547 | 0.0276 | 0.379553 | 0.996 | 10943 | tags=38%, list=1 |
| GO_REGUL | GO_REGULATION_OI | 347 | -0.3549 | -1.4692 | 0.0276 | 0.376559 | 0.999 | 13369 | tags=37%, list=2 |
| GO_MORPH | GO_MORPHOGENESI | 16 | -0.5837 | -1.5714 | 0.0282 | 0.372458 | 0.993 | 7643 | tags=50%, list=1 |
| GO_VASCU | GO_VASCULOGENES | 81 | -0.4784 | -1.6597 | 0.0283 | 0.298575 | 0.971 | 10247 | tags=44%, list=1 |
| GO_SMAD_ | GO_SMAD_PROTEIN_ | 76 | -0.4179 | -1.5315 | 0.0284 | 0.390527 | 0.996 | 18362 | tags=53%, list=3 |
| GO_NEGAT | GO_NEGATIVE_REGU | 21 | -0.5206 | -1.5681 | 0.0286 | 0.373968 | 0.994 | 8882 | tags=48%, list=1 |
| GO_DENDR | GO_DENDRITE_DEVE | 244 | -0.418 | -1.5886 | 0.0287 | 0.362631 | 0.993 | 10263 | tags=37%, list=1 |
| GO_POSITI | GO_POSITIVE_REGUI | 372 | -0.3857 | -1.5142 | 0.0288 | 0.375148 | 0.999 | 12629 | tags=38%, list=2 |
| GO_POSITI | GO_POSITIVE_REGUI | 28 | -0.4718 | -1.5288 | 0.029 | 0.384363 | 0.997 | 10307 | tags=39%, list=1 |
| GO_EMBRY | GO_EMBRYONIC_AX | 38 | -0.4416 | -1.5123 | 0.0291 | 0.37611 | 0.999 | 13642 | tags=45%, list=2 |
| GO_INTRIN | GO_INTRINSIC_COMI | 120 | -0.4771 | -1.5412 | 0.0295 | 0.380746 | 0.996 | 14870 | tags=52%, list=2 |
| GO_ADHER | GO_ADHERENS_JUNO | 61 | -0.4666 | -1.5948 | 0.0296 | 0.357948 | 0.992 | 11122 | tags=39%, list=1 |
| GO_CALCIU | GO_CALCIUM_CHAN | 63 | -0.4547 | -1.5463 | 0.0296 | 0.379993 | 0.996 | 10331 | tags=37%, list=1 |
| GO_REGUL | GO_REGULATION_OI | 52 | -0.5011 | -1.583 | 0.0296 | 0.35848 | 0.993 | 10263 | tags=50%, list=1 |
| GO_PEROX | GO_PEROXISOME_PI | 21 | -0.5168 | -1.6068 | 0.0297 | 0.333779 | 0.99 | 9464 | tags=43%, list=1 |
| GO_BETA_C | GO_BETA_CATENIN_ | 21 | -0.5482 | -1.6124 | 0.0298 | 0.327325 | 0.989 | 10834 | tags=48%, list=1 |
| GO_SEGME | GO_SEGMENTATION | 99 | -0.3861 | -1.4803 | 0.0298 | 0.373199 | 0.999 | 15942 | tags=44%, list=2 |

| | | | | | | | | | |
|---|---|---|---|---|---|---|---|---|---|
| GO_VENTR | GO_VENTRICULAR_S | 32 | -0.4956 | -1.5252 | 0.0298 | 0.375494 | 0.997 | 7111 | tags=31%, list=1 |
| GO_MAINT | GO_MAINTENANCE_ | 163 | -0.4096 | -1.5441 | 0.0299 | 0.382495 | 0.996 | 8726 | tags=31%, list=1 |
| GO_CHROM | GO_CHROMATIN_DIS | 18 | -0.6266 | -1.617 | 0.0301 | 0.328693 | 0.987 | 3233 | tags=39%, list=6 |
| GO_NUCLE | GO_NUCLEUS_LOCA | 28 | -0.5757 | -1.6102 | 0.0309 | 0.330886 | 0.989 | 6486 | tags=39%, list=1 |
| GO_TRANS | GO_TRANSPORTER_ | 324 | -0.4115 | -1.5319 | 0.0312 | 0.392375 | 0.996 | 14144 | tags=42%, list=2 |
| GO_NEURO | GO_NEUROMUSCULA | 49 | -0.4583 | -1.5518 | 0.0315 | 0.378383 | 0.995 | 10655 | tags=43%, list=1 |
| GO_POSITI | GO_POSITIVE_REGU | 185 | -0.3816 | -1.5053 | 0.0316 | 0.374772 | 0.999 | 13067 | tags=38%, list=2 |
| GO_MESOD | GO_MESODERMAL_C | 35 | -0.4666 | -1.5271 | 0.0323 | 0.377535 | 0.997 | 5704 | tags=31%, list=1 |
| GO_DENDR | GO_DENDRITIC_SPIN | 61 | -0.4538 | -1.5428 | 0.0324 | 0.381283 | 0.996 | 9869 | tags=34%, list=1 |
| GO_NEURO | GO_NEURON_RECOG | 50 | -0.4769 | -1.5906 | 0.0324 | 0.362842 | 0.993 | 8082 | tags=34%, list=1 |
| GO_FRIZZL | GO_FRIZZLED_BINDI | 38 | -0.4639 | -1.517 | 0.0324 | 0.379938 | 0.998 | 11983 | tags=42%, list=2 |
| GO_MESON | GO_MESONEPHRIC_T | 66 | -0.4314 | -1.529 | 0.0327 | 0.386541 | 0.996 | 5634 | tags=29%, list=1 |
| GO_PRESYI | GO_PRESYNAPTIC_M | 15 | -0.6198 | -1.5562 | 0.0329 | 0.381544 | 0.995 | 6730 | tags=40%, list=1 |
| GO_INTRIN | GO_INTRINSIC_COMI | 163 | -0.4531 | -1.5 | 0.0331 | 0.36916 | 0.999 | 11122 | tags=43%, list=1 |
| GO_EXCITA | GO_EXCITATORY_SY | 26 | -0.5277 | -1.5285 | 0.0332 | 0.382209 | 0.997 | 6442 | tags=42%, list=1 |
| GO_RIBOSO | GO_RIBOSOMAL_SM | 17 | -0.6188 | -1.6646 | 0.0332 | 0.293132 | 0.97 | 5882 | tags=24%, list=1 |
| GO_EPITHE | GO_EPITHELIAL_TO_ | 148 | -0.4151 | -1.5761 | 0.0333 | 0.36732 | 0.993 | 12664 | tags=42%, list=2 |
| GO_REGUL | GO_REGULATION_OI | 50 | -0.5124 | -1.6165 | 0.0333 | 0.327977 | 0.987 | 4065 | tags=26%, list=7 |
| GO_INTRIN | GO_INTRINSIC_COMI | 52 | -0.4845 | -1.5084 | 0.0333 | 0.37218 | 0.999 | 14870 | tags=52%, list=2 |
| GO_NEURO | GO_NEUROTROPHIN_ | 39 | -0.4614 | -1.5059 | 0.0335 | 0.374604 | 0.999 | 7373 | tags=38%, list=1 |
| GO_SYNAP | GO_SYNAPSE_ORGA | 422 | -0.3813 | -1.5008 | 0.0336 | 0.373816 | 0.999 | 11448 | tags=37%, list=2 |
| GO_MONOV | GO_MONOVALENT_I | 361 | -0.3371 | -1.4204 | 0.0337 | 0.384857 | 1 | 15786 | tags=40%, list=2 |
| GO_REGUL | GO_REGULATION_OI | 188 | -0.4179 | -1.5264 | 0.0341 | 0.377724 | 0.997 | 9188 | tags=39%, list=1 |
| GO_SOMITO | GO_SOMITOGENESIS | 69 | -0.4144 | -1.508 | 0.0341 | 0.371905 | 0.999 | 6069 | tags=28%, list=1 |
| GO_REPRO | GO_REPRODUCTIVE_ | 30 | -0.4604 | -1.5601 | 0.0343 | 0.378045 | 0.995 | 7870 | tags=30%, list=1 |
| GO_SENSO | GO_SENSORY_SYSTE | 391 | -0.3485 | -1.4389 | 0.0346 | 0.382381 | 1 | 15699 | tags=44%, list=2 |
| GO_SCHWA | GO_SCHWANN_CELL | 30 | -0.4949 | -1.5709 | 0.0346 | 0.371963 | 0.993 | 15394 | tags=53%, list=2 |
| GO_POSITI | GO_POSITIVE_REGU | 40 | -0.4682 | -1.5118 | 0.0348 | 0.375911 | 0.999 | 13631 | tags=43%, list=2 |
| GO_REGUL | GO_REGULATION_OI | 18 | -0.5795 | -1.5917 | 0.0349 | 0.361865 | 0.993 | 9771 | tags=50%, list=1 |
| GO_SCHAF | GO_SCHAFFER_COLI | 83 | -0.4225 | -1.5003 | 0.0351 | 0.371716 | 0.999 | 14369 | tags=42%, list=2 |
| GO_INTRIN | GO_INTRINSIC_COMI | 75 | -0.4842 | -1.4908 | 0.0357 | 0.371938 | 0.999 | 11028 | tags=45%, list=1 |
| GO_REGUL | GO_REGULATION_OI | 36 | -0.4625 | -1.5088 | 0.0359 | 0.37372 | 0.999 | 15888 | tags=53%, list=2 |
| GO_DORSA | GO_DORSAL_VENTR | 92 | -0.4179 | -1.4916 | 0.0359 | 0.371296 | 0.999 | 17220 | tags=49%, list=2 |
| GO_REGUL | GO_REGULATION_OI | 65 | -0.4121 | -1.463 | 0.0359 | 0.383785 | 0.999 | 12950 | tags=37%, list=2 |
| GO_REGUL | GO_REGULATION_OI | 32 | -0.4806 | -1.553 | 0.0359 | 0.380316 | 0.995 | 15514 | tags=44%, list=2 |
| GO_CARDIA | GO_CARDIAC_MYOF | 19 | -0.5787 | -1.5644 | 0.0361 | 0.376372 | 0.994 | 17984 | tags=68%, list=3 |
| GO_CYCLIC | GO_CYCLIC_NUCLEO | 20 | -0.5597 | -1.5479 | 0.0362 | 0.380407 | 0.996 | 19084 | tags=75%, list=3 |
| GO_EMBRY | GO_EMBRYONIC_CA | 28 | -0.5154 | -1.5736 | 0.0363 | 0.370075 | 0.993 | 13841 | tags=54%, list=2 |
| GO_RECEPT | GO_RECEPTOR_INTE | 113 | -0.3934 | -1.457 | 0.0363 | 0.386055 | 1 | 8230 | tags=35%, list=1 |
| GO_MORPH | GO_MORPHOGENESI | 150 | -0.3888 | -1.5194 | 0.0365 | 0.376702 | 0.998 | 10943 | tags=37%, list=1 |
| GO_STEM_ | GO_STEM_CELL_PRO | 126 | -0.3823 | -1.4691 | 0.0368 | 0.375916 | 0.999 | 11374 | tags=33%, list=1 |
| GO_CATEN | GO_CATENIN_COMPI | 31 | -0.5346 | -1.5904 | 0.0369 | 0.361601 | 0.993 | 5308 | tags=32%, list=9 |
| GO_NEGAT | GO_NEGATIVE_REGU | 23 | -0.5081 | -1.5517 | 0.0373 | 0.377142 | 0.995 | 22837 | tags=70%, list=3 |
| GO_ARF_PI | GO_ARF_PROTEIN_SI | 18 | -0.5521 | -1.5981 | 0.0373 | 0.353334 | 0.992 | 12242 | tags=61%, list=2 |
| GO_CORON | GO_CORONARY_VAS | 49 | -0.5091 | -1.6253 | 0.0375 | 0.319585 | 0.985 | 10888 | tags=39%, list=1 |
| GO_RESPO | GO_RESPONSE_TO_C | 47 | -0.424 | -1.4765 | 0.0375 | 0.378663 | 0.999 | 6841 | tags=28%, list=1 |
| GO_EMBRY | GO_EMBRYONIC_EY | 37 | -0.478 | -1.5271 | 0.0376 | 0.378846 | 0.997 | 10943 | tags=46%, list=1 |
| GO_METAL | GO_METAL_ION_TRA | 426 | -0.3486 | -1.4233 | 0.0377 | 0.385554 | 1 | 16280 | tags=44%, list=2 |
| GO_PULMO | GO_PULMONARY_VA | 17 | -0.6237 | -1.5528 | 0.0381 | 0.377614 | 0.995 | 15184 | tags=65%, list=2 |
| GO_MATIN | GO_MATING | 41 | -0.4088 | -1.4234 | 0.0382 | 0.386168 | 1 | 9389 | tags=32%, list=1 |
| GO_NEURO | GO_NEURON_PROJEC | 91 | -0.4086 | -1.4892 | 0.0382 | 0.370398 | 0.999 | 10114 | tags=35%, list=1 |
| GO_PERIPH | GO_PERIPHERAL_NE | 74 | -0.4062 | -1.4828 | 0.0382 | 0.373657 | 0.999 | 8858 | tags=35%, list=1 |
| GO_CARDIA | GO_CARDIAC_CELL_ | 88 | -0.4114 | -1.4897 | 0.0383 | 0.371408 | 0.999 | 12950 | tags=39%, list=2 |
| GO_GAMM | GO_GAMMA_AMINO | 16 | -0.5455 | -1.5256 | 0.0384 | 0.375802 | 0.997 | 15095 | tags=75%, list=2 |
| GO_ANTER | GO_ANTERIOR_POST | 51 | -0.4309 | -1.5118 | 0.0386 | 0.373611 | 0.999 | 17335 | tags=55%, list=3 |
| GO_CARDIA | GO_CARDIAC_COND | 146 | -0.4104 | -1.4946 | 0.0387 | 0.37066 | 0.999 | 16182 | tags=45%, list=2 |
| GO_FOREB | GO_FOREBRAIN_NEU | 26 | -0.515 | -1.5555 | 0.039 | 0.381846 | 0.995 | 11013 | tags=50%, list=1 |
| GO_BONE_ | GO_BONE_MORPHO | 92 | -0.4374 | -1.4949 | 0.039 | 0.372163 | 0.999 | 16214 | tags=54%, list=2 |

| | | | | | | | | | |
|---|---|---|---|---|---|---|---|---|---|
| GO_FOREB | GO_FOREBRAIN_DEV | 384 | -0.385 | -1.4746 | 0.0394 | 0.377876 | 0.999 | 15429 | tags=45%, list=2 |
| GO_MESEN | GO_MESENCHYMAL_ | 225 | -0.3979 | -1.5546 | 0.0396 | 0.382532 | 0.995 | 11983 | tags=41%, list=2 |
| GO_REGUL | GO_REGULATION_OI | 23 | -0.538 | -1.521 | 0.0396 | 0.376594 | 0.997 | 19852 | tags=74%, list=3 |
| GO_REGUL | GO_REGULATION_OI | 105 | -0.3798 | -1.4168 | 0.0396 | 0.384233 | 1 | 13991 | tags=41%, list=2 |
| GO_NEURA | GO_NEURAL_CREST_ | 89 | -0.3974 | -1.4761 | 0.0401 | 0.37846 | 0.999 | 15503 | tags=48%, list=2 |
| GO_CATIOI | GO_CATION_CHANN | 216 | -0.4279 | -1.4988 | 0.0409 | 0.368579 | 0.999 | 15172 | tags=44%, list=2 |
| GO_CRANI | GO_CRANIOFACIAL_ | 17 | -0.5953 | -1.5656 | 0.0412 | 0.376641 | 0.994 | 11834 | tags=59%, list=2 |
| GO_PHARY | GO_PHARYNGEAL_S | 25 | -0.5129 | -1.5259 | 0.0412 | 0.37643 | 0.997 | 15096 | tags=48%, list=2 |
| GO_CHEMC | GO_CHEMOSENSORY | 17 | -0.5547 | -1.5198 | 0.0413 | 0.376896 | 0.998 | 8980 | tags=47%, list=1 |
| GO_RNA_P | GO_RNA_POLYMERA | 55 | -0.4505 | -1.5134 | 0.0413 | 0.374718 | 0.999 | 5294 | tags=27%, list=9 |
| GO_PATTEI | GO_PATTERN_SPECI | 452 | -0.3474 | -1.4356 | 0.0413 | 0.383824 | 1 | 17224 | tags=44%, list=2 |
| GO_RENAL | GO_RENAL_TUBULE_ | 95 | -0.3892 | -1.4612 | 0.0413 | 0.384978 | 0.999 | 13137 | tags=40%, list=2 |
| GO_SOLUT | GO_SOLUTE_SODIUM | 72 | -0.3816 | -1.4069 | 0.0414 | 0.382752 | 1 | 18772 | tags=50%, list=3 |
| GO_RENAL | GO_RENAL_WATER_ | 37 | -0.4654 | -1.5369 | 0.0415 | 0.38396 | 0.996 | 15488 | tags=51%, list=2 |
| GO_NEGAT | GO_NEGATIVE_REGU | 219 | -0.3797 | -1.4952 | 0.0415 | 0.374587 | 0.999 | 8823 | tags=35%, list=1 |
| GO_MULTI | GO_MULTICELLULAI | 53 | -0.4019 | -1.4761 | 0.0416 | 0.377564 | 0.999 | 12152 | tags=40%, list=2 |
| GO_CELL_I | GO_CELL_MIGRATIO | 15 | -0.5884 | -1.495 | 0.0417 | 0.372927 | 0.999 | 1749 | tags=33%, list=3 |
| GO_CARTII | GO_CARTILAGE_DEV | 186 | -0.4235 | -1.5534 | 0.0417 | 0.38255 | 0.995 | 8947 | tags=32%, list=1 |
| GO_REGUL | GO_REGULATION_OI | 96 | -0.4242 | -1.5431 | 0.0419 | 0.381986 | 0.996 | 8211 | tags=34%, list=1 |
| GO_PHOTO | GO_PHOTORECEPTO | 40 | -0.4993 | -1.5145 | 0.0419 | 0.37563 | 0.999 | 18301 | tags=50%, list=3 |
| GO_NEPHR | GO_NEPHRON_DEVE | 143 | -0.3999 | -1.5037 | 0.042 | 0.374113 | 0.999 | 12175 | tags=39%, list=2 |
| GO_SYNAP | GO_SYNAPSE_ASSEM | 176 | -0.4015 | -1.4806 | 0.0421 | 0.373555 | 0.999 | 11122 | tags=38%, list=1 |
| GO_POSTS | GO_POSTSYNAPSE_A | 31 | -0.5509 | -1.5437 | 0.0422 | 0.381952 | 0.996 | 10943 | tags=52%, list=1 |
| GO_POSITI | GO_POSITIVE_REGUI | 35 | -0.4804 | -1.5012 | 0.0425 | 0.374105 | 0.999 | 13137 | tags=43%, list=2 |
| GO_RESPIR | GO_RESPIRATORY_C | 66 | -0.3995 | -1.4208 | 0.0428 | 0.385527 | 1 | 9188 | tags=35%, list=1 |
| GO_RETINA | GO_RETINA_VASCUI | 18 | -0.6418 | -1.6469 | 0.0428 | 0.31084 | 0.977 | 14838 | tags=67%, list=2 |
| GO_REGUL | GO_REGULATION_OI | 280 | -0.3381 | -1.3726 | 0.0428 | 0.395377 | 1 | 14188 | tags=40%, list=2 |
| GO_NEURC | GO_NEURON_PROJEC | 59 | -0.4266 | -1.5156 | 0.0434 | 0.37939 | 0.998 | 15514 | tags=44%, list=2 |
| GO_ADULT | GO_ADULT_LOCOMC | 74 | -0.4268 | -1.4833 | 0.0436 | 0.373603 | 0.999 | 15699 | tags=54%, list=2 |
| GO_DEVEL | GO_DEVELOPMENTA | 233 | -0.3881 | -1.4916 | 0.0437 | 0.372188 | 0.999 | 8067 | tags=30%, list=1 |
| GO_SYNAP | GO_SYNAPTIC_MEM | 380 | -0.4048 | -1.4715 | 0.0437 | 0.376345 | 0.999 | 13280 | tags=45%, list=2 |
| GO_REGUL | GO_REGULATION_OI | 127 | -0.3856 | -1.4487 | 0.0438 | 0.384876 | 1 | 5887 | tags=24%, list=1 |
| GO_ACTIVI | GO_ACTIVE_ION_TR | 228 | -0.3293 | -1.3869 | 0.0438 | 0.391148 | 1 | 15786 | tags=40%, list=2 |
| GO_TRANS | GO_TRANSFERASE_A | 21 | -0.5319 | -1.5442 | 0.044 | 0.383781 | 0.996 | 7085 | tags=33%, list=1 |
| GO_NEGAT | GO_NEGATIVE_REGU | 130 | -0.388 | -1.482 | 0.0442 | 0.374365 | 0.999 | 17271 | tags=45%, list=3 |
| GO_MIDBR | GO_MIDBRAIN_DEVI | 88 | -0.3999 | -1.4699 | 0.0443 | 0.375855 | 0.999 | 13941 | tags=41%, list=2 |
| GO_POSTS | GO_POSTSYNAPSE_C | 171 | -0.3875 | -1.5094 | 0.0444 | 0.37352 | 0.999 | 11448 | tags=36%, list=2 |
| GO_ESTABI | GO_ESTABLISHMENT | 25 | -0.5343 | -1.5232 | 0.0446 | 0.377578 | 0.997 | 5615 | tags=40%, list=1 |
| GO_NEGAT | GO_NEGATIVE_REGU | 85 | -0.3763 | -1.4487 | 0.0446 | 0.38239 | 1 | 13913 | tags=39%, list=2 |
| GO_POSITI | GO_POSITIVE_REGUI | 33 | -0.4591 | -1.474 | 0.0448 | 0.377376 | 0.999 | 8409 | tags=30%, list=1 |
| GO_REGUL | GO_REGULATION_OI | 142 | -0.4428 | -1.4994 | 0.0454 | 0.369539 | 0.999 | 16853 | tags=54%, list=2 |
| GO_NEGAT | GO_NEGATIVE_REGU | 32 | -0.4805 | -1.5394 | 0.0455 | 0.382493 | 0.996 | 4614 | tags=34%, list=8 |
| GO_INTRAS | GO_INTRASPECIES_I | 52 | -0.4489 | -1.4813 | 0.0455 | 0.375021 | 0.999 | 15769 | tags=48%, list=2 |
| GO_REGUL | GO_REGULATION_OI | 67 | -0.394 | -1.4469 | 0.0456 | 0.381044 | 1 | 8409 | tags=28%, list=1 |
| GO_REGUL | GO_REGULATION_OI | 47 | -0.4459 | -1.4894 | 0.0458 | 0.371073 | 0.999 | 8907 | tags=38%, list=1 |
| GO_SULFUI | GO_SULFUR_AMINO_ | 16 | -0.58 | -1.5318 | 0.0459 | 0.391051 | 0.996 | 2548 | tags=31%, list=4 |
| GO_CYCLIC | GO_CYCLIC_NUCLEC | 38 | -0.4794 | -1.5297 | 0.0459 | 0.387693 | 0.996 | 17430 | tags=58%, list=3 |
| GO_REGUL | GO_REGULATION_OI | 45 | -0.4745 | -1.5333 | 0.046 | 0.390429 | 0.996 | 11645 | tags=44%, list=2 |
| GO_NEPHR | GO_NEPHRON_EPITH | 110 | -0.3854 | -1.45 | 0.046 | 0.388036 | 1 | 13137 | tags=38%, list=2 |
| GO_NEGAT | GO_NEGATIVE_REGU | 15 | -0.5455 | -1.5146 | 0.046 | 0.376688 | 0.999 | 8426 | tags=47%, list=1 |
| GO_REGUL | GO_REGULATION_OI | 17 | -0.5904 | -1.5481 | 0.0463 | 0.381577 | 0.996 | 18511 | tags=76%, list=3 |
| GO_TELEN | GO_TELENCEPHALO | 252 | -0.3832 | -1.4543 | 0.0465 | 0.389536 | 1 | 15888 | tags=43%, list=2 |
| GO_DYNEII | GO_DYNEIN_LIGHT_ | 27 | -0.4692 | -1.5034 | 0.0467 | 0.373516 | 0.999 | 13484 | tags=56%, list=2 |
| GO_REGUL | GO_REGULATION_OI | 303 | -0.3707 | -1.476 | 0.0469 | 0.376874 | 0.999 | 12518 | tags=38%, list=2 |
| GO_REGUL | GO_REGULATION_OI | 18 | -0.5438 | -1.539 | 0.047 | 0.381963 | 0.996 | 10943 | tags=44%, list=1 |
| GO_EPHRII | GO_EPHRIN_RECEPT | 19 | -0.5665 | -1.5759 | 0.0474 | 0.366214 | 0.993 | 9789 | tags=47%, list=1 |
| GO_REGUL | GO_REGULATION_OI | 143 | -0.3681 | -1.4057 | 0.0474 | 0.383133 | 1 | 9573 | tags=35%, list=1 |
| GO_REGUL | GO_REGULATION_OI | 15 | -0.5916 | -1.5136 | 0.0476 | 0.375346 | 0.999 | 5887 | tags=40%, list=1 |

| | | | | | | | | | | |
|---|---|---|---|---|---|---|---|---|---|---|
| GO_TRANS | GO_TRANSMEMBRAI | 18 | -0.5585 | -1.562 | 0.0477 | 0.377881 | 0.994 | 9810 | tags=44%, list=1 | |
| GO_POTAS | GO_POTASSIUM_ION | 240 | -0.3596 | -1.4025 | 0.0479 | 0.38275 | 1 | 15498 | tags=41%, list=2 | |
| GO_NEGAT | GO_NEGATIVE_REGU | 25 | -0.5086 | -1.5529 | 0.0482 | 0.378897 | 0.995 | 17119 | tags=60%, list=2 | |
| GO_INTROI | GO_INTRONIC_TRAN | 16 | -0.5571 | -1.5285 | 0.0482 | 0.380879 | 0.997 | 19979 | tags=69%, list=3 | |
| GO_REGUL | GO_REGULATION_OI | 26 | -0.4895 | -1.5068 | 0.0484 | 0.373599 | 0.999 | 10731 | tags=42%, list=1 | |
| GO_CGMP_ | GO_CGMP_BINDING | 15 | -0.5348 | -1.4863 | 0.0484 | 0.372729 | 0.999 | 19825 | tags=67%, list=3 | |
| GO_NEGAT | GO_NEGATIVE_REGU | 23 | -0.4912 | -1.5102 | 0.0486 | 0.373752 | 0.999 | 22382 | tags=65%, list=3 | |
| GO_COPI_C | GO_COPI_COATED_V | 17 | -0.6707 | -1.651 | 0.0488 | 0.309333 | 0.976 | 10811 | tags=59%, list=1 | |
| GO_ECTOD | GO_ECTODERM_DEV | 22 | -0.4755 | -1.481 | 0.0491 | 0.373749 | 0.999 | 2934 | tags=32%, list=5 | |
| GO_MICRO | GO_MICROTUBULE_I | 77 | -0.4783 | -1.5597 | 0.0492 | 0.377525 | 0.995 | 14495 | tags=51%, list=2 | |
| GO_REGUL | GO_REGULATION_OI | 66 | -0.4635 | -1.5235 | 0.0495 | 0.378113 | 0.997 | 7894 | tags=36%, list=1 | |
| GO_CALCIU | GO_CALCIUM_ION_II | 80 | -0.3877 | -1.4417 | 0.0497 | 0.380407 | 1 | 14054 | tags=41%, list=2 | |
| GO_NBAF_ | GO_NBAF_COMPLEX | 15 | -0.6704 | -1.5841 | 0.0499 | 0.359415 | 0.993 | 12635 | tags=67%, list=2 | |
| GO_NEGAT | GO_NEGATIVE_REGU | 41 | -0.4245 | -1.4594 | 0.0502 | 0.38442 | 1 | 9340 | tags=34%, list=1 | |
| GO_BETA_ | GO_BETA_CATENIN_ | 30 | -0.5507 | -1.5662 | 0.0503 | 0.376976 | 0.994 | 9740 | tags=43%, list=1 | |
| GO_ATP_DI | GO_ATP_DEPENDEN | 18 | -0.5382 | -1.528 | 0.0503 | 0.380718 | 0.997 | 13484 | tags=56%, list=2 | |
| GO_CEREB | GO_CEREBELLAR_PU | 25 | -0.5338 | -1.5496 | 0.0504 | 0.380833 | 0.995 | 11744 | tags=56%, list=2 | |
| GO_REGUL | GO_REGULATION_OI | 20 | -0.4843 | -1.4905 | 0.0507 | 0.370511 | 0.999 | 5704 | tags=25%, list=1 | |
| GO_ACTIN_ | GO_ACTIN_MEDIATE | 120 | -0.4123 | -1.4714 | 0.0507 | 0.375572 | 0.999 | 11874 | tags=37%, list=2 | |
| GO_AXONA | GO_AXONAL_GROW | 28 | -0.4946 | -1.5218 | 0.0508 | 0.375883 | 0.997 | 12361 | tags=57%, list=2 | |
| GO_LUNG_ | GO_LUNG_MORPHOC | 52 | -0.4457 | -1.5286 | 0.0508 | 0.383311 | 0.997 | 8585 | tags=37%, list=1 | |
| GO_NEURA | GO_NEURAL_TUBE_I | 105 | -0.418 | -1.531 | 0.0511 | 0.390217 | 0.996 | 10943 | tags=39%, list=1 | |
| GO_POSITI | GO_POSITIVE_REGUI | 82 | -0.3316 | -1.342 | 0.0511 | 0.408383 | 1 | 7119 | tags=26%, list=1 | |
| GO_CARDI | GO_CARDIAC_MUSC | 54 | -0.4443 | -1.4715 | 0.0515 | 0.377278 | 0.999 | 10232 | tags=39%, list=1 | |
| GO_SULFUI | GO_SULFUR_COMPO | 56 | -0.4306 | -1.4672 | 0.0519 | 0.37813 | 0.999 | 13771 | tags=43%, list=2 | |
| GO_REGUL | GO_REGULATION_OI | 65 | -0.4333 | -1.4787 | 0.0522 | 0.374828 | 0.999 | 4065 | tags=22%, list=7 | |
| GO_KIDNE | GO_KIDNEY_MORPH | 94 | -0.3752 | -1.4277 | 0.0522 | 0.390861 | 1 | 7643 | tags=29%, list=1 | |
| GO_CARBO | GO_CARBOXY_LYAS | 33 | -0.4872 | -1.5158 | 0.0525 | 0.380011 | 0.998 | 10225 | tags=39%, list=1 | |
| GO_POSITI | GO_POSITIVE_REGUI | 15 | -0.5346 | -1.5221 | 0.0526 | 0.37643 | 0.997 | 5842 | tags=40%, list=1 | |
| GO_CHLOR | GO_CHLORIDE_CHAI | 49 | -0.452 | -1.5098 | 0.0527 | 0.373612 | 0.999 | 12825 | tags=49%, list=2 | |
| GO_ESTRO | GO_ESTROGEN_RECI | 39 | -0.4396 | -1.4343 | 0.0532 | 0.384227 | 1 | 8274 | tags=38%, list=1 | |
| GO_LOCOM | GO_LOCOMOTORY_I | 193 | -0.3802 | -1.4657 | 0.0533 | 0.379727 | 0.999 | 11233 | tags=38%, list=1 | |
| GO_CELL_I | GO_CELL_FATE_CON | 34 | -0.4488 | -1.4591 | 0.0535 | 0.384196 | 1 | 10888 | tags=44%, list=1 | |
| GO_REGUL | GO_REGULATION_OI | 43 | -0.4026 | -1.4236 | 0.0536 | 0.387155 | 1 | 10307 | tags=35%, list=1 | |
| GO_CELL_I | GO_CELL_DIFFEREN | 57 | -0.4429 | -1.47 | 0.0536 | 0.376694 | 0.999 | 9318 | tags=37%, list=1 | |
| GO_POSITI | GO_POSITIVE_REGUI | 282 | -0.369 | -1.4487 | 0.0536 | 0.384081 | 1 | 10105 | tags=32%, list=1 | |
| GO_AMINO | GO_AMINO_ACID_CA | 19 | -0.5155 | -1.4846 | 0.0537 | 0.371608 | 0.999 | 15413 | tags=58%, list=2 | |
| GO_ENDOC | GO_ENDOCRINE_PRO | 81 | -0.4007 | -1.4445 | 0.0539 | 0.379522 | 1 | 15424 | tags=40%, list=2 | |
| GO_MODIF | GO_MODIFICATION_ | 24 | -0.5353 | -1.526 | 0.0544 | 0.377365 | 0.997 | 5959 | tags=38%, list=1 | |
| GO_EXPOR | GO_EXPORT_ACROS | 60 | -0.4165 | -1.4745 | 0.0545 | 0.377288 | 0.999 | 14054 | tags=45%, list=2 | |
| GO_NEGAT | GO_NEGATIVE_REGU | 15 | -0.5543 | -1.5226 | 0.0547 | 0.377768 | 0.997 | 7826 | tags=47%, list=1 | |
| GO_CILIUM | GO_CILIUM_OR_FLA | 115 | -0.3776 | -1.4462 | 0.0547 | 0.380933 | 1 | 16638 | tags=43%, list=2 | |
| GO_LIGANI | GO_LIGAND_GATED_ | 19 | -0.6288 | -1.5639 | 0.0549 | 0.375897 | 0.994 | 12500 | tags=68%, list=2 | |
| GO_HMG_E | GO_HMG_BOX_DOM | 16 | -0.5455 | -1.5002 | 0.055 | 0.36976 | 0.999 | 8839 | tags=44%, list=1 | |
| GO_COLLE | GO_COLLECTING_DU | 15 | -0.5533 | -1.519 | 0.055 | 0.376265 | 0.998 | 7640 | tags=40%, list=1 | |
| GO_TRANS | GO_TRANSMEMBRAI | 23 | -0.5035 | -1.4948 | 0.0551 | 0.371423 | 0.999 | 7643 | tags=39%, list=1 | |
| GO_CENTR | GO_CENTRAL_NERV | 192 | -0.4167 | -1.5048 | 0.0551 | 0.372675 | 0.999 | 15429 | tags=48%, list=2 | |
| GO_LUNG_ | GO_LUNG_ALVEOLU | 41 | -0.4767 | -1.5289 | 0.0552 | 0.385472 | 0.997 | 10888 | tags=44%, list=1 | |
| GO_VITAM | GO_VITAMIN_TRANS | 40 | -0.4318 | -1.4536 | 0.0553 | 0.389075 | 1 | 14640 | tags=40%, list=2 | |
| GO_TUBE_I | GO_TUBE_FORMATIC | 149 | -0.384 | -1.4973 | 0.0557 | 0.370929 | 0.999 | 10943 | tags=36%, list=1 | |
| GO_BRANC | GO_BRANCHING_MC | 155 | -0.3721 | -1.4439 | 0.0557 | 0.379033 | 1 | 9208 | tags=33%, list=1 | |
| GO_MEMBI | GO_MEMBRANE_DEI | 23 | -0.5418 | -1.5053 | 0.0561 | 0.373817 | 0.999 | 20619 | tags=70%, list=3 | |
| GO_FEEDIN | GO_FEEDING_BEHA | 105 | -0.3887 | -1.4154 | 0.0562 | 0.384766 | 1 | 16390 | tags=42%, list=2 | |
| GO_INHIBI | GO_INHIBITORY_EX | 15 | -0.6619 | -1.57 | 0.0563 | 0.372443 | 0.994 | 12500 | tags=73%, list=2 | |
| GO_MEMBI | GO_MEMBRANE_DEI | 37 | -0.4955 | -1.4935 | 0.0564 | 0.371092 | 0.999 | 16280 | tags=54%, list=2 | |
| GO_EMBRY | GO_EMBRYONIC_HE | 80 | -0.3897 | -1.4487 | 0.0565 | 0.383224 | 1 | 12794 | tags=39%, list=2 | |
| GO_REGUL | GO_REGULATION_OI | 72 | -0.4098 | -1.4431 | 0.0566 | 0.379025 | 1 | 10140 | tags=33%, list=1 | |
| GO_POSITI | GO_POSITIVE_REGUI | 85 | -0.3733 | -1.4073 | 0.0566 | 0.382764 | 1 | 11153 | tags=32%, list=1 | |

| | | | | | | | | | |
|---|---|---|---|---|---|---|---|---|---|
| GO_REGUL | GO_REGULATION_OF | 16 | -0.627 | -1.5295 | 0.0568 | 0.386844 | 0.996 | 10105 | tags=56%, list=1 |
| GO_VITAM | GO_VITAMIN_TRANS | 29 | -0.4714 | -1.4622 | 0.0568 | 0.383761 | 0.999 | 14640 | tags=45%, list=2 |
| GO_MYELI | GO_MYELIN_ASSEMB | 21 | -0.5278 | -1.5118 | 0.0569 | 0.374785 | 0.999 | 13008 | tags=57%, list=2 |
| GO_POST_E | GO_POST_EMBRYON | 83 | -0.3794 | -1.4336 | 0.0569 | 0.384136 | 1 | 5942 | tags=25%, list=1 |
| GO_SYNTA | GO_SYNTAXIN_1_BIN | 26 | -0.5412 | -1.5204 | 0.0572 | 0.376827 | 0.997 | 16853 | tags=73%, list=2 |
| GO_MANGA | GO_MANGANESE_IO | 15 | -0.5209 | -1.4472 | 0.0574 | 0.383078 | 1 | 15610 | tags=53%, list=2 |
| GO_POSITI | GO_POSITIVE_REGUL | 43 | -0.4604 | -1.5003 | 0.0574 | 0.372726 | 0.999 | 16573 | tags=49%, list=2 |
| GO_REGUL | GO_REGULATION_OF | 46 | -0.409 | -1.4457 | 0.0577 | 0.380976 | 1 | 7861 | tags=30%, list=1 |
| GO_LYSOS | GO_LYSOSOMAL_PR | 15 | -0.5449 | -1.5302 | 0.0577 | 0.387903 | 0.996 | 3754 | tags=33%, list=6 |
| GO_LONG_ | GO_LONG_TERM_SY | 86 | -0.4001 | -1.4536 | 0.058 | 0.388309 | 1 | 8907 | tags=36%, list=1 |
| GO_PATHW | GO_PATHWAY_REST | 65 | -0.4139 | -1.4793 | 0.058 | 0.374575 | 0.999 | 18362 | tags=51%, list=3 |
| GO_VITAM | GO_VITAMIN_B6_BIN | 48 | -0.4125 | -1.4477 | 0.0585 | 0.383698 | 1 | 6471 | tags=27%, list=1 |
| GO_REGUL | GO_REGULATION_OF | 301 | -0.3471 | -1.414 | 0.0585 | 0.384134 | 1 | 12067 | tags=34%, list=2 |
| GO_TRIPAR | GO_TRIPARTITE_REC | 15 | -0.5036 | -1.451 | 0.0591 | 0.389456 | 1 | 12828 | tags=53%, list=2 |
| GO_MESOD | GO_MESODERM_MO | 75 | -0.3805 | -1.4165 | 0.0591 | 0.383992 | 1 | 10951 | tags=37%, list=1 |
| GO_REGUL | GO_REGULATION_OF | 76 | -0.3886 | -1.3934 | 0.0591 | 0.387511 | 1 | 13137 | tags=38%, list=2 |
| GO_GLOME | GO_GLOMERULUS_D | 64 | -0.4765 | -1.5279 | 0.0592 | 0.379726 | 0.997 | 11652 | tags=41%, list=2 |
| GO_POSITI | GO_POSITIVE_REGUL | 154 | -0.3687 | -1.441 | 0.0593 | 0.381197 | 1 | 9996 | tags=32%, list=1 |
| GO_REGUL | GO_REGULATION_OF | 23 | -0.5162 | -1.4951 | 0.0594 | 0.373725 | 0.999 | 7790 | tags=43%, list=1 |
| GO_MODIF | GO_MODIFICATION_ | 18 | -0.5639 | -1.5013 | 0.0595 | 0.374817 | 0.999 | 1449 | tags=28%, list=2 |
| GO_NEGAT | GO_NEGATIVE_REGU | 59 | -0.3704 | -1.3792 | 0.0596 | 0.394962 | 1 | 18325 | tags=49%, list=3 |
| GO_NEURA | GO_NEURAL_RETINA | 72 | -0.4093 | -1.4374 | 0.0598 | 0.384894 | 1 | 15415 | tags=46%, list=2 |
| GO_NUCLE | GO_NUCLEAR_TRAN | 31 | -0.4979 | -1.5015 | 0.0599 | 0.375632 | 0.999 | 12332 | tags=39%, list=2 |
| GO_REGUL | GO_REGULATION_OF | 89 | -0.4084 | -1.4602 | 0.06 | 0.383421 | 0.999 | 14016 | tags=44%, list=2 |
| GO_NEURO | GO_NEUROBLAST_D | 15 | -0.5701 | -1.5275 | 0.0604 | 0.379314 | 0.997 | 10477 | tags=53%, list=1 |
| GO_EXCITA | GO_EXCITATORY_SY | 52 | -0.4835 | -1.515 | 0.0604 | 0.378197 | 0.999 | 10885 | tags=50%, list=1 |
| GO_DENDR | GO_DENDRITIC_SHA | 37 | -0.4607 | -1.455 | 0.0607 | 0.388765 | 1 | 16473 | tags=59%, list=2 |
| GO_NEURO | GO_NEUROTRANSMI | 111 | -0.3992 | -1.3912 | 0.0611 | 0.388255 | 1 | 14144 | tags=41%, list=2 |
| GO_REGUL | GO_REGULATION_OF | 140 | -0.33 | -1.3484 | 0.0613 | 0.407119 | 1 | 11011 | tags=32%, list=1 |
| GO_REGUL | GO_REGULATION_OF | 25 | -0.5888 | -1.4406 | 0.0615 | 0.380502 | 1 | 8836 | tags=56%, list=1 |
| GO_FOREL | GO_FORELIMB_MOR | 40 | -0.4591 | -1.4855 | 0.0615 | 0.3715 | 0.999 | 8338 | tags=43%, list=1 |
| GO_CARDIA | GO_CARDIAC_MUSC | 72 | -0.4156 | -1.4367 | 0.0615 | 0.385474 | 1 | 11874 | tags=38%, list=2 |
| GO_PEPTID | GO_PEPTIDYL_LYSIN | 21 | -0.5537 | -1.5049 | 0.0615 | 0.373543 | 0.999 | 11713 | tags=57%, list=2 |
| GO_ACTIVA | GO_ACTIVATION_OF | 34 | -0.4219 | -1.4151 | 0.0619 | 0.383429 | 1 | 12950 | tags=44%, list=2 |
| GO_GROWT | GO_GROWTH_HORM | 15 | -0.5608 | -1.4861 | 0.0621 | 0.371116 | 0.999 | 15233 | tags=53%, list=2 |
| GO_NEGAT | GO_NEGATIVE_REGU | 25 | -0.5007 | -1.4712 | 0.0621 | 0.374947 | 0.999 | 8290 | tags=48%, list=1 |
| GO_MORPH | GO_MORPHOGENESI | 201 | -0.3661 | -1.4357 | 0.0625 | 0.384466 | 1 | 11180 | tags=35%, list=1 |
| GO_NEPHR | GO_NEPHRON_MORP | 77 | -0.3849 | -1.4097 | 0.0625 | 0.383255 | 1 | 5634 | tags=27%, list=1 |
| GO_RESPO | GO_RESPONSE_TO_P | 21 | -0.5406 | -1.5428 | 0.0626 | 0.379788 | 0.996 | 10708 | tags=43%, list=1 |
| GO_RIBON | GO_RIBONUCLEOTID | 31 | -0.428 | -1.4196 | 0.0628 | 0.38349 | 1 | 3557 | tags=29%, list=6 |
| GO_MULTI | GO_MULTICELLULAR | 204 | -0.3795 | -1.416 | 0.0629 | 0.384353 | 1 | 12152 | tags=36%, list=2 |
| GO_LABYR | GO_LABYRINTHINE_ | 48 | -0.3946 | -1.4019 | 0.0633 | 0.383377 | 1 | 10234 | tags=40%, list=1 |
| GO_MUSCL | GO_MUSCLE_TISSUE | 401 | -0.3446 | -1.3971 | 0.0634 | 0.385164 | 1 | 11823 | tags=34%, list=2 |
| GO_CELL_M | GO_CELL_MIGRATIO | 15 | -0.5459 | -1.488 | 0.0634 | 0.371032 | 0.999 | 14016 | tags=60%, list=2 |
| GO_ATP_DE | GO_ATP_DEPENDENT | 34 | -0.5199 | -1.5146 | 0.0637 | 0.377856 | 0.999 | 14495 | tags=56%, list=2 |
| GO_CONNE | GO_CONNECTIVE_TI | 248 | -0.389 | -1.4753 | 0.0637 | 0.377439 | 0.999 | 12749 | tags=38%, list=2 |
| GO_VOLTA | GO_VOLTAGE_GATE | 43 | -0.4724 | -1.4505 | 0.0637 | 0.387798 | 1 | 19511 | tags=58%, list=3 |
| GO_POSITI | GO_POSITIVE_REGUL | 36 | -0.4401 | -1.4886 | 0.0638 | 0.370722 | 0.999 | 5634 | tags=31%, list=1 |
| GO_REGUL | GO_REGULATION_OF | 193 | -0.3661 | -1.4111 | 0.0638 | 0.385139 | 1 | 9235 | tags=32%, list=1 |
| GO_STRIAT | GO_STRIATED_MUSC | 81 | -0.384 | -1.445 | 0.0641 | 0.379912 | 1 | 9641 | tags=33%, list=1 |
| GO_INHIBI | GO_INHIBITORY_SYN | 17 | -0.5879 | -1.5309 | 0.0641 | 0.389078 | 0.996 | 13278 | tags=59%, list=2 |
| GO_LYSINE | GO_LYSINE_N_METH | 49 | -0.4994 | -1.5151 | 0.0643 | 0.379201 | 0.999 | 12102 | tags=43%, list=2 |
| GO_BRAIN_ | GO_BRAIN_MORPHO | 34 | -0.4679 | -1.469 | 0.0644 | 0.375159 | 0.999 | 10943 | tags=29%, list=1 |
| GO_ATPASE | GO_ATPASE_DEPEND | 24 | -0.4809 | -1.461 | 0.0646 | 0.384542 | 0.999 | 13692 | tags=54%, list=2 |
| GO_MULTI | GO_MULTICELLULAR | 146 | -0.3508 | -1.3982 | 0.0646 | 0.386283 | 1 | 8409 | tags=27%, list=1 |
| GO_ISOPRE | GO_ISOPRENOID_BIO | 27 | -0.5028 | -1.4878 | 0.0648 | 0.37044 | 0.999 | 14556 | tags=52%, list=2 |
| GO_INTRIN | GO_INTRINSIC_COMP | 82 | -0.4441 | -1.4494 | 0.0649 | 0.38763 | 1 | 20073 | tags=62%, list=3 |
| GO_REGUL | GO_REGULATION_OF | 25 | -0.4928 | -1.4604 | 0.066 | 0.384053 | 0.999 | 8024 | tags=48%, list=1 |

| | | | | | | | | | | |
|---|---|---|---|---|---|---|---|---|---|---|
| GO_POSITI | GO_POSITIVE_REGU | 21 | -0.504 | -1.4496 | 0.0661 | 0.387963 | 1 | 15503 | tags=62%, list=2 |
| GO_EPITHE | GO_EPITHELIAL_TUI | 330 | -0.3251 | -1.3616 | 0.0661 | 0.396974 | 1 | 12794 | tags=36%, list=2 |
| GO_NEGAT | GO_NEGATIVE_REGU | 113 | -0.3366 | -1.3492 | 0.0664 | 0.40662 | 1 | 9340 | tags=30%, list=1 |
| GO_DEVEL | GO_DEVELOPMENTA | 233 | -0.3597 | -1.4053 | 0.0668 | 0.38273 | 1 | 13067 | tags=37%, list=2 |
| GO_REGUL | GO_REGULATION_OI | 56 | -0.395 | -1.4106 | 0.0668 | 0.384805 | 1 | 8265 | tags=27%, list=1 |
| GO_EMBRY | GO_EMBRYO_IMPLA | 55 | -0.3774 | -1.4092 | 0.0671 | 0.383574 | 1 | 9127 | tags=33%, list=1 |
| GO_SPERM | GO_SPERM_MOTILIT | 89 | -0.3677 | -1.3896 | 0.0672 | 0.39021 | 1 | 15680 | tags=42%, list=2 |
| GO_CELL_I | GO_CELL_PROLIFER | 18 | -0.5418 | -1.4862 | 0.0674 | 0.371899 | 0.999 | 15096 | tags=50%, list=2 |
| GO_NEGAT | GO_NEGATIVE_REGU | 28 | -0.4693 | -1.4523 | 0.0675 | 0.388328 | 1 | 7280 | tags=36%, list=1 |
| GO_AORTI | GO_AORTIC_VALVE_ | 32 | -0.5374 | -1.5168 | 0.0675 | 0.379001 | 0.998 | 11808 | tags=59%, list=2 |
| GO_NEGAT | GO_NEGATIVE_REGU | 19 | -0.5139 | -1.4606 | 0.0677 | 0.384498 | 0.999 | 9127 | tags=32%, list=1 |
| GO_GASTR | GO_GASTRULATION_ | 26 | -0.4845 | -1.4738 | 0.0679 | 0.376752 | 0.999 | 20508 | tags=73%, list=3 |
| GO_GLUTA | GO_GLUTAMATE_SE | 44 | -0.4431 | -1.4473 | 0.0679 | 0.383545 | 1 | 16390 | tags=55%, list=2 |
| GO_CELL_C | GO_CELL_CELL_CON | 70 | -0.3954 | -1.4138 | 0.0681 | 0.383897 | 1 | 8909 | tags=36%, list=1 |
| GO_HEMOC | GO_HEMOGLOBIN_M | 15 | -0.4966 | -1.4406 | 0.0682 | 0.381258 | 1 | 19350 | tags=60%, list=3 |
| GO_APPEN | GO_APPENDAGE_DE | 176 | -0.3824 | -1.4342 | 0.0683 | 0.383527 | 1 | 8807 | tags=32%, list=1 |
| GO_FIBROI | GO_FIBROBLAST_GR | 25 | -0.5702 | -1.5085 | 0.0683 | 0.373294 | 0.999 | 12526 | tags=56%, list=2 |
| GO_PROTE | GO_PROTEIN_KINAS | 50 | -0.4425 | -1.4934 | 0.0684 | 0.370324 | 0.999 | 8881 | tags=42%, list=1 |
| GO_CEREB | GO_CEREBELLAR_C | 47 | -0.489 | -1.5359 | 0.0685 | 0.385196 | 0.996 | 11744 | tags=47%, list=2 |
| GO_POST_ | GO_POST_ANAL_TAI | 18 | -0.5004 | -1.5003 | 0.0688 | 0.370641 | 0.999 | 6069 | tags=33%, list=1 |
| GO_CELLU | GO_CELLULAR_COM | 108 | -0.4114 | -1.4513 | 0.069 | 0.389672 | 1 | 13249 | tags=41%, list=2 |
| GO_POSTS | GO_POSTSYNAPTIC_ | 40 | -0.4504 | -1.4488 | 0.069 | 0.385481 | 1 | 8836 | tags=38%, list=1 |
| GO_NEGAT | GO_NEGATIVE_REGU | 52 | -0.4068 | -1.4324 | 0.069 | 0.38571 | 1 | 8882 | tags=42%, list=1 |
| GO_CARDI | GO_CARDIAC_MUSC | 73 | -0.403 | -1.3876 | 0.0692 | 0.391757 | 1 | 10232 | tags=34%, list=1 |
| GO_NEGAT | GO_NEGATIVE_REGU | 38 | -0.4211 | -1.4037 | 0.0693 | 0.381763 | 1 | 5704 | tags=29%, list=1 |
| GO_GLUTA | GO_GLUTAMATERGI | 351 | -0.3705 | -1.4054 | 0.0696 | 0.383122 | 1 | 11851 | tags=39%, list=2 |
| GO_NEURC | GO_NEUROTRANSMI | 18 | -0.5081 | -1.4654 | 0.0698 | 0.379589 | 0.999 | 18087 | tags=56%, list=3 |
| GO_REGUL | GO_REGULATION_OI | 55 | -0.3844 | -1.3715 | 0.0703 | 0.395797 | 1 | 10655 | tags=36%, list=1 |
| GO_REGUL | GO_REGULATION_OI | 65 | -0.3723 | -1.3631 | 0.0703 | 0.396308 | 1 | 10655 | tags=35%, list=1 |
| GO_POTAS | GO_POTASSIUM_ION | 29 | -0.4507 | -1.3854 | 0.0707 | 0.392298 | 1 | 15498 | tags=59%, list=2 |
| GO_POSITI | GO_POSITIVE_REGU | 19 | -0.5221 | -1.4562 | 0.0713 | 0.387069 | 1 | 6062 | tags=32%, list=1 |
| GO_REGUL | GO_REGULATION_OI | 445 | -0.3654 | -1.4151 | 0.0714 | 0.383964 | 1 | 9470 | tags=32%, list=1 |
| GO_AUTON | GO_AUTONOMIC_NE | 47 | -0.4302 | -1.4102 | 0.0714 | 0.384342 | 1 | 9908 | tags=38%, list=1 |
| GO_NEURA | GO_NEURAL_TUBE_I | 41 | -0.4696 | -1.5004 | 0.0716 | 0.373541 | 0.999 | 8640 | tags=34%, list=1 |
| GO_POSITI | GO_POSITIVE_REGU | 33 | -0.4362 | -1.4215 | 0.0717 | 0.385421 | 1 | 13311 | tags=45%, list=2 |
| GO_TRANS | GO_TRANSLATION_F | 18 | -0.6051 | -1.4727 | 0.0719 | 0.378255 | 0.999 | 5882 | tags=39%, list=1 |
| GO_ESTABI | GO_ESTABLISHMENT | 15 | -0.5506 | -1.4932 | 0.0721 | 0.369596 | 0.999 | 10943 | tags=47%, list=1 |
| GO_PROTE | GO_PROTEIN_LOCAL | 101 | -0.4049 | -1.4426 | 0.0724 | 0.379412 | 1 | 8159 | tags=32%, list=1 |
| GO_EAR_D | GO_EAR_DEVELOPM | 218 | -0.3625 | -1.3433 | 0.0738 | 0.407105 | 1 | 16463 | tags=45%, list=2 |
| GO_LIPOPR | GO_LIPOPROTEIN_L | 18 | -0.4756 | -1.4469 | 0.074 | 0.38187 | 1 | 7351 | tags=28%, list=1 |
| GO_ORGAN | GO_ORGANIC_ACID_ | 30 | -0.4371 | -1.3846 | 0.0741 | 0.392047 | 1 | 16260 | tags=50%, list=2 |
| GO_REGUL | GO_REGULATION_OI | 39 | -0.4094 | -1.3891 | 0.0744 | 0.390008 | 1 | 6939 | tags=28%, list=1 |
| GO_CELL_I | GO_CELL_PROLIFER | 16 | -0.5151 | -1.4293 | 0.0746 | 0.390698 | 1 | 8815 | tags=38%, list=1 |
| GO_OSTEO | GO_OSTEOBLAST_DI | 220 | -0.3476 | -1.4201 | 0.0746 | 0.383872 | 1 | 12059 | tags=37%, list=2 |
| GO_NEGAT | GO_NEGATIVE_REGU | 62 | -0.3558 | -1.3616 | 0.0746 | 0.396455 | 1 | 7285 | tags=27%, list=1 |
| GO_PHOSP | GO_PHOSPHATASE_I | 96 | -0.3448 | -1.398 | 0.075 | 0.386003 | 1 | 15207 | tags=42%, list=2 |
| GO_NEURC | GO_NEUROTRANSMI | 30 | -0.4309 | -1.3936 | 0.0757 | 0.38771 | 1 | 19243 | tags=50%, list=3 |
| GO_RESPO | GO_RESPONSE_TO_P | 32 | -0.4444 | -1.4274 | 0.0757 | 0.390816 | 1 | 3744 | tags=25%, list=6 |
| GO_ENERG | GO_ENERGY_RESER | 81 | -0.3552 | -1.3572 | 0.0758 | 0.399142 | 1 | 13436 | tags=40%, list=2 |
| GO_NEGAT | GO_NEGATIVE_REGU | 150 | -0.3468 | -1.363 | 0.076 | 0.395489 | 1 | 8823 | tags=33%, list=1 |
| GO_RESPO | GO_RESPONSE_TO_V | 15 | -0.5425 | -1.4489 | 0.0761 | 0.386283 | 1 | 7165 | tags=27%, list=1 |
| GO_CELL_C | GO_CELL_CELL_JUN | 143 | -0.351 | -1.3822 | 0.0762 | 0.391423 | 1 | 12361 | tags=36%, list=2 |
| GO_DIENC | GO_DIENCEPHALON_ | 74 | -0.396 | -1.3753 | 0.0768 | 0.396681 | 1 | 20089 | tags=55%, list=3 |
| GO_MAMM | GO_MAMMARY_GLA | 17 | -0.4696 | -1.4234 | 0.0769 | 0.386786 | 1 | 6439 | tags=35%, list=1 |
| GO_NUCLE | GO_NUCLEAR_MIGR | 23 | -0.5572 | -1.4668 | 0.0771 | 0.378295 | 0.999 | 14756 | tags=57%, list=2 |
| GO_NEURC | GO_NEUROTRANSMI | 51 | -0.4875 | -1.422 | 0.0771 | 0.385076 | 1 | 23857 | tags=76%, list=4 |
| GO_SODIU | GO_SODIUM_ION_HC | 52 | -0.3909 | -1.3606 | 0.0774 | 0.396723 | 1 | 14980 | tags=44%, list=2 |
| GO_PHOSP | GO_PHOSPHATIDIC_ | 49 | -0.3676 | -1.3495 | 0.0775 | 0.406651 | 1 | 10781 | tags=33%, list=1 |

| | | | | | | | | | |
|---|---|---|---|---|---|---|---|---|---|
| GO_NITRIC | GO_NITRIC_OXIDE_M | 27 | -0.4808 | -1.408 | 0.0779 | 0.382723 | 1 | 3576 | tags=26%, list=6 |
| GO_REGUL | GO_REGULATION_OI | 26 | -0.4708 | -1.4224 | 0.078 | 0.385836 | 1 | 12067 | tags=38%, list=2 |
| GO_ACTIOI | GO_ACTION_POTENT | 136 | -0.3718 | -1.3798 | 0.078 | 0.394985 | 1 | 10232 | tags=33%, list=1 |
| GO_MESOD | GO_MESODERM_DEV | 131 | -0.3254 | -1.323 | 0.0781 | 0.409346 | 1 | 10951 | tags=34%, list=1 |
| GO_ION_TF | GO_ION_TRANSMEM | 33 | -0.439 | -1.4066 | 0.0786 | 0.382805 | 1 | 12241 | tags=48%, list=2 |
| GO_POSITI | GO_POSITIVE_REGUI | 27 | -0.5002 | -1.4717 | 0.0787 | 0.377673 | 0.999 | 8426 | tags=33%, list=1 |
| GO_CELL_( | GO_CELL_CELL_JUN | 199 | -0.3354 | -1.3591 | 0.0788 | 0.397882 | 1 | 13008 | tags=36%, list=2 |
| GO_SENSOI | GO_SENSORY_ORGA | 265 | -0.3325 | -1.3371 | 0.0788 | 0.407252 | 1 | 14688 | tags=40%, list=2 |
| GO_EMBRY | GO_EMBRYONIC_OR | 299 | -0.3386 | -1.363 | 0.079 | 0.395989 | 1 | 12111 | tags=33%, list=2 |
| GO_PRESYI | GO_PRESYNAPSE_OI | 53 | -0.4379 | -1.4186 | 0.0792 | 0.383418 | 1 | 10943 | tags=42%, list=1 |
| GO_RESPOI | GO_RESPONSE_TO_N | 52 | -0.4446 | -1.4529 | 0.0793 | 0.38892 | 1 | 7870 | tags=38%, list=1 |
| GO_CELL_I | GO_CELL_DIFFEREN | 57 | -0.4249 | -1.3971 | 0.0797 | 0.384563 | 1 | 16459 | tags=44%, list=2 |
| GO_SMOOT | GO_SMOOTHENED_S | 135 | -0.403 | -1.4471 | 0.0806 | 0.382305 | 1 | 10490 | tags=35%, list=1 |
| GO_CELL_J | GO_CELL_JUNCTION | 422 | -0.3298 | -1.3638 | 0.081 | 0.397257 | 1 | 11122 | tags=33%, list=1 |
| GO_EMBRY | GO_EMBRYONIC_CA | 39 | -0.4535 | -1.4626 | 0.0811 | 0.383896 | 0.999 | 10943 | tags=46%, list=1 |
| GO_TRANS | GO_TRANSMEMBRAI | 353 | -0.3239 | -1.3551 | 0.0812 | 0.400012 | 1 | 12361 | tags=34%, list=2 |
| GO_PERIKA | GO_PERIKARYON | 153 | -0.3915 | -1.4173 | 0.0813 | 0.38473 | 1 | 17048 | tags=52%, list=2 |
| GO_NEGAT | GO_NEGATIVE_REGU | 176 | -0.3419 | -1.3609 | 0.0813 | 0.396661 | 1 | 17271 | tags=44%, list=3 |
| GO_NEURO | GO_NEURON_PROJE( | 37 | -0.4273 | -1.4432 | 0.0815 | 0.379714 | 1 | 11227 | tags=49%, list=1 |
| GO_9PLUS2 | GO_9PLUS2_MOTILE_ | 105 | -0.3483 | -1.3594 | 0.0817 | 0.397908 | 1 | 15284 | tags=38%, list=2 |
| GO_BASEM | GO_BASEMENT_MEM | 102 | -0.4546 | -1.4942 | 0.0818 | 0.370542 | 0.999 | 6867 | tags=38%, list=1 |
| GO_REGUL | GO_REGULATION_OI | 184 | -0.3681 | -1.3926 | 0.0824 | 0.38852 | 1 | 11792 | tags=35%, list=2 |
| GO_T_TUBI | GO_T_TUBULE | 52 | -0.4269 | -1.413 | 0.0828 | 0.384159 | 1 | 8909 | tags=40%, list=1 |
| GO_POSITI | GO_POSITIVE_REGUI | 20 | -0.5216 | -1.4246 | 0.0832 | 0.389044 | 1 | 7379 | tags=35%, list=1 |
| GO_CHEMI | GO_CHEMICAL_SYN | 110 | -0.4163 | -1.412 | 0.0833 | 0.384729 | 1 | 10937 | tags=39%, list=1 |
| GO_TAU_PI | GO_TAU_PROTEIN_B | 45 | -0.4442 | -1.4184 | 0.0835 | 0.383064 | 1 | 6486 | tags=31%, list=1 |
| GO_EPITHE | GO_EPITHELIAL_CEI | 47 | -0.4426 | -1.4082 | 0.0838 | 0.383732 | 1 | 6716 | tags=32%, list=1 |
| GO_POSITI | GO_POSITIVE_REGUI | 17 | -0.5645 | -1.4509 | 0.0841 | 0.388884 | 1 | 7201 | tags=41%, list=1 |
| GO_POSITI | GO_POSITIVE_REGUI | 40 | -0.4291 | -1.4186 | 0.0843 | 0.384105 | 1 | 12950 | tags=38%, list=2 |
| GO_NEURO | GO_NEURON_SPINE | 175 | -0.3541 | -1.3307 | 0.0845 | 0.408975 | 1 | 15095 | tags=47%, list=2 |
| GO_NEURO | GO_NEURONAL_STEI | 23 | -0.4977 | -1.4099 | 0.0845 | 0.383596 | 1 | 8726 | tags=30%, list=1 |
| GO_REGUL | GO_REGULATION_OI | 16 | -0.5696 | -1.4571 | 0.0847 | 0.386921 | 1 | 16720 | tags=69%, list=2 |
| GO_REGUL | GO_REGULATION_OI | 431 | -0.3405 | -1.3482 | 0.0848 | 0.406854 | 1 | 11031 | tags=33%, list=1 |
| GO_EMBRY | GO_EMBRYONIC_SK | 15 | -0.5167 | -1.417 | 0.0849 | 0.384518 | 1 | 8338 | tags=47%, list=1 |
| GO_COCHL | GO_COCHLEA_MORF | 24 | -0.4634 | -1.4286 | 0.0849 | 0.390509 | 1 | 10943 | tags=38%, list=1 |
| GO_NEGAT | GO_NEGATIVE_REGU | 44 | -0.4027 | -1.3961 | 0.0851 | 0.385246 | 1 | 10299 | tags=34%, list=1 |
| GO_REGUL | GO_REGULATION_OI | 165 | -0.2927 | -1.2499 | 0.0853 | 0.429732 | 1 | 11816 | tags=29%, list=2 |
| GO_ATRIAI | GO_ATRIAL_CARDIA | 20 | -0.5158 | -1.4323 | 0.0855 | 0.385091 | 1 | 15888 | tags=65%, list=2 |
| GO_CYCLA | GO_CYCLASE_ACTIV | 18 | -0.5184 | -1.4273 | 0.0855 | 0.390249 | 1 | 23618 | tags=83%, list=4 |
| GO_POSITI | GO_POSITIVE_REGUI | 71 | -0.3619 | -1.3544 | 0.0855 | 0.400308 | 1 | 11011 | tags=31%, list=1 |
| GO_REGUL | GO_REGULATION_OI | 34 | -0.3963 | -1.3241 | 0.0856 | 0.411509 | 1 | 21540 | tags=62%, list=3 |
| GO_CUL3_I | GO_CUL3_RING_UBI( | 36 | -0.4528 | -1.4243 | 0.0861 | 0.388813 | 1 | 3635 | tags=25%, list=6 |
| GO_DIVALI | GO_DIVALENT_INOR | 161 | -0.3459 | -1.3404 | 0.0862 | 0.407291 | 1 | 16273 | tags=44%, list=2 |
| GO_REGUL | GO_REGULATION_OI | 171 | -0.3348 | -1.3202 | 0.0862 | 0.408888 | 1 | 8089 | tags=29%, list=1 |
| GO_POSITI | GO_POSITIVE_REGUI | 25 | -0.4715 | -1.4525 | 0.0864 | 0.388668 | 1 | 4739 | tags=36%, list=8 |
| GO_POSITI | GO_POSITIVE_REGUI | 28 | -0.5246 | -1.4448 | 0.0866 | 0.379715 | 1 | 7648 | tags=43%, list=1 |
| GO_NEURO | GO_NEUROMUSCUL | 50 | -0.4548 | -1.3892 | 0.0867 | 0.390439 | 1 | 16273 | tags=60%, list=2 |
| GO_VOLTA | GO_VOLTAGE_GATE | 138 | -0.4182 | -1.3992 | 0.0869 | 0.38615 | 1 | 19616 | tags=55%, list=3 |
| GO_REGUL | GO_REGULATION_OI | 154 | -0.3512 | -1.3632 | 0.087 | 0.396835 | 1 | 11361 | tags=34%, list=1 |
| GO_EPITHE | GO_EPITHELIAL_TO_ | 17 | -0.5906 | -1.481 | 0.0871 | 0.374561 | 0.999 | 8882 | tags=41%, list=1 |
| GO_9PLUS( | GO_9PLUS0_NON_M( | 132 | -0.3447 | -1.324 | 0.0876 | 0.411203 | 1 | 19648 | tags=47%, list=3 |
| GO_AXON_ | GO_AXON_EXTENSI( | 122 | -0.3914 | -1.3881 | 0.0876 | 0.39126 | 1 | 15894 | tags=47%, list=2 |
| GO_GLUTA | GO_GLUTAMINE_ME | 21 | -0.52 | -1.4247 | 0.0877 | 0.389612 | 1 | 13876 | tags=57%, list=2 |
| GO_CEREB | GO_CEREBELLAR_C( | 23 | -0.5217 | -1.459 | 0.0878 | 0.383576 | 1 | 13615 | tags=52%, list=2 |
| GO_NEURO | GO_NEURON_CELL_( | 17 | -0.5662 | -1.4494 | 0.0888 | 0.386871 | 1 | 9300 | tags=53%, list=1 |
| GO_NEURO | GO_NEUROPEPTIDE_ | 105 | -0.3691 | -1.3416 | 0.0888 | 0.408069 | 1 | 15095 | tags=43%, list=2 |
| GO_MOTOI | GO_MOTOR_ACTIVIT | 132 | -0.3951 | -1.4045 | 0.0888 | 0.383573 | 1 | 14648 | tags=44%, list=2 |
| GO_EMBRY | GO_EMBRYONIC_OR | 447 | -0.3181 | -1.3381 | 0.0888 | 0.407406 | 1 | 10951 | tags=31%, list=1 |

| | | | | | | | | | |
|---|---|---|---|---|---|---|---|---|---|
| GO_BLAST( | GO_BLASTODERM_S | 19 | -0.4315 | -1.3349 | 0.0889 | 0.406741 | 1 | 12828 | tags=47%, list=2 |
| GO_NEGAT | GO_NEGATIVE_REGU | 30 | -0.4768 | -1.3791 | 0.0894 | 0.393949 | 1 | 17412 | tags=43%, list=3 |
| GO_VENTR | GO_VENTRAL_SPINA | 49 | -0.4377 | -1.4008 | 0.0898 | 0.384771 | 1 | 7336 | tags=29%, list=1 |
| GO_SMOOT | GO_SMOOTH_MUSCI | 108 | -0.4214 | -1.4133 | 0.0898 | 0.384144 | 1 | 10853 | tags=42%, list=1 |
| GO_REPLA( | GO_REPLACEMENT_( | 28 | -0.4923 | -1.436 | 0.0898 | 0.38535 | 1 | 10188 | tags=43%, list=1 |
| GO_REGUL | GO_REGULATION_OI | 82 | -0.4182 | -1.4344 | 0.0899 | 0.385454 | 1 | 13137 | tags=41%, list=2 |
| GO_ADREN | GO_ADRENERGIC_RI | 19 | -0.489 | -1.4453 | 0.09 | 0.380258 | 1 | 7522 | tags=37%, list=1 |
| GO_BRANC | GO_BRANCHING_INV | 36 | -0.4977 | -1.5109 | 0.0904 | 0.37464 | 0.999 | 9208 | tags=44%, list=1 |
| GO_BASOL | GO_BASOLATERAL_I | 244 | -0.3065 | -1.2835 | 0.0904 | 0.421631 | 1 | 10205 | tags=30%, list=1 |
| GO_LOCOM | GO_LOCOMOTOR_RI | 15 | -0.4991 | -1.3977 | 0.0906 | 0.385963 | 1 | 10462 | tags=47%, list=1 |
| GO_ZINC_I | GO_ZINC_ION_TRAN | 27 | -0.4941 | -1.4439 | 0.0907 | 0.379839 | 1 | 16073 | tags=56%, list=2 |
| GO_PROST | GO_PROSTATE_GLAI | 49 | -0.3855 | -1.3543 | 0.0907 | 0.399829 | 1 | 7643 | tags=29%, list=1 |
| GO_VITAM | GO_VITAMIN_TRANS | 17 | -0.5002 | -1.3965 | 0.0907 | 0.384993 | 1 | 14640 | tags=47%, list=2 |
| GO_BONE_ | GO_BONE_GROWTH | 31 | -0.4565 | -1.4253 | 0.0911 | 0.390438 | 1 | 14016 | tags=48%, list=2 |
| GO_MUSCL | GO_MUSCLE_CELL_I | 372 | -0.3331 | -1.3447 | 0.0914 | 0.407115 | 1 | 11823 | tags=33%, list=2 |
| GO_SEMI_I | GO_SEMI_LUNAR_V | 37 | -0.5055 | -1.4537 | 0.0917 | 0.389715 | 1 | 11808 | tags=54%, list=2 |
| GO_NEGAT | GO_NEGATIVE_CHEI | 47 | -0.4056 | -1.3842 | 0.0918 | 0.39165 | 1 | 11227 | tags=40%, list=1 |
| GO_BRANC | GO_BRANCH_ELONG | 19 | -0.5119 | -1.4209 | 0.092 | 0.386047 | 1 | 10943 | tags=42%, list=1 |
| GO_SKELE | GO_SKELETAL_SYST | 222 | -0.3525 | -1.3588 | 0.092 | 0.397313 | 1 | 16549 | tags=46%, list=2 |
| GO_CELLU | GO_CELLULAR_AMII | 81 | -0.3598 | -1.3313 | 0.0923 | 0.409498 | 1 | 7085 | tags=23%, list=1 |
| GO_LABYR | GO_LABYRINTHINE_ | 20 | -0.4797 | -1.4238 | 0.0925 | 0.388303 | 1 | 10234 | tags=45%, list=1 |
| GO_NEGAT | GO_NEGATIVE_REGU | 134 | -0.348 | -1.3486 | 0.0927 | 0.407131 | 1 | 16193 | tags=41%, list=2 |
| GO_NEURO | GO_NEURON_PROJE( | 281 | -0.3579 | -1.366 | 0.0927 | 0.398715 | 1 | 11227 | tags=35%, list=1 |
| GO_CEREB | GO_CEREBRAL_COR | 29 | -0.4613 | -1.4113 | 0.093 | 0.385576 | 1 | 18320 | tags=55%, list=3 |
| GO_LEARN | GO_LEARNING | 141 | -0.3815 | -1.3678 | 0.093 | 0.397925 | 1 | 9704 | tags=33%, list=1 |
| GO_POSITI | GO_POSITIVE_REGUI | 84 | -0.3773 | -1.4039 | 0.0931 | 0.382084 | 1 | 12976 | tags=40%, list=2 |
| GO_REGUL | GO_REGULATION_OI | 49 | -0.4188 | -1.399 | 0.0932 | 0.385931 | 1 | 7579 | tags=33%, list=1 |
| GO_INTER( | GO_INTERCALATED_ | 51 | -0.4104 | -1.3742 | 0.0933 | 0.397743 | 1 | 8909 | tags=37%, list=1 |
| GO_NEURO | GO_NEUROTROPHIN | 33 | -0.4169 | -1.3564 | 0.0936 | 0.399713 | 1 | 7373 | tags=36%, list=1 |
| GO_POSITI | GO_POSITIVE_REGUI | 21 | -0.4435 | -1.3787 | 0.0938 | 0.394131 | 1 | 3630 | tags=24%, list=6 |
| GO_CELLU | GO_CELLULAR_RESI | 91 | -0.3606 | -1.3359 | 0.0938 | 0.406921 | 1 | 10151 | tags=27%, list=1 |
| GO_REGUL | GO_REGULATION_OI | 21 | -0.4646 | -1.3925 | 0.094 | 0.388205 | 1 | 13442 | tags=43%, list=2 |
| GO_RECEP | GO_RECEPTOR_SERI | 28 | -0.4354 | -1.3939 | 0.0941 | 0.387855 | 1 | 7643 | tags=32%, list=1 |
| GO_OXIDA | GO_OXIDATIVE_DEM | 19 | -0.5183 | -1.4262 | 0.0945 | 0.390251 | 1 | 943 | tags=21%, list=2 |
| GO_TRIGLY | GO_TRIGLYCERIDE_ | 29 | -0.4119 | -1.3402 | 0.0947 | 0.407112 | 1 | 9980 | tags=34%, list=1 |
| GO_POSITI | GO_POSITIVE_REGUI | 38 | -0.4044 | -1.3549 | 0.0947 | 0.399787 | 1 | 20632 | tags=55%, list=3 |
| GO_NEURO | GO_NEURON_PROJE( | 170 | -0.362 | -1.3459 | 0.0947 | 0.406825 | 1 | 12077 | tags=36%, list=2 |
| GO_CELLU | GO_CELLULAR_SODI | 18 | -0.5066 | -1.4263 | 0.0948 | 0.390676 | 1 | 14054 | tags=61%, list=2 |
| GO_POSITI | GO_POSITIVE_REGUI | 34 | -0.4219 | -1.3915 | 0.0949 | 0.388922 | 1 | 4645 | tags=24%, list=8 |
| GO_REGUL | GO_REGULATION_OI | 17 | -0.4765 | -1.3791 | 0.0949 | 0.394466 | 1 | 13378 | tags=65%, list=2 |
| GO_CHLOR | GO_CHLORIDE_TRAI | 101 | -0.3515 | -1.3186 | 0.0951 | 0.407272 | 1 | 17190 | tags=51%, list=2 |
| GO_OVULA | GO_OVULATION_CY( | 69 | -0.3696 | -1.3264 | 0.0954 | 0.410544 | 1 | 12962 | tags=38%, list=2 |
| GO_POTAS | GO_POTASSIUM_CH | 95 | -0.4296 | -1.3854 | 0.0955 | 0.391773 | 1 | 20230 | tags=57%, list=3 |
| GO_REGIOI | GO_REGIONALIZATI( | 353 | -0.3268 | -1.3214 | 0.0956 | 0.409439 | 1 | 17335 | tags=43%, list=3 |
| GO_REGUL | GO_REGULATION_OI | 109 | -0.3698 | -1.3746 | 0.096 | 0.397402 | 1 | 13067 | tags=38%, list=2 |
| GO_NEGAT | GO_NEGATIVE_REGU | 50 | -0.4209 | -1.3858 | 0.0962 | 0.392316 | 1 | 18948 | tags=50%, list=3 |
| GO_CHONE | GO_CHONDROCYTE_ | 100 | -0.4073 | -1.4145 | 0.0963 | 0.383952 | 1 | 16573 | tags=46%, list=2 |
| GO_SODIUI | GO_SODIUM_CHANN | 26 | -0.479 | -1.3716 | 0.0963 | 0.396291 | 1 | 18109 | tags=58%, list=3 |
| GO_CEREB | GO_CEREBRAL_COR | 40 | -0.4376 | -1.4043 | 0.0965 | 0.382509 | 1 | 18320 | tags=52%, list=3 |
| GO_ACTIN_ | GO_ACTIN_FILAMEN | 150 | -0.3794 | -1.3831 | 0.0966 | 0.390252 | 1 | 11874 | tags=35%, list=2 |
| GO_GABA_ | GO_GABA_ERGIC_SY | 72 | -0.4583 | -1.4081 | 0.097 | 0.383219 | 1 | 18359 | tags=57%, list=3 |
| GO_MUSCL | GO_MUSCLE_CELL_I | 19 | -0.4834 | -1.3578 | 0.0976 | 0.398606 | 1 | 20687 | tags=53%, list=3 |
| GO_IN_UTE | GO_IN_UTERO_EMBI | 378 | -0.3064 | -1.3232 | 0.0977 | 0.410161 | 1 | 10444 | tags=30%, list=1 |
| GO_NEGAT | GO_NEGATIVE_REGU | 22 | -0.4556 | -1.4041 | 0.0981 | 0.382221 | 1 | 9340 | tags=36%, list=1 |
| GO_NOTCH | GO_NOTCH_BINDING | 22 | -0.5085 | -1.4127 | 0.0986 | 0.384093 | 1 | 7175 | tags=41%, list=1 |
| GO_LIPID_I | GO_LIPID_EXPORT_I | 38 | -0.4028 | -1.338 | 0.0986 | 0.40714 | 1 | 9864 | tags=32%, list=1 |
| GO_CARDI | GO_CARDIAC_EPITH | 32 | -0.4913 | -1.4453 | 0.0987 | 0.380984 | 1 | 17220 | tags=53%, list=2 |
| GO_METAN | GO_METANEPHRIC_( | 16 | -0.5762 | -1.427 | 0.0988 | 0.390047 | 1 | 11652 | tags=50%, list=2 |

| | | | | | | | | | |
|---|---|---|---|---|---|---|---|---|---|
| GO_CENTR | GO_CENTRAL_NERV | 82 | -0.3998 | -1.3915 | 0.0988 | 0.388321 | 1 | 18325 | tags=54%, list=3 |
| GO_SEROT | GO_SEROTONIN_REC | 34 | -0.4244 | -1.3481 | 0.099 | 0.406459 | 1 | 20978 | tags=53%, list=3 |
| GO_REGUL | GO_REGULATION_OI | 17 | -0.5152 | -1.4226 | 0.099 | 0.386192 | 1 | 11448 | tags=53%, list=2 |
| GO_CRANI | GO_CRANIAL_SKELE | 68 | -0.398 | -1.3731 | 0.0992 | 0.396225 | 1 | 9854 | tags=37%, list=1 |
| GO_CARBO | GO_CARBOHYDRATI | 112 | -0.3426 | -1.3085 | 0.0992 | 0.410638 | 1 | 11745 | tags=34%, list=2 |
| GO_HIPPO | GO_HIPPOCAMPUS_I | 80 | -0.3853 | -1.3306 | 0.0994 | 0.408645 | 1 | 15403 | tags=43%, list=2 |
| GO_MAIN_ | GO_MAIN_AXON | 68 | -0.4617 | -1.4088 | 0.0996 | 0.383161 | 1 | 14688 | tags=56%, list=2 |
| GO_ARF_G | GO_ARF_GUANYL_N | 18 | -0.4904 | -1.4241 | 0.0996 | 0.388422 | 1 | 12242 | tags=56%, list=2 |
| GO_ERYTH | GO_ERYTHROCYTE_ | 33 | -0.3988 | -1.3293 | 0.0996 | 0.409548 | 1 | 5294 | tags=21%, list=9 |
| GO_DISTAI | GO_DISTAL_AXON | 301 | -0.3735 | -1.374 | 0.1 | 0.397536 | 1 | 16005 | tags=46%, list=2 |
| GO_REGUL | GO_REGULATION_OI | 231 | -0.3546 | -1.3448 | 0.1 | 0.407517 | 1 | 11448 | tags=36%, list=2 |
| GO_ESTABI | GO_ESTABLISHMENT | 17 | -0.5065 | -1.4111 | 0.1004 | 0.384505 | 1 | 10943 | tags=41%, list=1 |
| GO_POTAS | GO_POTASSIUM_ION | 155 | -0.3753 | -1.336 | 0.1006 | 0.407246 | 1 | 15498 | tags=43%, list=2 |
| GO_ENDOC | GO_ENDOCHONDRAI | 54 | -0.428 | -1.387 | 0.1014 | 0.392201 | 1 | 16214 | tags=50%, list=2 |
| GO_REGUL | GO_REGULATION_OI | 25 | -0.4373 | -1.3646 | 0.1014 | 0.397398 | 1 | 5634 | tags=32%, list=1 |
| GO_POSITI | GO_POSITIVE_REGUI | 378 | -0.3242 | -1.3276 | 0.1015 | 0.410244 | 1 | 10105 | tags=30%, list=1 |
| GO_LONG_ | GO_LONG_TERM_SY | 31 | -0.4185 | -1.3277 | 0.1016 | 0.410513 | 1 | 11596 | tags=45%, list=2 |
| GO_CELL_I | GO_CELL_DIFFEREN | 22 | -0.5187 | -1.4203 | 0.1017 | 0.384357 | 1 | 13955 | tags=55%, list=2 |
| GO_RESPO | GO_RESPONSE_TO_C | 17 | -0.4888 | -1.373 | 0.1018 | 0.395912 | 1 | 16182 | tags=59%, list=2 |
| GO_ANTER | GO_ANTERIOR_POST | 218 | -0.3253 | -1.3018 | 0.102 | 0.415803 | 1 | 17335 | tags=43%, list=3 |
| GO_NEGAT | GO_NEGATIVE_REGU | 16 | -0.5269 | -1.4237 | 0.102 | 0.387807 | 1 | 11360 | tags=31%, list=1 |
| GO_EPITHE | GO_EPITHELIAL_CEI | 35 | -0.4121 | -1.3732 | 0.1022 | 0.396537 | 1 | 10272 | tags=43%, list=1 |
| GO_PALLIU | GO_PALLIUM_DEVEI | 166 | -0.3648 | -1.3405 | 0.1023 | 0.407545 | 1 | 15403 | tags=41%, list=2 |
| GO_AXON_ | GO_AXON_INITIAL_S | 17 | -0.5814 | -1.4362 | 0.1025 | 0.38577 | 1 | 19825 | tags=76%, list=3 |
| GO_CAVEO | GO_CAVEOLA | 76 | -0.3944 | -1.4029 | 0.1026 | 0.382699 | 1 | 9810 | tags=39%, list=1 |
| GO_SECON | GO_SECONDARY_AC | 234 | -0.2854 | -1.2341 | 0.1027 | 0.435886 | 1 | 16401 | tags=38%, list=2 |
| GO_POSTSY | GO_POSTSYNAPTIC_ | 65 | -0.4489 | -1.3661 | 0.1027 | 0.399077 | 1 | 19125 | tags=58%, list=3 |
| GO_ANATO | GO_ANATOMICAL_ST | 18 | -0.4985 | -1.4075 | 0.1028 | 0.382923 | 1 | 10523 | tags=50%, list=1 |
| GO_G_PRO | GO_G_PROTEIN_COU | 50 | -0.3965 | -1.3305 | 0.1031 | 0.408222 | 1 | 19125 | tags=50%, list=3 |
| GO_EMBRY | GO_EMBRYONIC_HE | 70 | -0.3638 | -1.3384 | 0.1032 | 0.407934 | 1 | 12794 | tags=36%, list=2 |
| GO_NODE_ | GO_NODE_OF_RANV | 15 | -0.5824 | -1.4358 | 0.1036 | 0.384931 | 1 | 11099 | tags=53%, list=1 |
| GO_NEGAT | GO_NEGATIVE_REGU | 100 | -0.3425 | -1.3134 | 0.1038 | 0.407589 | 1 | 15394 | tags=40%, list=2 |
| GO_NEURO | GO_NEURON_MIGRA | 159 | -0.3836 | -1.3257 | 0.1042 | 0.410947 | 1 | 20062 | tags=58%, list=3 |
| GO_NEGAT | GO_NEGATIVE_REGU | 33 | -0.4237 | -1.3735 | 0.1043 | 0.396568 | 1 | 8907 | tags=33%, list=1 |
| GO_NON_M | GO_NON_MOTILE_CI | 157 | -0.3457 | -1.3197 | 0.1043 | 0.407705 | 1 | 19648 | tags=48%, list=3 |
| GO_FOREB | GO_FOREBRAIN_CEL | 56 | -0.4183 | -1.385 | 0.1047 | 0.391971 | 1 | 18325 | tags=52%, list=3 |
| GO_OLFAC | GO_OLFACTORY_LO | 33 | -0.4658 | -1.3758 | 0.1048 | 0.396179 | 1 | 15403 | tags=58%, list=2 |
| GO_IONOTI | GO_IONOTROPIC_GL | 28 | -0.4424 | -1.3713 | 0.105 | 0.39558 | 1 | 15082 | tags=50%, list=2 |
| GO_PROTEI | GO_PROTEIN_LOCAL | 58 | -0.4692 | -1.4506 | 0.1051 | 0.388569 | 1 | 22739 | tags=72%, list=3 |
| GO_NEURO | GO_NEUROTRANSMI | 21 | -0.4775 | -1.378 | 0.1051 | 0.394266 | 1 | 8287 | tags=43%, list=1 |
| GO_EAR_M | GO_EAR_MORPHOGE | 121 | -0.3618 | -1.318 | 0.1051 | 0.407455 | 1 | 11779 | tags=34%, list=2 |
| GO_INTRA_ | GO_INTRA_GOLGI_V | 33 | -0.4858 | -1.439 | 0.1052 | 0.383067 | 1 | 13882 | tags=52%, list=2 |
| GO_LENS_I | GO_LENS_DEVELOPN | 81 | -0.3436 | -1.3077 | 0.1055 | 0.411281 | 1 | 10943 | tags=32%, list=1 |
| GO_EPITHE | GO_EPITHELIAL_TUI | 29 | -0.4476 | -1.4153 | 0.1055 | 0.384397 | 1 | 6907 | tags=34%, list=1 |
| GO_GASTR | GO_GASTRULATION | 185 | -0.3444 | -1.3511 | 0.1059 | 0.403981 | 1 | 10951 | tags=34%, list=1 |
| GO_CELL_( | GO_CELL_CELL_SIGI | 32 | -0.4577 | -1.3994 | 0.106 | 0.386922 | 1 | 15888 | tags=53%, list=2 |
| GO_LYSINI | GO_LYSINE_ACETYL | 22 | -0.5561 | -1.4344 | 0.1061 | 0.384713 | 1 | 4511 | tags=41%, list=8 |
| GO_STEM_( | GO_STEM_CELL_DIV | 44 | -0.4036 | -1.3694 | 0.1061 | 0.397606 | 1 | 6459 | tags=30%, list=1 |
| GO_REGUL | GO_REGULATION_OI | 112 | -0.4578 | -1.4854 | 0.1066 | 0.370817 | 0.999 | 9389 | tags=39%, list=1 |
| GO_RETINA | GO_RETINAL_BINDII | 17 | -0.505 | -1.3589 | 0.1069 | 0.397721 | 1 | 14556 | tags=53%, list=2 |
| GO_MICRO | GO_MICROTUBULE_I | 92 | -0.4035 | -1.4089 | 0.1069 | 0.383592 | 1 | 22421 | tags=59%, list=3 |
| GO_TONGU | GO_TONGUE_DEVEL | 23 | -0.4791 | -1.4199 | 0.1071 | 0.383584 | 1 | 7885 | tags=39%, list=1 |
| GO_NEGAT | GO_NEGATIVE_REGU | 40 | -0.3733 | -1.3163 | 0.1071 | 0.408885 | 1 | 21386 | tags=63%, list=3 |
| GO_TERPEI | GO_TERPENOID_BIO | 15 | -0.5223 | -1.3942 | 0.1072 | 0.387896 | 1 | 14556 | tags=47%, list=2 |
| GO_NEURO | GO_NEUROTRANSMI | 50 | -0.4622 | -1.3676 | 0.1074 | 0.397746 | 1 | 16720 | tags=56%, list=2 |
| GO_NOTOC | GO_NOTOCHORD_DE | 19 | -0.4662 | -1.3921 | 0.1078 | 0.388227 | 1 | 14539 | tags=53%, list=2 |
| GO_REGUL | GO_REGULATION_OI | 178 | -0.3311 | -1.326 | 0.1081 | 0.410937 | 1 | 11792 | tags=33%, list=2 |
| GO_REFLE | GO_REFLEX | 18 | -0.5263 | -1.3706 | 0.1083 | 0.395863 | 1 | 18132 | tags=61%, list=3 |

| | | | | | | | | | | |
|---|---|---|---|---|---|---|---|---|---|---|
| GO_PROTEI | GO_PROTEIN_METHY | 77 | -0.4357 | -1.4258 | 0.1085 | 0.390179 | 1 | 12102 | tags=36%, list=2 |
| GO_POSITI | GO_POSITIVE_REGUI | 208 | -0.3213 | -1.3386 | 0.1087 | 0.408071 | 1 | 8635 | tags=27%, list=1 |
| GO_DEVEL | GO_DEVELOPMENT_ | 105 | -0.3299 | -1.2942 | 0.1088 | 0.418131 | 1 | 12664 | tags=35%, list=2 |
| GO_SPECTI | GO_SPECTRIN_BINDI | 28 | -0.456 | -1.3762 | 0.1088 | 0.396037 | 1 | 3453 | tags=29%, list=6 |
| GO_EUCHR | GO_EUCHROMATIN | 33 | -0.4793 | -1.4293 | 0.1092 | 0.389924 | 1 | 6062 | tags=30%, list=1 |
| GO_NEGAT | GO_NEGATIVE_REGU | 69 | -0.3641 | -1.3335 | 0.1094 | 0.407309 | 1 | 16473 | tags=46%, list=2 |
| GO_VENTR | GO_VENTRICULAR_C | 36 | -0.4187 | -1.3554 | 0.1095 | 0.400039 | 1 | 10004 | tags=36%, list=1 |
| GO_CARBO | GO_CARBOHYDRATI | 35 | -0.4055 | -1.341 | 0.1098 | 0.407743 | 1 | 19217 | tags=51%, list=3 |
| GO_NEGAT | GO_NEGATIVE_REGU | 30 | -0.4217 | -1.3679 | 0.1098 | 0.39836 | 1 | 7754 | tags=43%, list=1 |
| GO_NEGAT | GO_NEGATIVE_REGU | 28 | -0.4032 | -1.3436 | 0.1098 | 0.40704 | 1 | 11227 | tags=46%, list=1 |
| GO_NEGAT | GO_NEGATIVE_REGU | 124 | -0.3217 | -1.2775 | 0.1098 | 0.42643 | 1 | 15394 | tags=40%, list=2 |
| GO_MOTILI | GO_MOTILE_CILIUM | 177 | -0.3373 | -1.3569 | 0.1105 | 0.39932 | 1 | 19501 | tags=45%, list=3 |
| GO_POSITI | GO_POSITIVE_REGUI | 46 | -0.3927 | -1.3358 | 0.1105 | 0.406507 | 1 | 15888 | tags=50%, list=2 |
| GO_NEURA | GO_NEURAL_CREST_ | 56 | -0.3857 | -1.3691 | 0.1107 | 0.397606 | 1 | 15503 | tags=50%, list=2 |
| GO_NEGAT | GO_NEGATIVE_REGU | 34 | -0.438 | -1.3653 | 0.1109 | 0.3973 | 1 | 7826 | tags=38%, list=1 |
| GO_CELL_F | GO_CELL_FATE_SPEC | 100 | -0.3874 | -1.3646 | 0.1116 | 0.396925 | 1 | 16459 | tags=44%, list=2 |
| GO_MAGNI | GO_MAGNESIUM_IOI | 17 | -0.5161 | -1.3988 | 0.1116 | 0.385657 | 1 | 8736 | tags=47%, list=1 |
| GO_NEGAT | GO_NEGATIVE_REGU | 23 | -0.4867 | -1.3903 | 0.1118 | 0.389483 | 1 | 6062 | tags=30%, list=1 |
| GO_REGUL | GO_REGULATION_OI | 114 | -0.3235 | -1.306 | 0.1122 | 0.414087 | 1 | 10136 | tags=31%, list=1 |
| GO_RESPOI | GO_RESPONSE_TO_F | 16 | -0.4739 | -1.3665 | 0.1122 | 0.398846 | 1 | 4712 | tags=31%, list=8 |
| GO_COLLA | GO_COLLATERAL_SI | 26 | -0.4424 | -1.3634 | 0.1122 | 0.396953 | 1 | 7870 | tags=31%, list=1 |
| GO_POSITI | GO_POSITIVE_REGUI | 85 | -0.3788 | -1.3418 | 0.1124 | 0.408324 | 1 | 9996 | tags=33%, list=1 |
| GO_AXONE | GO_AXONEME_ASSE | 61 | -0.436 | -1.4281 | 0.1126 | 0.390913 | 1 | 15680 | tags=51%, list=2 |
| GO_VOLTA | GO_VOLTAGE_GATE | 17 | -0.5259 | -1.3464 | 0.1127 | 0.408239 | 1 | 16280 | tags=65%, list=2 |
| GO_REGUL | GO_REGULATION_OI | 46 | -0.4239 | -1.3653 | 0.1129 | 0.397741 | 1 | 6421 | tags=30%, list=1 |
| GO_MICRO | GO_MICROTUBULE_ | 21 | -0.5127 | -1.4105 | 0.1129 | 0.384253 | 1 | 7501 | tags=33%, list=1 |
| GO_MOTILI | GO_MOTILE_CILIUM | 22 | -0.5194 | -1.4251 | 0.1132 | 0.390102 | 1 | 11488 | tags=45%, list=2 |
| GO_REGUL | GO_REGULATION_OI | 74 | -0.3384 | -1.2794 | 0.1133 | 0.425151 | 1 | 8426 | tags=27%, list=1 |
| GO_MUSCL | GO_MUSCLE_ORGAN | 393 | -0.3309 | -1.322 | 0.1133 | 0.409397 | 1 | 9340 | tags=30%, list=1 |
| GO_MEMBI | GO_MEMBRANE_REI | 47 | -0.4096 | -1.3415 | 0.1134 | 0.407106 | 1 | 15888 | tags=51%, list=2 |
| GO_MICRO | GO_MICROTUBULE_I | 351 | -0.3577 | -1.3838 | 0.1135 | 0.390677 | 1 | 15680 | tags=40%, list=2 |
| GO_EMBRY | GO_EMBRYONIC_DIC | 34 | -0.468 | -1.396 | 0.1137 | 0.384827 | 1 | 5708 | tags=32%, list=1 |
| GO_SULFUI | GO_SULFUR_COMPO | 244 | -0.3223 | -1.3164 | 0.1139 | 0.409255 | 1 | 14201 | tags=36%, list=2 |
| GO_DNA_M | GO_DNA_METHYLAT | 20 | -0.4876 | -1.3562 | 0.1141 | 0.399463 | 1 | 16537 | tags=40%, list=2 |
| GO_REGUL | GO_REGULATION_OI | 24 | -0.4339 | -1.3462 | 0.1141 | 0.40752 | 1 | 6239 | tags=29%, list=1 |
| GO_REGUL | GO_REGULATION_OI | 24 | -0.4349 | -1.3465 | 0.1143 | 0.40845 | 1 | 3694 | tags=29%, list=6 |
| GO_POSITI | GO_POSITIVE_REGUI | 171 | -0.3576 | -1.3342 | 0.1146 | 0.406992 | 1 | 8907 | tags=31%, list=1 |
| GO_POSITI | GO_POSITIVE_REGUI | 265 | -0.3108 | -1.2868 | 0.115 | 0.419031 | 1 | 11099 | tags=31%, list=1 |
| GO_ZINC_I | GO_ZINC_ION_TRAN | 21 | -0.5051 | -1.4063 | 0.1152 | 0.382617 | 1 | 16073 | tags=62%, list=2 |
| GO_NEGAT | GO_NEGATIVE_REGU | 89 | -0.3561 | -1.32 | 0.1156 | 0.408263 | 1 | 16743 | tags=45%, list=2 |
| GO_VENTR | GO_VENTRAL_SPINA | 17 | -0.5103 | -1.3388 | 0.1161 | 0.408178 | 1 | 16459 | tags=53%, list=2 |
| GO_CELLU | GO_CELLULAR_RESI | 22 | -0.4637 | -1.3721 | 0.1166 | 0.395848 | 1 | 3347 | tags=27%, list=6 |
| GO_REGUL | GO_REGULATION_OI | 56 | -0.3596 | -1.2943 | 0.1168 | 0.418594 | 1 | 5634 | tags=27%, list=1 |
| GO_VOLTA | GO_VOLTAGE_GATE | 87 | -0.4238 | -1.3557 | 0.1169 | 0.399934 | 1 | 20230 | tags=59%, list=3 |
| GO_RNA_P | GO_RNA_POLYMERA | 61 | -0.4219 | -1.3835 | 0.117 | 0.390573 | 1 | 12598 | tags=34%, list=2 |
| GO_REGUL | GO_REGULATION_OI | 26 | -0.4646 | -1.3993 | 0.1171 | 0.386545 | 1 | 7826 | tags=35%, list=1 |
| GO_VOCAL | GO_VOCALIZATION_ | 19 | -0.4931 | -1.3729 | 0.1172 | 0.39543 | 1 | 9854 | tags=47%, list=1 |
| GO_CILIAR | GO_CILIARY_MEMBI | 72 | -0.3656 | -1.3239 | 0.1172 | 0.410403 | 1 | 10493 | tags=32%, list=1 |
| GO_SARCO | GO_SARCOLEMMA | 132 | -0.3887 | -1.384 | 0.1176 | 0.390821 | 1 | 8909 | tags=35%, list=1 |
| GO_MEMOI | GO_MEMORY | 118 | -0.3508 | -1.3125 | 0.1178 | 0.407439 | 1 | 9340 | tags=35%, list=1 |
| GO_STRUC | GO_STRUCTURAL_C | 18 | -0.4967 | -1.3685 | 0.1182 | 0.398333 | 1 | 8159 | tags=44%, list=1 |
| GO_ACETY | GO_ACETYL_COA_M | 38 | -0.4326 | -1.3658 | 0.1183 | 0.398563 | 1 | 3955 | tags=21%, list=7 |
| GO_REGUL | GO_REGULATION_OI | 16 | -0.5337 | -1.3792 | 0.1186 | 0.395434 | 1 | 18541 | tags=63%, list=3 |
| GO_REGUL | GO_REGULATION_OI | 27 | -0.4723 | -1.4043 | 0.1186 | 0.383138 | 1 | 12743 | tags=48%, list=2 |
| GO_CEREB | GO_CEREBELLAR_PU | 15 | -0.5223 | -1.3974 | 0.119 | 0.385234 | 1 | 11744 | tags=53%, list=2 |
| GO_NEURO | GO_NEUROPEPTIDE_ | 26 | -0.431 | -1.3201 | 0.1193 | 0.408442 | 1 | 19821 | tags=69%, list=3 |
| GO_REGUL | GO_REGULATION_OI | 25 | -0.4461 | -1.3655 | 0.1197 | 0.398089 | 1 | 18362 | tags=60%, list=3 |
| GO_SULFUI | GO_SULFUR_AMINO_ | 38 | -0.3965 | -1.3383 | 0.1198 | 0.40751 | 1 | 3723 | tags=18%, list=6 |

| | | | | | | | | | |
|---|---|---|---|---|---|---|---|---|---|
| GO_NEGAT | GO_NEGATIVE_REGU | 16 | -0.4584 | -1.3049 | 0.1202 | 0.414667 | 1 | 5277 | tags=25%, list=9 |
| GO_LUNG_ | GO_LUNG_EPITHELIU | 37 | -0.3864 | -1.3007 | 0.1204 | 0.415164 | 1 | 17156 | tags=46%, list=2 |
| GO_NEURA | GO_NEURAL_PRECU | 149 | -0.358 | -1.3177 | 0.1206 | 0.40755 | 1 | 11374 | tags=34%, list=1 |
| GO_HEART | GO_HEART_PROCESS | 290 | -0.3268 | -1.3008 | 0.1208 | 0.415961 | 1 | 12152 | tags=33%, list=2 |
| GO_CILIAR | GO_CILIARY_PLASM | 113 | -0.4055 | -1.3834 | 0.121 | 0.390275 | 1 | 12242 | tags=38%, list=2 |
| GO_RENAL | GO_RENAL_SYSTEM_ | 28 | -0.5542 | -1.4727 | 0.1214 | 0.377461 | 0.999 | 9318 | tags=57%, list=1 |
| GO_AXOLE | GO_AXOLEMMA | 15 | -0.5679 | -1.3842 | 0.1215 | 0.39107 | 1 | 20089 | tags=73%, list=3 |
| GO_NEGAT | GO_NEGATIVE_REGU | 37 | -0.4338 | -1.3679 | 0.1215 | 0.398916 | 1 | 6847 | tags=30%, list=1 |
| GO_CILIUM | GO_CILIUM_MOVEM | 143 | -0.3485 | -1.329 | 0.1216 | 0.409209 | 1 | 16638 | tags=39%, list=2 |
| GO_NEURO | GO_NEUROTRANSMI | 225 | -0.3646 | -1.354 | 0.122 | 0.400047 | 1 | 10331 | tags=35%, list=1 |
| GO_VOLTA | GO_VOLTAGE_GATE | 41 | -0.4302 | -1.3346 | 0.122 | 0.406778 | 1 | 10331 | tags=37%, list=1 |
| GO_POSITI | GO_POSITIVE_REGU | 20 | -0.542 | -1.4248 | 0.122 | 0.38997 | 1 | 9721 | tags=50%, list=1 |
| GO_REGUL | GO_REGULATION_OI | 79 | -0.4284 | -1.4101 | 0.1221 | 0.383769 | 1 | 5882 | tags=27%, list=1 |
| GO_SPERM | GO_SPERM_PRINCIPA | 23 | -0.4253 | -1.3255 | 0.1223 | 0.410848 | 1 | 26388 | tags=65%, list=4 |
| GO_REGUL | GO_REGULATION_OI | 50 | -0.3678 | -1.2921 | 0.1223 | 0.418008 | 1 | 13631 | tags=46%, list=2 |
| GO_SOLUT | GO_SOLUTE_PROTON | 19 | -0.4419 | -1.3293 | 0.1227 | 0.409041 | 1 | 15610 | tags=53%, list=2 |
| GO_RETINA | GO_RETINA_LAYER_ | 25 | -0.4679 | -1.3975 | 0.1228 | 0.385588 | 1 | 12111 | tags=44%, list=2 |
| GO_TRANS | GO_TRANSMISSION_ | 72 | -0.3969 | -1.3155 | 0.1232 | 0.408438 | 1 | 11099 | tags=38%, list=1 |
| GO_POTAS | GO_POTASSIUM_CHA | 52 | -0.3659 | -1.2896 | 0.1233 | 0.417595 | 1 | 15888 | tags=44%, list=2 |
| GO_MESOD | GO_MESODERMAL_C | 18 | -0.4874 | -1.3425 | 0.1234 | 0.408138 | 1 | 9208 | tags=33%, list=1 |
| GO_EXCRE | GO_EXCRETION | 64 | -0.3651 | -1.2841 | 0.1235 | 0.420876 | 1 | 13846 | tags=39%, list=2 |
| GO_VITAM | GO_VITAMIN_BINDII | 135 | -0.3161 | -1.256 | 0.1235 | 0.42468 | 1 | 12094 | tags=30%, list=2 |
| GO_SEROT | GO_SEROTONIN_REC | 39 | -0.3866 | -1.2673 | 0.1238 | 0.428257 | 1 | 14144 | tags=38%, list=2 |
| GO_CARBO | GO_CARBOHYDRATE | 145 | -0.3221 | -1.2754 | 0.124 | 0.425499 | 1 | 11745 | tags=32%, list=2 |
| GO_NEGAT | GO_NEGATIVE_REGU | 73 | -0.3609 | -1.311 | 0.1241 | 0.408801 | 1 | 12556 | tags=38%, list=2 |
| GO_GOLGI_ | GO_GOLGI_LUMEN | 101 | -0.3667 | -1.2792 | 0.1245 | 0.42461 | 1 | 13137 | tags=35%, list=2 |
| GO_REGUL | GO_REGULATION_OI | 84 | -0.3501 | -1.2991 | 0.1252 | 0.417644 | 1 | 13913 | tags=37%, list=2 |
| GO_NEGAT | GO_NEGATIVE_REGU | 20 | -0.499 | -1.3869 | 0.1253 | 0.390601 | 1 | 6417 | tags=35%, list=1 |
| GO_ANION | GO_ANION_TRANSM | 311 | -0.2968 | -1.2514 | 0.1253 | 0.428957 | 1 | 15498 | tags=37%, list=2 |
| GO_ENDOC | GO_ENDOCRINE_SYS | 125 | -0.329 | -1.2598 | 0.1257 | 0.424781 | 1 | 15534 | tags=43%, list=2 |
| GO_SOMAT | GO_SOMATIC_STEM_ | 75 | -0.3382 | -1.2675 | 0.1257 | 0.429042 | 1 | 7643 | tags=24%, list=1 |
| GO_DYNEII | GO_DYNEIN_COMPL | 53 | -0.4149 | -1.3708 | 0.126 | 0.395927 | 1 | 12302 | tags=45%, list=2 |
| GO_INTRAI | GO_INTRAMOLECUL | 25 | -0.461 | -1.3513 | 0.126 | 0.404111 | 1 | 8903 | tags=32%, list=1 |
| GO_RETINA | GO_RETINA_MORPH | 59 | -0.3693 | -1.3033 | 0.1263 | 0.415457 | 1 | 7640 | tags=27%, list=1 |
| GO_NEGAT | GO_NEGATIVE_REGU | 18 | -0.5786 | -1.4204 | 0.1265 | 0.385594 | 1 | 8399 | tags=33%, list=1 |
| GO_REGUL | GO_REGULATION_OI | 98 | -0.3404 | -1.3085 | 0.1267 | 0.410241 | 1 | 4326 | tags=20%, list=7 |
| GO_REGUL | GO_REGULATION_OI | 29 | -0.4256 | -1.3274 | 0.1268 | 0.410138 | 1 | 18653 | tags=55%, list=3 |
| GO_MULTI_ | GO_MULTI_MULTICE | 222 | -0.2831 | -1.2227 | 0.127 | 0.441841 | 1 | 9408 | tags=28%, list=1 |
| GO_NEGAT | GO_NEGATIVE_REGU | 96 | -0.3356 | -1.2866 | 0.127 | 0.41854 | 1 | 8823 | tags=31%, list=1 |
| GO_NEGAT | GO_NEGATIVE_REGU | 147 | -0.3233 | -1.2855 | 0.1274 | 0.419178 | 1 | 6421 | tags=24%, list=1 |
| GO_REGUL | GO_REGULATION_OI | 49 | -0.3703 | -1.3096 | 0.1274 | 0.409919 | 1 | 6716 | tags=27%, list=1 |
| GO_SKELE | GO_SKELETAL_SYST | 499 | -0.3165 | -1.2968 | 0.1277 | 0.417804 | 1 | 14646 | tags=37%, list=2 |
| GO_NOREP | GO_NOREPINEPHRIN | 25 | -0.4253 | -1.2953 | 0.1277 | 0.418866 | 1 | 5221 | tags=24%, list=9 |
| GO_ERYTH | GO_ERYTHROCYTE_ | 15 | -0.4658 | -1.3357 | 0.1279 | 0.405747 | 1 | 3629 | tags=27%, list=6 |
| GO_SUGAR | GO_SUGAR_TRANSM | 23 | -0.4244 | -1.3141 | 0.128 | 0.407863 | 1 | 20436 | tags=61%, list=3 |
| GO_CELLU | GO_CELLULAR_RESI | 66 | -0.3418 | -1.2658 | 0.1289 | 0.426203 | 1 | 14822 | tags=44%, list=2 |
| GO_PROTEI | GO_PROTEIN_KINAS | 26 | -0.4628 | -1.3869 | 0.129 | 0.391752 | 1 | 5615 | tags=42%, list=1 |
| GO_ORGAN | GO_ORGANIC_HYDR | 262 | -0.2816 | -1.2161 | 0.1292 | 0.442757 | 1 | 9450 | tags=24%, list=1 |
| GO_LATER | GO_LATERAL_PLASM | 64 | -0.3809 | -1.3226 | 0.1293 | 0.409286 | 1 | 9790 | tags=28%, list=1 |
| GO_POSITI | GO_POSITIVE_REGU | 20 | -0.4587 | -1.3204 | 0.1293 | 0.409365 | 1 | 9318 | tags=40%, list=1 |
| GO_POTAS | GO_POTASSIUM_CHA | 119 | -0.392 | -1.3219 | 0.1294 | 0.40912 | 1 | 19152 | tags=52%, list=3 |
| GO_CATEC | GO_CATECHOLAMIN | 55 | -0.4271 | -1.3358 | 0.1296 | 0.406127 | 1 | 11361 | tags=38%, list=1 |
| GO_SITE_O | GO_SITE_OF_POLARI | 185 | -0.3585 | -1.3234 | 0.1298 | 0.410268 | 1 | 12361 | tags=39%, list=2 |
| GO_SYNAP | GO_SYNAPTIC_TRAN | 94 | -0.4163 | -1.3738 | 0.13 | 0.397305 | 1 | 13615 | tags=43%, list=2 |
| GO_PEPTID | GO_PEPTIDYL_ARGII | 15 | -0.5275 | -1.3452 | 0.1306 | 0.407644 | 1 | 13934 | tags=40%, list=2 |
| GO_RHYTH | GO_RHYTHMIC_PRO | 301 | -0.3173 | -1.273 | 0.1306 | 0.42466 | 1 | 12274 | tags=33%, list=2 |
| GO_HORM | GO_HORMONE_BIND | 82 | -0.3482 | -1.2711 | 0.1309 | 0.425033 | 1 | 13742 | tags=37%, list=2 |
| GO_POSITI | GO_POSITIVE_REGU | 97 | -0.3488 | -1.275 | 0.1312 | 0.424565 | 1 | 9235 | tags=34%, list=1 |

| | | | | | | | | | |
|---|---|---|---|---|---|---|---|---|---|
| GO_E_BOX_ | GO_E_BOX_BINDING | 50 | -0.4084 | -1.3243 | 0.1313 | 0.412056 | 1 | 13688 | tags=48%, list=2 |
| GO_CELL_( | GO_CELL_COMMUNI | 56 | -0.3858 | -1.304 | 0.1313 | 0.415033 | 1 | 10232 | tags=32%, list=1 |
| GO_PROTEI | GO_PROTEIN_CATAE | 19 | -0.4312 | -1.3273 | 0.1316 | 0.409765 | 1 | 3754 | tags=26%, list=6 |
| GO_SARCO | GO_SARCOPLASMIC_ | 38 | -0.4122 | -1.3197 | 0.1317 | 0.408176 | 1 | 13692 | tags=39%, list=2 |
| GO_REGUL | GO_REGULATION_OI | 47 | -0.4167 | -1.3638 | 0.1318 | 0.396794 | 1 | 8426 | tags=34%, list=1 |
| GO_ANATO | GO_ANATOMICAL_S | 17 | -0.4564 | -1.3142 | 0.1319 | 0.407972 | 1 | 8489 | tags=47%, list=1 |
| GO_REGUL | GO_REGULATION_OI | 36 | -0.4442 | -1.3377 | 0.132 | 0.407183 | 1 | 10834 | tags=42%, list=1 |
| GO_KERAT | GO_KERATAN_SULF. | 28 | -0.4721 | -1.381 | 0.1322 | 0.393183 | 1 | 8113 | tags=36%, list=1 |
| GO_INTRA( | GO_INTRACELLULAI | 16 | -0.4529 | -1.309 | 0.1331 | 0.410255 | 1 | 5852 | tags=44%, list=1 |
| GO_CATIOI | GO_CATION_CHANN | 313 | -0.3365 | -1.2613 | 0.1335 | 0.424052 | 1 | 15543 | tags=41%, list=2 |
| GO_NOREP | GO_NOREPINEPHRIN | 17 | -0.473 | -1.3184 | 0.1336 | 0.407171 | 1 | 3846 | tags=24%, list=7 |
| GO_NEURO | GO_NEURON_FATE_( | 66 | -0.4249 | -1.322 | 0.1336 | 0.409776 | 1 | 16670 | tags=45%, list=2 |
| GO_DYNEII | GO_DYNEIN_INTERM | 29 | -0.4113 | -1.3189 | 0.1338 | 0.407184 | 1 | 13484 | tags=48%, list=2 |
| GO_POSITI | GO_POSITIVE_REGUI | 36 | -0.4339 | -1.3229 | 0.1343 | 0.409078 | 1 | 18447 | tags=53%, list=3 |
| GO_URETE | GO_URETER_DEVEL( | 18 | -0.4515 | -1.2946 | 0.1344 | 0.418754 | 1 | 10881 | tags=33%, list=1 |
| GO_INNER_ | GO_INNER_EAR_MOI | 101 | -0.3715 | -1.29 | 0.1345 | 0.417852 | 1 | 11779 | tags=34%, list=2 |
| GO_GATED | GO_GATED_CHANNE | 334 | -0.3361 | -1.2732 | 0.1348 | 0.425092 | 1 | 15543 | tags=41%, list=2 |
| GO_REGUL | GO_REGULATION_OI | 177 | -0.344 | -1.2694 | 0.1349 | 0.426891 | 1 | 13991 | tags=40%, list=2 |
| GO_REGUL | GO_REGULATION_OI | 65 | -0.385 | -1.3202 | 0.1352 | 0.409344 | 1 | 17571 | tags=46%, list=3 |
| GO_REGUL | GO_REGULATION_OI | 32 | -0.375 | -1.273 | 0.1354 | 0.425007 | 1 | 5959 | tags=25%, list=1 |
| GO_NEURO | GO_NEUROBLAST_PI | 60 | -0.3952 | -1.315 | 0.1355 | 0.408954 | 1 | 11374 | tags=37%, list=1 |
| GO_ATPASI | GO_ATPASE_COUPLI | 57 | -0.3847 | -1.3134 | 0.1356 | 0.407202 | 1 | 12270 | tags=40%, list=2 |
| GO_BICARI | GO_BICARBONATE_T | 43 | -0.389 | -1.2967 | 0.1358 | 0.417617 | 1 | 6264 | tags=23%, list=1 |
| GO_OSSIFI( | GO_OSSIFICATION | 394 | -0.32 | -1.3105 | 0.1358 | 0.409229 | 1 | 10490 | tags=32%, list=1 |
| GO_POSITI | GO_POSITIVE_REGUI | 60 | -0.3683 | -1.3031 | 0.1358 | 0.415287 | 1 | 16475 | tags=40%, list=2 |
| GO_REGUL | GO_REGULATION_OI | 115 | -0.3433 | -1.2956 | 0.1358 | 0.418722 | 1 | 9901 | tags=35%, list=1 |
| GO_VOLTA | GO_VOLTAGE_GATE | 195 | -0.3566 | -1.2793 | 0.1364 | 0.42498 | 1 | 19152 | tags=48%, list=3 |
| GO_DELAY | GO_DELAYED_RECT | 32 | -0.4528 | -1.2895 | 0.1365 | 0.417401 | 1 | 15057 | tags=47%, list=2 |
| GO_REGUL | GO_REGULATION_OI | 296 | -0.3292 | -1.2876 | 0.1365 | 0.419227 | 1 | 12152 | tags=34%, list=2 |
| GO_POSITI | GO_POSITIVE_REGUI | 18 | -0.53 | -1.3769 | 0.137 | 0.395799 | 1 | 11596 | tags=44%, list=2 |
| GO_DETEC | GO_DETECTION_OF_ | 139 | -0.3183 | -1.2296 | 0.1372 | 0.43765 | 1 | 18221 | tags=42%, list=3 |
| GO_MYOSI | GO_MYOSIN_II_COM | 20 | -0.5045 | -1.3444 | 0.1376 | 0.406587 | 1 | 12596 | tags=45%, list=2 |
| GO_REGUL | GO_REGULATION_OI | 104 | -0.3339 | -1.2881 | 0.138 | 0.419249 | 1 | 11328 | tags=32%, list=1 |
| GO_DOPAM | GO_DOPAMINE_TRA | 48 | -0.4251 | -1.346 | 0.1382 | 0.407357 | 1 | 9243 | tags=38%, list=1 |
| GO_CATIOI | GO_CATION_TRANSI | 18 | -0.4647 | -1.2898 | 0.1383 | 0.417801 | 1 | 13692 | tags=56%, list=2 |
| GO_MAINT | GO_MAINTENANCE_ | 16 | -0.5332 | -1.3782 | 0.1385 | 0.394532 | 1 | 16473 | tags=63%, list=2 |
| GO_CELL_V | GO_CELL_VOLUME_ | 30 | -0.3857 | -1.2907 | 0.1387 | 0.416962 | 1 | 7472 | tags=30%, list=1 |
| GO_POSITI | GO_POSITIVE_REGUI | 42 | -0.4182 | -1.3209 | 0.1389 | 0.409533 | 1 | 13067 | tags=40%, list=2 |
| GO_REGUL | GO_REGULATION_OI | 231 | -0.3509 | -1.3194 | 0.1393 | 0.407207 | 1 | 10331 | tags=34%, list=1 |
| GO_TRANS | GO_TRANSMITTER_( | 60 | -0.421 | -1.3145 | 0.1393 | 0.408397 | 1 | 14794 | tags=50%, list=2 |
| GO_REGUL | GO_REGULATION_OI | 19 | -0.4351 | -1.2918 | 0.1394 | 0.4181 | 1 | 11233 | tags=42%, list=1 |
| GO_REGUL | GO_REGULATION_OI | 76 | -0.4579 | -1.4196 | 0.1398 | 0.382788 | 1 | 10263 | tags=41%, list=1 |
| GO_PROTEI | GO_PROTEIN_LOCAL | 83 | -0.4012 | -1.3399 | 0.1399 | 0.407082 | 1 | 8830 | tags=34%, list=1 |
| GO_NCRNA | GO_NCRNA_EXPORT | 36 | -0.5761 | -1.4493 | 0.14 | 0.386124 | 1 | 9700 | tags=47%, list=1 |
| GO_REGUL | GO_REGULATION_OI | 74 | -0.3357 | -1.2662 | 0.1403 | 0.425878 | 1 | 9635 | tags=27%, list=1 |
| GO_ENSHE | GO_ENSHEATHMENT | 138 | -0.319 | -1.2724 | 0.1404 | 0.424359 | 1 | 8858 | tags=29%, list=1 |
| GO_INORG | GO_INORGANIC_ANI | 143 | -0.3151 | -1.2587 | 0.1406 | 0.42508 | 1 | 7452 | tags=26%, list=1 |
| GO_HEXOS | GO_HEXOSE_TRANSI | 19 | -0.4267 | -1.2771 | 0.1409 | 0.425894 | 1 | 8827 | tags=37%, list=1 |
| GO_PROTO | GO_PROTON_EXPOR | 17 | -0.4646 | -1.302 | 0.1415 | 0.415972 | 1 | 12241 | tags=47%, list=2 |
| GO_SPINAI | GO_SPINAL_CORD_M | 35 | -0.4246 | -1.2879 | 0.1416 | 0.419227 | 1 | 16413 | tags=46%, list=2 |
| GO_REGUL | GO_REGULATION_OI | 129 | -0.3274 | -1.2648 | 0.142 | 0.425945 | 1 | 16894 | tags=44%, list=2 |
| GO_NOSE_I | GO_NOSE_DEVELOPI | 15 | -0.5588 | -1.3362 | 0.1426 | 0.4074 | 1 | 9043 | tags=47%, list=1 |
| GO_CYTOP | GO_CYTOPLASMIC_S | 72 | -0.4322 | -1.3653 | 0.1426 | 0.396715 | 1 | 9901 | tags=36%, list=1 |
| GO_CALCIU | GO_CALCIUM_ION_II | 21 | -0.4245 | -1.2822 | 0.1429 | 0.42299 | 1 | 14054 | tags=48%, list=2 |
| GO_ANTIP( | GO_ANTIPORTER_AC | 82 | -0.3319 | -1.2486 | 0.1429 | 0.429947 | 1 | 15374 | tags=37%, list=2 |
| GO_REGUL | GO_REGULATION_OI | 30 | -0.3871 | -1.2775 | 0.1431 | 0.426143 | 1 | 12398 | tags=37%, list=2 |
| GO_REGUL | GO_REGULATORY_R | 41 | -0.4447 | -1.3139 | 0.1434 | 0.407158 | 1 | 9576 | tags=39%, list=1 |
| GO_RESPOI | GO_RESPONSE_TO_M | 22 | -0.4384 | -1.3269 | 0.1434 | 0.410153 | 1 | 9901 | tags=41%, list=1 |

| | | | | | | | | | |
|---|---|---|---|---|---|---|---|---|---|
| GO_NEGAT | GO_NEGATIVE_REGU | 104 | -0.3184 | -1.2597 | 0.1437 | 0.424473 | 1 | 7285 | tags=24%, list=1 |
| GO_GLUCO | GO_GLUCOSE_IMPOI | 71 | -0.3566 | -1.2645 | 0.1449 | 0.425341 | 1 | 15413 | tags=42%, list=2 |
| GO_MEIOTI | GO_MEIOTIC_CHROM | 22 | -0.5009 | -1.3623 | 0.145 | 0.396095 | 1 | 15158 | tags=50%, list=2 |
| GO_FATTY_ | GO_FATTY_ACYL_CC | 29 | -0.4157 | -1.3307 | 0.1454 | 0.409437 | 1 | 12285 | tags=41%, list=2 |
| GO_CELLU | GO_CELLULAR_COM | 57 | -0.3488 | -1.2577 | 0.146 | 0.424712 | 1 | 11439 | tags=37%, list=2 |
| GO_CALYX | GO_CALYX_OF_HELI | 23 | -0.4921 | -1.3239 | 0.1466 | 0.410822 | 1 | 18541 | tags=61%, list=3 |
| GO_NEURO | GO_NEUROMUSCULA | 104 | -0.3763 | -1.2745 | 0.1468 | 0.424981 | 1 | 16273 | tags=49%, list=2 |
| GO_REGUL | GO_REGULATION_OI | 486 | -0.311 | -1.2722 | 0.147 | 0.423833 | 1 | 12556 | tags=35%, list=2 |
| GO_NEGAT | GO_NEGATIVE_REGU | 25 | -0.4201 | -1.2941 | 0.1473 | 0.41787 | 1 | 18576 | tags=48%, list=3 |
| GO_TRNA_ | GO_TRNA_TRANSPOI | 35 | -0.5759 | -1.4468 | 0.1477 | 0.380407 | 1 | 9700 | tags=46%, list=1 |
| GO_HIGH_I | GO_HIGH_DENSITY_ | 16 | -0.4819 | -1.3312 | 0.1479 | 0.409223 | 1 | 6746 | tags=31%, list=1 |
| GO_EXTRA | GO_EXTRACELLULA | 74 | -0.3847 | -1.2666 | 0.1482 | 0.426942 | 1 | 15363 | tags=50%, list=2 |
| GO_POSITI | GO_POSITIVE_REGUI | 22 | -0.4558 | -1.33 | 0.1486 | 0.408806 | 1 | 4305 | tags=32%, list=7 |
| GO_REGUL | GO_REGULATION_OI | 99 | -0.3396 | -1.2603 | 0.1491 | 0.424611 | 1 | 8907 | tags=30%, list=1 |
| GO_NEGAT | GO_NEGATIVE_REGU | 33 | -0.3793 | -1.2655 | 0.1492 | 0.425547 | 1 | 9340 | tags=30%, list=1 |
| GO_FEMAL | GO_FEMALE_SEX_DI | 119 | -0.3061 | -1.2193 | 0.15 | 0.44108 | 1 | 13841 | tags=38%, list=2 |
| GO_CALMC | GO_CALMODULIN_B | 199 | -0.3233 | -1.2733 | 0.1504 | 0.425512 | 1 | 10444 | tags=32%, list=1 |
| GO_DETEC | GO_DETECTION_OF_ | 43 | -0.3949 | -1.2437 | 0.1505 | 0.432177 | 1 | 10920 | tags=33%, list=1 |
| GO_POTAS | GO_POTASSIUM_ION | 18 | -0.4579 | -1.3027 | 0.1509 | 0.4156 | 1 | 20717 | tags=67%, list=3 |
| GO_POSITI | GO_POSITIVE_REGUI | 16 | -0.486 | -1.3366 | 0.1511 | 0.407666 | 1 | 8997 | tags=50%, list=1 |
| GO_TELEN | GO_TELENCEPHALO | 22 | -0.4477 | -1.3156 | 0.1511 | 0.409221 | 1 | 17193 | tags=55%, list=2 |
| GO_REGUL | GO_REGULATION_OI | 40 | -0.4052 | -1.3119 | 0.1512 | 0.407587 | 1 | 20444 | tags=52%, list=3 |
| GO_NEURO | GO_NEUROMUSCULA | 23 | -0.4571 | -1.2929 | 0.1512 | 0.417935 | 1 | 12506 | tags=52%, list=2 |
| GO_DENDR | GO_DENDRITE_SELF | 16 | -0.4878 | -1.2965 | 0.1513 | 0.417525 | 1 | 12923 | tags=38%, list=2 |
| GO_GAP_JU | GO_GAP_JUNCTION | 31 | -0.3814 | -1.2643 | 0.1517 | 0.42442 | 1 | 6854 | tags=29%, list=1 |
| GO_STRIAT | GO_STRIATED_MUSC | 21 | -0.4754 | -1.3395 | 0.1519 | 0.407485 | 1 | 6864 | tags=29%, list=1 |
| GO_FORMA | GO_FORMATION_OF_ | 124 | -0.3621 | -1.3416 | 0.1522 | 0.407635 | 1 | 10951 | tags=37%, list=1 |
| GO_PROTO | GO_PROTON_TRANS | 137 | -0.3029 | -1.2388 | 0.1523 | 0.433434 | 1 | 10114 | tags=27%, list=1 |
| GO_NEGAT | GO_NEGATIVE_REGU | 183 | -0.3057 | -1.242 | 0.1527 | 0.432371 | 1 | 8823 | tags=30%, list=1 |
| GO_C_ACY | GO_C_ACYLTRANSFI | 20 | -0.4666 | -1.2913 | 0.1529 | 0.418216 | 1 | 12175 | tags=40%, list=2 |
| GO_NEPHR | GO_NEPHRON_TUBU | 18 | -0.4363 | -1.286 | 0.1529 | 0.419016 | 1 | 5439 | tags=28%, list=9 |
| GO_PLASM | GO_PLASMA_MEMBI | 200 | -0.3551 | -1.309 | 0.1532 | 0.410632 | 1 | 17119 | tags=43%, list=2 |
| GO_VOLTA | GO_VOLTAGE_GATE | 23 | -0.4771 | -1.306 | 0.1534 | 0.413662 | 1 | 20619 | tags=65%, list=3 |
| GO_POSITI | GO_POSITIVE_REGUI | 26 | -0.4338 | -1.3156 | 0.1534 | 0.408832 | 1 | 15233 | tags=58%, list=2 |
| GO_BITTER | GO_BITTER_TASTE_I | 20 | -0.5866 | -1.3844 | 0.1535 | 0.391879 | 1 | 13202 | tags=60%, list=2 |
| GO_WALKI | GO_WALKING_BEHA | 29 | -0.4573 | -1.3132 | 0.1538 | 0.407191 | 1 | 18325 | tags=59%, list=3 |
| GO_MUSCL | GO_MUSCLE_FILAMI | 39 | -0.4313 | -1.3025 | 0.154 | 0.415448 | 1 | 11765 | tags=38%, list=2 |
| GO_REGUL | GO_REGULATION_OI | 15 | -0.5176 | -1.3445 | 0.1543 | 0.406987 | 1 | 4573 | tags=27%, list=8 |
| GO_REGUL | GO_REGULATION_OI | 117 | -0.3868 | -1.3373 | 0.1543 | 0.407331 | 1 | 10263 | tags=35%, list=1 |
| GO_SYNAP | GO_SYNAPTIC_CLEF | 16 | -0.4914 | -1.3437 | 0.1545 | 0.407327 | 1 | 7648 | tags=38%, list=1 |
| GO_REGUL | GO_REGULATION_OI | 58 | -0.3699 | -1.267 | 0.1549 | 0.427017 | 1 | 15413 | tags=43%, list=2 |
| GO_TROPH | GO_TROPHOBLAST_( | 16 | -0.4336 | -1.2848 | 0.155 | 0.419914 | 1 | 9127 | tags=38%, list=1 |
| GO_CELLU | GO_CELLULAR_RESI | 140 | -0.2998 | -1.2232 | 0.1551 | 0.442876 | 1 | 11903 | tags=31%, list=2 |
| GO_AXONE | GO_AXONEMAL_DYI | 29 | -0.4727 | -1.3518 | 0.1552 | 0.403739 | 1 | 11864 | tags=48%, list=2 |
| GO_ESTABI | GO_ESTABLISHMENT | 21 | -0.405 | -1.2728 | 0.1552 | 0.424137 | 1 | 13615 | tags=43%, list=2 |
| GO_REGUL | GO_REGULATION_OI | 78 | -0.3961 | -1.2736 | 0.1553 | 0.425743 | 1 | 15315 | tags=49%, list=2 |
| GO_MODIF | GO_MODIFIED_AMIN | 27 | -0.4063 | -1.2736 | 0.1555 | 0.425308 | 1 | 7781 | tags=30%, list=1 |
| GO_NEGAT | GO_NEGATIVE_REGU | 232 | -0.3032 | -1.2328 | 0.1556 | 0.435113 | 1 | 7482 | tags=22%, list=1 |
| GO_POSITI | GO_POSITIVE_REGUI | 111 | -0.3437 | -1.2927 | 0.1558 | 0.417754 | 1 | 11210 | tags=32%, list=1 |
| GO_VOLTA | GO_VOLTAGE_GATE | 17 | -0.4922 | -1.3314 | 0.1561 | 0.40989 | 1 | 2399 | tags=18%, list=4 |
| GO_ANION_ | GO_ANION_TRANSM | 353 | -0.2761 | -1.2024 | 0.1561 | 0.449371 | 1 | 15498 | tags=35%, list=2 |
| GO_EXTRA | GO_EXTRACELLULA | 22 | -0.533 | -1.3736 | 0.1562 | 0.397108 | 1 | 13904 | tags=55%, list=2 |
| GO_HEPAR | GO_HEPARIN_BINDII | 158 | -0.3715 | -1.3147 | 0.1563 | 0.408535 | 1 | 8308 | tags=30%, list=1 |
| GO_GLYCO | GO_GLYCOGEN_BIOS | 45 | -0.3567 | -1.2297 | 0.1563 | 0.437911 | 1 | 20825 | tags=58%, list=3 |
| GO_REGUL | GO_REGULATION_OI | 100 | -0.3578 | -1.2728 | 0.1564 | 0.424447 | 1 | 10943 | tags=37%, list=1 |
| GO_NEURO | GO_NEURONAL_ACT | 34 | -0.4227 | -1.2642 | 0.1564 | 0.424218 | 1 | 11031 | tags=41%, list=1 |
| GO_MALE_ | GO_MALE_GENITALI | 23 | -0.4358 | -1.2912 | 0.1566 | 0.417391 | 1 | 11652 | tags=39%, list=2 |
| GO_REPRO | GO_REPRODUCTIVE_ | 443 | -0.2735 | -1.2123 | 0.1567 | 0.445521 | 1 | 11903 | tags=31%, list=2 |

| | | | | | | | | | |
|---|---|---|---|---|---|---|---|---|---|
| GO_ESTABI | GO_ESTABLISHMENT | 137 | -0.3467 | -1.2969 | 0.1568 | 0.418229 | 1 | 12361 | tags=35%, list=2 |
| GO_REGUL | GO_REGULATION_OI | 52 | -0.3715 | -1.2407 | 0.1569 | 0.43257 | 1 | 15864 | tags=40%, list=2 |
| GO_STRIAT | GO_STRIATED_MUSC | 283 | -0.3096 | -1.245 | 0.157 | 0.433055 | 1 | 11823 | tags=33%, list=2 |
| GO_STRIAT | GO_STRIATED_MUSC | 171 | -0.3276 | -1.2663 | 0.1575 | 0.426965 | 1 | 11874 | tags=33%, list=2 |
| GO_REGUL | GO_REGULATION_OI | 80 | -0.3846 | -1.3336 | 0.1576 | 0.407632 | 1 | 7793 | tags=30%, list=1 |
| GO_INHIBI | GO_INHIBITORY_POS | 16 | -0.5428 | -1.3211 | 0.1578 | 0.409512 | 1 | 18875 | tags=63%, list=3 |
| GO_GLOME | GO_GLOMERULAR_E | 20 | -0.4524 | -1.2857 | 0.1579 | 0.419257 | 1 | 26429 | tags=65%, list=4 |
| GO_DETEC | GO_DETECTION_OF_ | 62 | -0.3298 | -1.2202 | 0.1582 | 0.44221 | 1 | 18221 | tags=42%, list=3 |
| GO_REGUL | GO_REGULATION_OI | 23 | -0.3975 | -1.2627 | 0.1582 | 0.424031 | 1 | 20059 | tags=61%, list=3 |
| GO_REGUL | GO_REGULATION_OI | 34 | -0.4592 | -1.3148 | 0.1587 | 0.408773 | 1 | 9243 | tags=41%, list=1 |
| GO_TUBUL | GO_TUBULIN_BINDII | 348 | -0.3539 | -1.2981 | 0.1587 | 0.418275 | 1 | 14612 | tags=39%, list=2 |
| GO_ACTIVA | GO_ACTIVATION_OF | 18 | -0.4756 | -1.3406 | 0.1588 | 0.407904 | 1 | 17430 | tags=56%, list=3 |
| GO_SARCO | GO_SARCOPLASM | 77 | -0.3755 | -1.3143 | 0.1588 | 0.408356 | 1 | 14545 | tags=43%, list=2 |
| GO_CARDIA | GO_CARDIAC_MUSC | 135 | -0.3326 | -1.2466 | 0.1592 | 0.432427 | 1 | 10232 | tags=31%, list=1 |
| GO_REGUL | GO_REGULATION_OI | 47 | -0.3671 | -1.2494 | 0.1594 | 0.429404 | 1 | 12168 | tags=43%, list=2 |
| GO_SYNAP | GO_SYNAPTIC_TRAN | 30 | -0.3908 | -1.2574 | 0.1596 | 0.423383 | 1 | 8287 | tags=30%, list=1 |
| GO_REGUL | GO_REGULATION_OI | 167 | -0.3185 | -1.2599 | 0.1596 | 0.424998 | 1 | 9577 | tags=27%, list=1 |
| GO_CELL_I | GO_CELL_FATE_DET | 42 | -0.3963 | -1.2608 | 0.1602 | 0.424227 | 1 | 7885 | tags=33%, list=1 |
| GO_MUSCL | GO_MUSCLE_HYPER | 106 | -0.3394 | -1.2566 | 0.1603 | 0.424316 | 1 | 12950 | tags=34%, list=2 |
| GO_PROTEI | GO_PROTEIN_MEMB | 20 | -0.4111 | -1.2513 | 0.1608 | 0.428799 | 1 | 13008 | tags=45%, list=2 |
| GO_TRANS | GO_TRANSFORMING | 23 | -0.5143 | -1.3463 | 0.1608 | 0.407734 | 1 | 17271 | tags=52%, list=3 |
| GO_NEGAT | GO_NEGATIVE_REGU | 101 | -0.3031 | -1.2227 | 0.161 | 0.442146 | 1 | 11087 | tags=28%, list=1 |
| GO_MICRO | GO_MICROTUBULE_I | 20 | -0.5337 | -1.3602 | 0.1612 | 0.396851 | 1 | 6486 | tags=30%, list=1 |
| GO_POSITI | GO_POSITIVE_REGUI | 50 | -0.4206 | -1.333 | 0.1615 | 0.407153 | 1 | 12664 | tags=44%, list=2 |
| GO_TRIGLY | GO_TRIGLYCERIDE_( | 38 | -0.3848 | -1.2445 | 0.1619 | 0.432636 | 1 | 5875 | tags=24%, list=1 |
| GO_DNA_P | GO_DNA_PACKAGIN | 23 | -0.5168 | -1.3769 | 0.1619 | 0.395179 | 1 | 13109 | tags=43%, list=2 |
| GO_MUSCL | GO_MUSCLE_CONTR | 353 | -0.353 | -1.3131 | 0.1619 | 0.406873 | 1 | 11874 | tags=37%, list=2 |
| GO_LIMBIC | GO_LIMBIC_SYSTEM | 108 | -0.3442 | -1.2449 | 0.1622 | 0.432781 | 1 | 15403 | tags=37%, list=2 |
| GO_PHENO | GO_PHENOTYPIC_SV | 15 | -0.5291 | -1.3044 | 0.1624 | 0.414697 | 1 | 13862 | tags=47%, list=2 |
| GO_REGUL | GO_REGULATION_OI | 72 | -0.3714 | -1.3013 | 0.1625 | 0.415805 | 1 | 9654 | tags=32%, list=1 |
| GO_NEURO | GO_NEUROTRANSMI | 31 | -0.4028 | -1.2613 | 0.1626 | 0.423732 | 1 | 18325 | tags=48%, list=3 |
| GO_ACIDIC | GO_ACIDIC_AMINO_ | 70 | -0.3512 | -1.2463 | 0.1626 | 0.432242 | 1 | 16390 | tags=46%, list=2 |
| GO_STERO | GO_STEROID_HORM | 78 | -0.3457 | -1.2662 | 0.1627 | 0.426288 | 1 | 8314 | tags=33%, list=1 |
| GO_COPII_ | GO_COPII_COATED_ | 15 | -0.5549 | -1.3615 | 0.1632 | 0.396052 | 1 | 15233 | tags=60%, list=2 |
| GO_CELL_I | GO_CELL_PROJECTIC | 335 | -0.2865 | -1.2123 | 0.1632 | 0.445856 | 1 | 13067 | tags=34%, list=2 |
| GO_GLYCO | GO_GLYCOPROTEIN_ | 19 | -0.5129 | -1.3655 | 0.1633 | 0.398655 | 1 | 5834 | tags=42%, list=1 |
| GO_ORGAN | GO_ORGANIC_HYDR | 256 | -0.2854 | -1.2106 | 0.1633 | 0.446338 | 1 | 7361 | tags=20%, list=1 |
| GO_VESICI | GO_VESICLE_MEDIA | 217 | -0.3747 | -1.3241 | 0.1636 | 0.411876 | 1 | 9535 | tags=34%, list=1 |
| GO_CORTIC | GO_CORTICAL_ACTI | 79 | -0.3342 | -1.2408 | 0.1638 | 0.432761 | 1 | 11765 | tags=32%, list=2 |
| GO_ASSOC | GO_ASSOCIATIVE_LI | 75 | -0.365 | -1.2671 | 0.1638 | 0.427218 | 1 | 11028 | tags=33%, list=1 |
| GO_POSITI | GO_POSITIVE_REGUI | 21 | -0.4202 | -1.2618 | 0.164 | 0.42433 | 1 | 5439 | tags=29%, list=9 |
| GO_RESPO | GO_RESPONSE_TO_V | 19 | -0.4431 | -1.2576 | 0.1641 | 0.424106 | 1 | 14804 | tags=58%, list=2 |
| GO_NEGAT | GO_NEGATIVE_REGU | 49 | -0.3452 | -1.2501 | 0.1641 | 0.429798 | 1 | 6476 | tags=24%, list=1 |
| GO_AMINE | GO_AMINE_TRANSP | 96 | -0.3614 | -1.2485 | 0.1643 | 0.42983 | 1 | 19324 | tags=49%, list=3 |
| GO_EYE_M | GO_EYE_MORPHOGE | 154 | -0.3105 | -1.2367 | 0.1644 | 0.43346 | 1 | 14688 | tags=41%, list=2 |
| GO_PASSIV | GO_PASSIVE_TRANS | 457 | -0.2992 | -1.204 | 0.1646 | 0.448836 | 1 | 15397 | tags=38%, list=2 |
| GO_BASIC_ | GO_BASIC_AMINO_A | 17 | -0.4617 | -1.2946 | 0.165 | 0.419189 | 1 | 6516 | tags=24%, list=1 |
| GO_CYTOP | GO_CYTOPLASMIC_I | 243 | -0.3412 | -1.2866 | 0.165 | 0.418822 | 1 | 9173 | tags=28%, list=1 |
| GO_NEGAT | GO_NEGATIVE_REGU | 154 | -0.3175 | -1.2621 | 0.1651 | 0.424276 | 1 | 12756 | tags=34%, list=2 |
| GO_REGUL | GO_REGULATION_OI | 217 | -0.303 | -1.249 | 0.1651 | 0.429764 | 1 | 6486 | tags=24%, list=1 |
| GO_DOPAM | GO_DOPAMINE_RECI | 17 | -0.4557 | -1.2947 | 0.1657 | 0.41964 | 1 | 12151 | tags=41%, list=2 |
| GO_POSITI | GO_POSITIVE_REGUI | 171 | -0.3191 | -1.2631 | 0.1657 | 0.424039 | 1 | 10045 | tags=33%, list=1 |
| GO_PRESYI | GO_PRESYNAPTIC_E | 62 | -0.3627 | -1.2647 | 0.1657 | 0.425694 | 1 | 7560 | tags=32%, list=1 |
| GO_COGNI | GO_COGNITION | 293 | -0.3091 | -1.2298 | 0.1657 | 0.438066 | 1 | 9854 | tags=32%, list=1 |
| GO_REGUL | GO_REGULATION_OI | 88 | -0.3322 | -1.2418 | 0.166 | 0.432023 | 1 | 11099 | tags=33%, list=1 |
| GO_PRESYI | GO_PRESYNAPTIC_M | 154 | -0.3787 | -1.297 | 0.1667 | 0.418383 | 1 | 12388 | tags=42%, list=2 |
| GO_REGUL | GO_REGULATION_OI | 38 | -0.4087 | -1.2751 | 0.1667 | 0.424828 | 1 | 20619 | tags=58%, list=3 |
| GO_VASCU | GO_VASCULAR_PRO | 189 | -0.3299 | -1.2399 | 0.1667 | 0.432476 | 1 | 11664 | tags=34%, list=2 |

| | | | | | | | | | |
|---|---|---|---|---|---|---|---|---|---|
| GO_HORMO | GO_HORMONE_TRAN | 315 | -0.2813 | -1.1972 | 0.1667 | 0.451869 | 1 | 12664 | tags=32%, list=2 |
| GO_GLUTA | GO_GLUTAMINE_FAI | 71 | -0.3351 | -1.2255 | 0.167 | 0.439715 | 1 | 14421 | tags=38%, list=2 |
| GO_CELLU | GO_CELLULAR_RESI | 81 | -0.3448 | -1.2378 | 0.167 | 0.433082 | 1 | 10943 | tags=30%, list=1 |
| GO_SYNAP | GO_SYNAPTIC_MEM | 28 | -0.4526 | -1.316 | 0.1673 | 0.409002 | 1 | 11122 | tags=43%, list=1 |
| GO_ENDOT | GO_ENDOTHELIUM_ | 132 | -0.3764 | -1.3366 | 0.1674 | 0.407193 | 1 | 9208 | tags=35%, list=1 |
| GO_OOCYT | GO_OOCYTE_DIFFER | 48 | -0.3503 | -1.2268 | 0.1676 | 0.439307 | 1 | 8476 | tags=27%, list=1 |
| GO_RETRO | GO_RETROGRADE_A | 20 | -0.5001 | -1.3097 | 0.1676 | 0.410238 | 1 | 4046 | tags=30%, list=7 |
| GO_INTRA | GO_INTRACELLULAI | 54 | -0.3878 | -1.2941 | 0.1677 | 0.417503 | 1 | 9654 | tags=31%, list=1 |
| GO_SMOOT | GO_SMOOTH_ENDOI | 34 | -0.3739 | -1.2382 | 0.1677 | 0.433254 | 1 | 8265 | tags=26%, list=1 |
| GO_TYPE_I | GO_TYPE_B_PANCRE | 20 | -0.4625 | -1.3007 | 0.1677 | 0.415545 | 1 | 10943 | tags=45%, list=1 |
| GO_CELL_I | GO_CELL_DIFFEREN | 26 | -0.4514 | -1.2921 | 0.1677 | 0.418399 | 1 | 9318 | tags=46%, list=1 |
| GO_NEGAT | GO_NEGATIVE_REGU | 18 | -0.4808 | -1.3452 | 0.168 | 0.40725 | 1 | 2766 | tags=28%, list=5 |
| GO_MUSCL | GO_MUSCLE_CELL_I | 177 | -0.3338 | -1.2542 | 0.168 | 0.425865 | 1 | 11808 | tags=32%, list=2 |
| GO_MYOSI | GO_MYOSIN_FILAMI | 22 | -0.4778 | -1.3016 | 0.1683 | 0.415868 | 1 | 12387 | tags=41%, list=2 |
| GO_MICRO | GO_MICROTUBULE_I | 185 | -0.353 | -1.2892 | 0.1686 | 0.417579 | 1 | 15533 | tags=42%, list=2 |
| GO_REGUL | GO_REGULATION_OI | 41 | -0.4049 | -1.2764 | 0.1687 | 0.425061 | 1 | 14070 | tags=44%, list=2 |
| GO_MICRO | GO_MICROTUBULE_I | 74 | -0.3816 | -1.3038 | 0.1696 | 0.414973 | 1 | 6486 | tags=27%, list=1 |
| GO_AMINO | GO_AMINOGLYCAN_ | 66 | -0.3715 | -1.2799 | 0.1696 | 0.425118 | 1 | 13771 | tags=45%, list=2 |
| GO_ACTIVI | GO_ACTIVIN_RECEP | 44 | -0.3669 | -1.2579 | 0.1697 | 0.425326 | 1 | 14016 | tags=36%, list=2 |
| GO_NEGAT | GO_NEGATIVE_REGU | 15 | -0.4759 | -1.2913 | 0.1702 | 0.417758 | 1 | 3653 | tags=27%, list=6 |
| GO_CATIO | GO_CATION_CHLORI | 17 | -0.4364 | -1.2419 | 0.1703 | 0.432223 | 1 | 17190 | tags=59%, list=2 |
| GO_AMPA_ | GO_AMPA_GLUTAM | 26 | -0.4982 | -1.2978 | 0.1704 | 0.417966 | 1 | 13473 | tags=50%, list=2 |
| GO_NEGAT | GO_NEGATIVE_REGU | 16 | -0.543 | -1.3193 | 0.1711 | 0.407009 | 1 | 10347 | tags=38%, list=1 |
| GO_INSULI | GO_INSULIN_RECEP | 23 | -0.4308 | -1.2699 | 0.1711 | 0.426761 | 1 | 12950 | tags=43%, list=2 |
| GO_PRESY | GO_PRESYNAPTIC_A | 74 | -0.4289 | -1.3285 | 0.1712 | 0.409534 | 1 | 11596 | tags=45%, list=2 |
| GO_REGUL | GO_REGULATION_OI | 44 | -0.3721 | -1.2624 | 0.1713 | 0.424036 | 1 | 11227 | tags=39%, list=1 |
| GO_NEGAT | GO_NEGATIVE_REGU | 27 | -0.4425 | -1.291 | 0.1714 | 0.416964 | 1 | 17571 | tags=52%, list=3 |
| GO_FOCAL | GO_FOCAL_ADHESIC | 83 | -0.3844 | -1.3246 | 0.1715 | 0.412089 | 1 | 8218 | tags=33%, list=1 |
| GO_ALDEH | GO_ALDEHYDE_BIOS | 15 | -0.4395 | -1.2794 | 0.1715 | 0.425543 | 1 | 14980 | tags=47%, list=2 |
| GO_AMINO | GO_AMINO_ACID_SC | 15 | -0.4723 | -1.2985 | 0.1715 | 0.417962 | 1 | 15413 | tags=53%, list=2 |
| GO_EUKAR | GO_EUKARYOTIC_TI | 17 | -0.556 | -1.3641 | 0.1719 | 0.397311 | 1 | 4823 | tags=29%, list=8 |
| GO_EXTRA | GO_EXTRACELLULA | 166 | -0.4452 | -1.3675 | 0.1721 | 0.397269 | 1 | 14616 | tags=49%, list=2 |
| GO_INSULI | GO_INSULIN_SECRE | 62 | -0.3135 | -1.2092 | 0.1722 | 0.446746 | 1 | 9702 | tags=31%, list=1 |
| GO_NEGAT | GO_NEGATIVE_REGU | 21 | -0.4984 | -1.3049 | 0.1723 | 0.414245 | 1 | 13862 | tags=33%, list=2 |
| GO_NEGAT | GO_NEGATIVE_REGU | 28 | -0.432 | -1.2761 | 0.1726 | 0.425066 | 1 | 6062 | tags=25%, list=1 |
| GO_MIRNA | GO_MIRNA_BINDING | 29 | -0.4914 | -1.3334 | 0.173 | 0.406924 | 1 | 9370 | tags=41%, list=1 |
| GO_NEGAT | GO_NEGATIVE_REGU | 37 | -0.42 | -1.2868 | 0.173 | 0.419461 | 1 | 12274 | tags=43%, list=2 |
| GO_CARBO | GO_CARBON_CARBC | 49 | -0.375 | -1.2575 | 0.1733 | 0.424007 | 1 | 10225 | tags=31%, list=1 |
| GO_NEGAT | GO_NEGATIVE_REGU | 27 | -0.4011 | -1.2654 | 0.1736 | 0.42535 | 1 | 12664 | tags=37%, list=2 |
| GO_OOGEN | GO_OOGENESIS | 82 | -0.3191 | -1.2185 | 0.1736 | 0.440635 | 1 | 8726 | tags=24%, list=1 |
| GO_TIGHT_ | GO_TIGHT_JUNCTIO | 125 | -0.3336 | -1.2759 | 0.1743 | 0.424995 | 1 | 5649 | tags=23%, list=1 |
| GO_CYCLI | GO_CYCLIC_NUCLEC | 36 | -0.3733 | -1.2386 | 0.1744 | 0.432894 | 1 | 20068 | tags=53%, list=3 |
| GO_ODONT | GO_ODONTOGENESI | 129 | -0.3349 | -1.2671 | 0.1751 | 0.428026 | 1 | 8585 | tags=31%, list=1 |
| GO_REGUL | GO_REGULATION_OI | 35 | -0.3918 | -1.231 | 0.1751 | 0.437538 | 1 | 17921 | tags=51%, list=3 |
| GO_RENAL | GO_RENAL_SYSTEM_ | 123 | -0.3214 | -1.2273 | 0.1753 | 0.43997 | 1 | 11922 | tags=32%, list=2 |
| GO_RESPO | GO_RESPONSE_TO_A | 69 | -0.3276 | -1.2159 | 0.1753 | 0.442415 | 1 | 10013 | tags=32%, list=1 |
| GO_LENS_ | GO_LENS_MORPHOG | 20 | -0.4322 | -1.2662 | 0.1758 | 0.426669 | 1 | 8997 | tags=40%, list=1 |
| GO_MONO | GO_MONOAMINE_TI | 82 | -0.3658 | -1.2438 | 0.1759 | 0.43278 | 1 | 11361 | tags=35%, list=1 |
| GO_MRNA_ | GO_MRNA_SPLICE_S | 48 | -0.452 | -1.2939 | 0.1765 | 0.416927 | 1 | 12984 | tags=46%, list=2 |
| GO_MEGA | GO_MEGAKARYOCY | 16 | -0.4724 | -1.2765 | 0.1765 | 0.425273 | 1 | 8217 | tags=38%, list=1 |
| GO_MESEN | GO_MESENCHYMAL_ | 20 | -0.4427 | -1.2812 | 0.1767 | 0.424079 | 1 | 5634 | tags=40%, list=1 |
| GO_PHOTO | GO_PHOTORECEPTO | 111 | -0.3242 | -1.2295 | 0.1777 | 0.437164 | 1 | 19648 | tags=45%, list=3 |
| GO_2_OXO | GO_2_OXOGLUTARA | 43 | -0.3808 | -1.2656 | 0.1779 | 0.425837 | 1 | 12094 | tags=35%, list=2 |
| GO_REGUL | GO_REGULATION_OI | 124 | -0.3228 | -1.2408 | 0.1783 | 0.43306 | 1 | 12950 | tags=38%, list=2 |
| GO_CATEC | GO_CATECHOLAMIN | 21 | -0.4149 | -1.2337 | 0.1786 | 0.436224 | 1 | 5221 | tags=24%, list=9 |
| GO_MODIF | GO_MODIFIED_AMIN | 20 | -0.4426 | -1.2874 | 0.1788 | 0.419141 | 1 | 7781 | tags=35%, list=1 |
| GO_DORSA | GO_DORSAL_SPINAL | 22 | -0.4983 | -1.2645 | 0.1789 | 0.425658 | 1 | 19979 | tags=64%, list=3 |
| GO_RESOL | GO_RESOLUTION_OI | 15 | -0.4976 | -1.2977 | 0.1789 | 0.417568 | 1 | 15158 | tags=47%, list=2 |

| | | | | | | | | | |
|---|---|---|---|---|---|---|---|---|---|
| GO_REGUL | GO_REGULATION_OI | 131 | -0.2979 | -1.2091 | 0.1789 | 0.446589 | 1 | 11233 | tags=27%, list=1 |
| GO_REGUL | GO_REGULATION_OI | 36 | -0.4043 | -1.277 | 0.1796 | 0.425526 | 1 | 9654 | tags=36%, list=1 |
| GO_REGUL | GO_REGULATION_OI | 478 | -0.2902 | -1.1844 | 0.18 | 0.458083 | 1 | 15397 | tags=40%, list=2 |
| GO_AXON_ | GO_AXON_CYTOPLA | 57 | -0.3807 | -1.2754 | 0.1802 | 0.425173 | 1 | 4046 | tags=23%, list=7 |
| GO_IMPOR | GO_IMPORT_ACROSS | 156 | -0.3044 | -1.2021 | 0.1807 | 0.448878 | 1 | 15498 | tags=37%, list=2 |
| GO_ION_CI | GO_ION_CHANNEL_I | 115 | -0.3227 | -1.212 | 0.1807 | 0.445571 | 1 | 6828 | tags=24%, list=1 |
| GO_SYNAP | GO_SYNAPTIC_VESI | 74 | -0.3548 | -1.2587 | 0.181 | 0.425389 | 1 | 9535 | tags=34%, list=1 |
| GO_POLY_ | GO_POLY_A_BINDIN | 23 | -0.4698 | -1.2978 | 0.1811 | 0.418367 | 1 | 10103 | tags=30%, list=1 |
| GO_MAGNI | GO_MAGNESIUM_IOI | 16 | -0.4921 | -1.3013 | 0.1814 | 0.415529 | 1 | 8736 | tags=44%, list=1 |
| GO_NEGAT | GO_NEGATIVE_REGU | 74 | -0.3567 | -1.2675 | 0.1815 | 0.428752 | 1 | 7971 | tags=27%, list=1 |
| GO_REGUL | GO_REGULATION_OI | 338 | -0.2955 | -1.1934 | 0.1816 | 0.452642 | 1 | 12141 | tags=35%, list=2 |
| GO_MITOC | GO_MITOCHONDRIO | 16 | -0.455 | -1.2712 | 0.1818 | 0.425275 | 1 | 18814 | tags=56%, list=3 |
| GO_CONNE | GO_CONNECTIVE_TI | 20 | -0.4527 | -1.2671 | 0.1818 | 0.42763 | 1 | 9470 | tags=35%, list=1 |
| GO_CELLU | GO_CELLULAR_GLU | 70 | -0.3262 | -1.2227 | 0.1818 | 0.442502 | 1 | 15406 | tags=43%, list=2 |
| GO_POSITI | GO_POSITIVE_REGUI | 49 | -0.4221 | -1.2911 | 0.182 | 0.417158 | 1 | 7830 | tags=39%, list=1 |
| GO_SENSOI | GO_SENSORY_PERCI | 217 | -0.2874 | -1.1811 | 0.182 | 0.460516 | 1 | 18301 | tags=39%, list=3 |
| GO_SODIUI | GO_SODIUM_CHANN | 44 | -0.3889 | -1.2445 | 0.1822 | 0.432274 | 1 | 11031 | tags=34%, list=1 |
| GO_ACROS | GO_ACROSOME_ASS | 15 | -0.4518 | -1.2696 | 0.1825 | 0.426975 | 1 | 13249 | tags=33%, list=2 |
| GO_RESPOI | GO_RESPONSE_TO_R | 107 | -0.2993 | -1.1929 | 0.1828 | 0.452711 | 1 | 4739 | tags=20%, list=8 |
| GO_CEREB | GO_CEREBRAL_COR | 112 | -0.3472 | -1.2463 | 0.183 | 0.432626 | 1 | 15403 | tags=39%, list=2 |
| GO_PITUIT | GO_PITUITARY_GLA | 42 | -0.3715 | -1.2066 | 0.1834 | 0.446669 | 1 | 19979 | tags=60%, list=3 |
| GO_POSITI | GO_POSITIVE_REGUI | 28 | -0.3945 | -1.2634 | 0.1839 | 0.423943 | 1 | 6716 | tags=29%, list=1 |
| GO_MICRO | GO_MICROTUBULE_I | 252 | -0.3553 | -1.2779 | 0.184 | 0.426179 | 1 | 14612 | tags=39%, list=2 |
| GO_SYNAP | GO_SYNAPTONEMAI | 40 | -0.4121 | -1.2639 | 0.1842 | 0.42383 | 1 | 17563 | tags=48%, list=3 |
| GO_NEGAT | GO_NEGATIVE_REGU | 30 | -0.3757 | -1.2218 | 0.1847 | 0.441899 | 1 | 19088 | tags=53%, list=3 |
| GO_RESPOI | GO_RESPONSE_TO_A | 234 | -0.2776 | -1.179 | 0.1848 | 0.46108 | 1 | 10151 | tags=26%, list=1 |
| GO_ANION | GO_ANION_CATION_ | 22 | -0.4214 | -1.24 | 0.185 | 0.43297 | 1 | 17190 | tags=59%, list=2 |
| GO_NEGAT | GO_NEGATIVE_REGU | 31 | -0.376 | -1.2313 | 0.185 | 0.437412 | 1 | 13442 | tags=35%, list=2 |
| GO_MATER | GO_MATERNAL_PLA | 36 | -0.3408 | -1.189 | 0.1858 | 0.455519 | 1 | 12819 | tags=39%, list=2 |
| GO_SWI_SI | GO_SWI_SNF_COMPI | 17 | -0.5231 | -1.3169 | 0.1859 | 0.408747 | 1 | 13852 | tags=59%, list=2 |
| GO_DNA_M | GO_DNA_METHYLAT | 70 | -0.3899 | -1.2673 | 0.1859 | 0.428673 | 1 | 16537 | tags=37%, list=2 |
| GO_BONE_ | GO_BONE_DEVELOP | 195 | -0.3367 | -1.255 | 0.186 | 0.425652 | 1 | 16573 | tags=43%, list=2 |
| GO_REGUL | GO_REGULATION_OI | 18 | -0.4561 | -1.264 | 0.186 | 0.424152 | 1 | 18362 | tags=44%, list=3 |
| GO_MYOFI | GO_MYOFILAMENT | 25 | -0.4502 | -1.2988 | 0.1862 | 0.41788 | 1 | 6864 | tags=28%, list=1 |
| GO_TRANS | GO_TRANSCYTOSIS | 20 | -0.4156 | -1.2443 | 0.1864 | 0.432198 | 1 | 3754 | tags=35%, list=6 |
| GO_COENZ | GO_COENZYME_A_M | 15 | -0.4898 | -1.323 | 0.1869 | 0.40984 | 1 | 643 | tags=20%, list=1 |
| GO_CYTOS | GO_CYTOSKELETON | 196 | -0.3504 | -1.2723 | 0.187 | 0.424164 | 1 | 8418 | tags=29%, list=1 |
| GO_GLUTA | GO_GLUTAMATE_RE | 101 | -0.3926 | -1.2381 | 0.1872 | 0.433073 | 1 | 13257 | tags=46%, list=2 |
| GO_ENTER | GO_ENTEROENDOCR | 27 | -0.4399 | -1.2807 | 0.1878 | 0.424439 | 1 | 11638 | tags=44%, list=2 |
| GO_POSITI | GO_POSITIVE_REGUI | 72 | -0.3326 | -1.2205 | 0.1878 | 0.442287 | 1 | 9470 | tags=31%, list=1 |
| GO_MAMM | GO_MAMMARY_GLA | 140 | -0.2837 | -1.2038 | 0.1879 | 0.448668 | 1 | 6873 | tags=24%, list=1 |
| GO_TRANS | GO_TRANSLATION_F | 16 | -0.5156 | -1.314 | 0.1886 | 0.40752 | 1 | 5527 | tags=31%, list=9 |
| GO_NEGAT | GO_NEGATIVE_REGU | 60 | -0.329 | -1.2011 | 0.1887 | 0.448543 | 1 | 12950 | tags=33%, list=2 |
| GO_POSTS | GO_POSTSYNAPTIC_ | 17 | -0.4526 | -1.2636 | 0.189 | 0.424007 | 1 | 11765 | tags=35%, list=2 |
| GO_CELLU | GO_CELLULAR_RESI | 57 | -0.3392 | -1.2195 | 0.1891 | 0.440979 | 1 | 11248 | tags=32%, list=1 |
| GO_POSTS | GO_POSTSYNAPTIC_ | 15 | -0.4468 | -1.2437 | 0.1899 | 0.432511 | 1 | 8907 | tags=40%, list=1 |
| GO_REGUL | GO_REGULATION_OI | 78 | -0.4225 | -1.3205 | 0.1902 | 0.409632 | 1 | 9389 | tags=36%, list=1 |
| GO_REGUL | GO_REGULATION_OI | 64 | -0.387 | -1.2645 | 0.1902 | 0.424946 | 1 | 10614 | tags=41%, list=1 |
| GO_BICARI | GO_BICARBONATE_T | 21 | -0.4359 | -1.2295 | 0.1902 | 0.437446 | 1 | 2930 | tags=19%, list=5 |
| GO_NEGAT | GO_NEGATIVE_REGU | 51 | -0.3361 | -1.2104 | 0.1908 | 0.446373 | 1 | 12398 | tags=37%, list=2 |
| GO_ORGAN | GO_ORGANELLE_TR | 82 | -0.3754 | -1.2686 | 0.191 | 0.427457 | 1 | 13484 | tags=41%, list=2 |
| GO_REGUL | GO_REGULATION_OI | 33 | -0.412 | -1.2656 | 0.1911 | 0.426097 | 1 | 14514 | tags=42%, list=2 |
| GO_POSITI | GO_POSITIVE_REGUI | 25 | -0.3972 | -1.2432 | 0.1912 | 0.432745 | 1 | 11248 | tags=40%, list=1 |
| GO_REGUL | GO_REGULATION_OI | 81 | -0.3359 | -1.2214 | 0.1918 | 0.441794 | 1 | 12556 | tags=35%, list=2 |
| GO_CORTI | GO_CORTICAL_CYTO | 105 | -0.3252 | -1.2272 | 0.1919 | 0.439716 | 1 | 11099 | tags=30%, list=1 |
| GO_KINESI | GO_KINESIN_COMPL | 50 | -0.4383 | -1.3122 | 0.1923 | 0.407613 | 1 | 14495 | tags=46%, list=2 |
| GO_CELL_I | GO_CELL_FATE_COM | 274 | -0.3029 | -1.207 | 0.1923 | 0.446284 | 1 | 15429 | tags=43%, list=2 |
| GO_MUSCI | GO_MUSCLE_ADAPT | 123 | -0.3048 | -1.1834 | 0.193 | 0.458166 | 1 | 11495 | tags=32%, list=2 |

| | | | | | | | | | |
|---|---|---|---|---|---|---|---|---|---|
| GO_CYTOS | GO_CYTOSKELETAL_ | 22 | -0.4364 | -1.2504 | 0.1932 | 0.430001 | 1 | 13008 | tags=45%, list=2 |
| GO_CELL_( | GO_CELL_COMMUNI | 31 | -0.423 | -1.2644 | 0.1932 | 0.424704 | 1 | 15184 | tags=42%, list=2 |
| GO_GLOME | GO_GLOMERULAR_E | 23 | -0.4287 | -1.2367 | 0.1933 | 0.43316 | 1 | 11652 | tags=30%, list=2 |
| GO_POSITI | GO_POSITIVE_REGU | 17 | -0.4342 | -1.2421 | 0.1936 | 0.433074 | 1 | 18926 | tags=59%, list=3 |
| GO_POSITI | GO_POSITIVE_REGU | 63 | -0.3436 | -1.222 | 0.1937 | 0.442705 | 1 | 10299 | tags=33%, list=1 |
| GO_REGUL | GO_REGULATION_OI | 46 | -0.342 | -1.2177 | 0.1937 | 0.441613 | 1 | 5697 | tags=28%, list=1 |
| GO_REGUL | GO_REGULATION_OI | 15 | -0.4955 | -1.2782 | 0.1938 | 0.426013 | 1 | 9127 | tags=40%, list=1 |
| GO_POSITI | GO_POSITIVE_REGU | 32 | -0.4536 | -1.2589 | 0.194 | 0.425436 | 1 | 6290 | tags=31%, list=1 |
| GO_LUNG_ | GO_LUNG_CELL_DIF | 23 | -0.4087 | -1.2238 | 0.194 | 0.442124 | 1 | 3835 | tags=22%, list=7 |
| GO_POSITI | GO_POSITIVE_REGU | 38 | -0.3687 | -1.2202 | 0.194 | 0.441844 | 1 | 10299 | tags=47%, list=1 |
| GO_N_MET | GO_N_METHYLTRAN | 84 | -0.3864 | -1.2817 | 0.1946 | 0.423502 | 1 | 12102 | tags=35%, list=2 |
| GO_POSITI | GO_POSITIVE_REGU | 41 | -0.4224 | -1.2768 | 0.1953 | 0.4255 | 1 | 7690 | tags=37%, list=1 |
| GO_CHEMC | GO_CHEMOREPELLE | 28 | -0.3664 | -1.2276 | 0.1954 | 0.439816 | 1 | 3046 | tags=25%, list=5 |
| GO_CYTOS | GO_CYTOSOLIC_TRA | 153 | -0.3452 | -1.2767 | 0.1956 | 0.425219 | 1 | 6767 | tags=27%, list=1 |
| GO_SIGNAI | GO_SIGNAL_TRANSD | 19 | -0.4425 | -1.2263 | 0.1957 | 0.439925 | 1 | 15534 | tags=58%, list=2 |
| GO_ANION_ | GO_ANION_CHANNE | 86 | -0.321 | -1.2013 | 0.196 | 0.448771 | 1 | 12825 | tags=38%, list=2 |
| GO_RENAL | GO_RENAL_SODIUM_ | 23 | -0.4319 | -1.2203 | 0.1962 | 0.442338 | 1 | 3514 | tags=22%, list=6 |
| GO_NEURC | GO_NEUROTRANSMI | 171 | -0.3634 | -1.2774 | 0.1964 | 0.425829 | 1 | 11615 | tags=38%, list=2 |
| GO_REGUL | GO_REGULATION_OI | 100 | -0.3026 | -1.1942 | 0.1965 | 0.452414 | 1 | 13258 | tags=30%, list=2 |
| GO_SKELET | GO_SKELETAL_MUS( | 41 | -0.3468 | -1.2081 | 0.1965 | 0.446111 | 1 | 11239 | tags=37%, list=1 |
| GO_DENTA | GO_DENTATE_GYRU | 16 | -0.491 | -1.2516 | 0.1969 | 0.429406 | 1 | 15403 | tags=56%, list=2 |
| GO_PLACEI | GO_PLACENTA_DEV | 155 | -0.2841 | -1.1865 | 0.1969 | 0.455792 | 1 | 10234 | tags=30%, list=1 |
| GO_PEPTID | GO_PEPTIDYL_THRE | 122 | -0.3409 | -1.233 | 0.1969 | 0.435457 | 1 | 12361 | tags=34%, list=2 |
| GO_MUSCL | GO_MUSCLE_MYOSI | 15 | -0.544 | -1.2931 | 0.1972 | 0.417932 | 1 | 7538 | tags=40%, list=1 |
| GO_EXTRA | GO_EXTRACELLULA | 41 | -0.5107 | -1.3236 | 0.1972 | 0.410395 | 1 | 14539 | tags=56%, list=2 |
| GO_CYCLI( | GO_CYCLIC_NUCLE( | 199 | -0.3232 | -1.2231 | 0.1972 | 0.442583 | 1 | 20038 | tags=55%, list=3 |
| GO_PHOTO | GO_PHOTORECEPTO | 23 | -0.3881 | -1.1785 | 0.1973 | 0.460115 | 1 | 14353 | tags=35%, list=2 |
| GO_ATP_DI | GO_ATP_DEPENDEN' | 16 | -0.5715 | -1.3477 | 0.1977 | 0.406608 | 1 | 24050 | tags=94%, list=4 |
| GO_POSITI | GO_POSITIVE_REGU | 18 | -0.4492 | -1.2392 | 0.1977 | 0.432898 | 1 | 10263 | tags=33%, list=1 |
| GO_NEURC | GO_NEUROTRANSMI | 22 | -0.4226 | -1.2426 | 0.1984 | 0.432934 | 1 | 24735 | tags=77%, list=4 |
| GO_MUSCL | GO_MUSCLE_SYSTEI | 456 | -0.3245 | -1.2549 | 0.1984 | 0.425372 | 1 | 11874 | tags=35%, list=2 |
| GO_NEGAT | GO_NEGATIVE_REGU | 29 | -0.432 | -1.2802 | 0.1984 | 0.424909 | 1 | 19665 | tags=55%, list=3 |
| GO_NUCLE | GO_NUCLEAR_RECE | 98 | -0.3299 | -1.2446 | 0.1985 | 0.432818 | 1 | 8314 | tags=32%, list=1 |
| GO_ACTIN_ | GO_ACTIN_MONOME | 25 | -0.4076 | -1.2428 | 0.1992 | 0.433007 | 1 | 4062 | tags=32%, list=7 |
| GO_PHOSPI | GO_PHOSPHORUS_O | 19 | -0.4391 | -1.2336 | 0.1992 | 0.435585 | 1 | 19084 | tags=63%, list=3 |
| GO_NEGAT | GO_NEGATIVE_REGU | 43 | -0.3403 | -1.2015 | 0.1996 | 0.449143 | 1 | 15514 | tags=44%, list=2 |
| GO_REGUL | GO_REGULATION_OI | 72 | -0.3965 | -1.2628 | 0.2 | 0.424251 | 1 | 13615 | tags=39%, list=2 |
| GO_CIS_GC | GO_CIS_GOLGI_NET\ | 68 | -0.3443 | -1.2246 | 0.2 | 0.441126 | 1 | 12821 | tags=31%, list=2 |
| GO_POSITI | GO_POSITIVE_REGU | 127 | -0.3075 | -1.1992 | 0.2 | 0.450778 | 1 | 10045 | tags=31%, list=1 |
| GO_NEGAT | GO_NEGATIVE_REGU | 39 | -0.3735 | -1.2371 | 0.2004 | 0.43359 | 1 | 13442 | tags=36%, list=2 |
| GO_DORSA | GO_DORSAL_VENTR | 24 | -0.4447 | -1.2666 | 0.2004 | 0.427288 | 1 | 10490 | tags=38%, list=1 |
| GO_CARBO | GO_CARBOHYDRATI | 16 | -0.4589 | -1.2617 | 0.2008 | 0.424208 | 1 | 19217 | tags=69%, list=3 |
| GO_METAN | GO_METANEPHRIC_I | 40 | -0.416 | -1.2739 | 0.2008 | 0.425581 | 1 | 7640 | tags=35%, list=1 |
| GO_ACTOM | GO_ACTOMYOSIN_S' | 196 | -0.3218 | -1.2279 | 0.2012 | 0.439498 | 1 | 10120 | tags=30%, list=1 |
| GO_TRANS | GO_TRANSFORMING | 24 | -0.4034 | -1.2336 | 0.2016 | 0.43595 | 1 | 4335 | tags=25%, list=7 |
| GO_EPHRIN | GO_EPHRIN_RECEPT | 27 | -0.3996 | -1.2495 | 0.2024 | 0.429532 | 1 | 3198 | tags=26%, list=5 |
| GO_CAMEF | GO_CAMERA_TYPE_ | 123 | -0.3033 | -1.1873 | 0.2028 | 0.456128 | 1 | 14688 | tags=38%, list=2 |
| GO_LEADIN | GO_LEADING_EDGE_ | 169 | -0.311 | -1.2005 | 0.2036 | 0.449154 | 1 | 13067 | tags=40%, list=2 |
| GO_GLAND | GO_GLANDULAR_EP | 25 | -0.4197 | -1.2387 | 0.2041 | 0.433204 | 1 | 6208 | tags=32%, list=1 |
| GO_NEURC | GO_NEURON_PROJE( | 146 | -0.3574 | -1.2415 | 0.2044 | 0.43217 | 1 | 15388 | tags=42%, list=2 |
| GO_MYOFI | GO_MYOFIBRIL_ASS | 66 | -0.3943 | -1.2552 | 0.2045 | 0.425634 | 1 | 10120 | tags=35%, list=1 |
| GO_REGUL | GO_REGULATION_OI | 25 | -0.4322 | -1.258 | 0.2046 | 0.42584 | 1 | 11448 | tags=44%, list=2 |
| GO_AXONA | GO_AXONAL_TRANS | 58 | -0.375 | -1.2574 | 0.2047 | 0.423747 | 1 | 4046 | tags=22%, list=7 |
| GO_ACTIVA | GO_ACTIVATION_OF | 106 | -0.3086 | -1.2078 | 0.205 | 0.445871 | 1 | 7306 | tags=25%, list=1 |
| GO_MICRO | GO_MICROTUBULE_ | 157 | -0.3546 | -1.2753 | 0.2051 | 0.424798 | 1 | 14495 | tags=39%, list=2 |
| GO_CELLU | GO_CELLULAR_GLU( | 148 | -0.2853 | -1.1736 | 0.2051 | 0.460382 | 1 | 11903 | tags=30%, list=2 |
| GO_REGUL | GO_REGULATION_OI | 113 | -0.2916 | -1.1686 | 0.2053 | 0.462906 | 1 | 14094 | tags=36%, list=2 |
| GO_MICRO | GO_MICROTUBULE_I | 33 | -0.4413 | -1.2616 | 0.2055 | 0.423953 | 1 | 8726 | tags=30%, list=1 |

| | | | | | | | | | |
|---|---|---|---|---|---|---|---|---|---|
| GO_GROWT | GO_GROWTH_FACTO | 129 | -0.3577 | -1.2528 | 0.2056 | 0.428018 | 1 | 8236 | tags=30%, list=1 |
| GO_DEMET | GO_DEMETHYLASE_ | 36 | -0.4327 | -1.2334 | 0.2058 | 0.43506 | 1 | 13642 | tags=47%, list=2 |
| GO_RESPO | GO_RESPONSE_TO_S | 35 | -0.3705 | -1.2268 | 0.2062 | 0.439597 | 1 | 3694 | tags=23%, list=6 |
| GO_TRANS | GO_TRANSPORT_AL | 157 | -0.353 | -1.258 | 0.2063 | 0.425504 | 1 | 8418 | tags=29%, list=1 |
| GO_RESPO | GO_RESPONSE_TO_F | 22 | -0.3859 | -1.2127 | 0.2064 | 0.445527 | 1 | 4301 | tags=32%, list=7 |
| GO_N_GLY | GO_N_GLYCAN_PRO | 18 | -0.4429 | -1.2094 | 0.2066 | 0.446779 | 1 | 9966 | tags=44%, list=1 |
| GO_APICAI | GO_APICAL_JUNCTIC | 137 | -0.3197 | -1.2269 | 0.2068 | 0.439938 | 1 | 2948 | tags=18%, list=5 |
| GO_SARCO | GO_SARCOMERE_OR | 44 | -0.3792 | -1.2217 | 0.207 | 0.441681 | 1 | 10120 | tags=32%, list=1 |
| GO_NEGAT | GO_NEGATIVE_REGU | 27 | -0.4049 | -1.2258 | 0.207 | 0.439664 | 1 | 18325 | tags=52%, list=3 |
| GO_NEGAT | GO_NEGATIVE_REGU | 16 | -0.4587 | -1.2309 | 0.2071 | 0.437375 | 1 | 7111 | tags=38%, list=1 |
| GO_RIBOSE | GO_RIBOSE_PHOSPH | 389 | -0.2733 | -1.1771 | 0.2072 | 0.457942 | 1 | 9901 | tags=26%, list=1 |
| GO_WATER | GO_WATER_HOMEOS | 79 | -0.3168 | -1.1797 | 0.2073 | 0.460488 | 1 | 15488 | tags=38%, list=2 |
| GO_POSITI | GO_POSITIVE_REGU | 25 | -0.367 | -1.1985 | 0.2079 | 0.451355 | 1 | 10444 | tags=32%, list=1 |
| GO_REGUL | GO_REGULATION_OI | 16 | -0.4285 | -1.2196 | 0.208 | 0.44127 | 1 | 8815 | tags=50%, list=1 |
| GO_NEURO | GO_NEUROPEPTIDE_ | 44 | -0.3788 | -1.1949 | 0.2084 | 0.453525 | 1 | 19614 | tags=57%, list=3 |
| GO_VASCU | GO_VASCULAR_ASS | 25 | -0.4505 | -1.2459 | 0.2087 | 0.432543 | 1 | 11664 | tags=48%, list=2 |
| GO_PROTEI | GO_PROTEIN_MANN | 20 | -0.446 | -1.2564 | 0.209 | 0.42441 | 1 | 22092 | tags=65%, list=3 |
| GO_REGUL | GO_REGULATION_OI | 89 | -0.3422 | -1.1939 | 0.209 | 0.452585 | 1 | 10366 | tags=40%, list=1 |
| GO_INORG | GO_INORGANIC_ION | 97 | -0.3062 | -1.1745 | 0.2093 | 0.459429 | 1 | 14248 | tags=36%, list=2 |
| GO_NEGAT | GO_NEGATIVE_REGU | 41 | -0.3631 | -1.213 | 0.2093 | 0.445732 | 1 | 2766 | tags=20%, list=5 |
| GO_RECEPT | GO_RECEPTOR_LOCA | 55 | -0.3997 | -1.2546 | 0.2096 | 0.425675 | 1 | 7952 | tags=33%, list=1 |
| GO_REGUL | GO_REGULATION_OI | 19 | -0.4115 | -1.222 | 0.2099 | 0.442365 | 1 | 6700 | tags=26%, list=1 |
| GO_XENOB | GO_XENOBIOTIC_TR | 27 | -0.3999 | -1.2145 | 0.2102 | 0.443813 | 1 | 4349 | tags=22%, list=7 |
| GO_REGUL | GO_REGULATION_OI | 18 | -0.4674 | -1.2525 | 0.2104 | 0.4283 | 1 | 3968 | tags=22%, list=7 |
| GO_UTERU | GO_UTERUS_DEVEL | 21 | -0.4184 | -1.2085 | 0.2104 | 0.44644 | 1 | 13941 | tags=38%, list=2 |
| GO_NUCLE | GO_NUCLEOSOME_B | 53 | -0.4611 | -1.3196 | 0.2108 | 0.407289 | 1 | 4301 | tags=28%, list=7 |
| GO_NEGAT | GO_NEGATIVE_REGU | 20 | -0.4224 | -1.2374 | 0.2112 | 0.433434 | 1 | 6429 | tags=30%, list=1 |
| GO_REGUL | GO_REGULATION_OI | 72 | -0.3477 | -1.1954 | 0.2113 | 0.452966 | 1 | 10366 | tags=42%, list=1 |
| GO_OTIC_V | GO_OTIC_VESICLE_D | 15 | -0.4813 | -1.2393 | 0.2114 | 0.433279 | 1 | 14539 | tags=60%, list=2 |
| GO_EMBRY | GO_EMBRYONIC_PLA | 88 | -0.3101 | -1.191 | 0.2114 | 0.452908 | 1 | 10234 | tags=34%, list=1 |
| GO_ISOPRE | GO_ISOPRENOID_ME | 143 | -0.297 | -1.1728 | 0.2116 | 0.459797 | 1 | 14868 | tags=35%, list=2 |
| GO_RAC_G | GO_RAC_GUANYL_N | 19 | -0.4398 | -1.2359 | 0.2123 | 0.433731 | 1 | 7772 | tags=37%, list=1 |
| GO_LEFT_F | GO_LEFT_RIGHT_PAT | 22 | -0.4116 | -1.219 | 0.2127 | 0.440838 | 1 | 14390 | tags=45%, list=2 |
| GO_REGUL | GO_REGULATION_OI | 100 | -0.3102 | -1.1794 | 0.2136 | 0.460746 | 1 | 11087 | tags=29%, list=1 |
| GO_LIGANI | GO_LIGAND_GATED_ | 139 | -0.3268 | -1.1921 | 0.2138 | 0.452436 | 1 | 14144 | tags=38%, list=2 |
| GO_NEGAT | GO_NEGATIVE_REGU | 16 | -0.4776 | -1.2578 | 0.214 | 0.425089 | 1 | 7372 | tags=44%, list=1 |
| GO_RETINO | GO_RETINOL_BINDIN | 16 | -0.4391 | -1.2098 | 0.215 | 0.447024 | 1 | 14556 | tags=50%, list=2 |
| GO_RESPO | GO_RESPONSE_TO_A | 103 | -0.2971 | -1.1691 | 0.2151 | 0.462968 | 1 | 8399 | tags=23%, list=1 |
| GO_POSITI | GO_POSITIVE_REGU | 49 | -0.3573 | -1.2023 | 0.2153 | 0.448886 | 1 | 16434 | tags=39%, list=2 |
| GO_CARTII | GO_CARTILAGE_DEV | 29 | -0.393 | -1.199 | 0.2154 | 0.450829 | 1 | 16214 | tags=48%, list=2 |
| GO_EXOCY | GO_EXOCYTIC_VESI | 216 | -0.3109 | -1.208 | 0.2158 | 0.446033 | 1 | 11013 | tags=34%, list=1 |
| GO_DRUG_ | GO_DRUG_BINDING | 101 | -0.2964 | -1.1591 | 0.2158 | 0.470019 | 1 | 10550 | tags=30%, list=1 |
| GO_REGUL | GO_REGULATION_OI | 101 | -0.3217 | -1.1776 | 0.216 | 0.459058 | 1 | 20619 | tags=49%, list=3 |
| GO_CELL_M | GO_CELL_MIGRATIO | 100 | -0.3236 | -1.1984 | 0.2161 | 0.450855 | 1 | 15403 | tags=32%, list=2 |
| GO_CILIAR | GO_CILIARY_BASE | 31 | -0.4089 | -1.2424 | 0.2162 | 0.432924 | 1 | 18128 | tags=55%, list=3 |
| GO_REGUL | GO_REGULATION_OI | 20 | -0.3827 | -1.1799 | 0.2162 | 0.4606 | 1 | 11652 | tags=35%, list=2 |
| GO_DNA_M | GO_DNA_METHYLAT | 18 | -0.4968 | -1.2692 | 0.2162 | 0.426836 | 1 | 6893 | tags=33%, list=1 |
| GO_EUKAR | GO_EUKARYOTIC_48 | 15 | -0.5701 | -1.3057 | 0.2164 | 0.41366 | 1 | 4823 | tags=33%, list=8 |
| GO_BASIC_ | GO_BASIC_AMINO_A | 15 | -0.4448 | -1.2147 | 0.2165 | 0.443735 | 1 | 6516 | tags=20%, list=1 |
| GO_SIN3_T | GO_SIN3_TYPE_COM | 15 | -0.5312 | -1.2826 | 0.2166 | 0.422676 | 1 | 8882 | tags=47%, list=1 |
| GO_VASCU | GO_VASCULAR_ASS | 37 | -0.3793 | -1.2097 | 0.218 | 0.446906 | 1 | 12067 | tags=32%, list=2 |
| GO_REGUL | GO_REGULATION_OI | 199 | -0.2839 | -1.1727 | 0.2182 | 0.459564 | 1 | 9901 | tags=30%, list=1 |
| GO_FATTY_ | GO_FATTY_ACYL_C | 38 | -0.3613 | -1.1917 | 0.2182 | 0.452348 | 1 | 12285 | tags=37%, list=2 |
| GO_INOSIT | GO_INOSITOL_PHOSI | 55 | -0.3426 | -1.2078 | 0.2184 | 0.445607 | 1 | 13788 | tags=45%, list=2 |
| GO_NEGAT | GO_NEGATIVE_REGU | 19 | -0.4025 | -1.2053 | 0.2186 | 0.447457 | 1 | 18653 | tags=42%, list=3 |
| GO_HORM | GO_HORMONE_ACTI | 118 | -0.3096 | -1.1285 | 0.2187 | 0.485538 | 1 | 18828 | tags=40%, list=3 |
| GO_PROTEI | GO_PROTEIN_DEMET | 26 | -0.4833 | -1.287 | 0.2188 | 0.419609 | 1 | 13642 | tags=54%, list=2 |
| GO_SULFU | GO_SULFUR_COMPO | 187 | -0.2846 | -1.1755 | 0.2189 | 0.459815 | 1 | 9865 | tags=25%, list=1 |

| | | | | | | | | | | |
|---|---|---|---|---|---|---|---|---|---|---|
| GO_NEGAT | GO_NEGATIVE_REGU | 19 | -0.4377 | -1.211 | 0.2191 | 0.446489 | 1 | 9318 | tags=32%, list=1 |
| GO_FOREB | GO_FOREBRAIN_GEN | 61 | -0.3702 | -1.2025 | 0.2198 | 0.449952 | 1 | 15403 | tags=49%, list=2 |
| GO_POSITI | GO_POSITIVE_REGU | 83 | -0.3179 | -1.1947 | 0.2198 | 0.452578 | 1 | 7870 | tags=30%, list=1 |
| GO_GLUTA | GO_GLUTAMATE_RE | 46 | -0.3625 | -1.2032 | 0.22 | 0.449538 | 1 | 15082 | tags=41%, list=2 |
| GO_REGUL | GO_REGULATION_OI | 261 | -0.2686 | -1.1376 | 0.2201 | 0.481501 | 1 | 12664 | tags=31%, list=2 |
| GO_PHOSPI | GO_PHOSPHATIDYLI | 18 | -0.5038 | -1.2945 | 0.2202 | 0.418467 | 1 | 9535 | tags=44%, list=1 |
| GO_HYALU | GO_HYALURONIC_A | 22 | -0.4485 | -1.2342 | 0.2208 | 0.436027 | 1 | 13904 | tags=59%, list=2 |
| GO_HISTON | GO_HISTONE_H3_AC | 58 | -0.3877 | -1.245 | 0.2214 | 0.433728 | 1 | 7417 | tags=31%, list=1 |
| GO_RESPOI | GO_RESPONSE_TO_M | 19 | -0.3844 | -1.1787 | 0.2216 | 0.460587 | 1 | 22566 | tags=58%, list=3 |
| GO_REGUL | GO_REGULATION_OI | 82 | -0.3207 | -1.1827 | 0.222 | 0.458513 | 1 | 4326 | tags=21%, list=7 |
| GO_POSITI | GO_POSITIVE_REGU | 37 | -0.3633 | -1.1802 | 0.2225 | 0.460373 | 1 | 17429 | tags=46%, list=3 |
| GO_CALCIU | GO_CALCIUM_ION_T | 133 | -0.3116 | -1.1731 | 0.2227 | 0.459887 | 1 | 11394 | tags=32%, list=2 |
| GO_DETEC | GO_DETECTION_OF_ | 74 | -0.2955 | -1.1403 | 0.2235 | 0.480682 | 1 | 18221 | tags=41%, list=3 |
| GO_REGUL | GO_REGULATION_OI | 248 | -0.3058 | -1.1945 | 0.2236 | 0.452478 | 1 | 11186 | tags=31%, list=1 |
| GO_GLYCO | GO_GLYCOSAMINOG | 219 | -0.3322 | -1.2024 | 0.224 | 0.449051 | 1 | 14201 | tags=38%, list=2 |
| GO_REGUL | GO_REGULATION_OI | 33 | -0.3903 | -1.2421 | 0.2241 | 0.432686 | 1 | 6954 | tags=21%, list=1 |
| GO_REGUL | GO_REGULATION_OI | 46 | -0.3564 | -1.2084 | 0.2243 | 0.445967 | 1 | 16180 | tags=37%, list=2 |
| GO_RESPOI | GO_RESPONSE_TO_T | 258 | -0.2976 | -1.1959 | 0.2248 | 0.453471 | 1 | 12361 | tags=33%, list=2 |
| GO_RESPOI | GO_RESPONSE_TO_E | 139 | -0.283 | -1.1509 | 0.2254 | 0.476927 | 1 | 11596 | tags=31%, list=2 |
| GO_CENTR | GO_CENTRAL_NERV | 36 | -0.3975 | -1.2159 | 0.2257 | 0.442761 | 1 | 19852 | tags=58%, list=3 |
| GO_POSITI | GO_POSITIVE_REGU | 22 | -0.4316 | -1.2059 | 0.2257 | 0.446743 | 1 | 1512 | tags=14%, list=3 |
| GO_AMINO | GO_AMINO_ACID_TR | 152 | -0.3036 | -1.1784 | 0.226 | 0.460067 | 1 | 9188 | tags=27%, list=1 |
| GO_TRANS | GO_TRANSLATION_F | 134 | -0.3461 | -1.2166 | 0.2261 | 0.442531 | 1 | 5882 | tags=21%, list=1 |
| GO_EPITHE | GO_EPITHELIAL_CEL | 116 | -0.3094 | -1.1868 | 0.2267 | 0.455832 | 1 | 3722 | tags=22%, list=6 |
| GO_APOLIF | GO_APOLIPOPROTEI | 17 | -0.4252 | -1.1925 | 0.227 | 0.452426 | 1 | 10013 | tags=35%, list=1 |
| GO_CARDI | GO_CARDIAC_MUSC | 39 | -0.3611 | -1.1872 | 0.2276 | 0.455819 | 1 | 15888 | tags=46%, list=2 |
| GO_HSP90_ | GO_HSP90_PROTEIN_ | 41 | -0.3559 | -1.2155 | 0.2277 | 0.442677 | 1 | 8021 | tags=34%, list=1 |
| GO_VASCU | GO_VASCULAR_WOU | 21 | -0.4437 | -1.2515 | 0.2279 | 0.429109 | 1 | 4750 | tags=29%, list=8 |
| GO_REGUL | GO_REGULATION_OI | 86 | -0.3296 | -1.1929 | 0.2285 | 0.452361 | 1 | 11374 | tags=31%, list=1 |
| GO_PHOSPI | GO_PHOSPHATIDYLI | 29 | -0.4313 | -1.2577 | 0.2295 | 0.424326 | 1 | 9535 | tags=38%, list=1 |
| GO_RESPOI | GO_RESPONSE_TO_L | 23 | -0.4041 | -1.2042 | 0.2297 | 0.449058 | 1 | 8635 | tags=30%, list=1 |
| GO_SERINE | GO_SERINE_FAMILY_ | 37 | -0.3736 | -1.1948 | 0.23 | 0.452989 | 1 | 10956 | tags=32%, list=1 |
| GO_NEGAT | GO_NEGATIVE_REGU | 107 | -0.3467 | -1.2211 | 0.2302 | 0.441949 | 1 | 10363 | tags=33%, list=1 |
| GO_REGUL | GO_REGULATION_OI | 166 | -0.3208 | -1.1982 | 0.2308 | 0.450879 | 1 | 11808 | tags=34%, list=2 |
| GO_NEGAT | GO_NEGATIVE_REGU | 15 | -0.4203 | -1.1949 | 0.2308 | 0.453172 | 1 | 15942 | tags=53%, list=2 |
| GO_GLUTA | GO_GLUTAMINE_FAI | 27 | -0.3785 | -1.1944 | 0.2312 | 0.452412 | 1 | 10225 | tags=30%, list=1 |
| GO_RESPOI | GO_RESPONSE_TO_D | 27 | -0.3587 | -1.1666 | 0.2314 | 0.464995 | 1 | 20508 | tags=56%, list=3 |
| GO_ORGAN | GO_ORGANIC_ACID_ | 148 | -0.2988 | -1.1652 | 0.2319 | 0.465603 | 1 | 9901 | tags=25%, list=1 |
| GO_OXIDO | GO_OXIDOREDUCTA | 159 | -0.2698 | -1.1353 | 0.2322 | 0.481953 | 1 | 12188 | tags=25%, list=2 |
| GO_EMBRY | GO_EMBRYONIC_CR | 48 | -0.3656 | -1.211 | 0.2323 | 0.446702 | 1 | 9208 | tags=35%, list=1 |
| GO_RESPOI | GO_RESPONSE_TO_A | 125 | -0.2985 | -1.1749 | 0.2326 | 0.459147 | 1 | 8989 | tags=27%, list=1 |
| GO_PROTEI | GO_PROTEIN_DEMET | 30 | -0.4343 | -1.2401 | 0.2329 | 0.433316 | 1 | 13642 | tags=50%, list=2 |
| GO_ANKYF | GO_ANKYRIN_BINDI | 20 | -0.4182 | -1.2015 | 0.2331 | 0.448792 | 1 | 18920 | tags=65%, list=3 |
| GO_IONOTI | GO_IONOTROPIC_GL | 26 | -0.4121 | -1.1934 | 0.2331 | 0.452309 | 1 | 23732 | tags=69%, list=4 |
| GO_CHONE | GO_CHONDROCYTE_ | 28 | -0.4297 | -1.2206 | 0.2339 | 0.44247 | 1 | 9810 | tags=39%, list=1 |
| GO_HISTON | GO_HISTONE_METHY | 54 | -0.4067 | -1.2345 | 0.2344 | 0.435886 | 1 | 11713 | tags=35%, list=2 |
| GO_PHOSPI | GO_PHOSPHOLIPID_I | 32 | -0.4118 | -1.233 | 0.2345 | 0.435124 | 1 | 9535 | tags=34%, list=1 |
| GO_REGUL | GO_REGULATION_OI | 42 | -0.333 | -1.1761 | 0.2354 | 0.458931 | 1 | 21540 | tags=60%, list=3 |
| GO_ORGAN | GO_ORGANIC_ACID_ | 161 | -0.2776 | -1.1276 | 0.2355 | 0.48557 | 1 | 9176 | tags=23%, list=1 |
| GO_REGUL | GO_REGULATION_OI | 70 | -0.3203 | -1.1518 | 0.2359 | 0.476023 | 1 | 12323 | tags=37%, list=2 |
| GO_COLLA | GO_COLLAGEN_TRIM | 87 | -0.4117 | -1.2503 | 0.2361 | 0.429829 | 1 | 14539 | tags=45%, list=2 |
| GO_PLASM | GO_PLASMA_MEMBI | 107 | -0.3191 | -1.1789 | 0.2361 | 0.460772 | 1 | 8343 | tags=32%, list=1 |
| GO_DICARI | GO_DICARBOXYLIC_ | 97 | -0.3123 | -1.1655 | 0.2361 | 0.465725 | 1 | 16390 | tags=41%, list=2 |
| GO_EPITHE | GO_EPITHELIAL_CEL | 215 | -0.2749 | -1.1405 | 0.2363 | 0.480644 | 1 | 11075 | tags=28%, list=1 |
| GO_GLOMI | GO_GLOMERULAR_M | 16 | -0.5029 | -1.2362 | 0.2364 | 0.433641 | 1 | 11652 | tags=63%, list=2 |
| GO_NOTCH | GO_NOTCH_SIGNALI | 187 | -0.2716 | -1.1415 | 0.2364 | 0.479803 | 1 | 13258 | tags=30%, list=2 |
| GO_RNA_P | GO_RNA_POLYMERA | 78 | -0.3479 | -1.206 | 0.2364 | 0.446897 | 1 | 12598 | tags=32%, list=2 |
| GO_AMEBC | GO_AMEBOIDAL_TY | 467 | -0.2672 | -1.1457 | 0.2364 | 0.479392 | 1 | 9908 | tags=27%, list=1 |

| | | | | | | | | | |
|---|---|---|---|---|---|---|---|---|---|
| GO_REGUL | GO_REGULATION_OI | 24 | -0.4355 | -1.2109 | 0.2368 | 0.446282 | 1 | 6893 | tags=33%, list=1 |
| GO_ADREN | GO_ADRENAL_GLAN | 24 | -0.3732 | -1.16 | 0.2373 | 0.469997 | 1 | 13841 | tags=42%, list=2 |
| GO_PEPTID | GO_PEPTIDYL_LYSIN | 43 | -0.4107 | -1.2227 | 0.2373 | 0.442865 | 1 | 15665 | tags=49%, list=2 |
| GO_POTAS | GO_POTASSIUM_ION | 48 | -0.3409 | -1.1572 | 0.2373 | 0.471419 | 1 | 10114 | tags=29%, list=1 |
| GO_ANIMA | GO_ANIMAL_ORGAN | 24 | -0.3852 | -1.1627 | 0.2378 | 0.467302 | 1 | 17571 | tags=50%, list=3 |
| GO_TASTE_ | GO_TASTE_RECEPTC | 26 | -0.4939 | -1.2497 | 0.2379 | 0.429643 | 1 | 13202 | tags=54%, list=2 |
| GO_CERAM | GO_CERAMIDE_BINI | 15 | -0.4142 | -1.2012 | 0.2379 | 0.448569 | 1 | 16638 | tags=60%, list=2 |
| GO_NEGAT | GO_NEGATIVE_REGU | 63 | -0.3321 | -1.1947 | 0.238 | 0.4528 | 1 | 10299 | tags=32%, list=1 |
| GO_DENDR | GO_DENDRITE_CYTC | 34 | -0.3833 | -1.2132 | 0.2383 | 0.445627 | 1 | 23782 | tags=68%, list=4 |
| GO_EMBRY | GO_EMBRYONIC_HE | 23 | -0.3822 | -1.1867 | 0.2386 | 0.45577 | 1 | 10083 | tags=39%, list=1 |
| GO_POSITI | GO_POSITIVE_REGUI | 29 | -0.4267 | -1.2334 | 0.2387 | 0.435439 | 1 | 6476 | tags=34%, list=1 |
| GO_CELL_( | GO_CELL_COMMUNI | 24 | -0.4118 | -1.1921 | 0.2387 | 0.452692 | 1 | 10114 | tags=33%, list=1 |
| GO_TAU_PI | GO_TAU_PROTEIN_K | 22 | -0.4476 | -1.2474 | 0.2388 | 0.431447 | 1 | 3847 | tags=27%, list=7 |
| GO_ALPHA | GO_ALPHA_AMINO_ | 64 | -0.3322 | -1.1731 | 0.2388 | 0.460183 | 1 | 7085 | tags=22%, list=1 |
| GO_MAMM | GO_MAMMARY_GLA | 71 | -0.2878 | -1.1574 | 0.2389 | 0.471608 | 1 | 6450 | tags=27%, list=1 |
| GO_PHOTO | GO_PHOTORECEPTO | 85 | -0.2962 | -1.1469 | 0.2392 | 0.479471 | 1 | 19521 | tags=41%, list=3 |
| GO_NADP_ | GO_NADP_BINDING | 53 | -0.3406 | -1.1925 | 0.2395 | 0.452715 | 1 | 6576 | tags=23%, list=1 |
| GO_POSITI | GO_POSITIVE_REGUI | 25 | -0.4207 | -1.2199 | 0.2402 | 0.441949 | 1 | 10863 | tags=40%, list=1 |
| GO_SPERM | GO_SPERM_MIDPIEC | 28 | -0.3592 | -1.1506 | 0.2402 | 0.476833 | 1 | 6841 | tags=25%, list=1 |
| GO_SIALYI | GO_SIALYLATION | 20 | -0.4332 | -1.1898 | 0.2403 | 0.454741 | 1 | 9966 | tags=35%, list=1 |
| GO_PHOTO | GO_PHOTORECEPTO | 57 | -0.3545 | -1.1655 | 0.2405 | 0.466031 | 1 | 15415 | tags=42%, list=2 |
| GO_2_OXO | GO_2_OXOGLUTARA | 15 | -0.4966 | -1.2399 | 0.2406 | 0.432834 | 1 | 11385 | tags=33%, list=1 |
| GO_CHRON | GO_CHROMOSOME_( | 61 | -0.3785 | -1.209 | 0.2406 | 0.446258 | 1 | 17563 | tags=46%, list=3 |
| GO_POSITI | GO_POSITIVE_REGUI | 20 | -0.4646 | -1.245 | 0.241 | 0.433386 | 1 | 25975 | tags=65%, list=4 |
| GO_POSITI | GO_POSITIVE_REGUI | 81 | -0.379 | -1.2096 | 0.241 | 0.4467 | 1 | 7830 | tags=32%, list=1 |
| GO_NEGAT | GO_NEGATIVE_REGU | 35 | -0.3462 | -1.1682 | 0.241 | 0.463218 | 1 | 12997 | tags=46%, list=2 |
| GO_REGUL | GO_REGULATION_OI | 367 | -0.2723 | -1.1539 | 0.2411 | 0.474589 | 1 | 8997 | tags=26%, list=1 |
| GO_CARDI | GO_CARDIOBLAST_I | 18 | -0.4417 | -1.2086 | 0.2411 | 0.446708 | 1 | 15096 | tags=50%, list=2 |
| GO_HISTON | GO_HISTONE_METHY | 17 | -0.4818 | -1.226 | 0.2412 | 0.439719 | 1 | 10083 | tags=35%, list=1 |
| GO_PROTE | GO_PROTEOGLYCAN | 64 | -0.3693 | -1.2041 | 0.2413 | 0.448979 | 1 | 13771 | tags=39%, list=2 |
| GO_PROTE | GO_PROTEIN_DEPOL | 108 | -0.3236 | -1.17 | 0.2414 | 0.462417 | 1 | 12556 | tags=32%, list=2 |
| GO_GLIAL_ | GO_GLIAL_CELL_DIF | 228 | -0.2811 | -1.1336 | 0.2417 | 0.48237 | 1 | 8692 | tags=24%, list=1 |
| GO_URONI | GO_URONIC_ACID_M | 23 | -0.4475 | -1.2219 | 0.2417 | 0.442161 | 1 | 7161 | tags=26%, list=1 |
| GO_ENDOP | GO_ENDOPLASMIC_I | 204 | -0.3121 | -1.1858 | 0.2418 | 0.455891 | 1 | 8051 | tags=25%, list=1 |
| GO_PROTE | GO_PROTEIN_METHY | 163 | -0.3682 | -1.2367 | 0.2422 | 0.43383 | 1 | 19156 | tags=49%, list=3 |
| GO_AXONE | GO_AXONEMAL_DYI | 20 | -0.4224 | -1.1835 | 0.2422 | 0.458305 | 1 | 11500 | tags=40%, list=2 |
| GO_VENTR | GO_VENTRICULAR_( | 17 | -0.4158 | -1.1777 | 0.2424 | 0.459607 | 1 | 8882 | tags=29%, list=1 |
| GO_NEGAT | GO_NEGATIVE_REGU | 59 | -0.3266 | -1.1808 | 0.2426 | 0.46067 | 1 | 11099 | tags=32%, list=1 |
| GO_MRNA_ | GO_MRNA_5_SPLICE | 23 | -0.556 | -1.294 | 0.2432 | 0.417172 | 1 | 12984 | tags=65%, list=2 |
| GO_CELLU | GO_CELLULAR_RESI | 26 | -0.4063 | -1.1879 | 0.2434 | 0.456391 | 1 | 22993 | tags=62%, list=3 |
| GO_SYMPC | GO_SYMPORTER_AC | 142 | -0.2669 | -1.1172 | 0.2436 | 0.489667 | 1 | 16401 | tags=39%, list=2 |
| GO_MICRO | GO_MICROTUBULE_I | 112 | -0.3535 | -1.219 | 0.2436 | 0.441211 | 1 | 7793 | tags=27%, list=1 |
| GO_NEGAT | GO_NEGATIVE_REGU | 23 | -0.3823 | -1.1593 | 0.2437 | 0.470059 | 1 | 17571 | tags=48%, list=3 |
| GO_RESPOI | GO_RESPONSE_TO_M | 200 | -0.2717 | -1.1418 | 0.2438 | 0.479986 | 1 | 12041 | tags=29%, list=2 |
| GO_ENDON | GO_ENDONUCLEASE | 36 | -0.4021 | -1.2129 | 0.2442 | 0.445465 | 1 | 5361 | tags=25%, list=9 |
| GO_THIOES | GO_THIOESTER_BIOS | 50 | -0.3546 | -1.1975 | 0.2444 | 0.451711 | 1 | 15930 | tags=40%, list=2 |
| GO_REGUL | GO_REGULATION_OI | 168 | -0.2758 | -1.1487 | 0.2447 | 0.477527 | 1 | 11628 | tags=30%, list=2 |
| GO_STRUC | GO_STRUCTURAL_C( | 27 | -0.516 | -1.2918 | 0.2449 | 0.417686 | 1 | 10044 | tags=44%, list=1 |
| GO_VENTR | GO_VENTRICULAR_( | 28 | -0.3931 | -1.1822 | 0.245 | 0.459062 | 1 | 15184 | tags=43%, list=2 |
| GO_POSITI | GO_POSITIVE_REGUI | 30 | -0.3694 | -1.1863 | 0.2452 | 0.455387 | 1 | 18362 | tags=57%, list=3 |
| GO_FIBROI | GO_FIBROBLAST_GR | 23 | -0.3776 | -1.1877 | 0.2454 | 0.456046 | 1 | 7101 | tags=26%, list=1 |
| GO_UBIQUI | GO_UBIQUITIN_CON. | 30 | -0.3814 | -1.1961 | 0.2455 | 0.453427 | 1 | 11621 | tags=40%, list=2 |
| GO_REGUL | GO_REGULATION_OI | 15 | -0.4558 | -1.2072 | 0.2458 | 0.446289 | 1 | 6442 | tags=40%, list=1 |
| GO_RESPOI | GO_RESPONSE_TO_C | 145 | -0.289 | -1.1387 | 0.2461 | 0.480193 | 1 | 11239 | tags=28%, list=1 |
| GO_MEIOTI | GO_MEIOTIC_CHRON | 80 | -0.3688 | -1.2199 | 0.2467 | 0.441599 | 1 | 21601 | tags=54%, list=3 |
| GO_NEGAT | GO_NEGATIVE_REGU | 26 | -0.4086 | -1.1863 | 0.2475 | 0.455708 | 1 | 15864 | tags=42%, list=2 |
| GO_CELLU | GO_CELLULAR_RESI | 71 | -0.3404 | -1.1985 | 0.2481 | 0.451077 | 1 | 8635 | tags=31%, list=1 |
| GO_POSITI | GO_POSITIVE_REGUI | 15 | -0.4583 | -1.192 | 0.2485 | 0.452156 | 1 | 14514 | tags=47%, list=2 |

| | | | | | | | | | |
|---|---|---|---|---|---|---|---|---|---|
| GO_SEX_DI | GO_SEX_DIFFERENT | 276 | -0.2693 | -1.1387 | 0.249 | 0.480453 | 1 | 13995 | tags=33%, list=2 |
| GO_PLATEI | GO_PLATELET_MORI | 21 | -0.3836 | -1.1732 | 0.2495 | 0.460412 | 1 | 6716 | tags=33%, list=1 |
| GO_NEGAT | GO_NEGATIVE_REGU | 29 | -0.3516 | -1.16 | 0.2495 | 0.469813 | 1 | 11233 | tags=28%, list=1 |
| GO_MUSCL | GO_MUSCLE_HYPER | 31 | -0.3563 | -1.1671 | 0.25 | 0.464549 | 1 | 11495 | tags=32%, list=2 |
| GO_VASCU | GO_VASCULAR_END | 48 | -0.3308 | -1.1608 | 0.25 | 0.469387 | 1 | 11210 | tags=25%, list=1 |
| GO_HISTON | GO_HISTONE_METHY | 132 | -0.3709 | -1.2114 | 0.2505 | 0.446372 | 1 | 16651 | tags=45%, list=2 |
| GO_ODONT | GO_ODONTOGENESIS | 89 | -0.3258 | -1.1773 | 0.2515 | 0.458943 | 1 | 10299 | tags=34%, list=1 |
| GO_FOREB | GO_FOREBRAIN_NEU | 51 | -0.3718 | -1.171 | 0.2515 | 0.461386 | 1 | 15008 | tags=49%, list=2 |
| GO_MUSCL | GO_MUSCLE_CELL_I | 239 | -0.293 | -1.1661 | 0.252 | 0.465596 | 1 | 9340 | tags=28%, list=1 |
| GO_MITOC | GO_MITOCHONDRIO | 45 | -0.3623 | -1.1804 | 0.2524 | 0.460215 | 1 | 5615 | tags=24%, list=1 |
| GO_RESPO | GO_RESPONSE_TO_E | 75 | -0.2926 | -1.1255 | 0.2524 | 0.486766 | 1 | 4349 | tags=20%, list=7 |
| GO_PEPTID | GO_PEPTIDYL_LYSIN | 113 | -0.373 | -1.2017 | 0.2524 | 0.44913 | 1 | 16445 | tags=48%, list=2 |
| GO_REGUL | GO_REGULATION_OI | 32 | -0.3304 | -1.1412 | 0.2524 | 0.480011 | 1 | 8094 | tags=22%, list=1 |
| GO_EYE_PI | GO_EYE_PHOTORECI | 35 | -0.345 | -1.1556 | 0.2526 | 0.472608 | 1 | 17875 | tags=51%, list=3 |
| GO_ENDOT | GO_ENDOTHELIAL_C | 191 | -0.2968 | -1.1782 | 0.253 | 0.460107 | 1 | 11210 | tags=30%, list=1 |
| GO_AXONA | GO_AXONAL_FASCIC | 23 | -0.3974 | -1.1767 | 0.2534 | 0.458241 | 1 | 8082 | tags=30%, list=1 |
| GO_REGUL | GO_REGULATION_OI | 25 | -0.4229 | -1.2166 | 0.2535 | 0.442901 | 1 | 10660 | tags=36%, list=1 |
| GO_EPHRIN | GO_EPHRIN_RECEPT | 87 | -0.3087 | -1.1541 | 0.2542 | 0.474634 | 1 | 6457 | tags=24%, list=1 |
| GO_REGUL | GO_REGULATION_OI | 232 | -0.3286 | -1.1955 | 0.2545 | 0.453165 | 1 | 8019 | tags=28%, list=1 |
| GO_NEGAT | GO_NEGATIVE_REGU | 152 | -0.2843 | -1.1241 | 0.2552 | 0.487577 | 1 | 18325 | tags=41%, list=3 |
| GO_RAC_G | GO_RAC_GTPASE_BI | 69 | -0.3382 | -1.1776 | 0.2559 | 0.459367 | 1 | 10477 | tags=35%, list=1 |
| GO_CILIAR | GO_CILIARY_TRANS | 63 | -0.3938 | -1.2167 | 0.2564 | 0.443044 | 1 | 18128 | tags=48%, list=3 |
| GO_MICRO | GO_MICROTUBULE_( | 29 | -0.444 | -1.2301 | 0.2568 | 0.437954 | 1 | 9173 | tags=34%, list=1 |
| GO_ACTIVI | GO_ACTIVE_TRANSM | 341 | -0.2545 | -1.1119 | 0.2569 | 0.491483 | 1 | 15786 | tags=36%, list=2 |
| GO_ESTABI | GO_ESTABLISHMENT | 214 | -0.2969 | -1.1639 | 0.2572 | 0.466068 | 1 | 12361 | tags=35%, list=2 |
| GO_INSULI | GO_INSULIN_RECEPT | 141 | -0.2861 | -1.135 | 0.2572 | 0.481877 | 1 | 12270 | tags=31%, list=2 |
| GO_HETER | GO_HETEROPHILIC_( | 42 | -0.3775 | -1.184 | 0.2573 | 0.45849 | 1 | 10254 | tags=38%, list=1 |
| GO_REGUL | GO_REGULATION_OI | 353 | -0.2737 | -1.1422 | 0.2574 | 0.479611 | 1 | 8997 | tags=26%, list=1 |
| GO_CELL_/ | GO_CELL_AGGREGA | 23 | -0.4255 | -1.1699 | 0.2577 | 0.462198 | 1 | 4391 | tags=26%, list=8 |
| GO_REGUL | GO_REGULATION_OI | 41 | -0.3429 | -1.152 | 0.2578 | 0.475873 | 1 | 15919 | tags=41%, list=2 |
| GO_CELLU | GO_CELLULAR_RESI | 201 | -0.2838 | -1.1361 | 0.2581 | 0.482057 | 1 | 10247 | tags=28%, list=1 |
| GO_NEURO | GO_NEURON_PROJE( | 26 | -0.3857 | -1.1839 | 0.2581 | 0.458301 | 1 | 11034 | tags=50%, list=1 |
| GO_ISOPRE | GO_ISOPRENOID_BIN | 37 | -0.3357 | -1.1172 | 0.2586 | 0.489446 | 1 | 20680 | tags=49%, list=3 |
| GO_CILIAR | GO_CILIARY_TIP | 45 | -0.4086 | -1.2262 | 0.259 | 0.439678 | 1 | 8248 | tags=36%, list=1 |
| GO_PHOTO | GO_PHOTORECEPTO | 47 | -0.326 | -1.1375 | 0.2592 | 0.48133 | 1 | 18128 | tags=49%, list=3 |
| GO_TRANS | GO_TRANSFERASE_/ | 248 | -0.2891 | -1.1574 | 0.2597 | 0.471336 | 1 | 12756 | tags=31%, list=2 |
| GO_CELL_( | GO_CELL_CELL_JUN | 476 | -0.2689 | -1.1426 | 0.2597 | 0.480882 | 1 | 8997 | tags=25%, list=1 |
| GO_TERPEI | GO_TERPENOID_MET | 124 | -0.2898 | -1.1277 | 0.2597 | 0.485735 | 1 | 14868 | tags=33%, list=2 |
| GO_ALPHA | GO_ALPHA_TUBULIN | 36 | -0.3655 | -1.1806 | 0.2604 | 0.460689 | 1 | 17190 | tags=42%, list=2 |
| GO_PHYSIC | GO_PHYSIOLOGICAL | 37 | -0.3475 | -1.1647 | 0.2608 | 0.466098 | 1 | 12950 | tags=35%, list=2 |
| GO_REGUL | GO_REGULATION_OI | 208 | -0.2721 | -1.1245 | 0.261 | 0.487253 | 1 | 11448 | tags=29%, list=2 |
| GO_REGUL | GO_REGULATION_OI | 15 | -0.4469 | -1.1873 | 0.2621 | 0.456366 | 1 | 10781 | tags=40%, list=1 |
| GO_VESICI | GO_VESICLE_COAT | 54 | -0.3572 | -1.1805 | 0.2628 | 0.460367 | 1 | 11813 | tags=37%, list=2 |
| GO_REGUL | GO_REGULATION_OI | 16 | -0.453 | -1.1889 | 0.2629 | 0.455209 | 1 | 6442 | tags=38%, list=1 |
| GO_STRIAT | GO_STRIATED_MUSC | 59 | -0.322 | -1.1448 | 0.2632 | 0.479553 | 1 | 10263 | tags=31%, list=1 |
| GO_POSITI | GO_POSITIVE_REGUI | 25 | -0.3922 | -1.1416 | 0.2635 | 0.479999 | 1 | 15047 | tags=52%, list=2 |
| GO_FILOPC | GO_FILOPODIUM_AS | 58 | -0.3112 | -1.1465 | 0.2641 | 0.479439 | 1 | 8956 | tags=28%, list=1 |
| GO_ENDOR | GO_ENDORIBONUCL | 30 | -0.4098 | -1.1911 | 0.2647 | 0.453044 | 1 | 5361 | tags=27%, list=9 |
| GO_INORG | GO_INORGANIC_ANI | 120 | -0.2871 | -1.1188 | 0.2652 | 0.48942 | 1 | 15498 | tags=43%, list=2 |
| GO_MICRO | GO_MICROTUBULE_I | 23 | -0.4534 | -1.2186 | 0.2653 | 0.440756 | 1 | 7579 | tags=30%, list=1 |
| GO_TRANS | GO_TRANSFORMING | 202 | -0.2869 | -1.1462 | 0.2653 | 0.479516 | 1 | 18362 | tags=44%, list=3 |
| GO_OVULA | GO_OVULATION | 21 | -0.4145 | -1.1744 | 0.2655 | 0.459222 | 1 | 8274 | tags=38%, list=1 |
| GO_CIRCAI | GO_CIRCADIAN_RHY | 215 | -0.3001 | -1.1533 | 0.2655 | 0.474636 | 1 | 12274 | tags=32%, list=2 |
| GO_RESPO | GO_RESPONSE_TO_C | 226 | -0.2647 | -1.1157 | 0.2655 | 0.489607 | 1 | 10951 | tags=27%, list=1 |
| GO_NEURO | GO_NEURON_CELLU | 32 | -0.3824 | -1.1688 | 0.2657 | 0.462918 | 1 | 18541 | tags=59%, list=3 |
| GO_LONG_ | GO_LONG_CHAIN_FA | 17 | -0.4016 | -1.1636 | 0.2661 | 0.466376 | 1 | 15873 | tags=41%, list=2 |
| GO_REGIOI | GO_REGION_OF_CYT | 25 | -0.4097 | -1.1937 | 0.2663 | 0.452446 | 1 | 18541 | tags=40%, list=3 |
| GO_OXYGE | GO_OXYGEN_BINDIN | 36 | -0.3487 | -1.1496 | 0.2664 | 0.477225 | 1 | 7577 | tags=19%, list=1 |

| | | | | | | | | | | |
|---|---|---|---|---|---|---|---|---|---|---|
| GO_REGUL | GO_REGULATION_OI | 388 | -0.2702 | -1.1261 | 0.267 | 0.486826 | 1 | 8997 | tags=26%, list=1 |
| GO_STEROI | GO_STEROID_BIOSYI | 193 | -0.2722 | -1.1315 | 0.2671 | 0.483421 | 1 | 4766 | tags=16%, list=8 |
| GO_REGUL | GO_REGULATION_OI | 204 | -0.2637 | -1.1105 | 0.2672 | 0.493112 | 1 | 4326 | tags=16%, list=7 |
| GO_HIPPO_ | GO_HIPPO_SIGNALIN | 44 | -0.3958 | -1.1958 | 0.2673 | 0.453042 | 1 | 13328 | tags=45%, list=2 |
| GO_RESPOI | GO_RESPONSE_TO_F | 36 | -0.3676 | -1.1605 | 0.2673 | 0.469498 | 1 | 7165 | tags=31%, list=1 |
| GO_ACYLG | GO_ACYLGLYCEROL | 22 | -0.3846 | -1.1594 | 0.2676 | 0.470232 | 1 | 10468 | tags=27%, list=1 |
| GO_HISTON | GO_HISTONE_LYSINI | 39 | -0.4309 | -1.2302 | 0.2676 | 0.438174 | 1 | 11713 | tags=38%, list=2 |
| GO_KERAT | GO_KERATINOCYTE_ | 19 | -0.4306 | -1.1682 | 0.2676 | 0.462944 | 1 | 9367 | tags=37%, list=1 |
| GO_NEGAT | GO_NEGATIVE_REGU | 24 | -0.4285 | -1.1832 | 0.2681 | 0.458116 | 1 | 12885 | tags=42%, list=2 |
| GO_REGUL | GO_REGULATION_OI | 34 | -0.3555 | -1.1494 | 0.2681 | 0.477331 | 1 | 13913 | tags=38%, list=2 |
| GO_PHOTO | GO_PHOTORECEPTO | 37 | -0.3879 | -1.1751 | 0.2682 | 0.45935 | 1 | 18128 | tags=51%, list=3 |
| GO_POSITI | GO_POSITIVE_REGUI | 15 | -0.4498 | -1.1779 | 0.2685 | 0.459548 | 1 | 9377 | tags=47%, list=1 |
| GO_POSITI | GO_POSITIVE_REGUI | 20 | -0.4071 | -1.1388 | 0.2687 | 0.480699 | 1 | 15095 | tags=45%, list=2 |
| GO_IMPOR | GO_IMPORT_INTO_C | 227 | -0.276 | -1.1146 | 0.2687 | 0.490837 | 1 | 8452 | tags=24%, list=1 |
| GO_APICAI | GO_APICAL_JUNCTIO | 81 | -0.3192 | -1.1632 | 0.2688 | 0.466709 | 1 | 14513 | tags=40%, list=2 |
| GO_ADHER | GO_ADHERENS_JUN( | 157 | -0.2862 | -1.1447 | 0.2692 | 0.479494 | 1 | 12361 | tags=31%, list=2 |
| GO_PHOTO | GO_PHOTORECEPTO | 62 | -0.3036 | -1.1171 | 0.2694 | 0.489019 | 1 | 18128 | tags=45%, list=3 |
| GO_ENDOT | GO_ENDOTHELIAL_( | 67 | -0.3449 | -1.1789 | 0.2695 | 0.460456 | 1 | 3722 | tags=24%, list=6 |
| GO_REGUL | GO_REGULATION_OI | 39 | -0.351 | -1.1462 | 0.2695 | 0.479204 | 1 | 13913 | tags=38%, list=2 |
| GO_SPLICE | GO_SPLICEOSOMAL_ | 27 | -0.4968 | -1.2196 | 0.2697 | 0.441604 | 1 | 9759 | tags=44%, list=1 |
| GO_FLUID_ | GO_FLUID_TRANSPO | 28 | -0.3724 | -1.1537 | 0.27 | 0.47425 | 1 | 12152 | tags=32%, list=2 |
| GO_CELLU | GO_CELLULAR_GLU( | 18 | -0.475 | -1.2024 | 0.2702 | 0.449699 | 1 | 7161 | tags=28%, list=1 |
| GO_REGUL | GO_REGULATION_OI | 136 | -0.2844 | -1.1427 | 0.2702 | 0.480967 | 1 | 10136 | tags=27%, list=1 |
| GO_ION_G/ | GO_ION_GATED_CH/ | 42 | -0.3504 | -1.153 | 0.2702 | 0.474844 | 1 | 9297 | tags=33%, list=1 |
| GO_SIALYI | GO_SIALYLTRANSFE | 21 | -0.412 | -1.1492 | 0.2703 | 0.477287 | 1 | 9966 | tags=33%, list=1 |
| GO_SOLUT | GO_SOLUTE_CATION | 32 | -0.337 | -1.1356 | 0.2703 | 0.482092 | 1 | 3579 | tags=19%, list=6 |
| GO_RESPOI | GO_RESPONSE_TO_II | 29 | -0.3318 | -1.1367 | 0.2706 | 0.482303 | 1 | 4301 | tags=24%, list=7 |
| GO_NEGAT | GO_NEGATIVE_REGU | 21 | -0.3815 | -1.1471 | 0.2707 | 0.479432 | 1 | 11087 | tags=43%, list=1 |
| GO_CELLU | GO_CELLULAR_RESI | 21 | -0.381 | -1.14 | 0.271 | 0.480478 | 1 | 7165 | tags=29%, list=1 |
| GO_REGUL | GO_REGULATION_OI | 19 | -0.4387 | -1.2026 | 0.2711 | 0.4502 | 1 | 12415 | tags=42%, list=2 |
| GO_WATEF | GO_WATER_TRANSP | 21 | -0.3959 | -1.1703 | 0.2714 | 0.462282 | 1 | 11581 | tags=29%, list=2 |
| GO_FORMA | GO_FORMATION_OF_ | 16 | -0.5044 | -1.2163 | 0.2715 | 0.442655 | 1 | 4823 | tags=31%, list=8 |
| GO_NEURO | GO_NEUROPEPTIDE_ | 33 | -0.345 | -1.1201 | 0.2717 | 0.490039 | 1 | 3611 | tags=18%, list=6 |
| GO_SPLICE | GO_SPLICEOSOMAL_ | 78 | -0.3741 | -1.1837 | 0.2717 | 0.458225 | 1 | 12984 | tags=37%, list=2 |
| GO_NEGAT | GO_NEGATIVE_REGU | 86 | -0.3272 | -1.1203 | 0.2718 | 0.490258 | 1 | 10853 | tags=33%, list=1 |
| GO_REGUL | GO_REGULATION_OI | 140 | -0.2639 | -1.104 | 0.272 | 0.494833 | 1 | 9806 | tags=28%, list=1 |
| GO_GENIT/ | GO_GENITALIA_DEV | 47 | -0.3472 | -1.1614 | 0.2724 | 0.468854 | 1 | 13941 | tags=43%, list=2 |
| GO_CLATH | GO_CLATHRIN_BIND | 64 | -0.3119 | -1.1352 | 0.2725 | 0.481732 | 1 | 12130 | tags=36%, list=2 |
| GO_CELLU | GO_CELLULAR_PRO( | 350 | -0.2657 | -1.1069 | 0.2727 | 0.494009 | 1 | 13279 | tags=29%, list=2 |
| GO_MICRO | GO_MICROTUBULE_I | 44 | -0.3975 | -1.1893 | 0.2729 | 0.455232 | 1 | 7793 | tags=30%, list=1 |
| GO_ORGAN | GO_ORGANIC_ACID_ | 346 | -0.2538 | -1.098 | 0.2733 | 0.497943 | 1 | 9188 | tags=23%, list=1 |
| GO_ANDRO | GO_ANDROGEN_REC | 28 | -0.3678 | -1.1627 | 0.2736 | 0.46704 | 1 | 4536 | tags=29%, list=8 |
| GO_CARBO | GO_CARBOHYDRATI | 235 | -0.2604 | -1.1048 | 0.2736 | 0.494751 | 1 | 12527 | tags=29%, list=2 |
| GO_COSTA | GO_COSTAMERE | 19 | -0.4731 | -1.2084 | 0.2737 | 0.446295 | 1 | 8533 | tags=47%, list=1 |
| GO_REGUL | GO_REGULATION_OI | 23 | -0.3768 | -1.1537 | 0.2738 | 0.47458 | 1 | 18131 | tags=57%, list=3 |
| GO_NUCLE | GO_NUCLEOSIDE_TR | 339 | -0.2752 | -1.1504 | 0.2738 | 0.476788 | 1 | 11448 | tags=30%, list=2 |
| GO_POSITI | GO_POSITIVE_REGUI | 23 | -0.4368 | -1.1958 | 0.274 | 0.453269 | 1 | 19653 | tags=61%, list=3 |
| GO_N_TERI | GO_N_TERMINAL_PF | 16 | -0.4276 | -1.1814 | 0.2741 | 0.460272 | 1 | 8564 | tags=38%, list=1 |
| GO_REGUL | GO_REGULATION_OI | 201 | -0.2881 | -1.1409 | 0.2744 | 0.480341 | 1 | 10490 | tags=32%, list=1 |
| GO_NEGAT | GO_NEGATIVE_REGU | 18 | -0.401 | -1.1643 | 0.2746 | 0.466166 | 1 | 8726 | tags=33%, list=1 |
| GO_ACTIVI | GO_ACTIVIN_BINDIN | 15 | -0.4432 | -1.1752 | 0.2752 | 0.459668 | 1 | 6239 | tags=27%, list=1 |
| GO_ALCOH | GO_ALCOHOL_BIOSY | 163 | -0.2887 | -1.1394 | 0.2752 | 0.480638 | 1 | 6020 | tags=19%, list=1 |
| GO_REGUL | GO_REGULATION_OI | 24 | -0.3901 | -1.1344 | 0.2761 | 0.481864 | 1 | 3514 | tags=21%, list=6 |
| GO_EPIDEF | GO_EPIDERMIS_MOF | 32 | -0.3829 | -1.1434 | 0.2764 | 0.480363 | 1 | 5708 | tags=25%, list=1 |
| GO_NEGAT | GO_NEGATIVE_REGU | 82 | -0.3244 | -1.1713 | 0.2765 | 0.461381 | 1 | 7095 | tags=26%, list=1 |
| GO_TISSUE | GO_TISSUE_MIGRAT | 362 | -0.269 | -1.1202 | 0.2766 | 0.490064 | 1 | 10485 | tags=28%, list=1 |
| GO_ANTER | GO_ANTEROGRADE_ | 47 | -0.3528 | -1.1729 | 0.2768 | 0.459892 | 1 | 15315 | tags=40%, list=2 |
| GO_ANDRO | GO_ANDROGEN_REC | 42 | -0.3575 | -1.1436 | 0.2768 | 0.480308 | 1 | 8314 | tags=31%, list=1 |

| | | | | | | | | | |
|---|---|---|---|---|---|---|---|---|---|
| GO_POSITI' | GO_POSITIVE_REGUI | 27 | -0.4258 | -1.178 | 0.2769 | 0.459719 | 1 | 19653 | tags=59%, list=3 |
| GO_NON_M | GO_NON_MOTILE_CI | 52 | -0.404 | -1.2188 | 0.2771 | 0.440716 | 1 | 20204 | tags=46%, list=3 |
| GO_MIRNA | GO_MIRNA_MEDIATI | 97 | -0.3465 | -1.171 | 0.2773 | 0.461652 | 1 | 15529 | tags=33%, list=2 |
| GO_ENDOD | GO_ENDODERM_DEV | 78 | -0.3377 | -1.1575 | 0.2773 | 0.471845 | 1 | 11075 | tags=36%, list=1 |
| GO_RESPO | GO_RESPONSE_TO_E | 40 | -0.3229 | -1.1283 | 0.2778 | 0.485154 | 1 | 11013 | tags=33%, list=1 |
| GO_GLAND | GO_GLANDULAR_EP | 47 | -0.3389 | -1.1389 | 0.278 | 0.481115 | 1 | 11638 | tags=36%, list=2 |
| GO_METAN | GO_METANEPHROS_ | 33 | -0.3544 | -1.1209 | 0.2784 | 0.489787 | 1 | 7165 | tags=30%, list=1 |
| GO_GTPASI | GO_GTPASE_REGUL/ | 300 | -0.2825 | -1.151 | 0.2784 | 0.477102 | 1 | 10059 | tags=29%, list=1 |
| GO_REGUL | GO_REGULATION_OI | 291 | -0.2676 | -1.113 | 0.2787 | 0.490823 | 1 | 9810 | tags=26%, list=1 |
| GO_REGUL | GO_REGULATION_OI | 39 | -0.3324 | -1.1281 | 0.2788 | 0.485194 | 1 | 10511 | tags=33%, list=1 |
| GO_CELLU | GO_CELLULAR_RESI | 44 | -0.3263 | -1.1259 | 0.2794 | 0.486671 | 1 | 10781 | tags=34%, list=1 |
| GO_BHLH_' | GO_BHLH_TRANSCR | 29 | -0.3534 | -1.1394 | 0.2812 | 0.480924 | 1 | 7826 | tags=34%, list=1 |
| GO_POSITI' | GO_POSITIVE_REGUI | 16 | -0.434 | -1.1718 | 0.2813 | 0.460882 | 1 | 18299 | tags=63%, list=3 |
| GO_MICRO | GO_MICROTUBULE | 418 | -0.3107 | -1.175 | 0.2814 | 0.459173 | 1 | 12302 | tags=31%, list=2 |
| GO_NEURC | GO_NEURON_FATE_S | 34 | -0.4464 | -1.1776 | 0.2814 | 0.458813 | 1 | 16459 | tags=47%, list=2 |
| GO_BIOMIN | GO_BIOMINERALIZA | 156 | -0.295 | -1.1041 | 0.2816 | 0.495032 | 1 | 14054 | tags=42%, list=2 |
| GO_SYNAP | GO_SYNAPTIC_VESIC | 119 | -0.3611 | -1.187 | 0.2816 | 0.455984 | 1 | 11615 | tags=37%, list=2 |
| GO_REGUL | GO_REGULATION_OI | 23 | -0.3897 | -1.1568 | 0.2819 | 0.471754 | 1 | 10185 | tags=30%, list=1 |
| GO_FEAR_I | GO_FEAR_RESPONSE | 41 | -0.3519 | -1.1498 | 0.2823 | 0.477281 | 1 | 15403 | tags=39%, list=2 |
| GO_PENTO | GO_PENTOSE_METAI | 15 | -0.4708 | -1.1772 | 0.2823 | 0.458469 | 1 | 10883 | tags=33%, list=1 |
| GO_SCAFF( | GO_SCAFFOLD_PROT | 60 | -0.3162 | -1.1324 | 0.2823 | 0.482903 | 1 | 12320 | tags=33%, list=2 |
| GO_NUCLE | GO_NUCLEOSIDE_BI | 131 | -0.2893 | -1.1198 | 0.2832 | 0.48927 | 1 | 15930 | tags=35%, list=2 |
| GO_OXIDO | GO_OXIDOREDUCTA | 35 | -0.3538 | -1.1353 | 0.2837 | 0.482182 | 1 | 3257 | tags=17%, list=6 |
| GO_PRESYI | GO_PRESYNAPTIC_A | 15 | -0.489 | -1.1753 | 0.284 | 0.459826 | 1 | 19869 | tags=60%, list=3 |
| GO_ENDOC | GO_ENDOCRINE_PAI | 45 | -0.3566 | -1.1643 | 0.284 | 0.466436 | 1 | 12398 | tags=42%, list=2 |
| GO_NUCLE | GO_NUCLEAR_EUCH | 25 | -0.4022 | -1.1548 | 0.2843 | 0.473714 | 1 | 12819 | tags=36%, list=2 |
| GO_MEMBI | GO_MEMBRANE_DEI | 88 | -0.3201 | -1.1256 | 0.2846 | 0.486828 | 1 | 11031 | tags=33%, list=1 |
| GO_HORM( | GO_HORMONE_MED | 195 | -0.2813 | -1.1159 | 0.2846 | 0.489762 | 1 | 10247 | tags=28%, list=1 |
| GO_SOLUT | GO_SOLUTE_PROTOI | 20 | -0.375 | -1.1366 | 0.2849 | 0.482237 | 1 | 1420 | tags=15%, list=2 |
| GO_HOMOI | GO_HOMOLOGOUS_( | 53 | -0.3452 | -1.1343 | 0.2849 | 0.481755 | 1 | 12772 | tags=32%, list=2 |
| GO_POSITI' | GO_POSITIVE_REGUI | 23 | -0.383 | -1.121 | 0.2849 | 0.489892 | 1 | 554 | tags=17%, list=1 |
| GO_POSITI' | GO_POSITIVE_REGUI | 67 | -0.2987 | -1.1026 | 0.2851 | 0.495208 | 1 | 3180 | tags=19%, list=5 |
| GO_BILE_A | GO_BILE_ACID_BIOS | 37 | -0.3264 | -1.1175 | 0.2852 | 0.490196 | 1 | 9049 | tags=27%, list=1 |
| GO_POSITI' | GO_POSITIVE_REGUI | 61 | -0.3438 | -1.1424 | 0.2854 | 0.480563 | 1 | 10254 | tags=36%, list=1 |
| GO_SODIUI | GO_SODIUM_ION_EX | 15 | -0.4135 | -1.1389 | 0.286 | 0.48092 | 1 | 14054 | tags=53%, list=2 |
| GO_REGUL | GO_REGULATION_OI | 113 | -0.2992 | -1.1223 | 0.2863 | 0.488576 | 1 | 6421 | tags=21%, list=1 |
| GO_REGUL | GO_REGULATION_OI | 50 | -0.3166 | -1.1132 | 0.2863 | 0.491723 | 1 | 12168 | tags=38%, list=2 |
| GO_NEGAT | GO_NEGATIVE_REGU | 17 | -0.3988 | -1.1357 | 0.2863 | 0.482128 | 1 | 19483 | tags=53%, list=3 |
| GO_PHOSPI | GO_PHOSPHATIDYLI | 31 | -0.3947 | -1.1735 | 0.2871 | 0.460272 | 1 | 9535 | tags=35%, list=1 |
| GO_UBIQUI | GO_UBIQUITIN_LIKE | 36 | -0.3528 | -1.1566 | 0.2877 | 0.471064 | 1 | 11621 | tags=36%, list=2 |
| GO_NEGAT | GO_NEGATIVE_REGU | 18 | -0.4171 | -1.1334 | 0.2882 | 0.482313 | 1 | 8426 | tags=44%, list=1 |
| GO_CELL_I | GO_CELL_LEADING_ | 411 | -0.2739 | -1.12 | 0.2884 | 0.48974 | 1 | 10885 | tags=30%, list=1 |
| GO_REGUL | GO_REGULATION_OI | 53 | -0.307 | -1.1169 | 0.2885 | 0.488931 | 1 | 5855 | tags=23%, list=1 |
| GO_NUCLE | GO_NUCLEAR_LOCA | 26 | -0.4479 | -1.1999 | 0.2886 | 0.449859 | 1 | 10044 | tags=38%, list=1 |
| GO_SUBPA | GO_SUBPALLIUM_DI | 24 | -0.4034 | -1.1508 | 0.2888 | 0.476801 | 1 | 24085 | tags=63%, list=4 |
| GO_NEGAT | GO_NEGATIVE_REGU | 39 | -0.3428 | -1.1236 | 0.289 | 0.487813 | 1 | 15095 | tags=36%, list=2 |
| GO_RETRO | GO_RETROGRADE_T | 83 | -0.3184 | -1.1462 | 0.2891 | 0.478897 | 1 | 6767 | tags=25%, list=1 |
| GO_RETIN( | GO_RETINOIC_ACID_ | 27 | -0.3572 | -1.1179 | 0.2891 | 0.490347 | 1 | 10781 | tags=41%, list=1 |
| GO_REGUL | GO_REGULATION_OI | 24 | -0.3955 | -1.166 | 0.2899 | 0.465386 | 1 | 19968 | tags=46%, list=3 |
| GO_NEGAT | GO_NEGATIVE_REGU | 18 | -0.3975 | -1.1383 | 0.2899 | 0.480502 | 1 | 11000 | tags=39%, list=1 |
| GO_NEGAT | GO_NEGATIVE_REGU | 15 | -0.3892 | -1.1206 | 0.2903 | 0.490085 | 1 | 9806 | tags=47%, list=1 |
| GO_PULMC | GO_PULMONARY_V/ | 21 | -0.4422 | -1.1879 | 0.2904 | 0.456733 | 1 | 15184 | tags=57%, list=2 |
| GO_G_PRO' | GO_G_PROTEIN_COU | 17 | -0.386 | -1.1158 | 0.2906 | 0.489667 | 1 | 6730 | tags=29%, list=1 |
| GO_NEGAT | GO_NEGATIVE_REGU | 73 | -0.3136 | -1.1145 | 0.2907 | 0.490793 | 1 | 12556 | tags=32%, list=2 |
| GO_POSITI' | GO_POSITIVE_REGUI | 15 | -0.4414 | -1.1429 | 0.2908 | 0.480916 | 1 | 9641 | tags=33%, list=1 |
| GO_AXO_D | GO_AXO_DENDRITIC | 69 | -0.3507 | -1.1879 | 0.2913 | 0.456157 | 1 | 15315 | tags=41%, list=2 |
| GO_REGUL | GO_REGULATION_OI | 29 | -0.3911 | -1.1476 | 0.2916 | 0.478762 | 1 | 6260 | tags=28%, list=1 |
| GO_PROTEI | GO_PROTEIN_LOCAI | 46 | -0.3601 | -1.1425 | 0.2916 | 0.480702 | 1 | 7952 | tags=30%, list=1 |

| | | | | | | | | | |
|---|---|---|---|---|---|---|---|---|---|
| GO_NEPHR | GO_NEPHRON_TUBU | 15 | -0.4153 | -1.1449 | 0.2917 | 0.479861 | 1 | 6716 | tags=40%, list=1 |
| GO_CHANN | GO_CHANNEL_REGU | 144 | -0.2902 | -1.1066 | 0.292 | 0.494204 | 1 | 6828 | tags=22%, list=1 |
| GO_MELAN | GO_MELANOCYTE_D | 25 | -0.4 | -1.1291 | 0.2941 | 0.485515 | 1 | 11664 | tags=40%, list=2 |
| GO_MYELI | GO_MYELIN_SHEATH | 46 | -0.3297 | -1.1261 | 0.2943 | 0.486512 | 1 | 4017 | tags=22%, list=7 |
| GO_NADPL | GO_NADPLUS_KINAS | 17 | -0.3818 | -1.131 | 0.2946 | 0.483369 | 1 | 9222 | tags=35%, list=1 |
| GO_DEVEL | GO_DEVELOPMENT_ | 226 | -0.2585 | -1.0965 | 0.2947 | 0.498538 | 1 | 12116 | tags=30%, list=2 |
| GO_EPITHE | GO_EPITHELIAL_CEL | 441 | -0.2505 | -1.1001 | 0.2949 | 0.497214 | 1 | 11210 | tags=28%, list=1 |
| GO_SMALL | GO_SMALL_SUBUNIT | 34 | -0.4243 | -1.161 | 0.2952 | 0.469225 | 1 | 12741 | tags=41%, list=2 |
| GO_ESTABI | GO_ESTABLISHMENT | 47 | -0.3151 | -1.1226 | 0.2955 | 0.488408 | 1 | 13615 | tags=34%, list=2 |
| GO_NEGAT | GO_NEGATIVE_REGU | 26 | -0.4079 | -1.1771 | 0.2956 | 0.458224 | 1 | 18369 | tags=35%, list=3 |
| GO_PLATEI | GO_PLATELET_DERIV | 59 | -0.3533 | -1.1642 | 0.2957 | 0.465971 | 1 | 8101 | tags=31%, list=1 |
| GO_NEGAT | GO_NEGATIVE_REGU | 15 | -0.3842 | -1.1287 | 0.2957 | 0.485473 | 1 | 8426 | tags=40%, list=1 |
| GO_REGUL | GO_REGULATION_OI | 28 | -0.4343 | -1.1772 | 0.2959 | 0.45878 | 1 | 12664 | tags=50%, list=2 |
| GO_DEFINI | GO_DEFINITIVE_HEM | 21 | -0.3831 | -1.1322 | 0.2967 | 0.482534 | 1 | 5294 | tags=29%, list=9 |
| GO_ACETY | GO_ACETYL_COA_BI | 22 | -0.4073 | -1.1567 | 0.2969 | 0.471209 | 1 | 15930 | tags=41%, list=2 |
| GO_NEGAT | GO_NEGATIVE_REGU | 16 | -0.4342 | -1.1597 | 0.2972 | 0.469934 | 1 | 10431 | tags=44%, list=1 |
| GO_ESTABI | GO_ESTABLISHMENT | 45 | -0.3607 | -1.1467 | 0.2976 | 0.479391 | 1 | 7372 | tags=29%, list=1 |
| GO_NEGAT | GO_NEGATIVE_REGU | 37 | -0.3748 | -1.1453 | 0.2977 | 0.479326 | 1 | 7103 | tags=24%, list=1 |
| GO_EXOCR | GO_EXOCRINE_SYST | 51 | -0.3104 | -1.1121 | 0.2978 | 0.491442 | 1 | 19965 | tags=45%, list=3 |
| GO_SYNTA | GO_SYNTAXIN_BIND | 78 | -0.3282 | -1.1324 | 0.2981 | 0.483089 | 1 | 8341 | tags=28%, list=1 |
| GO_RESPOI | GO_RESPONSE_TO_P | 16 | -0.4276 | -1.1522 | 0.2984 | 0.475944 | 1 | 15919 | tags=50%, list=2 |
| GO_REGUL | GO_REGULATION_OI | 202 | -0.2688 | -1.1073 | 0.2985 | 0.494202 | 1 | 10299 | tags=27%, list=1 |
| GO_POSITI | GO_POSITIVE_REGUI | 15 | -0.4192 | -1.135 | 0.2998 | 0.481571 | 1 | 18362 | tags=67%, list=3 |
| GO_METAN | GO_METANEPHRIC_I | 26 | -0.383 | -1.133 | 0.3 | 0.482474 | 1 | 7165 | tags=35%, list=1 |
| GO_LYASE | GO_LYASE_ACTIVITY | 171 | -0.2795 | -1.1034 | 0.3002 | 0.494684 | 1 | 10225 | tags=25%, list=1 |
| GO_GLYCO | GO_GLYCOPROTEIN_ | 329 | -0.2677 | -1.107 | 0.3002 | 0.494237 | 1 | 13066 | tags=29%, list=2 |
| GO_PHOSPI | GO_PHOSPHORIC_DI | 89 | -0.2867 | -1.0969 | 0.3002 | 0.498206 | 1 | 14223 | tags=36%, list=2 |
| GO_STRUC | GO_STRUCTURAL_C | 43 | -0.3893 | -1.1422 | 0.3006 | 0.479819 | 1 | 12596 | tags=44%, list=2 |
| GO_TRANS | GO_TRANSLATION_I | 50 | -0.3862 | -1.1689 | 0.301 | 0.463073 | 1 | 5882 | tags=22%, list=1 |
| GO_INWAR | GO_INWARD_RECTIF | 22 | -0.381 | -1.1174 | 0.3015 | 0.490013 | 1 | 8265 | tags=27%, list=1 |
| GO_REGUL | GO_REGULATION_OI | 26 | -0.3542 | -1.1138 | 0.3017 | 0.491324 | 1 | 22139 | tags=65%, list=3 |
| GO_REGUL | GO_REGULATION_OI | 32 | -0.3588 | -1.1198 | 0.3018 | 0.489578 | 1 | 694 | tags=16%, list=1 |
| GO_METAL | GO_METALLOENDOI | 104 | -0.303 | -1.1245 | 0.3024 | 0.487535 | 1 | 11456 | tags=30%, list=2 |
| GO_SYNAP | GO_SYNAPTIC_VESIC | 55 | -0.3555 | -1.1492 | 0.3028 | 0.477025 | 1 | 9535 | tags=38%, list=1 |
| GO_NUCLE | GO_NUCLEAR_HORM | 141 | -0.2963 | -1.119 | 0.3029 | 0.489594 | 1 | 8416 | tags=30%, list=1 |
| GO_ALPHA | GO_ALPHA_ACTININ | 27 | -0.3952 | -1.1322 | 0.303 | 0.48281 | 1 | 18359 | tags=59%, list=3 |
| GO_NADPH | GO_NADPH_BINDING | 16 | -0.4102 | -1.1442 | 0.303 | 0.480054 | 1 | 8553 | tags=31%, list=1 |
| GO_REGUL | GO_REGULATION_OI | 93 | -0.2962 | -1.1181 | 0.3034 | 0.490375 | 1 | 3722 | tags=20%, list=6 |
| GO_REGUL | GO_REGULATION_OI | 178 | -0.2863 | -1.1054 | 0.3037 | 0.49458 | 1 | 9641 | tags=28%, list=1 |
| GO_REGUL | GO_REGULATION_OI | 19 | -0.37 | -1.1229 | 0.3038 | 0.488072 | 1 | 10943 | tags=32%, list=1 |
| GO_SPINDI | GO_SPINDLE_MIDZO | 36 | -0.4294 | -1.2063 | 0.3039 | 0.446771 | 1 | 13727 | tags=36%, list=2 |
| GO_CELLU | GO_CELLULAR_RESP | 96 | -0.2966 | -1.1032 | 0.3039 | 0.494876 | 1 | 6062 | tags=19%, list=1 |
| GO_REGUL | GO_REGULATION_OI | 50 | -0.312 | -1.0965 | 0.3043 | 0.498268 | 1 | 16781 | tags=38%, list=2 |
| GO_APICAI | GO_APICAL_PART_O | 421 | -0.2552 | -1.0757 | 0.3047 | 0.511363 | 1 | 9810 | tags=24%, list=1 |
| GO_EXPLO | GO_EXPLORATION_E | 25 | -0.3679 | -1.1149 | 0.3049 | 0.490585 | 1 | 17607 | tags=52%, list=3 |
| GO_GROWT | GO_GROWTH_FACTO | 156 | -0.2961 | -1.0964 | 0.3054 | 0.498253 | 1 | 12526 | tags=35%, list=2 |
| GO_RESPOI | GO_RESPONSE_TO_E | 122 | -0.2624 | -1.0735 | 0.3055 | 0.513023 | 1 | 9793 | tags=25%, list=1 |
| GO_ORGAN | GO_ORGANELLE_INH | 15 | -0.4202 | -1.1333 | 0.3062 | 0.482227 | 1 | 5751 | tags=27%, list=1 |
| GO_ATPASI | GO_ATPASE_BINDING | 84 | -0.2731 | -1.0765 | 0.3062 | 0.510556 | 1 | 9306 | tags=25%, list=1 |
| GO_COMPL | GO_COMPLEX_OF_C | 21 | -0.5057 | -1.1654 | 0.3071 | 0.465486 | 1 | 14539 | tags=57%, list=2 |
| GO_POSITI | GO_POSITIVE_REGUI | 64 | -0.3227 | -1.1271 | 0.3072 | 0.48602 | 1 | 10462 | tags=36%, list=1 |
| GO_NEGAT | GO_NEGATIVE_REGU | 36 | -0.3381 | -1.1285 | 0.3074 | 0.485293 | 1 | 3347 | tags=22%, list=6 |
| GO_KERAT | GO_KERATAN_SULF | 33 | -0.3776 | -1.1423 | 0.3078 | 0.480111 | 1 | 8113 | tags=30%, list=1 |
| GO_FEMAL | GO_FEMALE_GAMET | 131 | -0.2712 | -1.0919 | 0.3084 | 0.501034 | 1 | 8726 | tags=22%, list=1 |
| GO_ACETY | GO_ACETYLTRANSFI | 103 | -0.3307 | -1.1583 | 0.3084 | 0.470666 | 1 | 9645 | tags=29%, list=1 |
| GO_VISUAI | GO_VISUAL_BEHAVI | 52 | -0.3225 | -1.0998 | 0.3086 | 0.496257 | 1 | 9704 | tags=29%, list=1 |
| GO_CELL_J | GO_CELL_JUNCTION | 30 | -0.3614 | -1.1219 | 0.3091 | 0.488931 | 1 | 18638 | tags=53%, list=3 |
| GO_SENSOI | GO_SENSORY_PERCI | 99 | -0.3121 | -1.1131 | 0.3091 | 0.491396 | 1 | 15154 | tags=42%, list=2 |

| | | | | | | | | | |
|---|---|---|---|---|---|---|---|---|---|
| GO_REGUL | GO_REGULATION_OI | 31 | -0.3336 | -1.09 | 0.3102 | 0.501565 | 1 | 17429 | tags=45%, list=3 |
| GO_POSITI | GO_POSITIVE_REGUI | 37 | -0.388 | -1.1455 | 0.3102 | 0.479378 | 1 | 16562 | tags=41%, list=2 |
| GO_PROTEI | GO_PROTEIN_STABII | 179 | -0.2708 | -1.0976 | 0.3105 | 0.497942 | 1 | 8168 | tags=23%, list=1 |
| GO_RESPOI | GO_RESPONSE_TO_P | 147 | -0.2737 | -1.0811 | 0.3106 | 0.506969 | 1 | 10013 | tags=28%, list=1 |
| GO_I_BANI | GO_I_BAND | 139 | -0.34 | -1.1586 | 0.3106 | 0.470578 | 1 | 14188 | tags=40%, list=2 |
| GO_POSITI | GO_POSITIVE_REGUI | 90 | -0.2999 | -1.1162 | 0.3109 | 0.489542 | 1 | 10299 | tags=33%, list=1 |
| GO_GERM_ | GO_GERM_CELL_DE | 253 | -0.2661 | -1.0852 | 0.3109 | 0.505008 | 1 | 13279 | tags=30%, list=2 |
| GO_N_TERI | GO_N_TERMINAL_PF | 28 | -0.3756 | -1.1365 | 0.3111 | 0.482032 | 1 | 10505 | tags=36%, list=1 |
| GO_NEGAT | GO_NEGATIVE_REGU | 46 | -0.3005 | -1.0879 | 0.3112 | 0.50206 | 1 | 9653 | tags=30%, list=1 |
| GO_CAMP_ | GO_CAMP_METABOI | 22 | -0.3774 | -1.113 | 0.3115 | 0.491112 | 1 | 17430 | tags=45%, list=3 |
| GO_REGUL | GO_REGULATION_OI | 15 | -0.4352 | -1.1481 | 0.3124 | 0.478191 | 1 | 2535 | tags=27%, list=4 |
| GO_ESTABI | GO_ESTABLISHMENT | 15 | -0.3858 | -1.1075 | 0.3124 | 0.494483 | 1 | 13615 | tags=40%, list=2 |
| GO_REGUL | GO_REGULATION_OI | 15 | -0.4011 | -1.1176 | 0.3125 | 0.490275 | 1 | 9340 | tags=33%, list=1 |
| GO_90S_PR | GO_90S_PRERIBOSOI | 26 | -0.4474 | -1.1568 | 0.3126 | 0.471422 | 1 | 3054 | tags=23%, list=5 |
| GO_NEGAT | GO_NEGATIVE_REGU | 66 | -0.3033 | -1.0999 | 0.3135 | 0.496252 | 1 | 22995 | tags=59%, list=3 |
| GO_EXTRA | GO_EXTRACELLULA | 31 | -0.3629 | -1.0958 | 0.3141 | 0.498352 | 1 | 13011 | tags=39%, list=2 |
| GO_POSITI | GO_POSITIVE_REGUI | 33 | -0.3583 | -1.1198 | 0.3145 | 0.489791 | 1 | 7579 | tags=27%, list=1 |
| GO_NEGAT | GO_NEGATIVE_REGU | 39 | -0.3314 | -1.1002 | 0.3146 | 0.497409 | 1 | 6429 | tags=28%, list=1 |
| GO_EXTRA | GO_EXTRACELLULA | 44 | -0.4192 | -1.1785 | 0.3149 | 0.460425 | 1 | 10198 | tags=41%, list=1 |
| GO_POSITI | GO_POSITIVE_REGUI | 43 | -0.3268 | -1.104 | 0.315 | 0.494638 | 1 | 3501 | tags=19%, list=6 |
| GO_RECEP | GO_RECEPTOR_CLUS | 55 | -0.3325 | -1.1265 | 0.3153 | 0.486778 | 1 | 8836 | tags=33%, list=1 |
| GO_CELL_( | GO_CELL_CORTEX_F | 42 | -0.3433 | -1.1082 | 0.3156 | 0.494617 | 1 | 11439 | tags=36%, list=2 |
| GO_NEURO | GO_NEUROTRANSMI | 41 | -0.3309 | -1.0933 | 0.3158 | 0.500075 | 1 | 8909 | tags=29%, list=1 |
| GO_BASAL | GO_BASAL_TRANSCI | 71 | -0.3325 | -1.1499 | 0.3161 | 0.477489 | 1 | 12067 | tags=30%, list=2 |
| GO_CHLOR | GO_CHLORIDE_TRAN | 110 | -0.2823 | -1.08 | 0.3167 | 0.507064 | 1 | 13975 | tags=39%, list=2 |
| GO_NEURO | GO_NEURON_PROJE( | 87 | -0.312 | -1.119 | 0.3172 | 0.489428 | 1 | 5721 | tags=21%, list=1 |
| GO_CELL_( | GO_CELL_CELL_ADH | 26 | -0.3477 | -1.1075 | 0.3172 | 0.494274 | 1 | 21835 | tags=54%, list=3 |
| GO_REGUL | GO_REGULATION_OI | 284 | -0.2598 | -1.0954 | 0.3173 | 0.498493 | 1 | 8171 | tags=23%, list=1 |
| GO_PROTEI | GO_PROTEIN_LOCAL | 65 | -0.2966 | -1.0802 | 0.3175 | 0.507082 | 1 | 11983 | tags=38%, list=2 |
| GO_PLATEI | GO_PLATELET_ALPH | 17 | -0.4177 | -1.1196 | 0.3176 | 0.488919 | 1 | 9024 | tags=35%, list=1 |
| GO_MULTI( | GO_MULTICELLULAI | 76 | -0.3064 | -1.0995 | 0.3179 | 0.496187 | 1 | 15514 | tags=41%, list=2 |
| GO_REGUL | GO_REGULATION_OI | 24 | -0.3613 | -1.1345 | 0.3179 | 0.482104 | 1 | 3119 | tags=29%, list=5 |
| GO_HISTON | GO_HISTONE_H3_K4_ | 53 | -0.404 | -1.1697 | 0.3181 | 0.462247 | 1 | 17788 | tags=51%, list=3 |
| GO_REGUL | GO_REGULATION_OI | 30 | -0.3504 | -1.1092 | 0.3182 | 0.494351 | 1 | 9127 | tags=33%, list=1 |
| GO_CALCIN | GO_CALCINEURIN_N | 47 | -0.3125 | -1.096 | 0.3183 | 0.498313 | 1 | 13788 | tags=40%, list=2 |
| GO_ENDOT | GO_ENDOTHELIAL_C | 62 | -0.3411 | -1.1288 | 0.3185 | 0.485706 | 1 | 8947 | tags=34%, list=1 |
| GO_STEROI | GO_STEROID_HORM( | 135 | -0.2916 | -1.0975 | 0.3185 | 0.497492 | 1 | 10247 | tags=29%, list=1 |
| GO_REGUL | GO_REGULATION_OI | 31 | -0.353 | -1.1082 | 0.3194 | 0.494922 | 1 | 12168 | tags=45%, list=2 |
| GO_REGUL | GO_REGULATION_OI | 15 | -0.4208 | -1.123 | 0.3196 | 0.488277 | 1 | 20576 | tags=53%, list=3 |
| GO_THIOES | GO_THIOESTER_MET | 98 | -0.294 | -1.0887 | 0.32 | 0.501734 | 1 | 15930 | tags=35%, list=2 |
| GO_HIPPO( | GO_HIPPOCAMPAL_N | 32 | -0.3722 | -1.1106 | 0.3207 | 0.4931 | 1 | 19653 | tags=53%, list=3 |
| GO_REGUL | GO_REGULATION_OI | 20 | -0.3465 | -1.0956 | 0.3207 | 0.498339 | 1 | 8635 | tags=30%, list=1 |
| GO_SIGNAI | GO_SIGNAL_SEQUEN | 47 | -0.3686 | -1.14 | 0.3208 | 0.480259 | 1 | 10044 | tags=32%, list=1 |
| GO_O_ACY | GO_O_ACYLTRANSFI | 47 | -0.3152 | -1.0915 | 0.3209 | 0.500777 | 1 | 13412 | tags=32%, list=2 |
| GO_PROTEI | GO_PROTEIN_C_TER | 186 | -0.2767 | -1.1063 | 0.3214 | 0.493967 | 1 | 6085 | tags=22%, list=1 |
| GO_BONE_ | GO_BONE_MINERAL | 112 | -0.2983 | -1.0843 | 0.3216 | 0.505311 | 1 | 14054 | tags=43%, list=2 |
| GO_REGUL | GO_REGULATION_OI | 365 | -0.2653 | -1.0727 | 0.3218 | 0.514162 | 1 | 8941 | tags=29%, list=1 |
| GO_POSITI | GO_POSITIVE_REGUI | 45 | -0.3235 | -1.0931 | 0.3219 | 0.499836 | 1 | 10299 | tags=42%, list=1 |
| GO_RESPOI | GO_RESPONSE_TO_D | 87 | -0.2827 | -1.0649 | 0.3219 | 0.519177 | 1 | 19410 | tags=46%, list=3 |
| GO_REGUL | GO_REGULATION_OI | 19 | -0.4233 | -1.1176 | 0.3221 | 0.490578 | 1 | 6893 | tags=37%, list=1 |
| GO_MUSCL | GO_MUSCLE_CELL_N | 103 | -0.3037 | -1.1046 | 0.3232 | 0.494812 | 1 | 8635 | tags=24%, list=1 |
| GO_GLIAL_ | GO_GLIAL_CELL_MI( | 53 | -0.316 | -1.0976 | 0.3233 | 0.497721 | 1 | 6187 | tags=23%, list=1 |
| GO_INSULI | GO_INSULIN_LIKE_G | 35 | -0.3278 | -1.0845 | 0.3235 | 0.50533 | 1 | 12950 | tags=34%, list=2 |
| GO_MANN( | GO_MANNOSYLTRAI | 28 | -0.3644 | -1.1212 | 0.3237 | 0.490004 | 1 | 15915 | tags=43%, list=2 |
| GO_MUSCL | GO_MUSCLE_FIBER_ | 56 | -0.3245 | -1.0952 | 0.3241 | 0.498551 | 1 | 9641 | tags=30%, list=1 |
| GO_MRNA_ | GO_MRNA_CIS_SPLI( | 52 | -0.3781 | -1.1293 | 0.3242 | 0.485414 | 1 | 12984 | tags=42%, list=2 |
| GO_GLUCU | GO_GLUCURONOSYI | 30 | -0.3591 | -1.1076 | 0.3244 | 0.494666 | 1 | 12247 | tags=37%, list=2 |
| GO_MEGAI | GO_MEGAKARYOCY | 70 | -0.3217 | -1.1091 | 0.3245 | 0.494155 | 1 | 10083 | tags=33%, list=1 |

| | | | | | | | | | | |
|---|---|---|---|---|---|---|---|---|---|---|
| GO_REGUL | GO_REGULATION_OI | 101 | -0.2956 | -1.0904 | 0.3245 | 0.50154 | 1 | 8907 | tags=28%, list=1 |
| GO_NEUTR | GO_NEUTRAL_LIPID_ | 48 | -0.3243 | -1.0803 | 0.3245 | 0.507207 | 1 | 13220 | tags=33%, list=2 |
| GO_INORG | GO_INORGANIC_ANI | 170 | -0.2588 | -1.0618 | 0.3248 | 0.519336 | 1 | 10550 | tags=27%, list=1 |
| GO_PROTEI | GO_PROTEIN_KINASI | 30 | -0.3345 | -1.0916 | 0.325 | 0.500902 | 1 | 13378 | tags=47%, list=2 |
| GO_PHOSPI | GO_PHOSPHOPROTEI | 85 | -0.2939 | -1.0835 | 0.3251 | 0.505876 | 1 | 7287 | tags=28%, list=1 |
| GO_REGUL | GO_REGULATION_OI | 228 | -0.2602 | -1.0824 | 0.3252 | 0.506702 | 1 | 9450 | tags=25%, list=1 |
| GO_RESPOI | GO_RESPONSE_TO_S | 340 | -0.244 | -1.063 | 0.3258 | 0.518913 | 1 | 10247 | tags=25%, list=1 |
| GO_SYNAP | GO_SYNAPSE_MATU | 25 | -0.4211 | -1.1309 | 0.3261 | 0.483229 | 1 | 11013 | tags=40%, list=1 |
| GO_POTAS | GO_POTASSIUM_ION | 16 | -0.3805 | -1.0884 | 0.3262 | 0.502074 | 1 | 15374 | tags=38%, list=2 |
| GO_REGUL | GO_REGULATION_OI | 17 | -0.4621 | -1.1311 | 0.3267 | 0.483571 | 1 | 7430 | tags=35%, list=1 |
| GO_HISTON | GO_HISTONE_H3_K9_ | 48 | -0.3713 | -1.1301 | 0.3269 | 0.484227 | 1 | 15678 | tags=46%, list=2 |
| GO_AMINO | GO_AMINO_ACID_TF | 80 | -0.3012 | -1.0888 | 0.3272 | 0.501973 | 1 | 4015 | tags=18%, list=7 |
| GO_CHROM | GO_CHROMATIN_DN | 89 | -0.3417 | -1.1231 | 0.3275 | 0.488388 | 1 | 5294 | tags=24%, list=9 |
| GO_POSITI | GO_POSITIVE_REGUI | 24 | -0.3842 | -1.0945 | 0.3281 | 0.498538 | 1 | 14609 | tags=46%, list=2 |
| GO_NEGAT | GO_NEGATIVE_REGU | 21 | -0.3526 | -1.0894 | 0.3281 | 0.501497 | 1 | 7754 | tags=38%, list=1 |
| GO_SEMAP | GO_SEMAPHORIN_RI | 23 | -0.3462 | -1.0921 | 0.3286 | 0.500869 | 1 | 3046 | tags=26%, list=5 |
| GO_REGUL | GO_REGULATION_OI | 93 | -0.2876 | -1.0793 | 0.33 | 0.507448 | 1 | 16570 | tags=39%, list=2 |
| GO_AMINO | GO_AMINO_SUGAR_ | 39 | -0.3384 | -1.1018 | 0.3301 | 0.496302 | 1 | 8437 | tags=28%, list=1 |
| GO_INTRA | GO_INTRACELLULAI | 112 | -0.2979 | -1.0995 | 0.3302 | 0.496455 | 1 | 9654 | tags=28%, list=1 |
| GO_STRESS | GO_STRESS_FIBER_A | 104 | -0.294 | -1.1 | 0.3302 | 0.49703 | 1 | 9937 | tags=29%, list=1 |
| GO_REGUL | GO_REGULATION_OI | 64 | -0.3824 | -1.1312 | 0.3306 | 0.483607 | 1 | 7870 | tags=36%, list=1 |
| GO_THYMU | GO_THYMUS_DEVEL | 46 | -0.3259 | -1.1097 | 0.3307 | 0.493745 | 1 | 8398 | tags=24%, list=1 |
| GO_SODIUI | GO_SODIUM_CHANN | 40 | -0.329 | -1.1078 | 0.3313 | 0.494729 | 1 | 10004 | tags=30%, list=1 |
| GO_SPANN | GO_SPANNING_COM | 19 | -0.3956 | -1.1016 | 0.3314 | 0.49613 | 1 | 13679 | tags=37%, list=2 |
| GO_HISTON | GO_HISTONE_H3_K9_ | 37 | -0.3923 | -1.1403 | 0.3314 | 0.480426 | 1 | 21001 | tags=59%, list=3 |
| GO_POSITI | GO_POSITIVE_REGUI | 110 | -0.3275 | -1.1144 | 0.332 | 0.490602 | 1 | 16983 | tags=37%, list=2 |
| GO_MIDDL | GO_MIDDLE_EAR_M | 20 | -0.3669 | -1.0888 | 0.332 | 0.502259 | 1 | 17220 | tags=50%, list=2 |
| GO_C2H2_Z | GO_C2H2_ZINC_FINC | 15 | -0.413 | -1.1172 | 0.332 | 0.489144 | 1 | 11652 | tags=40%, list=2 |
| GO_POSTSY | GO_POSTSYNAPTIC_ | 18 | -0.4279 | -1.1358 | 0.3321 | 0.482304 | 1 | 18541 | tags=44%, list=3 |
| GO_COLLA | GO_COLLAGEN_FIBR | 55 | -0.4471 | -1.178 | 0.3327 | 0.460061 | 1 | 16983 | tags=53%, list=2 |
| GO_REGUL | GO_REGULATION_OI | 23 | -0.3653 | -1.1058 | 0.3333 | 0.494089 | 1 | 8887 | tags=30%, list=1 |
| GO_LONG_ | GO_LONG_CHAIN_FA | 23 | -0.3588 | -1.1037 | 0.3333 | 0.494552 | 1 | 12285 | tags=35%, list=2 |
| GO_REGUL | GO_REGULATION_OI | 35 | -0.3266 | -1.1014 | 0.3333 | 0.496153 | 1 | 12629 | tags=37%, list=2 |
| GO_CELL_( | GO_CELL_GROWTH | 475 | -0.258 | -1.0961 | 0.3333 | 0.498464 | 1 | 13067 | tags=32%, list=2 |
| GO_REGUL | GO_REGULATION_OI | 35 | -0.3197 | -1.0847 | 0.3333 | 0.505227 | 1 | 13788 | tags=43%, list=2 |
| GO_ARGINI | GO_ARGININE_META | 20 | -0.3634 | -1.0752 | 0.3333 | 0.511789 | 1 | 3576 | tags=20%, list=6 |
| GO_CELL_( | GO_CELL_CORTEX | 285 | -0.2551 | -1.0647 | 0.334 | 0.519333 | 1 | 11780 | tags=30%, list=2 |
| GO_TRANS | GO_TRANSCRIPTION | 260 | -0.292 | -1.1066 | 0.334 | 0.493727 | 1 | 12828 | tags=34%, list=2 |
| GO_GLIOGI | GO_GLIOGENESIS | 303 | -0.2624 | -1.0723 | 0.334 | 0.514587 | 1 | 12629 | tags=29%, list=2 |
| GO_PHOSPI | GO_PHOSPHATIDYLI | 27 | -0.3378 | -1.0896 | 0.3346 | 0.502085 | 1 | 5751 | tags=26%, list=1 |
| GO_NEGAT | GO_NEGATIVE_REGU | 52 | -0.3614 | -1.1341 | 0.3346 | 0.481776 | 1 | 6879 | tags=29%, list=1 |
| GO_INSULI | GO_INSULIN_LIKE_G | 16 | -0.3823 | -1.0948 | 0.3353 | 0.498394 | 1 | 12950 | tags=50%, list=2 |
| GO_CCR4_I | GO_CCR4_NOT_COM | 15 | -0.4462 | -1.1173 | 0.3353 | 0.489895 | 1 | 7128 | tags=33%, list=1 |
| GO_CALCIU | GO_CALCIUM_ION_R | 63 | -0.3364 | -1.1086 | 0.3354 | 0.49481 | 1 | 11615 | tags=37%, list=2 |
| GO_CALCIU | GO_CALCIUM_CHAN | 45 | -0.3469 | -1.1016 | 0.3361 | 0.496378 | 1 | 6290 | tags=22%, list=1 |
| GO_REGUL | GO_REGULATION_OI | 314 | -0.2765 | -1.0948 | 0.3365 | 0.498639 | 1 | 10612 | tags=31%, list=1 |
| GO_PEPTID | GO_PEPTIDE_HORMO | 251 | -0.2515 | -1.0484 | 0.3367 | 0.526526 | 1 | 12629 | tags=31%, list=2 |
| GO_GLUTA | GO_GLUTAMATE_RE | 27 | -0.4351 | -1.1439 | 0.3368 | 0.480194 | 1 | 23732 | tags=70%, list=4 |
| GO_IONOTI | GO_IONOTROPIC_GL | 19 | -0.4439 | -1.1241 | 0.3369 | 0.487336 | 1 | 23732 | tags=74%, list=4 |
| GO_ASTRO | GO_ASTROCYTE_DIF | 82 | -0.299 | -1.079 | 0.3373 | 0.507403 | 1 | 8426 | tags=28%, list=1 |
| GO_PHOTO | GO_PHOTOTRANSDU | 58 | -0.2863 | -1.0658 | 0.3373 | 0.518999 | 1 | 18221 | tags=40%, list=3 |
| GO_MITOC | GO_MITOCHONDRIA | 39 | -0.3271 | -1.1 | 0.3384 | 0.496489 | 1 | 3119 | tags=28%, list=5 |
| GO_NUCLE | GO_NUCLEOSIDE_BI | 64 | -0.3165 | -1.1038 | 0.3387 | 0.49471 | 1 | 17293 | tags=41%, list=3 |
| GO_LYMPH | GO_LYMPH_VESSEL_ | 26 | -0.3627 | -1.0739 | 0.339 | 0.513167 | 1 | 14543 | tags=46%, list=2 |
| GO_RESPOI | GO_RESPONSE_TO_F | 89 | -0.2752 | -1.0667 | 0.3391 | 0.518729 | 1 | 11248 | tags=25%, list=1 |
| GO_NEGAT | GO_NEGATIVE_REGU | 18 | -0.3936 | -1.0942 | 0.3393 | 0.498773 | 1 | 10004 | tags=33%, list=1 |
| GO_NEGAT | GO_NEGATIVE_REGU | 47 | -0.3087 | -1.0741 | 0.3393 | 0.513014 | 1 | 15325 | tags=30%, list=2 |
| GO_POSITI | GO_POSITIVE_REGUI | 105 | -0.2916 | -1.0672 | 0.3397 | 0.518366 | 1 | 9641 | tags=28%, list=1 |

| | | | | | | | | | | |
|---|---|---|---|---|---|---|---|---|---|---|
| GO_STEROI | GO_STEROL_METAB | 164 | -0.265 | -1.0748 | 0.3404 | 0.512179 | 1 | 4065 | tags=14%, list=7 |
| GO_PROTO | GO_PROTON_TRANS | 108 | -0.2681 | -1.0641 | 0.3408 | 0.518619 | 1 | 12508 | tags=29%, list=2 |
| GO_LABYR | GO_LABYRINTHINE_ | 20 | -0.3639 | -1.0911 | 0.3414 | 0.501149 | 1 | 10185 | tags=40%, list=1 |
| GO_NEGAT | GO_NEGATIVE_REGU | 26 | -0.37 | -1.0895 | 0.3415 | 0.501905 | 1 | 10431 | tags=35%, list=1 |
| GO_ENDOD | GO_ENDODEOXYRIB | 17 | -0.407 | -1.108 | 0.3431 | 0.494521 | 1 | 10581 | tags=29%, list=1 |
| GO_PROTEI | GO_PROTEIN_O_LINI | 96 | -0.289 | -1.0645 | 0.3433 | 0.518943 | 1 | 6384 | tags=19%, list=1 |
| GO_CILIUM | GO_CILIUM_ORGANI | 367 | -0.3081 | -1.1084 | 0.3434 | 0.494753 | 1 | 13011 | tags=32%, list=2 |
| GO_NEGAT | GO_NEGATIVE_REGU | 94 | -0.2625 | -1.0697 | 0.3435 | 0.516767 | 1 | 12318 | tags=26%, list=2 |
| GO_ION_CI | GO_ION_CHANNEL_I | 130 | -0.2776 | -1.0714 | 0.3436 | 0.515744 | 1 | 15356 | tags=38%, list=2 |
| GO_ENDOD | GO_ENDODERMAL_C | 15 | -0.3949 | -1.0879 | 0.3437 | 0.502302 | 1 | 10888 | tags=47%, list=1 |
| GO_MYOSI | GO_MYOSIN_BINDIN | 71 | -0.2808 | -1.0632 | 0.3439 | 0.519223 | 1 | 9192 | tags=28%, list=1 |
| GO_ORGAN | GO_ORGANIC_HYDR | 48 | -0.2995 | -1.0608 | 0.3443 | 0.51844 | 1 | 7107 | tags=23%, list=1 |
| GO_HISTON | GO_HISTONE_H4_AC | 70 | -0.3618 | -1.1362 | 0.3444 | 0.482157 | 1 | 18074 | tags=49%, list=3 |
| GO_RAL_G | GO_RAL_GTPASE_BI | 15 | -0.4016 | -1.1066 | 0.345 | 0.493919 | 1 | 15888 | tags=47%, list=2 |
| GO_NEGAT | GO_NEGATIVE_REGU | 249 | -0.246 | -1.0544 | 0.3457 | 0.521801 | 1 | 13311 | tags=32%, list=2 |
| GO_ORGAN | GO_ORGANISM_EME | 28 | -0.3251 | -1.0859 | 0.3458 | 0.504283 | 1 | 7793 | tags=36%, list=1 |
| GO_RHYTH | GO_RHYTHMIC_BEH | 50 | -0.3027 | -1.0797 | 0.3459 | 0.507277 | 1 | 7968 | tags=24%, list=1 |
| GO_VESICL | GO_VESICLE_CARGC | 27 | -0.3799 | -1.111 | 0.3463 | 0.492784 | 1 | 15233 | tags=44%, list=2 |
| GO_HORMO | GO_HORMONE_RECI | 174 | -0.2733 | -1.0701 | 0.347 | 0.5169 | 1 | 8416 | tags=28%, list=1 |
| GO_AMINO | GO_AMINO_ACID_TI | 89 | -0.2948 | -1.066 | 0.3472 | 0.51911 | 1 | 9176 | tags=24%, list=1 |
| GO_OXIDO | GO_OXIDOREDUCTA | 43 | -0.3266 | -1.0791 | 0.3478 | 0.507614 | 1 | 13276 | tags=30%, list=2 |
| GO_P53_BI | GO_P53_BINDING | 64 | -0.3515 | -1.1104 | 0.349 | 0.492877 | 1 | 15621 | tags=42%, list=2 |
| GO_CELLU | GO_CELLULAR_RESI | 70 | -0.2879 | -1.0556 | 0.3504 | 0.521476 | 1 | 7789 | tags=27%, list=1 |
| GO_VENOU | GO_VENOUS_BLOOD | 15 | -0.4124 | -1.1131 | 0.3515 | 0.491562 | 1 | 1087 | tags=20%, list=2 |
| GO_REGUL | GO_REGULATION_OI | 88 | -0.3489 | -1.1168 | 0.3517 | 0.488852 | 1 | 9771 | tags=34%, list=1 |
| GO_DEVEL | GO_DEVELOPMENTA | 274 | -0.2621 | -1.0649 | 0.3518 | 0.519453 | 1 | 11099 | tags=27%, list=1 |
| GO_RRNA_ | GO_RRNA_TRANSCR | 28 | -0.4132 | -1.1423 | 0.3519 | 0.480401 | 1 | 13219 | tags=39%, list=2 |
| GO_PROTEI | GO_PROTEIN_TRANS | 67 | -0.3604 | -1.1127 | 0.3523 | 0.491001 | 1 | 12749 | tags=39%, list=2 |
| GO_ESTABI | GO_ESTABLISHMENT | 16 | -0.4084 | -1.103 | 0.3526 | 0.49476 | 1 | 2297 | tags=25%, list=4 |
| GO_METAN | GO_METANEPHRIC_I | 16 | -0.4186 | -1.0977 | 0.3534 | 0.498121 | 1 | 5634 | tags=38%, list=1 |
| GO_METAL | GO_METALLOAMINC | 21 | -0.3466 | -1.0705 | 0.3537 | 0.51672 | 1 | 3016 | tags=24%, list=5 |
| GO_EATIN | GO_EATING_BEHAVI | 33 | -0.3372 | -1.1049 | 0.354 | 0.494817 | 1 | 17571 | tags=42%, list=3 |
| GO_NEURO | GO_NEUROMUSCUL | 16 | -0.4055 | -1.0907 | 0.354 | 0.501313 | 1 | 7714 | tags=44%, list=1 |
| GO_PROTEI | GO_PROTEIN_DEMAI | 19 | -0.3835 | -1.0693 | 0.3543 | 0.517068 | 1 | 11143 | tags=42%, list=1 |
| GO_REGUL | GO_REGULATION_OI | 15 | -0.4145 | -1.1073 | 0.3544 | 0.493907 | 1 | 7372 | tags=40%, list=1 |
| GO_FATTY | GO_FATTY_ACID_DE | 99 | -0.2553 | -1.0572 | 0.3545 | 0.520568 | 1 | 12285 | tags=29%, list=2 |
| GO_POSITI | GO_POSITIVE_REGUI | 47 | -0.3333 | -1.0865 | 0.3555 | 0.50358 | 1 | 9318 | tags=30%, list=1 |
| GO_IRE1_M | GO_IRE1_MEDIATED | 65 | -0.3118 | -1.0815 | 0.3556 | 0.506913 | 1 | 7176 | tags=23%, list=1 |
| GO_TERTIA | GO_TERTIARY_ALCC | 20 | -0.3723 | -1.0704 | 0.3566 | 0.516623 | 1 | 22074 | tags=65%, list=3 |
| GO_NEGAT | GO_NEGATIVE_REGU | 186 | -0.2543 | -1.0504 | 0.3569 | 0.523544 | 1 | 6461 | tags=22%, list=1 |
| GO_GANGL | GO_GANGLION_DEV | 16 | -0.4716 | -1.1245 | 0.3573 | 0.487797 | 1 | 7998 | tags=31%, list=1 |
| GO_NEGAT | GO_NEGATIVE_REGU | 49 | -0.3132 | -1.0763 | 0.3573 | 0.510573 | 1 | 12059 | tags=37%, list=2 |
| GO_BONE_ | GO_BONE_MATURAT | 20 | -0.3736 | -1.0842 | 0.358 | 0.505225 | 1 | 17571 | tags=50%, list=3 |
| GO_INTEGI | GO_INTEGRAL_COMI | 34 | -0.362 | -1.083 | 0.358 | 0.50634 | 1 | 14389 | tags=44%, list=2 |
| GO_DNA_B | GO_DNA_BINDING_T | 333 | -0.2746 | -1.0811 | 0.358 | 0.50726 | 1 | 9390 | tags=26%, list=1 |
| GO_R_SMA | GO_R_SMAD_BINDIN | 23 | -0.3577 | -1.0821 | 0.3583 | 0.506865 | 1 | 15946 | tags=43%, list=2 |
| GO_NEGAT | GO_NEGATIVE_REGU | 32 | -0.3844 | -1.0933 | 0.3587 | 0.499824 | 1 | 8399 | tags=25%, list=1 |
| GO_REGUL | GO_REGULATION_OI | 150 | -0.2724 | -1.0633 | 0.3591 | 0.519267 | 1 | 9901 | tags=28%, list=1 |
| GO_POSITI | GO_POSITIVE_REGUI | 40 | -0.3607 | -1.1003 | 0.3594 | 0.4974 | 1 | 15109 | tags=38%, list=2 |
| GO_PEPTID | GO_PEPTIDE_LYSINI | 56 | -0.3813 | -1.1062 | 0.3595 | 0.493752 | 1 | 12756 | tags=34%, list=2 |
| GO_NEGAT | GO_NEGATIVE_REGU | 387 | -0.2782 | -1.0894 | 0.3596 | 0.501718 | 1 | 11099 | tags=29%, list=1 |
| GO_VASCU | GO_VASCULAR_END | 48 | -0.3192 | -1.0641 | 0.3597 | 0.518845 | 1 | 10523 | tags=35%, list=1 |
| GO_PURINI | GO_PURINE_CONTAI | 430 | -0.2441 | -1.0561 | 0.36 | 0.521089 | 1 | 9901 | tags=25%, list=1 |
| GO_POSITI | GO_POSITIVE_REGUI | 37 | -0.3173 | -1.0765 | 0.3602 | 0.510812 | 1 | 10660 | tags=32%, list=1 |
| GO_INSULI | GO_INSULIN_SECRE | 207 | -0.2506 | -1.0425 | 0.3602 | 0.529397 | 1 | 12629 | tags=32%, list=2 |
| GO_ORGAN | GO_ORGANIC_ACID_ | 219 | -0.2534 | -1.0517 | 0.3603 | 0.523573 | 1 | 12405 | tags=30%, list=2 |
| GO_REGUL | GO_REGULATION_OI | 183 | -0.2751 | -1.0573 | 0.3608 | 0.52126 | 1 | 10614 | tags=28%, list=1 |
| GO_DIOXY | GO_DIOXYGENASE_ | 88 | -0.2782 | -1.0643 | 0.3609 | 0.518778 | 1 | 12725 | tags=31%, list=2 |

| | | | | | | | | | | |
|---|---|---|---|---|---|---|---|---|---|---|
| GO_REGUL | GO_REGULATION_OI | 20 | -0.3685 | -1.0877 | 0.3614 | 0.501973 | 1 | 8997 | tags=30%, list=1 |
| GO_POSITI | GO_POSITIVE_REGUI | 20 | -0.393 | -1.0811 | 0.3615 | 0.506753 | 1 | 2889 | tags=25%, list=5 |
| GO_SPHINC | GO_SPHINGOLIPID_E | 24 | -0.3546 | -1.0857 | 0.3618 | 0.504368 | 1 | 2672 | tags=25%, list=5 |
| GO_POSITI | GO_POSITIVE_REGUI | 99 | -0.2814 | -1.0573 | 0.3619 | 0.521046 | 1 | 13137 | tags=36%, list=2 |
| GO_INTERM | GO_INTERMEDIATE_ | 50 | -0.3164 | -1.0553 | 0.362 | 0.521067 | 1 | 7970 | tags=26%, list=1 |
| GO_ENDOP | GO_ENDOPLASMIC_I | 17 | -0.4143 | -1.0993 | 0.3624 | 0.496117 | 1 | 11595 | tags=29%, list=2 |
| GO_PRE_M | GO_PRE_MRNA_BINI | 57 | -0.3734 | -1.101 | 0.3626 | 0.496545 | 1 | 12984 | tags=42%, list=2 |
| GO_CATIOI | GO_CATION_CATION | 26 | -0.3209 | -1.0461 | 0.3627 | 0.528001 | 1 | 15374 | tags=42%, list=2 |
| GO_GLYCO | GO_GLYCOSYLATIOI | 253 | -0.258 | -1.0456 | 0.3631 | 0.528282 | 1 | 12832 | tags=28%, list=2 |
| GO_INTERM | GO_INTERMEDIATE_ | 15 | -0.3795 | -1.0804 | 0.3634 | 0.507364 | 1 | 13136 | tags=47%, list=2 |
| GO_CAMP_ | GO_CAMP_BINDING | 23 | -0.3541 | -1.0623 | 0.3636 | 0.518777 | 1 | 20068 | tags=48%, list=3 |
| GO_REGUL | GO_REGULATION_OI | 40 | -0.3893 | -1.0949 | 0.3638 | 0.49876 | 1 | 10276 | tags=33%, list=1 |
| GO_LYSOPI | GO_LYSOPHOSPHOL | 18 | -0.3654 | -1.0756 | 0.3642 | 0.511334 | 1 | 646 | tags=17%, list=1 |
| GO_PEPTID | GO_PEPTIDE_N_ACE | 63 | -0.3716 | -1.1125 | 0.3642 | 0.491046 | 1 | 12756 | tags=35%, list=2 |
| GO_PRE_M | GO_PRE_MRNA_5_SP | 18 | -0.5452 | -1.1251 | 0.3642 | 0.487179 | 1 | 12984 | tags=72%, list=2 |
| GO_BLOOD | GO_BLOOD_VESSEL_ | 55 | -0.3008 | -1.045 | 0.3649 | 0.528807 | 1 | 15325 | tags=31%, list=2 |
| GO_NEGAT | GO_NEGATIVE_REGU | 30 | -0.3218 | -1.07 | 0.3655 | 0.516686 | 1 | 7971 | tags=27%, list=1 |
| GO_GOLGI_ | GO_GOLGI_CISTERN | 91 | -0.2709 | -1.0651 | 0.3656 | 0.519791 | 1 | 8047 | tags=20%, list=1 |
| GO_LIVER_ | GO_LIVER_REGENER | 32 | -0.3272 | -1.0632 | 0.3658 | 0.518999 | 1 | 3652 | tags=19%, list=6 |
| GO_CENTR | GO_CENTRAL_NERV | 26 | -0.3804 | -1.1 | 0.3661 | 0.496737 | 1 | 11108 | tags=38%, list=1 |
| GO_POSITI | GO_POSITIVE_REGUI | 40 | -0.3029 | -1.0608 | 0.3663 | 0.518919 | 1 | 7119 | tags=28%, list=1 |
| GO_POSITI | GO_POSITIVE_REGUI | 59 | -0.299 | -1.0511 | 0.3665 | 0.52435 | 1 | 9470 | tags=29%, list=1 |
| GO_REGUL | GO_REGULATION_OI | 184 | -0.3181 | -1.1051 | 0.3667 | 0.494711 | 1 | 10581 | tags=32%, list=1 |
| GO_NLS_BI | GO_NLS_BEARING_P | 20 | -0.4246 | -1.1197 | 0.3667 | 0.488997 | 1 | 14625 | tags=45%, list=2 |
| GO_ATPASI | GO_ATPASE_COMPLI | 76 | -0.3661 | -1.1045 | 0.3673 | 0.49462 | 1 | 8390 | tags=28%, list=1 |
| GO_ADREN | GO_ADRENERGIC_RI | 31 | -0.3525 | -1.0816 | 0.368 | 0.50709 | 1 | 19410 | tags=45%, list=3 |
| GO_REGUL | GO_REGULATION_OI | 35 | -0.348 | -1.0565 | 0.3682 | 0.521204 | 1 | 9470 | tags=29%, list=1 |
| GO_SERINE | GO_SERINE_FAMILY_ | 17 | -0.426 | -1.069 | 0.3685 | 0.516781 | 1 | 6471 | tags=29%, list=1 |
| GO_ACTIN_ | GO_ACTIN_BINDING | 425 | -0.2603 | -1.0315 | 0.3685 | 0.537633 | 1 | 11800 | tags=31%, list=2 |
| GO_PROTEI | GO_PROTEIN_LOCAL | 330 | -0.2507 | -1.0612 | 0.3696 | 0.519709 | 1 | 9188 | tags=24%, list=1 |
| GO_PIRNA_ | GO_PIRNA_METABOI | 18 | -0.3922 | -1.0591 | 0.3697 | 0.519561 | 1 | 16537 | tags=33%, list=2 |
| GO_REGUL | GO_REGULATION_OI | 126 | -0.2678 | -1.0554 | 0.37 | 0.521255 | 1 | 10299 | tags=26%, list=1 |
| GO_REGUL | GO_REGULATION_OI | 25 | -0.3494 | -1.0575 | 0.3702 | 0.521138 | 1 | 9127 | tags=36%, list=1 |
| GO_FORMA | GO_FORMATION_OF_ | 15 | -0.443 | -1.0929 | 0.3704 | 0.499824 | 1 | 11005 | tags=47%, list=1 |
| GO_NEGAT | GO_NEGATIVE_REGU | 20 | -0.3431 | -1.0523 | 0.3715 | 0.523078 | 1 | 9806 | tags=35%, list=1 |
| GO_GLIAL_ | GO_GLIAL_CELL_DE | 120 | -0.2604 | -1.0395 | 0.3716 | 0.532011 | 1 | 12426 | tags=30%, list=2 |
| GO_GOLGI_ | GO_GOLGI_TO_ENDO | 15 | -0.4194 | -1.0882 | 0.3719 | 0.502029 | 1 | 15530 | tags=53%, list=2 |
| GO_ORGAN | GO_ORGANIC_ANION | 231 | -0.2492 | -1.043 | 0.3723 | 0.52916 | 1 | 9711 | tags=22%, list=1 |
| GO_MICRO | GO_MICROVILLUS_A | 17 | -0.3772 | -1.0638 | 0.373 | 0.518853 | 1 | 8740 | tags=35%, list=1 |
| GO_CYTOP | GO_CYTOPLASMIC_N | 62 | -0.3266 | -1.0876 | 0.3733 | 0.501868 | 1 | 4421 | tags=21%, list=8 |
| GO_VESICI | GO_VESICLE_TETHE | 66 | -0.3078 | -1.0562 | 0.374 | 0.521224 | 1 | 5773 | tags=24%, list=1 |
| GO_HEPAR | GO_HEPARAN_SULF | 26 | -0.3565 | -1.0738 | 0.3748 | 0.513116 | 1 | 17308 | tags=50%, list=3 |
| GO_POSITI | GO_POSITIVE_REGUI | 37 | -0.3399 | -1.078 | 0.375 | 0.508885 | 1 | 11248 | tags=32%, list=1 |
| GO_REGUL | GO_REGULATION_OI | 16 | -0.4127 | -1.0677 | 0.375 | 0.517722 | 1 | 8882 | tags=50%, list=1 |
| GO_REGUL | GO_REGULATION_OI | 46 | -0.3092 | -1.0593 | 0.3752 | 0.519829 | 1 | 3829 | tags=20%, list=7 |
| GO_COLUM | GO_COLUMNAR_CUI | 116 | -0.2842 | -1.0506 | 0.3752 | 0.524082 | 1 | 12934 | tags=32%, list=2 |
| GO_REGUL | GO_REGULATION_OI | 48 | -0.3472 | -1.091 | 0.376 | 0.501158 | 1 | 6594 | tags=27%, list=1 |
| GO_PEPTID | GO_PEPTIDYL_GLUT | 30 | -0.3453 | -1.0609 | 0.377 | 0.51916 | 1 | 13631 | tags=37%, list=2 |
| GO_POSITI | GO_POSITIVE_REGUI | 76 | -0.2857 | -1.0446 | 0.3776 | 0.528636 | 1 | 10045 | tags=29%, list=1 |
| GO_SODIUI | GO_SODIUM_ION_IM | 19 | -0.3632 | -1.0504 | 0.378 | 0.523792 | 1 | 4750 | tags=21%, list=8 |
| GO_REGUL | GO_REGULATION_OI | 84 | -0.2883 | -1.0475 | 0.3786 | 0.52669 | 1 | 11210 | tags=24%, list=1 |
| GO_REGUL | GO_REGULATION_OI | 85 | -0.296 | -1.0601 | 0.3788 | 0.519342 | 1 | 7103 | tags=20%, list=1 |
| GO_STRESS | GO_STRESS_GRANUI | 23 | -0.4078 | -1.1136 | 0.3789 | 0.491371 | 1 | 14298 | tags=43%, list=2 |
| GO_REGUL | GO_REGULATION_OI | 22 | -0.4122 | -1.0986 | 0.3789 | 0.497205 | 1 | 19156 | tags=59%, list=3 |
| GO_REGUL | GO_REGULATION_OI | 20 | -0.3675 | -1.0395 | 0.379 | 0.532254 | 1 | 8022 | tags=35%, list=1 |
| GO_DYNEII | GO_DYNEIN_COMPL | 20 | -0.411 | -1.0901 | 0.3794 | 0.50177 | 1 | 9173 | tags=30%, list=1 |
| GO_NUCLE | GO_NUCLEOTIDE_DI | 17 | -0.3737 | -1.057 | 0.3796 | 0.520669 | 1 | 2118 | tags=18%, list=4 |
| GO_CYTOP | GO_CYTOPLASMIC_I | 21 | -0.3832 | -1.0692 | 0.3797 | 0.51695 | 1 | 7336 | tags=38%, list=1 |

| | | | | | | | | | |
|---|---|---|---|---|---|---|---|---|---|
| GO_RESPO! | GO_RESPONSE_TO_F | 161 | -0.286 | -1.0541 | 0.38 | 0.521671 | 1 | 10263 | tags=29%, list=1 |
| GO_CELLU | GO_CELLULAR_RESF | 23 | -0.3582 | -1.0692 | 0.3801 | 0.516711 | 1 | 4065 | tags=22%, list=7 |
| GO_STRUC | GO_STRUCTURAL_C | 101 | -0.2801 | -1.0426 | 0.3802 | 0.529548 | 1 | 12035 | tags=35%, list=2 |
| GO_POSITI | GO_POSITIVE_REGU | 96 | -0.2716 | -1.0449 | 0.3803 | 0.528576 | 1 | 8956 | tags=26%, list=1 |
| GO_PSEUD | GO_PSEUDOPODIUM | 18 | -0.3606 | -1.0572 | 0.3807 | 0.520788 | 1 | 14188 | tags=50%, list=2 |
| GO_EPIDEF | GO_EPIDERMAL_GR | 33 | -0.3388 | -1.0669 | 0.381 | 0.518582 | 1 | 2867 | tags=18%, list=5 |
| GO_GLUTA | GO_GLUTAMINE_FAI | 16 | -0.366 | -1.0588 | 0.3823 | 0.51985 | 1 | 5161 | tags=25%, list=9 |
| GO_ACTIN_ | GO_ACTIN_CYTOSKE | 486 | -0.2491 | -1.028 | 0.3825 | 0.540998 | 1 | 8907 | tags=26%, list=1 |
| GO_REGUL | GO_REGULATION_OI | 36 | -0.3224 | -1.0584 | 0.3826 | 0.520221 | 1 | 3829 | tags=19%, list=7 |
| GO_TRANS | GO_TRANSLATION_F | 105 | -0.3076 | -1.0545 | 0.383 | 0.521836 | 1 | 5882 | tags=17%, list=1 |
| GO_CONTR | GO_CONTRACTILE_F | 236 | -0.3046 | -1.084 | 0.3832 | 0.505303 | 1 | 14372 | tags=36%, list=2 |
| GO_INTRA | GO_INTRACELLULAI | 43 | -0.3202 | -1.0612 | 0.3839 | 0.519438 | 1 | 11233 | tags=30%, list=1 |
| GO_GLAND | GO_GLAND_DEVELO | 441 | -0.2324 | -1.0372 | 0.3839 | 0.533682 | 1 | 8692 | tags=22%, list=1 |
| GO_GROW | GO_GROWTH_PLATE | 19 | -0.356 | -1.0414 | 0.3846 | 0.530745 | 1 | 15844 | tags=42%, list=2 |
| GO_REGUL | GO_REGULATION_OI | 65 | -0.3191 | -1.0531 | 0.3849 | 0.522539 | 1 | 10511 | tags=34%, list=1 |
| GO_SMAD_ | GO_SMAD_BINDING | 79 | -0.3043 | -1.0344 | 0.3853 | 0.536391 | 1 | 15946 | tags=41%, list=2 |
| GO_NEGAT | GO_NEGATIVE_REGU | 194 | -0.2646 | -1.0308 | 0.3854 | 0.538069 | 1 | 11813 | tags=28%, list=2 |
| GO_NUCLE | GO_NUCLEOSOMAL_ | 24 | -0.4466 | -1.1263 | 0.3855 | 0.486815 | 1 | 4301 | tags=29%, list=7 |
| GO_CELL_I | GO_CELL_MATRIX_A | 227 | -0.2784 | -1.048 | 0.3855 | 0.526138 | 1 | 5834 | tags=22%, list=1 |
| GO_DIACYI | GO_DIACYLGLYCER | 24 | -0.3457 | -1.0522 | 0.3855 | 0.522959 | 1 | 10468 | tags=33%, list=1 |
| GO_POSITI | GO_POSITIVE_REGU | 37 | -0.3711 | -1.0735 | 0.386 | 0.513253 | 1 | 14474 | tags=43%, list=2 |
| GO_POSITI | GO_POSITIVE_REGU | 40 | -0.3211 | -1.0398 | 0.3868 | 0.53201 | 1 | 5634 | tags=23%, list=1 |
| GO_REGUL | GO_REGULATION_OI | 77 | -0.2867 | -1.0339 | 0.3868 | 0.536725 | 1 | 7567 | tags=26%, list=1 |
| GO_PHOSPI | GO_PHOSPHATIDYLS | 20 | -0.3528 | -1.0483 | 0.3869 | 0.526414 | 1 | 8929 | tags=35%, list=1 |
| GO_REGUL | GO_REGULATION_OI | 74 | -0.2981 | -1.0665 | 0.387 | 0.518808 | 1 | 6429 | tags=22%, list=1 |
| GO_L_ALPI | GO_L_ALPHA_AMIN | 59 | -0.3101 | -1.0388 | 0.3871 | 0.532668 | 1 | 9176 | tags=25%, list=1 |
| GO_CLATH | GO_CLATHRIN_DEPE | 45 | -0.3222 | -1.0623 | 0.3872 | 0.519236 | 1 | 11328 | tags=33%, list=1 |
| GO_SMOOT | GO_SMOOTH_MUSCI | 90 | -0.2925 | -1.0532 | 0.3874 | 0.52272 | 1 | 9996 | tags=27%, list=1 |
| GO_REGUL | GO_REGULATION_OI | 16 | -0.3872 | -1.0689 | 0.3876 | 0.516617 | 1 | 18948 | tags=50%, list=3 |
| GO_PHENO | GO_PHENOL_CONTA | 45 | -0.2927 | -1.0304 | 0.3881 | 0.538279 | 1 | 7111 | tags=22%, list=1 |
| GO_NEGAT | GO_NEGATIVE_REGU | 131 | -0.3421 | -1.0847 | 0.3886 | 0.505474 | 1 | 6879 | tags=27%, list=1 |
| GO_REGUL | GO_REGULATION_OI | 86 | -0.2994 | -1.0593 | 0.3894 | 0.520088 | 1 | 14094 | tags=41%, list=2 |
| GO_PROTEI | GO_PROTEIN_SUMO | 80 | -0.3365 | -1.0714 | 0.39 | 0.515531 | 1 | 10731 | tags=33%, list=1 |
| GO_RESPO! | GO_RESPONSE_TO_A | 28 | -0.3426 | -1.0549 | 0.3902 | 0.521561 | 1 | 14538 | tags=50%, list=2 |
| GO_CELLU | GO_CELLULAR_RESF | 72 | -0.3046 | -1.0459 | 0.3902 | 0.528003 | 1 | 9450 | tags=29%, list=1 |
| GO_ALTER | GO_ALTERNATIVE_M | 87 | -0.3563 | -1.0592 | 0.3903 | 0.519741 | 1 | 12613 | tags=36%, list=2 |
| GO_GLYCO | GO_GLYCOLIPID_BII | 25 | -0.3533 | -1.0437 | 0.3904 | 0.529061 | 1 | 2672 | tags=20%, list=5 |
| GO_NEGAT | GO_NEGATIVE_REGU | 41 | -0.3085 | -1.0509 | 0.3912 | 0.524338 | 1 | 6700 | tags=27%, list=1 |
| GO_PREPUI | GO_PREPULSE_INHII | 15 | -0.4421 | -1.0811 | 0.3913 | 0.506494 | 1 | 29412 | tags=93%, list=5 |
| GO_PCG_PI | GO_PCG_PROTEIN_C | 46 | -0.3663 | -1.0611 | 0.3916 | 0.519374 | 1 | 7569 | tags=37%, list=1 |
| GO_SAGA_ | GO_SAGA_TYPE_CON | 27 | -0.3855 | -1.0794 | 0.3919 | 0.507536 | 1 | 15421 | tags=44%, list=2 |
| GO_RESPO! | GO_RESPONSE_TO_T | 225 | -0.2587 | -1.044 | 0.3921 | 0.529027 | 1 | 10263 | tags=26%, list=1 |
| GO_MITOC | GO_MITOCHONDRIA | 24 | -0.3346 | -1.0576 | 0.3922 | 0.521361 | 1 | 3579 | tags=17%, list=6 |
| GO_ADENY | GO_ADENYLATE_CY | 86 | -0.3022 | -1.0507 | 0.3924 | 0.524108 | 1 | 12323 | tags=33%, list=2 |
| GO_REGUL | GO_REGULATION_OI | 63 | -0.2787 | -1.021 | 0.3928 | 0.545753 | 1 | 17921 | tags=40%, list=3 |
| GO_PROTEI | GO_PROTEIN_CONTA | 283 | -0.3075 | -1.0623 | 0.393 | 0.519017 | 1 | 9869 | tags=28%, list=1 |
| GO_REGUL | GO_REGULATION_OI | 25 | -0.3359 | -1.0437 | 0.3933 | 0.529295 | 1 | 6429 | tags=24%, list=1 |
| GO_PROTEI | GO_PROTEIN_DEGLY | 26 | -0.3512 | -1.0524 | 0.3934 | 0.523166 | 1 | 11143 | tags=38%, list=1 |
| GO_POSITI | GO_POSITIVE_REGU | 52 | -0.2863 | -1.0413 | 0.3934 | 0.530643 | 1 | 12756 | tags=29%, list=2 |
| GO_DEMET | GO_DEMETHYLATIO | 69 | -0.3155 | -1.0563 | 0.3941 | 0.521312 | 1 | 14019 | tags=39%, list=2 |
| GO_SPINDI | GO_SPINDLE_LOCAL | 49 | -0.3683 | -1.0608 | 0.3941 | 0.518722 | 1 | 10477 | tags=33%, list=1 |
| GO_EMBRY | GO_EMBRYONIC_SK | 97 | -0.2916 | -1.0505 | 0.3944 | 0.524025 | 1 | 9208 | tags=29%, list=1 |
| GO_MANN | GO_MANNOSYLATIC | 33 | -0.3468 | -1.0817 | 0.3948 | 0.507256 | 1 | 22092 | tags=58%, list=3 |
| GO_APICAI | GO_APICAL_PLASMA | 351 | -0.2427 | -1.0268 | 0.3949 | 0.541351 | 1 | 9810 | tags=23%, list=1 |
| GO_CILIAR | GO_CILIARY_BASAL | 140 | -0.3152 | -1.0682 | 0.3956 | 0.517195 | 1 | 18128 | tags=41%, list=3 |
| GO_POSITI | GO_POSITIVE_REGU | 72 | -0.2675 | -1.0202 | 0.3957 | 0.545098 | 1 | 8426 | tags=22%, list=1 |
| GO_INOSIT | GO_INOSITOL_LIPID | 194 | -0.2599 | -1.0274 | 0.3958 | 0.541138 | 1 | 7815 | tags=23%, list=1 |
| GO_P_BOD | GO_P_BODY_ASSEMI | 19 | -0.3943 | -1.0661 | 0.3968 | 0.519166 | 1 | 13484 | tags=53%, list=2 |

| | | | | | | | | | |
|---|---|---|---|---|---|---|---|---|---|
| GO_PROTEI | GO_PROTEIN_LOCAL | 29 | -0.388 | -1.0699 | 0.3976 | 0.516717 | 1 | 9146 | tags=34%, list=1 |
| GO_POSITI | GO_POSITIVE_REGUI | 25 | -0.3375 | -1.0486 | 0.398 | 0.526572 | 1 | 5232 | tags=20%, list=9 |
| GO_REGUL | GO_REGULATION_OI | 53 | -0.3176 | -1.0645 | 0.3981 | 0.518779 | 1 | 10083 | tags=34%, list=1 |
| GO_DNA_D | GO_DNA_DEPENDEN | 105 | -0.3751 | -1.0825 | 0.3984 | 0.506791 | 1 | 9177 | tags=29%, list=1 |
| GO_PROTEI | GO_PROTEIN_ACETY | 91 | -0.3432 | -1.0775 | 0.3985 | 0.509424 | 1 | 15855 | tags=42%, list=2 |
| GO_AMMO | GO_AMMONIUM_ION | 25 | -0.3478 | -1.0431 | 0.3988 | 0.529223 | 1 | 22025 | tags=52%, list=3 |
| GO_ACTIN_ | GO_ACTIN_FILAMEN | 154 | -0.2639 | -1.0273 | 0.3989 | 0.541099 | 1 | 9937 | tags=28%, list=1 |
| GO_PEPTID | GO_PEPTIDYL_LYSIN | 170 | -0.3058 | -1.0542 | 0.3992 | 0.521832 | 1 | 12883 | tags=32%, list=2 |
| GO_GLYCO | GO_GLYCOPROTEIN_ | 398 | -0.24 | -1.0105 | 0.3996 | 0.55105 | 1 | 13066 | tags=28%, list=2 |
| GO_POSITI | GO_POSITIVE_REGUI | 23 | -0.3531 | -1.0646 | 0.4 | 0.519163 | 1 | 13631 | tags=48%, list=2 |
| GO_PROST | GO_PROSTATE_GLAN | 28 | -0.3302 | -1.031 | 0.4 | 0.538268 | 1 | 7643 | tags=29%, list=1 |
| GO_CELLU | GO_CELLULAR_CARI | 85 | -0.2653 | -1.0237 | 0.4008 | 0.545654 | 1 | 20106 | tags=46%, list=3 |
| GO_PROTEI | GO_PROTEIN_ACETY | 200 | -0.2981 | -1.063 | 0.4012 | 0.518738 | 1 | 12883 | tags=33%, list=2 |
| GO_REGUL | GO_REGULATION_OI | 22 | -0.3653 | -1.0446 | 0.4012 | 0.528847 | 1 | 19084 | tags=55%, list=3 |
| GO_REGUL | GO_REGULATION_OI | 493 | -0.249 | -1.047 | 0.4015 | 0.527171 | 1 | 8481 | tags=22%, list=1 |
| GO_HISTON | GO_HISTONE_H2A_A | 17 | -0.4289 | -1.0828 | 0.4016 | 0.506522 | 1 | 23608 | tags=71%, list=4 |
| GO_DECIDU | GO_DECIDUALIZATI | 25 | -0.3236 | -1.0307 | 0.4016 | 0.537962 | 1 | 12819 | tags=40%, list=2 |
| GO_ACTIN_ | GO_ACTIN_FILAMEN | 53 | -0.3037 | -1.0261 | 0.4019 | 0.54188 | 1 | 12556 | tags=32%, list=2 |
| GO_NEGAT | GO_NEGATIVE_REGU | 304 | -0.2552 | -1.0386 | 0.4019 | 0.53275 | 1 | 7095 | tags=20%, list=1 |
| GO_TRANS | GO_TRANSFERASE_A | 202 | -0.283 | -1.0336 | 0.4031 | 0.53645 | 1 | 16653 | tags=37%, list=2 |
| GO_SUBST | GO_SUBSTANTIA_NI | 40 | -0.3218 | -1.0371 | 0.4031 | 0.533682 | 1 | 12976 | tags=38%, list=2 |
| GO_DICARI | GO_DICARBOXYLIC_ | 39 | -0.3076 | -1.0258 | 0.4035 | 0.542162 | 1 | 9176 | tags=28%, list=1 |
| GO_INTRA | GO_INTRACILIARY_T | 27 | -0.4099 | -1.0916 | 0.4035 | 0.501161 | 1 | 8248 | tags=30%, list=1 |
| GO_VESICI | GO_VESICLE_LOCAL | 222 | -0.2741 | -1.0361 | 0.4036 | 0.535064 | 1 | 6170 | tags=21%, list=1 |
| GO_LYMPH | GO_LYMPH_VESSEL_ | 20 | -0.3766 | -1.0398 | 0.4038 | 0.532278 | 1 | 13596 | tags=50%, list=2 |
| GO_METHY | GO_METHYLATED_H | 68 | -0.3499 | -1.0434 | 0.4039 | 0.52922 | 1 | 16569 | tags=43%, list=2 |
| GO_REGUL | GO_REGULATION_OI | 416 | -0.231 | -1.0174 | 0.4042 | 0.546938 | 1 | 9049 | tags=22%, list=1 |
| GO_MEIOTI | GO_MEIOTIC_CELL_ | 236 | -0.3008 | -1.0629 | 0.4043 | 0.518633 | 1 | 17789 | tags=39%, list=3 |
| GO_MEMBI | GO_MEMBRANE_REI | 29 | -0.327 | -1.022 | 0.4043 | 0.546633 | 1 | 3440 | tags=21%, list=6 |
| GO_POSITI | GO_POSITIVE_REGUI | 51 | -0.2952 | -1.0441 | 0.4054 | 0.529062 | 1 | 9222 | tags=29%, list=1 |
| GO_REGUL | GO_REGULATION_OI | 27 | -0.3713 | -1.045 | 0.4062 | 0.529015 | 1 | 15487 | tags=44%, list=2 |
| GO_CELL_S | GO_CELL_SUBSTRAT | 102 | -0.316 | -1.0611 | 0.4071 | 0.519107 | 1 | 8218 | tags=29%, list=1 |
| GO_POSITI | GO_POSITIVE_REGUI | 31 | -0.3128 | -1.0214 | 0.4079 | 0.54653 | 1 | 13311 | tags=32%, list=2 |
| GO_NEGAT | GO_NEGATIVE_REGU | 58 | -0.2685 | -1.0205 | 0.4082 | 0.545118 | 1 | 11823 | tags=24%, list=2 |
| GO_POSTS | GO_POSTSYNAPTIC_ | 21 | -0.3394 | -1.02 | 0.409 | 0.545145 | 1 | 11765 | tags=33%, list=2 |
| GO_PEPTID | GO_PEPTIDYL_LYSIN | 382 | -0.2926 | -1.0554 | 0.4093 | 0.521489 | 1 | 13852 | tags=34%, list=2 |
| GO_EYE_PI | GO_EYE_PHOTORECI | 46 | -0.2852 | -1.0128 | 0.4097 | 0.54953 | 1 | 17875 | tags=43%, list=3 |
| GO_MRNA_ | GO_MRNA_3_UTR_BI | 115 | -0.2926 | -1.0316 | 0.41 | 0.537879 | 1 | 16716 | tags=33%, list=2 |
| GO_REGUL | GO_REGULATION_OI | 28 | -0.361 | -1.0294 | 0.41 | 0.539391 | 1 | 9858 | tags=36%, list=1 |
| GO_NEGAT | GO_NEGATIVE_REGU | 22 | -0.3323 | -1.021 | 0.4102 | 0.545484 | 1 | 6746 | tags=18%, list=1 |
| GO_NEGAT | GO_NEGATIVE_REGU | 15 | -0.3835 | -1.0525 | 0.4103 | 0.523279 | 1 | 8740 | tags=27%, list=1 |
| GO_POSITI | GO_POSITIVE_REGUI | 30 | -0.3264 | -1.0296 | 0.4113 | 0.539235 | 1 | 12130 | tags=30%, list=2 |
| GO_POSITI | GO_POSITIVE_REGUI | 16 | -0.3968 | -1.0269 | 0.4115 | 0.54133 | 1 | 17806 | tags=56%, list=3 |
| GO_SALIVA | GO_SALIVARY_GLAN | 39 | -0.2999 | -1.0115 | 0.4132 | 0.550936 | 1 | 19965 | tags=46%, list=3 |
| GO_CELLU | GO_CELLULAR_RESI | 120 | -0.302 | -1.0222 | 0.4136 | 0.546428 | 1 | 10263 | tags=32%, list=1 |
| GO_RNA_SI | GO_RNA_SPLICING_ | 15 | -0.4221 | -1.0596 | 0.4138 | 0.519785 | 1 | 1292 | tags=13%, list=2 |
| GO_NEGAT | GO_NEGATIVE_REGU | 39 | -0.298 | -1.0278 | 0.4139 | 0.540627 | 1 | 8882 | tags=28%, list=1 |
| GO_POSITI | GO_POSITIVE_REGUI | 88 | -0.2939 | -1.0224 | 0.4144 | 0.546456 | 1 | 9573 | tags=31%, list=1 |
| GO_REGUL | GO_REGULATION_OI | 23 | -0.3948 | -1.0382 | 0.4145 | 0.532734 | 1 | 14054 | tags=43%, list=2 |
| GO_REGUL | GO_REGULATION_OI | 28 | -0.3566 | -1.0326 | 0.4148 | 0.536717 | 1 | 12474 | tags=39%, list=2 |
| GO_REGUL | GO_REGULATION_OI | 123 | -0.2764 | -1.0179 | 0.415 | 0.546242 | 1 | 4258 | tags=19%, list=7 |
| GO_ADIPOS | GO_ADIPOSE_TISSUI | 41 | -0.3074 | -1.0385 | 0.4154 | 0.532625 | 1 | 13607 | tags=37%, list=2 |
| GO_RETINC | GO_RETINOIC_ACID_ | 20 | -0.3438 | -1.0193 | 0.4155 | 0.54587 | 1 | 11709 | tags=30%, list=2 |
| GO_DETEC | GO_DETECTION_OF_ | 28 | -0.358 | -1.0168 | 0.4157 | 0.546974 | 1 | 24987 | tags=57%, list=4 |
| GO_RIBONI | GO_RIBONUCLEOPR | 226 | -0.292 | -1.032 | 0.4159 | 0.537455 | 1 | 14074 | tags=33%, list=2 |
| GO_DETEC | GO_DETECTION_OF_ | 19 | -0.3383 | -1.0287 | 0.4159 | 0.539921 | 1 | 19521 | tags=42%, list=3 |
| GO_RETRO | GO_RETROGRADE_V | 85 | -0.3088 | -1.038 | 0.4162 | 0.532784 | 1 | 15233 | tags=36%, list=2 |
| GO_RESPOI | GO_RESPONSE_TO_X | 31 | -0.3187 | -1.0079 | 0.4165 | 0.553074 | 1 | 9146 | tags=29%, list=1 |

| | | | | | | | | | | |
|---|---|---|---|---|---|---|---|---|---|---|
| GO_KINESI | GO_KINESIN_BINDIN | 41 | -0.3495 | -1.0432 | 0.4165 | 0.529386 | 1 | 15530 | tags=41%, list=2 |
| GO_SYNAP | GO_SYNAPTIC_VESI( | 101 | -0.312 | -1.0329 | 0.4174 | 0.536762 | 1 | 13475 | tags=41%, list=2 |
| GO_ACETY | GO_ACETYLGALACT | 45 | -0.3097 | -1.0149 | 0.4182 | 0.549006 | 1 | 13406 | tags=33%, list=2 |
| GO_MICRO | GO_MICROVILLUS_C | 25 | -0.3787 | -1.0337 | 0.4185 | 0.536528 | 1 | 9629 | tags=36%, list=1 |
| GO_N_ACY | GO_N_ACYLTRANSFI | 98 | -0.3045 | -1.048 | 0.4194 | 0.526365 | 1 | 12756 | tags=33%, list=2 |
| GO_LONG_ | GO_LONG_TERM_ME | 34 | -0.3255 | -1.0066 | 0.4204 | 0.553365 | 1 | 7944 | tags=29%, list=1 |
| GO_CHROM | GO_CHROMOSOME_S | 91 | -0.3513 | -1.0688 | 0.4208 | 0.516424 | 1 | 17523 | tags=41%, list=3 |
| GO_STRIAT | GO_STRIATED_MUSC | 54 | -0.2837 | -1.0168 | 0.4209 | 0.54678 | 1 | 11495 | tags=33%, list=2 |
| GO_SNARE | GO_SNARE_BINDING | 114 | -0.2798 | -1.0184 | 0.4211 | 0.545931 | 1 | 8423 | tags=28%, list=1 |
| GO_DENDR | GO_DENDRITE_EXTE | 35 | -0.3461 | -1.0213 | 0.4216 | 0.546382 | 1 | 10105 | tags=31%, list=1 |
| GO_NEGAT | GO_NEGATIVE_REGU | 60 | -0.3593 | -1.056 | 0.4221 | 0.521041 | 1 | 11360 | tags=33%, list=1 |
| GO_COLLA | GO_COLLAGEN_ACT | 15 | -0.4046 | -1.0332 | 0.4225 | 0.536496 | 1 | 8211 | tags=40%, list=1 |
| GO_PROLIN | GO_PROLINE_RICH_I | 17 | -0.3649 | -1.0338 | 0.4226 | 0.536551 | 1 | 8997 | tags=41%, list=1 |
| GO_NEGAT | GO_NEGATIVE_REGU | 34 | -0.369 | -1.0613 | 0.4226 | 0.519898 | 1 | 13631 | tags=38%, list=2 |
| GO_REGUL | GO_REGULATION_OI | 175 | -0.2454 | -1.0135 | 0.4233 | 0.549516 | 1 | 8956 | tags=23%, list=1 |
| GO_INTRA( | GO_INTRACILIARY_T | 52 | -0.3651 | -1.065 | 0.4235 | 0.519576 | 1 | 8248 | tags=31%, list=1 |
| GO_PROTEI | GO_PROTEIN_O_LINI | 15 | -0.3988 | -1.0464 | 0.4235 | 0.527999 | 1 | 22092 | tags=60%, list=3 |
| GO_SULFUI | GO_SULFUR_COMPO | 355 | -0.2351 | -1.0134 | 0.4235 | 0.549245 | 1 | 10936 | tags=23%, list=1 |
| GO_NEURA | GO_NEURAL_NUCLE | 59 | -0.3067 | -1.0152 | 0.4237 | 0.548831 | 1 | 14256 | tags=37%, list=2 |
| GO_AMINO | GO_AMINOGLYCAN_ | 115 | -0.2796 | -0.9996 | 0.4241 | 0.560297 | 1 | 14148 | tags=35%, list=2 |
| GO_CORTI( | GO_CORTICAL_ACTI | 38 | -0.3406 | -1.0464 | 0.4242 | 0.527758 | 1 | 4264 | tags=21%, list=7 |
| GO_EMBRY | GO_EMBRYONIC_SK | 129 | -0.2724 | -1.0185 | 0.4243 | 0.546093 | 1 | 8585 | tags=25%, list=1 |
| GO_HYDRO | GO_HYDRO_LYASE_ | 51 | -0.3101 | -1.0351 | 0.4244 | 0.536199 | 1 | 7353 | tags=20%, list=1 |
| GO_REGUL | GO_REGULATION_OI | 32 | -0.3206 | -1.0123 | 0.4245 | 0.549849 | 1 | 16781 | tags=38%, list=2 |
| GO_PSEUD | GO_PSEUDOPODIUM | 16 | -0.3579 | -1.0403 | 0.4248 | 0.532014 | 1 | 8364 | tags=38%, list=1 |
| GO_POSITI | GO_POSITIVE_REGUI | 165 | -0.2869 | -1.031 | 0.4248 | 0.538087 | 1 | 19953 | tags=39%, list=3 |
| GO_MEIOTI | GO_MEIOTIC_CELL_( | 177 | -0.2928 | -1.0232 | 0.4253 | 0.54611 | 1 | 21000 | tags=45%, list=3 |
| GO_CYTOP | GO_CYTOPLASMIC_T | 34 | -0.3643 | -1.0208 | 0.4253 | 0.545253 | 1 | 9370 | tags=29%, list=1 |
| GO_REGUL | GO_REGULATION_OI | 59 | -0.3387 | -1.0204 | 0.4258 | 0.545097 | 1 | 14474 | tags=37%, list=2 |
| GO_ENDOT | GO_ENDOTHELIAL_C | 31 | -0.3221 | -1.0186 | 0.4259 | 0.546184 | 1 | 7026 | tags=29%, list=1 |
| GO_BETA_ | GO_BETA_TUBULIN_ | 37 | -0.3148 | -1.0081 | 0.4263 | 0.552956 | 1 | 18442 | tags=46%, list=3 |
| GO_COLLA | GO_COLLAGEN_CON | 422 | -0.3074 | -1.0482 | 0.4265 | 0.526248 | 1 | 14616 | tags=40%, list=2 |
| GO_VIRAL_ | GO_VIRAL_RNA_GEN | 33 | -0.336 | -1.0343 | 0.4269 | 0.536429 | 1 | 5779 | tags=24%, list=1 |
| GO_ESTABI | GO_ESTABLISHMENT | 35 | -0.392 | -1.0541 | 0.4269 | 0.521489 | 1 | 10477 | tags=34%, list=1 |
| GO_L_AMI | GO_L_AMINO_ACID_ | 70 | -0.2916 | -1.0109 | 0.4271 | 0.550912 | 1 | 15413 | tags=36%, list=2 |
| GO_SECON | GO_SECOND_MESSEI | 427 | -0.2542 | -1.0107 | 0.4272 | 0.550933 | 1 | 11534 | tags=32%, list=2 |
| GO_NUCLE | GO_NUCLEAR_RECE | 54 | -0.3116 | -1.0219 | 0.4272 | 0.546499 | 1 | 15151 | tags=43%, list=2 |
| GO_PROTEI | GO_PROTEIN_HYDR( | 25 | -0.3587 | -1.0328 | 0.4274 | 0.536641 | 1 | 12094 | tags=32%, list=2 |
| GO_GAMM | GO_GAMMA_TUBULI | 28 | -0.3395 | -1.0509 | 0.4277 | 0.524135 | 1 | 5942 | tags=21%, list=1 |
| GO_ASYMM | GO_ASYMMETRIC_C | 20 | -0.3604 | -1.0292 | 0.428 | 0.539343 | 1 | 18797 | tags=60%, list=3 |
| GO_ACROS | GO_ACROSOMAL_VE | 111 | -0.2445 | -1.0092 | 0.4286 | 0.552939 | 1 | 10889 | tags=24%, list=1 |
| GO_NEGAT | GO_NEGATIVE_REGU | 32 | -0.3303 | -1.0214 | 0.4288 | 0.546695 | 1 | 6552 | tags=25%, list=1 |
| GO_PHOSPI | GO_PHOSPHATASE_ | 15 | -0.388 | -1.0333 | 0.4294 | 0.536571 | 1 | 10299 | tags=27%, list=1 |
| GO_REGUL | GO_REGULATION_OI | 143 | -0.2759 | -1.0032 | 0.4298 | 0.558476 | 1 | 10853 | tags=29%, list=1 |
| GO_NEGAT | GO_NEGATIVE_REGU | 43 | -0.3319 | -1.035 | 0.4302 | 0.536007 | 1 | 6062 | tags=26%, list=1 |
| GO_REGUL | GO_REGULATION_OI | 89 | -0.3143 | -1.0264 | 0.4303 | 0.541621 | 1 | 15493 | tags=34%, list=2 |
| GO_LYMPH | GO_LYMPHANGIOGE | 15 | -0.4154 | -1.0352 | 0.4303 | 0.536356 | 1 | 13596 | tags=53%, list=2 |
| GO_ASTRO | GO_ASTROCYTE_PR( | 18 | -0.3991 | -1.0392 | 0.4306 | 0.532245 | 1 | 18447 | tags=50%, list=3 |
| GO_GOLGI_ | GO_GOLGI_ORGANIZ | 148 | -0.2693 | -1.0007 | 0.431 | 0.559692 | 1 | 13249 | tags=30%, list=2 |
| GO_REGUL | GO_REGULATION_OI | 155 | -0.3104 | -1.0197 | 0.4316 | 0.545481 | 1 | 12743 | tags=30%, list=2 |
| GO_PROTEI | GO_PROTEIN_ACYLA | 244 | -0.2711 | -1.0001 | 0.4316 | 0.55991 | 1 | 12883 | tags=31%, list=2 |
| GO_ANATO | GO_ANATOMICAL_S | 223 | -0.2416 | -1.0029 | 0.4322 | 0.558208 | 1 | 11099 | tags=25%, list=1 |
| GO_POSITI | GO_POSITIVE_REGUI | 31 | -0.3252 | -1.0224 | 0.4324 | 0.546735 | 1 | 4975 | tags=26%, list=9 |
| GO_NEGAT | GO_NEGATIVE_REGU | 58 | -0.2772 | -1.0082 | 0.4327 | 0.552993 | 1 | 17923 | tags=41%, list=3 |
| GO_NEGAT | GO_NEGATIVE_REGU | 145 | -0.2731 | -1.0163 | 0.4329 | 0.547323 | 1 | 12974 | tags=36%, list=2 |
| GO_TRANS | GO_TRANSEPITHELI | 26 | -0.346 | -1.0114 | 0.4333 | 0.55079 | 1 | 17128 | tags=50%, list=2 |
| GO_PROTE( | GO_PROTEOGLYCAN | 88 | -0.2938 | -1.0032 | 0.4333 | 0.558213 | 1 | 14654 | tags=38%, list=2 |
| GO_THYRO | GO_THYROID_HORM | 26 | -0.3245 | -1.0072 | 0.4337 | 0.553088 | 1 | 12434 | tags=42%, list=2 |

| | | | | | | | | | |
|---|---|---|---|---|---|---|---|---|---|
| GO_BASAL_ | GO_BASAL_PART_OI | 68 | -0.2913 | -1.0141 | 0.4337 | 0.549427 | 1 | 9810 | tags=26%, list=1 |
| GO_TELOM | GO_TELOMERASE_R | 19 | -0.4421 | -1.0656 | 0.4339 | 0.519162 | 1 | 27032 | tags=79%, list=4 |
| GO_POSITI | GO_POSITIVE_REGU | 17 | -0.3674 | -1.0003 | 0.4344 | 0.560113 | 1 | 7991 | tags=24%, list=1 |
| GO_REGUL | GO_REGULATION_OI | 166 | -0.2444 | -0.9986 | 0.4348 | 0.560488 | 1 | 8426 | tags=22%, list=1 |
| GO_FEMAL | GO_FEMALE_MEIOTI | 28 | -0.363 | -1.0213 | 0.4349 | 0.545876 | 1 | 20799 | tags=50%, list=3 |
| GO_NEGAT | GO_NEGATIVE_REGU | 384 | -0.2343 | -0.9937 | 0.4354 | 0.565607 | 1 | 15704 | tags=33%, list=2 |
| GO_CHONI | GO_CHONDROITIN_S | 31 | -0.3595 | -1.0228 | 0.436 | 0.546354 | 1 | 19019 | tags=52%, list=3 |
| GO_LOW_D | GO_LOW_DENSITY_I | 16 | -0.3761 | -1.0173 | 0.4361 | 0.546728 | 1 | 6746 | tags=31%, list=1 |
| GO_COPUL | GO_COPULATION | 20 | -0.3411 | -1.013 | 0.4366 | 0.54954 | 1 | 6239 | tags=25%, list=1 |
| GO_RAB_G | GO_RAB_GTPASE_BI | 168 | -0.2529 | -0.9805 | 0.4371 | 0.578323 | 1 | 7878 | tags=23%, list=1 |
| GO_NUCLE | GO_NUCLEAR_PERIP | 130 | -0.2987 | -1.018 | 0.4377 | 0.546347 | 1 | 12268 | tags=36%, list=2 |
| GO_GLIAL_ | GO_GLIAL_CELL_PR | 50 | -0.283 | -1.0074 | 0.4381 | 0.553251 | 1 | 12629 | tags=36%, list=2 |
| GO_RENAL | GO_RENAL_VESICLE | 20 | -0.3613 | -0.9919 | 0.4381 | 0.566875 | 1 | 5634 | tags=30%, list=1 |
| GO_PHOSP | GO_PHOSPHATIDYLI | 152 | -0.2615 | -0.9992 | 0.4391 | 0.560372 | 1 | 7771 | tags=24%, list=1 |
| GO_TELOM | GO_TELOMERE_CAP | 38 | -0.3469 | -1.0346 | 0.4392 | 0.536355 | 1 | 6594 | tags=29%, list=1 |
| GO_FOREB | GO_FOREBRAIN_REG | 23 | -0.3469 | -1.0169 | 0.4392 | 0.547212 | 1 | 10299 | tags=35%, list=1 |
| GO_REGUL | GO_REGULATION_OI | 18 | -0.4262 | -1.0423 | 0.4393 | 0.529478 | 1 | 6091 | tags=33%, list=1 |
| GO_EXTRIN | GO_EXTRINSIC_COM | 168 | -0.259 | -1.0141 | 0.4393 | 0.549268 | 1 | 9326 | tags=26%, list=1 |
| GO_REGUL | GO_REGULATION_OI | 106 | -0.2923 | -1.0111 | 0.4401 | 0.55077 | 1 | 9810 | tags=27%, list=1 |
| GO_OXIDO | GO_OXIDOREDUCTA | 45 | -0.2984 | -1.0136 | 0.4401 | 0.549591 | 1 | 3386 | tags=13%, list=6 |
| GO_NEGAT | GO_NEGATIVE_REGU | 27 | -0.343 | -0.9986 | 0.4406 | 0.560223 | 1 | 9880 | tags=37%, list=1 |
| GO_RESPO | GO_RESPONSE_TO_P | 439 | -0.2258 | -0.9802 | 0.4407 | 0.578222 | 1 | 9208 | tags=23%, list=1 |
| GO_MALE_ | GO_MALE_SEX_DIFF | 161 | -0.2456 | -1.007 | 0.4412 | 0.553224 | 1 | 11808 | tags=29%, list=2 |
| GO_GENET | GO_GENETIC_IMPRI | 26 | -0.3511 | -1.0215 | 0.4419 | 0.54681 | 1 | 16435 | tags=46%, list=2 |
| GO_NUCLE | GO_NUCLEAR_MATR | 108 | -0.3016 | -1.0187 | 0.4427 | 0.546365 | 1 | 12268 | tags=36%, list=2 |
| GO_POSITI | GO_POSITIVE_REGU | 33 | -0.3697 | -1.0279 | 0.4429 | 0.540811 | 1 | 16562 | tags=45%, list=2 |
| GO_PRC1_C | GO_PRC1_COMPLEX | 15 | -0.3915 | -1.038 | 0.4436 | 0.532656 | 1 | 5223 | tags=33%, list=9 |
| GO_CATEC | GO_CATECHOLAMIN | 52 | -0.2733 | -0.9881 | 0.4442 | 0.571663 | 1 | 8976 | tags=23%, list=1 |
| GO_CLATH | GO_CLATHRIN_COA | 16 | -0.3456 | -1.0146 | 0.4449 | 0.549119 | 1 | 11018 | tags=44%, list=1 |
| GO_CELLU | GO_CELLULAR_AMII | 121 | -0.2628 | -1.0083 | 0.4449 | 0.553194 | 1 | 7085 | tags=18%, list=1 |
| GO_RNA_D | GO_RNA_DESTABILI | 34 | -0.3508 | -1.021 | 0.4451 | 0.545205 | 1 | 5380 | tags=32%, list=9 |
| GO_EPITHE | GO_EPITHELIAL_STR | 26 | -0.319 | -0.998 | 0.4456 | 0.560739 | 1 | 17625 | tags=35%, list=3 |
| GO_VESICI | GO_VESICLE_CYTOS | 72 | -0.3063 | -1.0207 | 0.4458 | 0.545107 | 1 | 6170 | tags=22%, list=1 |
| GO_AMINO | GO_AMINOGLYCAN_ | 170 | -0.2547 | -0.9744 | 0.4458 | 0.580037 | 1 | 14148 | tags=35%, list=2 |
| GO_ENDOP | GO_ENDOPLASMIC_I | 299 | -0.2673 | -0.9799 | 0.446 | 0.57789 | 1 | 13850 | tags=36%, list=2 |
| GO_POSITI | GO_POSITIVE_REGU | 23 | -0.3405 | -0.9921 | 0.4462 | 0.566937 | 1 | 8836 | tags=26%, list=1 |
| GO_REGUL | GO_REGULATION_OI | 117 | -0.264 | -1.0088 | 0.4463 | 0.553122 | 1 | 9901 | tags=28%, list=1 |
| GO_N_ACE | GO_N_ACETYLTRAN | 82 | -0.3091 | -1.0213 | 0.4464 | 0.546126 | 1 | 9645 | tags=28%, list=1 |
| GO_METAL | GO_METALLOCARBO | 29 | -0.3173 | -0.9798 | 0.4472 | 0.577902 | 1 | 25053 | tags=66%, list=4 |
| GO_INSULI | GO_INSULIN_LIKE_G | 23 | -0.3561 | -1.0126 | 0.4478 | 0.549617 | 1 | 22384 | tags=61%, list=3 |
| GO_BRANC | GO_BRANCHING_INV | 23 | -0.3405 | -0.9992 | 0.4479 | 0.560595 | 1 | 9996 | tags=35%, list=1 |
| GO_RESPO | GO_RESPONSE_TO_H | 19 | -0.3592 | -0.9946 | 0.4483 | 0.564362 | 1 | 18609 | tags=53%, list=3 |
| GO_MYOBI | GO_MYOBLAST_PRO | 27 | -0.322 | -0.9978 | 0.4499 | 0.560812 | 1 | 12950 | tags=41%, list=2 |
| GO_REGUL | GO_REGULATION_OI | 39 | -0.321 | -0.999 | 0.4509 | 0.560185 | 1 | 8426 | tags=31%, list=1 |
| GO_POLYS | GO_POLYSACCHARII | 108 | -0.2579 | -0.9922 | 0.4509 | 0.56753 | 1 | 14094 | tags=35%, list=2 |
| GO_INTRIN | GO_INTRINSIC_COMI | 47 | -0.3314 | -1.0075 | 0.4512 | 0.553432 | 1 | 14389 | tags=43%, list=2 |
| GO_FAT_CI | GO_FAT_CELL_DIFFE | 229 | -0.2348 | -0.9852 | 0.4513 | 0.574185 | 1 | 11011 | tags=27%, list=1 |
| GO_ACYL_ | GO_ACYL_COA_BINI | 22 | -0.3454 | -0.9858 | 0.4517 | 0.573746 | 1 | 1159 | tags=14%, list=2 |
| GO_POSITI | GO_POSITIVE_REGU | 297 | -0.2701 | -1.0086 | 0.4526 | 0.553094 | 1 | 18914 | tags=41%, list=3 |
| GO_POSITI | GO_POSITIVE_REGU | 15 | -0.3704 | -0.9968 | 0.4527 | 0.562349 | 1 | 7026 | tags=40%, list=1 |
| GO_POLYS | GO_POLYSACCHARII | 25 | -0.3171 | -0.9911 | 0.4533 | 0.567768 | 1 | 14094 | tags=44%, list=2 |
| GO_REGUL | GO_REGULATION_OI | 98 | -0.3193 | -1.0232 | 0.4535 | 0.545887 | 1 | 7154 | tags=28%, list=1 |
| GO_NEUTR | GO_NEUTROPHIL_HO | 16 | -0.3863 | -1.0139 | 0.4537 | 0.549366 | 1 | 359 | tags=13%, list=1 |
| GO_CILIAR | GO_CILIARY_BASAL | 94 | -0.3385 | -1.0212 | 0.4538 | 0.545612 | 1 | 8595 | tags=26%, list=1 |
| GO_NEGAT | GO_NEGATIVE_REGU | 20 | -0.3388 | -1.007 | 0.454 | 0.552967 | 1 | 9127 | tags=40%, list=1 |
| GO_CALCIU | GO_CALCIUM_ACTIV | 25 | -0.3375 | -1.0031 | 0.454 | 0.558008 | 1 | 11874 | tags=32%, list=2 |
| GO_CIRCAI | GO_CIRCADIAN_REG | 64 | -0.2885 | -0.9921 | 0.454 | 0.567094 | 1 | 11334 | tags=38%, list=1 |
| GO_HISTOI | GO_HISTONE_BINDII | 230 | -0.3131 | -1.0191 | 0.4549 | 0.54595 | 1 | 6879 | tags=25%, list=1 |

| | | | | | | | | | |
|---|---|---|---|---|---|---|---|---|---|
| GO_HEPAT | GO_HEPATICOBILIAI | 144 | -0.251 | -0.9996 | 0.4552 | 0.560552 | 1 | 11335 | tags=28%, list=1 |
| GO_PROTEI | GO_PROTEIN_SERINI | 75 | -0.2739 | -1.0074 | 0.4554 | 0.55298 | 1 | 11153 | tags=32%, list=1 |
| GO_REGUL | GO_REGULATION_OI | 27 | -0.3783 | -1.0161 | 0.4554 | 0.547448 | 1 | 7690 | tags=33%, list=1 |
| GO_COPII_ | GO_COPII_VESICLE_ | 15 | -0.3908 | -0.9954 | 0.4554 | 0.563267 | 1 | 11751 | tags=33%, list=2 |
| GO_TERMI | GO_TERMINAL_BOU | 56 | -0.3232 | -1.0057 | 0.4555 | 0.554565 | 1 | 16853 | tags=46%, list=2 |
| GO_AMINO | GO_AMINO_ACID_IM | 44 | -0.3109 | -0.9926 | 0.456 | 0.566997 | 1 | 8909 | tags=27%, list=1 |
| GO_POSITI | GO_POSITIVE_REGUI | 51 | -0.3078 | -0.9985 | 0.457 | 0.560169 | 1 | 8726 | tags=27%, list=1 |
| GO_OSTEO | GO_OSTEOBLAST_DI | 19 | -0.3394 | -1.0143 | 0.4571 | 0.549449 | 1 | 16015 | tags=53%, list=2 |
| GO_SPROU | GO_SPROUTING_ANG | 186 | -0.2461 | -0.9699 | 0.4572 | 0.583735 | 1 | 9208 | tags=20%, list=1 |
| GO_ALPHA | GO_ALPHA_AMINO_ | 190 | -0.2473 | -0.996 | 0.4575 | 0.562783 | 1 | 10956 | tags=24%, list=1 |
| GO_POSITI | GO_POSITIVE_REGUI | 23 | -0.3511 | -1.0013 | 0.4586 | 0.559487 | 1 | 9901 | tags=39%, list=1 |
| GO_GUANY | GO_GUANYL_NUCLE | 210 | -0.2508 | -0.9815 | 0.4587 | 0.577155 | 1 | 7772 | tags=20%, list=1 |
| GO_INTERC | GO_INTERCELLULAF | 70 | -0.278 | -0.9803 | 0.4588 | 0.578359 | 1 | 11877 | tags=29%, list=2 |
| GO_SEMAP | GO_SEMAPHORIN_PI | 40 | -0.2952 | -1.0028 | 0.4588 | 0.558039 | 1 | 7998 | tags=30%, list=1 |
| GO_REGEN | GO_REGENERATION | 196 | -0.2437 | -0.9885 | 0.4589 | 0.571241 | 1 | 8707 | tags=23%, list=1 |
| GO_GAS_TI | GO_GAS_TRANSPOR | 20 | -0.3644 | -1.0003 | 0.4592 | 0.559857 | 1 | 4675 | tags=20%, list=8 |
| GO_LOCAL | GO_LOCALIZATION_ | 95 | -0.2765 | -0.9866 | 0.4597 | 0.573175 | 1 | 8836 | tags=29%, list=1 |
| GO_FERRO | GO_FERROUS_IRON_ | 26 | -0.3147 | -0.9964 | 0.4602 | 0.562729 | 1 | 13270 | tags=35%, list=2 |
| GO_REGUL | GO_REGULATION_OI | 63 | -0.3285 | -1.0008 | 0.4605 | 0.559896 | 1 | 15678 | tags=46%, list=2 |
| GO_HYPER | GO_HYPEROSMOTIC_ | 28 | -0.3042 | -0.9992 | 0.4611 | 0.560141 | 1 | 10608 | tags=32%, list=1 |
| GO_CALCIU | GO_CALCIUM_ION_R | 23 | -0.3931 | -1.0091 | 0.4612 | 0.552809 | 1 | 19869 | tags=52%, list=3 |
| GO_BLAST | GO_BLASTOCYST_GI | 20 | -0.3752 | -1.0016 | 0.4612 | 0.559311 | 1 | 12950 | tags=35%, list=2 |
| GO_NEGAT | GO_NEGATIVE_REGU | 49 | -0.2873 | -1.0132 | 0.4617 | 0.549439 | 1 | 13913 | tags=31%, list=2 |
| GO_PHOSPI | GO_PHOSPHOLIPID_I | 263 | -0.2338 | -0.9763 | 0.4618 | 0.579516 | 1 | 9956 | tags=24%, list=1 |
| GO_ENTER | GO_ENTERIC_NERVO | 15 | -0.4077 | -1.0037 | 0.4627 | 0.557918 | 1 | 13955 | tags=47%, list=2 |
| GO_CELLU | GO_CELLULAR_HYPI | 15 | -0.3567 | -0.9841 | 0.4644 | 0.57467 | 1 | 13811 | tags=40%, list=2 |
| GO_BLOOD | GO_BLOOD_VESSEL_ | 183 | -0.2417 | -0.9807 | 0.4647 | 0.578203 | 1 | 11823 | tags=26%, list=2 |
| GO_DNA_M | GO_DNA_METHYLAT | 94 | -0.2895 | -0.9908 | 0.4656 | 0.568078 | 1 | 16537 | tags=35%, list=2 |
| GO_PHOSPI | GO_PHOSPHATIDYLI | 16 | -0.3741 | -1.0086 | 0.466 | 0.552981 | 1 | 8398 | tags=38%, list=1 |
| GO_PHOSPI | GO_PHOSPHATIDYLI | 27 | -0.3189 | -0.9873 | 0.4668 | 0.572536 | 1 | 2684 | tags=19%, list=5 |
| GO_CELLU | GO_CELLULAR_RESI | 22 | -0.3296 | -0.9801 | 0.467 | 0.578071 | 1 | 5278 | tags=18%, list=9 |
| GO_RESPOI | GO_RESPONSE_TO_K | 199 | -0.235 | -0.9852 | 0.4671 | 0.574424 | 1 | 5228 | tags=16%, list=9 |
| GO_RAC_PI | GO_RAC_PROTEIN_S | 37 | -0.3051 | -0.9958 | 0.4678 | 0.562919 | 1 | 8997 | tags=35%, list=1 |
| GO_MITOTI | GO_MITOTIC_SPINDI | 127 | -0.2996 | -0.9737 | 0.468 | 0.580185 | 1 | 11553 | tags=27%, list=2 |
| GO_SOLUT | GO_SOLUTE_ANION_ | 28 | -0.3251 | -0.9962 | 0.4692 | 0.562806 | 1 | 16343 | tags=39%, list=2 |
| GO_PROXII | GO_PROXIMAL_DIST | 33 | -0.3335 | -0.9792 | 0.4697 | 0.578378 | 1 | 8254 | tags=30%, list=1 |
| GO_REGUL | GO_REGULATION_OI | 23 | -0.3706 | -1.0012 | 0.4701 | 0.559424 | 1 | 1712 | tags=17%, list=3 |
| GO_CHAPE | GO_CHAPERONE_CO | 25 | -0.3486 | -0.9725 | 0.4701 | 0.580799 | 1 | 18833 | tags=48%, list=3 |
| GO_NEGAT | GO_NEGATIVE_REGU | 34 | -0.295 | -0.9835 | 0.4702 | 0.575288 | 1 | 13129 | tags=24%, list=2 |
| GO_CELLU | GO_CELLULAR_MON | 113 | -0.2581 | -0.9776 | 0.4704 | 0.578569 | 1 | 10114 | tags=27%, list=1 |
| GO_NEGAT | GO_NEGATIVE_REGU | 39 | -0.2993 | -0.9719 | 0.4706 | 0.581455 | 1 | 16193 | tags=38%, list=2 |
| GO_AMYLO | GO_AMYLOID_PREC | 18 | -0.3321 | -0.9933 | 0.4707 | 0.566186 | 1 | 5855 | tags=22%, list=1 |
| GO_METHY | GO_METHYLATION | 333 | -0.2727 | -0.9899 | 0.4709 | 0.569436 | 1 | 16653 | tags=37%, list=2 |
| GO_PRIMA | GO_PRIMARY_ALCO | 94 | -0.2588 | -0.9824 | 0.4712 | 0.57637 | 1 | 15827 | tags=33%, list=2 |
| GO_EXTRA | GO_EXTRACELLULA | 387 | -0.2924 | -0.9777 | 0.4715 | 0.578894 | 1 | 10359 | tags=31%, list=1 |
| GO_SPHING | GO_SPHINGOID_MET | 18 | -0.3509 | -0.9874 | 0.4717 | 0.572626 | 1 | 5845 | tags=22%, list=1 |
| GO_REGUL | GO_REGULATION_OI | 42 | -0.3205 | -0.9781 | 0.4717 | 0.578835 | 1 | 14502 | tags=45%, list=2 |
| GO_REGUL | GO_REGULATION_OI | 56 | -0.2888 | -0.9843 | 0.4734 | 0.574968 | 1 | 6438 | tags=23%, list=1 |
| GO_ENDOP | GO_ENDOPLASMIC_I | 120 | -0.2601 | -0.9831 | 0.4734 | 0.575576 | 1 | 7176 | tags=20%, list=1 |
| GO_PRERIE | GO_PRERIBOSOME | 72 | -0.3383 | -0.9727 | 0.4739 | 0.580695 | 1 | 3469 | tags=18%, list=6 |
| GO_RNA_P | GO_RNA_POLYADEN | 42 | -0.3453 | -0.9897 | 0.4744 | 0.569443 | 1 | 7870 | tags=29%, list=1 |
| GO_POSITI | GO_POSITIVE_REGUI | 65 | -0.2862 | -0.9873 | 0.4749 | 0.572333 | 1 | 9647 | tags=20%, list=1 |
| GO_HOMOI | GO_HOMOLOGOUS_ | 42 | -0.3034 | -0.9686 | 0.475 | 0.584156 | 1 | 17563 | tags=38%, list=3 |
| GO_SEROT | GO_SEROTONIN_TRA | 21 | -0.342 | -0.9692 | 0.4752 | 0.584088 | 1 | 9389 | tags=33%, list=1 |
| GO_POSITI | GO_POSITIVE_REGUI | 36 | -0.3125 | -0.983 | 0.4754 | 0.575474 | 1 | 6753 | tags=19%, list=1 |
| GO_MITOTI | GO_MITOTIC_CYTOK | 71 | -0.3108 | -0.984 | 0.4759 | 0.574587 | 1 | 8593 | tags=24%, list=1 |
| GO_RESPOI | GO_RESPONSE_TO_E | 45 | -0.298 | -0.9753 | 0.476 | 0.579757 | 1 | 8211 | tags=27%, list=1 |
| GO_NEGAT | GO_NEGATIVE_REGU | 25 | -0.367 | -1.0113 | 0.4762 | 0.550675 | 1 | 3475 | tags=20%, list=6 |

| | | | | | | | | | |
|---|---|---|---|---|---|---|---|---|---|
| GO_DETER | GO_DETERMINATION | 16 | -0.3646 | -0.9912 | 0.4765 | 0.567865 | 1 | 18070 | tags=56%, list=3 |
| GO_NEGAT | GO_NEGATIVE_REGU | 248 | -0.2486 | -0.9791 | 0.4771 | 0.578265 | 1 | 6622 | tags=20%, list=1 |
| GO_G_PRO | GO_G_PROTEIN_COU | 241 | -0.2542 | -0.9766 | 0.4776 | 0.579189 | 1 | 20489 | tags=49%, list=3 |
| GO_REGUL | GO_REGULATION_OF | 24 | -0.3614 | -0.9922 | 0.4782 | 0.567305 | 1 | 10105 | tags=33%, list=1 |
| GO_REGUL | GO_REGULATION_OF | 16 | -0.4408 | -1.0402 | 0.4782 | 0.531991 | 1 | 5865 | tags=31%, list=1 |
| GO_PROTE | GO_PROTEIN_HETER | 22 | -0.3337 | -0.974 | 0.4782 | 0.580157 | 1 | 8456 | tags=32%, list=1 |
| GO_MRNA_ | GO_MRNA_5_UTR_BI | 26 | -0.3491 | -0.9792 | 0.4784 | 0.578617 | 1 | 6459 | tags=23%, list=1 |
| GO_G_PRO | GO_G_PROTEIN_COU | 15 | -0.3891 | -1.0025 | 0.4787 | 0.558234 | 1 | 16646 | tags=53%, list=2 |
| GO_PHOSP | GO_PHOSPHATIDYLC | 18 | -0.3394 | -0.9714 | 0.481 | 0.582033 | 1 | 8929 | tags=33%, list=1 |
| GO_MECHA | GO_MECHANOSENSO | 15 | -0.3733 | -0.979 | 0.4813 | 0.57806 | 1 | 20089 | tags=67%, list=3 |
| GO_TRANS | GO_TRANSCRIPTION | 481 | -0.2512 | -0.9768 | 0.4816 | 0.579113 | 1 | 8870 | tags=23%, list=1 |
| GO_GLUCO | GO_GLUCOSAMINE_ | 24 | -0.322 | -0.9753 | 0.4818 | 0.579253 | 1 | 8437 | tags=29%, list=1 |
| GO_REGUL | GO_REGULATION_OF | 128 | -0.2377 | -0.9801 | 0.4826 | 0.577916 | 1 | 7458 | tags=18%, list=1 |
| GO_PHOSP | GO_PHOSPHATIDYLI | 179 | -0.2399 | -0.9681 | 0.4826 | 0.584137 | 1 | 9535 | tags=24%, list=1 |
| GO_COVAL | GO_COVALENT_CHR | 460 | -0.2735 | -0.9822 | 0.4828 | 0.576341 | 1 | 12756 | tags=31%, list=2 |
| GO_POSITI | GO_POSITIVE_REGU | 20 | -0.3409 | -0.9774 | 0.4831 | 0.578622 | 1 | 17171 | tags=40%, list=2 |
| GO_REGUL | GO_REGULATION_OF | 40 | -0.2889 | -0.9863 | 0.4832 | 0.57349 | 1 | 7461 | tags=23%, list=1 |
| GO_ADAPT | GO_ADAPTATION_OF | 21 | -0.3192 | -0.9746 | 0.4833 | 0.579973 | 1 | 6745 | tags=29%, list=1 |
| GO_M_BAN | GO_M_BAND | 25 | -0.3171 | -0.9698 | 0.4837 | 0.583674 | 1 | 6954 | tags=24%, list=1 |
| GO_MANNO | GO_MANNOSIDASE_ | 15 | -0.3702 | -0.9669 | 0.4839 | 0.584675 | 1 | 6183 | tags=33%, list=1 |
| GO_PROTE | GO_PROTEIN_DEPHO | 316 | -0.2311 | -0.9678 | 0.4859 | 0.583867 | 1 | 10136 | tags=25%, list=1 |
| GO_REGUL | GO_REGULATION_OF | 211 | -0.2459 | -0.9575 | 0.4862 | 0.593026 | 1 | 11638 | tags=29%, list=2 |
| GO_ACETY | GO_ACETYL_COA_BI | 16 | -0.3671 | -0.9753 | 0.4866 | 0.579487 | 1 | 15930 | tags=38%, list=2 |
| GO_STARTI | GO_STARTLE_RESPO | 27 | -0.3446 | -0.9756 | 0.4868 | 0.579516 | 1 | 18325 | tags=48%, list=3 |
| GO_RNA_B | GO_RNA_BINDING_IN | 259 | -0.2715 | -1.0016 | 0.4871 | 0.559586 | 1 | 23672 | tags=33%, list=4 |
| GO_UDP_N | GO_UDP_N_ACETYLC | 16 | -0.3716 | -0.974 | 0.4873 | 0.58036 | 1 | 10761 | tags=44%, list=1 |
| GO_S_ADE | GO_S_ADENOSYLME | 138 | -0.2792 | -0.9609 | 0.4875 | 0.589368 | 1 | 14041 | tags=33%, list=2 |
| GO_NEURO | GO_NEUROTRANSMI | 46 | -0.3362 | -0.9858 | 0.4876 | 0.573929 | 1 | 6136 | tags=28%, list=1 |
| GO_PROTE | GO_PROTEIN_SERINE | 422 | -0.241 | -0.9662 | 0.4876 | 0.585282 | 1 | 8398 | tags=23%, list=1 |
| GO_MUCOP | GO_MUCOPOLYSACC | 111 | -0.2786 | -0.9752 | 0.4881 | 0.579209 | 1 | 13893 | tags=35%, list=2 |
| GO_METHY | GO_METHYL_CPG_BI | 24 | -0.3462 | -0.9817 | 0.4884 | 0.576921 | 1 | 2333 | tags=13%, list=4 |
| GO_MITOC | GO_MITOCHONDRIA | 81 | -0.2581 | -0.9547 | 0.4885 | 0.595894 | 1 | 10643 | tags=26%, list=1 |
| GO_TRANS | GO_TRANSITION_ME | 37 | -0.3042 | -0.9628 | 0.4886 | 0.588309 | 1 | 16073 | tags=46%, list=2 |
| GO_REGUL | GO_REGULATION_OF | 30 | -0.3129 | -0.9614 | 0.4892 | 0.589118 | 1 | 8024 | tags=27%, list=1 |
| GO_HETER | GO_HETEROTRIMERI | 33 | -0.3295 | -0.9845 | 0.4897 | 0.575133 | 1 | 13655 | tags=45%, list=2 |
| GO_PHASIC | GO_PHASIC_SMOOTH | 21 | -0.338 | -0.9692 | 0.4906 | 0.583958 | 1 | 15363 | tags=43%, list=2 |
| GO_RHO_G | GO_RHO_GUANYL_N | 60 | -0.2955 | -0.9732 | 0.4908 | 0.580451 | 1 | 10477 | tags=35%, list=1 |
| GO_POSITI | GO_POSITIVE_REGU | 231 | -0.2497 | -0.9381 | 0.4909 | 0.610773 | 1 | 10943 | tags=28%, list=1 |
| GO_NEURO | GO_NEUROEPITHELI | 54 | -0.2981 | -0.969 | 0.4912 | 0.583978 | 1 | 16442 | tags=41%, list=2 |
| GO_CELLU | GO_CELLULAR_RESP | 220 | -0.2317 | -0.9597 | 0.4917 | 0.590966 | 1 | 10177 | tags=24%, list=1 |
| GO_POSITI | GO_POSITIVE_REGU | 27 | -0.3207 | -0.9602 | 0.4919 | 0.590386 | 1 | 21115 | tags=44%, list=3 |
| GO_MEIOSI | GO_MEIOSIS_I_CELL | 112 | -0.282 | -0.9559 | 0.4935 | 0.59476 | 1 | 6091 | tags=18%, list=1 |
| GO_MEMBI | GO_MEMBRANE_COA | 91 | -0.2698 | -0.9615 | 0.494 | 0.5892 | 1 | 12519 | tags=32%, list=2 |
| GO_PROTE | GO_PROTEIN_KINAS | 52 | -0.2645 | -0.9842 | 0.4945 | 0.574893 | 1 | 5779 | tags=21%, list=1 |
| GO_INTRIN | GO_INTRINSIC_COM | 18 | -0.3602 | -0.9695 | 0.4949 | 0.584002 | 1 | 21094 | tags=61%, list=3 |
| GO_G_PRO | GO_G_PROTEIN_BET | 19 | -0.3652 | -0.9776 | 0.495 | 0.578845 | 1 | 13655 | tags=53%, list=2 |
| GO_GOLGI_ | GO_GOLGI_VESICLE_ | 365 | -0.249 | -0.9761 | 0.495 | 0.579154 | 1 | 13249 | tags=30%, list=2 |
| GO_ANGIO | GO_ANGIOGENESIS_ | 30 | -0.3256 | -0.9545 | 0.495 | 0.595944 | 1 | 4750 | tags=23%, list=8 |
| GO_MICRO | GO_MICROTUBULE_ | 29 | -0.3291 | -0.9571 | 0.4951 | 0.593331 | 1 | 7579 | tags=24%, list=1 |
| GO_REGUL | GO_REGULATION_OF | 61 | -0.2819 | -0.9778 | 0.4952 | 0.579066 | 1 | 12883 | tags=31%, list=2 |
| GO_CAJAL_ | GO_CAJAL_BODY | 75 | -0.3136 | -0.9787 | 0.4952 | 0.578094 | 1 | 19578 | tags=60%, list=3 |
| GO_REGUL | GO_REGULATION_OF | 58 | -0.2791 | -0.9673 | 0.496 | 0.584546 | 1 | 6746 | tags=21%, list=1 |
| GO_REGUL | GO_REGULATION_OF | 66 | -0.3313 | -0.9623 | 0.4961 | 0.588592 | 1 | 17523 | tags=41%, list=3 |
| GO_REGUL | GO_REGULATION_OF | 80 | -0.263 | -0.9626 | 0.4969 | 0.588337 | 1 | 16570 | tags=36%, list=2 |
| GO_L_AMI | GO_L_AMINO_ACID_ | 57 | -0.2865 | -0.9699 | 0.497 | 0.58395 | 1 | 13965 | tags=32%, list=2 |
| GO_ARACH | GO_ARACHIDONIC_A | 31 | -0.294 | -0.973 | 0.498 | 0.580568 | 1 | 16012 | tags=39%, list=2 |
| GO_LENS_F | GO_LENS_FIBER_CEI | 35 | -0.2952 | -0.9787 | 0.498 | 0.578299 | 1 | 15683 | tags=43%, list=2 |
| GO_ACTIVA | GO_ACTIVATION_OF | 38 | -0.299 | -0.967 | 0.499 | 0.584785 | 1 | 15488 | tags=42%, list=2 |

| | | | | | | | | | | |
|---|---|---|---|---|---|---|---|---|---|---|
| GO_REGUL | GO_REGULATION_OI | 120 | -0.267 | -0.9615 | 0.501 | 0.589441 | 1 | 14094 | tags=34%, list=2 |
| GO_CLATH | GO_CLATHRIN_COAT | 45 | -0.2838 | -0.9657 | 0.501 | 0.585827 | 1 | 12519 | tags=33%, list=2 |
| GO_SYNAP | GO_SYNAPTIC_VESI | 20 | -0.384 | -0.9762 | 0.501 | 0.579307 | 1 | 16005 | tags=50%, list=2 |
| GO_MAMM | GO_MAMMARY_GLA | 32 | -0.2858 | -0.9573 | 0.501 | 0.593102 | 1 | 8067 | tags=31%, list=1 |
| GO_NUCLE | GO_NUCLEOSOME_C | 108 | -0.318 | -0.9758 | 0.5019 | 0.579555 | 1 | 7540 | tags=24%, list=1 |
| GO_OUTER | GO_OUTER_DYNEIN_ | 17 | -0.3515 | -0.952 | 0.5019 | 0.598357 | 1 | 22421 | tags=59%, list=3 |
| GO_POSITI | GO_POSITIVE_REGU | 201 | -0.2483 | -0.9773 | 0.502 | 0.578554 | 1 | 15413 | tags=39%, list=2 |
| GO_NEGAT | GO_NEGATIVE_REGU | 15 | -0.3609 | -0.9618 | 0.5029 | 0.58926 | 1 | 2895 | tags=20%, list=5 |
| GO_PROTEI | GO_PROTEIN_TARGE | 40 | -0.295 | -0.9291 | 0.5029 | 0.620245 | 1 | 15946 | tags=38%, list=2 |
| GO_WW_D | GO_WW_DOMAIN_BI | 30 | -0.306 | -0.9634 | 0.503 | 0.587801 | 1 | 13846 | tags=40%, list=2 |
| GO_OXYGE | GO_OXYGEN_TRANS | 15 | -0.3731 | -0.9486 | 0.503 | 0.6017 | 1 | 4675 | tags=20%, list=8 |
| GO_MITOTI | GO_MITOTIC_DNA_I | 104 | -0.2842 | -0.9414 | 0.503 | 0.609471 | 1 | 8824 | tags=27%, list=1 |
| GO_REGUL | GO_REGULATION_OI | 18 | -0.333 | -0.964 | 0.5039 | 0.587779 | 1 | 5959 | tags=22%, list=1 |
| GO_PHENO | GO_PHENOL_CONTA | 107 | -0.2394 | -0.9653 | 0.504 | 0.586045 | 1 | 12434 | tags=29%, list=2 |
| GO_RNA_P | GO_RNA_PHOSPHOD | 80 | -0.2714 | -0.9583 | 0.5047 | 0.592533 | 1 | 5896 | tags=16%, list=1 |
| GO_REGUL | GO_REGULATION_OI | 17 | -0.389 | -0.9684 | 0.5048 | 0.584166 | 1 | 3895 | tags=24%, list=7 |
| GO_FIBROI | GO_FIBROBLAST_MI | 44 | -0.2897 | -0.951 | 0.5048 | 0.598222 | 1 | 14514 | tags=41%, list=2 |
| GO_POSITI | GO_POSITIVE_REGU | 24 | -0.3243 | -0.9541 | 0.5048 | 0.596244 | 1 | 4975 | tags=29%, list=9 |
| GO_PHOSPI | GO_PHOSPHOLIPASE | 100 | -0.2478 | -0.964 | 0.5049 | 0.58761 | 1 | 9182 | tags=26%, list=1 |
| GO_POSITI | GO_POSITIVE_REGU | 15 | -0.4302 | -0.9843 | 0.5058 | 0.57514 | 1 | 26594 | tags=73%, list=4 |
| GO_POSITI | GO_POSITIVE_REGU | 22 | -0.325 | -0.9645 | 0.5059 | 0.587088 | 1 | 10511 | tags=36%, list=1 |
| GO_SUMO_ | GO_SUMO_TRANSFE | 19 | -0.3346 | -0.9553 | 0.5067 | 0.595401 | 1 | 7426 | tags=21%, list=1 |
| GO_RAB_G | GO_RAB_GUANYL_N | 32 | -0.3104 | -0.9269 | 0.5068 | 0.621654 | 1 | 7749 | tags=22%, list=1 |
| GO_SEGME | GO_SEGMENT_SPECI | 16 | -0.3411 | -0.9549 | 0.5068 | 0.595678 | 1 | 5536 | tags=25%, list=9 |
| GO_TELOM | GO_TELOMERIC_DN | 35 | -0.3465 | -0.9664 | 0.5075 | 0.58528 | 1 | 9146 | tags=29%, list=1 |
| GO_INTRA | GO_INTRACILIARY_ | 40 | -0.3452 | -0.9678 | 0.508 | 0.584136 | 1 | 18904 | tags=52%, list=3 |
| GO_ACETY | GO_ACETYLCHOLINI | 22 | -0.3321 | -0.9412 | 0.5082 | 0.609022 | 1 | 22111 | tags=64%, list=3 |
| GO_REGUL | GO_REGULATION_OI | 113 | -0.3023 | -0.9378 | 0.5095 | 0.610821 | 1 | 12743 | tags=29%, list=2 |
| GO_ESTABI | GO_ESTABLISHMENT | 36 | -0.3389 | -0.9578 | 0.5097 | 0.592863 | 1 | 10477 | tags=31%, list=1 |
| GO_REGUL | GO_REGULATION_OI | 27 | -0.3065 | -0.9395 | 0.5104 | 0.60924 | 1 | 6054 | tags=26%, list=1 |
| GO_STRIAT | GO_STRIATUM_DEVI | 18 | -0.3531 | -0.9735 | 0.5118 | 0.580202 | 1 | 22617 | tags=50%, list=3 |
| GO_NEGAT | GO_NEGATIVE_REGU | 44 | -0.3654 | -0.9634 | 0.512 | 0.588062 | 1 | 11360 | tags=36%, list=1 |
| GO_DENSE | GO_DENSE_CORE_GI | 27 | -0.3602 | -0.9524 | 0.5125 | 0.598107 | 1 | 13742 | tags=44%, list=2 |
| GO_ESTABI | GO_ESTABLISHMENT | 417 | -0.2437 | -0.9414 | 0.5132 | 0.609741 | 1 | 6630 | tags=20%, list=1 |
| GO_SENSOI | GO_SENSORY_PERCI | 166 | -0.28 | -0.9703 | 0.5139 | 0.583547 | 1 | 16160 | tags=37%, list=2 |
| GO_MEMBI | GO_MEMBRANE_REI | 17 | -0.3426 | -0.9419 | 0.5144 | 0.610456 | 1 | 2848 | tags=18%, list=5 |
| GO_NUCLE | GO_NUCLEAR_OUTE | 27 | -0.292 | -0.9519 | 0.5145 | 0.598271 | 1 | 18929 | tags=37%, list=3 |
| GO_CRISTA | GO_CRISTAE_FORMA | 18 | -0.3279 | -0.9454 | 0.515 | 0.606257 | 1 | 4162 | tags=22%, list=7 |
| GO_GLYCII | GO_GLYCINE_METAI | 15 | -0.3602 | -0.9537 | 0.5154 | 0.596761 | 1 | 16964 | tags=47%, list=2 |
| GO_RETRO | GO_RETROMER_COM | 20 | -0.3562 | -0.9413 | 0.5154 | 0.609114 | 1 | 6486 | tags=25%, list=1 |
| GO_POSITI | GO_POSITIVE_REGU | 389 | -0.2512 | -0.9569 | 0.5158 | 0.59329 | 1 | 11153 | tags=25%, list=1 |
| GO_NUCLE | GO_NUCLEAR_PORE | 83 | -0.2943 | -0.9297 | 0.5159 | 0.620196 | 1 | 10044 | tags=29%, list=1 |
| GO_REGUL | GO_REGULATION_OI | 15 | -0.3591 | -0.9681 | 0.5161 | 0.584352 | 1 | 17557 | tags=47%, list=3 |
| GO_PRERIE | GO_PRERIBOSOME_I | 23 | -0.3784 | -0.9399 | 0.5163 | 0.609744 | 1 | 3469 | tags=22%, list=6 |
| GO_BILE_A | GO_BILE_ACID_MET | 48 | -0.2665 | -0.951 | 0.5164 | 0.598494 | 1 | 9049 | tags=23%, list=1 |
| GO_REGUL | GO_REGULATION_OI | 36 | -0.3301 | -0.9688 | 0.5172 | 0.584024 | 1 | 13742 | tags=42%, list=2 |
| GO_HORM | GO_HORMONE_MET | 215 | -0.2261 | -0.9632 | 0.5172 | 0.587771 | 1 | 15827 | tags=32%, list=2 |
| GO_MIRNA | GO_MIRNA_METABC | 26 | -0.3486 | -0.9579 | 0.5174 | 0.592913 | 1 | 5380 | tags=23%, list=9 |
| GO_NEGAT | GO_NEGATIVE_REGU | 38 | -0.2849 | -0.9738 | 0.5176 | 0.580251 | 1 | 8882 | tags=26%, list=1 |
| GO_NEURO | GO_NEUROTRANSMI | 27 | -0.3661 | -0.9656 | 0.5176 | 0.585714 | 1 | 10511 | tags=41%, list=1 |
| GO_MORPH | GO_MORPHOGENESI | 55 | -0.2836 | -0.9511 | 0.518 | 0.598759 | 1 | 4326 | tags=20%, list=7 |
| GO_ADENY | GO_ADENYLATE_CY | 125 | -0.2666 | -0.951 | 0.5181 | 0.598592 | 1 | 20038 | tags=54%, list=3 |
| GO_PRONU | GO_PRONUCLEUS | 15 | -0.4154 | -0.9613 | 0.5198 | 0.58893 | 1 | 2606 | tags=20%, list=4 |
| GO_HETER | GO_HETEROCHROM | 49 | -0.3121 | -0.9638 | 0.522 | 0.587532 | 1 | 6893 | tags=27%, list=1 |
| GO_REGUL | GO_REGULATION_OI | 25 | -0.3118 | -0.9453 | 0.5224 | 0.606108 | 1 | 17429 | tags=44%, list=3 |
| GO_CHRON | GO_CHROMATIN_AS | 143 | -0.2933 | -0.9243 | 0.5242 | 0.62424 | 1 | 4155 | tags=19%, list=7 |
| GO_NEGAT | GO_NEGATIVE_REGU | 65 | -0.2565 | -0.9369 | 0.5251 | 0.611854 | 1 | 3334 | tags=17%, list=6 |
| GO_REGUL | GO_REGULATION_OI | 16 | -0.3183 | -0.9421 | 0.5252 | 0.610441 | 1 | 23440 | tags=56%, list=4 |

| | | | | | | | | | |
|---|---|---|---|---|---|---|---|---|---|
| GO_APICOI | GO_APICOLATERAL_ | 19 | -0.3268 | -0.9589 | 0.5268 | 0.591781 | 1 | 8956 | tags=26%, list=1 |
| GO_HISTON | GO_HISTONE_H4_K5_ | 16 | -0.4083 | -0.9721 | 0.5273 | 0.58139 | 1 | 10844 | tags=38%, list=1 |
| GO_POLYS. | GO_POLYSACCHARII | 77 | -0.2555 | -0.9416 | 0.5278 | 0.610404 | 1 | 20825 | tags=49%, list=3 |
| GO_RESPOI | GO_RESPONSE_TO_M | 19 | -0.3187 | -0.9427 | 0.5279 | 0.609669 | 1 | 19889 | tags=47%, list=3 |
| GO_SISTER | GO_SISTER_CHROMA | 54 | -0.3353 | -0.9595 | 0.5284 | 0.590993 | 1 | 20697 | tags=56%, list=3 |
| GO_REGUL | GO_REGULATION_OI | 24 | -0.3228 | -0.9416 | 0.5286 | 0.61014 | 1 | 9919 | tags=25%, list=1 |
| GO_REGUL | GO_REGULATION_OI | 101 | -0.3253 | -0.9451 | 0.5286 | 0.60632 | 1 | 17523 | tags=43%, list=3 |
| GO_GLIAL_ | GO_GLIAL_CELL_PR | 33 | -0.3235 | -0.9392 | 0.5288 | 0.609424 | 1 | 16357 | tags=45%, list=2 |
| GO_ENDOR | GO_ENDORIBONUCL | 29 | -0.3437 | -0.9402 | 0.5289 | 0.609607 | 1 | 5361 | tags=21%, list=9 |
| GO_NEURO | GO_NEURON_DEATH | 27 | -0.3074 | -0.9415 | 0.5291 | 0.609828 | 1 | 11000 | tags=33%, list=1 |
| GO_ENDOD | GO_ENDODERM_FOR | 54 | -0.3017 | -0.9353 | 0.5305 | 0.613426 | 1 | 10951 | tags=33%, list=1 |
| GO_NEGAT | GO_NEGATIVE_REGU | 18 | -0.3131 | -0.911 | 0.5309 | 0.63537 | 1 | 7458 | tags=33%, list=1 |
| GO_RESPOI | GO_RESPONSE_TO_IS | 31 | -0.2928 | -0.9364 | 0.531 | 0.612248 | 1 | 7944 | tags=19%, list=1 |
| GO_VESICL | GO_VESICLE_MEDIA | 41 | -0.281 | -0.9246 | 0.531 | 0.623916 | 1 | 5006 | tags=20%, list=9 |
| GO_POSITI | GO_POSITIVE_REGUI | 25 | -0.3425 | -0.9681 | 0.5314 | 0.58396 | 1 | 5634 | tags=28%, list=1 |
| GO_INTRA | GO_INTRACILIARY_T | 19 | -0.371 | -0.9553 | 0.5315 | 0.595593 | 1 | 5992 | tags=21%, list=1 |
| GO_POSITI | GO_POSITIVE_REGUI | 33 | -0.2883 | -0.9411 | 0.5315 | 0.608871 | 1 | 15621 | tags=39%, list=2 |
| GO_MATUF | GO_MATURATION_O | 15 | -0.4062 | -0.9478 | 0.5315 | 0.602858 | 1 | 23194 | tags=73%, list=4 |
| GO_REGUL | GO_REGULATION_OI | 75 | -0.2692 | -0.9523 | 0.5322 | 0.598144 | 1 | 12883 | tags=31%, list=2 |
| GO_ESTABI | GO_ESTABLISHMENT | 23 | -0.3605 | -0.9429 | 0.5322 | 0.609648 | 1 | 10459 | tags=30%, list=1 |
| GO_DIOL_E | GO_DIOL_BIOSYNTH | 22 | -0.3217 | -0.9413 | 0.5336 | 0.609328 | 1 | 6020 | tags=23%, list=1 |
| GO_POSITI | GO_POSITIVE_REGUI | 23 | -0.3623 | -0.9306 | 0.5342 | 0.619104 | 1 | 16562 | tags=39%, list=2 |
| GO_PEPTID | GO_PEPTIDYL_SERIN | 326 | -0.2312 | -0.9379 | 0.5352 | 0.610809 | 1 | 12398 | tags=29%, list=2 |
| GO_NEGAT | GO_NEGATIVE_REGU | 19 | -0.3768 | -0.9534 | 0.5355 | 0.596965 | 1 | 8687 | tags=37%, list=1 |
| GO_CELLU | GO_CELLULAR_POLY | 23 | -0.3091 | -0.9399 | 0.5356 | 0.609358 | 1 | 14094 | tags=43%, list=2 |
| GO_CULLIN | GO_CULLIN_RING_U | 156 | -0.2499 | -0.9293 | 0.5363 | 0.620025 | 1 | 6450 | tags=19%, list=1 |
| GO_SPERM | GO_SPERMATID_NU | 19 | -0.3417 | -0.923 | 0.5363 | 0.625454 | 1 | 11656 | tags=37%, list=2 |
| GO_REGUL | GO_REGULATION_OI | 26 | -0.3269 | -0.9512 | 0.5364 | 0.598971 | 1 | 12233 | tags=38%, list=2 |
| GO_MAMM | GO_MAMMARY_GLA | 45 | -0.2687 | -0.9533 | 0.5365 | 0.596876 | 1 | 6450 | tags=27%, list=1 |
| GO_ENDON | GO_ENDONUCLEASE | 42 | -0.3014 | -0.9191 | 0.537 | 0.628155 | 1 | 10581 | tags=24%, list=1 |
| GO_POSITI | GO_POSITIVE_REGUI | 69 | -0.2833 | -0.9345 | 0.5373 | 0.614034 | 1 | 9573 | tags=26%, list=1 |
| GO_ORGAN | GO_ORGANIC_CATIC | 49 | -0.2702 | -0.9287 | 0.5377 | 0.620312 | 1 | 9389 | tags=27%, list=1 |
| GO_TRANS | GO_TRANSCRIPTION | 29 | -0.2788 | -0.9254 | 0.5377 | 0.623396 | 1 | 8815 | tags=24%, list=1 |
| GO_NEGAT | GO_NEGATIVE_REGU | 37 | -0.3757 | -0.9463 | 0.5391 | 0.605071 | 1 | 11360 | tags=35%, list=1 |
| GO_POSITI | GO_POSITIVE_REGUI | 23 | -0.3078 | -0.9407 | 0.5411 | 0.609013 | 1 | 10511 | tags=35%, list=1 |
| GO_REGUL | GO_REGULATION_OI | 60 | -0.2807 | -0.9178 | 0.5412 | 0.628917 | 1 | 11664 | tags=33%, list=2 |
| GO_RNA_P | GO_RNA_POLYMERA | 18 | -0.3402 | -0.9287 | 0.5413 | 0.62017 | 1 | 10889 | tags=33%, list=1 |
| GO_POSITI | GO_POSITIVE_REGUI | 162 | -0.2404 | -0.9335 | 0.5413 | 0.614998 | 1 | 10105 | tags=25%, list=1 |
| GO_APICAI | GO_APICAL_DENDRI | 18 | -0.3432 | -0.9358 | 0.5417 | 0.612692 | 1 | 10937 | tags=28%, list=1 |
| GO_POLY_I | GO_POLY_PURINE_T | 29 | -0.328 | -0.9418 | 0.5417 | 0.610406 | 1 | 10103 | tags=24%, list=1 |
| GO_ENDOD | GO_ENDODERMAL_C | 45 | -0.307 | -0.9124 | 0.5423 | 0.634518 | 1 | 10888 | tags=36%, list=1 |
| GO_MITOC | GO_MITOCHONDRIA | 16 | -0.3589 | -0.9398 | 0.5424 | 0.608953 | 1 | 8465 | tags=31%, list=1 |
| GO_RETIN | GO_RETINAL_GANGI | 20 | -0.3422 | -0.9212 | 0.5431 | 0.627722 | 1 | 5001 | tags=20%, list=9 |
| GO_REGUL | GO_REGULATION_OI | 151 | -0.2952 | -0.9211 | 0.5434 | 0.627701 | 1 | 16716 | tags=36%, list=2 |
| GO_ACTINI | GO_ACTININ_BINDIN | 36 | -0.306 | -0.9199 | 0.5438 | 0.627843 | 1 | 12326 | tags=39%, list=2 |
| GO_AUDIT | GO_AUDITORY_RECI | 19 | -0.3473 | -0.9399 | 0.5444 | 0.609605 | 1 | 1855 | tags=16%, list=3 |
| GO_ETHAN | GO_ETHANOL_META | 22 | -0.3226 | -0.9276 | 0.5445 | 0.621466 | 1 | 15748 | tags=41%, list=2 |
| GO_VESICL | GO_VESICLE_TRANS | 46 | -0.2969 | -0.9336 | 0.5446 | 0.614956 | 1 | 6170 | tags=22%, list=1 |
| GO_SECON | GO_SECONDARY_ME | 59 | -0.2586 | -0.9416 | 0.5448 | 0.609908 | 1 | 15827 | tags=32%, list=2 |
| GO_MONO | GO_MONOVALENT_I | 158 | -0.2402 | -0.9384 | 0.5451 | 0.61041 | 1 | 10114 | tags=24%, list=1 |
| GO_NEGAT | GO_NEGATIVE_REGU | 30 | -0.2818 | -0.9296 | 0.5453 | 0.619928 | 1 | 10299 | tags=27%, list=1 |
| GO_REGUL | GO_REGULATION_OI | 94 | -0.2402 | -0.9515 | 0.5456 | 0.598629 | 1 | 20825 | tags=49%, list=3 |
| GO_CHOND | GO_CHONDROITIN_S | 43 | -0.308 | -0.9262 | 0.5456 | 0.622553 | 1 | 19068 | tags=51%, list=3 |
| GO_POSITI | GO_POSITIVE_REGUI | 58 | -0.2665 | -0.9366 | 0.5462 | 0.612048 | 1 | 9790 | tags=24%, list=1 |
| GO_AXON_ | GO_AXON_ENSHEAT | 23 | -0.3009 | -0.9375 | 0.5464 | 0.611019 | 1 | 11233 | tags=26%, list=1 |
| GO_A_BAN | GO_A_BAND | 37 | -0.2905 | -0.9175 | 0.5465 | 0.629051 | 1 | 6954 | tags=22%, list=1 |
| GO_RELAX | GO_RELAXATION_OI | 30 | -0.3135 | -0.9317 | 0.547 | 0.617434 | 1 | 14054 | tags=43%, list=2 |
| GO_TRNA_ | GO_TRNA_METHYLT | 32 | -0.3115 | -0.895 | 0.5472 | 0.653225 | 1 | 16653 | tags=44%, list=2 |

| | | | | | | | | | |
|---|---|---|---|---|---|---|---|---|---|
| GO_POSITI | GO_POSITIVE_REGUI | 16 | -0.3183 | -0.9198 | 0.5476 | 0.627764 | 1 | 3630 | tags=19%, list=6 |
| GO_NEUTR | GO_NEUTRAL_LIPID_ | 135 | -0.2376 | -0.9407 | 0.5479 | 0.609202 | 1 | 10468 | tags=22%, list=1 |
| GO_NEURO | GO_NEURON_MATUI | 46 | -0.2799 | -0.9275 | 0.5482 | 0.621445 | 1 | 11099 | tags=33%, list=1 |
| GO_ASPAR | GO_ASPARTATE_FAN | 21 | -0.3299 | -0.9361 | 0.5491 | 0.612458 | 1 | 3280 | tags=14%, list=6 |
| GO_NUCLE | GO_NUCLEAR_MEMI | 282 | -0.2349 | -0.9205 | 0.5491 | 0.627954 | 1 | 9389 | tags=24%, list=1 |
| GO_MITOTI | GO_MITOTIC_SPINDI | 111 | -0.3018 | -0.9187 | 0.5504 | 0.628464 | 1 | 8019 | tags=22%, list=1 |
| GO_NEGAT | GO_NEGATIVE_REGU | 78 | -0.2697 | -0.9496 | 0.5509 | 0.600369 | 1 | 2658 | tags=13%, list=5 |
| GO_REGUL | GO_REGULATION_OI | 17 | -0.3154 | -0.9234 | 0.5509 | 0.624929 | 1 | 5959 | tags=24%, list=1 |
| GO_SPINDI | GO_SPINDLE_ORGAN | 174 | -0.2786 | -0.911 | 0.5521 | 0.635868 | 1 | 8019 | tags=22%, list=1 |
| GO_RESPOI | GO_RESPONSE_TO_P | 30 | -0.2978 | -0.9288 | 0.5535 | 0.620512 | 1 | 15363 | tags=43%, list=2 |
| GO_PEPTID | GO_PEPTIDE_RECEPT | 151 | -0.2511 | -0.92 | 0.5542 | 0.627929 | 1 | 13464 | tags=36%, list=2 |
| GO_MICRO | GO_MICROTUBULE_( | 137 | -0.3027 | -0.9206 | 0.5542 | 0.627978 | 1 | 8171 | tags=23%, list=1 |
| GO_RETIN( | GO_RETINOIC_ACID_ | 33 | -0.305 | -0.924 | 0.5545 | 0.624479 | 1 | 11335 | tags=30%, list=1 |
| GO_REGUL | GO_REGULATION_OI | 185 | -0.2392 | -0.9184 | 0.5547 | 0.628288 | 1 | 9173 | tags=23%, list=1 |
| GO_VESICI | GO_VESICLE_TARGE | 92 | -0.2669 | -0.9132 | 0.5551 | 0.633907 | 1 | 15968 | tags=36%, list=2 |
| GO_NEGAT | GO_NEGATIVE_REGU | 86 | -0.2447 | -0.9251 | 0.5551 | 0.623649 | 1 | 15632 | tags=28%, list=2 |
| GO_SPINDI | GO_SPINDLE | 351 | -0.2563 | -0.8886 | 0.5553 | 0.659942 | 1 | 12046 | tags=26%, list=2 |
| GO_ACYL_( | GO_ACYL_COA_LIGA | 16 | -0.3567 | -0.9449 | 0.5558 | 0.606381 | 1 | 8465 | tags=19%, list=1 |
| GO_RESPOI | GO_RESPONSE_TO_S | 27 | -0.2783 | -0.9297 | 0.5562 | 0.620038 | 1 | 16894 | tags=37%, list=2 |
| GO_RESPOI | GO_RESPONSE_TO_A | 34 | -0.2778 | -0.9398 | 0.5564 | 0.609101 | 1 | 16041 | tags=41%, list=2 |
| GO_POLAR | GO_POLARIZED_EPI | 22 | -0.2981 | -0.9274 | 0.5579 | 0.62133 | 1 | 13615 | tags=32%, list=2 |
| GO_FAD_BI | GO_FAD_BINDING | 32 | -0.2772 | -0.9256 | 0.5583 | 0.623221 | 1 | 9494 | tags=25%, list=1 |
| GO_POSITI | GO_POSITIVE_REGUI | 15 | -0.3483 | -0.911 | 0.5584 | 0.6356 | 1 | 9858 | tags=27%, list=1 |
| GO_PROTEI | GO_PROTEIN_CONTA | 32 | -0.3193 | -0.9192 | 0.5589 | 0.628385 | 1 | 6746 | tags=22%, list=1 |
| GO_SEX_DI | GO_SEX_DETERMINA | 24 | -0.3057 | -0.9347 | 0.5592 | 0.614149 | 1 | 11652 | tags=33%, list=2 |
| GO_3_PHOS | GO_3_PHOSPHOADEI | 23 | -0.2941 | -0.9135 | 0.5595 | 0.633714 | 1 | 15354 | tags=39%, list=2 |
| GO_NEGAT | GO_NEGATIVE_REGU | 32 | -0.2988 | -0.9203 | 0.5605 | 0.627759 | 1 | 24245 | tags=66%, list=4 |
| GO_RESPOI | GO_RESPONSE_TO_A | 47 | -0.2597 | -0.9466 | 0.5607 | 0.604801 | 1 | 8929 | tags=28%, list=1 |
| GO_POSITI | GO_POSITIVE_REGUI | 36 | -0.3012 | -0.892 | 0.5613 | 0.656161 | 1 | 6594 | tags=22%, list=1 |
| GO_LAMEL | GO_LAMELLIPODIUN | 67 | -0.263 | -0.9219 | 0.5618 | 0.626911 | 1 | 4975 | tags=19%, list=9 |
| GO_COLLA | GO_COLLAGEN_BIOS | 52 | -0.2892 | -0.8885 | 0.562 | 0.659916 | 1 | 10863 | tags=29%, list=1 |
| GO_REGUL | GO_REGULATION_OI | 62 | -0.2591 | -0.9249 | 0.5631 | 0.623737 | 1 | 8882 | tags=26%, list=1 |
| GO_BASEM | GO_BASEMENT_MEN | 15 | -0.3586 | -0.8932 | 0.564 | 0.654719 | 1 | 7550 | tags=40%, list=1 |
| GO_REGUL | GO_REGULATION_OI | 47 | -0.286 | -0.8967 | 0.5643 | 0.652383 | 1 | 10863 | tags=28%, list=1 |
| GO_LAMEL | GO_LAMELLIPODIUN | 17 | -0.3214 | -0.9269 | 0.5645 | 0.621857 | 1 | 8823 | tags=24%, list=1 |
| GO_ALCOH | GO_ALCOHOL_BINDI | 86 | -0.2425 | -0.9347 | 0.5645 | 0.613974 | 1 | 14556 | tags=34%, list=2 |
| GO_CHRON | GO_CHROMATIN_RE | 182 | -0.2698 | -0.8984 | 0.5651 | 0.651255 | 1 | 6893 | tags=20%, list=1 |
| GO_REGUL | GO_REGULATION_OI | 22 | -0.357 | -0.9136 | 0.5656 | 0.633906 | 1 | 12756 | tags=36%, list=2 |
| GO_CENTR | GO_CENTRIOLE_ASS | 38 | -0.3257 | -0.8897 | 0.5665 | 0.658795 | 1 | 12794 | tags=34%, list=2 |
| GO_ANOIK | GO_ANOIKIS | 33 | -0.28 | -0.9156 | 0.5666 | 0.63141 | 1 | 9443 | tags=30%, list=1 |
| GO_OLIGOI | GO_OLIGODENDROC | 103 | -0.2403 | -0.9341 | 0.5667 | 0.614496 | 1 | 8082 | tags=20%, list=1 |
| GO_FLAVO | GO_FLAVONOID_ME | 16 | -0.3499 | -0.8919 | 0.5668 | 0.656135 | 1 | 12725 | tags=38%, list=2 |
| GO_CONDE | GO_CONDENSED_NU | 94 | -0.286 | -0.8933 | 0.5672 | 0.655151 | 1 | 13830 | tags=34%, list=2 |
| GO_INNER_ | GO_INNER_EAR_REC | 40 | -0.2975 | -0.9219 | 0.5677 | 0.627134 | 1 | 16160 | tags=38%, list=2 |
| GO_REVER | GO_REVERSE_CHOLI | 20 | -0.3334 | -0.921 | 0.5678 | 0.627667 | 1 | 6746 | tags=15%, list=1 |
| GO_CYTOS | GO_CYTOSKELETON | 85 | -0.2744 | -0.9004 | 0.5679 | 0.648852 | 1 | 8593 | tags=22%, list=1 |
| GO_PHOSPI | GO_PHOSPHOLIPID_ | 19 | -0.303 | -0.9146 | 0.5681 | 0.632397 | 1 | 24757 | tags=63%, list=4 |
| GO_ATP_DI | GO_ATP_DEPENDEN | 72 | -0.3015 | -0.8761 | 0.5684 | 0.6741 | 1 | 8549 | tags=24%, list=1 |
| GO_HISTON | GO_HISTONE_DEACE | 75 | -0.2834 | -0.8982 | 0.5697 | 0.651376 | 1 | 8882 | tags=25%, list=1 |
| GO_PODOS | GO_PODOSOME | 29 | -0.3104 | -0.8952 | 0.57 | 0.653142 | 1 | 10477 | tags=34%, list=1 |
| GO_NUCLE | GO_NUCLEOCYTOPL | 29 | -0.3683 | -0.9171 | 0.5706 | 0.629281 | 1 | 8809 | tags=34%, list=1 |
| GO_NEGAT | GO_NEGATIVE_REGU | 15 | -0.3384 | -0.9278 | 0.5706 | 0.621429 | 1 | 24735 | tags=53%, list=4 |
| GO_METAP | GO_METAPHASE_AN | 60 | -0.3166 | -0.8932 | 0.5706 | 0.65491 | 1 | 17523 | tags=40%, list=3 |
| GO_POSITI | GO_POSITIVE_REGUI | 19 | -0.3209 | -0.9132 | 0.5709 | 0.633651 | 1 | 20441 | tags=47%, list=3 |
| GO_RESPOI | GO_RESPONSE_TO_C | 106 | -0.2394 | -0.9197 | 0.5714 | 0.627671 | 1 | 9204 | tags=23%, list=1 |
| GO_BASAL_ | GO_BASAL_PLASMA_ | 50 | -0.2687 | -0.9187 | 0.572 | 0.628192 | 1 | 9810 | tags=26%, list=1 |
| GO_C21_ST | GO_C21_STEROID_HO | 24 | -0.2872 | -0.9299 | 0.5729 | 0.620183 | 1 | 5959 | tags=21%, list=1 |
| GO_INCLUS | GO_INCLUSION_BOD | 76 | -0.2482 | -0.9105 | 0.573 | 0.635924 | 1 | 7861 | tags=22%, list=1 |

| | | | | | | | | | |
|---|---|---|---|---|---|---|---|---|---|
| GO_NUCLE | GO_NUCLEAR_UBIQU | 42 | -0.2859 | -0.8758 | 0.5731 | 0.673864 | 1 | 5398 | tags=19%, list=9 |
| GO_ANION_ | GO_ANION_HOMEOS | 61 | -0.2675 | -0.9146 | 0.5734 | 0.632657 | 1 | 15534 | tags=38%, list=2 |
| GO_ENTRY | GO_ENTRY_OF_BACT | 15 | -0.359 | -0.8975 | 0.574 | 0.651462 | 1 | 441 | tags=13%, list=1 |
| GO_POSITI | GO_POSITIVE_REGUI | 43 | -0.2606 | -0.9174 | 0.575 | 0.629028 | 1 | 6208 | tags=19%, list=1 |
| GO_NUCLE | GO_NUCLEAR_TRAN | 72 | -0.2539 | -0.8943 | 0.575 | 0.653957 | 1 | 7449 | tags=19%, list=1 |
| GO_NEGAT | GO_NEGATIVE_REGU | 25 | -0.3012 | -0.9162 | 0.5752 | 0.630583 | 1 | 12168 | tags=40%, list=2 |
| GO_U1_SNI | GO_U1_SNRNP | 33 | -0.3231 | -0.9039 | 0.5755 | 0.644295 | 1 | 7422 | tags=27%, list=1 |
| GO_MITOTI | GO_MITOTIC_DNA_R | 15 | -0.3914 | -0.9014 | 0.5764 | 0.647951 | 1 | 5672 | tags=20%, list=1 |
| GO_HEPAR | GO_HEPARAN_SULFA | 32 | -0.2987 | -0.915 | 0.5766 | 0.632229 | 1 | 3026 | tags=16%, list=5 |
| GO_NEGAT | GO_NEGATIVE_REGU | 18 | -0.3344 | -0.8737 | 0.5767 | 0.675791 | 1 | 2649 | tags=17%, list=5 |
| GO_RNA_P | GO_RNA_POLYMERA | 46 | -0.2822 | -0.8878 | 0.5768 | 0.660027 | 1 | 5662 | tags=24%, list=1 |
| GO_L_ASC | GO_L_ASCORBIC_AC | 20 | -0.3305 | -0.8956 | 0.5775 | 0.653188 | 1 | 12094 | tags=35%, list=2 |
| GO_REGUL | GO_REGULATION_OI | 15 | -0.3312 | -0.8966 | 0.5781 | 0.652357 | 1 | 3910 | tags=20%, list=7 |
| GO_EPOXY | GO_EPOXYGENASE_ | 20 | -0.3316 | -0.9047 | 0.5787 | 0.643176 | 1 | 16530 | tags=45%, list=2 |
| GO_ESTABI | GO_ESTABLISHMENT | 18 | -0.3631 | -0.8976 | 0.5792 | 0.651625 | 1 | 8398 | tags=28%, list=1 |
| GO_NUCLE | GO_NUCLEAR_IMPOI | 19 | -0.3606 | -0.8925 | 0.5796 | 0.655613 | 1 | 8809 | tags=37%, list=1 |
| GO_REGUL | GO_REGULATION_OI | 16 | -0.3548 | -0.9006 | 0.5799 | 0.648796 | 1 | 15919 | tags=50%, list=2 |
| GO_SKIN_E | GO_SKIN_EPIDERMIS | 90 | -0.2541 | -0.8962 | 0.5813 | 0.652704 | 1 | 8635 | tags=21%, list=1 |
| GO_PEPTID | GO_PEPTIDYL_ARGII | 27 | -0.2918 | -0.9114 | 0.5814 | 0.63581 | 1 | 10781 | tags=22%, list=1 |
| GO_H4_HIS | GO_H4_HISTONE_AC | 44 | -0.3077 | -0.8937 | 0.5819 | 0.654629 | 1 | 18074 | tags=45%, list=3 |
| GO_CLEAV | GO_CLEAVAGE_FUR | 50 | -0.2692 | -0.8855 | 0.5825 | 0.662916 | 1 | 11638 | tags=32%, list=2 |
| GO_HISTON | GO_HISTONE_H3_K4_ | 18 | -0.3737 | -0.9077 | 0.5838 | 0.638916 | 1 | 17788 | tags=44%, list=3 |
| GO_NEGAT | GO_NEGATIVE_REGU | 119 | -0.2314 | -0.9189 | 0.5845 | 0.628359 | 1 | 15632 | tags=29%, list=2 |
| GO_POSITI | GO_POSITIVE_REGUI | 35 | -0.2787 | -0.91 | 0.5848 | 0.63589 | 1 | 11621 | tags=23%, list=2 |
| GO_DNA_H | GO_DNA_HELICASE_ | 75 | -0.3072 | -0.8832 | 0.5848 | 0.666232 | 1 | 9177 | tags=24%, list=1 |
| GO_ENDON | GO_ENDONUCLEOLY | 16 | -0.366 | -0.8819 | 0.5849 | 0.667436 | 1 | 2603 | tags=19%, list=4 |
| GO_TRANS | GO_TRANSFERASE_A | 18 | -0.3187 | -0.8952 | 0.585 | 0.653408 | 1 | 5103 | tags=22%, list=9 |
| GO_REGUL | GO_REGULATION_OI | 46 | -0.2759 | -0.8787 | 0.5858 | 0.671518 | 1 | 12598 | tags=30%, list=2 |
| GO_RESPOI | GO_RESPONSE_TO_A | 33 | -0.2788 | -0.8945 | 0.5859 | 0.653763 | 1 | 7648 | tags=21%, list=1 |
| GO_REGUL | GO_REGULATION_OI | 171 | -0.2338 | -0.8879 | 0.586 | 0.660146 | 1 | 9141 | tags=23%, list=1 |
| GO_TELOM | GO_TELOMERE_MAI | 78 | -0.273 | -0.8656 | 0.586 | 0.682952 | 1 | 6594 | tags=19%, list=1 |
| GO_REGUL | GO_REGULATION_OI | 51 | -0.3185 | -0.8725 | 0.5861 | 0.676904 | 1 | 8399 | tags=24%, list=1 |
| GO_RNA_P | GO_RNA_POLYMERA | 43 | -0.2903 | -0.8833 | 0.5878 | 0.666408 | 1 | 7398 | tags=26%, list=1 |
| GO_DNA_D | GO_DNA_DEMETHYI | 27 | -0.2982 | -0.8962 | 0.5883 | 0.652482 | 1 | 943 | tags=15%, list=2 |
| GO_CELLU | GO_CELLULAR_RESI | 18 | -0.3063 | -0.9113 | 0.5889 | 0.635724 | 1 | 19375 | tags=50%, list=3 |
| GO_MONOS | GO_MONOSACCHARI | 286 | -0.2124 | -0.906 | 0.5893 | 0.641187 | 1 | 9806 | tags=21%, list=1 |
| GO_RAN_G | GO_RAN_GTPASE_BI | 41 | -0.2859 | -0.8648 | 0.5893 | 0.683851 | 1 | 9626 | tags=24%, list=1 |
| GO_COLUM | GO_COLUMNAR_CUI | 60 | -0.2661 | -0.9099 | 0.5894 | 0.63586 | 1 | 6208 | tags=18%, list=1 |
| GO_CONNE | GO_CONNEXIN_COM | 21 | -0.3037 | -0.9102 | 0.5905 | 0.636139 | 1 | 6854 | tags=29%, list=1 |
| GO_GAP_JU | GO_GAP_JUNCTION_ | 21 | -0.3037 | -0.9102 | 0.5905 | 0.635865 | 1 | 6854 | tags=29%, list=1 |
| GO_CARBO | GO_CARBOHYDRATI | 21 | -0.3075 | -0.8917 | 0.5924 | 0.656111 | 1 | 15701 | tags=38%, list=2 |
| GO_RNA_IN | GO_RNA_INTERFERE | 17 | -0.3575 | -0.8773 | 0.5934 | 0.673346 | 1 | 5902 | tags=24%, list=1 |
| GO_NEGAT | GO_NEGATIVE_REGU | 43 | -0.2566 | -0.9125 | 0.5935 | 0.634583 | 1 | 10490 | tags=28%, list=1 |
| GO_MONO | GO_MONOCARBOXY | 171 | -0.2221 | -0.9321 | 0.5941 | 0.617131 | 1 | 16401 | tags=34%, list=2 |
| GO_PROTEI | GO_PROTEIN_OXIDA | 15 | -0.3378 | -0.8997 | 0.5949 | 0.649827 | 1 | 6746 | tags=27%, list=1 |
| GO_STERE | GO_STEREOCILIUM_ | 18 | -0.3376 | -0.9097 | 0.5953 | 0.635858 | 1 | 15397 | tags=50%, list=2 |
| GO_CLATH | GO_CLATHRIN_COAT | 16 | -0.3329 | -0.8992 | 0.5969 | 0.65041 | 1 | 11018 | tags=44%, list=1 |
| GO_PHOSPI | GO_PHOSPHATIDYLI | 17 | -0.3071 | -0.8978 | 0.5972 | 0.65155 | 1 | 24285 | tags=59%, list=4 |
| GO_CENTR | GO_CENTROSOME_D | 66 | -0.2898 | -0.8567 | 0.598 | 0.691629 | 1 | 12794 | tags=36%, list=2 |
| GO_NEGAT | GO_NEGATIVE_REGU | 51 | -0.2673 | -0.9236 | 0.5985 | 0.62496 | 1 | 6745 | tags=24%, list=1 |
| GO_CYTOK | GO_CYTOKINESIS | 156 | -0.252 | -0.8823 | 0.5988 | 0.66696 | 1 | 11646 | tags=24%, list=2 |
| GO_FOUR_ | GO_FOUR_WAY_JUN | 17 | -0.3842 | -0.8792 | 0.5992 | 0.670882 | 1 | 12831 | tags=41%, list=2 |
| GO_REGUL | GO_REGULATION_OI | 15 | -0.3505 | -0.9094 | 0.6008 | 0.636246 | 1 | 6429 | tags=20%, list=1 |
| GO_HYDRO | GO_HYDROGEN_PER | 32 | -0.2824 | -0.9024 | 0.6015 | 0.646642 | 1 | 4675 | tags=16%, list=8 |
| GO_REGUL | GO_REGULATION_OI | 50 | -0.2522 | -0.8992 | 0.6029 | 0.650232 | 1 | 12398 | tags=32%, list=2 |
| GO_MICRO | GO_MICROTUBULE_ | 132 | -0.2591 | -0.8356 | 0.6035 | 0.717421 | 1 | 14975 | tags=34%, list=2 |
| GO_DIOL_M | GO_DIOL_METABOLI | 25 | -0.2877 | -0.8702 | 0.604 | 0.679331 | 1 | 6020 | tags=20%, list=1 |
| GO_CELL_M | GO_CELL_MATURAT | 170 | -0.2243 | -0.9204 | 0.6045 | 0.627871 | 1 | 10220 | tags=22%, list=1 |

| | | | | | | | | | |
|---|---|---|---|---|---|---|---|---|---|
| GO_TRANS | GO_TRANSLATION_F | 80 | -0.2708 | -0.8866 | 0.6055 | 0.661625 | 1 | 5882 | tags=16%, list=1 |
| GO_POSTSY | GO_POSTSYNAPTIC_ | 20 | -0.3316 | -0.901 | 0.6057 | 0.648305 | 1 | 10511 | tags=40%, list=1 |
| GO_NEGAT | GO_NEGATIVE_REGU | 19 | -0.3029 | -0.8828 | 0.606 | 0.666694 | 1 | 12168 | tags=42%, list=2 |
| GO_HISTON | GO_HISTONE_DEACE | 112 | -0.2366 | -0.8794 | 0.606 | 0.670726 | 1 | 8882 | tags=21%, list=1 |
| GO_CONDE | GO_CONDENSED_CH | 205 | -0.2766 | -0.8535 | 0.6062 | 0.695466 | 1 | 17779 | tags=38%, list=3 |
| GO_POSITI | GO_POSITIVE_REGUI | 37 | -0.2872 | -0.8685 | 0.6068 | 0.681223 | 1 | 8067 | tags=27%, list=1 |
| GO_SMALL | GO_SMALL_NUCLEA | 95 | -0.2783 | -0.8749 | 0.607 | 0.67421 | 1 | 12984 | tags=31%, list=2 |
| GO_ORGAN | GO_ORGANOPHOSPH | 129 | -0.2204 | -0.898 | 0.6072 | 0.651396 | 1 | 10619 | tags=26%, list=1 |
| GO_SPERM | GO_SPERMATID_DIF | 152 | -0.2335 | -0.9073 | 0.6074 | 0.639369 | 1 | 15236 | tags=32%, list=2 |
| GO_STEROI | GO_STEROID_METAE | 326 | -0.2117 | -0.918 | 0.6081 | 0.628713 | 1 | 5277 | tags=14%, list=9 |
| GO_POLY_ | GO_POLY_PYRIMIDII | 29 | -0.2905 | -0.8556 | 0.6081 | 0.692261 | 1 | 9657 | tags=28%, list=1 |
| GO_DERMA | GO_DERMATAN_SUL | 16 | -0.3389 | -0.8955 | 0.6096 | 0.653182 | 1 | 13771 | tags=38%, list=2 |
| GO_TOXIN_ | GO_TOXIN_METABO | 15 | -0.3336 | -0.9022 | 0.6099 | 0.646664 | 1 | 14838 | tags=33%, list=2 |
| GO_POSITI | GO_POSITIVE_REGUI | 38 | -0.2703 | -0.8971 | 0.6107 | 0.652016 | 1 | 15095 | tags=34%, list=2 |
| GO_PERICE | GO_PERICENTRIOLA | 21 | -0.3371 | -0.8782 | 0.611 | 0.672092 | 1 | 14756 | tags=43%, list=2 |
| GO_REGUL | GO_REGULATION_OI | 42 | -0.2657 | -0.9068 | 0.6115 | 0.639894 | 1 | 12745 | tags=33%, list=2 |
| GO_DNA_D | GO_DNA_DOUBLE_S | 24 | -0.328 | -0.8771 | 0.6118 | 0.672821 | 1 | 11005 | tags=33%, list=1 |
| GO_POSITI | GO_POSITIVE_REGUI | 50 | -0.2607 | -0.8766 | 0.6126 | 0.673484 | 1 | 3729 | tags=14%, list=6 |
| GO_VESICI | GO_VESICLE_TARGE | 73 | -0.265 | -0.8835 | 0.6133 | 0.666317 | 1 | 15552 | tags=37%, list=2 |
| GO_PYRUV | GO_PYRUVATE_MET | 148 | -0.2373 | -0.8753 | 0.6142 | 0.673805 | 1 | 9901 | tags=24%, list=1 |
| GO_GROOM | GO_GROOMING_BEH | 19 | -0.3148 | -0.887 | 0.6143 | 0.661153 | 1 | 7522 | tags=32%, list=1 |
| GO_DESMO | GO_DESMOSOME | 25 | -0.3226 | -0.8351 | 0.6147 | 0.717707 | 1 | 5139 | tags=16%, list=9 |
| GO_POSITI | GO_POSITIVE_REGUI | 18 | -0.3437 | -0.8557 | 0.6148 | 0.69268 | 1 | 12664 | tags=44%, list=2 |
| GO_PHOSPI | GO_PHOSPHOLIPASE | 103 | -0.2506 | -0.8892 | 0.6148 | 0.659355 | 1 | 18325 | tags=46%, list=3 |
| GO_ESTABI | GO_ESTABLISHMENT | 52 | -0.2562 | -0.8694 | 0.6151 | 0.680204 | 1 | 18948 | tags=40%, list=3 |
| GO_DNA_R | GO_DNA_REPAIR_CO | 38 | -0.2873 | -0.8574 | 0.6155 | 0.69156 | 1 | 10439 | tags=24%, list=1 |
| GO_REGUL | GO_REGULATION_OI | 37 | -0.2756 | -0.8888 | 0.6175 | 0.659968 | 1 | 4975 | tags=22%, list=9 |
| GO_MICRO | GO_MICROBODY | 130 | -0.2304 | -0.8589 | 0.6218 | 0.690587 | 1 | 11448 | tags=28%, list=2 |
| GO_PROTEI | GO_PROTEIN_ACTIV | 28 | -0.3312 | -0.8677 | 0.6218 | 0.681265 | 1 | 9751 | tags=29%, list=1 |
| GO_COPII_ | GO_COPII_COATED_ | 72 | -0.2556 | -0.8727 | 0.6225 | 0.676992 | 1 | 15552 | tags=35%, list=2 |
| GO_ATPASI | GO_ATPASE_ACTIVI | 407 | -0.2343 | -0.8411 | 0.6228 | 0.710295 | 1 | 11510 | tags=28%, list=2 |
| GO_UBIQUI | GO_UBIQUITIN_PROT | 18 | -0.3101 | -0.8713 | 0.6233 | 0.678153 | 1 | 7307 | tags=28%, list=1 |
| GO_REGUL | GO_REGULATION_OI | 17 | -0.3194 | -0.8773 | 0.624 | 0.673093 | 1 | 12320 | tags=41%, list=2 |
| GO_VITAM | GO_VITAMIN_D_REC | 15 | -0.3624 | -0.8759 | 0.6245 | 0.674231 | 1 | 11914 | tags=33%, list=2 |
| GO_REGUL | GO_REGULATION_OI | 36 | -0.2762 | -0.8677 | 0.6245 | 0.681673 | 1 | 2086 | tags=14%, list=4 |
| GO_MYOTU | GO_MYOTUBE_CELL | 31 | -0.2801 | -0.8696 | 0.625 | 0.680054 | 1 | 5030 | tags=19%, list=9 |
| GO_REGUL | GO_REGULATION_OI | 23 | -0.3053 | -0.8507 | 0.6252 | 0.698492 | 1 | 8088 | tags=26%, list=1 |
| GO_TRANS | GO_TRANSCRIPTION | 61 | -0.2744 | -0.868 | 0.6257 | 0.681749 | 1 | 13219 | tags=30%, list=2 |
| GO_NEGAT | GO_NEGATIVE_REGU | 57 | -0.2502 | -0.9119 | 0.626 | 0.635219 | 1 | 8089 | tags=23%, list=1 |
| GO_STEROI | GO_STEROID_HYDRO | 38 | -0.2724 | -0.8915 | 0.6262 | 0.656219 | 1 | 16530 | tags=34%, list=2 |
| GO_PEPTID | GO_PEPTIDYL_ASPA | 31 | -0.2731 | -0.8369 | 0.6263 | 0.715678 | 1 | 12796 | tags=32%, list=2 |
| GO_MRNA_ | GO_MRNA_3_END_PI | 96 | -0.2755 | -0.8322 | 0.6264 | 0.720932 | 1 | 7870 | tags=22%, list=1 |
| GO_MITOC | GO_MITOCHONDRIA | 24 | -0.2953 | -0.8685 | 0.627 | 0.681464 | 1 | 1656 | tags=17%, list=3 |
| GO_DNA_D | GO_DNA_DEALKYLA | 33 | -0.2739 | -0.8758 | 0.6272 | 0.674116 | 1 | 943 | tags=12%, list=2 |
| GO_LIGASE | GO_LIGASE_ACTIVIT | 22 | -0.3252 | -0.8455 | 0.6274 | 0.70416 | 1 | 15754 | tags=41%, list=2 |
| GO_NEGAT | GO_NEGATIVE_REGU | 77 | -0.2417 | -0.8631 | 0.6286 | 0.685715 | 1 | 10490 | tags=29%, list=1 |
| GO_RNA_D | GO_RNA_DEPENDEN | 68 | -0.2613 | -0.8153 | 0.6286 | 0.740379 | 1 | 6594 | tags=19%, list=1 |
| GO_RETINO | GO_RETINOL_METAE | 47 | -0.2551 | -0.868 | 0.6292 | 0.681564 | 1 | 15748 | tags=32%, list=2 |
| GO_OLIGOI | GO_OLIGODENDROC | 47 | -0.2458 | -0.8899 | 0.6294 | 0.658711 | 1 | 9077 | tags=21%, list=1 |
| GO_REGUL | GO_REGULATION_OI | 184 | -0.2405 | -0.8424 | 0.6297 | 0.70842 | 1 | 16983 | tags=36%, list=2 |
| GO_POSITI | GO_POSITIVE_REGUI | 26 | -0.2832 | -0.8558 | 0.6298 | 0.692817 | 1 | 11005 | tags=31%, list=1 |
| GO_CLATH | GO_CLATHRIN_ADAI | 24 | -0.2885 | -0.8653 | 0.6317 | 0.683321 | 1 | 11018 | tags=29%, list=1 |
| GO_CARBO | GO_CARBON_OXYGI | 66 | -0.2545 | -0.8773 | 0.6337 | 0.672926 | 1 | 9927 | tags=21%, list=1 |
| GO_ESTABI | GO_ESTABLISHMENT | 198 | -0.2503 | -0.8136 | 0.6351 | 0.742961 | 1 | 10044 | tags=24%, list=1 |
| GO_POSTSY | GO_POSTSYNAPTIC_ | 16 | -0.3188 | -0.8814 | 0.6357 | 0.668119 | 1 | 28358 | tags=69%, list=4 |
| GO_INTRAC | GO_INTRACELLULAI | 24 | -0.2786 | -0.8708 | 0.6358 | 0.678829 | 1 | 11233 | tags=25%, list=1 |
| GO_PROTEI | GO_PROTEIN_LOCAL | 33 | -0.2994 | -0.8354 | 0.6364 | 0.71749 | 1 | 27350 | tags=67%, list=4 |
| GO_ACROS | GO_ACROSOME_REA | 38 | -0.2728 | -0.8861 | 0.6365 | 0.662154 | 1 | 19501 | tags=58%, list=3 |

| | | | | | | | | | |
|---|---|---|---|---|---|---|---|---|---|
| GO_RESPOI | GO_RESPONSE_TO_L | 97 | -0.23 | -0.8384 | 0.6387 | 0.713914 | 1 | 11709 | tags=31%, list=2 |
| GO_RETIN( | GO_RETINOL_DEHYI | 20 | -0.3089 | -0.8664 | 0.6389 | 0.681821 | 1 | 21629 | tags=55%, list=3 |
| GO_REGUL | GO_REGULATION_OI | 15 | -0.3133 | -0.8283 | 0.6392 | 0.723541 | 1 | 3308 | tags=20%, list=6 |
| GO_GOLGI_ | GO_GOLGI_CISTERN. | 115 | -0.2224 | -0.888 | 0.6393 | 0.660252 | 1 | 9923 | tags=22%, list=1 |
| GO_PYROP. | GO_PYROPHOSPHAT | 30 | -0.2876 | -0.8525 | 0.6394 | 0.696823 | 1 | 12270 | tags=33%, list=2 |
| GO_REGUL | GO_REGULATION_OI | 80 | -0.2768 | -0.8111 | 0.6397 | 0.746196 | 1 | 18666 | tags=41%, list=3 |
| GO_POLYPI | GO_POLYPEPTIDE_N | 18 | -0.3079 | -0.8544 | 0.6399 | 0.694127 | 1 | 17072 | tags=44%, list=2 |
| GO_BRANC | GO_BRANCHED_CHA | 25 | -0.2968 | -0.8505 | 0.6402 | 0.698383 | 1 | 12830 | tags=32%, list=2 |
| GO_REGUL | GO_REGULATION_OI | 19 | -0.3261 | -0.8704 | 0.6408 | 0.679281 | 1 | 24735 | tags=47%, list=4 |
| GO_WOUNI | GO_WOUND_HEALIN | 18 | -0.3258 | -0.8569 | 0.6409 | 0.691665 | 1 | 4213 | tags=22%, list=7 |
| GO_NEGAT | GO_NEGATIVE_REGU | 61 | -0.2479 | -0.8828 | 0.6416 | 0.666416 | 1 | 13913 | tags=30%, list=2 |
| GO_TRANS. | GO_TRANSLATION_I | 32 | -0.2915 | -0.8587 | 0.6419 | 0.690126 | 1 | 6272 | tags=22%, list=1 |
| GO_REGUL | GO_REGULATION_OI | 23 | -0.3085 | -0.8481 | 0.642 | 0.701452 | 1 | 6438 | tags=26%, list=1 |
| GO_RNA_SI | GO_RNA_SPLICING | 471 | -0.2377 | -0.8295 | 0.6423 | 0.722851 | 1 | 12984 | tags=27%, list=2 |
| GO_RNA_P( | GO_RNA_POLYMERA | 159 | -0.2423 | -0.8521 | 0.6424 | 0.697325 | 1 | 12664 | tags=30%, list=2 |
| GO_RECEP' | GO_RECEPTOR_ANT/ | 32 | -0.2781 | -0.8606 | 0.6433 | 0.688722 | 1 | 12885 | tags=34%, list=2 |
| GO_ACYLG | GO_ACYLGLYCEROL | 38 | -0.2814 | -0.8576 | 0.6433 | 0.691631 | 1 | 16012 | tags=42%, list=2 |
| GO_PIGMEI | GO_PIGMENT_CELL_ | 35 | -0.2839 | -0.8674 | 0.644 | 0.681418 | 1 | 13068 | tags=34%, list=2 |
| GO_UDP_G. | GO_UDP_GLYCOSYL' | 136 | -0.2211 | -0.8884 | 0.644 | 0.65978 | 1 | 13687 | tags=29%, list=2 |
| GO_G_PRO' | GO_G_PROTEIN_COU | 18 | -0.3053 | -0.8714 | 0.6441 | 0.678241 | 1 | 14538 | tags=50%, list=2 |
| GO_POSITI' | GO_POSITIVE_REGUI | 26 | -0.3115 | -0.8285 | 0.6453 | 0.723472 | 1 | 9845 | tags=31%, list=1 |
| GO_REGUL | GO_REGULATION_OI | 19 | -0.3271 | -0.8184 | 0.6456 | 0.736655 | 1 | 5865 | tags=26%, list=1 |
| GO_MRNA_ | GO_MRNA_EXPORT_ | 111 | -0.282 | -0.8297 | 0.6464 | 0.723076 | 1 | 12883 | tags=28%, list=2 |
| GO_ORGAN | GO_ORGANIC_CATIC | 24 | -0.2841 | -0.8619 | 0.6472 | 0.687498 | 1 | 4341 | tags=21%, list=7 |
| GO_BARBE | GO_BARBED_END_A | 21 | -0.2874 | -0.8492 | 0.6475 | 0.70004 | 1 | 12556 | tags=33%, list=2 |
| GO_MOLTII | GO_MOLTING_CYCLI | 110 | -0.2502 | -0.8727 | 0.6485 | 0.676808 | 1 | 8635 | tags=21%, list=1 |
| GO_RELAX | GO_RELAXATION_OI | 16 | -0.3403 | -0.8599 | 0.6491 | 0.689423 | 1 | 16241 | tags=56%, list=2 |
| GO_POSITI' | GO_POSITIVE_REGUI | 48 | -0.2608 | -0.8456 | 0.6492 | 0.704299 | 1 | 6874 | tags=21%, list=1 |
| GO_NUCLE | GO_NUCLEOBASE_C( | 256 | -0.2394 | -0.8248 | 0.6497 | 0.727659 | 1 | 9700 | tags=24%, list=1 |
| GO_INNER' | GO_INNERVATION | 26 | -0.2967 | -0.8467 | 0.6498 | 0.703146 | 1 | 20398 | tags=54%, list=3 |
| GO_HISTOI | GO_HISTONE_DEUBI | 22 | -0.3011 | -0.8252 | 0.6503 | 0.727605 | 1 | 11768 | tags=32%, list=2 |
| GO_PEPTID | GO_PEPTIDYL_L_CY! | 23 | -0.2802 | -0.8322 | 0.651 | 0.721383 | 1 | 7566 | tags=22%, list=1 |
| GO_PROTEI | GO_PROTEIN_SERINI | 38 | -0.2772 | -0.8512 | 0.6515 | 0.698277 | 1 | 8409 | tags=24%, list=1 |
| GO_CHONI | GO_CHONDROITIN_S | 26 | -0.3092 | -0.8277 | 0.652 | 0.724292 | 1 | 19019 | tags=46%, list=3 |
| GO_APOPT( | GO_APOPTOTIC_DNA | 25 | -0.2814 | -0.8556 | 0.6525 | 0.692446 | 1 | 23801 | tags=48%, list=4 |
| GO_DENDR | GO_DENDRITIC_SPIN | 20 | -0.2916 | -0.8724 | 0.6526 | 0.676818 | 1 | 11239 | tags=35%, list=1 |
| GO_PROTEI | GO_PROTEIN_PHOSP | 31 | -0.278 | -0.842 | 0.654 | 0.708912 | 1 | 9654 | tags=29%, list=1 |
| GO_FMN_B | GO_FMN_BINDING | 16 | -0.3064 | -0.8615 | 0.6552 | 0.687903 | 1 | 12725 | tags=31%, list=2 |
| GO_POSITI' | GO_POSITIVE_REGUI | 17 | -0.3143 | -0.8602 | 0.6555 | 0.6892 | 1 | 6594 | tags=29%, list=1 |
| GO_HISTOI | GO_HISTONE_H3_K9_ | 16 | -0.3225 | -0.8315 | 0.6566 | 0.720661 | 1 | 15348 | tags=50%, list=2 |
| GO_FLEMM | GO_FLEMMING_BOD | 31 | -0.2842 | -0.8318 | 0.6575 | 0.720652 | 1 | 11768 | tags=26%, list=2 |
| GO_REGUL | GO_REGULATION_OI | 48 | -0.2506 | -0.8573 | 0.6577 | 0.691507 | 1 | 4975 | tags=19%, list=9 |
| GO_TRANS | GO_TRANSCRIPTION | 47 | -0.2714 | -0.8251 | 0.6586 | 0.727387 | 1 | 13069 | tags=34%, list=2 |
| GO_CAMEF | GO_CAMERA_TYPE_ | 26 | -0.2743 | -0.8677 | 0.6587 | 0.681426 | 1 | 782 | tags=12%, list=1 |
| GO_CALMC | GO_CALMODULIN_D | 28 | -0.2674 | -0.8672 | 0.6588 | 0.681526 | 1 | 12445 | tags=32%, list=2 |
| GO_AMINO | GO_AMINO_ACID_BE | 17 | -0.3064 | -0.823 | 0.659 | 0.729028 | 1 | 1186 | tags=12%, list=2 |
| GO_ESTROI | GO_ESTROUS_CYCLI | 15 | -0.3143 | -0.8666 | 0.659 | 0.681725 | 1 | 694 | tags=13%, list=1 |
| GO_PRODU | GO_PRODUCTION_OI | 51 | -0.2783 | -0.8488 | 0.659 | 0.700487 | 1 | 5902 | tags=20%, list=1 |
| GO_TRANS. | GO_TRANSMEMBRAI | 17 | -0.2968 | -0.8351 | 0.66 | 0.717439 | 1 | 13713 | tags=41%, list=2 |
| GO_FILOPC | GO_FILOPODIUM | 101 | -0.2363 | -0.864 | 0.6603 | 0.684524 | 1 | 11800 | tags=33%, list=2 |
| GO_REGUL | GO_REGULATION_OI | 34 | -0.2763 | -0.8804 | 0.6612 | 0.66925 | 1 | 15095 | tags=44%, list=2 |
| GO_DNA_C | GO_DNA_CATABOLI( | 32 | -0.2683 | -0.8588 | 0.6621 | 0.690327 | 1 | 23801 | tags=44%, list=4 |
| GO_PROTEI | GO_PROTEIN_KINAS | 27 | -0.2743 | -0.8755 | 0.6623 | 0.674073 | 1 | 15209 | tags=41%, list=2 |
| GO_LIGANI | GO_LIGAND_GATED_ | 108 | -0.2406 | -0.861 | 0.6626 | 0.688287 | 1 | 20230 | tags=43%, list=3 |
| GO_POSITI' | GO_POSITIVE_REGUI | 18 | -0.3107 | -0.8319 | 0.6634 | 0.721134 | 1 | 8835 | tags=33%, list=1 |
| GO_RNA_E. | GO_RNA_EXPORT_FI | 142 | -0.2671 | -0.7954 | 0.6641 | 0.765972 | 1 | 12883 | tags=27%, list=2 |
| GO_PHOSPI | GO_PHOSPHOTRANS | 18 | -0.2962 | -0.8263 | 0.6646 | 0.726127 | 1 | 17839 | tags=44%, list=3 |
| GO_CYTOP | GO_CYTOPLASMIC_S | 23 | -0.2736 | -0.8466 | 0.6648 | 0.702734 | 1 | 15888 | tags=48%, list=2 |

| | | | | | | | | | |
|---|---|---|---|---|---|---|---|---|---|
| GO_COLLA | GO_COLLAGEN_BINI | 68 | -0.2957 | -0.8174 | 0.6653 | 0.737445 | 1 | 17625 | tags=51%, list=3 |
| GO_ATTAC | GO_ATTACHMENT_C | 30 | -0.3093 | -0.7656 | 0.668 | 0.800695 | 1 | 22531 | tags=53%, list=3 |
| GO_REGUL | GO_REGULATION_OI | 44 | -0.2515 | -0.8647 | 0.668 | 0.683683 | 1 | 10468 | tags=25%, list=1 |
| GO_REGUL | GO_REGULATION_OI | 47 | -0.2685 | -0.8611 | 0.6686 | 0.688483 | 1 | 8338 | tags=28%, list=1 |
| GO_TRANS | GO_TRANSLATIONAI | 190 | -0.2405 | -0.79 | 0.6687 | 0.772832 | 1 | 7176 | tags=16%, list=1 |
| GO_MEMBI | GO_MEMBRANE_DO | 178 | -0.2298 | -0.8044 | 0.6693 | 0.75409 | 1 | 9326 | tags=23%, list=1 |
| GO_ALKAL | GO_ALKALI_METAL_ | 17 | -0.3129 | -0.85 | 0.6699 | 0.699046 | 1 | 12175 | tags=41%, list=2 |
| GO_POSITI | GO_POSITIVE_REGUI | 20 | -0.2939 | -0.851 | 0.6706 | 0.698296 | 1 | 6062 | tags=20%, list=1 |
| GO_REGUL | GO_REGULATION_OI | 16 | -0.3404 | -0.8425 | 0.6714 | 0.708785 | 1 | 10198 | tags=31%, list=1 |
| GO_NUCLE | GO_NUCLEOBASE_C | 54 | -0.2511 | -0.8596 | 0.6715 | 0.689788 | 1 | 10619 | tags=30%, list=1 |
| GO_PRESYI | GO_PRESYNAPTIC_A | 30 | -0.2817 | -0.8237 | 0.672 | 0.728708 | 1 | 11596 | tags=37%, list=2 |
| GO_PROTEI | GO_PROTEIN_KINAS | 36 | -0.2617 | -0.8731 | 0.6725 | 0.676571 | 1 | 16853 | tags=42%, list=2 |
| GO_MITOC | GO_MITOCHONDRIA | 23 | -0.2826 | -0.8366 | 0.6729 | 0.7159 | 1 | 3579 | tags=13%, list=6 |
| GO_ORGAN | GO_ORGANELLE_FIS | 438 | -0.219 | -0.7839 | 0.6732 | 0.780696 | 1 | 18914 | tags=37%, list=3 |
| GO_INHIBI | GO_INHIBITORY_SYI | 16 | -0.3277 | -0.8305 | 0.6754 | 0.722073 | 1 | 10943 | tags=44%, list=1 |
| GO_POSITI | GO_POSITIVE_REGUI | 38 | -0.2674 | -0.8373 | 0.6759 | 0.715226 | 1 | 8067 | tags=24%, list=1 |
| GO_VACUC | GO_VACUOLAR_PRO | 17 | -0.3088 | -0.8323 | 0.676 | 0.72159 | 1 | 12270 | tags=35%, list=2 |
| GO_HOMOI | GO_HOMOLOGOUS_I | 52 | -0.2563 | -0.8056 | 0.6778 | 0.753706 | 1 | 8562 | tags=21%, list=1 |
| GO_CUL4_I | GO_CUL4_RING_E3_U | 33 | -0.2693 | -0.8008 | 0.6789 | 0.759081 | 1 | 9307 | tags=24%, list=1 |
| GO_PROTO | GO_PROTON_TRANS | 31 | -0.2723 | -0.8233 | 0.679 | 0.729164 | 1 | 12270 | tags=29%, list=2 |
| GO_REGUL | GO_REGULATION_OI | 26 | -0.2894 | -0.8466 | 0.6792 | 0.702954 | 1 | 17984 | tags=42%, list=3 |
| GO_TRANS | GO_TRANSCRIPTION | 46 | -0.258 | -0.8106 | 0.6801 | 0.746822 | 1 | 8088 | tags=24%, list=1 |
| GO_MECHA | GO_MECHANORECEI | 62 | -0.2827 | -0.8753 | 0.6824 | 0.67402 | 1 | 16442 | tags=42%, list=2 |
| GO_SPLEEI | GO_SPLEEN_DEVELC | 34 | -0.2629 | -0.8506 | 0.6832 | 0.698395 | 1 | 5380 | tags=21%, list=9 |
| GO_ENDOR | GO_ENDORIBONUCL | 63 | -0.2336 | -0.8167 | 0.6842 | 0.738531 | 1 | 15983 | tags=30%, list=2 |
| GO_PROTEI | GO_PROTEIN_LOCAL | 42 | -0.2491 | -0.8347 | 0.6848 | 0.717816 | 1 | 18948 | tags=36%, list=3 |
| GO_KINETO | GO_KINETOCHORE | 128 | -0.2667 | -0.7559 | 0.685 | 0.810201 | 1 | 11646 | tags=27%, list=2 |
| GO_POSITI | GO_POSITIVE_REGUI | 19 | -0.288 | -0.8468 | 0.6857 | 0.70323 | 1 | 12506 | tags=42%, list=2 |
| GO_RIBONI | GO_RIBONUCLEOPRO | 455 | -0.229 | -0.7882 | 0.6865 | 0.775357 | 1 | 9172 | tags=20%, list=1 |
| GO_METAL | GO_METALLOENDOI | 16 | -0.308 | -0.8043 | 0.6868 | 0.753971 | 1 | 12162 | tags=44%, list=2 |
| GO_REGUL | GO_REGULATION_OI | 141 | -0.2148 | -0.8479 | 0.6875 | 0.701537 | 1 | 11813 | tags=21%, list=2 |
| GO_REGUL | GO_REGULATION_OI | 39 | -0.273 | -0.8254 | 0.6881 | 0.727453 | 1 | 15184 | tags=31%, list=2 |
| GO_CELLU | GO_CELLULAR_RESI | 62 | -0.2424 | -0.867 | 0.6886 | 0.681395 | 1 | 8947 | tags=23%, list=1 |
| GO_CELLU | GO_CELLULAR_RESI | 15 | -0.3046 | -0.8056 | 0.6892 | 0.75343 | 1 | 20646 | tags=53%, list=3 |
| GO_DNA_C | GO_DNA_CONFORM | 273 | -0.2352 | -0.7568 | 0.6892 | 0.809972 | 1 | 9177 | tags=21%, list=1 |
| GO_PROTEI | GO_PROTEIN_LIPID_ | 54 | -0.2493 | -0.8374 | 0.6902 | 0.715321 | 1 | 6746 | tags=19%, list=1 |
| GO_HISTON | GO_HISTONE_METHY | 76 | -0.2624 | -0.7754 | 0.6911 | 0.790043 | 1 | 16569 | tags=41%, list=2 |
| GO_NUCLE | GO_NUCLEAR_CHRO | 251 | -0.2305 | -0.7466 | 0.6919 | 0.819849 | 1 | 17836 | tags=33%, list=3 |
| GO_LYMPH | GO_LYMPHOID_PRO | 20 | -0.2757 | -0.8233 | 0.6923 | 0.728879 | 1 | 5634 | tags=20%, list=1 |
| GO_MITOC | GO_MITOCHONDRIA | 15 | -0.3192 | -0.7662 | 0.6923 | 0.800219 | 1 | 17399 | tags=47%, list=3 |
| GO_DNA_B | GO_DNA_BINDING_E | 18 | -0.2959 | -0.8156 | 0.6926 | 0.740077 | 1 | 12817 | tags=33%, list=2 |
| GO_MONO | GO_MONOOXYGENA | 101 | -0.2239 | -0.8744 | 0.6932 | 0.674815 | 1 | 12725 | tags=23%, list=2 |
| GO_PROTEI | GO_PROTEIN_DNA_C | 110 | -0.2398 | -0.7953 | 0.6935 | 0.765846 | 1 | 3322 | tags=13%, list=6 |
| GO_PROTEI | GO_PROTEIN_DNA_C | 206 | -0.2374 | -0.7646 | 0.6941 | 0.801522 | 1 | 4155 | tags=14%, list=7 |
| GO_ACTIN_ | GO_ACTIN_BASED_C | 212 | -0.2231 | -0.8645 | 0.6944 | 0.683845 | 1 | 11800 | tags=29%, list=2 |
| GO_POSITI | GO_POSITIVE_REGUI | 30 | -0.2874 | -0.7637 | 0.6946 | 0.802434 | 1 | 7870 | tags=20%, list=1 |
| GO_DNA_P | GO_DNA_POLYMERA | 20 | -0.3163 | -0.7982 | 0.6946 | 0.761465 | 1 | 5902 | tags=25%, list=1 |
| GO_CEREB | GO_CEREBRAL_COR | 25 | -0.2911 | -0.8286 | 0.6959 | 0.723877 | 1 | 19175 | tags=56%, list=3 |
| GO_MITOTI | GO_MITOTIC_CELL_ | 161 | -0.234 | -0.776 | 0.6969 | 0.789929 | 1 | 8824 | tags=22%, list=1 |
| GO_NCRNA | GO_NCRNA_TRANSC | 101 | -0.2428 | -0.7721 | 0.6969 | 0.793517 | 1 | 7201 | tags=19%, list=1 |
| GO_NEGAT | GO_NEGATIVE_REGU | 17 | -0.2913 | -0.841 | 0.6978 | 0.710102 | 1 | 7567 | tags=29%, list=1 |
| GO_REGUL | GO_REGULATION_OI | 58 | -0.252 | -0.7543 | 0.6988 | 0.810906 | 1 | 14495 | tags=36%, list=2 |
| GO_LIPID_I | GO_LIPID_DROPLET_ | 26 | -0.2544 | -0.8408 | 0.6994 | 0.710222 | 1 | 8620 | tags=23%, list=1 |
| GO_GOLGI_ | GO_GOLGI_STACK | 146 | -0.2057 | -0.8319 | 0.6994 | 0.72088 | 1 | 9923 | tags=21%, list=1 |
| GO_REGUL | GO_REGULATION_OI | 54 | -0.2423 | -0.8304 | 0.7002 | 0.72206 | 1 | 20646 | tags=41%, list=3 |
| GO_MICRO | GO_MICROFILAMEN | 29 | -0.2854 | -0.8078 | 0.7006 | 0.750576 | 1 | 11314 | tags=28%, list=1 |
| GO_MLL1_ | GO_MLL1_2_COMPLI | 29 | -0.2913 | -0.7844 | 0.7006 | 0.780356 | 1 | 10844 | tags=31%, list=1 |
| GO_RECEP | GO_RECEPTOR_INHI | 40 | -0.2608 | -0.845 | 0.7016 | 0.704733 | 1 | 12885 | tags=33%, list=2 |

| | | | | | | | | | |
|---|---|---|---|---|---|---|---|---|---|
| GO_POSITI | GO_POSITIVE_REGUI | 15 | -0.2993 | -0.829 | 0.702 | 0.723356 | 1 | 3289 | tags=13%, list=6 |
| GO_INTEGI | GO_INTEGRATOR_CC | 25 | -0.3107 | -0.8046 | 0.7036 | 0.754299 | 1 | 15517 | tags=24%, list=2 |
| GO_BLOOD | GO_BLOOD_COAGUI | 18 | -0.3187 | -0.7915 | 0.7043 | 0.77092 | 1 | 9751 | tags=28%, list=1 |
| GO_CHROM | GO_CHROMOSOME_S | 310 | -0.2215 | -0.7234 | 0.7052 | 0.842967 | 1 | 17836 | tags=33%, list=3 |
| GO_PEPTID | GO_PEPTIDYL_CYST | 49 | -0.2285 | -0.8296 | 0.7056 | 0.72288 | 1 | 7566 | tags=18%, list=1 |
| GO_CORE_ | GO_CORE_PROMOTE | 42 | -0.2635 | -0.8091 | 0.7061 | 0.748511 | 1 | 7201 | tags=21%, list=1 |
| GO_HAIR_C | GO_HAIR_CELL_DIFF | 43 | -0.3069 | -0.857 | 0.7068 | 0.691692 | 1 | 16442 | tags=42%, list=2 |
| GO_TOXIN_ | GO_TOXIN_TRANSPO | 41 | -0.2448 | -0.8285 | 0.707 | 0.72374 | 1 | 10198 | tags=24%, list=1 |
| GO_SNORN | GO_SNORNA_BINDIN | 30 | -0.2796 | -0.7559 | 0.7086 | 0.81043 | 1 | 9146 | tags=23%, list=1 |
| GO_POSITI | GO_POSITIVE_REGUI | 15 | -0.2889 | -0.8006 | 0.7087 | 0.758793 | 1 | 21972 | tags=60%, list=3 |
| GO_CENTR | GO_CENTRIOLE | 131 | -0.2445 | -0.7775 | 0.709 | 0.788949 | 1 | 6486 | tags=18%, list=1 |
| GO_PYRIMI | GO_PYRIMIDINE_RIE | 16 | -0.2826 | -0.7807 | 0.711 | 0.784504 | 1 | 8153 | tags=25%, list=1 |
| GO_CHROM | GO_CHROMOSOME_C | 183 | -0.2539 | -0.7285 | 0.7112 | 0.837706 | 1 | 17779 | tags=38%, list=3 |
| GO_RNA_L | GO_RNA_LOCALIZAT | 231 | -0.232 | -0.7506 | 0.7115 | 0.814763 | 1 | 9014 | tags=21%, list=1 |
| GO_CONDE | GO_CONDENSED_CH | 111 | -0.2511 | -0.7029 | 0.7123 | 0.864922 | 1 | 11646 | tags=26%, list=2 |
| GO_ALCOH | GO_ALCOHOL_DEHY | 21 | -0.2925 | -0.7934 | 0.7132 | 0.768165 | 1 | 27057 | tags=67%, list=4 |
| GO_HISTON | GO_HISTONE_H4_K1 | 21 | -0.3031 | -0.7708 | 0.7135 | 0.79477 | 1 | 16590 | tags=43%, list=2 |
| GO_PARAL | GO_PARALLEL_FIBE | 16 | -0.3087 | -0.8182 | 0.7137 | 0.736462 | 1 | 10254 | tags=31%, list=1 |
| GO_TRAPP_ | GO_TRAPP_COMPLE | 15 | -0.2914 | -0.8126 | 0.7146 | 0.743974 | 1 | 4704 | tags=20%, list=8 |
| GO_PSEUD | GO_PSEUDOURIDINE | 18 | -0.2842 | -0.7546 | 0.7151 | 0.810818 | 1 | 4290 | tags=17%, list=7 |
| GO_ZINC_I | GO_ZINC_ION_HOME | 38 | -0.2648 | -0.799 | 0.7154 | 0.760552 | 1 | 14248 | tags=37%, list=2 |
| GO_GOLGI_ | GO_GOLGI_VESICLE_ | 80 | -0.2383 | -0.8048 | 0.7154 | 0.754307 | 1 | 15552 | tags=34%, list=2 |
| GO_FERTIL | GO_FERTILIZATION | 184 | -0.2072 | -0.8812 | 0.7156 | 0.668081 | 1 | 11099 | tags=23%, list=1 |
| GO_PHOSP | GO_PHOSPHOLIPID_ | 44 | -0.235 | -0.833 | 0.7157 | 0.720523 | 1 | 14517 | tags=34%, list=2 |
| GO_INNER_ | GO_INNER_EAR_AUI | 34 | -0.2895 | -0.8272 | 0.7165 | 0.724834 | 1 | 16442 | tags=41%, list=2 |
| GO_NEGAT | GO_NEGATIVE_REGU | 26 | -0.2953 | -0.7421 | 0.7171 | 0.824528 | 1 | 5527 | tags=15%, list=9 |
| GO_REGUL | GO_REGULATION_OI | 29 | -0.2733 | -0.8007 | 0.7171 | 0.758906 | 1 | 10347 | tags=24%, list=1 |
| GO_CATAL | GO_CATALYTIC_ACT | 99 | -0.2304 | -0.7646 | 0.7173 | 0.80182 | 1 | 15754 | tags=32%, list=2 |
| GO_REGUL | GO_REGULATION_OI | 77 | -0.2468 | -0.7716 | 0.7176 | 0.793676 | 1 | 6594 | tags=21%, list=1 |
| GO_FILOPC | GO_FILOPODIUM_TII | 17 | -0.2907 | -0.7994 | 0.7176 | 0.760196 | 1 | 3074 | tags=18%, list=5 |
| GO_REGUL | GO_REGULATION_OI | 108 | -0.237 | -0.7438 | 0.7179 | 0.823556 | 1 | 8769 | tags=21%, list=1 |
| GO_ODORA | GO_ODORANT_BIND | 101 | -0.2722 | -0.8582 | 0.719 | 0.690732 | 1 | 21322 | tags=35%, list=3 |
| GO_AMINO | GO_AMINO_ACID_BI | 55 | -0.2373 | -0.8316 | 0.7194 | 0.720812 | 1 | 10550 | tags=25%, list=1 |
| GO_REGUL | GO_REGULATION_OI | 94 | -0.236 | -0.8401 | 0.7197 | 0.711113 | 1 | 17322 | tags=34%, list=3 |
| GO_TRANS | GO_TRANSFERASE_A | 70 | -0.224 | -0.8424 | 0.7202 | 0.708689 | 1 | 15562 | tags=33%, list=2 |
| GO_POSITI | GO_POSITIVE_REGUI | 39 | -0.2631 | -0.7671 | 0.7208 | 0.799456 | 1 | 15678 | tags=41%, list=2 |
| GO_NEGAT | GO_NEGATIVE_REGU | 80 | -0.2554 | -0.7674 | 0.7209 | 0.799213 | 1 | 12278 | tags=26%, list=2 |
| GO_NEGAT | GO_NEGATIVE_REGU | 16 | -0.2857 | -0.8046 | 0.721 | 0.754029 | 1 | 5704 | tags=25%, list=1 |
| GO_CATAL | GO_CATALYTIC_ACT | 200 | -0.23 | -0.7597 | 0.7218 | 0.806495 | 1 | 9242 | tags=21%, list=1 |
| GO_REPLIC | GO_REPLICATION_FO | 31 | -0.2775 | -0.7522 | 0.7221 | 0.813365 | 1 | 12831 | tags=42%, list=2 |
| GO_REGUL | GO_REGULATION_OI | 333 | -0.2186 | -0.7615 | 0.7247 | 0.804422 | 1 | 7417 | tags=19%, list=1 |
| GO_HISTON | GO_HISTONE_MRNA_ | 22 | -0.2805 | -0.7667 | 0.7248 | 0.799718 | 1 | 14433 | tags=36%, list=2 |
| GO_HELICA | GO_HELICASE_ACTIV | 156 | -0.2378 | -0.7363 | 0.7248 | 0.829406 | 1 | 9177 | tags=25%, list=1 |
| GO_GLUCO | GO_GLUCOCORTICO | 19 | -0.2763 | -0.8238 | 0.726 | 0.728827 | 1 | 22074 | tags=58%, list=3 |
| GO_POLY_A | GO_POLY_A_PLUS_M | 21 | -0.2938 | -0.8014 | 0.7271 | 0.758209 | 1 | 18666 | tags=38%, list=3 |
| GO_CARBO | GO_CARBOHYDRATE | 208 | -0.2007 | -0.8562 | 0.7283 | 0.692291 | 1 | 9806 | tags=20%, list=1 |
| GO_ALDO_ | GO_ALDO_KETO_REI | 27 | -0.2573 | -0.7742 | 0.7295 | 0.7905 | 1 | 27057 | tags=63%, list=4 |
| GO_SPINDI | GO_SPINDLE_ASSEM | 109 | -0.2314 | -0.7428 | 0.7296 | 0.824571 | 1 | 9173 | tags=18%, list=1 |
| GO_INTERM | GO_INTERMEDIATE_ | 25 | -0.2758 | -0.8041 | 0.7308 | 0.753971 | 1 | 11611 | tags=32%, list=2 |
| GO_SM_LIK | GO_SM_LIKE_PROTE | 107 | -0.2486 | -0.7938 | 0.7318 | 0.767853 | 1 | 12984 | tags=28%, list=2 |
| GO_RACEM | GO_RACEMASE_AND | 16 | -0.2686 | -0.8063 | 0.7319 | 0.752823 | 1 | 9659 | tags=25%, list=1 |
| GO_TERMII | GO_TERMINATION_C | 35 | -0.2761 | -0.7424 | 0.7328 | 0.824354 | 1 | 7203 | tags=20%, list=1 |
| GO_MYOSI | GO_MYOSIN_COMPL | 57 | -0.2497 | -0.8135 | 0.7328 | 0.742842 | 1 | 14648 | tags=33%, list=2 |
| GO_PROTEI | GO_PROTEIN_LOCAL | 21 | -0.2776 | -0.7317 | 0.7345 | 0.833871 | 1 | 17062 | tags=38%, list=2 |
| GO_GLYCE | GO_GLYCEROLIPID_ | 74 | -0.2337 | -0.8589 | 0.7346 | 0.690316 | 1 | 10220 | tags=23%, list=1 |
| GO_PHOSP | GO_PHOSPHATIDYLI | 16 | -0.2971 | -0.7767 | 0.7368 | 0.789911 | 1 | 17096 | tags=38%, list=2 |
| GO_REGUL | GO_REGULATION_OI | 38 | -0.2542 | -0.7596 | 0.7369 | 0.806348 | 1 | 9173 | tags=26%, list=1 |
| GO_DNA_G | GO_DNA_GEOMETRI | 113 | -0.2476 | -0.7367 | 0.7371 | 0.829346 | 1 | 9177 | tags=21%, list=1 |

| | | | | | | | | | |
|---|---|---|---|---|---|---|---|---|---|
| GO_RNA_SI | GO_RNA_SPLICING_V | 381 | -0.2133 | -0.7177 | 0.7371 | 0.847728 | 1 | 12984 | tags=26%, list=2 |
| GO_EPITHE | GO_EPITHELIAL_CEL | 15 | -0.3071 | -0.7474 | 0.738 | 0.818864 | 1 | 16442 | tags=47%, list=2 |
| GO_PROTEI | GO_PROTEIN_N_LINI | 73 | -0.2245 | -0.761 | 0.7381 | 0.80488 | 1 | 14906 | tags=29%, list=2 |
| GO_MRNA_ | GO_MRNA_TRANSPO | 148 | -0.2392 | -0.7394 | 0.7383 | 0.827192 | 1 | 12883 | tags=26%, list=2 |
| GO_SPLICE | GO_SPLICEOSOMAL_ | 54 | -0.2572 | -0.7721 | 0.7388 | 0.793796 | 1 | 9759 | tags=26%, list=1 |
| GO_PROTEI | GO_PROTEIN_LOCAL | 57 | -0.2418 | -0.7511 | 0.7391 | 0.814761 | 1 | 15772 | tags=32%, list=2 |
| GO_TRIGLY | GO_TRIGLYCERIDE_ | 107 | -0.2197 | -0.824 | 0.7395 | 0.728723 | 1 | 10468 | tags=20%, list=1 |
| GO_CYCLIN | GO_CYCLIN_DEPENI | 29 | -0.2445 | -0.779 | 0.7396 | 0.787098 | 1 | 19997 | tags=48%, list=3 |
| GO_CELL_I | GO_CELL_DIVISION_ | 58 | -0.2364 | -0.7908 | 0.7402 | 0.771737 | 1 | 11638 | tags=28%, list=2 |
| GO_H4_H2A | GO_H4_H2A_HISTON | 21 | -0.2863 | -0.7701 | 0.7402 | 0.795658 | 1 | 23608 | tags=67%, list=4 |
| GO_MITOTI | GO_MITOTIC_NUCLE | 266 | -0.2023 | -0.6795 | 0.7424 | 0.8899 | 1 | 19968 | tags=37%, list=3 |
| GO_NEGAT | GO_NEGATIVE_REGU | 59 | -0.2476 | -0.7567 | 0.7432 | 0.809831 | 1 | 12278 | tags=27%, list=2 |
| GO_PROTEI | GO_PROTEIN_HETER | 15 | -0.2992 | -0.7826 | 0.7441 | 0.782299 | 1 | 13986 | tags=40%, list=2 |
| GO_MATUR | GO_MATURATION_O | 25 | -0.292 | -0.7379 | 0.7441 | 0.829029 | 1 | 3054 | tags=16%, list=5 |
| GO_TRANS | GO_TRANSCRIPTION | 405 | -0.2041 | -0.7748 | 0.7443 | 0.790476 | 1 | 13705 | tags=28%, list=2 |
| GO_RNA_3_ | GO_RNA_3_END_PRO | 145 | -0.2339 | -0.7272 | 0.7452 | 0.838929 | 1 | 17509 | tags=33%, list=3 |
| GO_MITOTI | GO_MITOTIC_G2_DN | 21 | -0.2728 | -0.7406 | 0.7454 | 0.825574 | 1 | 18290 | tags=48%, list=3 |
| GO_PRERIE | GO_PRERIBOSOME_S | 15 | -0.3129 | -0.7348 | 0.7457 | 0.830818 | 1 | 1885 | tags=13%, list=3 |
| GO_SINGLE | GO_SINGLE_FERTILI | 153 | -0.2089 | -0.8672 | 0.7462 | 0.681355 | 1 | 10831 | tags=23%, list=1 |
| GO_POSITI | GO_POSITIVE_REGUI | 15 | -0.3043 | -0.7848 | 0.7465 | 0.78002 | 1 | 19965 | tags=60%, list=3 |
| GO_LIPOPR | GO_LIPOPROTEIN_PA | 25 | -0.2733 | -0.7856 | 0.7466 | 0.778872 | 1 | 6746 | tags=20%, list=1 |
| GO_INTRA_ | GO_INTRA_S_DNA_D | 15 | -0.2882 | -0.7229 | 0.7466 | 0.842885 | 1 | 4348 | tags=20%, list=7 |
| GO_PROTEI | GO_PROTEIN_TRANS | 35 | -0.2461 | -0.7554 | 0.7475 | 0.810417 | 1 | 10643 | tags=26%, list=1 |
| GO_NUCLE | GO_NUCLEOBASE_BI | 18 | -0.3069 | -0.7418 | 0.7485 | 0.824684 | 1 | 13876 | tags=39%, list=2 |
| GO_REGUL | GO_REGULATION_OI | 15 | -0.3013 | -0.8054 | 0.751 | 0.753495 | 1 | 16390 | tags=47%, list=2 |
| GO_PROTEI | GO_PROTEIN_PHOSP | 15 | -0.2733 | -0.7785 | 0.7516 | 0.787661 | 1 | 17654 | tags=47%, list=3 |
| GO_MITOTI | GO_MITOTIC_SPINDI | 61 | -0.2436 | -0.7066 | 0.752 | 0.860761 | 1 | 8019 | tags=16%, list=1 |
| GO_U5_SNI | GO_U5_SNRNP | 22 | -0.3009 | -0.7765 | 0.752 | 0.789889 | 1 | 8662 | tags=27%, list=1 |
| GO_ARACH | GO_ARACHIDONIC_A | 16 | -0.3015 | -0.7581 | 0.7524 | 0.808446 | 1 | 16530 | tags=44%, list=2 |
| GO_ACID_T | GO_ACID_THIOL_LIG | 30 | -0.2397 | -0.7764 | 0.7529 | 0.789535 | 1 | 2881 | tags=10%, list=5 |
| GO_CHONE | GO_CHONDROCYTE_ | 17 | -0.2833 | -0.7661 | 0.7531 | 0.800093 | 1 | 9810 | tags=24%, list=1 |
| GO_OLIGOS | GO_OLIGOSACCHAR | 21 | -0.272 | -0.7243 | 0.7535 | 0.842258 | 1 | 15915 | tags=33%, list=2 |
| GO_VESICI | GO_VESICLE_DOCKI | 44 | -0.2388 | -0.7835 | 0.7553 | 0.781019 | 1 | 17970 | tags=45%, list=3 |
| GO_NUCLE | GO_NUCLEAR_EXPO | 202 | -0.2227 | -0.7257 | 0.7558 | 0.840922 | 1 | 10044 | tags=23%, list=1 |
| GO_DNA_S | GO_DNA_SECONDAR | 33 | -0.2632 | -0.7181 | 0.7564 | 0.847858 | 1 | 12831 | tags=36%, list=2 |
| GO_GDP_D | GO_GDP_DISSOCIATI | 16 | -0.2842 | -0.7549 | 0.7571 | 0.810605 | 1 | 4769 | tags=19%, list=8 |
| GO_METHY | GO_METHYLTRANSF | 102 | -0.2372 | -0.722 | 0.7578 | 0.843609 | 1 | 16569 | tags=39%, list=2 |
| GO_RESPO | GO_RESPONSE_TO_A | 47 | -0.2382 | -0.8105 | 0.7578 | 0.746619 | 1 | 7789 | tags=19%, list=1 |
| GO_OUTER | GO_OUTER_MITOCH | 21 | -0.2878 | -0.7363 | 0.7612 | 0.82971 | 1 | 17033 | tags=38%, list=2 |
| GO_LONG_ | GO_LONG_CHAIN_FA | 73 | -0.2232 | -0.8322 | 0.7621 | 0.721138 | 1 | 16156 | tags=33%, list=2 |
| GO_MONO | GO_MONOCARBOXY | 59 | -0.229 | -0.8518 | 0.7626 | 0.697491 | 1 | 7107 | tags=19%, list=1 |
| GO_POSITI | GO_POSITIVE_REGUI | 23 | -0.2664 | -0.7639 | 0.7646 | 0.802295 | 1 | 2644 | tags=13%, list=5 |
| GO_PROTEI | GO_PROTEIN_QUALI | 28 | -0.2494 | -0.7743 | 0.7648 | 0.791018 | 1 | 15109 | tags=32%, list=2 |
| GO_NURD_ | GO_NURD_COMPLEX | 15 | -0.302 | -0.7158 | 0.7648 | 0.848916 | 1 | 6622 | tags=27%, list=1 |
| GO_NEGAT | GO_NEGATIVE_REGU | 18 | -0.2624 | -0.7627 | 0.7655 | 0.803119 | 1 | 6677 | tags=17%, list=1 |
| GO_SISTER | GO_SISTER_CHROMA | 187 | -0.2025 | -0.6275 | 0.7666 | 0.930279 | 1 | 20886 | tags=40%, list=3 |
| GO_CHROM | GO_CHROMOSOMAL | 314 | -0.2157 | -0.6657 | 0.7669 | 0.901264 | 1 | 11713 | tags=25%, list=2 |
| GO_3_5_DN | GO_3_5_DNA_HELICA | 18 | -0.2944 | -0.6902 | 0.767 | 0.879163 | 1 | 8399 | tags=22%, list=1 |
| GO_RNA_S | GO_RNA_STABILIZAT | 49 | -0.2547 | -0.7369 | 0.7701 | 0.829381 | 1 | 14561 | tags=29%, list=2 |
| GO_MONO | GO_MONOVALENT_I | 26 | -0.2691 | -0.7947 | 0.771 | 0.766557 | 1 | 9880 | tags=35%, list=1 |
| GO_CENTR | GO_CENTROSOME_S | 15 | -0.3016 | -0.71 | 0.7722 | 0.856371 | 1 | 23739 | tags=60%, list=4 |
| GO_FATTY_ | GO_FATTY_ACID_BE | 73 | -0.2248 | -0.7651 | 0.7763 | 0.801279 | 1 | 9927 | tags=22%, list=1 |
| GO_NEGAT | GO_NEGATIVE_REGU | 29 | -0.2445 | -0.7871 | 0.7776 | 0.776619 | 1 | 8823 | tags=21%, list=1 |
| GO_GOLGI_ | GO_GOLGI_CIS_CIST | 29 | -0.2461 | -0.7623 | 0.7815 | 0.80342 | 1 | 4642 | tags=10%, list=8 |
| GO_BRUSH | GO_BRUSH_BORDER | 58 | -0.2373 | -0.7931 | 0.782 | 0.768426 | 1 | 24481 | tags=52%, list=4 |
| GO_OLIGOS | GO_OLIGOSACCHAR | 15 | -0.3228 | -0.7511 | 0.7822 | 0.814525 | 1 | 8442 | tags=33%, list=1 |
| GO_CYCLIN | GO_CYCLIN_BINDIN | 29 | -0.2427 | -0.7598 | 0.7826 | 0.806531 | 1 | 17539 | tags=41%, list=3 |
| GO_SULFA | GO_SULFATION | 18 | -0.2676 | -0.757 | 0.7826 | 0.809858 | 1 | 14920 | tags=33%, list=2 |

| | | | | | | | | | |
|---|---|---|---|---|---|---|---|---|---|
| GO_SPINDL | GO_SPINDLE_POLE | 160 | -0.2108 | -0.6868 | 0.7829 | 0.88199 | 1 | 11153 | tags=21%, list=1 |
| GO_HEMAT | GO_HEMATOPOIETIC | 26 | -0.2506 | -0.7782 | 0.7849 | 0.788052 | 1 | 10943 | tags=23%, list=1 |
| GO_POSITI | GO_POSITIVE_REGUI | 23 | -0.2442 | -0.7874 | 0.785 | 0.776419 | 1 | 21972 | tags=52%, list=3 |
| GO_NUCLE | GO_NUCLEOSOME_A | 71 | -0.2437 | -0.7007 | 0.7864 | 0.866844 | 1 | 8867 | tags=21%, list=1 |
| GO_PROTEI | GO_PROTEIN_LIPID_ | 34 | -0.2509 | -0.7634 | 0.7864 | 0.802487 | 1 | 16638 | tags=35%, list=2 |
| GO_POSITI | GO_POSITIVE_REGUI | 35 | -0.2398 | -0.734 | 0.7865 | 0.831357 | 1 | 17193 | tags=37%, list=2 |
| GO_REGUL | GO_REGULATION_OI | 33 | -0.2407 | -0.7718 | 0.7871 | 0.793607 | 1 | 8941 | tags=24%, list=1 |
| GO_REGUL | GO_REGULATION_OI | 23 | -0.2518 | -0.7327 | 0.7879 | 0.832773 | 1 | 9118 | tags=22%, list=1 |
| GO_PHOTO | GO_PHOTOTRANSDU | 34 | -0.2397 | -0.8099 | 0.7881 | 0.747361 | 1 | 2640 | tags=12%, list=5 |
| GO_SMALL | GO_SMALL_NUCLEO | 29 | -0.2672 | -0.7253 | 0.7885 | 0.841139 | 1 | 9800 | tags=28%, list=1 |
| GO_RESPIR | GO_RESPIRATORY_C | 19 | -0.2847 | -0.7001 | 0.7889 | 0.867349 | 1 | 9949 | tags=32%, list=1 |
| GO_MITOTI | GO_MITOTIC_G2_M_ | 29 | -0.2487 | -0.6873 | 0.7894 | 0.881714 | 1 | 18515 | tags=41%, list=3 |
| GO_REGUL | GO_REGULATION_OI | 16 | -0.2663 | -0.7479 | 0.7901 | 0.818758 | 1 | 10444 | tags=25%, list=1 |
| GO_DNA_M | GO_DNA_MODIFICAT | 116 | -0.2118 | -0.7566 | 0.7902 | 0.809572 | 1 | 16537 | tags=31%, list=2 |
| GO_RESPOI | GO_RESPONSE_TO_L | 23 | -0.2621 | -0.7757 | 0.7905 | 0.790168 | 1 | 26232 | tags=65%, list=4 |
| GO_ACID_S | GO_ACID_SECRETIOI | 43 | -0.2316 | -0.8182 | 0.7918 | 0.736697 | 1 | 8032 | tags=23%, list=1 |
| GO_NEGAT | GO_NEGATIVE_REGU | 22 | -0.2789 | -0.6659 | 0.7923 | 0.901347 | 1 | 5527 | tags=14%, list=9 |
| GO_ER_NU | GO_ER_NUCLEUS_SI | 50 | -0.227 | -0.7475 | 0.7926 | 0.818979 | 1 | 4873 | tags=16%, list=8 |
| GO_TRNA_ | GO_TRNA_METHYLA | 37 | -0.2495 | -0.7359 | 0.7938 | 0.829303 | 1 | 16653 | tags=38%, list=2 |
| GO_EXCITA | GO_EXCITATORY_EX | 30 | -0.2592 | -0.7811 | 0.7946 | 0.784252 | 1 | 16720 | tags=47%, list=2 |
| GO_TRNA_ | GO_TRNA_MODIFICA | 83 | -0.2141 | -0.7012 | 0.7951 | 0.866532 | 1 | 16653 | tags=31%, list=2 |
| GO_LIPID_ | GO_LIPID_OXIDATIO | 110 | -0.2058 | -0.7629 | 0.7957 | 0.803076 | 1 | 13129 | tags=25%, list=2 |
| GO_PROTEI | GO_PROTEIN_TARGE | 22 | -0.2429 | -0.7024 | 0.7965 | 0.865296 | 1 | 15043 | tags=32%, list=2 |
| GO_REGUL | GO_REGULATION_OI | 28 | -0.2545 | -0.8129 | 0.7972 | 0.743658 | 1 | 20196 | tags=50%, list=3 |
| GO_REGUL | GO_REGULATION_OI | 17 | -0.2573 | -0.7156 | 0.7977 | 0.849013 | 1 | 9118 | tags=24%, list=1 |
| GO_G_PRO | GO_G_PROTEIN_COU | 15 | -0.2938 | -0.7193 | 0.7984 | 0.846674 | 1 | 16241 | tags=27%, list=2 |
| GO_REGUL | GO_REGULATION_OI | 37 | -0.2337 | -0.7195 | 0.7984 | 0.846737 | 1 | 12024 | tags=27%, list=2 |
| GO_PARAS | GO_PARASYMPATHE | 19 | -0.263 | -0.7697 | 0.7996 | 0.796004 | 1 | 14543 | tags=42%, list=2 |
| GO_POSITI | GO_POSITIVE_REGUI | 68 | -0.2121 | -0.7755 | 0.8008 | 0.79022 | 1 | 18604 | tags=35%, list=3 |
| GO_NEGAT | GO_NEGATIVE_REGU | 22 | -0.2728 | -0.7683 | 0.8019 | 0.797974 | 1 | 20847 | tags=36%, list=3 |
| GO_SPINDL | GO_SPINDLE_MICRO | 64 | -0.2303 | -0.6526 | 0.8028 | 0.910967 | 1 | 22327 | tags=39%, list=3 |
| GO_MITOC | GO_MITOCHONDRIA | 23 | -0.2606 | -0.7231 | 0.8049 | 0.842866 | 1 | 1196 | tags=13%, list=2 |
| GO_REGUL | GO_REGULATION_OI | 25 | -0.2522 | -0.7432 | 0.8053 | 0.824262 | 1 | 24735 | tags=48%, list=4 |
| GO_MRNA_ | GO_MRNA_CLEAVAC | 18 | -0.291 | -0.7195 | 0.8059 | 0.846974 | 1 | 20353 | tags=39%, list=3 |
| GO_INTERM | GO_INTERMEMBRAN | 41 | -0.2256 | -0.7765 | 0.8079 | 0.789701 | 1 | 10316 | tags=22%, list=1 |
| GO_POSITI | GO_POSITIVE_REGUI | 41 | -0.2343 | -0.7996 | 0.8083 | 0.760215 | 1 | 12950 | tags=34%, list=2 |
| GO_C21_ST | GO_C21_STEROID_H | 38 | -0.2246 | -0.7819 | 0.8091 | 0.783233 | 1 | 7872 | tags=18%, list=1 |
| GO_SENSOI | GO_SENSORY_PERCI | 446 | -0.2223 | -0.7984 | 0.8092 | 0.761462 | 1 | 27389 | tags=47%, list=4 |
| GO_ERBB2_ | GO_ERBB2_SIGNALII | 31 | -0.2226 | -0.7374 | 0.8123 | 0.829449 | 1 | 19903 | tags=39%, list=3 |
| GO_REGUL | GO_REGULATION_OI | 46 | -0.2309 | -0.6771 | 0.8127 | 0.892191 | 1 | 7890 | tags=22%, list=1 |
| GO_NEGAT | GO_NEGATIVE_REGU | 31 | -0.2671 | -0.6763 | 0.8127 | 0.892801 | 1 | 7690 | tags=19%, list=1 |
| GO_SULFO | GO_SULFOTRANSFEI | 52 | -0.2246 | -0.7752 | 0.8134 | 0.790017 | 1 | 15562 | tags=33%, list=2 |
| GO_LYSOPI | GO_LYSOPHOSPHOL | 25 | -0.2449 | -0.7511 | 0.8135 | 0.814255 | 1 | 21985 | tags=52%, list=3 |
| GO_AU_RIC | GO_AU_RICH_ELEMI | 27 | -0.2492 | -0.7161 | 0.814 | 0.84917 | 1 | 16310 | tags=44%, list=2 |
| GO_MITOTI | GO_MITOTIC_SPINDI | 30 | -0.2492 | -0.6728 | 0.8151 | 0.895933 | 1 | 9173 | tags=23%, list=1 |
| GO_REGUL | GO_REGULATION_OI | 49 | -0.2337 | -0.7227 | 0.8152 | 0.842843 | 1 | 17322 | tags=37%, list=3 |
| GO_REGUL | GO_REGULATION_OI | 19 | -0.2497 | -0.7425 | 0.8193 | 0.824499 | 1 | 5730 | tags=21%, list=1 |
| GO_ZYMOG | GO_ZYMOGEN_GRAI | 15 | -0.2748 | -0.7374 | 0.8195 | 0.829262 | 1 | 17970 | tags=47%, list=3 |
| GO_HISTON | GO_HISTONE_H3_DE | 20 | -0.2547 | -0.7191 | 0.8205 | 0.846671 | 1 | 6062 | tags=20%, list=1 |
| GO_PHOSPI | GO_PHOSPHATIDYL | 38 | -0.2291 | -0.7842 | 0.8209 | 0.780462 | 1 | 11054 | tags=24%, list=1 |
| GO_POSITI | GO_POSITIVE_REGUI | 33 | -0.2391 | -0.753 | 0.8227 | 0.812424 | 1 | 12065 | tags=30%, list=2 |
| GO_DNA_T | GO_DNA_TEMPLATE | 74 | -0.2168 | -0.6463 | 0.8295 | 0.916151 | 1 | 7203 | tags=16%, list=1 |
| GO_OLFAC | GO_OLFACTORY_RE | 421 | -0.2231 | -0.7743 | 0.8307 | 0.790777 | 1 | 27336 | tags=47%, list=4 |
| GO_PROTEI | GO_PROTEIN_TRANS | 59 | -0.2103 | -0.7288 | 0.8333 | 0.837965 | 1 | 16133 | tags=31%, list=2 |
| GO_CHROM | GO_CHROMOCENTEI | 15 | -0.278 | -0.6715 | 0.8333 | 0.896833 | 1 | 4311 | tags=20%, list=7 |
| GO_GENER | GO_GENERAL_TRAN | 44 | -0.2169 | -0.671 | 0.8343 | 0.89679 | 1 | 10622 | tags=23%, list=1 |
| GO_CYTOP | GO_CYTOPLASMIC_I | 70 | -0.2095 | -0.7178 | 0.8353 | 0.847889 | 1 | 14125 | tags=26%, list=2 |
| GO_SULFUI | GO_SULFUR_COMPO | 42 | -0.2325 | -0.7537 | 0.8356 | 0.811651 | 1 | 7781 | tags=19%, list=1 |

| | | | | | | | | | |
|---|---|---|---|---|---|---|---|---|---|
| GO_DOSAG | GO_DOSAGE_COMPE | 17 | -0.26 | -0.6579 | 0.8369 | 0.906973 | 1 | 26262 | tags=59%, list=4 |
| GO_MRNA_ | GO_MRNA_CLEAVAC | 22 | -0.2591 | -0.6619 | 0.8376 | 0.904171 | 1 | 21770 | tags=41%, list=3 |
| GO_MAINT | GO_MAINTENANCE_ | 19 | -0.2574 | -0.7425 | 0.8377 | 0.824736 | 1 | 10518 | tags=21%, list=1 |
| GO_CARBO | GO_CARBOHYDRATI | 22 | -0.24 | -0.7277 | 0.839 | 0.838571 | 1 | 8443 | tags=23%, list=1 |
| GO_REGUL | GO_REGULATION_OI | 48 | -0.2222 | -0.7559 | 0.8391 | 0.809929 | 1 | 19410 | tags=38%, list=3 |
| GO_ESTRO | GO_ESTROGEN_MET | 32 | -0.2354 | -0.7154 | 0.8406 | 0.84891 | 1 | 11913 | tags=25%, list=2 |
| GO_BRUSH | GO_BRUSH_BORDER | 106 | -0.2152 | -0.7409 | 0.8437 | 0.825308 | 1 | 13050 | tags=25%, list=2 |
| GO_NEGAT | GO_NEGATIVE_REGU | 26 | -0.236 | -0.7338 | 0.8444 | 0.831442 | 1 | 17153 | tags=38%, list=2 |
| GO_INTRA | GO_INTRACELLULAI | 29 | -0.235 | -0.736 | 0.8449 | 0.829474 | 1 | 20068 | tags=41%, list=3 |
| GO_MALE_ | GO_MALE_SEX_DETI | 15 | -0.2626 | -0.7234 | 0.8454 | 0.842695 | 1 | 10388 | tags=27%, list=1 |
| GO_CELLU | GO_CELLULAR_HOR | 132 | -0.1985 | -0.7892 | 0.8473 | 0.773952 | 1 | 15827 | tags=30%, list=2 |
| GO_RETIN | GO_RETINAL_METAI | 15 | -0.2662 | -0.703 | 0.8477 | 0.865009 | 1 | 15827 | tags=33%, list=2 |
| GO_ICOSAI | GO_ICOSANOID_REC | 15 | -0.2629 | -0.6986 | 0.8479 | 0.868828 | 1 | 4644 | tags=20%, list=8 |
| GO_NEURO | GO_NEUROPEPTIDE_ | 30 | -0.2587 | -0.7415 | 0.8494 | 0.824718 | 1 | 18674 | tags=30%, list=3 |
| GO_PROTEI | GO_PROTEIN_LOCAL | 74 | -0.2132 | -0.6124 | 0.8499 | 0.94118 | 1 | 10459 | tags=23%, list=1 |
| GO_QUATE | GO_QUATERNARY_A | 28 | -0.2421 | -0.7465 | 0.8521 | 0.819688 | 1 | 19238 | tags=43%, list=3 |
| GO_RHODO | GO_RHODOPSIN_ME | 29 | -0.2346 | -0.755 | 0.8528 | 0.810728 | 1 | 2042 | tags=10%, list=3 |
| GO_TBP_CI | GO_TBP_CLASS_PRO | 26 | -0.2369 | -0.6716 | 0.8539 | 0.89707 | 1 | 10197 | tags=23%, list=1 |
| GO_TRIGLY | GO_TRIGLYCERIDE_ | 41 | -0.2224 | -0.7287 | 0.856 | 0.837698 | 1 | 1653 | tags=7%, list=3% |
| GO_CHROM | GO_CHROMOSOME_ | 32 | -0.2226 | -0.6097 | 0.8563 | 0.942202 | 1 | 21505 | tags=34%, list=3 |
| GO_SIGNAI | GO_SIGNALING_REC | 32 | -0.2267 | -0.6848 | 0.86 | 0.884207 | 1 | 4232 | tags=13%, list=7 |
| GO_REGUL | GO_REGULATION_OI | 40 | -0.2138 | -0.6899 | 0.8607 | 0.879239 | 1 | 20646 | tags=45%, list=3 |
| GO_MITOTI | GO_MITOTIC_CHRON | 15 | -0.2567 | -0.5941 | 0.8615 | 0.951751 | 1 | 23311 | tags=40%, list=4 |
| GO_TELOM | GO_TELOMERASE_H | 21 | -0.2465 | -0.6357 | 0.8621 | 0.923822 | 1 | 8833 | tags=19%, list=1 |
| GO_BINDIN | GO_BINDING_OF_SPI | 43 | -0.2124 | -0.7341 | 0.8635 | 0.831462 | 1 | 24643 | tags=49%, list=4 |
| GO_VASCU | GO_VASCULAR_END | 63 | -0.2049 | -0.7371 | 0.8657 | 0.829383 | 1 | 18245 | tags=35%, list=3 |
| GO_RIBOS | GO_RIBOSOMAL_LAI | 29 | -0.2307 | -0.5912 | 0.869 | 0.95298 | 1 | 16922 | tags=34%, list=2 |
| GO_ARACH | GO_ARACHIDONIC_ | 20 | -0.2509 | -0.6701 | 0.8715 | 0.89749 | 1 | 16530 | tags=40%, list=2 |
| GO_STEROI | GO_STEROID_HORM | 26 | -0.231 | -0.7241 | 0.8717 | 0.842329 | 1 | 20810 | tags=50%, list=3 |
| GO_MALE_ | GO_MALE_MEIOTIC_ | 45 | -0.2242 | -0.7169 | 0.8725 | 0.848349 | 1 | 9845 | tags=22%, list=1 |
| GO_STABII | GO_STABILIZATION_ | 17 | -0.2616 | -0.7159 | 0.8735 | 0.849171 | 1 | 20717 | tags=47%, list=3 |
| GO_GLYCO | GO_GLYCOSIDE_ME | 21 | -0.2367 | -0.6715 | 0.8753 | 0.896564 | 1 | 4812 | tags=14%, list=8 |
| GO_STRUC | GO_STRUCTURAL_C | 23 | -0.2436 | -0.7039 | 0.8767 | 0.864194 | 1 | 22190 | tags=43%, list=3 |
| GO_CYTOC | GO_CYTOCHROME_ | 33 | -0.2216 | -0.5933 | 0.8768 | 0.951919 | 1 | 11858 | tags=27%, list=2 |
| GO_MITOC | GO_MITOCHONDRIA | 42 | -0.2092 | -0.6114 | 0.8783 | 0.941661 | 1 | 20105 | tags=38%, list=3 |
| GO_RRNA_ | GO_RRNA_CONTAINI | 15 | -0.2478 | -0.6147 | 0.8819 | 0.939983 | 1 | 18453 | tags=47%, list=3 |
| GO_PROTEI | GO_PROTEIN_LOCAL | 18 | -0.2349 | -0.6677 | 0.8825 | 0.899557 | 1 | 8830 | tags=22%, list=1 |
| GO_POSITI | GO_POSITIVE_REGUI | 67 | -0.1997 | -0.6742 | 0.8838 | 0.894689 | 1 | 14975 | tags=28%, list=2 |
| GO_CELL_I | GO_CELL_DEATH_IN | 33 | -0.2178 | -0.6986 | 0.8844 | 0.869028 | 1 | 20847 | tags=36%, list=3 |
| GO_SEQUE | GO_SEQUESTERING_ | 17 | -0.2249 | -0.6444 | 0.8845 | 0.917054 | 1 | 9653 | tags=24%, list=1 |
| GO_PROTEI | GO_PROTEIN_PHOSP | 18 | -0.2367 | -0.6648 | 0.8948 | 0.901642 | 1 | 14849 | tags=33%, list=2 |
| GO_RESPOI | GO_RESPONSE_TO_M | 18 | -0.2463 | -0.6792 | 0.8953 | 0.88984 | 1 | 7373 | tags=22%, list=1 |
| GO_CHANN | GO_CHANNEL_INHIB | 37 | -0.2078 | -0.6824 | 0.896 | 0.886525 | 1 | 6828 | tags=16%, list=1 |
| GO_INNER_ | GO_INNER_EAR_REC | 27 | -0.2482 | -0.689 | 0.8967 | 0.880158 | 1 | 18458 | tags=37%, list=3 |
| GO_ANCHO | GO_ANCHORED_COM | 19 | -0.24 | -0.6554 | 0.8983 | 0.908972 | 1 | 1000 | tags=11%, list=2 |
| GO_REGUL | GO_REGULATION_OI | 51 | -0.1976 | -0.6599 | 0.9022 | 0.905668 | 1 | 4751 | tags=12%, list=8 |
| GO_PRIMAI | GO_PRIMARY_ALCO | 16 | -0.2345 | -0.6508 | 0.9028 | 0.912203 | 1 | 14920 | tags=31%, list=2 |
| GO_SECON | GO_SECONDARY_ME | 24 | -0.2239 | -0.7174 | 0.9029 | 0.847884 | 1 | 18645 | tags=42%, list=3 |
| GO_ACETY | GO_ACETYLCHOLINI | 16 | -0.2589 | -0.6578 | 0.9059 | 0.906748 | 1 | 14794 | tags=38%, list=2 |
| GO_CENTR | GO_CENTROMERE_C | 37 | -0.2011 | -0.5043 | 0.9082 | 0.986492 | 1 | 8549 | tags=16%, list=1 |
| GO_DRUG_ | GO_DRUG_CATABOL | 26 | -0.2258 | -0.6611 | 0.9091 | 0.904697 | 1 | 19093 | tags=38%, list=3 |
| GO_SYNAP | GO_SYNAPTIC_TRAN | 26 | -0.2207 | -0.6653 | 0.9113 | 0.901318 | 1 | 6123 | tags=15%, list=1 |
| GO_LAMEL | GO_LAMELLIPODIUN | 22 | -0.2184 | -0.5993 | 0.9122 | 0.949146 | 1 | 11877 | tags=27%, list=2 |
| GO_MEMBI | GO_MEMBRANE_DIS | 16 | -0.2349 | -0.5791 | 0.9122 | 0.960107 | 1 | 22327 | tags=38%, list=3 |
| GO_SENSOI | GO_SENSORY_PERCI | 23 | -0.2344 | -0.6847 | 0.9153 | 0.88403 | 1 | 19156 | tags=39%, list=3 |
| GO_SKIN_I | GO_SKIN_DEVELOPN | 420 | -0.2073 | -0.6884 | 0.9198 | 0.880567 | 1 | 16432 | tags=33%, list=2 |
| GO_POSITI | GO_POSITIVE_REGUI | 30 | -0.1992 | -0.5249 | 0.9202 | 0.981482 | 1 | 18223 | tags=33%, list=3 |
| GO_TRANS | GO_TRANSCRIPTION | 36 | -0.2074 | -0.6143 | 0.921 | 0.939951 | 1 | 13219 | tags=25%, list=2 |

| | | | | | | | | | |
|---|---|---|---|---|---|---|---|---|---|
| GO_HEPAT | GO_HEPATOCYTE_D | 15 | -0.2501 | -0.6454 | 0.9229 | 0.916696 | 1 | 11335 | tags=27%, list=1 |
| GO_NEGAT | GO_NEGATIVE_REGU | 78 | -0.1821 | -0.6935 | 0.9258 | 0.875233 | 1 | 11813 | tags=19%, list=2 |
| GO_KERAT | GO_KERATIN_FILAM | 94 | -0.2793 | -0.6622 | 0.9269 | 0.904088 | 1 | 22704 | tags=55%, list=3 |
| GO_LOW_D | GO_LOW_DENSITY_I | 17 | -0.2323 | -0.6078 | 0.9273 | 0.94329 | 1 | 18948 | tags=35%, list=3 |
| GO_DETEC | GO_DETECTION_OF_ | 23 | -0.2287 | -0.6537 | 0.9302 | 0.910426 | 1 | 19156 | tags=48%, list=3 |
| GO_ACETY | GO_ACETYLCHOLINI | 16 | -0.2457 | -0.6244 | 0.9315 | 0.932647 | 1 | 8287 | tags=25%, list=1 |
| GO_NEGAT | GO_NEGATIVE_REGU | 21 | -0.2104 | -0.6382 | 0.9341 | 0.922398 | 1 | 6062 | tags=10%, list=1 |
| GO_CELLU | GO_CELLULAR_COM | 36 | -0.1916 | -0.6423 | 0.9342 | 0.918874 | 1 | 22533 | tags=44%, list=3 |
| GO_PHOTO | GO_PHOTORECEPTO | 17 | -0.2153 | -0.6197 | 0.9368 | 0.936295 | 1 | 14223 | tags=24%, list=2 |
| GO_INTRIN | GO_INTRINSIC_COM | 64 | -0.1834 | -0.6497 | 0.938 | 0.913057 | 1 | 10565 | tags=19%, list=1 |
| GO_DETEC | GO_DETECTION_OF_ | 24 | -0.2306 | -0.6692 | 0.9382 | 0.898127 | 1 | 7971 | tags=21%, list=1 |
| GO_INO80_ | GO_INO80_TYPE_CO | 25 | -0.1941 | -0.5361 | 0.94 | 0.978781 | 1 | 6344 | tags=12%, list=1 |
| GO_REGUL | GO_REGULATION_OI | 23 | -0.2113 | -0.6379 | 0.9409 | 0.92209 | 1 | 24735 | tags=39%, list=4 |
| GO_POSITI | GO_POSITIVE_REGU | 54 | -0.1884 | -0.6381 | 0.9415 | 0.92225 | 1 | 18604 | tags=33%, list=3 |
| GO_FATTY_ | GO_FATTY_ACID_LIG | 22 | -0.2131 | -0.6279 | 0.9472 | 0.930199 | 1 | 2881 | tags=9%, list=5% |
| GO_EPITHE | GO_EPITHELIAL_CEI | 15 | -0.2211 | -0.6108 | 0.9519 | 0.941707 | 1 | 9937 | tags=20%, list=1 |
| GO_CELLU | GO_CELLULAR_ALDI | 62 | -0.1845 | -0.6589 | 0.9525 | 0.906463 | 1 | 15827 | tags=26%, list=2 |
| GO_EPIDEF | GO_EPIDERMAL_CEI | 358 | -0.2119 | -0.6589 | 0.9543 | 0.90617 | 1 | 16442 | tags=35%, list=2 |
| GO_NEGAT | GO_NEGATIVE_REGU | 37 | -0.1985 | -0.653 | 0.9572 | 0.910846 | 1 | 13692 | tags=27%, list=2 |
| GO_GLUCO | GO_GLUCOSYLTRAN | 16 | -0.2033 | -0.5618 | 0.9582 | 0.968273 | 1 | 22571 | tags=44%, list=3 |
| GO_POTAS | GO_POTASSIUM_ION | 16 | -0.2237 | -0.5979 | 0.9603 | 0.949764 | 1 | 20717 | tags=50%, list=3 |
| GO_GLUCO | GO_GLUCOCORTICO | 26 | -0.2008 | -0.6334 | 0.9608 | 0.925586 | 1 | 22074 | tags=46%, list=3 |
| GO_CATEC | GO_CATECHOLAMIN | 17 | -0.2117 | -0.5978 | 0.9685 | 0.94951 | 1 | 18325 | tags=35%, list=3 |
| GO_DRUG_ | GO_DRUG_METABOI | 46 | -0.1913 | -0.6519 | 0.9734 | 0.911388 | 1 | 19093 | tags=35%, list=3 |
| GO_REGUL | GO_REGULATION_OI | 18 | -0.2067 | -0.526 | 0.9754 | 0.981487 | 1 | 27396 | tags=61%, list=4 |
| GO_REGUL | GO_REGULATION_OI | 25 | -0.1915 | -0.5131 | 0.9791 | 0.984655 | 1 | 1992 | tags=8%, list=3% |
| GO_LONG_ | GO_LONG_CHAIN_FA | 33 | -0.1714 | -0.5741 | 0.9861 | 0.96259 | 1 | 27131 | tags=52%, list=4 |
| GO_KERAT | GO_KERATINIZATIOI | 223 | -0.2249 | -0.5738 | 0.9865 | 0.962372 | 1 | 22704 | tags=49%, list=3 |
| GO_POSITI | GO_POSITIVE_REGU | 15 | -0.1832 | -0.4832 | 0.9867 | 0.990684 | 1 | 2375 | tags=7%, list=4% |
| GO_INTERM | GO_INTERMEDIATE_ | 239 | -0.2558 | -0.6745 | 0.988 | 0.894575 | 1 | 19653 | tags=48%, list=3 |
| GO_SPERM | GO_SPERM_EGG_RE( | 60 | -0.1757 | -0.6453 | 0.9885 | 0.916481 | 1 | 10826 | tags=18%, list=1 |
| GO_KERAT | GO_KERATINOCYTE_ | 301 | -0.1833 | -0.5344 | 0.9981 | 0.979031 | 1 | 16432 | tags=33%, list=2 |

5%, signal=61%
2%, signal=67%
3%, signal=67%
6%, signal=55%
2%, signal=61%
2%, signal=64%
5%, signal=57%
5%, signal=59%
2%, signal=61%
3%, signal=81%
3%, signal=54%
2%, signal=62%
7%, signal=80%
2%, signal=57%
2%, signal=68%
2%, signal=39%
6%, signal=89%
5%, signal=47%
2%, signal=58%
3%, signal=78%
1%, signal=83%
6%, signal=50%
3%, signal=80%
5%, signal=54%
%, signal=41%
5%, signal=59%
1%, signal=68%
6%, signal=43%
1%, signal=61%
0%, signal=63%
2%, signal=54%
7%, signal=62%
5%, signal=44%
6%, signal=39%
2%, signal=45%
1%, signal=67%
9%, signal=57%
4%, signal=56%
1%, signal=64%
4%, signal=45%
%, signal=46%
1%, signal=60%
1%, signal=63%
2%, signal=82%
7%, signal=52%
2%, signal=59%
5%, signal=47%
6%, signal=70%
1%, signal=63%
5%, signal=64%
1%, signal=83%
1%, signal=65%
2%, signal=93%
2%, signal=102%
1%, signal=93%
6%, signal=45%
7%, signal=46%

4%, signal=57%
2%, signal=53%
1%, signal=47%
6%, signal=67%
5%, signal=65%
8%, signal=80%
1%, signal=93%
9%, signal=53%
%, signal=48%
1%, signal=63%
%, signal=43%
6%, signal=107%
5%, signal=57%
7%, signal=82%
%, signal=36%
4%, signal=51%
9%, signal=79%
0%, signal=73%
7%, signal=103%
5%, signal=65%
6%, signal=77%
3%, signal=46%
4%, signal=42%
0%, signal=50%
0%, signal=44%
2%, signal=40%
7%, signal=97%
1%, signal=91%
5%, signal=46%
%, signal=24%
4%, signal=47%
2%, signal=60%
1%, signal=80%
1%, signal=59%
2%, signal=51%
4%, signal=77%
7%, signal=45%
2%, signal=33%
3%, signal=36%
1%, signal=53%
1%, signal=74%
%, signal=48%
5%, signal=62%
2%, signal=56%
2%, signal=50%
4%, signal=35%
9%, signal=77%
3%, signal=60%
3%, signal=51%
5%, signal=47%
8%, signal=72%
6%, signal=62%
1%, signal=59%
2%, signal=74%
8%, signal=46%
9%, signal=41%
%, signal=51%
9%, signal=86%

3%, signal=40%
9%, signal=56%
4%, signal=61%
3%, signal=54%
2%, signal=53%
9%, signal=45%
6%, signal=66%
9%, signal=87%
2%, signal=51%
9%, signal=96%
5%, signal=45%
2%, signal=60%
8%, signal=56%
8%, signal=61%
9%, signal=52%
7%, signal=76%
6%, signal=73%
5%, signal=44%
7%, signal=63%
1%, signal=52%
4%, signal=43%
4%, signal=58%
%, signal=48%
9%, signal=64%
6%, signal=44%
0%, signal=52%
2%, signal=44%
1%, signal=93%
9%, signal=38%
6%, signal=69%
1%, signal=46%
1%, signal=65%
9%, signal=77%
5%, signal=61%
3%, signal=66%
8%, signal=46%
8%, signal=61%
3%, signal=55%
%, signal=48%
3%, signal=64%
3%, signal=64%
0%, signal=81%
0%, signal=55%
6%, signal=82%
2%, signal=80%
5%, signal=57%
9%, signal=65%
2%, signal=54%
%, signal=36%
4%, signal=45%
6%, signal=50%
5%, signal=41%
1%, signal=64%
3%, signal=47%
2%, signal=50%
6%, signal=41%
%, signal=46%
1%, signal=59%

3%, signal=51%
%, signal=41%
%, signal=42%
%, signal=24%
0%, signal=65%
6%, signal=47%
%, signal=34%
8%, signal=64%
1%, signal=71%
0%, signal=51%
1%, signal=91%
5%, signal=39%
4%, signal=52%
4%, signal=55%
5%, signal=40%
%, signal=23%
9%, signal=97%
0%, signal=52%
5%, signal=91%
8%, signal=87%
7%, signal=49%
7%, signal=59%
9%, signal=47%
2%, signal=56%
1%, signal=76%
3%, signal=36%
5%, signal=95%
4%, signal=39%
1%, signal=67%
7%, signal=68%
3%, signal=43%
0%, signal=44%
7%, signal=53%
6%, signal=68%
5%, signal=44%
4%, signal=46%
%, signal=40%
0%, signal=32%
0%, signal=56%
2%, signal=51%
5%, signal=95%
9%, signal=46%
3%, signal=48%
3%, signal=58%
8%, signal=54%
1%, signal=77%
5%, signal=56%
8%, signal=45%
2%, signal=48%
8%, signal=48%
3%, signal=58%
5%, signal=70%
9%, signal=49%
8%, signal=44%
8%, signal=61%
6%, signal=51%
9%, signal=58%
7%, signal=61%

2%, signal=36%
5%, signal=37%
%, signal=41%
1%, signal=44%
4%, signal=55%
8%, signal=52%
2%, signal=49%
0%, signal=35%
7%, signal=41%
4%, signal=39%
1%, signal=53%
0%, signal=32%
2%, signal=45%
9%, signal=53%
1%, signal=48%
0%, signal=26%
2%, signal=53%
%, signal=28%
5%, signal=70%
3%, signal=44%
0%, signal=46%
7%, signal=55%
6%, signal=46%
0%, signal=31%
3%, signal=35%
7%, signal=59%
6%, signal=72%
3%, signal=55%
7%, signal=60%
5%, signal=56%
9%, signal=56%
7%, signal=72%
9%, signal=69%
2%, signal=47%
7%, signal=60%
1%, signal=99%
3%, signal=111%
4%, signal=70%
4%, signal=41%
9%, signal=46%
9%, signal=41%
%, signal=35%
9%, signal=114%
1%, signal=77%
9%, signal=48%
2%, signal=31%
9%, signal=57%
8%, signal=60%
6%, signal=87%
6%, signal=38%
7%, signal=42%
5%, signal=41%
2%, signal=50%
6%, signal=101%
0%, signal=78%
8%, signal=62%
9%, signal=62%
8%, signal=75%

6%, signal=61%
1%, signal=52%
4%, signal=112%
4%, signal=54%
7%, signal=66%
6%, signal=60%
0%, signal=74%
6%, signal=65%
5%, signal=56%
%, signal=30%
9%, signal=62%
2%, signal=52%
2%, signal=74%
7%, signal=70%
5%, signal=41%
1%, signal=50%
%, signal=34%
5%, signal=38%
4%, signal=40%
1%, signal=73%
1%, signal=49%
9%, signal=47%
9%, signal=63%
2%, signal=55%
6%, signal=41%
5%, signal=89%
4%, signal=53%
7%, signal=60%
7%, signal=74%
4%, signal=35%
3%, signal=58%
0%, signal=26%
7%, signal=55%
2%, signal=38%
0%, signal=64%
4%, signal=54%
0%, signal=45%
0%, signal=44%
4%, signal=51%
4%, signal=35%
9%, signal=75%
%, signal=37%
7%, signal=66%
4%, signal=33%
5%, signal=45%
%, signal=33%
0%, signal=82%
0%, signal=55%
2%, signal=49%
4%, signal=55%
2%, signal=112%
7%, signal=59%
3%, signal=72%
1%, signal=48%
9%, signal=55%
7%, signal=57%
6%, signal=42%
0%, signal=44%

7%, signal=53%
7%, signal=55%
9%, signal=85%
4%, signal=104%
8%, signal=52%
4%, signal=101%
8%, signal=106%
9%, signal=72%
%, signal=33%
5%, signal=67%
4%, signal=42%
4%, signal=54%
2%, signal=85%
6%, signal=41%
7%, signal=52%
3%, signal=72%
0%, signal=70%
0%, signal=28%
0%, signal=46%
1%, signal=72%
5%, signal=43%
9%, signal=48%
2%, signal=29%
8%, signal=47%
4%, signal=56%
%, signal=23%
3%, signal=33%
8%, signal=48%
0%, signal=44%
2%, signal=63%
4%, signal=45%
9%, signal=47%
9%, signal=54%
8%, signal=42%
6%, signal=44%
7%, signal=39%
6%, signal=79%
6%, signal=54%
0%, signal=42%
4%, signal=59%
3%, signal=54%
8%, signal=59%
1%, signal=87%
5%, signal=52%
3%, signal=46%
3%, signal=45%
6%, signal=66%
9%, signal=54%
5%, signal=53%
9%, signal=44%
6%, signal=39%
5%, signal=108%
8%, signal=58%
1%, signal=93%
8%, signal=75%
2%, signal=50%
7%, signal=40%
9%, signal=39%

7%, signal=68%
5%, signal=60%
2%, signal=73%
0%, signal=28%
9%, signal=103%
7%, signal=73%
8%, signal=68%
3%, signal=35%
%, signal=36%
5%, signal=42%
1%, signal=74%
1%, signal=30%
1%, signal=42%
2%, signal=68%
9%, signal=46%
2%, signal=49%
0%, signal=51%
7%, signal=38%
3%, signal=50%
%, signal=28%
1%, signal=72%
6%, signal=62%
1%, signal=49%
4%, signal=58%
8%, signal=65%
9%, signal=61%
8%, signal=83%
4%, signal=55%
9%, signal=40%
5%, signal=66%
4%, signal=50%
0%, signal=47%
0%, signal=71%
2%, signal=57%
6%, signal=72%
4%, signal=56%
9%, signal=44%
0%, signal=30%
8%, signal=52%
%, signal=31%
1%, signal=45%
8%, signal=48%
0%, signal=43%
4%, signal=79%
5%, signal=74%
2%, signal=48%
3%, signal=87%
0%, signal=34%
6%, signal=38%
7%, signal=40%
3%, signal=76%
1%, signal=54%
9%, signal=36%
3%, signal=71%
4%, signal=32%
5%, signal=69%
4%, signal=95%
4%, signal=56%

7%, signal=84%
2%, signal=46%
6%, signal=36%
2%, signal=47%
4%, signal=31%
6%, signal=39%
7%, signal=57%
6%, signal=67%
2%, signal=41%
0%, signal=74%
6%, signal=37%
5%, signal=113%
8%, signal=76%
5%, signal=42%
3%, signal=90%
5%, signal=38%
1%, signal=71%
5%, signal=49%
0%, signal=59%
0%, signal=37%
3%, signal=53%
5%, signal=44%
5%, signal=50%
8%, signal=41%
0%, signal=32%
0%, signal=48%
1%, signal=80%
8%, signal=44%
8%, signal=43%
7%, signal=80%
0%, signal=35%
6%, signal=38%
7%, signal=46%
5%, signal=40%
3%, signal=59%
0%, signal=43%
9%, signal=57%
4%, signal=37%
8%, signal=63%
3%, signal=32%
8%, signal=69%
2%, signal=32%
5%, signal=44%
1%, signal=46%
2%, signal=31%
6%, signal=56%
3%, signal=75%
%, signal=27%
3%, signal=51%
5%, signal=39%
2%, signal=30%
1%, signal=45%
4%, signal=84%
1%, signal=40%
5%, signal=76%
1%, signal=129%
6%, signal=59%
8%, signal=40%

%, signal=28%
1%, signal=48%
8%, signal=40%
9%, signal=41%
1%, signal=61%
4%, signal=39%
2%, signal=46%
5%, signal=53%
1%, signal=42%
9%, signal=51%
3%, signal=44%
8%, signal=61%
8%, signal=42%
9%, signal=40%
9%, signal=57%
1%, signal=43%
9%, signal=73%
0%, signal=62%
9%, signal=60%
6%, signal=52%
2%, signal=43%
0%, signal=43%
5%, signal=48%
3%, signal=40%
9%, signal=48%
1%, signal=35%
2%, signal=36%
2%, signal=47%
2%, signal=48%
6%, signal=63%
5%, signal=36%
9%, signal=96%
9%, signal=40%
4%, signal=54%
9%, signal=46%
8%, signal=41%
0%, signal=36%
7%, signal=89%
0%, signal=140%
9%, signal=38%
7%, signal=98%
%, signal=27%
8%, signal=61%
4%, signal=33%
%, signal=39%
3%, signal=49%
8%, signal=83%
4%, signal=83%
9%, signal=43%
5%, signal=49%
4%, signal=71%
7%, signal=64%
4%, signal=75%
3%, signal=68%
6%, signal=63%
6%, signal=58%
5%, signal=59%
9%, signal=37%

2%, signal=61%
0%, signal=62%
3%, signal=33%
9%, signal=51%
7%, signal=52%
2%, signal=53%
3%, signal=42%
6%, signal=53%
7%, signal=37%
8%, signal=57%
8%, signal=77%
3%, signal=33%
5%, signal=63%
4%, signal=64%
0%, signal=42%
0%, signal=68%
9%, signal=50%
9%, signal=52%
8%, signal=64%
2%, signal=27%
8%, signal=55%
8%, signal=57%
9%, signal=43%
1%, signal=80%
7%, signal=40%
2%, signal=52%
3%, signal=37%
5%, signal=44%
3%, signal=42%
%, signal=25%
7%, signal=33%
3%, signal=56%
3%, signal=37%
%, signal=21%
7%, signal=42%
5%, signal=85%
1%, signal=45%
4%, signal=80%
%, signal=26%
3%, signal=84%
9%, signal=73%
2%, signal=48%
5%, signal=87%
0%, signal=61%
2%, signal=48%
2%, signal=74%
8%, signal=64%
1%, signal=84%
1%, signal=76%
0%, signal=43%
1%, signal=83%
5%, signal=81%
8%, signal=37%
6%, signal=43%
2%, signal=47%
7%, signal=38%
9%, signal=75%
0%, signal=62%

1%, signal=78%
6%, signal=83%
0%, signal=66%
7%, signal=44%
0%, signal=42%
6%, signal=58%
5%, signal=75%
1%, signal=70%
%, signal=23%
7%, signal=63%
0%, signal=45%
9%, signal=51%
7%, signal=59%
8%, signal=69%
0%, signal=35%
7%, signal=36%
0%, signal=56%
4%, signal=72%
8%, signal=81%
0%, signal=60%
9%, signal=39%
8%, signal=52%
6%, signal=55%
4%, signal=116%
7%, signal=47%
8%, signal=52%
3%, signal=87%
8%, signal=61%
3%, signal=74%
2%, signal=46%
9%, signal=66%
6%, signal=54%
4%, signal=89%
5%, signal=39%
4%, signal=72%
1%, signal=75%
6%, signal=78%
6%, signal=67%
9%, signal=118%
4%, signal=50%
0%, signal=42%
4%, signal=68%
9%, signal=39%
2%, signal=39%
9%, signal=42%
7%, signal=73%
%, signal=44%
1%, signal=33%
6%, signal=47%
5%, signal=71%
8%, signal=95%
4%, signal=45%
7%, signal=99%
5%, signal=62%
9%, signal=78%
5%, signal=70%
0%, signal=41%
1%, signal=89%

1%, signal=46%
5%, signal=32%
2%, signal=45%
%, signal=30%
0%, signal=34%
8%, signal=65%
7%, signal=44%
3%, signal=77%
3%, signal=50%
9%, signal=57%
6%, signal=54%
3%, signal=68%
7%, signal=69%
7%, signal=68%
3%, signal=44%
8%, signal=61%
5%, signal=55%
0%, signal=34%
7%, signal=37%
%, signal=34%
3%, signal=36%
7%, signal=40%
7%, signal=69%
8%, signal=90%
1%, signal=34%
3%, signal=38%
0%, signal=57%
4%, signal=32%
6%, signal=35%
7%, signal=70%
7%, signal=55%
0%, signal=36%
4%, signal=47%
8%, signal=56%
1%, signal=33%
%, signal=31%
5%, signal=36%
9%, signal=38%
8%, signal=85%
9%, signal=63%
8%, signal=74%
%, signal=29%
0%, signal=30%
5%, signal=90%
2%, signal=44%
3%, signal=40%
7%, signal=57%
8%, signal=39%
5%, signal=41%
6%, signal=41%
4%, signal=52%
%, signal=23%
2%, signal=92%
2%, signal=62%
0%, signal=67%
4%, signal=105%
1%, signal=87%
%, signal=20%

%, signal=27%
9%, signal=65%
9%, signal=42%
1%, signal=42%
1%, signal=48%
6%, signal=68%
4%, signal=112%
2%, signal=34%
8%, signal=55%
8%, signal=42%
8%, signal=44%
7%, signal=60%
0%, signal=30%
5%, signal=119%
3%, signal=60%
7%, signal=72%
1%, signal=55%
9%, signal=46%
7%, signal=61%
6%, signal=40%
4%, signal=51%
1%, signal=37%
4%, signal=51%
0%, signal=40%
2%, signal=49%
2%, signal=45%
4%, signal=48%
1%, signal=39%
7%, signal=51%
7%, signal=59%
3%, signal=28%
1%, signal=57%
5%, signal=38%
3%, signal=31%
4%, signal=39%
%, signal=22%
2%, signal=81%
6%, signal=34%
5%, signal=37%
1%, signal=27%
2%, signal=30%
5%, signal=49%
%, signal=26%
%, signal=28%
5%, signal=94%
5%, signal=59%
0%, signal=47%
6%, signal=29%
7%, signal=34%
6%, signal=48%
3%, signal=77%
9%, signal=47%
1%, signal=49%
3%, signal=55%
4%, signal=53%
1%, signal=42%
4%, signal=48%
6%, signal=40%

3%, signal=63%
8%, signal=39%
%, signal=28%
3%, signal=52%
4%, signal=40%
5%, signal=55%
9%, signal=51%
4%, signal=41%
0%, signal=49%
7%, signal=55%
%, signal=25%
9%, signal=64%
3%, signal=63%
2%, signal=77%
9%, signal=41%
0%, signal=42%
7%, signal=56%
4%, signal=52%
0%, signal=66%
0%, signal=28%
9%, signal=45%
1%, signal=51%
1%, signal=26%
8%, signal=39%
8%, signal=56%
7%, signal=42%
3%, signal=71%
6%, signal=63%
1%, signal=42%
0%, signal=55%
1%, signal=62%
2%, signal=57%
9%, signal=39%
6%, signal=45%
3%, signal=73%
8%, signal=87%
3%, signal=34%
2%, signal=52%
8%, signal=41%
5%, signal=67%
9%, signal=52%
8%, signal=49%
5%, signal=40%
7%, signal=57%
7%, signal=32%
5%, signal=34%
3%, signal=30%
5%, signal=43%
1%, signal=60%
8%, signal=64%
9%, signal=62%
5%, signal=55%
7%, signal=43%
4%, signal=63%
6%, signal=50%
1%, signal=47%
6%, signal=47%
7%, signal=49%

2%, signal=27%
6%, signal=57%
6%, signal=68%
1%, signal=52%
0%, signal=46%
2%, signal=89%
8%, signal=68%
2%, signal=44%
2%, signal=70%
7%, signal=55%
2%, signal=35%
6%, signal=68%
%, signal=34%
5%, signal=36%
6%, signal=36%
4%, signal=49%
8%, signal=39%
9%, signal=40%
5%, signal=103%
5%, signal=59%
9%, signal=77%
5%, signal=81%
1%, signal=66%
2%, signal=48%
2%, signal=33%
2%, signal=32%
9%, signal=46%
7%, signal=33%
5%, signal=35%
1%, signal=51%
%, signal=31%
9%, signal=61%
5%, signal=101%
6%, signal=78%
3%, signal=78%
1%, signal=85%
0%, signal=48%
%, signal=29%
8%, signal=42%
3%, signal=43%
6%, signal=59%
6%, signal=44%
0%, signal=39%
0%, signal=61%
3%, signal=56%
6%, signal=66%
3%, signal=34%
3%, signal=26%
9%, signal=39%
%, signal=18%
7%, signal=47%
4%, signal=72%
4%, signal=35%
6%, signal=90%
9%, signal=45%
9%, signal=51%
0%, signal=49%
0%, signal=39%

1%, signal=44%
7%, signal=55%
0%, signal=41%
0%, signal=42%
3%, signal=35%
2%, signal=92%
5%, signal=119%
1%, signal=61%
4%, signal=93%
6%, signal=49%
5%, signal=52%
0%, signal=79%
5%, signal=57%
8%, signal=38%
1%, signal=54%
4%, signal=35%
6%, signal=32%
4%, signal=39%
2%, signal=44%
2%, signal=58%
0%, signal=74%
9%, signal=34%
1%, signal=34%
2%, signal=56%
0%, signal=26%
2%, signal=56%
0%, signal=46%
6%, signal=50%
4%, signal=61%
7%, signal=38%
1%, signal=70%
8%, signal=63%
4%, signal=39%
6%, signal=81%
2%, signal=44%
0%, signal=47%
3%, signal=23%
6%, signal=40%
0%, signal=40%
9%, signal=41%
%, signal=31%
5%, signal=78%
1%, signal=28%
3%, signal=73%
5%, signal=55%
6%, signal=52%
1%, signal=26%
6%, signal=34%
2%, signal=44%
1%, signal=27%
1%, signal=52%
7%, signal=39%
3%, signal=37%
7%, signal=38%
9%, signal=41%
1%, signal=53%
5%, signal=89%
0%, signal=42%

2%, signal=41%
5%, signal=50%
9%, signal=36%
9%, signal=53%
6%, signal=41%
5%, signal=32%
%, signal=32%
7%, signal=38%
4%, signal=31%
9%, signal=55%
6%, signal=55%
%, signal=29%
0%, signal=40%
1%, signal=52%
7%, signal=57%
4%, signal=58%
1%, signal=30%
4%, signal=59%
4%, signal=48%
%, signal=28%
9%, signal=83%
3%, signal=65%
8%, signal=46%
2%, signal=56%
0%, signal=56%
9%, signal=48%
0%, signal=74%
4%, signal=38%
6%, signal=63%
6%, signal=72%
%, signal=32%
5%, signal=66%
7%, signal=37%
4%, signal=44%
0%, signal=28%
6%, signal=49%
1%, signal=55%
8%, signal=37%
2%, signal=47%
5%, signal=29%
0%, signal=26%
4%, signal=80%
5%, signal=36%
1%, signal=74%
0%, signal=40%
7%, signal=38%
5%, signal=47%
9%, signal=44%
2%, signal=59%
4%, signal=44%
0%, signal=44%
4%, signal=68%
1%, signal=44%
2%, signal=49%
%, signal=26%
3%, signal=40%
4%, signal=97%
6%, signal=63%

9%, signal=34%
7%, signal=43%
6%, signal=53%
%, signal=24%
7%, signal=50%
2%, signal=28%
6%, signal=40%
7%, signal=37%
5%, signal=51%
4%, signal=31%
1%, signal=43%
2%, signal=83%
6%, signal=42%
6%, signal=58%
3%, signal=45%
1%, signal=57%
9%, signal=42%
3%, signal=43%
%, signal=21%
6%, signal=53%
4%, signal=90%
2%, signal=32%
5%, signal=52%
0%, signal=68%
3%, signal=79%
7%, signal=31%
9%, signal=84%
3%, signal=46%
2%, signal=50%
4%, signal=77%
8%, signal=52%
8%, signal=60%
1%, signal=65%
2%, signal=32%
%, signal=37%
%, signal=20%
4%, signal=33%
3%, signal=59%
0%, signal=55%
6%, signal=36%
2%, signal=27%
%, signal=35%
2%, signal=43%
0%, signal=44%
9%, signal=39%
5%, signal=47%
6%, signal=43%
8%, signal=50%
%, signal=20%
1%, signal=47%
3%, signal=54%
5%, signal=56%
9%, signal=50%
2%, signal=44%
9%, signal=36%
5%, signal=61%
6%, signal=58%
0%, signal=39%

2%, signal=58%
6%, signal=57%
0%, signal=38%
2%, signal=87%
8%, signal=40%
0%, signal=31%
6%, signal=47%
1%, signal=35%
%, signal=23%
8%, signal=57%
1%, signal=43%
3%, signal=42%
%, signal=26%
2%, signal=30%
7%, signal=79%
2%, signal=49%
%, signal=23%
0%, signal=47%
3%, signal=39%
9%, signal=45%
6%, signal=76%
8%, signal=36%
1%, signal=43%
3%, signal=46%
5%, signal=75%
4%, signal=83%
5%, signal=46%
1%, signal=159%
8%, signal=40%
2%, signal=134%
0%, signal=43%
4%, signal=83%
4%, signal=37%
%, signal=34%
3%, signal=94%
7%, signal=60%
3%, signal=51%
2%, signal=40%
7%, signal=38%
3%, signal=47%
8%, signal=46%
3%, signal=102%
3%, signal=40%
7%, signal=36%
%, signal=27%
%, signal=27%
5%, signal=51%
2%, signal=51%
1%, signal=36%
6%, signal=58%
7%, signal=42%
0%, signal=55%
%, signal=24%
3%, signal=29%
5%, signal=52%
0%, signal=37%
4%, signal=48%
5%, signal=36%

4%, signal=35%
3%, signal=62%
%, signal=24%
4%, signal=34%
%, signal=34%
7%, signal=54%
%, signal=19%
7%, signal=38%
1%, signal=76%
2%, signal=43%
7%, signal=31%
7%, signal=52%
8%, signal=39%
5%, signal=59%
4%, signal=85%
0%, signal=60%
8%, signal=105%
8%, signal=49%
4%, signal=48%
%, signal=20%
4%, signal=38%
1%, signal=30%
%, signal=24%
%, signal=24%
4%, signal=50%
%, signal=31%
1%, signal=34%
8%, signal=51%
5%, signal=80%
8%, signal=41%
5%, signal=47%
3%, signal=42%
5%, signal=60%
9%, signal=36%
4%, signal=50%
3%, signal=50%
5%, signal=67%
4%, signal=27%
8%, signal=54%
8%, signal=67%
9%, signal=41%
8%, signal=36%
5%, signal=75%
6%, signal=43%
1%, signal=79%
0%, signal=44%
2%, signal=38%
%, signal=36%
1%, signal=23%
5%, signal=55%
1%, signal=41%
7%, signal=36%
1%, signal=47%
4%, signal=59%
2%, signal=62%
2%, signal=59%
3%, signal=70%
7%, signal=30%

6%, signal=38%
6%, signal=67%
3%, signal=35%
6%, signal=56%
2%, signal=39%
6%, signal=53%
4%, signal=78%
3%, signal=36%
9%, signal=94%
%, signal=22%
0%, signal=65%
0%, signal=40%
1%, signal=59%
9%, signal=39%
4%, signal=50%
2%, signal=24%
8%, signal=51%
1%, signal=41%
0%, signal=39%
4%, signal=88%
%, signal=14%
6%, signal=32%
0%, signal=23%
%, signal=23%
7%, signal=43%
7%, signal=63%
4%, signal=40%
%, signal=31%
9%, signal=39%
6%, signal=45%
5%, signal=36%
9%, signal=40%
8%, signal=40%
0%, signal=42%
7%, signal=73%
8%, signal=36%
5%, signal=86%
7%, signal=30%
1%, signal=32%
6%, signal=42%
5%, signal=32%
3%, signal=65%
2%, signal=96%
1%, signal=117%
7%, signal=47%
0%, signal=44%
6%, signal=41%
7%, signal=94%
6%, signal=27%
1%, signal=47%
5%, signal=60%
4%, signal=37%
8%, signal=57%
9%, signal=34%
0%, signal=78%
3%, signal=39%
2%, signal=41%
7%, signal=32%

2%, signal=38%
4%, signal=55%
7%, signal=67%
7%, signal=35%
0%, signal=71%
3%, signal=70%
8%, signal=84%
8%, signal=39%
1%, signal=114%
7%, signal=47%
1%, signal=39%
7%, signal=40%
%, signal=29%
2%, signal=25%
1%, signal=30%
3%, signal=62%
1%, signal=25%
9%, signal=49%
2%, signal=28%
7%, signal=42%
6%, signal=57%
9%, signal=41%
0%, signal=66%
4%, signal=117%
3%, signal=37%
2%, signal=59%
5%, signal=30%
6%, signal=67%
7%, signal=43%
4%, signal=51%
2%, signal=41%
5%, signal=28%
2%, signal=30%
4%, signal=29%
3%, signal=73%
0%, signal=50%
5%, signal=35%
9%, signal=40%
2%, signal=84%
9%, signal=101%
8%, signal=54%
3%, signal=31%
0%, signal=68%
1%, signal=36%
%, signal=28%
7%, signal=55%
0%, signal=38%
7%, signal=54%
6%, signal=58%
1%, signal=83%
2%, signal=30%
0%, signal=50%
1%, signal=45%
9%, signal=35%
7%, signal=85%
7%, signal=58%
5%, signal=36%
5%, signal=62%

4%, signal=44%
2%, signal=38%
9%, signal=34%
0%, signal=40%
9%, signal=31%
9%, signal=63%
8%, signal=41%
6%, signal=66%
6%, signal=33%
0%, signal=27%
%, signal=22%
8%, signal=66%
4%, signal=25%
1%, signal=74%
9%, signal=37%
4%, signal=35%
8%, signal=44%
1%, signal=27%
4%, signal=32%
1%, signal=60%
8%, signal=42%
1%, signal=69%
6%, signal=41%
7%, signal=50%
1%, signal=44%
1%, signal=39%
8%, signal=46%
5%, signal=30%
%, signal=28%
7%, signal=57%
8%, signal=34%
9%, signal=62%
5%, signal=75%
4%, signal=41%
1%, signal=71%
2%, signal=40%
5%, signal=29%
5%, signal=44%
9%, signal=59%
2%, signal=45%
0%, signal=36%
8%, signal=49%
0%, signal=46%
1%, signal=42%
8%, signal=37%
6%, signal=70%
5%, signal=33%
%, signal=29%
7%, signal=58%
3%, signal=35%
1%, signal=64%
4%, signal=44%
1%, signal=40%
9%, signal=33%
2%, signal=87%
7%, signal=57%
2%, signal=59%
3%, signal=22%

5%, signal=30%
%, signal=17%
%, signal=17%
3%, signal=59%
2%, signal=35%
8%, signal=33%
0%, signal=48%
6%, signal=44%
2%, signal=53%
4%, signal=50%
1%, signal=74%
6%, signal=56%
6%, signal=61%
4%, signal=28%
5%, signal=53%
1%, signal=39%
1%, signal=65%
%, signal=25%
4%, signal=50%
7%, signal=53%
1%, signal=41%
2%, signal=32%
7%, signal=33%
6%, signal=40%
7%, signal=40%
%, signal=20%
%, signal=26%
9%, signal=53%
2%, signal=33%
1%, signal=53%
0%, signal=36%
%, signal=34%
%, signal=19%
2%, signal=48%
9%, signal=40%
7%, signal=33%
4%, signal=56%
1%, signal=45%
3%, signal=37%
3%, signal=34%
6%, signal=27%
%, signal=31%
1%, signal=37%
5%, signal=55%
1%, signal=82%
0%, signal=38%
4%, signal=92%
5%, signal=44%
8%, signal=39%
5%, signal=39%
1%, signal=30%
0%, signal=21%
%, signal=22%
0%, signal=28%
2%, signal=29%
8%, signal=33%
6%, signal=55%
4%, signal=36%

4%, signal=89%
5%, signal=71%
7%, signal=45%
9%, signal=44%
9%, signal=40%
0%, signal=45%
2%, signal=35%
7%, signal=34%
7%, signal=31%
8%, signal=41%
8%, signal=42%
3%, signal=40%
1%, signal=91%
1%, signal=39%
8%, signal=65%
4%, signal=56%
0%, signal=46%
7%, signal=37%
6%, signal=53%
9%, signal=41%
1%, signal=42%
7%, signal=48%
%, signal=18%
4%, signal=91%
1%, signal=54%
2%, signal=46%
9%, signal=41%
8%, signal=33%
%, signal=15%
2%, signal=41%
%, signal=18%
%, signal=20%
5%, signal=32%
8%, signal=44%
4%, signal=70%
1%, signal=24%
1%, signal=48%
3%, signal=79%
6%, signal=42%
0%, signal=45%
4%, signal=52%
9%, signal=37%
0%, signal=25%
7%, signal=46%
1%, signal=106%
6%, signal=48%
2%, signal=29%
8%, signal=50%
4%, signal=70%
9%, signal=48%
7%, signal=56%
6%, signal=77%
2%, signal=33%
2%, signal=40%
7%, signal=40%
6%, signal=55%
1%, signal=31%
4%, signal=35%

2%, signal=45%
2%, signal=24%
0%, signal=50%
%, signal=23%
6%, signal=42%
1%, signal=38%
9%, signal=34%
2%, signal=53%
3%, signal=44%
1%, signal=50%
4%, signal=35%
4%, signal=47%
2%, signal=64%
%, signal=31%
7%, signal=56%
8%, signal=53%
3%, signal=33%
2%, signal=28%
4%, signal=68%
4%, signal=33%
7%, signal=69%
8%, signal=33%
1%, signal=97%
2%, signal=39%
8%, signal=30%
2%, signal=37%
4%, signal=47%
2%, signal=56%
0%, signal=24%
4%, signal=32%
8%, signal=105%
%, signal=16%
0%, signal=37%
6%, signal=46%
4%, signal=36%
1%, signal=86%
5%, signal=37%
%, signal=22%
7%, signal=33%
9%, signal=39%
4%, signal=47%
0%, signal=21%
9%, signal=53%
7%, signal=29%
0%, signal=74%
1%, signal=44%
7%, signal=30%
0%, signal=30%
6%, signal=30%
5%, signal=76%
8%, signal=44%
%, signal=24%
4%, signal=35%
5%, signal=26%
7%, signal=35%
7%, signal=35%
2%, signal=78%
6%, signal=57%

0%, signal=64%
8%, signal=57%
4%, signal=27%
7%, signal=34%
4%, signal=53%
8%, signal=40%
3%, signal=38%
8%, signal=44%
7%, signal=36%
0%, signal=65%
%, signal=28%
3%, signal=52%
6%, signal=40%
%, signal=24%
9%, signal=97%
2%, signal=50%
3%, signal=31%
1%, signal=32%
7%, signal=50%
%, signal=20%
5%, signal=39%
0%, signal=44%
5%, signal=35%
1%, signal=37%
4%, signal=51%
0%, signal=23%
7%, signal=86%
4%, signal=26%
1%, signal=48%
5%, signal=42%
7%, signal=55%
%, signal=31%
0%, signal=73%
6%, signal=39%
4%, signal=53%
5%, signal=40%
8%, signal=35%
1%, signal=57%
5%, signal=82%
7%, signal=48%
4%, signal=80%
5%, signal=35%
7%, signal=39%
3%, signal=41%
0%, signal=25%
4%, signal=56%
5%, signal=34%
8%, signal=51%
3%, signal=69%
2%, signal=42%
5%, signal=28%
1%, signal=25%
2%, signal=44%
7%, signal=59%
7%, signal=36%
2%, signal=54%
1%, signal=46%
7%, signal=40%

5%, signal=33%
3%, signal=43%
8%, signal=33%
3%, signal=61%
2%, signal=32%
6%, signal=30%
8%, signal=30%
9%, signal=49%
6%, signal=51%
3%, signal=40%
7%, signal=63%
%, signal=19%
%, signal=26%
5%, signal=61%
3%, signal=44%
%, signal=28%
8%, signal=54%
4%, signal=33%
7%, signal=33%
7%, signal=35%
3%, signal=41%
4%, signal=28%
7%, signal=36%
3%, signal=48%
6%, signal=93%
9%, signal=52%
9%, signal=71%
0%, signal=50%
2%, signal=65%
9%, signal=74%
5%, signal=36%
1%, signal=44%
2%, signal=47%
2%, signal=41%
4%, signal=56%
%, signal=21%
0%, signal=37%
2%, signal=43%
2%, signal=37%
0%, signal=29%
2%, signal=33%
2%, signal=64%
2%, signal=38%
0%, signal=46%
1%, signal=25%
8%, signal=38%
2%, signal=39%
1%, signal=119%
1%, signal=124%
4%, signal=33%
1%, signal=58%
%, signal=30%
0%, signal=58%
5%, signal=61%
9%, signal=31%
7%, signal=40%
6%, signal=40%
7%, signal=33%

%, signal=15%
1%, signal=36%
7%, signal=48%
8%, signal=42%
8%, signal=36%
1%, signal=21%
2%, signal=40%
1%, signal=32%
6%, signal=52%
9%, signal=57%
6%, signal=33%
2%, signal=26%
1%, signal=70%
7%, signal=64%
3%, signal=41%
3%, signal=41%
4%, signal=28%
6%, signal=60%
4%, signal=32%
6%, signal=28%
3%, signal=39%
7%, signal=58%
3%, signal=31%
%, signal=20%
7%, signal=41%
9%, signal=33%
3%, signal=51%
2%, signal=50%
%, signal=26%
0%, signal=41%
%, signal=25%
0%, signal=61%
3%, signal=50%
9%, signal=52%
3%, signal=46%
1%, signal=37%
6%, signal=35%
2%, signal=26%
8%, signal=104%
1%, signal=25%
4%, signal=36%
1%, signal=46%
0%, signal=72%
5%, signal=59%
6%, signal=30%
7%, signal=60%
4%, signal=29%
7%, signal=34%
6%, signal=51%
2%, signal=43%
9%, signal=35%
8%, signal=43%
7%, signal=29%
8%, signal=40%
2%, signal=41%
1%, signal=38%
8%, signal=35%
2%, signal=39%

5%, signal=35%
%, signal=26%
%, signal=26%
2%, signal=47%
4%, signal=30%
0%, signal=37%
2%, signal=54%
6%, signal=57%
2%, signal=36%
2%, signal=60%
4%, signal=73%
8%, signal=39%
%, signal=17%
2%, signal=45%
2%, signal=93%
6%, signal=42%
4%, signal=31%
4%, signal=23%
%, signal=20%
9%, signal=47%
2%, signal=31%
6%, signal=34%
8%, signal=38%
5%, signal=60%
4%, signal=32%
3%, signal=68%
6%, signal=34%
1%, signal=33%
0%, signal=39%
6%, signal=28%
8%, signal=46%
8%, signal=32%
6%, signal=43%
9%, signal=57%
7%, signal=42%
1%, signal=38%
7%, signal=73%
7%, signal=26%
5%, signal=41%
%, signal=23%
0%, signal=27%
0%, signal=71%
9%, signal=40%
5%, signal=59%
%, signal=21%
2%, signal=41%
1%, signal=31%
3%, signal=48%
7%, signal=35%
%, signal=23%
9%, signal=29%
2%, signal=23%
4%, signal=58%
3%, signal=88%
4%, signal=41%
6%, signal=36%
%, signal=18%
3%, signal=44%

8%, signal=35%
%, signal=23%
1%, signal=44%
5%, signal=31%
4%, signal=66%
%, signal=19%
%, signal=27%
5%, signal=30%
%, signal=21%
0%, signal=19%
5%, signal=48%
9%, signal=37%
5%, signal=25%
7%, signal=58%
8%, signal=41%
7%, signal=56%
0%, signal=35%
%, signal=31%
0%, signal=24%
8%, signal=41%
5%, signal=57%
0%, signal=25%
3%, signal=30%
5%, signal=41%
1%, signal=24%
6%, signal=30%
9%, signal=41%
7%, signal=32%
2%, signal=74%
2%, signal=25%
2%, signal=30%
4%, signal=54%
8%, signal=40%
5%, signal=67%
6%, signal=35%
2%, signal=45%
%, signal=21%
1%, signal=30%
0%, signal=188%
3%, signal=42%
6%, signal=60%
8%, signal=32%
%, signal=18%
1%, signal=41%
1%, signal=57%
7%, signal=34%
1%, signal=27%
9%, signal=48%
2%, signal=37%
4%, signal=51%
8%, signal=40%
6%, signal=34%
8%, signal=93%
7%, signal=28%
1%, signal=60%
4%, signal=26%
3%, signal=27%
3%, signal=68%

6%, signal=41%
%, signal=22%
7%, signal=41%
6%, signal=34%
7%, signal=57%
8%, signal=83%
7%, signal=34%
2%, signal=41%
2%, signal=36%
3%, signal=62%
3%, signal=33%
4%, signal=70%
2%, signal=42%
3%, signal=81%
5%, signal=25%
0%, signal=118%
2%, signal=51%
2%, signal=41%
2%, signal=23%
9%, signal=51%
2%, signal=48%
6%, signal=33%
4%, signal=34%
1%, signal=23%
3%, signal=65%
8%, signal=59%
5%, signal=25%
0%, signal=56%
%, signal=22%
6%, signal=35%
7%, signal=60%
4%, signal=34%
3%, signal=42%
0%, signal=30%
0%, signal=42%
4%, signal=44%
1%, signal=63%
9%, signal=46%
7%, signal=43%
2%, signal=21%
5%, signal=31%
1%, signal=38%
0%, signal=81%
4%, signal=70%
8%, signal=38%
%, signal=14%
5%, signal=33%
6%, signal=37%
4%, signal=57%
1%, signal=50%
%, signal=20%
3%, signal=48%
0%, signal=38%
3%, signal=100%
4%, signal=44%
3%, signal=63%
6%, signal=49%
6%, signal=34%

7%, signal=56%
3%, signal=53%
3%, signal=43%
6%, signal=43%
2%, signal=42%
4%, signal=34%
0%, signal=58%
0%, signal=41%
4%, signal=33%
7%, signal=38%
9%, signal=41%
4%, signal=47%
5%, signal=49%
3%, signal=50%
5%, signal=28%
4%, signal=36%
8%, signal=96%
9%, signal=28%
4%, signal=49%
4%, signal=46%
%, signal=23%
5%, signal=29%
3%, signal=22%
9%, signal=53%
4%, signal=44%
4%, signal=60%
6%, signal=69%
6%, signal=35%
5%, signal=50%
2%, signal=33%
2%, signal=67%
5%, signal=52%
0%, signal=27%
8%, signal=42%
6%, signal=48%
0%, signal=39%
6%, signal=57%
1%, signal=40%
0%, signal=24%
2%, signal=88%
9%, signal=30%
1%, signal=28%
8%, signal=32%
9%, signal=36%
0%, signal=29%
7%, signal=46%
3%, signal=70%
2%, signal=73%
3%, signal=38%
2%, signal=38%
2%, signal=39%
9%, signal=30%
%, signal=28%
1%, signal=60%
2%, signal=46%
9%, signal=71%
5%, signal=50%
1%, signal=54%

7%, signal=32%
6%, signal=147%
4%, signal=27%
4%, signal=26%
6%, signal=78%
7%, signal=45%
3%, signal=77%
2%, signal=35%
1%, signal=28%
3%, signal=27%
1%, signal=46%
2%, signal=46%
0%, signal=33%
3%, signal=27%
1%, signal=33%
8%, signal=42%
0%, signal=37%
6%, signal=31%
7%, signal=33%
%, signal=14%
7%, signal=45%
6%, signal=27%
0%, signal=36%
8%, signal=64%
1%, signal=46%
8%, signal=63%
%, signal=37%
5%, signal=27%
9%, signal=54%
2%, signal=21%
%, signal=36%
0%, signal=50%
1%, signal=25%
4%, signal=46%
4%, signal=47%
5%, signal=31%
7%, signal=34%
7%, signal=34%
3%, signal=115%
8%, signal=99%
7%, signal=42%
2%, signal=77%
2%, signal=52%
4%, signal=36%
4%, signal=46%
5%, signal=56%
9%, signal=33%
%, signal=14%
2%, signal=60%
2%, signal=45%
4%, signal=58%
2%, signal=31%
%, signal=13%
5%, signal=30%
6%, signal=47%
0%, signal=40%
9%, signal=46%
2%, signal=28%

9%, signal=34%
9%, signal=40%
3%, signal=38%
0%, signal=42%
9%, signal=65%
5%, signal=32%
5%, signal=32%
7%, signal=73%
6%, signal=24%
9%, signal=29%
7%, signal=47%
3%, signal=24%
0%, signal=36%
4%, signal=35%
5%, signal=27%
%, signal=22%
5%, signal=35%
3%, signal=45%
7%, signal=63%
8%, signal=39%
4%, signal=79%
2%, signal=45%
4%, signal=40%
7%, signal=28%
4%, signal=61%
4%, signal=52%
0%, signal=32%
8%, signal=49%
4%, signal=44%
%, signal=19%
%, signal=20%
%, signal=17%
5%, signal=42%
0%, signal=33%
8%, signal=55%
4%, signal=35%
%, signal=18%
2%, signal=71%
2%, signal=30%
7%, signal=32%
8%, signal=53%
0%, signal=25%
9%, signal=51%
7%, signal=45%
8%, signal=37%
0%, signal=25%
5%, signal=60%
1%, signal=26%
2%, signal=23%
%, signal=19%
3%, signal=33%
7%, signal=24%
0%, signal=54%
6%, signal=40%
2%, signal=22%
5%, signal=28%
4%, signal=31%
%, signal=21%

1%, signal=81%
1%, signal=22%
5%, signal=74%
7%, signal=40%
0%, signal=35%
4%, signal=37%
1%, signal=26%
9%, signal=75%
5%, signal=39%
4%, signal=102%
5%, signal=27%
4%, signal=34%
3%, signal=21%
6%, signal=29%
2%, signal=39%
9%, signal=57%
3%, signal=26%
2%, signal=32%
2%, signal=27%
1%, signal=37%
7%, signal=30%
0%, signal=36%
7%, signal=52%
1%, signal=70%
1%, signal=55%
8%, signal=54%
4%, signal=44%
1%, signal=32%
4%, signal=27%
4%, signal=46%
%, signal=13%
8%, signal=32%
8%, signal=63%
4%, signal=31%
3%, signal=59%
6%, signal=58%
8%, signal=43%
9%, signal=34%
8%, signal=57%
7%, signal=29%
6%, signal=70%
0%, signal=20%
1%, signal=41%
0%, signal=23%
6%, signal=96%
3%, signal=69%
3%, signal=38%
%, signal=25%
3%, signal=28%
2%, signal=40%
4%, signal=90%
2%, signal=23%
0%, signal=58%
8%, signal=51%
4%, signal=41%
7%, signal=53%
7%, signal=59%
7%, signal=57%

4%, signal=45%
1%, signal=42%
7%, signal=69%
4%, signal=36%
3%, signal=28%
8%, signal=95%
6%, signal=53%
%, signal=21%
7%, signal=52%
4%, signal=52%
%, signal=22%
5%, signal=32%
0%, signal=25%
1%, signal=37%
0%, signal=18%
%, signal=25%
5%, signal=54%
%, signal=32%
6%, signal=31%
6%, signal=135%
8%, signal=44%
3%, signal=24%
3%, signal=25%
%, signal=28%
6%, signal=34%
2%, signal=78%
8%, signal=102%
2%, signal=37%
8%, signal=37%
0%, signal=29%
9%, signal=82%
9%, signal=45%
4%, signal=58%
1%, signal=22%
8%, signal=51%
%, signal=19%
2%, signal=55%
%, signal=24%
9%, signal=66%
1%, signal=28%
9%, signal=31%
7%, signal=35%
0%, signal=67%
%, signal=23%
5%, signal=27%
4%, signal=54%
7%, signal=43%
%, signal=25%
5%, signal=31%
8%, signal=50%
%, signal=22%
4%, signal=81%
%, signal=21%
2%, signal=30%
0%, signal=63%
%, signal=20%
%, signal=18%
0%, signal=94%

5%, signal=31%
9%, signal=46%
6%, signal=77%
4%, signal=72%
5%, signal=86%
7%, signal=30%
0%, signal=61%
8%, signal=63%
%, signal=23%
9%, signal=41%
9%, signal=41%
3%, signal=38%
4%, signal=22%
%, signal=21%
0%, signal=31%
0%, signal=23%
7%, signal=54%
0%, signal=122%
2%, signal=39%
8%, signal=37%
0%, signal=25%
8%, signal=55%
1%, signal=37%
5%, signal=43%
4%, signal=57%
1%, signal=21%
0%, signal=46%
1%, signal=49%
1%, signal=30%
8%, signal=29%
6%, signal=31%
6%, signal=32%
5%, signal=28%
9%, signal=44%
8%, signal=42%
0%, signal=42%
9%, signal=41%
7%, signal=30%
9%, signal=34%
7%, signal=29%
9%, signal=44%
4%, signal=37%
%, signal=22%
9%, signal=51%
1%, signal=49%
%, signal=16%
7%, signal=56%
1%, signal=24%
7%, signal=44%
7%, signal=29%
8%, signal=32%
6%, signal=76%
3%, signal=76%
7%, signal=29%
9%, signal=32%
2%, signal=25%
4%, signal=57%
9%, signal=61%

%, signal=20%
8%, signal=27%
9%, signal=40%
%, signal=15%
6%, signal=29%
4%, signal=25%
%, signal=13%
0%, signal=26%
4%, signal=25%
6%, signal=59%
3%, signal=47%
4%, signal=26%
9%, signal=38%
6%, signal=27%
7%, signal=49%
7%, signal=38%
1%, signal=33%
4%, signal=22%
9%, signal=52%
7%, signal=57%
3%, signal=41%
6%, signal=30%
7%, signal=32%
2%, signal=25%
0%, signal=42%
6%, signal=53%
2%, signal=112%
5%, signal=33%
1%, signal=25%
%, signal=21%
9%, signal=35%
5%, signal=30%
3%, signal=46%
9%, signal=34%
5%, signal=28%
5%, signal=45%
2%, signal=23%
2%, signal=47%
2%, signal=44%
6%, signal=36%
4%, signal=24%
2%, signal=48%
4%, signal=45%
8%, signal=52%
2%, signal=17%
5%, signal=26%
2%, signal=110%
5%, signal=28%
5%, signal=30%
8%, signal=42%
5%, signal=41%
2%, signal=93%
0%, signal=57%
5%, signal=73%
6%, signal=27%
7%, signal=31%
0%, signal=23%
3%, signal=26%

%, signal=21%
7%, signal=51%
%, signal=13%
1%, signal=21%
3%, signal=22%
1%, signal=51%
3%, signal=31%
0%, signal=22%
%, signal=16%
%, signal=17%
0%, signal=26%
1%, signal=44%
%, signal=21%
8%, signal=63%
4%, signal=32%
5%, signal=43%
7%, signal=69%
5%, signal=25%
8%, signal=27%
1%, signal=66%
0%, signal=40%
0%, signal=64%
7%, signal=39%
0%, signal=29%
6%, signal=28%
%, signal=20%
%, signal=24%
2%, signal=39%
3%, signal=24%
6%, signal=27%
1%, signal=22%
4%, signal=27%
3%, signal=29%
%, signal=15%
3%, signal=75%
7%, signal=25%
6%, signal=29%
1%, signal=20%
2%, signal=32%
2%, signal=32%
7%, signal=52%
0%, signal=26%
8%, signal=34%
8%, signal=47%
2%, signal=30%
6%, signal=68%
9%, signal=54%
2%, signal=101%
2%, signal=47%
2%, signal=27%
0%, signal=30%
2%, signal=53%
1%, signal=22%
%, signal=17%
1%, signal=41%
6%, signal=46%
0%, signal=22%
8%, signal=27%

0%, signal=18%
8%, signal=49%
1%, signal=53%
5%, signal=25%
0%, signal=55%
4%, signal=31%
2%, signal=39%
8%, signal=31%
6%, signal=44%
%, signal=15%
7%, signal=33%
4%, signal=49%
5%, signal=45%
6%, signal=46%
5%, signal=57%
2%, signal=43%
9%, signal=41%
%, signal=15%
7%, signal=50%
7%, signal=28%
3%, signal=36%
%, signal=18%
2%, signal=57%
1%, signal=66%
2%, signal=60%
8%, signal=29%
%, signal=24%
0%, signal=34%
7%, signal=34%
7%, signal=47%
0%, signal=34%
3%, signal=32%
1%, signal=52%
0%, signal=42%
%, signal=14%
%, signal=21%
4%, signal=30%
3%, signal=38%
4%, signal=26%
8%, signal=48%
2%, signal=41%
3%, signal=25%
%, signal=17%
%, signal=12%
7%, signal=56%
8%, signal=35%
1%, signal=22%
7%, signal=44%
6%, signal=25%
9%, signal=50%
9%, signal=38%
9%, signal=36%
7%, signal=26%
7%, signal=29%
9%, signal=134%
9%, signal=31%
7%, signal=125%
3%, signal=87%

0%, signal=39%
7%, signal=87%
%, signal=21%
7%, signal=26%
1%, signal=42%
2%, signal=61%
9%, signal=63%
2%, signal=41%
2%, signal=82%
%, signal=24%
4%, signal=39%
1%, signal=24%
1%, signal=29%
2%, signal=35%
2%, signal=38%
2%, signal=44%
7%, signal=58%
2%, signal=44%
3%, signal=37%
5%, signal=67%
7%, signal=37%
0%, signal=29%
2%, signal=36%
%, signal=22%
2%, signal=42%
5%, signal=24%
8%, signal=78%
2%, signal=24%
7%, signal=28%
5%, signal=83%
0%, signal=40%
3%, signal=25%
4%, signal=28%
3%, signal=68%
1%, signal=81%
9%, signal=43%
7%, signal=35%
2%, signal=40%
1%, signal=33%
6%, signal=68%
0%, signal=32%
%, signal=20%
2%, signal=44%
%, signal=12%
1%, signal=41%
%, signal=12%
%, signal=13%
0%, signal=22%
3%, signal=54%
0%, signal=41%
6%, signal=59%
1%, signal=74%
6%, signal=55%
5%, signal=65%
5%, signal=39%
2%, signal=35%
1%, signal=64%
7%, signal=66%

0%, signal=74%
9%, signal=87%
8%, signal=30%
4%, signal=32%
2%, signal=18%
6%, signal=27%
1%, signal=52%
0%, signal=22%
7%, signal=38%
8%, signal=36%
0%, signal=46%
9%, signal=59%
%, signal=14%
2%, signal=54%
9%, signal=54%
4%, signal=27%
1%, signal=45%
5%, signal=25%
6%, signal=29%
1%, signal=37%
1%, signal=61%
4%, signal=28%
8%, signal=58%
%, signal=23%
7%, signal=41%
2%, signal=53%
0%, signal=33%
1%, signal=54%
6%, signal=24%
1%, signal=55%
0%, signal=26%
6%, signal=42%
5%, signal=27%
5%, signal=82%
6%, signal=25%
2%, signal=21%
8%, signal=57%
1%, signal=48%
0%, signal=22%
0%, signal=66%
2%, signal=43%
2%, signal=29%
%, signal=13%
%, signal=15%
0%, signal=36%
3%, signal=23%
0%, signal=28%
3%, signal=83%
5%, signal=26%
2%, signal=21%
3%, signal=34%
5%, signal=48%
5%, signal=27%
7%, signal=25%
5%, signal=63%
9%, signal=34%
9%, signal=38%
2%, signal=42%

%, signal=14%
7%, signal=33%
7%, signal=33%
1%, signal=47%
3%, signal=21%
2%, signal=24%
8%, signal=58%
7%, signal=30%
6%, signal=28%
8%, signal=96%
1%, signal=20%
4%, signal=29%
0%, signal=54%
5%, signal=24%
0%, signal=33%
6%, signal=124%
8%, signal=60%
8%, signal=38%
%, signal=22%
%, signal=18%
4%, signal=49%
7%, signal=46%
9%, signal=29%
5%, signal=45%
8%, signal=57%
%, signal=17%
8%, signal=29%
7%, signal=44%
1%, signal=23%
%, signal=19%
5%, signal=25%
7%, signal=54%
8%, signal=31%
0%, signal=48%
7%, signal=45%
7%, signal=56%
1%, signal=33%
0%, signal=28%
6%, signal=24%
2%, signal=54%
3%, signal=21%
5%, signal=48%
6%, signal=30%
8%, signal=93%
2%, signal=56%
7%, signal=24%
6%, signal=117%
6%, signal=22%
0%, signal=40%
2%, signal=36%
7%, signal=30%
2%, signal=23%
5%, signal=44%
9%, signal=54%
8%, signal=28%
9%, signal=53%
6%, signal=31%
6%, signal=25%

2%, signal=33%
8%, signal=65%
6%, signal=39%
2%, signal=34%
7%, signal=31%
7%, signal=43%
8%, signal=24%
4%, signal=73%
0%, signal=34%
0%, signal=112%
4%, signal=56%
1%, signal=34%
4%, signal=53%
%, signal=17%
3%, signal=36%
0%, signal=47%
1%, signal=69%
%, signal=14%
9%, signal=28%
4%, signal=91%
2%, signal=23%
%, signal=22%
8%, signal=31%
4%, signal=51%
8%, signal=65%
0%, signal=67%
4%, signal=19%
5%, signal=32%
8%, signal=61%
%, signal=11%
7%, signal=28%
7%, signal=46%
1%, signal=66%
7%, signal=27%
2%, signal=47%
%, signal=20%
8%, signal=55%
3%, signal=22%
9%, signal=54%
8%, signal=45%
2%, signal=21%
%, signal=14%
6%, signal=43%
1%, signal=30%
1%, signal=19%
6%, signal=61%
0%, signal=31%
4%, signal=26%
5%, signal=38%
7%, signal=42%
1%, signal=101%
7%, signal=26%
5%, signal=24%
%, signal=11%
2%, signal=89%
4%, signal=39%
0%, signal=59%
6%, signal=45%

9%, signal=25%
9%, signal=28%
8%, signal=84%
5%, signal=25%
8%, signal=49%
9%, signal=53%
5%, signal=29%
6%, signal=26%
%, signal=12%
7%, signal=33%
7%, signal=38%
2%, signal=61%
8%, signal=30%
8%, signal=43%
5%, signal=118%
4%, signal=27%
%, signal=15%
%, signal=17%
9%, signal=53%
9%, signal=65%
9%, signal=44%
2%, signal=33%
6%, signal=43%
5%, signal=76%
6%, signal=28%
8%, signal=37%
1%, signal=34%
5%, signal=56%
2%, signal=52%
6%, signal=57%
8%, signal=63%
%, signal=13%
2%, signal=83%
5%, signal=60%
8%, signal=27%
2%, signal=44%
3%, signal=21%
7%, signal=88%
4%, signal=59%
4%, signal=25%
3%, signal=22%
7%, signal=45%
8%, signal=83%
8%, signal=62%
6%, signal=28%
0%, signal=52%
0%, signal=23%
1%, signal=67%
0%, signal=22%
9%, signal=29%
1%, signal=38%
2%, signal=18%
7%, signal=88%
8%, signal=42%
%, signal=22%
8%, signal=28%
4%, signal=34%
3%, signal=22%

5%, signal=107%
7%, signal=65%
8%, signal=26%
4%, signal=27%
3%, signal=56%
0%, signal=31%
2%, signal=32%
9%, signal=54%
4%, signal=63%
8%, signal=32%
7%, signal=40%
7%, signal=46%
%, signal=22%
2%, signal=44%
8%, signal=28%
3%, signal=64%
%, signal=11%
7%, signal=28%

7%, signal=54%
%, signal=13%
5%, signal=70%
0%, signal=67%
5%, signal=22%
2%, signal=84%
1%, signal=51%
9%, signal=49%
8%, signal=56%
6%, signal=78%
7%, signal=27%
5%, signal=73%
%, signal=16%
8%, signal=70%
0%, signal=34%
4%, signal=58%
2%, signal=68%
5%, signal=26%
6%, signal=38%
6%, signal=57%
7%, signal=28%
5%, signal=45%
3%, signal=25%
2%, signal=18%
2%, signal=54%
%, signal=11%
%, signal=13%
6%, signal=42%
2%, signal=61%
5%, signal=50%
5%, signal=19%
3%, signal=57%
0%, signal=17%
0%, signal=34%
8%, signal=61%
3%, signal=58%
8%, signal=46%
1%, signal=48%
3%, signal=32%

9%, signal=33%
0%, signal=24%
9%, signal=90%
2%, signal=52%
3%, signal=71%
4%, signal=29%
0%, signal=11%
9%, signal=72%
4%, signal=31%
8%, signal=23%
4%, signal=24%
1%, signal=13%
2%, signal=68%
2%, signal=49%

7%, signal=24%
7%, signal=35%
8%, signal=48%
3%, signal=35%
9%, signal=71%
5%, signal=77%
8%, signal=74%
1%, signal=51%
3%, signal=52%
7%, signal=115%

6%, signal=96%
9%, signal=80%

4%, signal=72%
9%, signal=22%
8%, signal=46%

# LOW-ETV7

| NAME | GS<br> follow | GS DETAIL | SIZE | ES | NES | NOM p | FDR q-v | FWER p- | RANK | LEADING EDGE |
|---|---|---|---|---|---|---|---|---|---|---|
| GO RESPON | GO RESPONS | Details ... | 195 | 0.8 | 2.717 | 0 | 0 | 0 | 4226 | tags=57%, list=7%, sign |
| GO TUMOR | GO TUMOR N | Details ... | 167 | 0.71 | 2.664 | 0 | 0 | 0 | 7722 | tags=55%, list=13%, sig |
| GO RESPON | GO RESPONS | Details ... | 337 | 0.68 | 2.649 | 0 | 0 | 0 | 3967 | tags=41%, list=7%, sign |
| GO DEFENS | GO DEFENSE | Details ... | 248 | 0.72 | 2.642 | 0 | 0 | 0 | 4091 | tags=44%, list=7%, sign |
| GO INTERFE | GO INTERFER | Details ... | 89 | 0.88 | 2.635 | 0 | 0 | 0 | 2184 | tags=63%, list=4%, sign |
| GO RESPON | GO RESPONS | Details ... | 98 | 0.83 | 2.635 | 0 | 0 | 0 | 2184 | tags=48%, list=4%, sign |
| GO T CELL | GO T CELL R | Details ... | 200 | 0.75 | 2.614 | 0 | 0 | 0 | 2512 | tags=45%, list=4%, sign |
| GO NEGATI | GO NEGATIV | Details ... | 100 | 0.76 | 2.561 | 0 | 0 | 0 | 1638 | tags=39%, list=3%, sign |
| GO NEGATI | GO NEGATIV | Details ... | 157 | 0.7 | 2.547 | 0 | 0 | 0 | 4992 | tags=45%, list=9%, sign |
| GO REGULA | GO REGULAT | Details ... | 397 | 0.66 | 2.519 | 0 | 0 | 0 | 5145 | tags=41%, list=9%, sign |
| GO LEUKOC | GO LEUKOCY | Details ... | 106 | 0.75 | 2.51 | 0 | 0 | 0 | 4639 | tags=54%, list=8%, sign |
| GO CELL KI | GO CELL KIL | Details ... | 168 | 0.71 | 2.508 | 0 | 0 | 0 | 7298 | tags=55%, list=12%, sig |
| GO REGULA | GO REGULAT | Details ... | 300 | 0.68 | 2.504 | 0 | 0 | 0 | 5145 | tags=44%, list=9%, sign |
| GO RESPON | GO RESPONS | Details ... | 27 | 0.85 | 2.503 | 0 | 0 | 0 | 3436 | tags=56%, list=6%, sign |
| GO NEGATI | GO NEGATIV | Details ... | 82 | 0.76 | 2.501 | 0 | 0 | 0 | 1638 | tags=41%, list=3%, sign |
| GO T CELL | GO T CELL M | Details ... | 102 | 0.73 | 2.493 | 0 | 0 | 0 | 4639 | tags=46%, list=8%, sign |
| GO NATURA | GO NATURAL | Details ... | 84 | 0.71 | 2.49 | 0 | 0 | 0 | 3951 | tags=44%, list=7%, sign |
| GO REGULA | GO REGULAT | Details ... | 90 | 0.8 | 2.487 | 0 | 0 | 0 | 5312 | tags=61%, list=9%, sign |
| GO NEGATI | GO NEGATIV | Details ... | 150 | 0.69 | 2.484 | 0 | 0 | 0 | 3062 | tags=38%, list=5%, sign |
| GO ANTIGE | GO ANTIGEN | Details ... | 99 | 0.8 | 2.473 | 0 | 0 | 0 | 2352 | tags=48%, list=4%, sign |
| GO NEGATI | GO NEGATIV | Details ... | 133 | 0.67 | 2.471 | 0 | 0 | 0 | 4797 | tags=38%, list=8%, sign |
| GO NEGATI | GO NEGATIV | Details ... | 45 | 0.74 | 2.471 | 0 | 0 | 0 | 2916 | tags=38%, list=5%, sign |
| GO PHAGOC | GO PHAGOCY | Details ... | 75 | 0.76 | 2.47 | 0 | 0 | 0 | 3436 | tags=51%, list=6%, sign |
| GO REGULA | GO REGULAT | Details ... | 146 | 0.72 | 2.468 | 0 | 0 | 0 | 4639 | tags=50%, list=8%, sign |
| GO PROGRA | GO PROGRAM | Details ... | 49 | 0.73 | 2.461 | 0 | 0 | 0 | 6083 | tags=55%, list=10%, sig |
| GO NEGATI | GO NEGATIV | Details ... | 61 | 0.74 | 2.457 | 0 | 0 | 0 | 4992 | tags=51%, list=9%, sign |
| GO RESPON | GO RESPONS | Details ... | 308 | 0.6 | 2.452 | 0 | 0 | 0 | 6375 | tags=42%, list=11%, sig |
| GO POSITIV | GO POSITIVE | Details ... | 26 | 0.85 | 2.45 | 0 | 0 | 0 | 2307 | tags=54%, list=4%, sign |
| GO REGULA | GO REGULAT | Details ... | 151 | 0.67 | 2.449 | 0 | 0 | 0 | 5636 | tags=42%, list=10%, sig |
| GO POSITIV | GO POSITIVE | Details ... | 245 | 0.66 | 2.449 | 0 | 0 | 0 | 5145 | tags=42%, list=9%, sign |
| GO POSITIV | GO POSITIVE | Details ... | 208 | 0.67 | 2.443 | 0 | 0 | 0 | 5145 | tags=44%, list=9%, sign |
| GO NEGATI | GO NEGATIV | Details ... | 94 | 0.69 | 2.442 | 0 | 0 | 0 | 4992 | tags=45%, list=9%, sign |
| GO NECROT | GO NECROTI | Details ... | 61 | 0.67 | 2.442 | 0 | 0 | 0 | 6795 | tags=49%, list=12%, sig |
| GO NEGATI | GO NEGATIV | Details ... | 42 | 0.81 | 2.442 | 0 | 0 | 0 | 4639 | tags=60%, list=8%, sign |
| GO T CELL | GO T CELL A | Details ... | 109 | 0.7 | 2.437 | 0 | 0 | 0 | 3951 | tags=44%, list=7%, sign |
| GO ANTIGE | GO ANTIGEN | Details ... | 80 | 0.85 | 2.434 | 0 | 0 | 0 | 2352 | tags=54%, list=4%, sign |
| GO NEGATI | GO NEGATIV | Details ... | 61 | 0.68 | 2.431 | 0 | 0 | 0 | 5319 | tags=48%, list=9%, sign |
| GO REGULA | GO REGULAT | Details ... | 37 | 0.79 | 2.421 | 0 | 0 | 0 | 2307 | tags=46%, list=4%, sign |
| GO ANTIGE | GO ANTIGEN | Details ... | 228 | 0.7 | 2.42 | 0 | 0 | 0 | 3517 | tags=39%, list=6%, sign |
| GO REGULA | GO REGULAT | Details ... | 160 | 0.69 | 2.416 | 0 | 0 | 0 | 4639 | tags=44%, list=8%, sign |
| GO INTERFE | GO INTERFERON GAMM | | 112 | 0.74 | 2.415 | 0 | 0 | 0 | 6299 | tags=59%, list=11%, sig |
| GO T CELL | GO T CELL PROLIFERAT | | 191 | 0.68 | 2.415 | 0 | 0 | 0 | 3062 | tags=38%, list=5%, sign |
| GO NEGATI | GO NEGATIVE REGULA | | 48 | 0.75 | 2.411 | 0 | 0 | 0 | 4639 | tags=54%, list=8%, sign |
| GO POSITIV | GO POSITIVE REGULAT | | 374 | 0.61 | 2.408 | 0 | 0 | 0 | 6109 | tags=42%, list=10%, sig |
| GO ANTIGE | GO ANTIGEN RECEPTOR | | 306 | 0.72 | 2.405 | 0 | 0 | 0 | 6103 | tags=51%, list=10%, sig |
| GO REGULA | GO REGULATION OF DE | | 71 | 0.72 | 2.404 | 0 | 0 | 0 | 3254 | tags=41%, list=6%, sign |
| GO NEGATI | GO NEGATIVE REGULA | | 57 | 0.79 | 2.404 | 0 | 0 | 0 | 1550 | tags=49%, list=3%, sign |
| GO ACTIVA | GO ACTIVATION OF CY | | 84 | 0.62 | 2.401 | 0 | 0 | 0 | 6948 | tags=51%, list=12%, sig |
| GO REGULA | GO REGULATION OF T C | | 71 | 0.73 | 2.398 | 0 | 0 | 0 | 4639 | tags=45%, list=8%, sign |
| GO REGULA | GO REGULATION OF LE | | 202 | 0.68 | 2.396 | 0 | 0 | 0 | 4977 | tags=49%, list=9%, sign |
| GO POSITIV | GO POSITIVE REGULAT | | 98 | 0.73 | 2.396 | 0 | 0 | 0 | 4152 | tags=50%, list=7%, sign |
| GO ANTIGE | GO ANTIGEN PROCESSI | | 190 | 0.72 | 2.395 | 0 | 0 | 0 | 3473 | tags=41%, list=6%, sign |
| GO MYD88 I | GO MYD88 INDEPENDE | | 34 | 0.78 | 2.363 | 0 | ###### | 0.001 | 6083 | tags=74%, list=10%, sig |
| GO POSITIV | GO POSITIVE REGULAT | | 59 | 0.82 | 2.363 | 0 | ###### | 0.001 | 3623 | tags=58%, list=6%, sign |
| GO NEGATI | GO NEGATIVE REGULA | | 52 | 0.77 | 2.365 | 0 | ###### | 0.001 | 4639 | tags=58%, list=8%, sign |
| GO INTERLE | GO INTERLEUKIN 10 PR | | 61 | 0.73 | 2.368 | 0 | ###### | 0.001 | 5312 | tags=51%, list=9%, sign |
| GO NEGATI | GO NEGATIVE REGULA | | 44 | 0.76 | 2.37 | 0 | ###### | 0.001 | 952 | tags=36%, list=2%, sign |

| | | | | | | | | | | |
|---|---|---|---|---|---|---|---|---|---|---|
| GO REGULA | GO REGULATION OF AL | 96 | 0.71 | 2.37 | 0 | ###### | 0.001 | 3033 | tags=41%, list=5%, sign |
| GO T CELL M | GO T CELL MEDIATED C | 42 | 0.81 | 2.378 | 0 | ###### | 0.001 | 4639 | tags=60%, list=8%, sign |
| GO REGULA | GO REGULATION OF LE | 73 | 0.8 | 2.388 | 0 | ###### | 0.001 | 4977 | tags=62%, list=9%, sign |
| GO POSITIV | GO POSITIVE REGULATI | 93 | 0.67 | 2.389 | 0 | ###### | 0.001 | 4977 | tags=45%, list=9%, sign |
| GO NATURA | GO NATURAL KILLER C | 65 | 0.8 | 2.389 | 0 | ###### | 0.001 | 4152 | tags=65%, list=7%, sign |
| GO REGULA | GO REGULATION OF IM | 452 | 0.66 | 2.342 | 0 | ###### | 0.001 | 6328 | tags=48%, list=11%, sig |
| GO NEGATI | GO NEGATIVE REGULAT | 121 | 0.66 | 2.342 | 0 | ###### | 0.001 | 5890 | tags=44%, list=10%, sig |
| GO POSITIV | GO POSITIVE REGULATI | 64 | 0.77 | 2.343 | 0 | ###### | 0.001 | 3594 | tags=53%, list=6%, sign |
| GO REGULA | GO REGULATION OF RE | 190 | 0.6 | 2.343 | 0 | ###### | 0.001 | 7722 | tags=43%, list=13%, sig |
| GO NEGATI | GO NEGATIVE REGULAT | 54 | 0.67 | 2.343 | 0 | ###### | 0.001 | 2916 | tags=31%, list=5%, sign |
| GO REGULA | GO REGULATION OF PR | 136 | 0.63 | 2.348 | 0 | ###### | 0.001 | 4977 | tags=40%, list=9%, sign |
| GO POSITIV | GO POSITIVE REGULATI | 100 | 0.74 | 2.348 | 0 | ###### | 0.001 | 3033 | tags=42%, list=5%, sign |
| GO NIK NF K | GO NIK NF KAPPAB SIG | 181 | 0.62 | 2.349 | 0 | ###### | 0.001 | 5095 | tags=38%, list=9%, sign |
| GO NEGATI | GO NEGATIVE REGULAT | 83 | 0.67 | 2.35 | 0 | ###### | 0.001 | 3062 | tags=41%, list=5%, sign |
| GO POSITIV | GO POSITIVE REGULATI | 130 | 0.68 | 2.352 | 0 | ###### | 0.001 | 2648 | tags=43%, list=5%, sign |
| GO ALPHA B | GO ALPHA BETA T CELL | 145 | 0.68 | 2.341 | 0 | ###### | 0.001 | 5785 | tags=47%, list=10%, sig |
| GO TUMOR | GO TUMOR NECROSIS F | 46 | 0.72 | 2.341 | 0 | ###### | 0.001 | 7035 | tags=61%, list=12%, sig |
| GO REGULA | GO REGULATION OF NA | 44 | 0.86 | 2.333 | 0 | ###### | 0.001 | 4639 | tags=82%, list=8%, sign |
| GO T CELL A | GO T CELL ACTIVATION | 475 | 0.62 | 2.336 | 0 | ###### | 0.001 | 5856 | tags=43%, list=10%, sig |
| GO T CELL D | GO T CELL DIFFERENTIA | 71 | 0.71 | 2.337 | 0 | ###### | 0.001 | 3678 | tags=46%, list=6%, sign |
| GO REGULA | GO REGULATION OF T C | 325 | 0.64 | 2.337 | 0 | ###### | 0.001 | 3340 | tags=37%, list=6%, sign |
| GO MODULA | GO MODULATION BY SY | 44 | 0.74 | 2.337 | 0 | ###### | 0.001 | 6795 | tags=52%, list=12%, sig |
| GO LYMPHO | GO LYMPHOCYTE MEDI | 345 | 0.73 | 2.331 | 0 | ###### | 0.001 | 7314 | tags=60%, list=13%, sig |
| GO NUCLEO | GO NUCLEOSIDE CATAB | 31 | 0.74 | 2.332 | 0 | ###### | 0.001 | 9155 | tags=65%, list=16%, sig |
| GO REGULA | GO REGULATION OF T C | 32 | 0.77 | 2.333 | 0 | ###### | 0.001 | 2050 | tags=41%, list=4%, sign |
| GO CD4 POS | GO CD4 POSITIVE ALPH | 96 | 0.66 | 2.327 | 0 | ###### | 0.002 | 3062 | tags=40%, list=5%, sign |
| GO PROTEIN | GO PROTEIN ADP RIBOS | 32 | 0.72 | 2.328 | 0 | ###### | 0.002 | 3915 | tags=47%, list=7%, sign |
| GO NEGATI | GO NEGATIVE REGULAT | 436 | 0.6 | 2.329 | 0 | ###### | 0.002 | 6384 | tags=39%, list=11%, sig |
| GO REGULA | GO REGULATION OF VII | 37 | 0.77 | 2.33 | 0 | ###### | 0.002 | 1764 | tags=41%, list=3%, sign |
| GO REGULA | GO REGULATION OF NA | 34 | 0.79 | 2.321 | 0 | ###### | 0.003 | 3033 | tags=50%, list=5%, sign |
| GO REGULA | GO REGULATION OF TU | 57 | 0.65 | 2.323 | 0 | ###### | 0.003 | 7722 | tags=54%, list=13%, sig |
| GO POSITIV | GO POSITIVE REGULATI | 159 | 0.62 | 2.324 | 0 | ###### | 0.003 | 7035 | tags=51%, list=12%, sig |
| GO POSITIV | GO POSITIVE REGULATI | 54 | 0.72 | 2.325 | 0 | ###### | 0.003 | 2315 | tags=43%, list=4%, sign |
| GO IMMUNE | GO IMMUNE RESPONSE | 496 | 0.66 | 2.325 | 0 | ###### | 0.003 | 6131 | tags=46%, list=11%, sig |
| GO RESPON | GO RESPONSE TO INTEF | 206 | 0.6 | 2.304 | 0 | ###### | 0.004 | 5368 | tags=36%, list=9%, sign |
| GO REGULA | GO REGULATION OF CD | 62 | 0.68 | 2.307 | 0 | ###### | 0.004 | 2916 | tags=37%, list=5%, sign |
| GO POSITIV | GO POSITIVE REGULATI | 213 | 0.64 | 2.308 | 0 | ###### | 0.004 | 3075 | tags=37%, list=5%, sign |
| GO T CELL A | GO T CELL APOPTOTIC I | 49 | 0.68 | 2.31 | 0 | ###### | 0.004 | 3621 | tags=47%, list=6%, sign |
| GO REGULA | GO REGULATION OF LE | 236 | 0.64 | 2.311 | 0 | ###### | 0.004 | 3062 | tags=36%, list=5%, sign |
| GO INTERLE | GO INTERLEUKIN 17 PR | 43 | 0.73 | 2.312 | 0 | ###### | 0.004 | 7273 | tags=51%, list=12%, sig |
| GO PHAGOC | GO PHAGOCYTIC VESIC | 138 | 0.64 | 2.314 | 0 | ###### | 0.004 | 4392 | tags=41%, list=8%, sign |
| GO POSITIV | GO POSITIVE REGULATI | 450 | 0.6 | 2.298 | 0 | ###### | 0.005 | 4977 | tags=39%, list=9%, sign |
| GO REGULA | GO REGULATION OF CY | 82 | 0.68 | 2.299 | 0 | ###### | 0.005 | 2315 | tags=37%, list=4%, sign |
| GO POSITIV | GO POSITIVE REGULATI | 48 | 0.75 | 2.3 | 0 | ###### | 0.005 | 3033 | tags=40%, list=5%, sign |
| GO ALPHA B | GO ALPHA BETA T CELL | 35 | 0.78 | 2.3 | 0 | ###### | 0.005 | 5312 | tags=54%, list=9%, sign |
| GO NEGATI | GO NEGATIVE REGULAT | 39 | 0.7 | 2.297 | 0 | ###### | 0.006 | 2916 | tags=36%, list=5%, sign |
| GO REGULA | GO REGULATION OF T C | 42 | 0.75 | 2.291 | 0 | ###### | 0.006 | 5319 | tags=60%, list=9%, sign |
| GO REGULA | GO REGULATION OF T C | 148 | 0.64 | 2.292 | 0 | ###### | 0.006 | 3623 | tags=37%, list=6%, sign |
| GO LYMPHO | GO LYMPHOCYTE APOP | 73 | 0.64 | 2.297 | 0 | ###### | 0.006 | 3621 | tags=40%, list=6%, sign |
| GO ALPHA B | GO ALPHA BETA T CELL | 63 | 0.69 | 2.285 | 0 | ###### | 0.006 | 2916 | tags=43%, list=5%, sign |
| GO POSITIV | GO POSITIVE REGULATI | 102 | 0.69 | 2.285 | 0 | ###### | 0.006 | 4152 | tags=40%, list=7%, sign |
| GO RESPIRA | GO RESPIRATORY BURS | 34 | 0.79 | 2.285 | 0 | ###### | 0.006 | 6750 | tags=65%, list=12%, sig |
| GO T CELL C | GO T CELL CYTOKINE P | 43 | 0.71 | 2.288 | 0 | ###### | 0.006 | 3436 | tags=40%, list=6%, sign |
| GO RESPON | GO RESPONSE TO INTEF | 28 | 0.79 | 2.262 | 0 | ###### | 0.008 | 1015 | tags=46%, list=2%, sign |
| GO REGULA | GO REGULATION OF T C | 33 | 0.81 | 2.271 | 0 | ###### | 0.008 | 4639 | tags=55%, list=8%, sign |
| GO NEGATI | GO NEGATIVE REGULAT | 23 | 0.85 | 2.28 | 0 | ###### | 0.007 | 5319 | tags=70%, list=9%, sign |
| GO REGULA | GO REGULATION OF CD | 48 | 0.72 | 2.263 | 0 | ###### | 0.008 | 2916 | tags=40%, list=5%, sign |
| GO LEUKOC | GO LEUKOCYTE PROLIF | 309 | 0.61 | 2.271 | 0 | ###### | 0.008 | 5596 | tags=38%, list=10%, sig |

| | | | | | | | | | | |
|---|---|---|---|---|---|---|---|---|---|---|
| GO INTERLE | GO INTERLEUKIN 1 MEI | 103 | 0.67 | 2.283 | 0 | ###### | 0.007 | 7814 | tags=49%, list=13%, sig |
| GO POSITIV | GO POSITIVE REGULATI | 232 | 0.66 | 2.264 | 0 | ###### | 0.008 | 3188 | tags=40%, list=5%, sign |
| GO REGULA | GO REGULATION OF VII | 95 | 0.67 | 2.272 | 0 | ###### | 0.008 | 3263 | tags=37%, list=6%, sign |
| GO POSITIV | GO POSITIVE REGULATI | 20 | 0.83 | 2.279 | 0 | ###### | 0.008 | 3033 | tags=55%, list=5%, sign |
| GO ADAPTIV | GO ADAPTIVE IMMUNE | 349 | 0.71 | 2.264 | 0 | ###### | 0.008 | 7314 | tags=57%, list=13%, sig |
| GO INNATE | GO INNATE IMMUNE RE | 116 | 0.66 | 2.272 | 0 | ###### | 0.008 | 6872 | tags=45%, list=12%, sig |
| GO REGULA | GO REGULATION OF SY | 225 | 0.6 | 2.266 | 0 | ###### | 0.008 | 6065 | tags=39%, list=10%, sig |
| GO NEGATI | GO NEGATIVE REGULAT | 31 | 0.81 | 2.276 | 0 | ###### | 0.008 | 5319 | tags=58%, list=9%, sign |
| GO NEGATI | GO NEGATIVE REGULAT | 201 | 0.62 | 2.267 | 0 | ###### | 0.008 | 3331 | tags=34%, list=6%, sign |
| GO LYMPHO | GO LYMPHOCYTE ACTI | 181 | 0.63 | 2.277 | 0 | ###### | 0.008 | 4039 | tags=37%, list=7%, sign |
| GO POSITIV | GO POSITIVE REGULATI | 149 | 0.66 | 2.261 | 0 | ###### | 0.008 | 3033 | tags=37%, list=5%, sign |
| GO ACTIVA | GO ACTIVATION OF INN | 140 | 0.64 | 2.262 | 0 | ###### | 0.008 | 5312 | tags=39%, list=9%, sign |
| GO LEUKOC | GO LEUKOCYTE CELL C | 358 | 0.62 | 2.259 | 0 | ###### | 0.008 | 5319 | tags=41%, list=9%, sign |
| GO THREON | GO THREONINE TYPE PI | 31 | 0.81 | 2.26 | 0 | ###### | 0.008 | 3035 | tags=48%, list=5%, sign |
| GO LUMEN | GO LUMENAL SIDE OF M | 33 | 0.87 | 2.26 | 0 | ###### | 0.008 | 2478 | tags=61%, list=4%, sign |
| GO PATTER | GO PATTERN RECOGNIT | 194 | 0.61 | 2.257 | 0 | ###### | 0.01 | 7299 | tags=46%, list=13%, sig |
| GO TRANSF | GO TRANSFERASE ACTI | 44 | 0.62 | 2.257 | 0 | ###### | 0.01 | 4201 | tags=34%, list=7%, sign |
| GO POSITIV | GO POSITIVE REGULATI | 186 | 0.58 | 2.254 | 0 | ###### | 0.01 | 6083 | tags=41%, list=10%, sig |
| GO REGULA | GO REGULATION OF RE | 25 | 0.76 | 2.258 | 0 | ###### | 0.01 | 3625 | tags=48%, list=6%, sign |
| GO NEGATI | GO NEGATIVE REGULAT | 27 | 0.71 | 2.255 | 0 | ###### | 0.01 | 1638 | tags=33%, list=3%, sign |
| GO POSITIV | GO POSITIVE REGULATI | 54 | 0.66 | 2.255 | 0 | ###### | 0.01 | 6083 | tags=46%, list=10%, sig |
| GO RESPON | GO RESPONSE TO INTEF | 50 | 0.68 | 2.253 | 0 | ###### | 0.01 | 4625 | tags=36%, list=8%, sign |
| GO I KAPPA | GO I KAPPAB KINASE N | 282 | 0.55 | 2.251 | 0 | ###### | 0.011 | 4977 | tags=37%, list=9%, sign |
| GO TYPE 2 I | GO TYPE 2 IMMUNE RES | 39 | 0.69 | 2.246 | 0 | ###### | 0.013 | 3678 | tags=41%, list=6%, sign |
| GO CYTOKI | GO CYTOKINE PRODUCT | 102 | 0.63 | 2.246 | 0 | ###### | 0.013 | 2315 | tags=33%, list=4%, sign |
| GO CD4 POS | GO CD4 POSITIVE ALPH | 78 | 0.67 | 2.247 | 0 | ###### | 0.013 | 3062 | tags=41%, list=5%, sign |
| GO INTERLE | GO INTERLEUKIN 12 PR | 57 | 0.7 | 2.247 | 0 | ###### | 0.013 | 6386 | tags=49%, list=11%, sig |
| GO POSITIV | GO POSITIVE REGULATI | 195 | 0.53 | 2.239 | 0 | ###### | 0.015 | 7430 | tags=40%, list=13%, sig |
| GO LATE EN | GO LATE ENDOSOME M | 129 | 0.57 | 2.239 | 0 | ###### | 0.015 | 5939 | tags=41%, list=10%, sig |
| GO POSITIV | GO POSITIVE REGULATI | 147 | 0.54 | 2.24 | 0 | ###### | 0.015 | 7430 | tags=41%, list=13%, sig |
| GO RECYCL | GO RECYCLING ENDOS | 83 | 0.6 | 2.237 | 0 | ###### | 0.015 | 5164 | tags=37%, list=9%, sign |
| GO POSITIV | GO POSITIVE REGULATI | 274 | 0.62 | 2.242 | 0 | ###### | 0.015 | 3188 | tags=35%, list=5%, sign |
| GO ENDOCY | GO ENDOCYTIC VESICL | 160 | 0.62 | 2.236 | 0 | ###### | 0.015 | 4152 | tags=35%, list=7%, sign |
| GO INTERLE | GO INTERLEUKIN 4 PRO | 35 | 0.77 | 2.233 | 0 | ###### | 0.015 | 2987 | tags=54%, list=5%, sign |
| GO VIRAL R | GO VIRAL RELEASE FRO | 34 | 0.71 | 2.233 | 0 | ###### | 0.015 | 6065 | tags=47%, list=10%, sig |
| GO T CELL I | GO T CELL DIFFERENTIA | 251 | 0.61 | 2.229 | 0 | ###### | 0.015 | 3678 | tags=37%, list=6%, sign |
| GO REGULA | GO REGULATION OF TY | 31 | 0.72 | 2.227 | 0 | ###### | 0.015 | 4795 | tags=48%, list=8%, sign |
| GO DEFENS | GO DEFENSE RESPONSE | 90 | 0.62 | 2.227 | 0 | ###### | 0.015 | 8635 | tags=46%, list=15%, sig |
| GO PYRIMII | GO PYRIMIDINE NUCLE | 34 | 0.69 | 2.227 | 0 | ###### | 0.015 | 9155 | tags=62%, list=16%, sig |
| GO NEGATI | GO NEGATIVE REGULAT | 258 | 0.54 | 2.216 | 0 | ###### | 0.015 | 4992 | tags=31%, list=9%, sign |
| GO REGULA | GO REGULATION OF LY | 489 | 0.61 | 2.219 | 0 | ###### | 0.015 | 5919 | tags=43%, list=10%, sig |
| GO NEGATI | GO NEGATIVE REGULAT | 186 | 0.56 | 2.222 | 0 | ###### | 0.015 | 5416 | tags=33%, list=9%, sign |
| GO TOLL LI | GO TOLL LIKE RECEPTO | 150 | 0.62 | 2.219 | 0 | ###### | 0.015 | 7299 | tags=49%, list=13%, sig |
| GO MHC PR | GO MHC PROTEIN BIND | 36 | 0.88 | 2.22 | 0 | ###### | 0.015 | 1764 | tags=64%, list=3%, sign |
| GO REGULA | GO REGULATION OF PR | 26 | 0.75 | 2.214 | 0 | ###### | 0.015 | 1937 | tags=42%, list=3%, sign |
| GO REGULA | GO REGULATION OF LY | 179 | 0.61 | 2.211 | 0 | ###### | 0.015 | 3623 | tags=34%, list=6%, sign |
| GO CELLUL | GO CELLULAR RESPONS | 218 | 0.61 | 2.205 | 0 | ###### | 0.016 | 6064 | tags=41%, list=10%, sig |
| GO POSITIV | GO POSITIVE REGULATI | 335 | 0.64 | 2.206 | 0 | ###### | 0.016 | 6083 | tags=47%, list=10%, sig |
| GO CYTOKI | GO CYTOKINE RECEPTO | 260 | 0.57 | 2.209 | 0 | ###### | 0.016 | 6389 | tags=37%, list=11%, sig |
| GO TYPE I II | GO TYPE I INTERFERON | 121 | 0.63 | 2.206 | 0 | ###### | 0.016 | 3678 | tags=35%, list=6%, sign |
| GO NEGATI | GO NEGATIVE REGULAT | 24 | 0.85 | 2.202 | 0 | ###### | 0.018 | 5089 | tags=67%, list=9%, sign |
| GO THREON | GO THREONINE TYPE EI | 21 | 0.91 | 2.202 | 0 | ###### | 0.018 | 2770 | tags=67%, list=5%, sign |
| GO REGULA | GO REGULATORY T CEL | 34 | 0.76 | 2.204 | 0 | ###### | 0.017 | 4152 | tags=47%, list=7%, sign |
| GO POSITIV | GO POSITIVE REGULATI | 28 | 0.87 | 2.2 | 0 | ###### | 0.018 | 3033 | tags=82%, list=5%, sign |
| GO ALPHA E | GO ALPHA BETA T CELL | 106 | 0.65 | 2.203 | 0 | ###### | 0.018 | 3062 | tags=37%, list=5%, sign |
| GO PYRIMII | GO PYRIMIDINE NUCLE | 23 | 0.74 | 2.19 | 0 | ###### | 0.021 | 10152 | tags=70%, list=17%, sig |
| GO NEGATI | GO NEGATIVE REGULAT | 357 | 0.51 | 2.191 | 0 | ###### | 0.021 | 5947 | tags=30%, list=10%, sig |
| GO REGULA | GO REGULATION OF AL | 64 | 0.67 | 2.189 | 0 | ###### | 0.021 | 2916 | tags=36%, list=5%, sign |

| | | | | | | | | | |
|---|---|---|---|---|---|---|---|---|---|
| GO TUMOR | GO TUMOR NECROSIS F | 30 | 0.76 | 2.193 | 0 | ###### | 0.02 | 6975 | tags=67%, list=12%, sig |
| GO CELLUL. | GO CELLULAR DEFENSI | 52 | 0.76 | 2.192 | 0 | ###### | 0.021 | 4317 | tags=56%, list=7%, sign |
| GO POSITIV | GO POSITIVE REGULATI | 63 | 0.71 | 2.189 | 0 | ###### | 0.021 | 3033 | tags=41%, list=5%, sign |
| GO POSITIV | GO POSITIVE REGULATI | 26 | 0.83 | 2.194 | 0 | ###### | 0.02 | 3033 | tags=46%, list=5%, sign |
| GO REGULA | GO REGULATION OF CE | 430 | 0.56 | 2.188 | 0 | ###### | 0.021 | 5416 | tags=35%, list=9%, sign |
| GO POSITIV | GO POSITIVE REGULATI | 30 | 0.76 | 2.189 | 0 | ###### | 0.021 | 2916 | tags=43%, list=5%, sign |
| GO REGULA | GO REGULATION OF CY | 234 | 0.51 | 2.187 | 0 | ###### | 0.021 | 7829 | tags=38%, list=13%, sig |
| GO NLRP3 II | GO NLRP3 INFLAMMAS( | 16 | 0.77 | 2.187 | 0 | ###### | 0.021 | 2198 | tags=44%, list=4%, sign |
| GO REGULA | GO REGULATION OF LY | 56 | 0.65 | 2.187 | 0 | ###### | 0.021 | 3621 | tags=41%, list=6%, sign |
| GO REGULA | GO REGULATION OF HE | 464 | 0.53 | 2.184 | 0 | ###### | 0.024 | 6389 | tags=35%, list=11%, sig |
| GO REGULA | GO REGULATION OF PA | 93 | 0.61 | 2.183 | 0 | ###### | 0.025 | 6065 | tags=41%, list=10%, sig |
| GO POSITIV | GO POSITIVE REGULATI | 56 | 0.68 | 2.183 | 0 | ###### | 0.026 | 2052 | tags=34%, list=4%, sign |
| GO MYELOI | GO MYELOID DENDRITI | 29 | 0.79 | 2.183 | 0 | ###### | 0.026 | 3953 | tags=55%, list=7%, sign |
| GO ER TO G | GO ER TO GOLGI TRANS | 59 | 0.72 | 2.18 | 0 | ###### | 0.027 | 2619 | tags=37%, list=4%, sign |
| GO NADPLU | GO NADPLUS ADP RIBO | 24 | 0.71 | 2.181 | 0 | ###### | 0.027 | 3915 | tags=42%, list=7%, sign |
| GO IMMUNI | GO IMMUNE RECEPTOR | 132 | 0.68 | 2.181 | 0 | ###### | 0.027 | 4457 | tags=49%, list=8%, sign |
| GO ACTIVA | GO ACTIVATED T CELL | 46 | 0.63 | 2.179 | 0 | ###### | 0.028 | 2711 | tags=33%, list=5%, sign |
| GO CELLUL. | GO CELLULAR RESPONS | 240 | 0.59 | 2.175 | 0 | ###### | 0.028 | 6064 | tags=39%, list=10%, sig |
| GO IMMUNI | GO IMMUNE RESPONSE | 19 | 0.81 | 2.176 | 0 | ###### | 0.028 | 4639 | tags=68%, list=8%, sign |
| GO POSITIV | GO POSITIVE REGULATI | 394 | 0.63 | 2.174 | 0 | ###### | 0.029 | 6083 | tags=46%, list=10%, sig |
| GO T HELPE | GO T HELPER 1 TYPE IM | 44 | 0.73 | 2.177 | 0 | ###### | 0.028 | 3678 | tags=50%, list=6%, sign |
| GO GLYCOS | GO GLYCOSYL COMPOU | 41 | 0.65 | 2.176 | 0 | ###### | 0.028 | 9155 | tags=56%, list=16%, sig |
| GO ANTIGEI | GO ANTIGEN PROCESSI | 24 | 0.91 | 2.168 | 0 | ###### | 0.03 | 856 | tags=58%, list=1%, sign |
| GO POSITIV | GO POSITIVE REGULATI | 104 | 0.63 | 2.168 | 0 | ###### | 0.03 | 3623 | tags=37%, list=6%, sign |
| GO IMMUN( | GO IMMUNOGLOBULIN | 21 | 0.8 | 2.167 | 0 | ###### | 0.03 | 2354 | tags=43%, list=4%, sign |
| GO MHC CL. | GO MHC CLASS I PROTE | 19 | 0.88 | 2.17 | 0 | ###### | 0.03 | 1536 | tags=58%, list=3%, sign |
| GO MHC PR( | GO MHC PROTEIN COMI | 24 | 0.88 | 2.169 | 0 | ###### | 0.03 | 1586 | tags=67%, list=3%, sign |
| GO PRODUC | GO PRODUCTION OF MC | 296 | 0.64 | 2.168 | 0 | ###### | 0.03 | 6737 | tags=48%, list=12%, sig |
| GO INTERLI | GO INTERLEUKIN 1 PRO | 117 | 0.62 | 2.165 | 0 | ###### | 0.032 | 5216 | tags=42%, list=9%, sign |
| GO POSITIV | GO POSITIVE REGULATI | 71 | 0.61 | 2.161 | 0 | ###### | 0.033 | 3273 | tags=30%, list=6%, sign |
| GO POSITIV | GO POSITIVE REGULATI | 57 | 0.72 | 2.16 | 0 | ###### | 0.034 | 5926 | tags=58%, list=10%, sig |
| GO NEGATI | GO NEGATIVE REGULA | 17 | 0.89 | 2.161 | 0 | ###### | 0.033 | 4639 | tags=82%, list=8%, sign |
| GO INTERLI | GO INTERLEUKIN 1 BET | 103 | 0.63 | 2.162 | 0 | ###### | 0.032 | 5216 | tags=43%, list=9%, sign |
| GO PEPTIDE | GO PEPTIDE ANTIGEN B | 30 | 0.94 | 2.159 | 0 | ###### | 0.034 | 1461 | tags=70%, list=3%, sign |
| GO SCF DEP | GO SCF DEPENDENT PR | 91 | 0.64 | 2.159 | 0 | ###### | 0.034 | 3078 | tags=32%, list=5%, sign |
| GO CELLUL. | GO CELLULAR RESPONS | 60 | 0.62 | 2.157 | 0 | ###### | 0.038 | 4792 | tags=35%, list=8%, sign |
| GO CELLUL. | GO CELLULAR METABO | 32 | 0.66 | 2.157 | 0 | ###### | 0.038 | 8789 | tags=56%, list=15%, sig |
| GO T HELPE | GO T HELPER 17 TYPE II | 31 | 0.74 | 2.156 | 0 | ###### | 0.04 | 2916 | tags=45%, list=5%, sign |
| GO REGULA | GO REGULATION OF LE | 287 | 0.56 | 2.155 | 0 | ###### | 0.044 | 6389 | tags=38%, list=11%, sig |
| GO POSITIV | GO POSITIVE REGULATI | 23 | 0.88 | 2.153 | 0 | ###### | 0.044 | 3033 | tags=83%, list=5%, sign |
| GO POSITIV | GO POSITIVE REGULATI | 36 | 0.73 | 2.152 | 0 | ###### | 0.045 | 2916 | tags=42%, list=5%, sign |
| GO REGULA | GO REGULATION OF T H | 36 | 0.71 | 2.15 | 0 | ###### | 0.046 | 2916 | tags=39%, list=5%, sign |
| GO LUMEN/ | GO LUMENAL SIDE OF E | 27 | 0.9 | 2.15 | 0 | ###### | 0.046 | 2478 | tags=70%, list=4%, sign |
| GO POSITIV | GO POSITIVE T CELL SE | 36 | 0.81 | 2.15 | 0 | ###### | 0.046 | 4279 | tags=64%, list=7%, sign |
| GO RESPON | GO RESPONSE TO TUMC | 25 | 0.72 | 2.146 | 0 | ###### | 0.051 | 4639 | tags=56%, list=8%, sign |
| GO CYSTEIN | GO CYSTEINE TYPE ENI | 15 | 0.84 | 2.147 | 0 | ###### | 0.051 | 597 | tags=53%, list=1%, sign |
| GO NEGATI | GO NEGATIVE REGULA | 22 | 0.75 | 2.147 | 0 | ###### | 0.051 | 5143 | tags=50%, list=9%, sign |
| GO REGULA | GO REGULATION OF TR | 75 | 0.66 | 2.144 | 0 | ###### | 0.053 | 6103 | tags=43%, list=10%, sig |
| GO DEFENS. | GO DEFENSE RESPONSE | 318 | 0.6 | 2.14 | 0 | ###### | 0.058 | 8773 | tags=48%, list=15%, sig |
| GO POSITIV | GO POSITIVE REGULATI | 24 | 0.81 | 2.141 | 0 | ###### | 0.058 | 2512 | tags=63%, list=4%, sign |
| GO INTERFI | GO INTERFERON BETA I | 49 | 0.65 | 2.138 | 0 | ###### | 0.06 | 3254 | tags=37%, list=6%, sign |
| GO LYMPH( | GO LYMPHOCYTE COST | 58 | 0.7 | 2.136 | 0 | ###### | 0.062 | 2552 | tags=43%, list=4%, sign |
| GO POSITIV | GO POSITIVE REGULATI | 158 | 0.59 | 2.136 | 0 | ###### | 0.063 | 5416 | tags=37%, list=9%, sign |
| GO PROTEA | GO PROTEASOMAL UBI( | 22 | 0.86 | 2.134 | 0 | ###### | 0.065 | 2770 | tags=64%, list=5%, sign |
| GO TOLERA | GO TOLERANCE INDUCT | 24 | 0.85 | 2.135 | 0 | ###### | 0.065 | 937 | tags=54%, list=2%, sign |
| GO VIRAL G | GO VIRAL GENOME REF | 121 | 0.6 | 2.133 | 0 | ###### | 0.065 | 3263 | tags=31%, list=6%, sign |
| GO CELLUL. | GO CELLULAR RESPONS | 20 | 0.77 | 2.133 | 0 | ###### | 0.068 | 2122 | tags=55%, list=4%, sign |
| GO POSITIV | GO POSITIVE REGULATI | 92 | 0.65 | 2.132 | 0 | ###### | 0.068 | 4795 | tags=43%, list=8%, sign |

| | | | | | | | | | |
|---|---|---|---|---|---|---|---|---|---|
| GO NEGATI' | GO NEGATIVE REGULA' | 30 | 0.68 | 2.13 | 0 | ###### | 0.069 | 2916 | tags=33%, list=5%, sign |
| GO REGULA | GO REGULATION OF CD | 17 | 0.83 | 2.13 | 0 | ###### | 0.069 | 2916 | tags=59%, list=5%, sign |
| GO REGULA | GO REGULATION OF TO | 72 | 0.63 | 2.129 | 0 | ###### | 0.069 | 7299 | tags=47%, list=13%, sig |
| GO T CELL I | GO T CELL RECEPTOR C | 125 | 0.87 | 2.13 | 0 | ###### | 0.069 | 3220 | tags=77%, list=6%, sign |
| GO FC RECE | GO FC RECEPTOR SIGN/ | 239 | 0.63 | 2.128 | 0 | ###### | 0.069 | 6131 | tags=45%, list=11%, sig |
| GO MOVEM | GO MOVEMENT IN HOS' | 165 | 0.54 | 2.128 | 0 | ###### | 0.069 | 7367 | tags=38%, list=13%, sig |
| GO RESPON | GO RESPONSE TO MOLE | 348 | 0.54 | 2.126 | 0 | ###### | 0.07 | 6161 | tags=36%, list=11%, sig |
| GO TUMOR | GO TUMOR NECROSIS F | 178 | 0.58 | 2.123 | 0 | ###### | 0.071 | 4795 | tags=36%, list=8%, sign |
| GO RESPON | GO RESPONSE TO INTEF | 20 | 0.81 | 2.122 | 0 | ###### | 0.074 | 1015 | tags=50%, list=2%, sign |
| GO NEGATI' | GO NEGATIVE REGULA' | 20 | 0.87 | 2.121 | 0 | ###### | 0.077 | 4639 | tags=75%, list=8%, sign |
| GO REGULA | GO REGULATION OF T C | 34 | 0.67 | 2.121 | 0 | ###### | 0.077 | 3621 | tags=47%, list=6%, sign |
| GO POSITIV | GO POSITIVE REGULATI | 20 | 0.74 | 2.119 | 0 | ###### | 0.081 | 2050 | tags=40%, list=4%, sign |
| GO NEGATI' | GO NEGATIVE REGULA' | 36 | 0.65 | 2.118 | 0 | ###### | 0.082 | 5890 | tags=42%, list=10%, sig |
| GO POSITIV | GO POSITIVE REGULATI | 35 | 0.68 | 2.119 | 0 | ###### | 0.081 | 5926 | tags=51%, list=10%, sig |
| GO NUCLEO | GO NUCLEOSIDE SALV/ | 15 | 0.8 | 2.117 | 0 | ###### | 0.082 | 8789 | tags=73%, list=15%, sig |
| GO INTERA( | GO INTERACTION WITH | 208 | 0.53 | 2.117 | 0 | ###### | 0.082 | 7367 | tags=37%, list=13%, sig |
| GO FC EPSII | GO FC EPSILON RECEPT | 168 | 0.66 | 2.116 | 0 | ###### | 0.084 | 7851 | tags=52%, list=13%, sig |
| GO NEGATI' | GO NEGATIVE REGULA' | 89 | 0.55 | 2.113 | 0 | ###### | 0.085 | 7080 | tags=43%, list=12%, sig |
| GO LEUKOC | GO LEUKOCYTE APOPT | 107 | 0.58 | 2.113 | 0 | ###### | 0.085 | 5081 | tags=38%, list=9%, sign |
| GO CD8 POS | GO CD8 POSITIVE ALPH | 26 | 0.77 | 2.108 | 0 | ###### | 0.089 | 2916 | tags=46%, list=5%, sign |
| GO REGULA | GO REGULATION OF NE | 38 | 0.61 | 2.106 | 0 | ###### | 0.09 | 1937 | tags=29%, list=3%, sign |
| GO TERTIAF | GO TERTIARY GRANULI | 55 | 0.62 | 2.107 | 0 | ###### | 0.09 | 4842 | tags=44%, list=8%, sign |
| GO NUCLEO | GO NUCLEOBASE CONT | 48 | 0.59 | 2.103 | 0 | 0.001 | 0.091 | 9289 | tags=52%, list=16%, sig |
| GO POSITIV | GO POSITIVE REGULATI | 152 | 0.56 | 2.103 | 0 | 0.001 | 0.091 | 6027 | tags=38%, list=10%, sig |
| GO REGULA | GO REGULATION OF IM | 61 | 0.62 | 2.103 | 0 | 0.001 | 0.091 | 5890 | tags=41%, list=10%, sig |
| GO T CELL S | GO T CELL SELECTION | 49 | 0.74 | 2.1 | 0 | 0.001 | 0.096 | 7430 | tags=67%, list=13%, sig |
| GO NEGATI' | GO NEGATIVE REGULA' | 31 | 0.64 | 2.1 | 0 | 0.001 | 0.096 | 3621 | tags=42%, list=6%, sign |
| GO INTRINS | GO INTRINSIC COMPON | 154 | 0.57 | 2.099 | 0 | 0.0011 | 0.098 | 5351 | tags=31%, list=9%, sign |
| GO B CELL I | GO B CELL MEDIATED I | 210 | 0.72 | 2.097 | 0 | 0.0011 | 0.102 | 7314 | tags=60%, list=13%, sig |
| GO NEGATI' | GO NEGATIVE REGULA' | 36 | 0.66 | 2.095 | 0 | 0.0011 | 0.102 | 8426 | tags=56%, list=14%, sig |
| GO REGULA | GO REGULATION OF CY | 17 | 0.75 | 2.094 | 0 | 0.0011 | 0.104 | 2630 | tags=41%, list=5%, sign |
| GO PLASMA | GO PLASMA MEMBRAN | 291 | 0.64 | 2.095 | 0 | 0.0011 | 0.103 | 3254 | tags=44%, list=6%, sign |
| GO TYROSII | GO TYROSINE PHOSPHO | 89 | 0.58 | 2.092 | 0 | 0.0011 | 0.109 | 3273 | tags=28%, list=6%, sign |
| GO POSITIV | GO POSITIVE REGULATI | 68 | 0.66 | 2.089 | 0 | 0.0011 | 0.112 | 5307 | tags=46%, list=9%, sign |
| GO NEGATI' | GO NEGATIVE REGULA' | 19 | 0.79 | 2.089 | 0 | 0.0012 | 0.114 | 5785 | tags=63%, list=10%, sig |
| GO REGULA | GO REGULATION OF B C | 49 | 0.65 | 2.088 | 0 | 0.0012 | 0.115 | 7243 | tags=49%, list=12%, sig |
| GO CHEMOI | GO CHEMOKINE RECEP' | 63 | 0.69 | 2.082 | 0 | 0.0013 | 0.126 | 4110 | tags=40%, list=7%, sign |
| GO VIRAL L | GO VIRAL LIFE CYCLE | 330 | 0.53 | 2.081 | 0 | 0.0013 | 0.127 | 6485 | tags=34%, list=11%, sig |
| GO LYMPHO | GO LYMPHOCYTE CHEM | 63 | 0.67 | 2.08 | 0 | 0.0013 | 0.127 | 4110 | tags=46%, list=7%, sign |
| GO POSITIV | GO POSITIVE REGULATI | 18 | 0.81 | 2.078 | 0 | 0.0013 | 0.127 | 6605 | tags=67%, list=11%, sig |
| GO ENDOPE | GO ENDOPEPTIDASE CO | 68 | 0.72 | 2.074 | 0 | 0.0014 | 0.134 | 3994 | tags=38%, list=7%, sign |
| GO CYTOPL | GO CYTOPLASMIC PATT | 63 | 0.59 | 2.074 | 0 | 0.0014 | 0.134 | 7270 | tags=43%, list=12%, sig |
| GO REGULA | GO REGULATION OF AN | 67 | 0.68 | 2.073 | 0 | 0.0014 | 0.137 | 5319 | tags=43%, list=9%, sign |
| GO INFLAM | GO INFLAMMASOME CO | 16 | 0.81 | 2.073 | 0 | 0.0014 | 0.135 | 2238 | tags=50%, list=4%, sign |
| GO IMMUNO | GO IMMUNOGLOBULIN | 202 | 0.66 | 2.072 | 0 | 0.0014 | 0.137 | 6737 | tags=51%, list=12%, sig |
| GO POSITIV | GO POSITIVE REGULATI | 39 | 0.64 | 2.073 | 0 | 0.0014 | 0.137 | 4039 | tags=41%, list=7%, sign |
| GO ZYMOGI | GO ZYMOGEN ACTIVAT | 55 | 0.63 | 2.07 | 0 | 0.0015 | 0.143 | 8621 | tags=45%, list=15%, sig |
| GO REGULA | GO REGULATION OF DN | 111 | 0.6 | 2.069 | 0 | 0.0015 | 0.147 | 7705 | tags=42%, list=13%, sig |
| GO REGULA | GO REGULATION OF HY | 20 | 0.64 | 2.066 | 0 | 0.0015 | 0.15 | 7462 | tags=50%, list=13%, sig |
| GO REGULA | GO REGULATION OF T H | 22 | 0.73 | 2.065 | 0 | 0.0015 | 0.15 | 2916 | tags=41%, list=5%, sign |
| GO HUMOR. | GO HUMORAL IMMUNE | 352 | 0.62 | 2.065 | 0 | 0.0016 | 0.15 | 8001 | tags=48%, list=14%, sig |
| GO T CELL I | GO T CELL HOMEOSTAS | 38 | 0.65 | 2.064 | 0 | 0.0016 | 0.154 | 6691 | tags=55%, list=11%, sig |
| GO POSITIV | GO POSITIVE REGULATI | 16 | 0.77 | 2.063 | 0 | 0.0016 | 0.156 | 2043 | tags=50%, list=3%, sign |
| GO LYMPHO | GO LYMPHOCYTE DIFFI | 364 | 0.55 | 2.062 | 0 | 0.0016 | 0.158 | 4279 | tags=32%, list=7%, sign |
| GO CD4 POS | GO CD4 POSITIVE ALPH | 23 | 0.69 | 2.063 | 0 | 0.0016 | 0.158 | 3033 | tags=39%, list=5%, sign |
| GO TOLL LII | GO TOLL LIKE RECEPTO | 20 | 0.74 | 2.061 | 0 | 0.0016 | 0.159 | 7273 | tags=70%, list=12%, sig |
| GO ENTRY I | GO ENTRY INTO HOST | 143 | 0.54 | 2.056 | 0 | 0.0018 | 0.168 | 7367 | tags=38%, list=13%, sig |
| GO RIBONU | GO RIBONUCLEOSIDE C | 21 | 0.7 | 2.055 | 0 | 0.0018 | 0.169 | 8764 | tags=57%, list=15%, sig |

| | | | | | | | | | |
|---|---|---|---|---|---|---|---|---|---|
| GO PROTEA | GO PROTEASOME CORE | 20 | 0.89 | 2.052 | 0 | 0.0018 | 0.172 | 2770 | tags=70%, list=5%, sign |
| GO POSITIV | GO POSITIVE REGULATI | 37 | 0.65 | 2.049 | 0 | 0.0019 | 0.184 | 6795 | tags=49%, list=12%, sig |
| GO CLATHR | GO CLATHRIN COATED | 57 | 0.62 | 2.048 | 0 | 0.0019 | 0.187 | 3159 | tags=32%, list=5%, sign |
| GO INTERLI | GO INTERLEUKIN 8 PRO | 101 | 0.54 | 2.047 | 0 | 0.002 | 0.19 | 6083 | tags=35%, list=10%, sig |
| GO CYTOKI | GO CYTOKINE SECRETI | 128 | 0.58 | 2.04 | 0 | 0.0021 | 0.202 | 5312 | tags=41%, list=9%, sign |
| GO POSITIV | GO POSITIVE REGULATI | 42 | 0.62 | 2.036 | 0 | 0.0022 | 0.208 | 1939 | tags=31%, list=3%, sign |
| GO NEGATI | GO NEGATIVE REGULA | 48 | 0.61 | 2.036 | 0 | 0.0022 | 0.209 | 6299 | tags=48%, list=11%, sig |
| GO POSITIV | GO POSITIVE REGULATI | 37 | 0.67 | 2.037 | 0 | 0.0022 | 0.208 | 5312 | tags=46%, list=9%, sign |
| GO T CELL I | GO T CELL LINEAGE CO | 28 | 0.7 | 2.034 | 0 | 0.0023 | 0.218 | 7430 | tags=64%, list=13%, sig |
| GO NEGATI | GO NEGATIVE REGULA | 18 | 0.75 | 2.034 | 0 | 0.0023 | 0.218 | 1638 | tags=33%, list=3%, sign |
| GO ACTIVA | GO ACTIVATION OF CYS | 15 | 0.76 | 2.031 | 0 | 0.0023 | 0.222 | 7263 | tags=60%, list=12%, sig |
| GO REGULA | GO REGULATION OF RE | 16 | 0.75 | 2.03 | 0 | 0.0024 | 0.224 | 6665 | tags=63%, list=11%, sig |
| GO MYELOI | GO MYELOID DENDRITI | 18 | 0.75 | 2.028 | 0 | 0.0024 | 0.229 | 3897 | tags=50%, list=7%, sign |
| GO ANAPHA | GO ANAPHASE PROMOT | 82 | 0.69 | 2.025 | 0 | 0.0025 | 0.236 | 7798 | tags=48%, list=13%, sig |
| GO ENDOSC | GO ENDOSOME MEMBR | 484 | 0.48 | 2.025 | 0 | 0.0025 | 0.236 | 4686 | tags=29%, list=8%, sign |
| GO ANTIGEI | GO ANTIGEN BINDING | 152 | 0.8 | 2.023 | 0 | 0.0025 | 0.237 | 6303 | tags=64%, list=11%, sig |
| GO INTERLI | GO INTERLEUKIN 6 PRO | 165 | 0.56 | 2.024 | 0 | 0.0025 | 0.237 | 6299 | tags=38%, list=11%, sig |
| GO EOSINOI | GO EOSINOPHIL MIGRA | 22 | 0.79 | 2.024 | 0 | 0.0025 | 0.237 | 3400 | tags=50%, list=6%, sign |
| GO NEGATI | GO NEGATIVE REGULA | 18 | 0.78 | 2.022 | 0 | 0.0025 | 0.239 | 1718 | tags=33%, list=3%, sign |
| GO PEPTIDE | GO PEPTIDE TRANSMEM | 30 | 0.64 | 2.022 | 0 | 0.0025 | 0.239 | 2875 | tags=23%, list=5%, sign |
| GO T HELPE | GO T HELPER 2 CELL DI | 15 | 0.72 | 2.022 | 0 | 0.0025 | 0.239 | 2043 | tags=40%, list=3%, sign |
| GO REGULA | GO REGULATION OF LE | 85 | 0.58 | 2.02 | 0 | 0.0026 | 0.247 | 3621 | tags=35%, list=6%, sign |
| GO EXTRINS | GO EXTRINSIC APOPTO | 226 | 0.49 | 2.018 | 0 | 0.0026 | 0.25 | 6800 | tags=35%, list=12%, sig |
| GO POSITIV | GO POSITIVE REGULATI | 202 | 0.53 | 2.015 | 0 | 0.0028 | 0.256 | 5416 | tags=33%, list=9%, sign |
| GO CYTOPL | GO CYTOPLASMIC PATT | 28 | 0.65 | 2.012 | 0 | 0.0028 | 0.262 | 7270 | tags=46%, list=12%, sig |
| GO MHC CL | GO MHC CLASS II PROT | 16 | 0.86 | 2.011 | 0 | 0.0029 | 0.266 | 3139 | tags=63%, list=5%, sign |
| GO I KAPPA | GO I KAPPAB PHOSPHO | 20 | 0.72 | 2.009 | 0 | 0.0029 | 0.27 | 6845 | tags=55%, list=12%, sig |
| GO REGULA | GO REGULATION OF TO | 16 | 0.84 | 2.008 | 0 | 0.003 | 0.273 | 937 | tags=56%, list=2%, sign |
| GO CHEMOI | GO CHEMOKINE ACTIVI | 47 | 0.72 | 2.007 | 0 | 0.003 | 0.273 | 4110 | tags=45%, list=7%, sign |
| GO REGULA | GO REGULATION OF INI | 412 | 0.48 | 2.005 | 0 | 0.0031 | 0.276 | 5619 | tags=32%, list=10%, sig |
| GO NEGATI | GO NEGATIVE REGULA | 52 | 0.58 | 2.005 | 0 | 0.0031 | 0.276 | 4374 | tags=38%, list=7%, sign |
| GO REGULA | GO REGULATION OF B C | 176 | 0.65 | 2.004 | 0 | 0.0031 | 0.278 | 7877 | tags=54%, list=13%, sig |
| GO REGULA | GO REGULATION OF NII | 116 | 0.53 | 2.003 | 0 | 0.0032 | 0.279 | 4938 | tags=34%, list=8%, sign |
| GO EOSINOI | GO EOSINOPHIL CHEMC | 18 | 0.84 | 2 | 0 | 0.0032 | 0.286 | 3400 | tags=50%, list=6%, sign |
| GO REGULA | GO REGULATION OF SY | 28 | 0.72 | 1.997 | 0 | 0.0034 | 0.294 | 3679 | tags=43%, list=6%, sign |
| GO NEGATI | GO NEGATIVE REGULA | 15 | 0.73 | 1.995 | 0 | 0.0035 | 0.303 | 4795 | tags=40%, list=8%, sign |
| GO RECEPT | GO RECEPTOR SIGNALI | 174 | 0.5 | 1.994 | 0 | 0.0035 | 0.305 | 4938 | tags=28%, list=8%, sign |
| GO ANTIMI | GO ANTIMICROBIAL HU | 66 | 0.63 | 1.993 | 0 | 0.0035 | 0.309 | 8971 | tags=50%, list=15%, sig |
| GO CELLUL | GO CELLULAR RESPONS | 20 | 0.7 | 1.992 | 0 | 0.0035 | 0.312 | 3698 | tags=45%, list=6%, sign |
| GO REGULA | GO REGULATION OF MY | 20 | 0.74 | 1.99 | 0 | 0.0036 | 0.316 | 3679 | tags=45%, list=6%, sign |
| GO ANTIGEI | GO ANTIGEN PROCESSI | 17 | 0.94 | 1.988 | 0 | 0.0037 | 0.326 | 856 | tags=76%, list=1%, sign |
| GO REGULA | GO REGULATION OF PE | 450 | 0.46 | 1.985 | 0 | 0.0038 | 0.329 | 7829 | tags=34%, list=13%, sig |
| GO NATURA | GO NATURAL KILLER C | 31 | 0.64 | 1.983 | 0 | 0.0039 | 0.331 | 3951 | tags=35%, list=7%, sign |
| GO NEGATI | GO NEGATIVE REGULA | 25 | 0.66 | 1.98 | 0 | 0.004 | 0.338 | 2916 | tags=32%, list=5%, sign |
| GO PRODUC | GO PRODUCTION OF MC | 90 | 0.53 | 1.977 | 0 | 0.0041 | 0.344 | 6027 | tags=38%, list=10%, sig |
| GO POSITIV | GO POSITIVE REGULATI | 18 | 0.72 | 1.977 | 0 | 0.0041 | 0.347 | 3340 | tags=50%, list=6%, sign |
| GO POSITIV | GO POSITIVE REGULATI | 21 | 0.8 | 1.972 | 0 | 0.0043 | 0.36 | 2361 | tags=48%, list=4%, sign |
| GO AMINE M | GO AMINE METABOLIC | 138 | 0.51 | 1.972 | 0 | 0.0044 | 0.36 | 6782 | tags=33%, list=12%, sig |
| GO POSITIV | GO POSITIVE REGULATI | 356 | 0.45 | 1.97 | 0 | 0.0044 | 0.365 | 7430 | tags=34%, list=13%, sig |
| GO TOLL LI | GO TOLL LIKE RECEPTC | 25 | 0.67 | 1.967 | 0 | 0.0045 | 0.367 | 3930 | tags=40%, list=7%, sign |
| GO NEGATI | GO NEGATIVE REGULA | 21 | 0.69 | 1.963 | 0 | 0.0047 | 0.378 | 5947 | tags=38%, list=10%, sig |
| GO CHEMOI | GO CHEMOKINE C X C M | 15 | 0.7 | 1.963 | 0 | 0.0047 | 0.378 | 7614 | tags=47%, list=13%, sig |
| GO POSITIV | GO POSITIVE REGULATI | 93 | 0.55 | 1.959 | 0 | 0.0049 | 0.384 | 4938 | tags=32%, list=8%, sign |
| GO ANTIMI | GO ANTIMICROBIAL HU | 125 | 0.56 | 1.957 | 0 | 0.0049 | 0.386 | 10480 | tags=46%, list=18%, sig |
| GO NEGATI | GO NEGATIVE REGULA | 19 | 0.7 | 1.955 | 0 | 0.005 | 0.391 | 6083 | tags=63%, list=10%, sig |
| GO HUMOR | GO HUMORAL IMMUNE | 142 | 0.76 | 1.953 | 0 | 0.0051 | 0.397 | 7872 | tags=66%, list=13%, sig |
| GO POSITIV | GO POSITIVE REGULATI | 71 | 0.56 | 1.951 | 0 | 0.0052 | 0.4 | 6196 | tags=41%, list=11%, sig |
| GO ENDOCY | GO ENDOCYTIC VESICL | 308 | 0.49 | 1.947 | 0 | 0.0054 | 0.415 | 4795 | tags=30%, list=8%, sign |

| | | | | | | | | | |
|---|---|---|---|---|---|---|---|---|---|
| GO MHC PR( | GO MHC PROTEIN COMI | 23 | 0.96 | 1.947 | 0 | 0.0054 | 0.415 | 1500 | tags=96%, list=3%, sign |
| GO CYTOKII | GO CYTOKINE PRODUC' | 67 | 0.55 | 1.945 | 0 | 0.0055 | 0.419 | 5216 | tags=36%, list=9%, sign |
| GO ACTIVA' | GO ACTIVATION OF NF | 17 | 0.71 | 1.946 | 0 | 0.0055 | 0.419 | 3003 | tags=41%, list=5%, sign |
| GO POSITIV | GO POSITIVE REGULATI | 177 | 0.46 | 1.941 | 0 | 0.0057 | 0.428 | 7615 | tags=39%, list=13%, sig |
| GO FICOLIN | GO FICOLIN 1 RICH GRA | 124 | 0.53 | 1.933 | 0 | 0.0062 | 0.45 | 4842 | tags=38%, list=8%, sign |
| GO POSITIV | GO POSITIVE REGULATI | 267 | 0.48 | 1.933 | 0 | 0.0062 | 0.452 | 7243 | tags=39%, list=12%, sig |
| GO ACTIVA' | GO ACTIVATION OF JAN | 15 | 0.74 | 1.933 | 0 | 0.0062 | 0.452 | 3483 | tags=47%, list=6%, sign |
| GO RESPON | GO RESPONSE TO INTEF | 35 | 0.58 | 1.929 | 0 | 0.0065 | 0.462 | 3678 | tags=31%, list=6%, sign |
| GO EARLY E | GO EARLY ENDOSOME I | 152 | 0.5 | 1.924 | 0 | 0.0068 | 0.479 | 3740 | tags=23%, list=6%, sign |
| GO B CELL A | GO B CELL APOPTOTIC I | 26 | 0.61 | 1.921 | 0 | 0.0069 | 0.486 | 2630 | tags=31%, list=5%, sign |
| GO DEOXYF | GO DEOXYRIBONUCLE( | 15 | 0.75 | 1.91 | 0 | 0.0078 | 0.508 | 7852 | tags=60%, list=13%, sig |
| GO KILLING | GO KILLING OF CELLS ( | 63 | 0.57 | 1.901 | 0 | 0.0085 | 0.535 | 8971 | tags=46%, list=15%, sig |
| GO LATE EN | GO LATE ENDOSOME | 263 | 0.45 | 1.886 | 0 | 0.0097 | 0.571 | 5939 | tags=30%, list=10%, sig |
| GO NEGATI' | GO NEGATIVE REGULA' | 83 | 0.47 | 1.886 | 0 | 0.0097 | 0.571 | 7662 | tags=34%, list=13%, sig |
| GO REGULA | GO REGULATION OF MY | 122 | 0.5 | 1.873 | 0 | 0.0111 | 0.616 | 6389 | tags=34%, list=11%, sig |
| GO HYDROI | GO HYDROLASE ACTIVI | 34 | 0.57 | 1.87 | 0 | 0.0113 | 0.622 | 4524 | tags=35%, list=8%, sign |
| GO REGULA | GO REGULATION OF DN | 444 | 0.43 | 1.862 | 0 | 0.0125 | 0.643 | 7243 | tags=34%, list=12%, sig |
| GO CYSTEIN | GO CYSTEINE TYPE ENI | 41 | 0.55 | 1.859 | 0 | 0.0127 | 0.651 | 6860 | tags=39%, list=12%, sig |
| GO REGULA | GO REGULATION OF RE | 151 | 0.46 | 1.856 | 0 | 0.0131 | 0.662 | 4938 | tags=27%, list=8%, sign |
| GO PEPTIDA | GO PEPTIDASE ACTIVA' | 18 | 0.6 | 1.842 | 0 | 0.0148 | 0.698 | 5115 | tags=44%, list=9%, sign |
| GO DEAMIN | GO DEAMINASE ACTIVI | 30 | 0.58 | 1.841 | 0 | 0.015 | 0.702 | 4524 | tags=37%, list=8%, sign |
| GO NEGATI' | GO NEGATIVE REGULA' | 92 | 0.48 | 1.839 | 0 | 0.0152 | 0.703 | 7829 | tags=34%, list=13%, sig |
| GO CELLUL | GO CELLULAR KETONE | 253 | 0.44 | 1.839 | 0 | 0.0152 | 0.705 | 6795 | tags=32%, list=12%, sig |
| GO POSITIV | GO POSITIVE REGULATI | 26 | 0.57 | 1.836 | 0 | 0.0155 | 0.712 | 6083 | tags=35%, list=10%, sig |
| GO CYSTEIN | GO CYSTEINE TYPE PEP | 137 | 0.48 | 1.835 | 0 | 0.0155 | 0.713 | 6375 | tags=28%, list=11%, sig |
| GO PEPTIDE | GO PEPTIDE BINDING | 299 | 0.44 | 1.835 | 0 | 0.0156 | 0.715 | 4903 | tags=25%, list=8%, sign |
| GO VESICLE | GO VESICLE LUMEN | 322 | 0.46 | 1.828 | 0 | 0.0164 | 0.723 | 6500 | tags=34%, list=11%, sig |
| GO REGULA | GO REGULATION OF CE | 187 | 0.45 | 1.826 | 0 | 0.0165 | 0.728 | 6795 | tags=30%, list=12%, sig |
| GO INTRINS | GO INTRINSIC COMPON | 377 | 0.44 | 1.821 | 0 | 0.0172 | 0.739 | 6323 | tags=28%, list=11%, sig |
| GO RECYCL | GO RECYCLING ENDOS( | 180 | 0.44 | 1.812 | 0 | 0.0187 | 0.762 | 6267 | tags=31%, list=11%, sig |
| GO MULTIV | GO MULTIVESICULAR E | 59 | 0.49 | 1.771 | 0 | 0.0261 | 0.845 | 4279 | tags=29%, list=7%, sign |
| GO ENDOPE | GO ENDOPEPTIDASE AC | 430 | 0.39 | 1.733 | 0 | 0.0347 | 0.904 | 6588 | tags=26%, list=11%, sig |
| GO NAD BIC | GO NAD BIOSYNTHETIC | 27 | 0.51 | 1.645 | 0 | 0.0614 | 0.979 | 4542 | tags=30%, list=8%, sign |
| GO REGULA | GO REGULATION OF HU | 132 | 0.67 | 1.899 | 0.002 | 0.0087 | 0.541 | 7314 | tags=55%, list=13%, sig |
| GO PHAGOC | GO PHAGOCYTOSIS | 362 | 0.58 | 1.958 | 0.002 | 0.0049 | 0.385 | 6328 | tags=44%, list=11%, sig |
| GO NEUTRO | GO NEUTROPHIL CHEM( | 98 | 0.61 | 1.981 | 0.002 | 0.004 | 0.335 | 6161 | tags=42%, list=11%, sig |
| GO POSITIV | GO POSITIVE REGULATI | 132 | 0.65 | 1.892 | 0.002 | 0.0093 | 0.559 | 7877 | tags=56%, list=13%, sig |
| GO POSITIV | GO POSITIVE REGULATI | 33 | 0.64 | 1.898 | 0.002 | 0.0087 | 0.546 | 5416 | tags=45%, list=9%, sign |
| GO DENDRI' | GO DENDRITIC CELL DI | 44 | 0.7 | 2.098 | 0.002 | 0.0011 | 0.1 | 6299 | tags=55%, list=11%, sig |
| GO CD4 POS | GO CD4 POSITIVE OR CI | 22 | 0.75 | 2.012 | 0.002 | 0.0028 | 0.261 | 7133 | tags=68%, list=12%, sig |
| GO POSITIV | GO POSITIVE REGULATI | 221 | 0.44 | 1.848 | 0.002 | 0.0141 | 0.681 | 5820 | tags=29%, list=10%, sig |
| GO NEGATI' | GO NEGATIVE REGULA' | 23 | 0.64 | 1.968 | 0.002 | 0.0044 | 0.366 | 5319 | tags=48%, list=9%, sign |
| GO POSITIV | GO POSITIVE REGULATI | 16 | 0.72 | 1.836 | 0.002 | 0.0154 | 0.71 | 5045 | tags=44%, list=9%, sign |
| GO FC RECE | GO FC RECEPTOR MEDI | 143 | 0.64 | 1.898 | 0.002 | 0.0087 | 0.545 | 6719 | tags=52%, list=12%, sig |
| GO NEUTRO | GO NEUTROPHIL MIGRA | 117 | 0.61 | 1.996 | 0.002 | 0.0034 | 0.299 | 5820 | tags=43%, list=10%, sig |
| GO PROTEIN | GO PROTEIN ADP RIBOS | 16 | 0.76 | 1.979 | 0.002 | 0.004 | 0.339 | 3915 | tags=50%, list=7%, sign |
| GO REGULA | GO REGULATION OF MC | 22 | 0.71 | 2.037 | 0.002 | 0.0022 | 0.208 | 2052 | tags=32%, list=4%, sign |
| GO COPII CC | GO COPII COATED ER T( | 91 | 0.62 | 2.018 | 0.002 | 0.0026 | 0.25 | 4459 | tags=34%, list=8%, sign |
| GO NEGATI' | GO NEGATIVE REGULA' | 22 | 0.68 | 2.012 | 0.002 | 0.0028 | 0.263 | 725 | tags=27%, list=1%, sign |
| GO CYTOKII | GO CYTOKINE ACTIVIT' | 232 | 0.49 | 1.848 | 0.002 | 0.0141 | 0.681 | 6195 | tags=30%, list=11%, sig |
| GO NEGATI' | GO NEGATIVE REGULA' | 175 | 0.39 | 1.702 | 0.002 | 0.043 | 0.937 | 7080 | tags=30%, list=12%, sig |
| GO NEGATI' | GO NEGATIVE REGULA' | 76 | 0.52 | 1.83 | 0.002 | 0.0161 | 0.721 | 5546 | tags=32%, list=9%, sign |
| GO NEGATI' | GO NEGATIVE REGULA' | 104 | 0.56 | 2.137 | 0.002 | ###### | 0.062 | 3263 | tags=28%, list=6%, sign |
| GO INFLAM | GO INFLAMMATORY RE | 63 | 0.59 | 1.925 | 0.002 | 0.0067 | 0.474 | 7314 | tags=40%, list=13%, sig |
| GO REGULA | GO REGULATION OF PEI | 423 | 0.38 | 1.683 | 0.002 | 0.0477 | 0.956 | 4629 | tags=23%, list=8%, sign |
| GO REGULA | GO REGULATION OF CE | 80 | 0.64 | 2.07 | 0.002 | 0.0015 | 0.143 | 6782 | tags=44%, list=12%, sig |
| GO MACROI | GO MACROPHAGE ACTI | 97 | 0.63 | 2.064 | 0.002 | 0.0016 | 0.154 | 5312 | tags=43%, list=9%, sign |
| GO POSITIV | GO POSITIVE REGULATI | 22 | 0.69 | 1.955 | 0.002 | 0.005 | 0.393 | 2043 | tags=32%, list=3%, sign |

| | | | | | | | | | |
|---|---|---|---|---|---|---|---|---|---|
| GO SUPERO | GO SUPEROXIDE ANION | 35 | 0.6 | 1.883 | 0.002 | 0.01 | 0.586 | 6444 | tags=51%, list=11%, sig |
| GO CHEMOI | GO CHEMOKINE BIOSYI | 16 | 0.65 | 1.801 | 0.002 | 0.0204 | 0.79 | 5876 | tags=50%, list=10%, sig |
| GO INTERLI | GO INTERLEUKIN 2 PRO | 54 | 0.64 | 2.042 | 0.002 | 0.0021 | 0.198 | 3436 | tags=37%, list=6%, sign |
| GO POSITIV | GO POSITIVE REGULATI | 60 | 0.6 | 2.015 | 0.002 | 0.0028 | 0.256 | 5312 | tags=37%, list=9%, sign |
| GO NEGATI | GO NEGATIVE REGULAT | 24 | 0.68 | 1.988 | 0.002 | 0.0037 | 0.325 | 5785 | tags=46%, list=10%, sig |
| GO CLATHR | GO CLATHRIN COATED | 38 | 0.67 | 1.948 | 0.002 | 0.0053 | 0.415 | 4653 | tags=42%, list=8%, sign |
| GO IMMUN( | GO IMMUNOLOGICAL S | 38 | 0.67 | 1.928 | 0.002 | 0.0065 | 0.467 | 3379 | tags=45%, list=6%, sign |
| GO POSITIV | GO POSITIVE REGULATI | 58 | 0.54 | 1.79 | 0.002 | 0.0221 | 0.811 | 3238 | tags=29%, list=6%, sign |
| GO PEPTIDA | GO PEPTIDASE REGULA | 221 | 0.41 | 1.595 | 0.002 | 0.0825 | 0.992 | 7209 | tags=29%, list=12%, sig |
| GO LEUKOC | GO LEUKOCYTE ACTIV | 48 | 0.73 | 2.137 | 0.002 | ###### | 0.062 | 4938 | tags=52%, list=8%, sign |
| GO REGULA | GO REGULATION OF LE | 46 | 0.61 | 1.982 | 0.002 | 0.0039 | 0.335 | 6769 | tags=54%, list=12%, sig |
| GO INTERLI | GO INTERLEUKIN 13 PR | 21 | 0.67 | 1.973 | 0.002 | 0.0043 | 0.357 | 4359 | tags=48%, list=7%, sign |
| GO MYD88 I | GO MYD88 DEPENDENT | 36 | 0.68 | 2.069 | 0.002 | 0.0015 | 0.147 | 8635 | tags=61%, list=15%, sig |
| GO CYTOKI | GO CYTOKINE RECEPTC | 97 | 0.61 | 1.972 | 0.002 | 0.0043 | 0.358 | 8001 | tags=53%, list=14%, sig |
| GO LIPOPOI | GO LIPOPOLYSACCHAR | 58 | 0.59 | 1.945 | 0.002 | 0.0055 | 0.419 | 5876 | tags=47%, list=10%, sig |
| GO MIDBOD | GO MIDBODY ABSCISSI | 17 | 0.69 | 1.938 | 0.002 | 0.0059 | 0.437 | 5636 | tags=47%, list=10%, sig |
| GO ANTIGEI | GO ANTIGEN PROCESSI | 100 | 0.57 | 1.914 | 0.002 | 0.0074 | 0.5 | 4390 | tags=32%, list=8%, sign |
| GO REGULA | GO REGULATION OF ISC | 31 | 0.6 | 1.899 | 0.002 | 0.0086 | 0.539 | 3991 | tags=39%, list=7%, sign |
| GO SPECIFI | GO SPECIFIC GRANULE | 62 | 0.56 | 1.897 | 0.002 | 0.0089 | 0.549 | 4998 | tags=39%, list=9%, sign |
| GO REGULA | GO REGULATION OF CE | 63 | 0.71 | 2.091 | 0.002 | 0.0011 | 0.11 | 3994 | tags=40%, list=7%, sign |
| GO REGULA | GO REGULATION OF PH | 95 | 0.62 | 2.056 | 0.002 | 0.0018 | 0.167 | 5312 | tags=44%, list=9%, sign |
| GO NEGATI | GO NEGATIVE REGULAT | 143 | 0.5 | 2 | 0.002 | 0.0032 | 0.284 | 6384 | tags=31%, list=11%, sig |
| GO INTRINS | GO INTRINSIC APOPTOT | 101 | 0.47 | 1.8 | 0.002 | 0.0203 | 0.79 | 7681 | tags=40%, list=13%, sig |
| GO POSITIV | GO POSITIVE REGULATI | 47 | 0.67 | 2.098 | 0.002 | 0.0011 | 0.102 | 2916 | tags=36%, list=5%, sign |
| GO TERTIAI | GO TERTIARY GRANULI | 160 | 0.56 | 2.048 | 0.002 | 0.0019 | 0.185 | 4859 | tags=39%, list=8%, sign |
| GO SPECIFI | GO SPECIFIC GRANULE | 158 | 0.52 | 1.949 | 0.002 | 0.0053 | 0.405 | 4998 | tags=37%, list=9%, sign |
| GO NEGATI | GO NEGATIVE REGULAT | 30 | 0.54 | 1.9 | 0.002 | 0.0086 | 0.536 | 5045 | tags=43%, list=9%, sign |
| GO INTERFI | GO INTERFERON ALPHA | 27 | 0.68 | 1.96 | 0.002 | 0.0048 | 0.383 | 1068 | tags=30%, list=2%, sign |
| GO MACROI | GO MACROPHAGE CHEI | 40 | 0.6 | 1.939 | 0.002 | 0.0058 | 0.434 | 4317 | tags=38%, list=7%, sign |
| GO NEGATI | GO NEGATIVE REGULAT | 21 | 0.67 | 1.891 | 0.002 | 0.0093 | 0.562 | 2916 | tags=33%, list=5%, sign |
| GO OXIDOR | GO OXIDOREDUCTASE | 19 | 0.67 | 1.821 | 0.002 | 0.0172 | 0.739 | 8581 | tags=58%, list=15%, sig |
| GO NEGATI | GO NEGATIVE REGULAT | 292 | 0.43 | 1.828 | 0.002 | 0.0164 | 0.723 | 6069 | tags=27%, list=10%, sig |
| GO LYMPH( | GO LYMPHOCYTE MIGR | 113 | 0.61 | 1.979 | 0.002 | 0.004 | 0.339 | 6434 | tags=47%, list=11%, sig |
| GO POSITIV | GO POSITIVE REGULATI | 21 | 0.66 | 1.98 | 0.002 | 0.004 | 0.339 | 5838 | tags=33%, list=10%, sig |
| GO CELLUL | GO CELLULAR RESPONS | 15 | 0.76 | 1.965 | 0.002 | 0.0046 | 0.372 | 2122 | tags=53%, list=4%, sign |
| GO TRANS ( | GO TRANS GOLGI NETW | 86 | 0.52 | 1.923 | 0.002 | 0.0068 | 0.479 | 4952 | tags=30%, list=8%, sign |
| GO NEGATI | GO NEGATIVE REGULAT | 17 | 0.69 | 1.872 | 0.002 | 0.0112 | 0.618 | 2916 | tags=35%, list=5%, sign |
| GO REGULA | GO REGULATION OF HE | 86 | 0.59 | 1.967 | 0.002 | 0.0045 | 0.369 | 3131 | tags=27%, list=5%, sign |
| GO B CELL | GO B CELL ACTIVATION | 307 | 0.57 | 1.928 | 0.002 | 0.0065 | 0.467 | 7939 | tags=45%, list=14%, sig |
| GO CELLUL | GO CELLULAR MODIFIE | 48 | 0.55 | 1.88 | 0.002 | 0.0103 | 0.593 | 5400 | tags=33%, list=9%, sign |
| GO NEGATI | GO NEGATIVE REGULAT | 15 | 0.78 | 2.053 | 0.002 | 0.0018 | 0.172 | 6065 | tags=53%, list=10%, sig |
| GO INFLAM | GO INFLAMMATORY CE | 21 | 0.68 | 1.949 | 0.002 | 0.0053 | 0.406 | 4795 | tags=43%, list=8%, sign |
| GO REACTI | GO REACTIVE OXYGEN | 274 | 0.41 | 1.722 | 0.002 | 0.0374 | 0.918 | 5619 | tags=28%, list=10%, sig |
| GO TUMOR | GO TUMOR NECROSIS F | 47 | 0.58 | 1.888 | 0.002 | 0.0096 | 0.569 | 4124 | tags=32%, list=7%, sign |
| GO REGULA | GO REGULATION OF RE | 15 | 0.75 | 1.949 | 0.002 | 0.0053 | 0.411 | 4639 | tags=60%, list=8%, sign |
| GO KILLING | GO KILLING OF CELLS I | 28 | 0.61 | 1.768 | 0.002 | 0.0266 | 0.85 | 10123 | tags=50%, list=17%, sig |
| GO POSITIV | GO POSITIVE REGULATI | 30 | 0.66 | 2.014 | 0.002 | 0.0028 | 0.259 | 2002 | tags=33%, list=3%, sign |
| GO DEOXYI | GO DEOXYRIBONUCLE( | 17 | 0.71 | 1.922 | 0.002 | 0.0069 | 0.482 | 2242 | tags=35%, list=4%, sign |
| GO REGULA | GO REGULATION OF HE | 73 | 0.63 | 1.994 | 0.002 | 0.0035 | 0.305 | 3131 | tags=32%, list=5%, sign |
| GO POSITIV | GO POSITIVE REGULATI | 37 | 0.66 | 1.925 | 0.002 | 0.0067 | 0.475 | 6093 | tags=51%, list=10%, sig |
| GO NEGATI | GO NEGATIVE REGULAT | 41 | 0.58 | 1.868 | 0.002 | 0.0117 | 0.632 | 3003 | tags=32%, list=5%, sign |
| GO POSITIV | GO POSITIVE REGULATI | 79 | 0.51 | 1.831 | 0.002 | 0.0161 | 0.721 | 4938 | tags=34%, list=8%, sign |
| GO CYSTEII | GO CYSTEINE TYPE ENI | 114 | 0.47 | 1.768 | 0.002 | 0.0266 | 0.85 | 6375 | tags=26%, list=11%, sig |
| GO POSITIV | GO POSITIVE REGULATI | 86 | 0.6 | 1.995 | 0.002 | 0.0035 | 0.304 | 5312 | tags=42%, list=9%, sign |
| GO PEPTIDA | GO PEPTIDASE ACTIVAT | 37 | 0.6 | 1.979 | 0.002 | 0.004 | 0.339 | 5115 | tags=35%, list=9%, sign |
| GO NADPLU | GO NADPLUS NUCLEOS | 16 | 0.71 | 1.841 | 0.002 | 0.0149 | 0.702 | 3466 | tags=50%, list=6%, sign |
| GO RESPON | GO RESPONSE TO DSRN | 51 | 0.54 | 1.823 | 0.002 | 0.0169 | 0.735 | 8377 | tags=47%, list=14%, sig |
| GO NEGATI | GO NEGATIVE REGULAT | 422 | 0.43 | 1.861 | 0.002 | 0.0125 | 0.645 | 4992 | tags=24%, list=9%, sign |

| | | | | | | | | | |
|---|---|---|---|---|---|---|---|---|---|
| GO REGULA | GO REGULATION OF RE | 191 | 0.42 | 1.732 | 0.002 | 0.0349 | 0.905 | 5619 | tags=30%, list=10%, sig |
| GO CYTOKI | GO CYTOKINE METABO | 73 | 0.57 | 1.943 | 0.002 | 0.0056 | 0.424 | 2705 | tags=32%, list=5%, sign |
| GO NUCLEO | GO NUCLEOSIDE METAI | 99 | 0.49 | 1.871 | 0.002 | 0.0112 | 0.619 | 10245 | tags=48%, list=18%, sig |
| GO POSITIV | GO POSITIVE REGULATI | 21 | 0.63 | 1.764 | 0.002 | 0.0274 | 0.859 | 10532 | tags=62%, list=18%, sig |
| GO POSITIV | GO POSITIVE REGULATI | 54 | 0.51 | 1.722 | 0.002 | 0.0374 | 0.918 | 6444 | tags=39%, list=11%, sig |
| GO NEGATI | GO NEGATIVE REGULAT | 43 | 0.59 | 1.93 | 0.002 | 0.0064 | 0.46 | 7299 | tags=47%, list=13%, sig |
| GO REGULA | GO REGULATION OF RE | 47 | 0.54 | 1.873 | 0.002 | 0.0111 | 0.616 | 7601 | tags=43%, list=13%, sig |
| GO INTRINS | GO INTRINSIC APOPTOT | 280 | 0.42 | 1.717 | 0.002 | 0.0386 | 0.926 | 7681 | tags=35%, list=13%, sig |
| GO INTRINS | GO INTRINSIC APOPTOT | 44 | 0.55 | 1.881 | 0.004 | 0.0102 | 0.592 | 7681 | tags=48%, list=13%, sig |
| GO REGULA | GO REGULATION OF T H | 27 | 0.71 | 1.928 | 0.004 | 0.0065 | 0.467 | 3436 | tags=44%, list=6%, sign |
| GO CCR CH | GO CCR CHEMOKINE RE | 41 | 0.71 | 2.002 | 0.004 | 0.0032 | 0.281 | 4110 | tags=46%, list=7%, sign |
| GO T CELL | GO T CELL CHEMOTAXI | 28 | 0.68 | 1.949 | 0.004 | 0.0053 | 0.411 | 2542 | tags=36%, list=4%, sign |
| GO DEATH | GO DEATH RECEPTOR B | 19 | 0.64 | 1.904 | 0.004 | 0.0083 | 0.521 | 6375 | tags=53%, list=11%, sig |
| GO T HELPE | GO T HELPER 1 CELL DI | 19 | 0.67 | 1.859 | 0.004 | 0.0128 | 0.652 | 1318 | tags=42%, list=2%, sign |
| GO GOLGI A | GO GOLGI ASSOCIATED | 107 | 0.55 | 1.957 | 0.004 | 0.0049 | 0.386 | 4459 | tags=30%, list=8%, sign |
| GO REGULA | GO REGULATION OF LY | 27 | 0.67 | 1.98 | 0.004 | 0.004 | 0.339 | 3483 | tags=44%, list=6%, sign |
| GO VIRION | GO VIRION ASSEMBLY | 40 | 0.54 | 1.793 | 0.004 | 0.0215 | 0.802 | 5777 | tags=45%, list=10%, sig |
| GO ANTIBA | GO ANTIBACTERIAL HU | 49 | 0.6 | 1.802 | 0.004 | 0.0201 | 0.78 | 9849 | tags=45%, list=17%, sig |
| GO HEPATO | GO HEPATOCYTE GROW | 16 | 0.6 | 1.744 | 0.004 | 0.0319 | 0.89 | 6471 | tags=50%, list=11%, sig |
| GO ENDOSO | GO ENDOSOME ORGANI | 75 | 0.51 | 1.85 | 0.004 | 0.0139 | 0.676 | 7629 | tags=43%, list=13%, sig |
| GO THYMO | GO THYMOCYTE APOPT | 17 | 0.63 | 1.85 | 0.004 | 0.0139 | 0.677 | 2894 | tags=41%, list=5%, sign |
| GO REGULA | GO REGULATION OF TY | 35 | 0.61 | 1.97 | 0.004 | 0.0044 | 0.365 | 1735 | tags=29%, list=3%, sign |
| GO EXTERN | GO EXTERNAL SIDE OF | 401 | 0.58 | 1.978 | 0.004 | 0.0041 | 0.343 | 3878 | tags=34%, list=7%, sign |
| GO POSITIV | GO POSITIVE REGULATI | 26 | 0.67 | 1.856 | 0.004 | 0.0131 | 0.661 | 2361 | tags=35%, list=4%, sign |
| GO REGULA | GO REGULATION OF DE | 29 | 0.65 | 1.943 | 0.004 | 0.0056 | 0.424 | 3967 | tags=38%, list=7%, sign |
| GO GLIAL C | GO GLIAL CELL ACTIVA | 58 | 0.63 | 1.937 | 0.004 | 0.0059 | 0.439 | 4938 | tags=45%, list=8%, sign |
| GO POSITIV | GO POSITIVE REGULATI | 24 | 0.68 | 1.932 | 0.004 | 0.0062 | 0.453 | 6795 | tags=54%, list=12%, sig |
| GO T HELPE | GO T HELPER CELL LINI | 16 | 0.76 | 1.901 | 0.004 | 0.0086 | 0.535 | 6605 | tags=69%, list=11%, sig |
| GO POSITIV | GO POSITIVE REGULATI | 28 | 0.56 | 1.851 | 0.004 | 0.0138 | 0.674 | 3623 | tags=32%, list=6%, sign |
| GO AMIDE T | GO AMIDE TRANSMEMBI | 49 | 0.48 | 1.694 | 0.004 | 0.0449 | 0.948 | 4959 | tags=22%, list=8%, sign |
| GO DEFENS | GO DEFENSE RESPONSE | 78 | 0.49 | 1.752 | 0.004 | 0.0299 | 0.872 | 8971 | tags=37%, list=15%, sig |
| GO POSITIV | GO POSITIVE REGULATI | 37 | 0.62 | 1.876 | 0.004 | 0.0107 | 0.606 | 3483 | tags=38%, list=6%, sign |
| GO NEGATI | GO NEGATIVE REGULAT | 54 | 0.5 | 1.826 | 0.004 | 0.0165 | 0.728 | 7903 | tags=44%, list=14%, sig |
| GO POSITIV | GO POSITIVE REGULATI | 60 | 0.52 | 1.846 | 0.004 | 0.0144 | 0.689 | 5636 | tags=35%, list=10%, sig |
| GO POSITIV | GO POSITIVE REGULATI | 102 | 0.48 | 1.811 | 0.004 | 0.0187 | 0.762 | 5546 | tags=37%, list=9%, sign |
| GO AMIDE B | GO AMIDE BINDING | 368 | 0.4 | 1.699 | 0.004 | 0.0436 | 0.94 | 4903 | tags=23%, list=8%, sign |
| GO IMMUNO | GO IMMUNOGLOBULIN | 55 | 0.59 | 1.892 | 0.004 | 0.0092 | 0.559 | 4039 | tags=35%, list=7%, sign |
| GO POSITIV | GO POSITIVE REGULATI | 21 | 0.6 | 1.84 | 0.004 | 0.0151 | 0.703 | 4852 | tags=38%, list=8%, sign |
| GO NON ME | GO NON MEMBRANE SP | 46 | 0.56 | 1.829 | 0.004 | 0.0163 | 0.723 | 7949 | tags=46%, list=14%, sig |
| GO T CELL I | GO T CELL DIFFERENTI | 75 | 0.51 | 1.83 | 0.004 | 0.0161 | 0.721 | 2519 | tags=28%, list=4%, sign |
| GO PEPTIDA | GO PEPTIDASE COMPLE | 90 | 0.63 | 2.004 | 0.004 | 0.0031 | 0.278 | 2770 | tags=28%, list=5%, sign |
| GO TRANSL | GO TRANSLESION SYNT | 39 | 0.68 | 1.97 | 0.004 | 0.0044 | 0.365 | 9018 | tags=56%, list=15%, sig |
| GO PURINE | GO PURINE CONTAININ | 15 | 0.7 | 1.909 | 0.004 | 0.0079 | 0.512 | 8546 | tags=67%, list=15%, sig |
| GO POSITIV | GO POSITIVE REGULATI | 25 | 0.65 | 1.975 | 0.004 | 0.0042 | 0.349 | 2359 | tags=44%, list=4%, sign |
| GO MACROP | GO MACROPHAGE ACTI | 15 | 0.74 | 1.855 | 0.004 | 0.0132 | 0.662 | 2086 | tags=53%, list=4%, sign |
| GO MACROP | GO MACROPHAGE DIFF | 47 | 0.61 | 1.885 | 0.004 | 0.0099 | 0.578 | 2629 | tags=32%, list=5%, sign |
| GO LYMPHO | GO LYMPHOCYTE HOM | 62 | 0.64 | 2.033 | 0.004 | 0.0023 | 0.218 | 6691 | tags=47%, list=11%, sig |
| GO HYDROL | GO HYDROLASE ACTIVI | 34 | 0.52 | 1.737 | 0.004 | 0.0337 | 0.898 | 5989 | tags=41%, list=10%, sig |
| GO SUPERO | GO SUPEROXIDE METAI | 73 | 0.48 | 1.733 | 0.004 | 0.0348 | 0.903 | 6665 | tags=37%, list=11%, sig |
| GO RELEAS | GO RELEASE OF CYTOC | 58 | 0.53 | 1.918 | 0.004 | 0.0071 | 0.491 | 6251 | tags=38%, list=11%, sig |
| GO DETECT | GO DETECTION OF EXTI | 24 | 0.73 | 1.874 | 0.004 | 0.011 | 0.611 | 2983 | tags=42%, list=5%, sign |
| GO HEMATO | GO HEMATOPOIETIC ST | 87 | 0.58 | 1.985 | 0.004 | 0.0038 | 0.328 | 5095 | tags=33%, list=9%, sign |
| GO PYRIMI | GO PYRIMIDINE CONTA | 38 | 0.51 | 1.777 | 0.004 | 0.0249 | 0.833 | 10152 | tags=55%, list=17%, sig |
| GO COMPLE | GO COMPLEMENT ACTI | 165 | 0.66 | 1.801 | 0.006 | 0.0202 | 0.789 | 7872 | tags=58%, list=13%, sig |
| GO NUCLEO | GO NUCLEOTIDE BINDI | 36 | 0.57 | 1.837 | 0.006 | 0.0155 | 0.709 | 10512 | tags=58%, list=18%, sig |
| GO CHEMOK | GO CHEMOKINE PRODU | 93 | 0.53 | 1.963 | 0.006 | 0.0047 | 0.378 | 5876 | tags=34%, list=10%, sig |
| GO SECRET | GO SECRETORY GRANU | 296 | 0.46 | 1.84 | 0.006 | 0.0151 | 0.703 | 7668 | tags=36%, list=13%, sig |
| GO POSITIV | GO POSITIVE REGULATI | 138 | 0.54 | 1.92 | 0.006 | 0.007 | 0.488 | 6386 | tags=42%, list=11%, sig |

| | | | | | | | | | |
|---|---|---|---|---|---|---|---|---|---|
| GO CXCR CI | GO CXCR CHEMOKINE I | 17 | 0.79 | 1.908 | 0.006 | 0.008 | 0.516 | 2542 | tags=35%, list=4%, sign |
| GO POSITIV | GO POSITIVE REGULATI | 67 | 0.6 | 2.011 | 0.006 | 0.0029 | 0.265 | 5619 | tags=43%, list=10%, sig |
| GO LEUKOC | GO LEUKOCYTE CHEMC | 222 | 0.53 | 1.925 | 0.006 | 0.0067 | 0.473 | 6386 | tags=36%, list=11%, sig |
| GO NUCLEC | GO NUCLEOTIDE RECEF | 21 | 0.69 | 1.826 | 0.006 | 0.0166 | 0.728 | 6701 | tags=52%, list=11%, sig |
| GO IMMUNC | GO IMMUNOGLOBULIN | 148 | 0.77 | 1.8 | 0.006 | 0.0204 | 0.79 | 8081 | tags=69%, list=14%, sig |
| GO REGULA | GO REGULATION OF B C | 20 | 0.59 | 1.764 | 0.006 | 0.0273 | 0.856 | 2630 | tags=30%, list=5%, sign |
| GO REGULA | GO REGULATION OF AN | 20 | 0.76 | 1.885 | 0.006 | 0.0098 | 0.575 | 5785 | tags=65%, list=10%, sig |
| GO REGULA | GO REGULATION OF MY | 57 | 0.57 | 1.884 | 0.006 | 0.0099 | 0.58 | 3331 | tags=37%, list=6%, sign |
| GO ENDOSC | GO ENDOSOME LUMEN | 35 | 0.53 | 1.74 | 0.006 | 0.0329 | 0.893 | 8253 | tags=37%, list=14%, sig |
| GO CYSTEIN | GO CYSTEINE TYPE ENI | 59 | 0.49 | 1.699 | 0.006 | 0.0437 | 0.94 | 6860 | tags=37%, list=12%, sig |
| GO REGULA | GO REGULATION OF LEI | 120 | 0.54 | 1.946 | 0.006 | 0.0054 | 0.416 | 6386 | tags=39%, list=11%, sig |
| GO POSITIV | GO POSITIVE REGULATI | 20 | 0.68 | 1.849 | 0.006 | 0.014 | 0.681 | 1068 | tags=30%, list=2%, sign |
| GO ESCRT C | GO ESCRT COMPLEX | 27 | 0.58 | 1.808 | 0.006 | 0.0192 | 0.769 | 5636 | tags=44%, list=10%, sig |
| GO PHAGOC | GO PHAGOCYTIC CUP | 25 | 0.64 | 1.901 | 0.006 | 0.0085 | 0.532 | 5939 | tags=52%, list=10%, sig |
| GO REGULA | GO REGULATION OF LEI | 206 | 0.52 | 1.914 | 0.006 | 0.0074 | 0.499 | 6434 | tags=39%, list=11%, sig |
| GO T CELL I | GO T CELL MIGRATION | 65 | 0.63 | 1.922 | 0.006 | 0.0069 | 0.483 | 2846 | tags=35%, list=5%, sign |
| GO RESPON | GO RESPONSE TO UV C | 16 | 0.64 | 1.817 | 0.006 | 0.0178 | 0.746 | 2111 | tags=31%, list=4%, sign |
| GO RESPON | GO RESPONSE TO FUNG | 54 | 0.56 | 1.773 | 0.006 | 0.0255 | 0.84 | 7298 | tags=43%, list=12%, sig |
| GO REGULA | GO REGULATION OF MA | 42 | 0.62 | 1.956 | 0.006 | 0.0049 | 0.387 | 7814 | tags=52%, list=13%, sig |
| GO POSITIV | GO POSITIVE REGULATI | 419 | 0.5 | 1.921 | 0.006 | 0.0069 | 0.486 | 3623 | tags=28%, list=6%, sign |
| GO POSITIV | GO POSITIVE REGULATI | 34 | 0.6 | 1.874 | 0.006 | 0.011 | 0.614 | 2290 | tags=38%, list=4%, sign |
| GO CELLUL | GO CELLULAR RESPONS | 78 | 0.53 | 1.871 | 0.006 | 0.0112 | 0.619 | 6506 | tags=38%, list=11%, sig |
| GO MACROI | GO MACROPHAGE MIGI | 54 | 0.6 | 1.937 | 0.006 | 0.0059 | 0.441 | 8001 | tags=50%, list=14%, sig |
| GO EXTRINS | GO EXTRINSIC APOPTO | 87 | 0.48 | 1.831 | 0.006 | 0.016 | 0.721 | 3003 | tags=26%, list=5%, sign |
| GO COATED | GO COATED VESICLE M | 177 | 0.49 | 1.821 | 0.006 | 0.0172 | 0.74 | 4653 | tags=27%, list=8%, sign |
| GO POSITIV | GO POSITIVE REGULATI | 29 | 0.64 | 1.9 | 0.006 | 0.0086 | 0.538 | 3340 | tags=41%, list=6%, sign |
| GO INTERLI | GO INTERLEUKIN 7 MEI | 17 | 0.69 | 1.805 | 0.006 | 0.0197 | 0.777 | 2519 | tags=47%, list=4%, sign |
| GO PINOCY | GO PINOCYTOSIS | 22 | 0.58 | 1.788 | 0.006 | 0.0224 | 0.814 | 8857 | tags=59%, list=15%, sig |
| GO GLYCOS | GO GLYCOSYL COMPOU | 124 | 0.43 | 1.689 | 0.006 | 0.0462 | 0.952 | 8789 | tags=39%, list=15%, sig |
| GO DEOXYI | GO DEOXYRIBONUCLE( | 34 | 0.62 | 1.887 | 0.006 | 0.0096 | 0.569 | 7852 | tags=53%, list=13%, sig |
| GO REGULA | GO REGULATION OF EX | 61 | 0.52 | 1.793 | 0.006 | 0.0216 | 0.802 | 3003 | tags=28%, list=5%, sign |
| GO RESPON | GO RESPONSE TO INTEI | 55 | 0.49 | 1.642 | 0.006 | 0.0622 | 0.979 | 1386 | tags=20%, list=2%, sign |
| GO PROTEIN | GO PROTEIN MODIFICA | 288 | 0.5 | 1.83 | 0.006 | 0.0161 | 0.721 | 6166 | tags=29%, list=11%, sig |
| GO INTRINS | GO INTRINSIC APOPTOT | 59 | 0.5 | 1.815 | 0.006 | 0.0181 | 0.75 | 6471 | tags=34%, list=11%, sig |
| GO NUCLEC | GO NUCLEOSIDE TRIPH | 87 | 0.43 | 1.656 | 0.007 | 0.0577 | 0.973 | 8789 | tags=40%, list=15%, sig |
| GO T HELPE | GO T HELPER 2 CELL CY | 15 | 0.62 | 1.76 | 0.007 | 0.0282 | 0.863 | 2043 | tags=27%, list=3%, sign |
| GO REGULA | GO REGULATION OF MA | 55 | 0.59 | 1.92 | 0.008 | 0.0069 | 0.487 | 5104 | tags=36%, list=9%, sign |
| GO POSITIV | GO POSITIVE REGULATI | 41 | 0.59 | 1.858 | 0.008 | 0.0128 | 0.653 | 2361 | tags=32%, list=4%, sign |
| GO ORGAN ( | GO ORGAN OR TISSUE S | 32 | 0.56 | 1.665 | 0.008 | 0.0545 | 0.969 | 8589 | tags=50%, list=15%, sig |
| GO NITRIC ( | GO NITRIC OXIDE SYNT | 19 | 0.65 | 1.796 | 0.008 | 0.0211 | 0.796 | 2266 | tags=32%, list=4%, sign |
| GO REGULA | GO REGULATION OF MI | 19 | 0.7 | 1.807 | 0.008 | 0.0195 | 0.775 | 4352 | tags=47%, list=7%, sign |
| GO REGULA | GO REGULATION OF NE | 41 | 0.58 | 1.789 | 0.008 | 0.0222 | 0.812 | 5045 | tags=39%, list=9%, sign |
| GO GRANUI | GO GRANULOCYTE MIG | 143 | 0.57 | 1.969 | 0.008 | 0.0044 | 0.366 | 6386 | tags=41%, list=11%, sig |
| GO CHRONI | GO CHRONIC INFLAMM | 20 | 0.67 | 1.791 | 0.008 | 0.0218 | 0.808 | 9938 | tags=65%, list=17%, sig |
| GO MYELOI | GO MYELOID LEUKOCY | 208 | 0.47 | 1.831 | 0.008 | 0.016 | 0.721 | 6389 | tags=34%, list=11%, sig |
| GO POSITIV | GO POSITIVE REGULATI | 93 | 0.54 | 1.907 | 0.008 | 0.008 | 0.516 | 6386 | tags=42%, list=11%, sig |
| GO GRANUI | GO GRANULOCYTE CHE | 120 | 0.57 | 1.903 | 0.008 | 0.0083 | 0.527 | 6386 | tags=39%, list=11%, sig |
| GO NEUROI | GO NEUROINFLAMMAT | 75 | 0.58 | 1.9 | 0.008 | 0.0086 | 0.536 | 5045 | tags=41%, list=9%, sign |
| GO NEGATI | GO NEGATIVE REGULA | 188 | 0.42 | 1.714 | 0.008 | 0.0397 | 0.929 | 5619 | tags=27%, list=10%, sig |
| GO NEGATI | GO NEGATIVE REGULA | 25 | 0.56 | 1.692 | 0.008 | 0.0454 | 0.951 | 5416 | tags=44%, list=9%, sign |
| GO NEGATI | GO NEGATIVE REGULA | 32 | 0.63 | 1.884 | 0.008 | 0.0099 | 0.58 | 4359 | tags=38%, list=7%, sign |
| GO MYELOI | GO MYELOID LEUKOCY | 211 | 0.53 | 1.904 | 0.008 | 0.0083 | 0.521 | 6161 | tags=37%, list=11%, sig |
| GO CLATHR | GO CLATHRIN COATED | 115 | 0.48 | 1.786 | 0.008 | 0.0227 | 0.818 | 3159 | tags=24%, list=5%, sign |
| GO VIRAL B | GO VIRAL BUDDING | 26 | 0.58 | 1.751 | 0.008 | 0.0301 | 0.875 | 5636 | tags=42%, list=10%, sig |
| GO OXIDOR | GO OXIDOREDUCTASE | 108 | 0.5 | 1.816 | 0.008 | 0.018 | 0.747 | 8955 | tags=48%, list=15%, sig |
| GO NEGATI | GO NEGATIVE REGULA | 102 | 0.61 | 1.938 | 0.008 | 0.0059 | 0.437 | 6103 | tags=33%, list=10%, sig |
| GO INTERLI | GO INTERLEUKIN 1 REC | 16 | 0.69 | 1.706 | 0.008 | 0.0421 | 0.932 | 6836 | tags=56%, list=12%, sig |
| GO PROTEIN | GO PROTEIN POLYUBIQ | 332 | 0.46 | 1.805 | 0.008 | 0.0196 | 0.778 | 6850 | tags=30%, list=12%, sig |

| | | | | | | | | | |
|---|---|---|---|---|---|---|---|---|---|
| GO THYMIC | GO THYMIC T CELL SEL | 22 | 0.7 | 1.89 | 0.008 | 0.0094 | 0.565 | 3953 | tags=55%, list=7%, sign |
| GO MHC CL | GO MHC CLASS II BIOSY | 16 | 0.73 | 1.836 | 0.008 | 0.0155 | 0.71 | 3436 | tags=44%, list=6%, sign |
| GO POSITIV | GO POSITIVE REGULATI | 22 | 0.63 | 1.77 | 0.008 | 0.0262 | 0.846 | 4938 | tags=50%, list=8%, sign |
| GO RIG I SIG | GO RIG I SIGNALING PA | 23 | 0.57 | 1.69 | 0.008 | 0.0459 | 0.952 | 1144 | tags=22%, list=2%, sign |
| GO ACUTE I | GO ACUTE INFLAMMAT | 110 | 0.5 | 1.8 | 0.008 | 0.0204 | 0.79 | 8635 | tags=41%, list=15%, sig |
| GO BLOC C( | GO BLOC COMPLEX | 19 | 0.63 | 1.827 | 0.009 | 0.0165 | 0.728 | 8714 | tags=63%, list=15%, sig |
| GO REGULA | GO REGULATION OF MY | 232 | 0.42 | 1.685 | 0.009 | 0.0471 | 0.955 | 6389 | tags=29%, list=11%, sig |
| GO MYELOI | GO MYELOID CELL DIFF | 396 | 0.41 | 1.701 | 0.009 | 0.0432 | 0.939 | 6389 | tags=28%, list=11%, sig |
| GO NEGATI | GO NEGATIVE REGULAT | 16 | 0.61 | 1.7 | 0.009 | 0.0435 | 0.94 | 1338 | tags=25%, list=2%, sign |
| GO NATURA | GO NATURAL KILLER C | 22 | 0.58 | 1.67 | 0.009 | 0.0524 | 0.968 | 2396 | tags=27%, list=4%, sign |
| GO HYDRO( | GO HYDROGEN PEROXI | 16 | 0.62 | 1.766 | 0.009 | 0.0269 | 0.851 | 4738 | tags=50%, list=8%, sign |
| GO REGULA | GO REGULATION OF AP | 401 | 0.38 | 1.631 | 0.009 | 0.0671 | 0.984 | 7681 | tags=31%, list=13%, sig |
| GO POSITIV | GO POSITIVE REGULATI | 393 | 0.38 | 1.627 | 0.009 | 0.0688 | 0.985 | 5494 | tags=25%, list=9%, sign |
| GO NEGATI | GO NEGATIVE REGULAT | 16 | 0.71 | 1.819 | 0.01 | 0.0175 | 0.744 | 2748 | tags=44%, list=5%, sign |
| GO TRANSP | GO TRANSPORT VESICL | 200 | 0.44 | 1.684 | 0.01 | 0.0476 | 0.956 | 5698 | tags=26%, list=10%, sig |
| GO REGULA | GO REGULATION OF INI | 32 | 0.69 | 1.875 | 0.01 | 0.0109 | 0.611 | 7314 | tags=50%, list=13%, sig |
| GO IRON IO | GO IRON ION TRANSME | 18 | 0.59 | 1.706 | 0.01 | 0.0421 | 0.932 | 6306 | tags=44%, list=11%, sig |
| GO FAT SOL | GO FAT SOLUBLE VITAI | 15 | 0.66 | 1.792 | 0.01 | 0.0217 | 0.806 | 5219 | tags=47%, list=9%, sign |
| GO MULTIV | GO MULTIVESICULAR B | 31 | 0.58 | 1.823 | 0.01 | 0.017 | 0.735 | 3932 | tags=39%, list=7%, sign |
| GO REGULA | GO REGULATION OF VII | 19 | 0.58 | 1.725 | 0.01 | 0.0368 | 0.916 | 2002 | tags=26%, list=3%, sign |
| GO REGULA | GO REGULATION OF B C | 62 | 0.58 | 1.825 | 0.01 | 0.0167 | 0.732 | 2802 | tags=32%, list=5%, sign |
| GO POSTRE | GO POSTREPLICATION I | 49 | 0.62 | 1.848 | 0.01 | 0.0141 | 0.681 | 9018 | tags=51%, list=15%, sig |
| GO NEGATI | GO NEGATIVE REGULAT | 15 | 0.67 | 1.746 | 0.01 | 0.0314 | 0.885 | 1735 | tags=33%, list=3%, sign |
| GO REGULA | GO REGULATION OF OS | 66 | 0.49 | 1.71 | 0.01 | 0.0408 | 0.93 | 6716 | tags=39%, list=12%, sig |
| GO REGULA | GO REGULATION OF SU | 37 | 0.52 | 1.737 | 0.01 | 0.0337 | 0.896 | 6444 | tags=38%, list=11%, sig |
| GO MEMBR | GO MEMBRANE RAFT O | 25 | 0.6 | 1.836 | 0.01 | 0.0155 | 0.712 | 8159 | tags=68%, list=14%, sig |
| GO POSITIV | GO POSITIVE REGULATI | 25 | 0.67 | 1.845 | 0.01 | 0.0144 | 0.69 | 8001 | tags=60%, list=14%, sig |
| GO CD8 POS | GO CD8 POSITIVE ALPH | 15 | 0.69 | 1.73 | 0.01 | 0.0352 | 0.91 | 6975 | tags=47%, list=12%, sig |
| GO NUCLEO | GO NUCLEOBASE CONT | 102 | 0.42 | 1.659 | 0.01 | 0.0563 | 0.973 | 8966 | tags=38%, list=15%, sig |
| GO POSITIV | GO POSITIVE REGULATI | 26 | 0.67 | 1.814 | 0.01 | 0.0183 | 0.752 | 4124 | tags=42%, list=7%, sign |
| GO POSITIV | GO POSITIVE REGULATI | 31 | 0.54 | 1.667 | 0.011 | 0.0534 | 0.969 | 5619 | tags=32%, list=10%, sig |
| GO DNA DA | GO DNA DAMAGE RESP | 18 | 0.55 | 1.622 | 0.011 | 0.0706 | 0.987 | 4763 | tags=28%, list=8%, sign |
| GO REGULA | GO REGULATION OF LY | 62 | 0.57 | 1.848 | 0.011 | 0.0141 | 0.681 | 8773 | tags=48%, list=15%, sig |
| GO PHOSPH | GO PHOSPHATIDYLINOS | 29 | 0.56 | 1.77 | 0.011 | 0.0262 | 0.847 | 8059 | tags=52%, list=14%, sig |
| GO ENDOPL | GO ENDOPLASMIC RETI | 23 | 0.56 | 1.758 | 0.011 | 0.0286 | 0.865 | 7829 | tags=39%, list=13%, sig |
| GO APOPTO | GO APOPTOTIC MITOCH | 120 | 0.44 | 1.713 | 0.011 | 0.04 | 0.929 | 7601 | tags=37%, list=13%, sig |
| GO HYDROI | GO HYDROLASE ACTIVI | 119 | 0.4 | 1.627 | 0.011 | 0.0688 | 0.985 | 6396 | tags=28%, list=11%, sig |
| GO HEMAT( | GO HEMATOPOIETIC PR | 170 | 0.43 | 1.679 | 0.011 | 0.0494 | 0.961 | 7843 | tags=32%, list=13%, sig |
| GO DETECT | GO DETECTION OF OTH | 19 | 0.73 | 1.806 | 0.011 | 0.0195 | 0.775 | 2198 | tags=37%, list=4%, sign |
| GO RIBONU | GO RIBONUCLEOSIDE M | 69 | 0.44 | 1.682 | 0.011 | 0.0479 | 0.957 | 8764 | tags=41%, list=15%, sig |
| GO NEGATI | GO NEGATIVE REGULAT | 356 | 0.35 | 1.48 | 0.011 | 0.1439 | 0.999 | 7829 | tags=27%, list=13%, sig |
| GO PIGMEN | GO PIGMENT GRANULE | 26 | 0.55 | 1.731 | 0.011 | 0.0352 | 0.909 | 7458 | tags=35%, list=13%, sig |
| GO ESTABLI | GO ESTABLISHMENT OI | 24 | 0.55 | 1.686 | 0.011 | 0.047 | 0.955 | 6036 | tags=29%, list=10%, sig |
| GO LAMELL | GO LAMELLAR BODY | 17 | 0.66 | 1.648 | 0.012 | 0.0603 | 0.977 | 4659 | tags=29%, list=8%, sign |
| GO MONOC | GO MONOCYTE CHEMO | 64 | 0.59 | 1.799 | 0.012 | 0.0206 | 0.79 | 5097 | tags=41%, list=9%, sign |
| GO IMMUN( | GO IMMUNOGLOBULIN | 62 | 0.79 | 1.733 | 0.012 | 0.0347 | 0.903 | 7872 | tags=74%, list=13%, sig |
| GO OXIDOR | GO OXIDOREDUCTASE | 55 | 0.51 | 1.792 | 0.012 | 0.0216 | 0.804 | 6732 | tags=38%, list=12%, sig |
| GO INTRINS | GO INTRINSIC APOPTOT | 76 | 0.48 | 1.756 | 0.012 | 0.0289 | 0.868 | 7681 | tags=41%, list=13%, sig |
| GO VIRAL B | GO VIRAL BUDDING VIA | 21 | 0.6 | 1.704 | 0.012 | 0.0425 | 0.936 | 5636 | tags=48%, list=10%, sig |
| GO MONON | GO MONONUCLEAR CEI | 38 | 0.52 | 1.73 | 0.012 | 0.0353 | 0.91 | 2629 | tags=26%, list=5%, sign |
| GO LIPOPOI | GO LIPOPOLYSACCHAR | 31 | 0.61 | 1.788 | 0.012 | 0.0224 | 0.813 | 4094 | tags=42%, list=7%, sign |
| GO PYRIDIN | GO PYRIDINE CONTAIN | 33 | 0.47 | 1.565 | 0.012 | 0.0969 | 0.996 | 8909 | tags=39%, list=15%, sig |
| GO REGULA | GO REGULATION OF B C | 29 | 0.61 | 1.763 | 0.012 | 0.0275 | 0.86 | 7576 | tags=48%, list=13%, sig |
| GO CYTOLY | GO CYTOLYSIS | 29 | 0.62 | 1.765 | 0.012 | 0.0272 | 0.855 | 6482 | tags=45%, list=11%, sig |
| GO HYDRO( | GO HYDROGEN PEROXI | 56 | 0.41 | 1.508 | 0.013 | 0.1269 | 0.999 | 4738 | tags=25%, list=8%, sign |
| GO REGULA | GO REGULATION OF MA | 29 | 0.58 | 1.805 | 0.013 | 0.0197 | 0.778 | 10492 | tags=55%, list=18%, sig |
| GO PURINE | GO PURINE NUCLEOSID | 65 | 0.43 | 1.642 | 0.013 | 0.0624 | 0.981 | 8753 | tags=43%, list=15%, sig |
| GO GRANUI | GO GRANULOCYTE MA( | 16 | 0.64 | 1.72 | 0.013 | 0.0379 | 0.922 | 5312 | tags=50%, list=9%, sign |

| | | | | | | | | | |
|---|---|---|---|---|---|---|---|---|---|
| GO INTERLE | GO INTERLEUKIN 6 MEI | 34 | 0.54 | 1.675 | 0.013 | 0.0504 | 0.965 | 1386 | tags=21%, list=2%, sign |
| GO COPPER | GO COPPER ION HOMEC | 17 | 0.59 | 1.644 | 0.013 | 0.0614 | 0.979 | 2308 | tags=35%, list=4%, sign |
| GO RESPON | GO RESPONSE TO INTEF | 28 | 0.56 | 1.703 | 0.013 | 0.0427 | 0.936 | 2988 | tags=36%, list=5%, sign |
| GO REGULA | GO REGULATION OF NU | 22 | 0.6 | 1.722 | 0.013 | 0.0375 | 0.918 | 4960 | tags=36%, list=8%, sign |
| GO RESPON | GO RESPONSE TO CADM | 64 | 0.45 | 1.61 | 0.013 | 0.0761 | 0.989 | 5332 | tags=25%, list=9%, sign |
| GO MYELOI | GO MYELOID CELL APO | 32 | 0.55 | 1.73 | 0.013 | 0.0353 | 0.911 | 4795 | tags=31%, list=8%, sign |
| GO NEGATI | GO NEGATIVE REGULAT | 22 | 0.57 | 1.698 | 0.013 | 0.044 | 0.941 | 2481 | tags=27%, list=4%, sign |
| GO IMMUNO | GO IMMUNOGLOBULIN | 66 | 0.77 | 1.733 | 0.014 | 0.0348 | 0.903 | 7872 | tags=71%, list=13%, sig |
| GO SERINE I | GO SERINE HYDROLASI | 186 | 0.36 | 1.473 | 0.014 | 0.1478 | 0.999 | 8238 | tags=30%, list=14%, sig |
| GO CELL CH | GO CELL CHEMOTAXIS | 298 | 0.47 | 1.804 | 0.014 | 0.0198 | 0.779 | 6386 | tags=33%, list=11%, sig |
| GO POSITIV | GO POSITIVE REGULATI | 26 | 0.61 | 1.768 | 0.014 | 0.0266 | 0.85 | 4352 | tags=31%, list=7%, sign |
| GO ENDOPE | GO ENDOPEPTIDASE RE | 187 | 0.4 | 1.54 | 0.014 | 0.1086 | 0.999 | 7209 | tags=28%, list=12%, sig |
| GO POSITIV | GO POSITIVE REGULATI | 25 | 0.65 | 1.808 | 0.014 | 0.0193 | 0.77 | 10492 | tags=76%, list=18%, sig |
| GO B CELL A | GO B CELL ACTIVATION | 74 | 0.54 | 1.75 | 0.014 | 0.0302 | 0.876 | 5416 | tags=34%, list=9%, sign |
| GO LATE EN | GO LATE ENDOSOME TC | 24 | 0.59 | 1.765 | 0.014 | 0.0272 | 0.855 | 5636 | tags=38%, list=10%, sig |
| GO POSITIV | GO POSITIVE REGULATI | 30 | 0.62 | 1.827 | 0.014 | 0.0163 | 0.723 | 2542 | tags=33%, list=4%, sign |
| GO REGULA | GO REGULATION OF AM | 40 | 0.46 | 1.628 | 0.014 | 0.0685 | 0.985 | 6375 | tags=38%, list=11%, sig |
| GO REGULA | GO REGULATION OF PEI | 259 | 0.41 | 1.66 | 0.014 | 0.0561 | 0.972 | 7314 | tags=31%, list=13%, sig |
| GO MAINTE | GO MAINTENANCE OF F | 23 | 0.56 | 1.726 | 0.015 | 0.0365 | 0.916 | 7037 | tags=39%, list=12%, sig |
| GO REGULA | GO REGULATION OF EX | 160 | 0.44 | 1.693 | 0.015 | 0.0451 | 0.949 | 3039 | tags=22%, list=5%, sign |
| GO PEPTIDY | GO PEPTIDYL TYROSINI | 41 | 0.5 | 1.651 | 0.015 | 0.0594 | 0.977 | 7843 | tags=41%, list=13%, sig |
| GO POSITIV | GO POSITIVE REGULATI | 58 | 0.41 | 1.588 | 0.015 | 0.0855 | 0.994 | 6828 | tags=36%, list=12%, sig |
| GO MODULA | GO MODULATION BY HO | 29 | 0.57 | 1.693 | 0.015 | 0.0451 | 0.949 | 5615 | tags=34%, list=10%, sig |
| GO INTRINS | GO INTRINSIC APOPTOT | 42 | 0.52 | 1.662 | 0.016 | 0.0555 | 0.972 | 9734 | tags=45%, list=17%, sig |
| GO RESPON | GO RESPONSE TO CHEM | 94 | 0.57 | 1.8 | 0.016 | 0.0203 | 0.79 | 4110 | tags=34%, list=7%, sign |
| GO MEMBRA | GO MEMBRANE INVAGI | 129 | 0.59 | 1.747 | 0.016 | 0.031 | 0.883 | 8121 | tags=57%, list=14%, sig |
| GO CYSTEIN | GO CYSTEINE TYPE ENI | 25 | 0.52 | 1.593 | 0.016 | 0.083 | 0.993 | 7800 | tags=40%, list=13%, sig |
| GO NEGATI | GO NEGATIVE REGULAT | 18 | 0.63 | 1.716 | 0.016 | 0.039 | 0.927 | 2846 | tags=39%, list=5%, sign |
| GO POSITIV | GO POSITIVE REGULATI | 100 | 0.44 | 1.651 | 0.016 | 0.0595 | 0.977 | 5416 | tags=28%, list=9%, sign |
| GO POSITIV | GO POSITIVE REGULATI | 16 | 0.66 | 1.727 | 0.016 | 0.0361 | 0.915 | 1644 | tags=31%, list=3%, sign |
| GO B CELL I | GO B CELL PROLIFERAT | 97 | 0.54 | 1.783 | 0.016 | 0.0235 | 0.822 | 5364 | tags=34%, list=9%, sign |
| GO TUMOR | GO TUMOR NECROSIS F | 41 | 0.59 | 1.814 | 0.016 | 0.0183 | 0.751 | 8001 | tags=46%, list=14%, sig |
| GO UBIQUIT | GO UBIQUITIN BINDING | 72 | 0.48 | 1.704 | 0.016 | 0.0424 | 0.936 | 2742 | tags=24%, list=5%, sign |
| GO LEUKOC | GO LEUKOCYTE HOMEC | 86 | 0.55 | 1.842 | 0.016 | 0.0149 | 0.7 | 8001 | tags=45%, list=14%, sig |
| GO MITOCH | GO MITOCHONDRIAL CY | 21 | 0.65 | 1.721 | 0.017 | 0.0375 | 0.919 | 10686 | tags=62%, list=18%, sig |
| GO NEGATI | GO NEGATIVE REGULAT | 264 | 0.37 | 1.516 | 0.017 | 0.1225 | 0.999 | 7829 | tags=28%, list=13%, sig |
| GO DETECT | GO DETECTION OF BIOT | 36 | 0.62 | 1.779 | 0.017 | 0.0244 | 0.827 | 5546 | tags=44%, list=9%, sign |
| GO VACUOI | GO VACUOLAR MEMBR | 403 | 0.43 | 1.769 | 0.017 | 0.0263 | 0.849 | 7659 | tags=34%, list=13%, sig |
| GO MEMBRA | GO MEMBRANE FUSION | 156 | 0.41 | 1.619 | 0.017 | 0.0722 | 0.988 | 6323 | tags=29%, list=11%, sig |
| GO AMYLOI | GO AMYLOID FIBRIL FC | 18 | 0.58 | 1.683 | 0.017 | 0.0476 | 0.956 | 6008 | tags=33%, list=10%, sig |
| GO SNAP RE | GO SNAP RECEPTOR AC | 36 | 0.51 | 1.692 | 0.017 | 0.0452 | 0.949 | 6323 | tags=42%, list=11%, sig |
| GO APOPTO | GO APOPTOTIC PROCES | 25 | 0.54 | 1.687 | 0.018 | 0.0467 | 0.954 | 3131 | tags=32%, list=5%, sign |
| GO GOLGI A | GO GOLGI ASSOCIATED | 175 | 0.46 | 1.721 | 0.018 | 0.0375 | 0.918 | 4459 | tags=24%, list=8%, sign |
| GO TERTIAF | GO TERTIARY GRANULI | 71 | 0.55 | 1.811 | 0.018 | 0.0188 | 0.763 | 4639 | tags=41%, list=8%, sign |
| GO POSITIV | GO POSITIVE REGULATI | 193 | 0.42 | 1.662 | 0.018 | 0.0554 | 0.972 | 6471 | tags=28%, list=11%, sig |
| GO POSITIV | GO POSITIVE REGULATI | 28 | 0.53 | 1.696 | 0.018 | 0.0446 | 0.946 | 6161 | tags=36%, list=11%, sig |
| GO OSTEOC | GO OSTEOCLAST DIFFEI | 96 | 0.46 | 1.627 | 0.018 | 0.0687 | 0.985 | 7877 | tags=42%, list=13%, sig |
| GO POSITIV | GO POSITIVE REGULATI | 366 | 0.34 | 1.487 | 0.018 | 0.1403 | 0.999 | 4124 | tags=20%, list=7%, sign |
| GO DNA SYI | GO DNA SYNTHESIS INV | 49 | 0.59 | 1.76 | 0.019 | 0.0283 | 0.864 | 9018 | tags=47%, list=15%, sig |
| GO VITAMII | GO VITAMIN BIOSYNTH | 21 | 0.55 | 1.635 | 0.019 | 0.0656 | 0.982 | 5219 | tags=33%, list=9%, sign |
| GO ACTIVA | GO ACTIVATION OF MA | 151 | 0.38 | 1.54 | 0.019 | 0.1087 | 0.999 | 8568 | tags=36%, list=15%, sig |
| GO HOMEOS | GO HOMEOSTASIS OF N | 251 | 0.4 | 1.645 | 0.019 | 0.0613 | 0.979 | 6691 | tags=28%, list=11%, sig |
| GO NEGATI | GO NEGATIVE REGULAT | 33 | 0.51 | 1.609 | 0.019 | 0.0765 | 0.989 | 4694 | tags=27%, list=8%, sign |
| GO NUCLEC | GO NUCLEOSIDE PHOSF | 71 | 0.4 | 1.546 | 0.019 | 0.105 | 0.998 | 10440 | tags=45%, list=18%, sig |
| GO PROTEII | GO PROTEIN MATURATI | 277 | 0.33 | 1.448 | 0.019 | 0.162 | 0.999 | 8256 | tags=27%, list=14%, sig |
| GO POSITIV | GO POSITIVE REGULATI | 25 | 0.6 | 1.723 | 0.02 | 0.0374 | 0.918 | 8559 | tags=56%, list=15%, sig |
| GO IRON IOI | GO IRON ION TRANSPOI | 77 | 0.43 | 1.643 | 0.02 | 0.0621 | 0.979 | 9251 | tags=40%, list=16%, sig |
| GO LYMPH I | GO LYMPH NODE DEVE | 17 | 0.65 | 1.777 | 0.02 | 0.0248 | 0.832 | 7717 | tags=53%, list=13%, sig |

| | | | | | | | | | |
|---|---|---|---|---|---|---|---|---|---|
| GO POSITIV | GO POSITIVE REGULATI | 51 | 0.5 | 1.684 | 0.02 | 0.0476 | 0.956 | 7972 | tags=45%, list=14%, sig |
| GO POSITIV | GO POSITIVE REGULATI | 57 | 0.45 | 1.618 | 0.02 | 0.0721 | 0.988 | 4938 | tags=33%, list=8%, sign |
| GO LATE EN | GO LATE ENDOSOME TO | 17 | 0.59 | 1.677 | 0.02 | 0.05 | 0.963 | 3870 | tags=35%, list=7%, sign |
| GO REGULA | GO REGULATION OF TR | 22 | 0.55 | 1.613 | 0.02 | 0.0746 | 0.988 | 1169 | tags=23%, list=2%, sign |
| GO SIGNAL | GO SIGNAL TRANSDUCT | 74 | 0.44 | 1.598 | 0.02 | 0.081 | 0.992 | 7662 | tags=34%, list=13%, sig |
| GO POSITIV | GO POSITIVE REGULATI | 85 | 0.4 | 1.554 | 0.021 | 0.1014 | 0.996 | 8753 | tags=39%, list=15%, sig |
| GO REGULA | GO REGULATION OF T C | 42 | 0.56 | 1.762 | 0.021 | 0.0276 | 0.86 | 2846 | tags=31%, list=5%, sign |
| GO B CELL I | GO B CELL RECEPTOR S | 121 | 0.68 | 1.776 | 0.021 | 0.0249 | 0.833 | 7872 | tags=56%, list=13%, sig |
| GO NEGATI | GO NEGATIVE REGULAT | 81 | 0.41 | 1.578 | 0.021 | 0.0903 | 0.996 | 6716 | tags=32%, list=12%, sig |
| GO PURINE | GO PURINE CONTAININ | 49 | 0.45 | 1.604 | 0.021 | 0.0783 | 0.992 | 2599 | tags=24%, list=4%, sign |
| GO PYRIMII | GO PYRIMIDINE CONTA | 81 | 0.43 | 1.612 | 0.021 | 0.0749 | 0.989 | 9155 | tags=41%, list=16%, sig |
| GO NEGATI | GO NEGATIVE REGULAT | 60 | 0.42 | 1.553 | 0.021 | 0.1016 | 0.996 | 7614 | tags=32%, list=13%, sig |
| GO HEXOSE | GO HEXOSE CATABOLIC | 54 | 0.49 | 1.657 | 0.021 | 0.0574 | 0.973 | 4282 | tags=30%, list=7%, sign |
| GO ENDOLY | GO ENDOLYSOSOME | 20 | 0.62 | 1.714 | 0.021 | 0.0394 | 0.928 | 4279 | tags=40%, list=7%, sign |
| GO ORGANE | GO ORGANELLE MEMBI | 100 | 0.45 | 1.645 | 0.021 | 0.0614 | 0.979 | 6267 | tags=33%, list=11%, sig |
| GO FIBROBI | GO FIBROBLAST PROLII | 83 | 0.41 | 1.562 | 0.021 | 0.0984 | 0.996 | 6729 | tags=34%, list=12%, sig |
| GO B CELL I | GO B CELL HOMEOSTAS | 29 | 0.63 | 1.749 | 0.022 | 0.0306 | 0.879 | 2630 | tags=28%, list=5%, sign |
| GO REGULA | GO REGULATION OF STI | 117 | 0.49 | 1.704 | 0.022 | 0.0425 | 0.936 | 4080 | tags=26%, list=7%, sign |
| GO CARD D | GO CARD DOMAIN BIND | 15 | 0.65 | 1.739 | 0.022 | 0.0332 | 0.894 | 1465 | tags=33%, list=3%, sign |
| GO RESPON | GO RESPONSE TO MURA | 18 | 0.61 | 1.695 | 0.023 | 0.0447 | 0.946 | 11765 | tags=67%, list=20%, sig |
| GO INTESTI | GO INTESTINAL EPITHE | 15 | 0.62 | 1.643 | 0.023 | 0.0622 | 0.979 | 8888 | tags=47%, list=15%, sig |
| GO CELL CE | GO CELL CELL ADHESIC | 16 | 0.69 | 1.701 | 0.023 | 0.0432 | 0.939 | 3663 | tags=38%, list=6%, sign |
| GO PROTEA | GO PROTEASOME ACCE | 24 | 0.72 | 1.759 | 0.023 | 0.0282 | 0.864 | 2348 | tags=33%, list=4%, sign |
| GO CELLUL | GO CELLULAR MODIFIE | 184 | 0.34 | 1.432 | 0.023 | 0.1725 | 0.999 | 8059 | tags=31%, list=14%, sig |
| GO POSITIV | GO POSITIVE REGULATI | 19 | 0.65 | 1.711 | 0.024 | 0.0404 | 0.929 | 9845 | tags=68%, list=17%, sig |
| GO NEGATI | GO NEGATIVE REGULAT | 16 | 0.59 | 1.69 | 0.024 | 0.046 | 0.952 | 5785 | tags=31%, list=10%, sig |
| GO REGULA | GO REGULATION OF INT | 159 | 0.37 | 1.528 | 0.024 | 0.1155 | 0.999 | 7829 | tags=31%, list=13%, sig |
| GO PHOSPH | GO PHOSPHATIDYLINOS | 76 | 0.38 | 1.467 | 0.024 | 0.1511 | 0.999 | 4380 | tags=25%, list=8%, sign |
| GO DISULFI | GO DISULFIDE OXIDORI | 38 | 0.52 | 1.687 | 0.024 | 0.0469 | 0.955 | 6732 | tags=39%, list=12%, sig |
| GO AMYLOI | GO AMYLOID PRECURS | 50 | 0.42 | 1.566 | 0.024 | 0.0965 | 0.996 | 6375 | tags=32%, list=11%, sig |
| GO CELLUL | GO CELLULAR PIGMENT | 52 | 0.46 | 1.664 | 0.024 | 0.0548 | 0.969 | 9027 | tags=40%, list=15%, sig |
| GO POSITIV | GO POSITIVE REGULATI | 90 | 0.4 | 1.539 | 0.024 | 0.1087 | 0.999 | 2824 | tags=21%, list=5%, sign |
| GO MAP KIN | GO MAP KINASE KINASI | 22 | 0.57 | 1.693 | 0.025 | 0.0451 | 0.949 | 5542 | tags=36%, list=9%, sign |
| GO SOMATI | GO SOMATIC DIVERSIFI | 60 | 0.5 | 1.649 | 0.025 | 0.0603 | 0.977 | 4441 | tags=30%, list=8%, sign |
| GO ACUTE I | GO ACUTE PHASE RESP | 47 | 0.55 | 1.638 | 0.025 | 0.0641 | 0.982 | 9190 | tags=49%, list=16%, sig |
| GO POSITIV | GO POSITIVE REGULATI | 37 | 0.49 | 1.648 | 0.025 | 0.0605 | 0.977 | 2630 | tags=24%, list=5%, sign |
| GO INTERA | GO INTERACTION WITH | 92 | 0.43 | 1.572 | 0.025 | 0.0937 | 0.996 | 7298 | tags=32%, list=12%, sig |
| GO VACUOI | GO VACUOLAR ACIDIFI | 23 | 0.55 | 1.634 | 0.025 | 0.0659 | 0.983 | 8159 | tags=52%, list=14%, sig |
| GO INNATE | GO INNATE IMMUNE RE | 17 | 0.63 | 1.548 | 0.026 | 0.1043 | 0.998 | 8589 | tags=53%, list=15%, sig |
| GO PROTEIN | GO PROTEIN INSERTION | 61 | 0.45 | 1.578 | 0.026 | 0.0904 | 0.996 | 7432 | tags=36%, list=13%, sig |
| GO REGULA | GO REGULATION OF PEI | 23 | 0.57 | 1.591 | 0.026 | 0.084 | 0.994 | 10532 | tags=48%, list=18%, sig |
| GO REGULA | GO REGULATION OF MI | 44 | 0.48 | 1.578 | 0.026 | 0.0902 | 0.996 | 9259 | tags=52%, list=16%, sig |
| GO ACTIVA | GO ACTIVATION OF JUN | 37 | 0.47 | 1.56 | 0.026 | 0.099 | 0.996 | 6471 | tags=41%, list=11%, sig |
| GO CYTOPL | GO CYTOPLASMIC SIDE | 15 | 0.56 | 1.574 | 0.026 | 0.0924 | 0.996 | 7383 | tags=60%, list=13%, sig |
| GO MULTIV | GO MULTIVESICULAR E | 39 | 0.5 | 1.701 | 0.026 | 0.0433 | 0.939 | 5636 | tags=41%, list=10%, sig |
| GO REGULA | GO REGULATION OF MA | 42 | 0.53 | 1.692 | 0.027 | 0.0455 | 0.951 | 3075 | tags=36%, list=5%, sign |
| GO REGULA | GO REGULATION OF RE | 27 | 0.51 | 1.591 | 0.027 | 0.0841 | 0.994 | 5785 | tags=33%, list=10%, sig |
| GO MITOTIC | GO MITOTIC CYTOKINE | 23 | 0.57 | 1.661 | 0.027 | 0.0559 | 0.972 | 7430 | tags=39%, list=13%, sig |
| GO POSITIV | GO POSITIVE REGULATI | 21 | 0.59 | 1.688 | 0.028 | 0.0467 | 0.954 | 3483 | tags=38%, list=6%, sign |
| GO CELLUL | GO CELLULAR OXIDANT | 96 | 0.39 | 1.518 | 0.028 | 0.1208 | 0.999 | 5400 | tags=26%, list=9%, sign |
| GO NUCLEC | GO NUCLEOTIDE EXCIS | 29 | 0.57 | 1.691 | 0.028 | 0.0455 | 0.951 | 8544 | tags=45%, list=15%, sig |
| GO PHOSPH | GO PHOSPHATIDYLINOS | 19 | 0.59 | 1.677 | 0.028 | 0.0498 | 0.962 | 3076 | tags=37%, list=5%, sign |
| GO NEGATI | GO NEGATIVE REGULAT | 204 | 0.33 | 1.404 | 0.028 | 0.1931 | 0.999 | 7003 | tags=25%, list=12%, sig |
| GO REGULA | GO REGULATION OF MY | 52 | 0.47 | 1.607 | 0.028 | 0.0772 | 0.991 | 5416 | tags=37%, list=9%, sign |
| GO PURINEI | GO PURINERGIC NUCLE | 32 | 0.58 | 1.698 | 0.029 | 0.0439 | 0.941 | 6701 | tags=44%, list=11%, sig |
| GO BENZEN | GO BENZENE CONTAINI | 23 | 0.51 | 1.539 | 0.029 | 0.1088 | 0.999 | 5608 | tags=35%, list=10%, sig |
| GO SECOND | GO SECONDARY LYSOS | 16 | 0.63 | 1.607 | 0.029 | 0.0771 | 0.991 | 8159 | tags=56%, list=14%, sig |
| GO PROTEIN | GO PROTEIN K63 LINKE | 56 | 0.46 | 1.635 | 0.029 | 0.0655 | 0.982 | 8055 | tags=39%, list=14%, sig |

| | | | | | | | | | |
|---|---|---|---|---|---|---|---|---|---|
| GO POSITIV | GO POSITIVE REGULATI | 23 | 0.56 | 1.588 | 0.029 | 0.0856 | 0.994 | 8593 | tags=43%, list=15%, sig |
| GO SOMATI | GO SOMATIC RECOMBII | 50 | 0.52 | 1.67 | 0.03 | 0.0524 | 0.968 | 3991 | tags=30%, list=7%, sign |
| GO PYRIMII | GO PYRIMIDINE CONTA | 38 | 0.51 | 1.684 | 0.03 | 0.0474 | 0.955 | 7852 | tags=37%, list=13%, sig |
| GO TOLL LII | GO TOLL LIKE RECEPTC | 39 | 0.53 | 1.645 | 0.03 | 0.0614 | 0.979 | 5939 | tags=49%, list=10%, sig |
| GO POSITIV | GO POSITIVE REGULATI | 27 | 0.54 | 1.562 | 0.03 | 0.0982 | 0.996 | 6389 | tags=41%, list=11%, sig |
| GO CYTOPL | GO CYTOPLASMIC SIDE | 184 | 0.37 | 1.517 | 0.03 | 0.1213 | 0.999 | 7643 | tags=34%, list=13%, sig |
| GO MEMBR/ | GO MEMBRANE PROTEI | 60 | 0.47 | 1.645 | 0.03 | 0.0614 | 0.979 | 6075 | tags=35%, list=10%, sig |
| GO MODUL/ | GO MODULATION OF PF | 112 | 0.43 | 1.564 | 0.03 | 0.0974 | 0.996 | 7298 | tags=34%, list=12%, sig |
| GO NEGATI | GO NEGATIVE REGULAT | 39 | 0.47 | 1.536 | 0.03 | 0.111 | 0.999 | 5216 | tags=26%, list=9%, sign |
| GO REGULA | GO REGULATION OF ME | 85 | 0.41 | 1.531 | 0.03 | 0.113 | 0.999 | 9399 | tags=40%, list=16%, sig |
| GO MONONI | GO MONONUCLEAR CEI | 91 | 0.54 | 1.736 | 0.03 | 0.0341 | 0.899 | 5097 | tags=38%, list=9%, sign |
| GO NEGATI | GO NEGATIVE REGULAT | 119 | 0.35 | 1.417 | 0.03 | 0.1837 | 0.999 | 4977 | tags=21%, list=9%, sign |
| GO ACUTE I | GO ACUTE INFLAMMAT | 23 | 0.6 | 1.694 | 0.03 | 0.0451 | 0.949 | 7243 | tags=43%, list=12%, sig |
| GO CD40 SI( | GO CD40 SIGNALING PA | 15 | 0.62 | 1.653 | 0.03 | 0.0585 | 0.974 | 2354 | tags=40%, list=4%, sign |
| GO NEGATI | GO NEGATIVE REGULAT | 19 | 0.59 | 1.587 | 0.03 | 0.0855 | 0.994 | 6181 | tags=32%, list=11%, sig |
| GO RESPON | GO RESPONSE TO GAMI | 53 | 0.45 | 1.605 | 0.031 | 0.0778 | 0.991 | 3397 | tags=25%, list=6%, sign |
| GO NEGATI | GO NEGATIVE REGULAT | 51 | 0.44 | 1.558 | 0.031 | 0.0996 | 0.996 | 6716 | tags=33%, list=12%, sig |
| GO REGULA | GO REGULATION OF PR | 24 | 0.53 | 1.595 | 0.031 | 0.0825 | 0.993 | 5482 | tags=29%, list=9%, sign |
| GO NEGATI | GO NEGATIVE REGULAT | 38 | 0.47 | 1.555 | 0.031 | 0.1013 | 0.996 | 2711 | tags=24%, list=5%, sign |
| GO DEOXYF | GO DEOXYRIBOSE PHO! | 20 | 0.59 | 1.656 | 0.031 | 0.0577 | 0.973 | 6751 | tags=50%, list=12%, sig |
| GO NEGATI | GO NEGATIVE REGULAT | 21 | 0.48 | 1.548 | 0.032 | 0.1044 | 0.998 | 9091 | tags=43%, list=16%, sig |
| GO FICOLIN | GO FICOLIN 1 RICH GRA | 57 | 0.52 | 1.677 | 0.032 | 0.0498 | 0.961 | 8559 | tags=47%, list=15%, sig |
| GO POSITIV | GO POSITIVE REGULATI | 30 | 0.52 | 1.656 | 0.032 | 0.0576 | 0.973 | 6161 | tags=37%, list=11%, sig |
| GO COATED | GO COATED VESICLE | 290 | 0.41 | 1.63 | 0.032 | 0.0678 | 0.985 | 4653 | tags=22%, list=8%, sign |
| GO NEGATI | GO NEGATIVE REGULAT | 20 | 0.61 | 1.655 | 0.032 | 0.0579 | 0.974 | 2489 | tags=40%, list=4%, sign |
| GO LRR DOI | GO LRR DOMAIN BINDII | 16 | 0.58 | 1.62 | 0.033 | 0.0718 | 0.987 | 3028 | tags=31%, list=5%, sign |
| GO SPECIFI( | GO SPECIFIC GRANULE | 89 | 0.48 | 1.702 | 0.033 | 0.0431 | 0.937 | 4639 | tags=35%, list=8%, sign |
| GO REGULA | GO REGULATION OF CH | 223 | 0.4 | 1.585 | 0.033 | 0.0863 | 0.994 | 6386 | tags=32%, list=11%, sig |
| GO CYTOKI | GO CYTOKINE BINDING | 134 | 0.51 | 1.655 | 0.033 | 0.0579 | 0.974 | 8001 | tags=37%, list=14%, sig |
| GO REGULA | GO REGULATION OF MA | 23 | 0.56 | 1.609 | 0.033 | 0.0763 | 0.989 | 6083 | tags=35%, list=10%, sig |
| GO IMMUN( | GO IMMUNOGLOBULIN | 23 | 0.67 | 1.651 | 0.033 | 0.0593 | 0.977 | 4939 | tags=43%, list=8%, sign |
| GO NEGATI | GO NEGATIVE REGULAT | 106 | 0.42 | 1.563 | 0.033 | 0.0981 | 0.996 | 3003 | tags=20%, list=5%, sign |
| GO PHAGOC | GO PHAGOCYTOSIS REC | 74 | 0.7 | 1.675 | 0.034 | 0.0505 | 0.966 | 7872 | tags=64%, list=13%, sig |
| GO DEFENS | GO DEFENSE RESPONSE | 41 | 0.52 | 1.544 | 0.034 | 0.1064 | 0.998 | 7298 | tags=39%, list=12%, sig |
| GO SIGNALI | GO SIGNALING RECEPT( | 41 | 0.47 | 1.568 | 0.034 | 0.0958 | 0.996 | 2179 | tags=29%, list=4%, sign |
| GO NEGATI | GO NEGATIVE REGULAT | 102 | 0.38 | 1.487 | 0.034 | 0.1404 | 0.999 | 7843 | tags=36%, list=13%, sig |
| GO HETERO | GO HETEROTYPIC CELL | 62 | 0.47 | 1.599 | 0.034 | 0.0805 | 0.992 | 7662 | tags=34%, list=13%, sig |
| GO PROTEII | GO PROTEIN TRIMERIZ/ | 17 | 0.55 | 1.556 | 0.034 | 0.1008 | 0.996 | 4152 | tags=29%, list=7%, sign |
| GO TRANS ( | GO TRANS GOLGI NETW | 225 | 0.39 | 1.587 | 0.034 | 0.0857 | 0.994 | 4982 | tags=24%, list=9%, sign |
| GO REGULA | GO REGULATION OF MC | 181 | 0.4 | 1.559 | 0.034 | 0.0992 | 0.996 | 3994 | tags=21%, list=7%, sign |
| GO PROTEII | GO PROTEIN PROCESSII | 213 | 0.35 | 1.449 | 0.035 | 0.1619 | 0.999 | 8256 | tags=28%, list=14%, sig |
| GO SIGNALI | GO SIGNALING ADAPTC | 67 | 0.44 | 1.597 | 0.035 | 0.0817 | 0.992 | 2179 | tags=25%, list=4%, sign |
| GO NEGATI | GO NEGATIVE REGULAT | 27 | 0.49 | 1.622 | 0.035 | 0.0707 | 0.987 | 7903 | tags=56%, list=14%, sig |
| GO REGULA | GO REGULATION OF SPI | 17 | 0.53 | 1.569 | 0.035 | 0.0951 | 0.996 | 6670 | tags=47%, list=11%, sig |
| GO PHAGOL | GO PHAGOLYSOSOME A | 15 | 0.54 | 1.554 | 0.035 | 0.1015 | 0.996 | 11162 | tags=73%, list=19%, sig |
| GO RESPON | GO RESPONSE TO OXID/ | 446 | 0.32 | 1.43 | 0.035 | 0.1736 | 0.999 | 5802 | tags=22%, list=10%, sig |
| GO CLATHR | GO CLATHRIN COATED | 190 | 0.38 | 1.51 | 0.036 | 0.1262 | 0.999 | 4653 | tags=22%, list=8%, sign |
| GO FOAM C | GO FOAM CELL DIFFERI | 37 | 0.5 | 1.61 | 0.037 | 0.076 | 0.989 | 2748 | tags=22%, list=5%, sign |
| GO ORGAN( | GO ORGANOPHOSPHAT | 141 | 0.34 | 1.414 | 0.037 | 0.185 | 0.999 | 8569 | tags=32%, list=15%, sig |
| GO POSITIV | GO POSITIVE REGULATI | 34 | 0.5 | 1.604 | 0.037 | 0.0781 | 0.991 | 7432 | tags=47%, list=13%, sig |
| GO PEPTIDY | GO PEPTIDYL TYROSINI | 373 | 0.36 | 1.487 | 0.037 | 0.1403 | 0.999 | 7949 | tags=30%, list=14%, sig |
| GO MAST CI | GO MAST CELL ACTIVA | 59 | 0.53 | 1.695 | 0.037 | 0.0448 | 0.946 | 3188 | tags=36%, list=5%, sign |
| GO DENDRI | GO DENDRITIC CELL CH | 24 | 0.6 | 1.646 | 0.037 | 0.0611 | 0.977 | 8001 | tags=50%, list=14%, sig |
| GO REGULA | GO REGULATION OF OX | 114 | 0.36 | 1.434 | 0.038 | 0.171 | 0.999 | 7462 | tags=29%, list=13%, sig |
| GO REGULA | GO REGULATION OF NE | 41 | 0.54 | 1.695 | 0.038 | 0.0447 | 0.946 | 6161 | tags=39%, list=11%, sig |
| GO LYSOSO | GO LYSOSOME LOCALI | 71 | 0.44 | 1.59 | 0.038 | 0.0847 | 0.994 | 7851 | tags=45%, list=13%, sig |
| GO AZUROP | GO AZUROPHIL GRANU | 150 | 0.43 | 1.624 | 0.038 | 0.07 | 0.986 | 9621 | tags=43%, list=16%, sig |
| GO REGULA | GO REGULATION OF HE | 26 | 0.53 | 1.602 | 0.038 | 0.0792 | 0.992 | 7662 | tags=42%, list=13%, sig |

| | | | | | | | | | |
|---|---|---|---|---|---|---|---|---|---|
| GO POSITIV | GO POSITIVE REGULATI | 149 | 0.42 | 1.568 | 0.038 | 0.0958 | 0.996 | 5416 | tags=26%, list=9%, sign |
| GO VESICLE | GO VESICLE ORGANIZA | 326 | 0.38 | 1.561 | 0.038 | 0.0989 | 0.996 | 4727 | tags=25%, list=8%, sign |
| GO EARLY E | GO EARLY ENDOSOME | 359 | 0.38 | 1.558 | 0.038 | 0.0995 | 0.996 | 4527 | tags=21%, list=8%, sign |
| GO MATURE | GO MATURE B CELL DIF | 22 | 0.59 | 1.542 | 0.038 | 0.1077 | 0.999 | 8719 | tags=50%, list=15%, sig |
| GO POSITIV | GO POSITIVE REGULATI | 23 | 0.51 | 1.548 | 0.039 | 0.1046 | 0.998 | 11877 | tags=57%, list=20%, sig |
| GO POSITIV | GO POSITIVE REGULATI | 20 | 0.51 | 1.529 | 0.039 | 0.1144 | 0.999 | 2807 | tags=25%, list=5%, sign |
| GO UNSATU | GO UNSATURATED FAT | 116 | 0.36 | 1.465 | 0.039 | 0.1525 | 0.999 | 7814 | tags=29%, list=13%, sig |
| GO POSITIV | GO POSITIVE REGULATI | 17 | 0.55 | 1.595 | 0.039 | 0.0825 | 0.993 | 7615 | tags=53%, list=13%, sig |
| GO NEGATI | GO NEGATIVE REGULAT | 16 | 0.6 | 1.577 | 0.039 | 0.0908 | 0.996 | 2873 | tags=25%, list=5%, sign |
| GO ENDOPL | GO ENDOPLASMIC RETI | 72 | 0.48 | 1.62 | 0.039 | 0.0717 | 0.987 | 2856 | tags=24%, list=5%, sign |
| GO UBIQUIT | GO UBIQUITIN LIKE PRO | 90 | 0.43 | 1.558 | 0.04 | 0.0995 | 0.996 | 2742 | tags=20%, list=5%, sign |
| GO RESPON | GO RESPONSE TO HYDR | 18 | 0.5 | 1.496 | 0.04 | 0.135 | 0.999 | 1641 | tags=28%, list=3%, sign |
| GO ERROR F | GO ERROR FREE TRANS | 21 | 0.63 | 1.603 | 0.04 | 0.0789 | 0.992 | 9018 | tags=52%, list=15%, sig |
| GO RESPON | GO RESPONSE TO OXYG | 387 | 0.33 | 1.439 | 0.041 | 0.1677 | 0.999 | 6166 | tags=23%, list=11%, sig |
| GO IRON IO | GO IRON ION HOMEOST | 84 | 0.38 | 1.478 | 0.041 | 0.1449 | 0.999 | 4769 | tags=23%, list=8%, sign |
| GO CELLUL | GO CELLULAR SENESCE | 75 | 0.43 | 1.535 | 0.041 | 0.1113 | 0.999 | 4893 | tags=23%, list=8%, sign |
| GO SYNCYT | GO SYNCYTIUM FORMA | 55 | 0.48 | 1.539 | 0.041 | 0.1087 | 0.999 | 3679 | tags=25%, list=6%, sign |
| GO POSITIV | GO POSITIVE REGULATI | 141 | 0.44 | 1.611 | 0.041 | 0.0755 | 0.989 | 6386 | tags=38%, list=11%, sig |
| GO SOMATI | GO SOMATIC DIVERSIFI | 70 | 0.47 | 1.563 | 0.041 | 0.0979 | 0.996 | 4441 | tags=27%, list=8%, sign |
| GO MORPHO | GO MORPHOGENESIS OI | 144 | 0.42 | 1.545 | 0.042 | 0.1059 | 0.998 | 5095 | tags=24%, list=9%, sign |
| GO MODULA | GO MODULATION BY HO | 17 | 0.58 | 1.601 | 0.042 | 0.0793 | 0.992 | 5615 | tags=35%, list=10%, sig |
| GO RRNA CA | GO RRNA CATABOLIC P | 20 | 0.56 | 1.624 | 0.042 | 0.07 | 0.986 | 11964 | tags=55%, list=20%, sig |
| GO REPLISO | GO REPLISOME | 26 | 0.63 | 1.645 | 0.042 | 0.0615 | 0.979 | 6794 | tags=46%, list=12%, sig |
| GO POST TR | GO POST TRANSLATION | 356 | 0.36 | 1.486 | 0.042 | 0.1408 | 0.999 | 4674 | tags=21%, list=8%, sign |
| GO PROTEA | GO PROTEASE BINDING | 133 | 0.41 | 1.521 | 0.042 | 0.1199 | 0.999 | 6375 | tags=34%, list=11%, sig |
| GO MYELOI | GO MYELOID CELL HOM | 150 | 0.37 | 1.493 | 0.042 | 0.137 | 0.999 | 5377 | tags=23%, list=9%, sign |
| GO POSITIV | GO POSITIVE REGULATI | 64 | 0.41 | 1.461 | 0.043 | 0.1541 | 0.999 | 9259 | tags=42%, list=16%, sig |
| GO POSITIV | GO POSITIVE REGULATI | 46 | 0.47 | 1.547 | 0.043 | 0.1046 | 0.998 | 2198 | tags=28%, list=4%, sign |
| GO B CELL I | GO B CELL DIFFERENTI | 135 | 0.44 | 1.549 | 0.044 | 0.1037 | 0.998 | 9075 | tags=38%, list=16%, sig |
| GO ANTIOX | GO ANTIOXIDANT ACTI | 79 | 0.41 | 1.505 | 0.045 | 0.1289 | 0.999 | 5400 | tags=28%, list=9%, sign |
| GO POSITIV | GO POSITIVE REGULATI | 19 | 0.54 | 1.555 | 0.045 | 0.1009 | 0.996 | 2052 | tags=26%, list=4%, sign |
| GO ICOSAN | GO ICOSANOID METABO | 121 | 0.35 | 1.426 | 0.045 | 0.1762 | 0.999 | 7814 | tags=29%, list=13%, sig |
| GO GLYCOS | GO GLYCOSYL COMPOU | 40 | 0.5 | 1.627 | 0.045 | 0.0689 | 0.985 | 7852 | tags=38%, list=13%, sig |
| GO HYDROI | GO HYDROLASE ACTIVI | 126 | 0.36 | 1.453 | 0.045 | 0.1593 | 0.999 | 6465 | tags=32%, list=11%, sig |
| GO REGULA | GO REGULATION OF SU | 22 | 0.57 | 1.603 | 0.045 | 0.0789 | 0.992 | 6444 | tags=50%, list=11%, sig |
| GO UBIQUIT | GO UBIQUITIN LIKE PRO | 307 | 0.4 | 1.577 | 0.045 | 0.0909 | 0.996 | 4306 | tags=23%, list=7%, sign |
| GO POSITIV | GO POSITIVE REGULATI | 80 | 0.43 | 1.534 | 0.045 | 0.1115 | 0.999 | 6506 | tags=31%, list=11%, sig |
| GO MONOSA | GO MONOSACCHARIDE | 64 | 0.43 | 1.482 | 0.045 | 0.1429 | 0.999 | 4282 | tags=25%, list=7%, sign |
| GO MYOBLA | GO MYOBLAST FUSION | 40 | 0.49 | 1.552 | 0.045 | 0.1022 | 0.996 | 3679 | tags=25%, list=6%, sign |
| GO REGULA | GO REGULATION OF CA | 97 | 0.42 | 1.534 | 0.046 | 0.1114 | 0.999 | 2723 | tags=21%, list=5%, sign |
| GO POSITIV | GO POSITIVE REGULATI | 20 | 0.61 | 1.624 | 0.046 | 0.07 | 0.986 | 3436 | tags=35%, list=6%, sign |
| GO DETOXI | GO DETOXIFICATION | 129 | 0.35 | 1.423 | 0.046 | 0.1786 | 0.999 | 6151 | tags=24%, list=11%, sig |
| GO PATTER | GO PATTERN RECOGNIT | 23 | 0.61 | 1.62 | 0.046 | 0.0718 | 0.987 | 4795 | tags=43%, list=8%, sign |
| GO PROTEIN | GO PROTEIN HOMOTETI | 59 | 0.4 | 1.458 | 0.047 | 0.1562 | 0.999 | 7756 | tags=27%, list=13%, sig |
| GO REGULA | GO REGULATION OF DE | 19 | 0.51 | 1.508 | 0.047 | 0.1269 | 0.999 | 4977 | tags=32%, list=9%, sign |
| GO BLOOD I | GO BLOOD MICROPART | 146 | 0.48 | 1.562 | 0.047 | 0.0983 | 0.996 | 7462 | tags=36%, list=13%, sig |
| GO POSITIV | GO POSITIVE REGULATI | 17 | 0.55 | 1.559 | 0.047 | 0.0992 | 0.996 | 8900 | tags=47%, list=15%, sig |
| GO ESTABL | GO ESTABLISHMENT OF | 123 | 0.45 | 1.579 | 0.047 | 0.0899 | 0.996 | 4089 | tags=24%, list=7%, sign |
| GO AZUROP | GO AZUROPHIL GRANU | 56 | 0.49 | 1.592 | 0.047 | 0.0838 | 0.994 | 4415 | tags=30%, list=8%, sign |
| GO REGULA | GO REGULATION OF ER | 49 | 0.44 | 1.508 | 0.047 | 0.1269 | 0.999 | 6297 | tags=29%, list=11%, sig |
| GO POSITIV | GO POSITIVE REGULATI | 127 | 0.43 | 1.608 | 0.047 | 0.0766 | 0.989 | 4469 | tags=24%, list=8%, sign |
| GO P38MAP | GO P38MAPK CASCADE | 51 | 0.41 | 1.446 | 0.048 | 0.1627 | 0.999 | 6093 | tags=35%, list=10%, sig |
| GO PHOSPH | GO PHOSPHATIDYLINOS | 38 | 0.46 | 1.498 | 0.048 | 0.1339 | 0.999 | 8059 | tags=42%, list=14%, sig |
| GO POSITIV | GO POSITIVE REGULATI | 35 | 0.49 | 1.49 | 0.048 | 0.1387 | 0.999 | 3679 | tags=20%, list=6%, sign |
| GO CELL RE | GO CELL REDOX HOME | 55 | 0.45 | 1.535 | 0.048 | 0.1112 | 0.999 | 5126 | tags=29%, list=9%, sign |
| GO PYRIMI | GO PYRIMIDINE DEOXY | 18 | 0.6 | 1.58 | 0.049 | 0.0893 | 0.996 | 7756 | tags=56%, list=13%, sig |
| GO NEGATI | GO NEGATIVE REGULAT | 36 | 0.47 | 1.478 | 0.049 | 0.145 | 0.999 | 2875 | tags=25%, list=5%, sign |
| GO NUCLEO | GO NUCLEOSIDE TRIPH | 61 | 0.4 | 1.482 | 0.049 | 0.1427 | 0.999 | 8789 | tags=39%, list=15%, sig |

| | | | | | | | | | |
|---|---|---|---|---|---|---|---|---|---|
| GO EXTRAC | GO EXTRACELLULAR V | 21 | 0.5 | 1.551 | 0.05 | 0.103 | 0.997 | 5938 | tags=33%, list=10%, sig |
| GO REACTI\ | GO REACTIVE OXYGEN | 122 | 0.37 | 1.469 | 0.05 | 0.1499 | 0.999 | 3483 | tags=24%, list=6%, sign |

al=61%
nal=63%
al=43%
al=47%
al=65%
al=50%
al=47%
al=40%
al=49%
al=45%
al=58%
nal=62%
al=48%
al=59%
al=43%
al=50%
al=47%
al=67%
al=40%
al=50%
al=42%
al=40%
al=54%
al=54%
nal=61%
al=56%
nal=47%
al=56%
nal=46%
al=46%
al=48%
al=49%
nal=56%
al=65%
al=47%
al=56%
al=52%
al=48%
al=42%
al=47%
nal=66%
al=40%
al=59%
nal=46%
nal=57%
al=43%
al=50%
nal=58%
al=49%
al=53%
al=54%
al=44%
nal=82%
al=61%
al=63%
al=56%
al=37%

al=43%
al=65%
al=67%
al=49%
al=69%
nal=53%
nal=49%
al=57%
nal=50%
al=33%
al=43%
al=44%
al=41%
al=43%
al=45%
nal=52%
nal=69%
al=89%
nal=47%
al=50%
al=39%
nal=59%
nal=68%
nal=76%
al=42%
al=42%
al=50%
nal=43%
al=42%
al=53%
nal=63%
nal=58%
al=44%
nal=51%
al=40%
al=39%
al=39%
al=50%
al=38%
nal=58%
al=45%
al=42%
al=38%
al=42%
al=60%
al=38%
al=65%
al=40%
al=42%
al=45%
al=43%
nal=73%
al=42%
al=47%
al=59%
al=77%
al=42%
nal=42%

;nal=56%
ıal=42%
ıal=39%
ıal=58%
;nal=65%
;nal=51%
;nal=43%
ıal=64%
ıal=36%
ıal=40%
ıal=39%
ıal=43%
ıal=44%
ıal=51%
ıal=63%
;nal=53%
ıal=37%
;nal=46%
ıal=51%
ıal=34%
;nal=52%
ıal=39%
ıal=41%
ıal=44%
ıal=35%
ıal=43%
;nal=55%
;nal=46%
;nal=46%
;nal=47%
ıal=41%
ıal=37%
ıal=38%
ıal=57%
;nal=52%
ıal=39%
ıal=53%
;nal=53%
;nal=73%
ıal=33%
;nal=47%
ıal=36%
;nal=56%
ıal=66%
ıal=44%
ıal=36%
;nal=45%
;nal=52%
;nal=42%
ıal=37%
ıal=73%
ıal=70%
ıal=51%
ıal=87%
ıal=39%
;nal=84%
;nal=33%
ıal=38%

nal=76%
al=60%
al=43%
al=49%
al=38%
al=46%
nal=44%
al=45%
al=44%
nal=39%
nal=46%
al=35%
al=59%
al=39%
al=45%
al=53%
al=34%
nal=44%
al=74%
nal=51%
al=53%
nal=66%
al=59%
al=39%
al=45%
al=59%
al=68%
nal=54%
al=46%
al=31%
nal=64%
al=89%
al=47%
al=72%
al=34%
al=38%
nal=66%
al=48%
nal=42%
al=87%
al=44%
al=41%
al=73%
al=69%
al=61%
al=54%
al=55%
nal=48%
nal=56%
al=65%
al=39%
al=45%
al=41%
al=67%
al=55%
al=33%
al=57%
al=47%

al=35%
al=62%
nal=54%
al=81%
nal=50%
nal=43%
nal=41%
al=39%
al=51%
al=81%
al=50%
al=41%
nal=46%
nal=57%
nal=86%
nal=42%
nal=60%
nal=49%
al=42%
al=49%
al=30%
al=48%
nal=62%
nal=42%
nal=46%
nal=77%
al=45%
al=34%
nal=68%
nal=65%
al=43%
al=46%
al=30%
al=50%
nal=70%
nal=56%
al=43%
nal=38%
al=49%
nal=75%
al=41%
nal=49%
al=48%
al=52%
nal=57%
al=44%
nal=53%
nal=49%
nal=57%
al=43%
nal=55%
nal=62%
al=52%
al=34%
al=41%
nal=80%
nal=44%
nal=67%

ıal=73%
;nal=55%
ıal=33%
;nal=39%
ıal=45%
ıal=32%
;nal=54%
ıal=51%
;nal=74%
ıal=34%
;nal=69%
;nal=71%
ıal=54%
;nal=55%
ıal=32%
;nal=72%
;nal=42%
ıal=53%
ıal=34%
ıal=25%
ıal=41%
ıal=38%
;nal=39%
ıal=36%
;nal=53%
ıal=66%
;nal=62%
ıal=57%
ıal=48%
;nal=35%
ıal=42%
;nal=62%
ıal=37%
ıal=53%
ıal=46%
ıal=44%
ıal=31%
;nal=59%
ıal=48%
ıal=48%
ıal=78%
;nal=39%
ıal=38%
ıal=34%
;nal=42%
ıal=53%
ıal=50%
;nal=37%
;nal=39%
ıal=43%
;nal=42%
;nal=54%
ıal=35%
;nal=55%
;nal=70%
;nal=76%
;nal=46%
ıal=32%

al=98%
al=39%
al=43%
nal=45%
al=41%
nal=45%
al=50%
al=34%
al=25%
al=32%
nal=69%
nal=54%
nal=33%
nal=39%
nal=39%
al=38%
nal=39%
nal=44%
al=30%
al=49%
al=40%
nal=39%
nal=36%
nal=39%
nal=32%
al=27%
nal=38%
nal=34%
nal=31%
nal=35%
al=31%
nal=29%
al=32%
nal=63%
nal=50%
nal=47%
nal=65%
al=50%
nal=61%
nal=78%
nal=33%
al=53%
al=48%
nal=58%
nal=47%
al=54%
al=33%
al=37%
al=28%
nal=34%
nal=34%
al=35%
al=29%
nal=45%
al=24%
nal=49%
al=48%
al=33%

nal=58%
nal=56%
al=39%
al=40%
nal=51%
al=46%
al=47%
al=31%
nal=33%
al=57%
nal=61%
al=51%
nal=72%
nal=61%
nal=52%
nal=52%
al=35%
al=42%
al=42%
al=43%
al=49%
nal=35%
nal=46%
al=38%
al=43%
al=41%
al=47%
al=30%
al=40%
al=35%
nal=68%
nal=30%
nal=53%
nal=37%
al=55%
al=33%
al=37%
al=28%
nal=51%
al=37%
nal=60%
al=47%
nal=31%
al=34%
al=65%
nal=60%
al=34%
al=37%
al=33%
nal=57%
al=33%
al=37%
nal=29%
al=46%
al=38%
al=53%
nal=55%
al=26%

nal=33%
al=33%
nal=59%
nal=76%
nal=44%
nal=53%
nal=49%
nal=40%
nal=55%
al=47%
al=50%
al=37%
nal=59%
al=43%
al=32%
al=47%
nal=50%
nal=54%
nal=56%
nal=49%
al=43%
al=29%
al=36%
al=36%
al=41%
al=49%
nal=61%
nal=77%
al=34%
al=25%
nal=44%
al=40%
nal=51%
nal=39%
al=41%
al=25%
al=37%
al=42%
nal=53%
al=29%
al=29%
nal=67%
nal=78%
al=46%
al=55%
al=33%
nal=53%
nal=46%
nal=42%
nal=42%
al=44%
al=36%
nal=67%
nal=66%
nal=71%
nal=38%
nal=42%
nal=47%

ıal=37%
;nal=48%
;nal=40%
;nal=59%
;nal=80%
ıal=31%
;nal=72%
ıal=39%
;nal=43%
;nal=42%
;nal=44%
ıal=31%
;nal=49%
;nal=58%
;nal=43%
ıal=37%
ıal=32%
;nal=49%
;nal=60%
ıal=30%
ıal=40%
;nal=43%
;nal=58%
ıal=28%
ıal=29%
ıal=44%
ıal=49%
;nal=70%
;nal=45%
;nal=61%
ıal=29%
ıal=20%
;nal=32%
;nal=38%
;nal=47%
ıal=28%
ıal=40%
ıal=33%
;nal=59%
ıal=33%
ıal=51%
ıal=43%
;nal=46%
;nal=78%
;nal=38%
;nal=47%
;nal=44%
ıal=45%
;nal=30%
ıal=48%
ıal=41%
;nal=42%
ıal=26%
;nal=47%
;nal=57%
;nal=37%
;nal=64%
;nal=34%

al=58%
al=46%
al=55%
al=22%
;nal=48%
;nal=74%
;nal=32%
;nal=31%
al=26%
al=28%
al=54%
;nal=36%
al=28%
al=46%
;nal=29%
;nal=57%
;nal=50%
al=51%
al=41%
al=27%
al=34%
;nal=60%
al=34%
;nal=44%
;nal=43%
;nal=79%
;nal=69%
;nal=53%
;nal=45%
al=46%
;nal=36%
al=30%
;nal=57%
;nal=60%
;nal=45%
;nal=42%
;nal=31%
;nal=37%
al=38%
;nal=48%
;nal=31%
;nal=40%
;nal=33%
al=32%
al=44%
;nal=86%
;nal=43%
;nal=47%
;nal=53%
al=28%
al=45%
;nal=46%
;nal=55%
;nal=50%
al=27%
;nal=67%
;nal=51%
al=55%

al=21%
al=37%
al=38%
al=40%
al=27%
al=34%
al=28%
nal=82%
nal=34%
nal=37%
al=33%
nal=32%
nal=93%
al=37%
nal=41%
al=35%
nal=42%
nal=35%
nal=44%
al=23%
nal=48%
nal=41%
nal=38%
nal=54%
al=37%
nal=66%
nal=46%
al=41%
al=31%
al=32%
al=37%
nal=54%
al=25%
nal=52%
nal=76%
nal=32%
al=49%
nal=39%
nal=32%
nal=37%
nal=47%
al=34%
al=26%
al=44%
nal=31%
nal=40%
nal=48%
al=22%
nal=55%
al=37%
nal=43%
nal=31%
al=30%
nal=55%
nal=32%
nal=66%
nal=48%
nal=61%

nal=52%
al=36%
al=38%
al=23%
nal=39%
nal=46%
al=33%
nal=65%
nal=36%
al=26%
nal=48%
nal=36%
al=32%
al=43%
nal=37%
nal=38%
al=29%
al=28%
al=34%
nal=83%
nal=55%
al=40%
al=35%
nal=36%
nal=82%
nal=35%
nal=36%
al=27%
nal=45%
nal=36%
nal=48%
al=22%
al=40%
al=32%
nal=58%
al=25%
nal=36%
nal=61%
nal=62%
nal=41%
nal=58%
nal=62%
nal=46%
nal=69%
nal=45%
al=38%
nal=37%
nal=45%
al=40%
al=29%
nal=52%
al=39%
nal=28%
al=40%
nal=49%
nal=38%
nal=65%
nal=46%

;nal=51%
ıal=32%
;nal=43%
;nal=54%
;nal=46%
;nal=39%
;nal=39%
;nal=39%
ıal=28%
;nal=48%
ıal=42%
ıal=23%
;nal=50%
ıal=42%
;nal=35%
ıal=26%
;nal=38%
ıal=32%
ıal=25%
;nal=57%
;nal=51%
;nal=55%
;nal=41%
ıal=24%
ıal=42%
ıal=33%
ıal=38%
;nal=36%
;nal=43%
;nal=39%
ıal=47%
ıal=21%
;nal=73%
;nal=45%
ıal=30%
;nal=42%
;nal=39%
ıal=32%
ıal=26%
ıal=22%
;nal=32%
ıal=26%
;nal=64%
;nal=53%
;nal=91%
;nal=24%
ıal=24%
ıal=23%
;nal=37%
;nal=54%
;nal=35%
ıal=38%
;nal=58%
;nal=33%
;nal=44%
;nal=52%
;nal=51%
;nal=49%

al=28%
al=28%
al=23%
nal=59%
nal=71%
al=26%
nal=34%
nal=61%
al=26%
al=25%
al=21%
al=29%
nal=62%
nal=26%
al=25%
al=25%
al=27%
nal=43%
al=29%
al=27%
nal=39%
nal=69%
nal=52%
al=22%
nal=38%
al=25%
nal=50%
al=29%
nal=45%
al=31%
al=27%
nal=33%
nal=43%
nal=36%
nal=56%
al=25%
nal=35%
al=27%
al=27%
al=22%
al=37%
nal=27%
al=47%
nal=31%
al=35%
nal=41%
nal=56%
al=25%
al=33%
nal=32%
al=26%
nal=39%
nal=49%
al=21%
al=32%
nal=64%
al=26%
nal=46%

ginal=37%
al=25%

# Supplementary Table S12
# HIGH-ETV7

| NAME | GS<br> fo | GS DETA | SIZE | ES | NES | NOM p | FDR q-va | FWER p- | RANK A | LEADING EDGE |
|---|---|---|---|---|---|---|---|---|---|---|
| KEGG_BAS | KEGG_BA | Details ... | 55 | -0.58 | -1.97 | 0.002 | 0.05303 | 0.046 | 11983 | tags=55%, list=21%, signal=69% |
| KEGG_HEI | KEGG_HE | Details ... | 56 | -0.51 | -1.79 | 0.006 | 0.20421 | 0.241 | 12159 | tags=50%, list=21%, signal=63% |
| KEGG_MEI | KEGG_MI | Details ... | 101 | -0.42 | -1.59 | 0.022 | 0.72224 | 0.653 | 10983 | tags=36%, list=19%, signal=44% |
| KEGG_WN | KEGG_W | Details ... | 150 | -0.41 | -1.58 | 0.024 | 0.58769 | 0.667 | 12664 | tags=41%, list=22%, signal=53% |
| KEGG_TGF | KEGG_TC | Details ... | 85 | -0.4 | -1.47 | 0.069 | 0.74151 | 0.82 | 10951 | tags=39%, list=19%, signal=48% |
| KEGG_GLY | KEGG_GI | Details ... | 31 | -0.48 | -1.49 | 0.073 | 0.8036 | 0.795 | 9508 | tags=32%, list=16%, signal=39% |
| KEGG_THY | KEGG_TH | Details ... | 29 | -0.42 | -1.36 | 0.098 | 0.76568 | 0.92 | 3454 | tags=24%, list=6%, signal=26% |
| KEGG_TAS | KEGG_TA | Details ... | 51 | -0.43 | -1.38 | 0.099 | 0.79558 | 0.913 | 13846 | tags=49%, list=24%, signal=64% |
| KEGG_GLY | KEGG_GI | Details ... | 43 | -0.36 | -1.33 | 0.101 | 0.69017 | 0.945 | 10468 | tags=33%, list=18%, signal=40% |
| KEGG_TER | KEGG_TE | Details ... | 15 | -0.57 | -1.44 | 0.107 | 0.73815 | 0.855 | 14002 | tags=60%, list=24%, signal=79% |
| KEGG_CYS | KEGG_CY | Details ... | 34 | -0.42 | -1.34 | 0.108 | 0.78905 | 0.941 | 2713 | tags=24%, list=5%, signal=25% |
| KEGG_ADI | KEGG_AI | Details ... | 68 | -0.4 | -1.38 | 0.121 | 0.85938 | 0.901 | 12976 | tags=43%, list=22%, signal=55% |
| KEGG_NEU | KEGG_NE | Details ... | 271 | -0.33 | -1.26 | 0.14 | 0.66414 | 0.973 | 16803 | tags=47%, list=29%, signal=65% |
| KEGG_CAI | KEGG_CA | Details ... | 177 | -0.34 | -1.27 | 0.151 | 0.75093 | 0.971 | 16796 | tags=48%, list=29%, signal=67% |
| KEGG_AXO | KEGG_AX | Details ... | 128 | -0.33 | -1.27 | 0.151 | 0.67795 | 0.971 | 11441 | tags=37%, list=20%, signal=46% |
| KEGG_VAS | KEGG_VA | Details ... | 112 | -0.35 | -1.27 | 0.173 | 0.71534 | 0.971 | 10983 | tags=37%, list=19%, signal=45% |
| KEGG_ARE | KEGG_AE | Details ... | 74 | -0.41 | -1.31 | 0.178 | 0.69452 | 0.956 | 5081 | tags=30%, list=9%, signal=33% |
| KEGG_STE | KEGG_ST | Details ... | 17 | -0.52 | -1.33 | 0.184 | 0.72927 | 0.943 | 3808 | tags=35%, list=7%, signal=38% |
| KEGG_CAE | KEGG_CA | Details ... | 78 | -0.36 | -1.22 | 0.193 | 0.67633 | 0.98 | 10114 | tags=35%, list=17%, signal=42% |
| KEGG_DIL | KEGG_DI | Details ... | 90 | -0.38 | -1.26 | 0.216 | 0.62984 | 0.973 | 8375 | tags=34%, list=14%, signal=40% |
| KEGG_TIG | KEGG_TI | Details ... | 129 | -0.3 | -1.16 | 0.216 | 0.6966 | 0.994 | 14188 | tags=36%, list=24%, signal=48% |
| KEGG_HYI | KEGG_HY | Details ... | 83 | -0.38 | -1.25 | 0.221 | 0.62664 | 0.974 | 9937 | tags=37%, list=17%, signal=45% |
| KEGG_GAI | KEGG_GA | Details ... | 88 | -0.33 | -1.18 | 0.239 | 0.69933 | 0.988 | 13078 | tags=35%, list=22%, signal=45% |
| KEGG_NIT | KEGG_NI | Details ... | 23 | -0.38 | -1.17 | 0.249 | 0.71056 | 0.993 | 8749 | tags=30%, list=15%, signal=36% |
| KEGG_ENI | KEGG_EN | Details ... | 52 | -0.35 | -1.19 | 0.256 | 0.71435 | 0.987 | 7373 | tags=25%, list=13%, signal=29% |
| KEGG_VAS | KEGG_VA | Details ... | 44 | -0.34 | -1.15 | 0.275 | 0.70916 | 0.996 | 14782 | tags=39%, list=25%, signal=52% |
| KEGG_MEI | KEGG_MI | Details ... | 71 | -0.33 | -1.13 | 0.276 | 0.72375 | 0.996 | 11361 | tags=37%, list=19%, signal=45% |
| KEGG_LON | KEGG_LC | Details ... | 68 | -0.3 | -1.11 | 0.279 | 0.71052 | 0.997 | 13078 | tags=40%, list=22%, signal=51% |
| KEGG_RNA | KEGG_RN | Details ... | 29 | -0.4 | -1.13 | 0.329 | 0.70202 | 0.996 | 7398 | tags=34%, list=13%, signal=39% |
| KEGG_PRO | KEGG_PR | Details ... | 89 | -0.29 | -1.08 | 0.338 | 0.69489 | 0.999 | 2978 | tags=17%, list=5%, signal=18% |
| KEGG_ECM | KEGG_EC | Details ... | 84 | -0.36 | -1.1 | 0.343 | 0.71284 | 0.997 | 5853 | tags=31%, list=10%, signal=34% |
| KEGG_VAI | KEGG_VA | Details ... | 43 | -0.35 | -1.08 | 0.349 | 0.7127 | 0.999 | 13739 | tags=40%, list=24%, signal=52% |
| KEGG_SEL | KEGG_SE | Details ... | 24 | -0.38 | -1.1 | 0.356 | 0.70687 | 0.997 | 1725 | tags=17%, list=3%, signal=17% |
| KEGG_FAT | KEGG_FA | Details ... | 42 | -0.33 | -1.08 | 0.36 | 0.69075 | 1 | 9927 | tags=26%, list=17%, signal=32% |
| KEGG_REN | KEGG_RE | Details ... | 17 | -0.4 | -1.07 | 0.368 | 0.66127 | 1 | 1992 | tags=18%, list=3%, signal=18% |
| KEGG_ALE | KEGG_AI | Details ... | 19 | -0.37 | -1.07 | 0.368 | 0.6795 | 1 | 8929 | tags=32%, list=15%, signal=37% |
| KEGG_LON | KEGG_LC | Details ... | 70 | -0.29 | -1.04 | 0.381 | 0.69626 | 1 | 14094 | tags=39%, list=24%, signal=51% |
| KEGG_BUT | KEGG_BU | Details ... | 34 | -0.32 | -1.04 | 0.395 | 0.70103 | 1 | 13739 | tags=35%, list=24%, signal=46% |
| KEGG_ALA | KEGG_AI | Details ... | 30 | -0.32 | -1.02 | 0.41 | 0.69777 | 1 | 10761 | tags=37%, list=18%, signal=45% |
| KEGG_INS | KEGG_INSULIN_SI | | 137 | -0.25 | -1.01 | 0.421 | 0.70997 | 1 | 12445 | tags=27%, list=21%, signal=34% |
| KEGG_REG | KEGG_REGULATIO | | 212 | -0.27 | -1.01 | 0.422 | 0.69781 | 1 | 12596 | tags=37%, list=22%, signal=47% |
| KEGG_PRO | KEGG_PR | Details ... | 32 | -0.34 | -1.02 | 0.425 | 0.70964 | 1 | 12175 | tags=28%, list=21%, signal=36% |
| KEGG_COI | KEGG_COLORECT | | 62 | -0.28 | -0.98 | 0.457 | 0.71236 | 1 | 7373 | tags=23%, list=13%, signal=26% |
| KEGG_MAT | KEGG_MATURITY | | 25 | -0.4 | -0.99 | 0.467 | 0.71104 | 1 | 20436 | tags=56%, list=35%, signal=86% |
| KEGG_PRO | KEGG_PROXIMAL | | 23 | -0.33 | -0.98 | 0.472 | 0.69988 | 1 | 10114 | tags=30%, list=17%, signal=37% |
| KEGG_N_G | KEGG_N_GLYCAN | | 46 | -0.31 | -0.96 | 0.483 | 0.70577 | 1 | 8949 | tags=26%, list=15%, signal=31% |
| KEGG_FOC | KEGG_FOCAL_AD | | 197 | -0.28 | -0.96 | 0.489 | 0.69583 | 1 | 14616 | tags=39%, list=25%, signal=51% |
| KEGG_PEN | KEGG_PENTOSE_ | | 28 | -0.33 | -0.95 | 0.495 | 0.69723 | 1 | 7161 | tags=21%, list=12%, signal=24% |
| KEGG_GLY | KEGG_GLYCEROP | | 72 | -0.25 | -0.97 | 0.498 | 0.70235 | 1 | 9719 | tags=24%, list=17%, signal=28% |
| KEGG_ETH | KEGG_ETHER_LIP | | 30 | -0.28 | -0.94 | 0.506 | 0.70127 | 1 | 9494 | tags=23%, list=16%, signal=28% |
| KEGG_BIO | KEGG_BIOSYNTHI | | 20 | -0.35 | -0.94 | 0.541 | 0.68942 | 1 | 8137 | tags=25%, list=14%, signal=29% |
| KEGG_ALI | KEGG_ALDOSTER | | 42 | -0.29 | -0.94 | 0.552 | 0.68205 | 1 | 13846 | tags=38%, list=24%, signal=50% |
| KEGG_CEI | KEGG_CELL_CYCI | | 124 | -0.27 | -0.85 | 0.588 | 0.78302 | 1 | 6335 | tags=21%, list=11%, signal=23% |
| KEGG_OOC | KEGG_OOCYTE_M | | 111 | -0.25 | -0.88 | 0.598 | 0.76009 | 1 | 10983 | tags=24%, list=19%, signal=30% |
| KEGG_RET | KEGG_RETINOL_M | | 64 | -0.26 | -0.88 | 0.625 | 0.7717 | 1 | 12457 | tags=30%, list=21%, signal=38% |
| KEGG_GLY | KEGG_GLYCOSAM | | 26 | -0.28 | -0.88 | 0.628 | 0.77349 | 1 | 14148 | tags=42%, list=24%, signal=56% |
| KEGG_PRI | KEGG_PRIMARY_ | | 16 | -0.32 | -0.84 | 0.654 | 0.79428 | 1 | 3897 | tags=19%, list=7%, signal=20% |

| | | | | | | | | | |
|---|---|---|---|---|---|---|---|---|---|
| KEGG_BET | KEGG_BETA_ALA | 22 | -0.28 | -0.86 | 0.655 | 0.78172 | 1 | 10225 | tags=32%, list=18%, signal=39% |
| KEGG_PRC | KEGG_PROGESTE | 85 | -0.23 | -0.82 | 0.682 | 0.80926 | 1 | 7944 | tags=21%, list=14%, signal=24% |
| KEGG_AM | KEGG_AMINOACY | 22 | -0.28 | -0.78 | 0.692 | 0.80953 | 1 | 15754 | tags=36%, list=27%, signal=50% |
| KEGG_VIB | KEGG_VIBRIO_CH | 53 | -0.25 | -0.83 | 0.703 | 0.78874 | 1 | 12270 | tags=26%, list=21%, signal=33% |
| KEGG_DRU | KEGG_DRUG_MET | 71 | -0.23 | -0.81 | 0.713 | 0.79332 | 1 | 12457 | tags=24%, list=21%, signal=30% |
| KEGG_LYS | KEGG_LYSINE_DE | 39 | -0.26 | -0.8 | 0.718 | 0.79555 | 1 | 15507 | tags=33%, list=27%, signal=45% |
| KEGG_ASC | KEGG_ASCORBAT | 25 | -0.27 | -0.79 | 0.721 | 0.80873 | 1 | 7161 | tags=20%, list=12%, signal=23% |
| KEGG_BAS | KEGG_BASAL_TR | 35 | -0.24 | -0.73 | 0.746 | 0.85078 | 1 | 8359 | tags=20%, list=14%, signal=23% |
| KEGG_O_C | KEGG_O_GLYCAN | 29 | -0.26 | -0.77 | 0.778 | 0.81067 | 1 | 6278 | tags=21%, list=11%, signal=23% |
| KEGG_GLY | KEGG_GLYCOSAM | 15 | -0.28 | -0.74 | 0.787 | 0.84923 | 1 | 6384 | tags=20%, list=11%, signal=22% |
| KEGG_OLF | KEGG_OLFACTOR | 387 | -0.23 | -0.81 | 0.788 | 0.7986 | 1 | 25693 | tags=44%, list=44%, signal=78% |
| KEGG_MET | KEGG_METABOLI | 69 | -0.21 | -0.71 | 0.843 | 0.85804 | 1 | 16652 | tags=30%, list=29%, signal=43% |
| KEGG_GLY | KEGG_GLYCOSAM | 22 | -0.23 | -0.62 | 0.871 | 0.93712 | 1 | 13687 | tags=32%, list=23%, signal=42% |
| KEGG_PPA | KEGG_PPAR_SIGN | 69 | -0.2 | -0.72 | 0.882 | 0.85736 | 1 | 16885 | tags=32%, list=29%, signal=45% |
| KEGG_TYF | KEGG_TYROSINE_ | 40 | -0.2 | -0.66 | 0.953 | 0.90538 | 1 | 21853 | tags=40%, list=37%, signal=64% |
| KEGG_STE | KEGG_STEROID_F | 55 | -0.17 | -0.59 | 0.987 | 0.94646 | 1 | 21547 | tags=40%, list=37%, signal=63% |

# LOW-ETV7

| NAME | GS<br> f | GS DETAI | SIZE | ES | NES | NOM p | FDR q-val | FWER p | RANK / | LEADING EDGE |
|---|---|---|---|---|---|---|---|---|---|---|
| KEGG_R | KEGG_F | Details ... | 69 | 0.7394 | 2.5376 | 0 | 0 | 0 | 5113 | tags=46%, list=9%, signal=51% |
| KEGG_T( | KEGG_T | Details ... | 102 | 0.6917 | 2.4542 | 0 | 0 | 0 | 5230 | tags=45%, list=9%, signal=49% |
| KEGG_A | KEGG_A | Details ... | 81 | 0.8407 | 2.4412 | 0 | 0 | 0 | 1969 | tags=48%, list=3%, signal=50% |
| KEGG_N | KEGG_N | Details ... | 132 | 0.6877 | 2.3568 | 0 | 0 | 0 | 4152 | tags=44%, list=7%, signal=47% |
| KEGG_C | KEGG_C | Details ... | 53 | 0.6957 | 2.3341 | 0 | 0 | 0 | 4772 | tags=43%, list=8%, signal=47% |
| KEGG_LI | KEGG_L | Details ... | 70 | 0.7787 | 2.3015 | 0 | 0 | 0 | 5416 | tags=54%, list=9%, signal=60% |
| KEGG_A | KEGG_A | Details ... | 50 | 0.8365 | 2.2788 | 0 | 0 | 0 | 1416 | tags=48%, list=2%, signal=49% |
| KEGG_T | KEGG_T | Details ... | 41 | 0.8633 | 2.1907 | 0 | 2.04E-04 | 0.002 | 1416 | tags=61%, list=2%, signal=62% |
| KEGG_JA | KEGG_J | Details ... | 155 | 0.592 | 2.1886 | 0 | 1.81E-04 | 0.002 | 3951 | tags=35%, list=7%, signal=37% |
| KEGG_G | KEGG_C | Details ... | 37 | 0.9237 | 2.1668 | 0 | 3.76E-04 | 0.005 | 1416 | tags=68%, list=2%, signal=69% |
| KEGG_N | KEGG_N | Details ... | 61 | 0.6433 | 2.1321 | 0 | 6.07E-04 | 0.007 | 4938 | tags=39%, list=8%, signal=43% |
| KEGG_IN | KEGG_I | Details ... | 46 | 0.8277 | 2.131 | 0 | 5.56E-04 | 0.007 | 1479 | tags=50%, list=3%, signal=51% |
| KEGG_PI | KEGG_P | Details ... | 35 | 0.8694 | 2.1239 | 0 | 5.14E-04 | 0.007 | 2519 | tags=63%, list=4%, signal=66% |
| KEGG_PI | KEGG_P | Details ... | 45 | 0.8184 | 2.1067 | 0 | 4.77E-04 | 0.007 | 2770 | tags=47%, list=5%, signal=49% |
| KEGG_V | KEGG_V | Details ... | 68 | 0.7075 | 2.0924 | 0 | 6.13E-04 | 0.009 | 1499 | tags=40%, list=3%, signal=41% |
| KEGG_A | KEGG_A | Details ... | 35 | 0.9048 | 2.0896 | 0 | 5.75E-04 | 0.009 | 1416 | tags=71%, list=2%, signal=73% |
| KEGG_C | KEGG_C | Details ... | 262 | 0.6056 | 2.078 | 0 | 6.88E-04 | 0.01 | 4938 | tags=38%, list=8%, signal=42% |
| KEGG_A | KEGG_A | Details ... | 28 | 0.833 | 2.0584 | 0 | 8.11E-04 | 0.013 | 2354 | tags=54%, list=4%, signal=56% |
| KEGG_A | KEGG_A | Details ... | 87 | 0.564 | 2.049 | 0 | 0.001024 | 0.016 | 7295 | tags=41%, list=12%, signal=47% |
| KEGG_S | KEGG_S | Details ... | 55 | 0.7761 | 2.0476 | 0 | 9.72E-04 | 0.016 | 4993 | tags=58%, list=9%, signal=64% |
| KEGG_C | KEGG_C | Details ... | 128 | 0.6082 | 1.965 | 0 | 0.003656 | 0.055 | 2424 | tags=33%, list=4%, signal=34% |
| KEGG_T_ | KEGG_T | Details ... | 107 | 0.5798 | 1.974 | 0.002 | 0.00337 | 0.052 | 2962 | tags=34%, list=5%, signal=35% |
| KEGG_SI | KEGG_S | Details ... | 38 | 0.5253 | 1.7411 | 0.002 | 0.037199 | 0.349 | 6323 | tags=42%, list=11%, signal=47% |
| KEGG_B | KEGG_E | Details ... | 41 | 0.4665 | 1.6264 | 0.0061 | 0.071072 | 0.561 | 4779 | tags=29%, list=8%, signal=32% |
| KEGG_H | KEGG_F | Details ... | 85 | 0.6379 | 1.8874 | 0.0061 | 0.009901 | 0.121 | 6871 | tags=41%, list=12%, signal=47% |
| KEGG_EI | KEGG_E | Details ... | 179 | 0.3949 | 1.6193 | 0.0062 | 0.070835 | 0.575 | 4152 | tags=22%, list=7%, signal=24% |
| KEGG_A | KEGG_A | Details ... | 53 | 0.4677 | 1.6904 | 0.0062 | 0.051639 | 0.439 | 3679 | tags=28%, list=6%, signal=30% |
| KEGG_P | KEGG_P | Details ... | 69 | 0.4749 | 1.707 | 0.008 | 0.046557 | 0.408 | 5416 | tags=32%, list=9%, signal=35% |
| KEGG_C | KEGG_C | Details ... | 185 | 0.5026 | 1.8164 | 0.0119 | 0.019633 | 0.21 | 4110 | tags=30%, list=7%, signal=32% |
| KEGG_A | KEGG_A | Details ... | 67 | 0.4803 | 1.7095 | 0.0127 | 0.047067 | 0.4 | 4938 | tags=25%, list=8%, signal=28% |
| KEGG_A | KEGG_A | Details ... | 57 | 0.4664 | 1.6575 | 0.0173 | 0.06029 | 0.509 | 4779 | tags=32%, list=8%, signal=34% |
| KEGG_R | KEGG_F | Details ... | 34 | 0.5204 | 1.6616 | 0.0179 | 0.060155 | 0.498 | 10204 | tags=38%, list=17%, signal=46% |
| KEGG_P | KEGG_P | Details ... | 66 | 0.4639 | 1.6804 | 0.0195 | 0.053874 | 0.459 | 7460 | tags=38%, list=13%, signal=43% |
| KEGG_L | KEGG_L | Details ... | 121 | 0.5155 | 1.7594 | 0.0204 | 0.033567 | 0.318 | 8480 | tags=48%, list=15%, signal=56% |
| KEGG_P | KEGG_P | Details ... | 98 | 0.4734 | 1.6441 | 0.0213 | 0.064283 | 0.534 | 9339 | tags=48%, list=16%, signal=57% |
| KEGG_F( | KEGG_F | Details ... | 79 | 0.4188 | 1.5818 | 0.0267 | 0.086035 | 0.629 | 3679 | tags=27%, list=6%, signal=28% |
| KEGG_N | KEGG_N | Details ... | 24 | 0.4585 | 1.4983 | 0.0368 | 0.132922 | 0.756 | 13070 | tags=54%, list=22%, signal=70% |
| KEGG_B | KEGG_E | Details ... | 75 | 0.5096 | 1.6259 | 0.0435 | 0.069279 | 0.561 | 5312 | tags=31%, list=9%, signal=34% |
| KEGG_N | KEGG_N | Details ... | 44 | 0.5645 | 1.6102 | 0.0438 | 0.073587 | 0.588 | 9018 | tags=45%, list=15%, signal=54% |
| KEGG_D | KEGG_DRUG_ME | | 51 | 0.41 | 1.4066 | 0.0631 | 0.205037 | 0.879 | 10228 | tags=39%, list=18%, signal=48% |
| KEGG_EI | KEGG_EPITHELIA | | 68 | 0.3931 | 1.4265 | 0.0707 | 0.189534 | 0.857 | 7659 | tags=34%, list=13%, signal=39% |
| KEGG_A | KEGG_ABC_TRAN | | 44 | 0.4087 | 1.3775 | 0.071 | 0.211885 | 0.905 | 2599 | tags=14%, list=4%, signal=14% |
| KEGG_F( | KEGG_FC_GAMM | | 93 | 0.3907 | 1.452 | 0.0782 | 0.168368 | 0.828 | 5312 | tags=32%, list=9%, signal=35% |
| KEGG_P | KEGG_PANTOTHE | | 16 | 0.4875 | 1.3982 | 0.0811 | 0.204622 | 0.887 | 5260 | tags=44%, list=9%, signal=48% |
| KEGG_D | KEGG_D | Details ... | 36 | 0.647 | 1.5374 | 0.0853 | 0.108318 | 0.695 | 9659 | tags=64%, list=17%, signal=77% |
| KEGG_G | KEGG_GALACTOS | | 26 | 0.442 | 1.3508 | 0.099 | 0.230996 | 0.928 | 7247 | tags=38%, list=12%, signal=44% |
| KEGG_A | KEGG_ALZHEIME | | 152 | 0.3581 | 1.3977 | 0.1066 | 0.200529 | 0.887 | 6375 | tags=32%, list=11%, signal=36% |
| KEGG_C( | KEGG_COMPLEM | | 69 | 0.4625 | 1.399 | 0.1377 | 0.208358 | 0.886 | 7209 | tags=36%, list=12%, signal=41% |
| KEGG_SI | KEGG_SMALL_CE | | 84 | 0.3825 | 1.3679 | 0.1389 | 0.217411 | 0.917 | 4893 | tags=25%, list=8%, signal=27% |
| KEGG_B | KEGG_BASE_EXC | | 34 | 0.4945 | 1.3973 | 0.14 | 0.19669 | 0.889 | 8950 | tags=53%, list=15%, signal=62% |
| KEGG_V | KEGG_VEGF_SIGN | | 76 | 0.3093 | 1.2198 | 0.1772 | 0.339008 | 0.982 | 3740 | tags=22%, list=6%, signal=24% |
| KEGG_N | KEGG_NON_SMAI | | 54 | 0.3484 | 1.2151 | 0.188 | 0.333643 | 0.983 | 5194 | tags=24%, list=9%, signal=26% |
| KEGG_N | KEGG_NEUROTR( | | 125 | 0.323 | 1.2325 | 0.1952 | 0.353451 | 0.978 | 3679 | tags=18%, list=6%, signal=20% |
| KEGG_PI | KEGG_PEROXISO | | 78 | 0.352 | 1.2296 | 0.2065 | 0.35091 | 0.98 | 7807 | tags=31%, list=13%, signal=35% |
| KEGG_C | KEGG_CHRONIC_ | | 73 | 0.3363 | 1.2255 | 0.2102 | 0.343847 | 0.98 | 5416 | tags=26%, list=9%, signal=29% |
| KEGG_O | KEGG_OTHER_GL | | 16 | 0.4972 | 1.2602 | 0.2162 | 0.329655 | 0.974 | 8380 | tags=50%, list=14%, signal=58% |
| KEGG_A | KEGG_ARGININE_ | | 53 | 0.3261 | 1.1719 | 0.2237 | 0.356609 | 0.992 | 3608 | tags=19%, list=6%, signal=20% |

| | | | | | | | | | |
|---|---|---|---|---|---|---|---|---|---|
| KEGG_A | KEGG_AMINO_SU | 43 | 0.3811 | 1.2222 | 0.2283 | 0.34227 | 0.981 | 3815 | tags=28%, list=7%, signal=30% |
| KEGG_G | KEGG_GLYCOSA | 21 | 0.4386 | 1.2359 | 0.2305 | 0.35566 | 0.978 | 2583 | tags=33%, list=4%, signal=35% |
| KEGG_G | KEGG_GLUTATHI | 49 | 0.348 | 1.2022 | 0.2341 | 0.345267 | 0.986 | 6970 | tags=29%, list=12%, signal=32% |
| KEGG_G | KEGG_GLYCOSPH | 26 | 0.3747 | 1.1878 | 0.2377 | 0.353601 | 0.989 | 4615 | tags=19%, list=8%, signal=21% |
| KEGG_H | KEGG_HUNTINGT | 167 | 0.3224 | 1.2172 | 0.2379 | 0.336581 | 0.983 | 5427 | tags=27%, list=9%, signal=30% |
| KEGG_L | KEGG_LEUKOCY | 115 | 0.3317 | 1.1774 | 0.2451 | 0.355289 | 0.992 | 8107 | tags=35%, list=14%, signal=40% |
| KEGG_M | KEGG_MISMATCH | 23 | 0.5333 | 1.2709 | 0.251 | 0.321072 | 0.97 | 9018 | tags=43%, list=15%, signal=51% |
| KEGG_P | KEGG_PARKINSO | 113 | 0.3731 | 1.2295 | 0.2531 | 0.344741 | 0.98 | 5427 | tags=28%, list=9%, signal=31% |
| KEGG_G | KEGG_GLYCOSPH | 15 | 0.455 | 1.1988 | 0.2551 | 0.344585 | 0.986 | 4888 | tags=27%, list=8%, signal=29% |
| KEGG_D | KEGG_DORSO_VE | 24 | 0.3976 | 1.1779 | 0.2651 | 0.360145 | 0.992 | 2282 | tags=8%, list=4%, signal=9% |
| KEGG_A | KEGG_ARACHIDO | 58 | 0.3075 | 1.1246 | 0.2725 | 0.399014 | 0.995 | 8244 | tags=31%, list=14%, signal=36% |
| KEGG_T | KEGG_TRYPTOPH | 39 | 0.3418 | 1.1246 | 0.2806 | 0.404691 | 0.995 | 6419 | tags=23%, list=11%, signal=26% |
| KEGG_N | KEGG_NOTCH_SI | 47 | 0.3125 | 1.1051 | 0.2916 | 0.420731 | 0.996 | 5143 | tags=23%, list=9%, signal=26% |
| KEGG_U | KEGG_UBIQUITIN | 130 | 0.3133 | 1.1277 | 0.3057 | 0.406363 | 0.995 | 6666 | tags=20%, list=11%, signal=23% |
| KEGG_O | KEGG_OXIDATIV | 115 | 0.3576 | 1.1444 | 0.3144 | 0.389029 | 0.994 | 9341 | tags=43%, list=16%, signal=51% |
| KEGG_M | KEGG_MTOR_SIG | 51 | 0.3071 | 1.0992 | 0.3156 | 0.423348 | 0.996 | 5619 | tags=24%, list=10%, signal=26% |
| KEGG_P | KEGG_PATHWAY | 322 | 0.2668 | 1.0943 | 0.3288 | 0.424847 | 0.996 | 6436 | tags=21%, list=11%, signal=23% |
| KEGG_F | KEGG_FRUCTOSE | 33 | 0.3345 | 1.0773 | 0.334 | 0.431541 | 0.998 | 5318 | tags=24%, list=9%, signal=27% |
| KEGG_G | KEGG_GLYCOLYS | 62 | 0.3122 | 1.0749 | 0.3373 | 0.429558 | 0.998 | 4426 | tags=19%, list=8%, signal=21% |
| KEGG_M | KEGG_MAPK_SIG | 266 | 0.2571 | 1.0556 | 0.3387 | 0.451136 | 0.999 | 6872 | tags=22%, list=12%, signal=25% |
| KEGG_P | KEGG_PRION_DIS | 35 | 0.3444 | 1.0835 | 0.3707 | 0.428877 | 0.997 | 7814 | tags=29%, list=13%, signal=33% |
| KEGG_P | KEGG_PHENYLAL | 17 | 0.3655 | 1.054 | 0.3768 | 0.447627 | 0.999 | 181 | tags=6%, list=0%, signal=6% |
| KEGG_P | KEGG_PHOSPHAT | 76 | 0.2861 | 1.033 | 0.3829 | 0.466592 | 0.999 | 5659 | tags=20%, list=10%, signal=22% |
| KEGG_O | KEGG_ONE_CARE | 17 | 0.4098 | 1.0836 | 0.3919 | 0.434583 | 0.997 | 9467 | tags=41%, list=16%, signal=49% |
| KEGG_R | KEGG_RENAL_CE | 66 | 0.3001 | 1.0382 | 0.4044 | 0.464795 | 0.999 | 5822 | tags=24%, list=10%, signal=27% |
| KEGG_P | KEGG_PURINE_M | 154 | 0.2544 | 0.9892 | 0.4561 | 0.527215 | 0.999 | 8661 | tags=32%, list=15%, signal=37% |
| KEGG_G | KEGG_GLYOXYL | 16 | 0.3689 | 0.9764 | 0.4646 | 0.528011 | 0.999 | 12323 | tags=50%, list=21%, signal=63% |
| KEGG_S | KEGG_SPLICEOS | 126 | 0.3223 | 0.9783 | 0.4656 | 0.531249 | 0.999 | 11437 | tags=33%, list=20%, signal=40% |
| KEGG_E | KEGG_ERBB_SIG | 86 | 0.2645 | 0.9879 | 0.4672 | 0.52283 | 0.999 | 5194 | tags=19%, list=9%, signal=20% |
| KEGG_G | KEGG_GNRH_SIG | 101 | 0.2425 | 0.9733 | 0.4835 | 0.526375 | 0.999 | 8278 | tags=25%, list=14%, signal=29% |
| KEGG_P | KEGG_PATHOGEN | 56 | 0.2785 | 0.9432 | 0.5108 | 0.550449 | 0.999 | 5537 | tags=27%, list=9%, signal=30% |
| KEGG_T | KEGG_TYPE_II_D | 47 | 0.2758 | 0.948 | 0.5245 | 0.555092 | 0.999 | 5619 | tags=21%, list=10%, signal=24% |
| KEGG_G | KEGG_GLIOMA | 65 | 0.265 | 0.947 | 0.534 | 0.550309 | 0.999 | 5346 | tags=18%, list=9%, signal=20% |
| KEGG_P | KEGG_PORPHYRI | 40 | 0.2837 | 0.9299 | 0.5405 | 0.564787 | 0.999 | 4216 | tags=18%, list=7%, signal=19% |
| KEGG_R | KEGG_RIBOSOME | 88 | 0.4374 | 0.9531 | 0.5775 | 0.552912 | 0.999 | 11521 | tags=45%, list=20%, signal=57% |
| KEGG_P | KEGG_PROTEIN_ | 23 | 0.312 | 0.8814 | 0.5849 | 0.626644 | 1 | 10237 | tags=35%, list=18%, signal=42% |
| KEGG_S | KEGG_SPHINGOL | 36 | 0.2718 | 0.8874 | 0.5965 | 0.624103 | 1 | 4766 | tags=25%, list=8%, signal=27% |
| KEGG_P | KEGG_PENTOSE_ | 27 | 0.2855 | 0.8512 | 0.649 | 0.670163 | 1 | 3816 | tags=19%, list=7%, signal=20% |
| KEGG_P | KEGG_PYRUVATE | 40 | 0.2721 | 0.8461 | 0.6586 | 0.671998 | 1 | 11307 | tags=38%, list=19%, signal=46% |
| KEGG_S | KEGG_STARCH_A | 52 | 0.2446 | 0.8449 | 0.6816 | 0.666834 | 1 | 10144 | tags=33%, list=17%, signal=40% |
| KEGG_H | KEGG_HOMOLOG | 26 | 0.2894 | 0.753 | 0.6929 | 0.790617 | 1 | 6781 | tags=27%, list=12%, signal=30% |
| KEGG_L | KEGG_LINOLEIC_ | 29 | 0.2666 | 0.8197 | 0.7064 | 0.699062 | 1 | 8059 | tags=31%, list=14%, signal=36% |
| KEGG_I | KEGG_INOSITOL_ | 54 | 0.2301 | 0.7987 | 0.7079 | 0.726346 | 1 | 3391 | tags=13%, list=6%, signal=14% |
| KEGG_R | KEGG_RNA_DEGR | 56 | 0.2251 | 0.7143 | 0.7945 | 0.839877 | 1 | 4005 | tags=14%, list=7%, signal=15% |
| KEGG_C | KEGG_CITRATE_ | 31 | 0.2432 | 0.645 | 0.8168 | 0.897515 | 1 | 11160 | tags=35%, list=19%, signal=44% |
| KEGG_G | KEGG_GLYCOSYI | 25 | 0.2278 | 0.6621 | 0.8819 | 0.888242 | 1 | 11700 | tags=40%, list=20%, signal=50% |
| KEGG_H | KEGG_HISTIDINE_ | 28 | 0.2106 | 0.6727 | 0.899 | 0.884859 | 1 | 3608 | tags=11%, list=6%, signal=11% |

# Supplementary Table S13

| id | StromalScore | ImmuneScore | ESTIMATEScore |
|---|---|---|---|
| TCGA-ZF-AA51-01A-21R-A39I-07 | 32.78758553 | 1444.819214 | 1477.6068 |
| TCGA-BT-A42C-01A-11R-A23N-07 | -1816.216696 | -483.7612023 | -2299.977898 |
| TCGA-G2-A3IB-01A-11R-A20F-07 | -1518.143937 | -225.0357511 | -1743.179688 |
| TCGA-2F-A9KW-01A-11R-A38B-07 | -48.54229396 | 356.7002633 | 308.1579693 |
| TCGA-XF-AAME-01A-12R-A42T-07 | 1578.90321 | 2416.129621 | 3995.032831 |
| TCGA-XF-AAN0-01A-11R-A42T-07 | -478.6836735 | 905.2061996 | 426.5225261 |
| TCGA-CF-A47W-01A-11R-A23W-07 | -1716.804659 | -427.4608438 | -2144.265503 |
| TCGA-DK-A1A7-01A-11R-A13Y-07 | -1140.910945 | 14.5936377 | -1126.317308 |
| TCGA-PQ-A6FN-01A-11R-A31N-07 | -581.3200502 | 695.3542011 | 114.0341509 |
| TCGA-XF-AAMY-01A-11R-A42T-07 | 160.4655591 | 170.5982823 | 331.0638414 |
| TCGA-G2-AA3F-01A-12R-A42T-07 | -1186.437579 | -504.1818732 | -1690.619452 |
| TCGA-FJ-A871-01A-11R-A352-07 | -855.7657065 | -553.759908 | -1409.525614 |
| TCGA-GC-A3RB-01A-12R-A220-07 | -1391.079424 | -236.0362272 | -1627.115651 |
| TCGA-UY-A9PF-01A-11R-A38B-07 | -492.9209929 | 439.8202686 | -53.1007243 |
| TCGA-YC-A89H-01A-11R-A36F-07 | -696.6120188 | 326.9249216 | -369.6870972 |
| TCGA-DK-A6AW-01A-11R-A30C-07 | -1173.620338 | -350.3122251 | -1523.932563 |
| TCGA-FD-A43N-01A-11R-A23W-07 | -70.3616635 | 321.5900412 | 251.2283777 |
| TCGA-UY-A8OD-01A-11R-A36F-07 | 535.1499961 | 957.2512666 | 1492.401263 |
| TCGA-2F-A9KT-01A-11R-A38B-07 | -992.4049851 | -140.5560666 | -1132.961052 |
| TCGA-CU-A0YR-01A-12R-A10U-07 | 100.2806991 | 1292.605424 | 1392.886123 |
| TCGA-4Z-AA84-01A-11R-A39I-07 | -1061.160717 | -394.0577722 | -1455.218489 |
| TCGA-CF-A27C-01A-11R-A16R-07 | -2243.149142 | -473.6248049 | -2716.773947 |
| TCGA-ZF-AA5N-01A-11R-A42T-07 | -1576.902874 | -206.0772239 | -1782.980098 |
| TCGA-DK-AA6U-01A-11R-A39I-07 | -1946.190087 | -454.0905119 | -2400.280599 |
| TCGA-GD-A3OS-01A-12R-A220-07 | -388.7079012 | -97.09982404 | -485.8077253 |
| TCGA-4Z-AA82-01A-11R-A39I-07 | -209.9392227 | 316.0597709 | 106.1205482 |
| TCGA-DK-AA76-01A-11R-A39I-07 | -1893.2902 | -1172.424071 | -3065.714271 |
| TCGA-DK-A3IL-01A-11R-A20F-07 | -818.9546252 | -309.9665023 | -1128.921127 |
| TCGA-ZF-A9R1-01A-11R-A39I-07 | -1266.521024 | -193.0916174 | -1459.612642 |
| TCGA-XF-A9SH-01A-11R-A39I-07 | -938.7449668 | 94.17531933 | -844.5696475 |
| TCGA-C4-A0F7-01A-11R-A084-07 | -1010.934687 | -503.9408277 | -1514.875514 |
| TCGA-C4-A0F0-01A-12R-A10U-07 | -226.5068584 | 824.9287192 | 598.4218607 |
| TCGA-DK-A3WX-01A-22R-A22U-07 | 83.46013378 | 1462.896289 | 1546.356422 |
| TCGA-BT-A2LA-01A-11R-A18C-07 | -1464.130769 | -1513.818931 | -2977.9497 |
| TCGA-4Z-AA87-01A-11R-A39I-07 | -872.3538145 | 670.1355676 | -202.2182469 |
| TCGA-BT-A2LB-01A-11R-A18C-07 | 204.2524299 | 1000.185237 | 1204.437667 |
| TCGA-DK-A2I6-01A-12R-A18C-07 | -1006.766968 | -242.5377144 | -1249.304683 |
| TCGA-BT-A20W-01A-21R-A14Y-07 | -653.8803137 | 576.592141 | -77.28817278 |
| TCGA-FD-A5BX-01A-11R-A26T-07 | 775.9310027 | 1853.73093 | 2629.661933 |
| TCGA-FJ-A3Z7-01A-12R-A23N-07 | 110.5601186 | 322.5521122 | 433.1122307 |
| TCGA-E7-A7PW-01A-11R-A352-07 | -1959.356768 | -179.0879645 | -2138.444732 |
| TCGA-4Z-AA7S-01A-11R-A39I-07 | -1447.857701 | -662.0392345 | -2109.896935 |
| TCGA-CF-A3MH-01A-11R-A20F-07 | -2027.983214 | -153.7949789 | -2181.778193 |
| TCGA-FD-A6TG-01A-11R-A32O-07 | 540.0878306 | 831.4355355 | 1371.523366 |
| TCGA-DK-A6AV-01A-12R-A30C-07 | -1054.822791 | 291.8651778 | -762.9576129 |
| TCGA-DK-A1A3-01A-11R-A13Y-07 | 198.295734 | 647.8116132 | 846.1073472 |
| TCGA-GC-A3RD-01A-12R-A22U-07 | -1229.620182 | -152.1366199 | -1381.756801 |
| TCGA-BT-A20R-01A-12R-A16R-07 | 853.666859 | 565.4212595 | 1419.088119 |
| TCGA-XF-A9T0-01A-11R-A39I-07 | -256.927916 | 613.0123839 | 356.0844679 |
| TCGA-YF-AA3M-01A-11R-A42T-07 | -1580.181137 | -145.5690255 | -1725.750163 |
| TCGA-FD-A62O-01A-11R-A30C-07 | -911.152237 | -472.2078529 | -1383.36009 |
| TCGA-BL-A3JM-01A-12R-A21D-07 | -596.2249657 | -225.9493769 | -822.1743426 |
| TCGA-FJ-A3ZF-01A-11R-A23N-07 | -1923.981545 | -1086.243591 | -3010.225135 |
| TCGA-K4-AAQO-01A-11R-A38B-07 | 438.9785152 | 820.0172589 | 1258.995774 |
| TCGA-XF-AAMJ-01A-11R-A42T-07 | 1228.824521 | 590.482679 | 1819.3072 |
| TCGA-G2-A2EC-01A-11R-A180-07 | 361.7462563 | 953.5753123 | 1315.321569 |
| TCGA-CU-A0YN-01A-21R-A10U-07 | -126.2738067 | 912.9617221 | 786.6879155 |

| | | | |
|---|---|---|---|
| TCGA-FD-A5BZ-01A-11R-A28M-07 | 1044.086987 | 114.9040768 | 1158.991064 |
| TCGA-UY-A9PE-01A-11R-A38B-07 | -570.4405076 | -302.1433496 | -872.5838573 |
| TCGA-BT-A2LD-01A-12R-A20F-07 | -666.3928008 | 182.4357063 | -483.9570945 |
| TCGA-UY-A9PD-01A-11R-A38B-07 | -302.7679125 | -89.78217376 | -392.5500863 |
| TCGA-CU-A5W6-01A-11R-A28M-07 | -1014.502831 | 131.0137774 | -883.4890535 |
| TCGA-CF-A3MF-01A-12R-A21D-07 | -1360.903018 | -318.9058452 | -1679.808863 |
| TCGA-CF-A47T-01A-11R-A23W-07 | -1362.24037 | -583.3195482 | -1945.559918 |
| TCGA-YC-A8S6-01A-31R-A38B-07 | 601.02009 | 1310.698215 | 1911.718305 |
| TCGA-DK-AA6T-01A-11R-A39I-07 | -167.0789988 | 2176.911384 | 2009.832385 |
| TCGA-BT-A20N-01A-11R-A14Y-07 | -1085.321534 | -733.3445574 | -1818.666091 |
| TCGA-C4-A0F6-01A-11R-A10U-07 | -405.7798152 | 69.03659456 | -336.7432207 |
| TCGA-FD-A3B6-01A-21R-A20F-07 | -238.7557348 | 1723.945563 | 1485.189829 |
| TCGA-XF-A9T2-01A-11R-A42T-07 | -192.3304104 | -125.504888 | -317.8352984 |
| TCGA-GC-A3OO-01A-11R-A22U-07 | -50.91724238 | 384.1812845 | 333.2640421 |
| TCGA-FD-A3SN-01A-12R-A22U-07 | -332.1015498 | 277.9910893 | -54.11046043 |
| TCGA-ZF-A9R2-01A-11R-A39I-07 | -2523.807661 | -812.8730472 | -3336.680708 |
| TCGA-E7-A4IJ-01A-31R-A26T-07 | -1415.960934 | 502.3874337 | -913.5735004 |
| TCGA-H4-A2HO-01A-11R-A180-07 | -854.6917222 | 517.1168972 | -337.574825 |
| TCGA-XF-AAN3-01A-11R-A42T-07 | 253.5486609 | 731.8099038 | 985.3585646 |
| TCGA-XF-AAMH-01A-11R-A42T-07 | -941.0822289 | -654.4744663 | -1595.556695 |
| TCGA-XF-A9ST-01A-11R-A42T-07 | -1532.276709 | -943.9025576 | -2476.179267 |
| TCGA-GU-A42R-01A-11R-A23N-07 | -1671.688448 | -712.150787 | -2383.839235 |
| TCGA-R3-A69X-01A-22R-A30C-07 | 475.0582581 | 1277.384239 | 1752.442497 |
| TCGA-FD-A6TF-01A-52R-A32O-07 | 445.7657454 | 405.8761549 | 851.6419003 |
| TCGA-XF-AAMR-01A-31R-A42T-07 | 568.425506 | 559.6323707 | 1128.057877 |
| TCGA-DK-A3IQ-01A-31R-A32Y-07 | 1788.15184 | 359.371394 | 2147.523234 |
| TCGA-E7-A4XJ-01A-11R-A26T-07 | -1435.63348 | -529.1924223 | -1964.825902 |
| TCGA-4Z-AA7M-01A-11R-A39I-07 | -1117.078008 | -201.8104095 | -1318.888418 |
| TCGA-ZF-AA56-01A-31R-A39I-07 | -214.6232854 | 1024.735281 | 810.1119953 |
| TCGA-XF-A8HH-01A-11R-A38B-07 | 208.4458413 | 274.5703945 | 483.0162358 |
| TCGA-C4-A0EZ-01A-21R-A24X-07 | -1369.738353 | -1194.460849 | -2564.199202 |
| TCGA-FD-A62P-01A-32R-A30C-07 | 128.5319136 | 1162.745729 | 1291.277643 |
| TCGA-GU-A42Q-01A-11R-A23W-07 | -1500.810052 | -551.5190562 | -2052.329108 |
| TCGA-GD-A3OQ-01A-32R-A220-07 | -1232.185027 | -127.7196991 | -1359.904726 |
| TCGA-BT-A20U-01A-11R-A14Y-07 | -382.5827482 | 199.1047669 | -183.4779812 |
| TCGA-XF-A9SW-01A-11R-A42T-07 | 1487.841307 | 1133.862423 | 2621.70373 |
| TCGA-ZF-A9RC-01A-11R-A38B-07 | -9.625174738 | -284.4898875 | -294.1150622 |
| TCGA-E7-A6MD-01A-41R-A352-07 | 29.90544266 | 1081.904457 | 1111.809899 |
| TCGA-E5-A4TZ-01A-11R-A31N-07 | -2067.906824 | -317.3408843 | -2385.247709 |
| TCGA-BT-A0YX-01A-11R-A10U-07 | -579.917691 | 955.6813362 | 375.7636453 |
| TCGA-FT-A3EE-01A-11R-A206-07 | -1111.13526 | -284.8973122 | -1396.032573 |
| TCGA-ZF-A9RL-01A-11R-A38B-07 | -2280.334192 | -960.0256762 | -3240.359869 |
| TCGA-ZF-A9R9-01A-11R-A38B-07 | 752.5509131 | 519.0648935 | 1271.615807 |
| TCGA-ZF-A9RE-01A-11R-A38B-07 | -2084.600796 | -382.3380309 | -2466.938827 |
| TCGA-CF-A5U8-01A-11R-A28M-07 | -1834.582029 | -519.9362869 | -2354.518316 |
| TCGA-XF-AAMW-01A-11R-A42T-07 | -479.1744084 | 808.3740612 | 329.1996528 |
| TCGA-GU-A767-01A-11R-A32O-07 | -411.9720135 | -151.5376607 | -563.5096742 |
| TCGA-SY-A9G0-01A-12R-A38B-07 | 1125.940816 | 675.7224522 | 1801.663269 |
| TCGA-GV-A40G-01A-11R-A23N-07 | -1904.050598 | -719.6521703 | -2623.702768 |
| TCGA-ZF-AA4R-01A-11R-A38B-07 | 142.3784664 | -166.9331611 | -24.55469468 |
| TCGA-K4-A3WV-01A-11R-A22U-07 | -1432.971567 | -898.8744651 | -2331.846033 |
| TCGA-G2-A3VY-01A-11R-A22U-07 | -2141.305525 | -1052.268306 | -3193.573831 |
| TCGA-XF-AAN8-01A-11R-A42T-07 | 2173.232609 | 2220.757388 | 4393.989998 |
| TCGA-K4-A3WU-01B-11R-A23N-07 | 159.6359884 | 1563.817383 | 1723.453372 |
| TCGA-FD-A3SJ-01A-12R-A22U-07 | -241.1800793 | -37.37734681 | -278.5574261 |
| TCGA-FD-A3B5-01A-11R-A20F-07 | -827.669585 | -300.3755513 | -1128.045136 |
| TCGA-4Z-AA80-01A-11R-A39I-07 | -1797.747274 | -519.8471313 | -2317.594405 |
| TCGA-G2-A3IE-01A-11R-A20F-07 | -1417.870033 | -173.5491115 | -1591.419145 |

| | | | |
|---|---|---|---|
| TCGA-XF-A8HG-01A-11R-A36F-07 | -1718.539812 | -537.1999557 | -2255.739767 |
| TCGA-K4-A83P-01A-11R-A352-07 | 1135.523307 | 2564.842421 | 3700.365728 |
| TCGA-GD-A2C5-01A-12R-A180-07 | -231.137795 | 1.799243369 | -229.3385517 |
| TCGA-XF-AAMX-01A-11R-A42T-07 | -424.7911888 | -297.7370873 | -722.5282762 |
| TCGA-DK-A1AE-01A-11R-A13Y-07 | -1303.741983 | -411.3217483 | -1715.063731 |
| TCGA-FJ-A3ZE-01A-11R-A23N-07 | -2127.572862 | -1083.612839 | -3211.185701 |
| TCGA-DK-A2I1-01A-11R-A180-07 | 797.1507132 | 1228.090061 | 2025.240774 |
| TCGA-FD-A5BR-01A-11R-A26T-07 | -182.6517508 | 1038.227009 | 855.575258 |
| TCGA-DK-A1AF-01A-11R-A13Y-07 | 1249.508009 | 1761.440866 | 3010.948874 |
| TCGA-LT-A5Z6-01A-11R-A28M-07 | -1882.686355 | 29.5367305 | -1853.149624 |
| TCGA-K4-A4AB-01B-12R-A28M-07 | 142.4038111 | 527.8312289 | 670.23504 |
| TCGA-G2-A2EO-01A-11R-A180-07 | 453.7178217 | 1415.61875 | 1869.336572 |
| TCGA-GV-A3JZ-01A-11R-A21D-07 | -403.8390167 | 78.82385838 | -325.0151583 |
| TCGA-XF-A9SV-01A-21R-A42T-07 | 143.7136967 | -104.6311617 | 39.08253496 |
| TCGA-FD-A6TH-01A-11R-A32O-07 | -6.917396765 | 118.6160187 | 111.6986219 |
| TCGA-DK-AA6W-01A-12R-A39I-07 | -1255.623448 | -112.2454485 | -1367.868896 |
| TCGA-DK-A3X1-01A-12R-A22U-07 | -574.1578472 | 74.44436244 | -499.7134848 |
| TCGA-XF-AAMG-01A-11R-A42T-07 | -197.7613322 | -66.20829109 | -263.9696233 |
| TCGA-UY-A78N-01A-12R-A33J-07 | -1236.422921 | -942.8949187 | -2179.31784 |
| TCGA-DK-AA75-01A-11R-A39I-07 | -1972.59542 | -796.1124949 | -2768.707915 |
| TCGA-ZF-AA5P-01A-11R-A39I-07 | 729.3630128 | 922.5889047 | 1651.951917 |
| TCGA-FD-A3SL-01A-21R-A22U-07 | 1147.831214 | 726.9441881 | 1874.775402 |
| TCGA-XF-AAN7-01A-11R-A42T-07 | -171.193373 | 10.51867845 | -160.6746946 |
| TCGA-DK-A3IM-01A-11R-A20F-07 | -1830.555732 | -695.9575307 | -2526.513262 |
| TCGA-FD-A3SS-01A-12R-A22U-07 | -1028.806074 | -464.6981206 | -1493.504195 |
| TCGA-4Z-AA7N-01A-11R-A39I-07 | 827.6745637 | 2635.708844 | 3463.383407 |
| TCGA-HQ-A2OE-01A-11R-A206-07 | -1835.818542 | -440.5767062 | -2276.395248 |
| TCGA-UY-A8OC-01A-11R-A36F-07 | 343.0170832 | 302.6127004 | 645.6297836 |
| TCGA-GV-A3JV-01A-11R-A220-07 | -217.5467589 | 602.62852 | 385.0817611 |
| TCGA-KQ-A41S-01A-12R-A33J-07 | 333.8836771 | 262.8166139 | 596.700291 |
| TCGA-DK-A2HX-01A-12R-A18C-07 | 991.3564904 | 253.722966 | 1245.079456 |
| TCGA-CF-A8HY-01A-11R-A36F-07 | -1698.927477 | -142.2891317 | -1841.216609 |
| TCGA-BT-A0S7-01A-11R-A10U-07 | -526.6595009 | -122.6401389 | -649.2996398 |
| TCGA-GU-A42P-01A-11R-A23W-07 | -1997.738307 | -1036.828308 | -3034.566615 |
| TCGA-FD-A3NA-01A-11R-A21D-07 | -371.7782667 | 672.1949436 | 300.4166769 |
| TCGA-XF-A9SL-01A-11R-A39I-07 | 1104.762651 | 2056.881516 | 3161.644167 |
| TCGA-ZF-AA54-01A-11R-A39I-07 | 852.3590577 | 1768.691196 | 2621.050254 |
| TCGA-ZF-AA4N-01A-11R-A38B-07 | -825.1804995 | 777.6069411 | -47.57355842 |
| TCGA-BT-A20X-01A-11R-A16R-07 | -263.7677593 | 419.6619824 | 155.8942231 |
| TCGA-GV-A3JW-01A-11R-A20F-07 | -1625.82104 | -1052.891038 | -2678.712078 |
| TCGA-FD-A6TI-01A-11R-A32O-07 | -757.3087017 | -73.43860388 | -830.7473056 |
| TCGA-FD-A3SP-01A-31R-A22U-07 | 1721.496654 | 1948.137055 | 3669.633709 |
| TCGA-E7-A97Q-01A-11R-A38B-07 | -191.4426117 | 267.3977259 | 75.95511422 |
| TCGA-GV-A3QG-01A-11R-A220-07 | 1355.165006 | 2745.437968 | 4100.602975 |
| TCGA-ZF-AA52-01A-12R-A39I-07 | 824.4039566 | 1057.351027 | 1881.754984 |
| TCGA-CF-A9FL-01A-11R-A38B-07 | -406.2534876 | -26.95832667 | -433.2118142 |
| TCGA-G2-AA3D-01A-11R-A39I-07 | -2172.978646 | -396.9958516 | -2569.974497 |
| TCGA-DK-A2I2-01A-11R-A180-07 | 224.6640859 | 1605.855233 | 1830.519319 |
| TCGA-FD-A62S-01A-11R-A30C-07 | 1251.034062 | 1439.004175 | 2690.038238 |
| TCGA-MV-A51V-01A-11R-A26T-07 | -1296.676001 | -614.729522 | -1911.405523 |
| TCGA-DK-A3X2-01A-11R-A22U-07 | -1883.308754 | -1012.314809 | -2895.623562 |
| TCGA-DK-A1AD-01A-11R-A13Y-07 | -377.5006652 | 270.698277 | -106.8023881 |
| TCGA-K4-A5RI-01A-11R-A28M-07 | -624.4263477 | 218.7532106 | -405.6731372 |
| TCGA-GV-A3QF-01A-31R-A22U-07 | -1773.780482 | -487.8241834 | -2261.604665 |
| TCGA-E7-A7DV-01A-11R-A33J-07 | 568.209344 | 2127.30066 | 2695.510004 |
| TCGA-HQ-A2OF-01A-11R-A26T-07 | -1767.350566 | -973.7110747 | -2741.061641 |
| TCGA-DK-AA6P-01A-11R-A39I-07 | -1680.012178 | -850.9457257 | -2530.957903 |
| TCGA-CF-A47Y-01A-11R-A23W-07 | -1708.484958 | -439.0111195 | -2147.496077 |

| | | | |
|---|---|---|---|
| TCGA-G2-A2EL-01A-12R-A18C-07 | -2124.970789 | -1658.358265 | -3783.329054 |
| TCGA-K4-A6MB-01A-11R-A31N-07 | -457.1787123 | -245.4388237 | -702.617536 |
| TCGA-CU-A3KJ-01A-11R-A21D-07 | -1059.523645 | 400.9905439 | -658.5331013 |
| TCGA-E7-A85H-01A-11R-A352-07 | -588.2514974 | 572.298086 | -15.95341146 |
| TCGA-G2-AA3C-01A-21R-A39I-07 | -37.48969662 | 2038.93435 | 2001.444653 |
| TCGA-DK-A3IN-01A-11R-A20F-07 | 465.616211 | 1027.236029 | 1492.85224 |
| TCGA-FJ-A3Z9-01A-11R-A26T-07 | -2017.966953 | -786.2358303 | -2804.202783 |
| TCGA-DK-A1AC-01A-11R-A13Y-07 | -22.76497704 | 943.8747838 | 921.1098068 |
| TCGA-C4-A0F1-01A-11R-A034-07 | -412.7782617 | 275.0028923 | -137.7753693 |
| TCGA-E7-A5KE-01A-11R-A28M-07 | -2261.04139 | -1028.366253 | -3289.407643 |
| TCGA-ZF-A9RM-01A-11R-A38B-07 | -2588.148024 | -922.9273562 | -3511.07538 |
| TCGA-CU-A72E-01A-12R-A33J-07 | -186.6435857 | 266.4353444 | 79.79175873 |
| TCGA-E7-A3Y1-01A-11R-A22U-07 | -1339.211405 | -158.9554072 | -1498.166812 |
| TCGA-FD-A5BV-01A-11R-A26T-07 | -1126.249514 | -514.0468952 | -1640.296409 |
| TCGA-UY-A78M-01A-21R-A352-07 | -344.521255 | -288.7816628 | -633.3029178 |
| TCGA-4Z-AA7R-01A-11R-A39I-07 | -1004.307293 | -722.8728613 | -1727.180154 |
| TCGA-FD-A3SM-01A-11R-A22U-07 | 136.8667851 | -63.02683872 | 73.83994641 |
| TCGA-BL-A5ZZ-01A-31R-A30C-07 | 959.5110654 | 1240.12303 | 2199.634095 |
| TCGA-CF-A1HS-01A-11R-A13Y-07 | -687.9632531 | 599.1528819 | -88.81037127 |
| TCGA-XF-AAMT-01A-11R-A42T-07 | 861.5669208 | 1019.31705 | 1880.883971 |
| TCGA-XF-A9SU-01A-31R-A39I-07 | 424.4102853 | 935.8789488 | 1360.289234 |
| TCGA-UY-A78L-01A-12R-A33J-07 | -283.35069 | 184.7672186 | -98.5834714 |
| TCGA-S5-A6DX-01A-11R-A31N-07 | 306.3621256 | 1893.493591 | 2199.855717 |
| TCGA-XF-A8HI-01A-11R-A38B-07 | -1606.874392 | -23.16932086 | -1630.043713 |
| TCGA-G2-A2EK-01A-22R-A18C-07 | -960.0274924 | 307.2632962 | -652.7641962 |
| TCGA-H4-A2HQ-01A-11R-A180-07 | -1815.266986 | -390.6468364 | -2205.913823 |
| TCGA-XF-A9SP-01A-11R-A39I-07 | 935.2435017 | 823.3006988 | 1758.544201 |
| TCGA-DK-A3IK-01A-32R-A21D-07 | -437.8304164 | -57.91427346 | -495.7446898 |
| TCGA-BL-A13J-01A-11R-A277-07 | 313.7952719 | -98.3449596 | 215.4503123 |
| TCGA-BL-A0C8-01A-11R-A277-07 | -523.2953681 | -161.1414728 | -684.4368409 |
| TCGA-BL-A13I-01A-11R-A277-07 | 801.5887458 | 2090.466539 | 2892.055285 |
| TCGA-CF-A47X-01A-31R-A23W-07 | -1848.82024 | -663.0271473 | -2511.847387 |
| TCGA-2F-A9KP-01A-11R-A38B-07 | -1709.896998 | -741.1543763 | -2451.051374 |
| TCGA-DK-A3IT-01A-31R-A20F-07 | -81.53021808 | 495.4069134 | 413.8766953 |
| TCGA-FD-A43P-01A-31R-A23W-07 | -317.5126138 | 1974.137375 | 1656.624761 |
| TCGA-DK-A3IV-01A-22R-A21D-07 | -1572.831352 | 1264.139589 | -308.6917635 |
| TCGA-GU-A762-01A-11R-A33J-07 | 402.0133415 | 2424.909083 | 2826.922424 |
| TCGA-GC-A3I6-01A-11R-A20F-07 | -537.0423196 | 845.8469502 | 308.8046306 |
| TCGA-FD-A6TA-01A-12R-A33J-07 | 523.898597 | 1256.301763 | 1780.20036 |
| TCGA-FD-A3SR-01A-11R-A22U-07 | 341.0635199 | 981.2454676 | 1322.308988 |
| TCGA-GU-AATP-01A-11R-A39I-07 | 511.8350025 | 729.421091 | 1241.256094 |
| TCGA-DK-AA6X-01A-12R-A42T-07 | -609.4335956 | 860.3999819 | 250.9663863 |
| TCGA-XF-A9T3-01A-11R-A42T-07 | 492.7313344 | 1010.732116 | 1503.463451 |
| TCGA-DK-A3WW-01A-22R-A23N-07 | -503.2795337 | 1984.798444 | 1481.51891 |
| TCGA-K4-A3WS-01A-11R-A22U-07 | 1151.375337 | 1231.980508 | 2383.355845 |
| TCGA-UY-A9PA-01A-11R-A38B-07 | -1715.101731 | 809.5126444 | -905.5890863 |
| TCGA-ZF-A9RN-01A-11R-A42T-07 | -1208.53587 | 1068.752541 | -139.7833294 |
| TCGA-XF-AAMQ-01A-11R-A42T-07 | -743.6207282 | 1138.105582 | 394.4848539 |
| TCGA-CF-A1HR-01A-11R-A13Y-07 | -861.159498 | 358.8656037 | -502.2938943 |
| TCGA-E7-A3X6-01A-12R-A22U-07 | -676.5265375 | 1082.542682 | 406.0161441 |
| TCGA-GC-A3BM-01A-11R-A22U-07 | -1575.717898 | -69.24486766 | -1644.962766 |
| TCGA-GV-A6ZA-01A-12R-A33J-07 | -1425.267806 | 362.6185483 | -1062.649258 |
| TCGA-G2-A2EJ-01A-11R-A180-07 | -1373.677426 | 382.4072563 | -991.2701697 |
| TCGA-BT-A20Q-01A-11R-A14Y-07 | 411.1787277 | 1045.015366 | 1456.194094 |
| TCGA-SY-A9G5-01A-11R-A38B-07 | 770.7190819 | 1712.124038 | 2482.84312 |
| TCGA-4Z-AA7Q-01A-11R-A39I-07 | -563.8149181 | 1814.351433 | 1250.536515 |
| TCGA-FD-A5BT-01A-11R-A26T-07 | 1476.900148 | 2406.39686 | 3883.297008 |
| TCGA-E7-A6MF-01A-12R-A32O-07 | -1915.665027 | -335.4330596 | -2251.098087 |

| | | | |
|---|---|---|---|
| TCGA-FT-A61P-01A-11R-A30C-07 | 644.0366851 | 1189.782331 | 1833.819016 |
| TCGA-DK-AA77-01A-11R-A39I-07 | -1499.781866 | 474.0717997 | -1025.710066 |
| TCGA-BT-A20O-01A-21R-A14Y-07 | 1019.523722 | 3151.039523 | 4170.563245 |
| TCGA-XF-A9SZ-01A-11R-A39I-07 | 618.9346292 | 820.4232616 | 1439.357891 |
| TCGA-E7-A678-01A-11R-A30C-07 | -1978.549287 | -36.27803697 | -2014.827324 |
| TCGA-FD-A5BU-01A-31R-A26T-07 | 468.3143181 | 1326.942104 | 1795.256423 |
| TCGA-FD-A3N5-01A-11R-A21D-07 | -969.2172299 | 328.8625387 | -640.3546912 |
| TCGA-ZF-AA5H-01A-11R-A39I-07 | -231.0234485 | 1297.840865 | 1066.817417 |
| TCGA-YF-AA3L-01A-11R-A38B-07 | -1372.501922 | -170.2256283 | -1542.727551 |
| TCGA-XF-A8HB-01A-11R-A36F-07 | -2002.990837 | -439.7866781 | -2442.777515 |
| TCGA-ZF-AA4X-01A-11R-A38B-07 | -2533.601785 | -415.0545048 | -2948.656289 |
| TCGA-BT-A20P-01A-11R-A14Y-07 | -1572.721914 | -435.4062115 | -2008.128125 |
| TCGA-CF-A8HX-01A-11R-A36F-07 | -1670.945487 | -533.7359621 | -2204.681449 |
| TCGA-PQ-A6FI-01A-11R-A31N-07 | -1267.741531 | 437.6005744 | -830.1409566 |
| TCGA-DK-A6B2-01A-11R-A30C-07 | 609.0382062 | 1286.128871 | 1895.167077 |
| TCGA-4Z-AA86-01A-11R-A39I-07 | 638.1567973 | 1888.156489 | 2526.313286 |
| TCGA-5N-A9KI-01A-31R-A42T-07 | -48.34767329 | 719.9197549 | 671.5720816 |
| TCGA-GU-AATO-01A-11R-A39I-07 | -142.5775717 | 1144.076157 | 1001.498585 |
| TCGA-CF-A9FH-01A-11R-A38B-07 | -2008.774173 | -301.9735391 | -2310.747712 |
| TCGA-4Z-AA7O-01A-31R-A39I-07 | -668.3140391 | 624.4774728 | -43.83656624 |
| TCGA-GV-A3QI-01A-11R-A220-07 | -1923.958005 | -1225.787417 | -3149.745422 |
| TCGA-GD-A6C6-01A-21R-A31N-07 | -1109.785263 | 121.3666558 | -988.4186073 |
| TCGA-4Z-AA7Y-01A-11R-A39I-07 | -1795.928912 | -528.5197698 | -2324.448681 |
| TCGA-CU-A0YO-01A-11R-A10U-07 | -370.7622481 | 858.9446806 | 488.1824325 |
| TCGA-CF-A5UA-01A-11R-A28M-07 | -1631.416187 | -274.0619484 | -1905.478136 |
| TCGA-GU-A763-01A-11R-A32O-07 | -2043.548404 | -94.22525745 | -2137.773661 |
| TCGA-FD-A3B7-01A-31R-A20F-07 | 1307.473753 | 2178.621502 | 3486.095255 |
| TCGA-FD-A5C0-01A-11R-A28M-07 | -478.6449067 | 169.6606306 | -308.9842761 |
| TCGA-FD-A5BS-01A-21R-A26T-07 | 1676.875644 | 3222.838941 | 4899.714585 |
| TCGA-GV-A40E-01A-12R-A23N-07 | -649.5693633 | 378.9344242 | -270.6349391 |
| TCGA-G2-AA3B-01A-11R-A39I-07 | -1802.724911 | 265.3575045 | -1537.367407 |
| TCGA-K4-A54R-01A-11R-A26T-07 | 229.7982701 | 1383.333752 | 1613.132022 |
| TCGA-DK-A1AA-01A-11R-A13Y-07 | -1496.805699 | 26.4058372 | -1470.399862 |
| TCGA-DK-AA6R-01A-11R-A42T-07 | -955.068776 | 162.063047 | -793.005729 |
| TCGA-BT-A3PH-01A-11R-A220-07 | -1053.160723 | -435.3754732 | -1488.536196 |
| TCGA-DK-A3IS-01A-21R-A21D-07 | -1903.05477 | -224.8047015 | -2127.859471 |
| TCGA-XF-A9SJ-01A-11R-A39I-07 | 167.0876112 | 1410.224228 | 1577.311839 |
| TCGA-CF-A7I0-01A-22R-A352-07 | -505.7188032 | 53.7238252 | -451.994978 |
| TCGA-GC-A3YS-01A-11R-A23N-07 | 457.5273144 | 925.7018167 | 1383.229131 |
| TCGA-UY-A78O-01A-12R-A33J-07 | -940.4286026 | -76.3763374 | -1016.80494 |
| TCGA-E7-A6ME-01A-22R-A32O-07 | -1676.948414 | 170.375103 | -1506.573311 |
| TCGA-XF-AAN5-01A-11R-A42T-07 | -633.4749981 | 1476.43372 | 842.9587216 |
| TCGA-ZF-AA4T-01A-11R-A38B-07 | -1841.793029 | -429.0312969 | -2270.824326 |
| TCGA-FD-A6TE-01A-12R-A33J-07 | -1997.023821 | -138.1366701 | -2135.160492 |
| TCGA-FD-A6TB-01A-12R-A33J-07 | 463.5365261 | 2001.234907 | 2464.771433 |
| TCGA-FD-A3B8-01A-31R-A20F-07 | 1684.673792 | 2749.287768 | 4433.961561 |
| TCGA-XF-A9SY-01A-21R-A42T-07 | 574.6123891 | 2118.849622 | 2693.462011 |
| TCGA-CF-A9FF-01A-11R-A38B-07 | -671.0588926 | 427.4448425 | -243.61405 |
| TCGA-DK-A6B5-01A-11R-A31N-07 | -997.0761807 | 761.5287405 | -235.5474402 |
| TCGA-GC-A3RC-01A-11R-A22U-07 | -453.8394384 | 1424.27857 | 970.4391312 |
| TCGA-GV-A3QK-01B-11R-A23N-07 | -1312.556734 | 886.6372722 | -425.919462 |
| TCGA-GU-A764-01A-11R-A352-07 | 258.2604641 | 1446.796261 | 1705.056725 |
| TCGA-DK-A6B6-01A-11R-A30C-07 | -1527.169061 | 765.2468446 | -761.9222167 |
| TCGA-5N-A9KM-01A-11R-A42T-07 | -114.2816029 | 865.7125958 | 751.4309929 |
| TCGA-XF-A8HF-01A-11R-A36F-07 | -220.5700229 | 450.473264 | 229.9032411 |
| TCGA-ZF-AA4U-01A-11R-A38B-07 | -2331.30795 | -325.5973252 | -2656.905276 |
| TCGA-XF-A9T6-01A-11R-A42T-07 | -1275.929362 | 274.9708245 | -1000.958538 |
| TCGA-CF-A47V-01A-11R-A23W-07 | -885.9062731 | 359.3802344 | -526.5260387 |

| | | | |
|---|---|---|---|
| TCGA-XF-A9SI-01A-11R-A39I-07 | 701.3164857 | 2861.25513 | 3562.571615 |
| TCGA-BT-A42F-01A-11R-A23W-07 | -263.0806128 | 2080.801078 | 1817.720466 |
| TCGA-ZF-A9RF-01A-11R-A38B-07 | -820.6767404 | 1900.256751 | 1079.58001 |
| TCGA-KQ-A41N-01A-11R-A33J-07 | -1310.12826 | -798.2579723 | -2108.386232 |
| TCGA-FD-A3B3-01A-12R-A206-07 | 728.4621731 | 2018.206635 | 2746.668808 |
| TCGA-ZF-A9R4-01A-11R-A38B-07 | -1667.657169 | 144.2200828 | -1523.437086 |
| TCGA-GV-A3QH-01A-11R-A220-07 | -2315.126991 | -796.1678231 | -3111.294814 |
| TCGA-DK-A6B1-01A-12R-A30C-07 | -1285.21522 | -450.0220625 | -1735.237282 |
| TCGA-BT-A3PJ-01A-21R-A220-07 | -690.013622 | 1040.838577 | 350.8249553 |
| TCGA-ZF-A9R7-01A-11R-A38B-07 | -1030.289194 | 2039.911953 | 1009.622759 |
| TCGA-FD-A3SO-01A-11R-A22U-07 | 376.3905814 | 1008.022168 | 1384.41275 |
| TCGA-E7-A97P-01A-11R-A38B-07 | 85.41712198 | 1758.170782 | 1843.587904 |
| TCGA-4Z-AA81-01A-11R-A39I-07 | -1339.501233 | 794.196638 | -545.3045952 |
| TCGA-2F-A9KQ-01A-11R-A38B-07 | -1267.917662 | -622.5833127 | -1890.500975 |
| TCGA-FD-A43Y-01A-21R-A26T-07 | 25.24793983 | 811.3713864 | 836.6193262 |
| TCGA-S5-AA26-01A-11R-A38B-07 | -1965.669533 | -651.4471161 | -2617.116649 |
| TCGA-BT-A42E-01A-11R-A23W-07 | -553.8595471 | 1714.42268 | 1160.563133 |
| TCGA-FD-A43X-01A-11R-A23W-07 | -1872.637832 | -561.1370293 | -2433.774861 |
| TCGA-BT-A20T-01A-11R-A14Y-07 | -61.98117668 | 758.9264427 | 696.945266 |
| TCGA-E7-A7DU-01A-11R-A32O-07 | -1609.985081 | -233.9700033 | -1843.955085 |
| TCGA-DK-A3WY-01A-11R-A22U-07 | 861.6143521 | 3106.438631 | 3968.052983 |
| TCGA-G2-A2EF-01A-12R-A18C-07 | -728.9693036 | 1674.273404 | 945.3041006 |
| TCGA-BT-A20V-01A-11R-A14Y-07 | -1678.36541 | 461.7712266 | -1216.594184 |
| TCGA-GU-AATQ-01A-11R-A39I-07 | -1143.554395 | 130.1087361 | -1013.445659 |
| TCGA-CU-A3QU-01A-11R-A22U-07 | -2122.563116 | -443.7451121 | -2566.308229 |
| TCGA-GD-A3OP-01A-21R-A220-07 | -4.885339164 | 306.2879674 | 301.4026282 |
| TCGA-KQ-A41P-01A-12R-A33J-07 | -113.0210206 | 511.7070521 | 398.6860315 |
| TCGA-UY-A9PH-01A-11R-A38B-07 | 195.566241 | 2037.051923 | 2232.618164 |
| TCGA-FD-A5BY-01A-31R-A28M-07 | -221.1067779 | 422.4666339 | 201.3598559 |
| TCGA-GC-A6I3-01A-11R-A31N-07 | -266.297353 | 1655.925947 | 1389.628594 |
| TCGA-ZF-A9R0-01A-11R-A38B-07 | -653.2427849 | 352.8323174 | -300.4104675 |
| TCGA-FD-A43S-01A-21R-A23W-07 | 789.0673245 | 2299.092267 | 3088.159592 |
| TCGA-DK-A6B0-01A-11R-A31N-07 | -1823.490888 | -271.5698067 | -2095.060694 |
| TCGA-KQ-A41Q-01A-11R-A33J-07 | -1488.340056 | -466.7211157 | -1955.061172 |
| TCGA-GC-A4ZW-01A-11R-A26T-07 | -1748.249737 | -605.5915716 | -2353.841309 |
| TCGA-DK-AA74-01A-11R-A39I-07 | 1437.047766 | 2409.08864 | 3846.136406 |
| TCGA-DK-A1AG-01A-11R-A13Y-07 | -1836.599059 | 150.1596338 | -1686.439426 |
| TCGA-UY-A78K-01A-11R-A33J-07 | -581.1620123 | 1809.74824 | 1228.586227 |
| TCGA-CF-A3MG-01A-11R-A20F-07 | -2232.594209 | -659.3294039 | -2891.923613 |
| TCGA-DK-A1A6-01A-11R-A13Y-07 | -511.8082838 | 952.039678 | 440.2313942 |
| TCGA-E7-A541-01A-11R-A26T-07 | -879.8186074 | 1257.309063 | 377.4904555 |
| TCGA-XF-A8HE-01A-11R-A36F-07 | -299.5006307 | 1338.901558 | 1039.400927 |
| TCGA-DK-AA6Q-01A-11R-A39I-07 | -1597.07376 | 1100.98942 | -496.0843402 |
| TCGA-GC-A3WC-01A-31R-A22U-07 | -226.1007212 | 1770.137538 | 1544.036817 |
| TCGA-K4-A4AC-01A-21R-A26T-07 | -581.9779849 | 970.3238015 | 388.3458166 |
| TCGA-K4-A5RH-01A-11R-A30C-07 | 909.439295 | 2532.638723 | 3442.078018 |
| TCGA-FD-A5C1-01A-11R-A28M-07 | 669.1189458 | 1824.191966 | 2493.310912 |
| TCGA-2F-A9KO-01A-11R-A38B-07 | -161.8736535 | 1493.746541 | 1331.872887 |
| TCGA-XF-A9T8-01A-11R-A39I-07 | 332.4904965 | 2014.121867 | 2346.612363 |
| TCGA-CU-A3YL-01A-11R-A22U-07 | -1022.911338 | -156.5561219 | -1179.46746 |
| TCGA-E7-A7XN-01A-11R-A352-07 | -973.6604474 | 1940.965514 | 967.3050663 |
| TCGA-4Z-AA7W-01A-11R-A39I-07 | -120.2389766 | 2503.076128 | 2382.837152 |
| TCGA-FD-A43U-01A-11R-A23W-07 | 1011.250719 | 2322.557889 | 3333.808608 |
| TCGA-ZF-A9R3-01A-11R-A38B-07 | -1250.781485 | 213.0911189 | -1037.690366 |
| TCGA-4Z-AA89-01A-11R-A39I-07 | -2259.354207 | -109.1984497 | -2368.552657 |
| TCGA-XF-AAN2-01A-11R-A42T-07 | -975.3213791 | 930.8740714 | -44.44730766 |
| TCGA-GU-A766-01A-11R-A32O-07 | 195.0621659 | 2466.897305 | 2661.95947 |
| TCGA-FD-A6TD-01A-51R-A33J-07 | 248.8665104 | 1641.192188 | 1890.058699 |

| | | | |
|---|---|---|---|
| TCGA-FD-A3B4-01A-12R-A206-07 | 133.3733211 | 1361.332141 | 1494.705462 |
| TCGA-DK-A1AB-01A-11R-A13Y-07 | 916.5544277 | 983.9636554 | 1900.518083 |
| TCGA-UY-A9PB-01A-11R-A38B-07 | 742.0603299 | 1956.712288 | 2698.772618 |
| TCGA-XF-AAML-01A-11R-A42T-07 | -2047.822511 | 202.3436944 | -1845.478817 |
| TCGA-ZF-AA58-01A-12R-A42T-07 | 805.6731736 | 2438.800865 | 3244.474039 |
| TCGA-YC-A9TC-01A-22R-A39I-07 | -993.9997731 | -219.0162723 | -1213.016045 |
| TCGA-K4-A6FZ-01A-11R-A31N-07 | -986.3385852 | 906.9215332 | -79.41705203 |
| TCGA-KQ-A41R-01A-21R-A352-07 | -1385.798899 | -409.5779862 | -1795.376885 |
| TCGA-DK-AA6M-01A-11R-A39I-07 | -61.36532586 | 1635.813719 | 1574.448393 |
| TCGA-K4-A5RJ-01A-11R-A28M-07 | 430.9058846 | 2851.186287 | 3282.092172 |
| TCGA-HQ-A5ND-01A-11R-A26T-07 | -1768.411829 | -161.6735963 | -1930.085425 |
| TCGA-BT-A3PK-01A-21R-A220-07 | 292.6698977 | 996.6827945 | 1289.352692 |
| TCGA-FD-A3N6-01A-11R-A21D-07 | -1115.554824 | 282.5503169 | -833.0045073 |
| TCGA-UY-A78P-01A-12R-A36F-07 | 145.8544856 | 2022.382299 | 2168.236785 |
| TCGA-CF-A9FM-01A-11R-A38B-07 | -1596.205833 | 186.8179386 | -1409.387894 |
| TCGA-ZF-A9RD-01A-11R-A42T-07 | -252.8536021 | 1869.219779 | 1616.366177 |
| TCGA-E5-A2PC-01A-11R-A206-07 | -1609.003846 | 428.8184961 | -1180.18535 |
| TCGA-GD-A76B-01A-11R-A32O-07 | -790.3707292 | 1389.137916 | 598.7671868 |
| TCGA-GC-A6I1-01A-12R-A31N-07 | -226.3933988 | 2385.615733 | 2159.222334 |
| TCGA-XF-A9SK-01A-11R-A42T-07 | 753.839608 | 1232.181322 | 1986.02093 |
| TCGA-FD-A3SQ-01A-21R-A22U-07 | 826.3206792 | 874.748291 | 1701.06897 |
| TCGA-XF-AAN1-01A-31R-A42T-07 | -1533.309465 | -179.8087465 | -1713.118212 |
| TCGA-CF-A3MI-01A-11R-A20F-07 | -1083.354352 | 305.0378533 | -778.316499 |
| TCGA-XF-AAMZ-01A-11R-A42T-07 | -1040.171478 | 277.0433006 | -763.1281777 |
| TCGA-DK-AA6L-01A-11R-A39I-07 | -996.4174984 | 806.9454933 | -189.4720052 |
| TCGA-XF-A9T4-01A-11R-A39I-07 | -766.355213 | 633.6415239 | -132.7136891 |
| TCGA-UY-A8OB-01A-12R-A42T-07 | -1353.194513 | 559.3032572 | -793.8912562 |
| TCGA-ZF-A9R5-01A-12R-A42T-07 | -1815.834321 | -129.4551807 | -1945.289502 |
| TCGA-DK-AA6S-01A-21R-A39I-07 | 782.7296813 | 1143.21047 | 1925.940151 |
| TCGA-E7-A8O8-01A-11R-A36F-07 | -1496.13599 | -257.3871474 | -1753.523137 |
| TCGA-XF-A9SM-01A-11R-A42T-07 | 634.4185294 | 2503.287899 | 3137.706429 |
| TCGA-XF-A8HC-01A-11R-A36F-07 | -2107.396829 | -1006.849264 | -3114.246093 |
| TCGA-ZF-AA4W-01A-12R-A38B-07 | -1370.094795 | -28.20276328 | -1398.297558 |
| TCGA-LC-A66R-01A-41R-A30C-07 | -318.2627684 | 1546.1339 | 1227.871132 |
| TCGA-E7-A8O7-01A-11R-A36F-07 | -1388.360374 | 619.6363924 | -768.7239817 |
| TCGA-LT-A8JT-01A-11R-A36F-07 | -1556.331424 | 130.824802 | -1425.506622 |
| TCGA-G2-A2ES-01A-11R-A180-07 | -68.42878294 | 858.0730644 | 789.6442814 |
| TCGA-XF-AAN4-01A-11R-A42T-07 | 1656.09277 | 2083.460102 | 3739.552872 |
| TCGA-4Z-AA83-01A-11R-A39I-07 | -892.8526444 | 433.9900057 | -458.8626387 |
| TCGA-ZF-AA4V-01A-11R-A38B-07 | -501.210461 | 835.9013809 | 334.6909199 |
| TCGA-ZF-AA53-01A-11R-A39I-07 | 627.4177166 | 1716.30379 | 2343.721506 |
| TCGA-2F-A9KR-01A-11R-A38B-07 | -1517.31536 | -265.5124642 | -1782.827825 |
| TCGA-E5-A4U1-01A-11R-A31N-07 | -2138.538263 | -1004.774796 | -3143.313059 |
| TCGA-FD-A6TC-01A-21R-A33J-07 | 275.8638956 | 92.00264738 | 367.866543 |
| TCGA-DK-A2I4-01A-11R-A21D-07 | 1185.889107 | 2810.607313 | 3996.49642 |
| TCGA-E7-A519-01A-11R-A26T-07 | -862.9036785 | 1295.520672 | 432.616994 |
| TCGA-DK-A3IU-01A-11R-A20F-07 | 907.4536714 | 2780.344056 | 3687.797727 |
| TCGA-FD-A62N-01A-11R-A30C-07 | 1349.404838 | 3059.573389 | 4408.978227 |
| TCGA-BT-A20J-01A-11R-A14Y-07 | -349.6230465 | 1808.946079 | 1459.323032 |
| TCGA-DK-AA71-01A-31R-A39I-07 | -1331.309229 | -277.1844743 | -1608.493703 |
| TCGA-KQ-A41O-01A-12R-A352-07 | -2613.724799 | -1515.696098 | -4129.420897 |
| TCGA-E7-A677-01A-11R-A30C-07 | -1148.375527 | 130.352718 | -1018.022809 |
| TCGA-DK-A1A5-01A-11R-A13Y-07 | 318.5220176 | 370.5145254 | 689.0365431 |
| TCGA-HQ-A5NE-01A-12R-A28M-07 | -476.961112 | 355.1763586 | -121.7847534 |
| TCGA-CF-A47S-01A-11R-A23W-07 | -1566.93325 | 225.0535836 | -1341.879666 |
| TCGA-FD-A6TK-01A-42R-A33J-07 | 899.7378973 | 1612.090718 | 2511.828615 |
| TCGA-E7-A5KF-01A-11R-A28M-07 | -2486.722808 | -588.0182852 | -3074.741093 |
| TCGA-XF-A9T5-01A-11R-A42T-07 | 63.12894021 | 2157.275735 | 2220.404675 |

| | | | |
|---|---|---|---|
| TCGA-XF-A9SX-01A-21R-A39I-07 | 957.0337531 | 1915.062531 | 2872.096284 |
| TCGA-XF-A8HD-01A-11R-A36F-07 | -290.1865231 | 1430.546284 | 1140.359761 |
| TCGA-GV-A3JX-01A-11R-A20F-07 | -1347.360869 | 611.0888419 | -736.2720272 |

# Figure S1

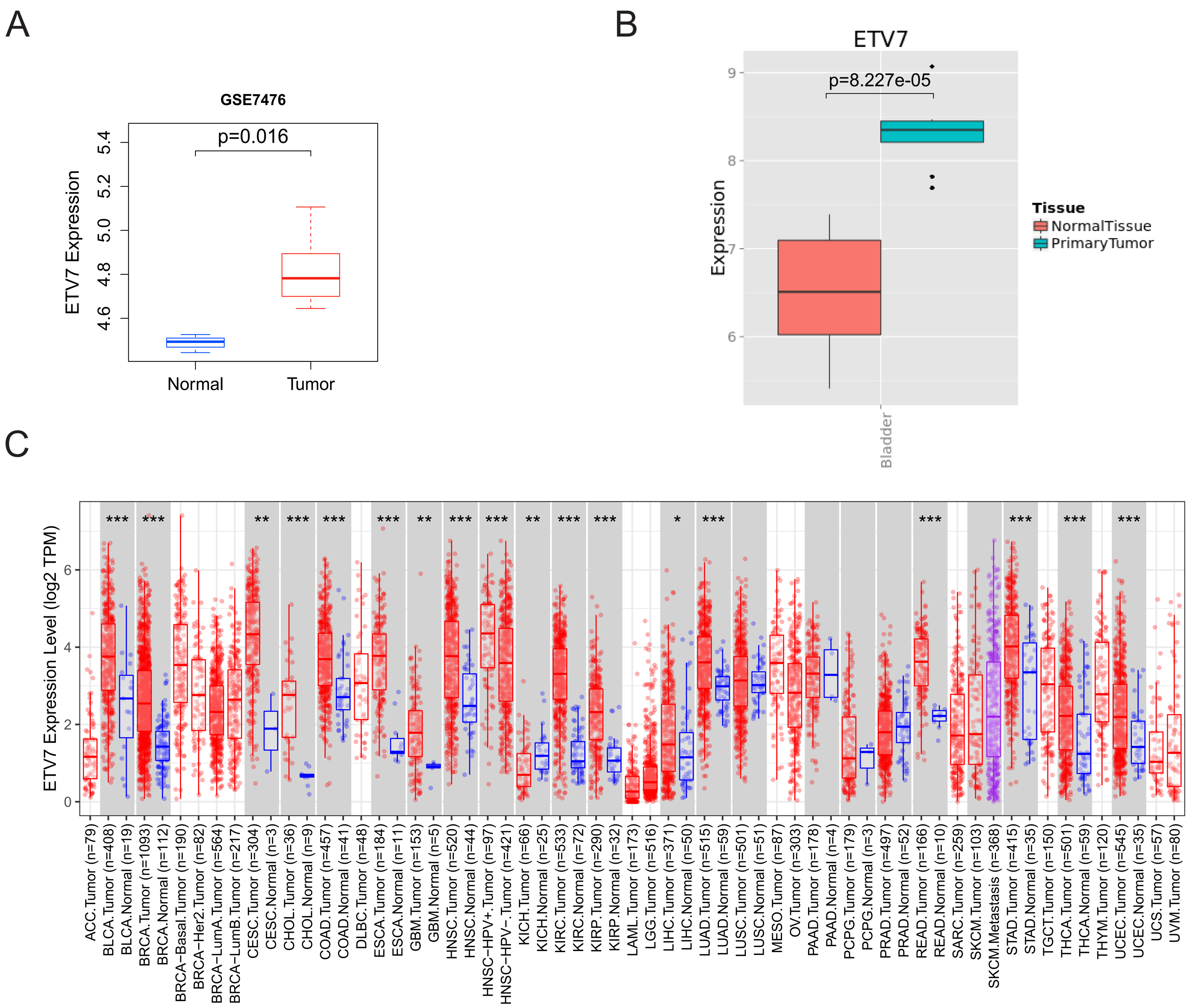

A
GSE7476
p=0.016
ETV7 Expression
Normal
Tumor
B
ETV7
p=8.227e-05
Expression
Tissue
NormalTissue
PrimaryTumor
Bladder
C
ETV7 Expression Level (log2 TPM)
ACC.Tumor (n=79)
BLCA.Tumor (n=408)
BLCA.Normal (n=19)
BRCA.Tumor (n=1093)
BRCA.Normal (n=112)
BRCA−Basal.Tumor (n=190)
BRCA−Her2.Tumor (n=82)
BRCA−LumA.Tumor (n=564)
BRCA−LumB.Tumor (n=217)
CESC.Tumor (n=304)
CESC.Normal (n=3)
CHOL.Tumor (n=36)
CHOL.Normal (n=9)
COAD.Tumor (n=457)
COAD.Normal (n=41)
DLBC.Tumor (n=48)
ESCA.Tumor (n=184)
ESCA.Normal (n=11)
GBM.Tumor (n=153)
GBM.Normal (n=5)
HNSC.Tumor (n=520)
HNSC.Normal (n=44)
HNSC−HPV+.Tumor (n=97)
HNSC−HPV−.Tumor (n=421)
KICH.Tumor (n=66)
KICH.Normal (n=25)
KIRC.Tumor (n=533)
KIRC.Normal (n=72)
KIRP.Tumor (n=290)
KIRP.Normal (n=32)
LAML.Tumor (n=173)
LGG.Tumor (n=516)
LIHC.Tumor (n=371)
LIHC.Normal (n=50)
LUAD.Tumor (n=515)
LUAD.Normal (n=59)
LUSC.Tumor (n=501)
LUSC.Normal (n=51)
MESO.Tumor (n=87)
OV.Tumor (n=303)
PAAD.Tumor (n=178)
PAAD.Normal (n=4)
PCPG.Tumor (n=179)
PCPG.Normal (n=3)
PRAD.Tumor (n=497)
PRAD.Normal (n=52)
READ.Tumor (n=166)
READ.Normal (n=10)
SARC.Tumor (n=259)
SKCM.Tumor (n=103)
SKCM.Metastasis (n=368)
STAD.Tumor (n=415)
STAD.Normal (n=35)
TGCT.Tumor (n=150)
THCA.Tumor (n=501)
THCA.Normal (n=59)
THYM.Tumor (n=120)
UCEC.Tumor (n=545)
UCEC.Normal (n=35)
UCS.Tumor (n=57)
UVM.Tumor (n=80)
