# Supplementary figures and images for: Comprehensive Analysis Identified ETV7 as a Potential Prognostic Biomarker in Bladder Cancer
Source: Biomed Res Int. 2021 Dec 9;2021:8530186. doi: 10.1155/2021/8530186 (PMC8678556; doi:10.1155/2021/8530186)

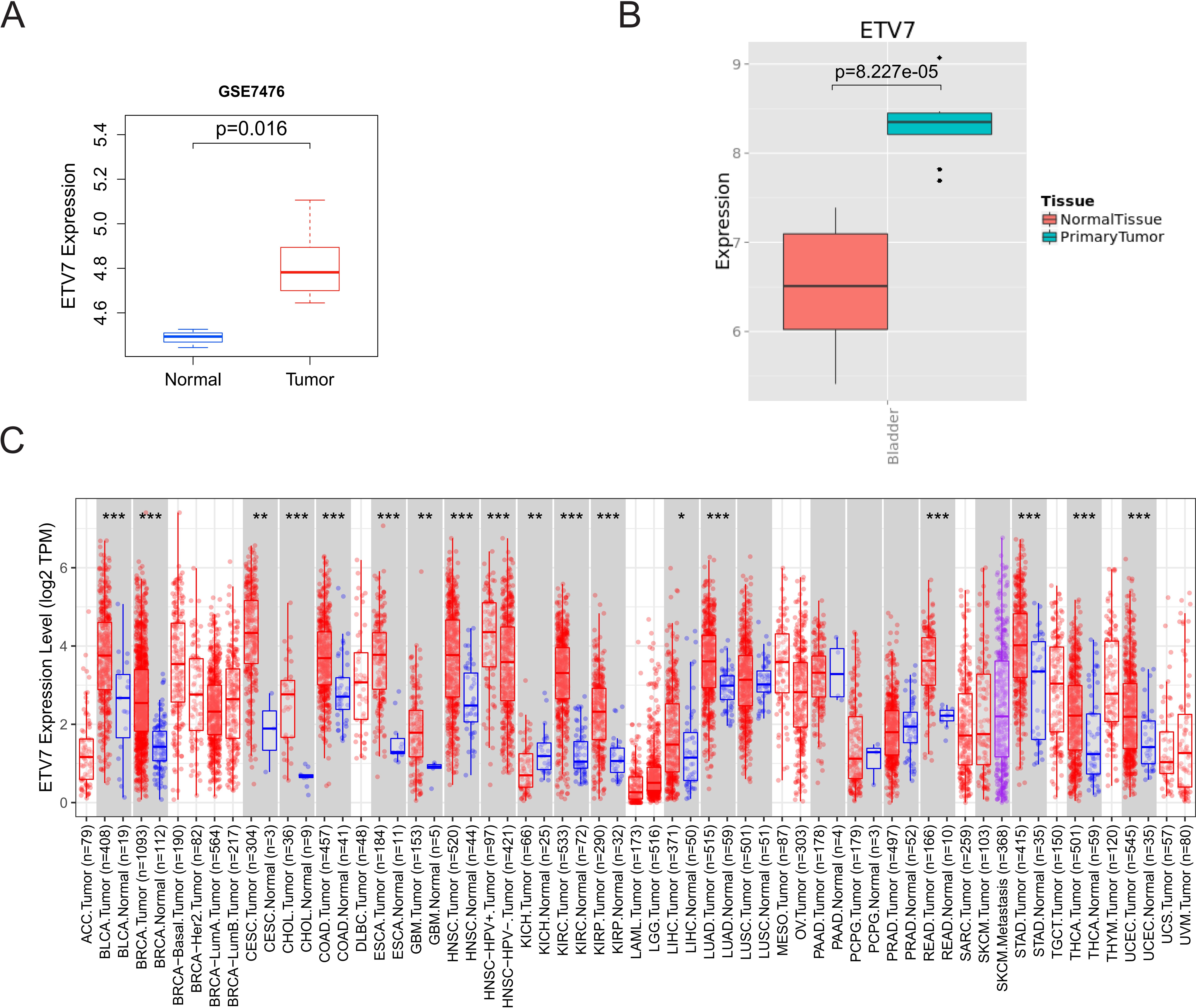

Supplement: Supplementary 2 — Figure S1: supplementary Figure 1: ETV7 expression in multiple database. (a) The differential expression of ETV7 between BLCA and normal tissues in GEO database. (b) ETV7 expression in MERAV database. (c) ETV7 expression levels in different cancer types in the TIMER database. [file 8530186.f2.docx]
